# Mechanisms by Which Increased pH Ameliorates Copper Excess in *Citrus sinensis* Roots: Insight from a Combined Analysis of Physiology, Transcriptome, and Metabolome

**DOI:** 10.3390/plants13213054

**Published:** 2024-10-31

**Authors:** Jiang Zhang, Wei-Lin Huang, Wen-Shu Chen, Rong-Yu Rao, Ning-Wei Lai, Zeng-Rong Huang, Lin-Tong Yang, Li-Song Chen

**Affiliations:** College of Resources and Environment, Fujian Agriculture and Forestry University, Fuzhou 350002, China; 2190807006@fafu.edu.cn (J.Z.); 2210807003@fafu.edu.cn (W.-L.H.); 1220807020@fafu.edu.cn (W.-S.C.); 1220807012@fafu.edu.cn (R.-Y.R.); lainingwei1109@fafu.edu.cn (N.-W.L.); huangzengrong@fafu.edu.cn (Z.-R.H.); talstoy@fafu.edu.cn (L.-T.Y.)

**Keywords:** *Citrus sinensis*, copper–pH interaction, transcriptome, widely targeted metabolome

## Abstract

Limited data are available on copper (Cu)–pH interaction-responsive genes and/or metabolites in plant roots. *Citrus sinensis* seedlings were treated with 300 μM (Cu toxicity) or 0.5 μM (control) CuCl_2_ at pH 3.0 or 4.8 for 17 weeks. Thereafter, gene expression and metabolite profiles were obtained using RNA-Seq and widely targeted metabolome, respectively. Additionally, several related physiological parameters were measured in roots. The results indicated that elevating the pH decreased the toxic effects of Cu on the abundances of secondary metabolites and primary metabolites in roots. This difference was related to the following several factors: (*a*) elevating the pH increased the capacity of Cu-toxic roots to maintain Cu homeostasis by reducing Cu uptake and Cu translocation to young leaves; (*b*) elevating the pH alleviated Cu toxicity-triggered oxidative damage by decreasing reactive oxygen species (ROS) formation and free fatty acid abundances and increasing the ability to detoxify ROS and maintain cell redox homeostasis in roots; and (*c*) increasing the pH prevented root senescence and cell wall (CW) metabolism impairments caused by Cu toxicity by lowering Cu levels in roots and root CWs, thus improving root growth. There were some differences and similarities in Cu–pH interaction-responsive genes and metabolites between leaves and roots.

## 1. Introduction

In plants, micronutrient copper (Cu) functions in a series of physiological processes, including growth, nutrient and water uptake, chlorophyll biosynthesis, photosynthesis, cell wall (CW) formation, nitrogen (N) and carbohydrate metabolisms, and protection against oxidative stress [1,2,3]. Like other heavy metals (HMs), however, Cu can become toxic to plants when it is present in excess [4]. Excess Cu in the soils of some old citrus orchards is a major factor affecting yield and quality due to the extensive and long-term use of Cu-containing fungicides and fertilizers [5,6].

Excessive Cu can impair plant uptake of nutrients and water and inhibit plant growth [7,8]. After exposure to excessive Cu, the inhibition of root growth usually precedes that of shoot growth because most Cu preferentially accumulates in roots [9]. However, the growth of roots is not inherently more sensitive to excess Cu than shoots [10]. Root CWs are the first physical barrier against Cu toxicity. Moreover, these materials can hinder Cu from entering more sensitive targets. Cu toxicity-induced preferential accumulation of Cu in roots is regarded as an adaptive strategy of plants [1,11]. Cu toxicity can also disturb root CW metabolism, thereby reducing root growth [12,13].

Plant Cu tolerance heavily depends on soil acidity (pH) because soil Cu levels decrease with increasing pH [14]. In acidic soils, rhizosphere alkalization induced by roots leads to a decrease in Cu bioavailability, thus reducing plant exposure to Cu [5]. A high pH in the growth matrix can lessen the inhibition of plant growth induced by Cu toxicity [1,7]. Nonetheless, most related studies have focused on investigating the effects of Cu–pH interactions on plant growth; water and nutrient uptake; root exudates and architecture; photosynthesis, pigments, CW components (CWCs), and nonstructural carbohydrates (NCs) in leaves; and hormones and reactive oxygen species (ROS) formation and detoxification in leaves and roots [1,2,15,16].

An integrated analysis of the transcriptome and metabolome provides a powerful approach for revealing the mechanisms underlying plant tolerance to metals, including Cu [12,17,18]. Recently, some researchers have used transcriptome and/or metabolome to investigate Cu toxicity-responsive genes and/or metabolites in plants and have identified some genes [*probable Cu-transporting ATPase HMA5*, *basic helix–loop–helix protein 29* (*bHLH29*), *glutathione S-transferase* (*GST*), *protein FERRIC REDUCTASE DEFECTIVE 3* (*FRD3*), *Cu transporter* (*COPT*) *2* (*COPT2*), *COPT6*, *ZRT/IRT-like protein 1* (*ZIP1*), *iron* (*Fe*) *superoxide dismutase* (*SOD*) *1* (*FSD1*), *Cu/zinc* (*Zn*) *SOD 1* (*CSD1*), *CSD2*, *laccase (LAC) 3* (*LAC3*), and *yellow stripe-like protein 7* (*YSL7*)], as well as metabolites [coumarins, luteolin, lignin, citrate, glucose, salicylic acid (SA), putrescine, reduced glutathione (GSH), aminolevulinic acid (5-ALA), raffinose, and nicotianamine (NA)] and/or metabolic pathways (phenylpropanoid biosynthesis), that are possibly involved in Cu tolerance [12,17,19,20,21,22,23,24]. To our knowledge, there are currently no reports on Cu–pH interaction-responsive genes and/or metabolites in plant roots and/or leaves, except for a report from our laboratory [19]. To conclude, limited reports are available on the molecular mechanisms through which increasing the pH reduces Cu toxicity in higher plants.

In China, the soils of old citrus orchards often have a low pH and high bioavailability of Cu [6,25]. In Pinghe, Fujian, 90.0% and 28.3% of *Citrus grandis* orchards had acidic soils with a pH < 5.0 and excessive soil available Cu (> 6 μg g^−1^ DW), respectively [6]. In China, 49.1% and 42.5% of soils in citrus orchards had a pH < 4.8 and excess in available Cu, respectively [25]. Recently, we examined Cu–pH interaction-responsive genes and metabolites in *Citrus sinensis* leaves via an integrated analysis of physiology, transcriptome, and metabolome, and identified some genes, metabolites, and/or metabolic pathways possibly involved in the increased pH-mediated mitigation of leaf Cu toxicity [19]. In accordance with the recent work, we used RNA-Seq and widely targeted metabolome to examine Cu–pH interaction-responsive genes and metabolites in *C. sinensis* roots. Additionally, we examined the impacts of Cu–pH interactions on root growth; the concentrations of NCs, CW materials (CWMs), CWCs, total free amino acids (TFAAs), and total soluble proteins (TSPs) in roots; and the concentrations of Cu in roots and CWs. The objectives were to uncover the mechanisms of the increased pH-mediated mitigation of Cu toxicity in roots at omics level and the links between transcriptome and metabolome; to identify genes, metabolites, and/or metabolism pathways possibly involved in the raised pH-mediated mitigation of Cu toxicity in roots; and to understand the differences and similarities in Cu–pH interaction-responsive genes and metabolites between roots and leaves.

## 2. Results

### 2.1. Increasing the pH Reduced the Toxic Effects of Cu on Root Growth, the Cu Levels in Roots and CWs, and the TFAA and TSP Levels in Roots

**Figure 1:**
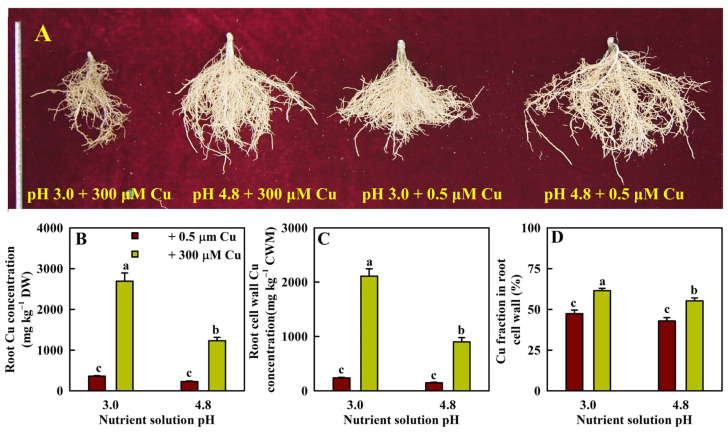
Impacts of Cu–pH interactions on root growth (**A**), as well as the mean (±SE, *n* = 4) Cu concentrations in roots (**B**) and root CWs (**C**) and Cu fraction in root CWs (**D**). Different letters above the bars indicate a significant difference at *p* < 0.05.

Cu toxicity reduced root growth less at pH 4.8 than at pH 3.0. Cu-treated seedlings had sparse, thicker, and darker roots at pH 3.0, but not at pH 4.8 (Figure 1A).

Cu toxicity improved the Cu concentrations in roots and CWs by 651% and 784%, respectively, at pH 3.0 and by 433% and 509%, respectively, at pH 4.8. Because Cu toxicity increased the Cu content [tissue dry weight (DW) × Cu concentration] more in CWs than in roots, the fraction of Cu in CWs in Cu-treated roots increased by 30% at pH 3.0 and 29% at pH 4.8 (Figure 1B–D).

**Figure 2:**
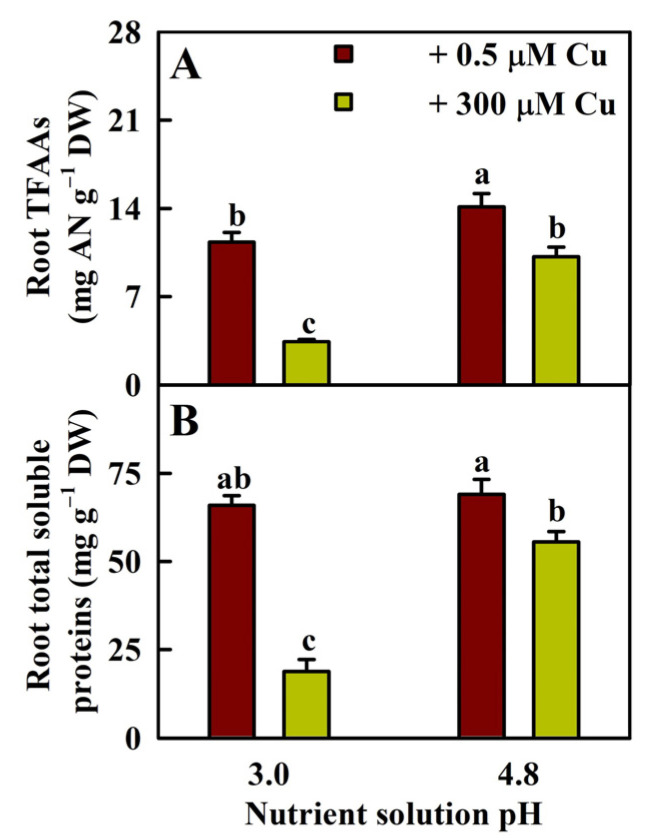
Effects of Cu–pH interactions on the mean (±SE, *n* = 4) concentrations of TFAAs (**A**) and TSPs (**B**) in roots. Different letters above the bars indicate a significant difference at *p* < 0.05. AN, amino nitrogen.

Cu toxicity reduced the levels of TFAAs and TSPs in roots by 70% and 71%, respectively, at pH 3.0 and by 28% and 19%, respectively, at pH 4.8 (Figure 2).

### 2.2. Increasing the pH Reduced Cu Toxicity-Induced Alterations in the Concentrations of NCs, CWMs, and CWCs in Roots

**Figure 3:**
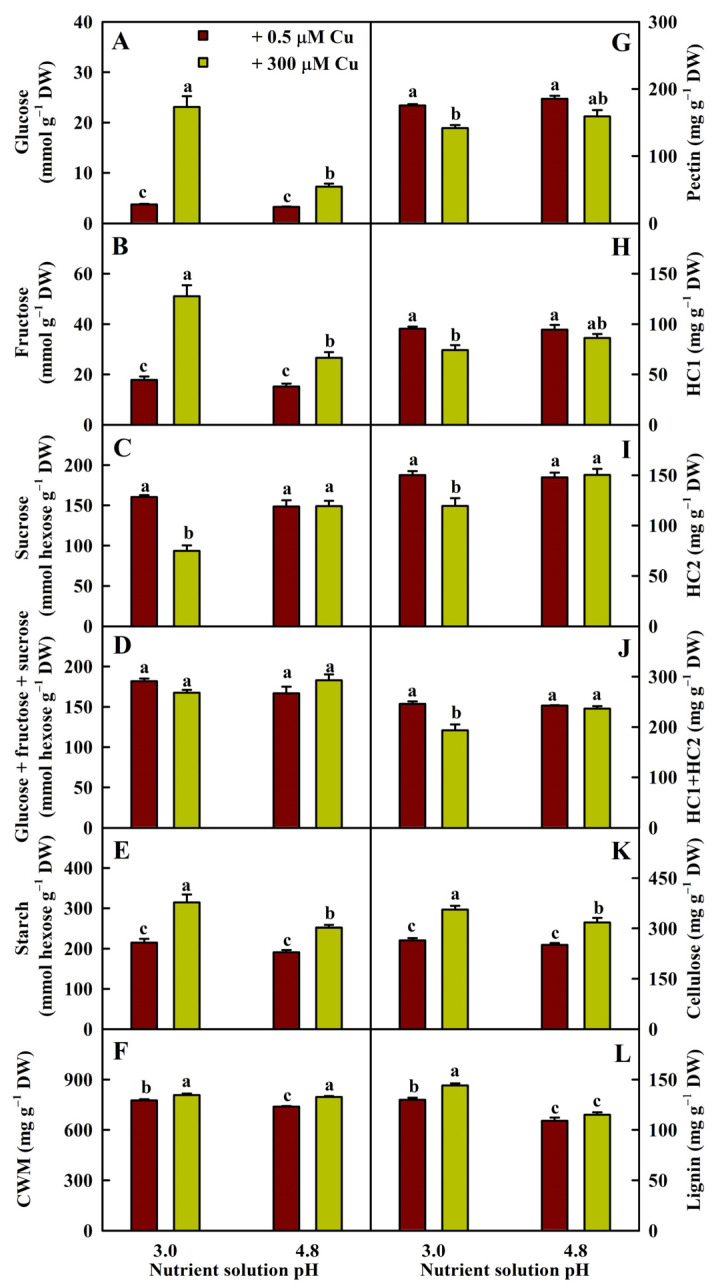
Impacts of Cu–pH interactions on the mean (±SE, *n* = 4) concentrations of glucose (**A**), fructose (**B**), sucrose (**C**), glucose + fructose + sucrose (**D**), starch (**E**), CWMs (**F**), pectin (**G**), HC1 (**H**), HC2 (**I**), HC1 + HC2 (**J**), cellulose (**K**), and lignin (**L**) in roots. HC1, hemicellulose 1; HC2, hemicellulose 2. Different letters above the bars indicate a significant difference at *p* < 0.05.

Cu toxicity increased the glucose, fructose, and starch concentrations in roots more at pH 3.0 than at pH 4.8. Cu toxicity decreased the sucrose concentration in roots at pH 3.0, but not at pH 4.8. Cu toxicity did not significantly affect the glucose + fructose + sucrose concentration in roots (Figure 3A–E).

At pH 3.0, Cu toxicity increased the concentrations of CWMs, cellulose, and lignin and decreased the concentrations of pectin, hemicellulose 1 (HC1), HC2, and HC1 + HC2 in roots. At pH 4.8, Cu toxicity increased the concentrations of CWMs and cellulose, but it had no significant impact on the other five parameters in the roots (Figure 3F–L).

### 2.3. RNA-Seq, Mapping, Transcript Assembly, Functional Annotation, and Differentially Expressed Genes (DEGs) in Roots

**Figure 4:**
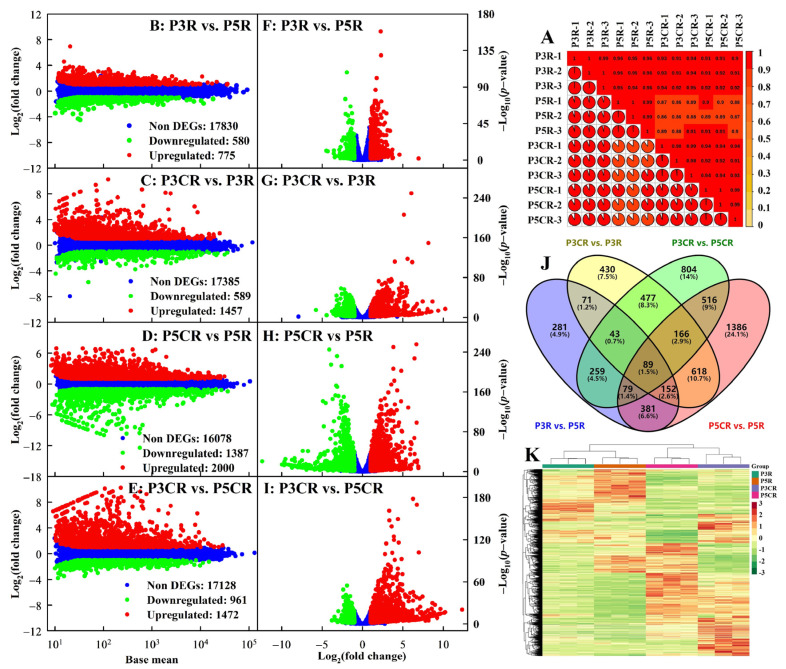
Pearson correlation coefficient (*r*) matrix for P3R, P5R, P3CR, and P5CR (**A**), as well as MA maps (**B**–**E**), volcano plots (**F**–**I**), Venn diagram (**J**), and HCA (**K**) of DEGs identified in R3R vs. P5R, P3CR vs. P3R, P5CR vs. P5R, and P3CR vs. P5CR.

Twelve RNA-Seq libraries constructed from pH 4.8 + 0.5 μM Cu-treated roots (P5R), pH 3.0 + 0.5 μM Cu-treated roots (P3R), pH 4.8 + 300 μM Cu-treated roots (P5CR), and pH 3.0 + 300 μM Cu-treated roots (P3CR) were sequenced, yielding 43,936,214–56,963,646 raw reads, 41,542,740–53,364,872 clean reads, and 6.23–8.00 G clean bases (Table S1). The good correlation (*r* ≥ 0.98) between biological replicates per treatment indicated that the experiment had good reproducibility (Figure 4A). The lower error rate, low quality, and reads containing poly-N and adaptor sequences, as well as the higher clean read number, Q20, and Q30 (Table S1), suggested that the RNA-Seq data were of high quality. A total of 18,730 known and 1392 novel genes were identified in the roots (Table S2).

We obtained 775, 1457, 2000, and 1472 upregulated (580, 589, 1387, and 961 downregulated) genes in P3R vs. P5R, P3CR vs. P3R, P5CR vs. P5R, and P3CR vs. P5CR, respectively (Figure 4B–I). Among the 5752 DEGs isolated in this study, 281, 430, 1386, and 804 DEGs were isolated only from P3R vs. P5R, P3CR vs. P3R, P5CR vs. P5R, and P3CR vs. P5CR, respectively. Only 89 DEGs were simultaneously isolated in the four comparison groups (Figure 4J). Hierarchical cluster analysis (HCA) revealed that the DEGs were highly separated among the four treatments (P3R, P5R, P3CR, and P5CR) but clustered together among the three biological replicates of each treatment (Figure 4K).

As shown in Tables S3 and S4, 701 and 1158 DEGs were enriched in 123 and 136 KEGG pathways, respectively, with 23 and 21 significantly enriched pathways at *p* < 0.05 in P3CR vs. P3R and P5CR vs. P5R, respectively. The top three enriched KEGG pathways were biosynthesis of secondary metabolites (SMs; ko01110), phenylpropanoid biosynthesis (ko00940), and MAPK signaling pathway—plant (ko04016) for P3CR vs. P3R, and phenylpropanoid biosynthesis, biosynthesis of SMs, and flavonoid biosynthesis (ko00941) for P5CR vs. P5R.

In P3CR vs. P3R, 1346, 1240, and 1383 DEGs were enriched in 205 GO terms in cellular component (CC) with 10 significantly enriched GO terms at *p* < 0.05, 1652 GO terms in biological process (BP) with 170 significantly enriched GO terms, and 574 GO terms in molecular function (MF) with 111 significantly enriched GO terms. The top three enriched GO terms were apoplast (GO:0048046), nucleosome (GO:0000786), and protein–DNA complex (GO:0032993) for CC; plant-type hypersensitive response (GO:0009626), host programmed cell death induced by symbiont (GO:0034050), and secondary metabolic process (GO:0019748) for BP; and oxidoreductase activity, oxidizing metal ions (GO:0016722), monooxygenase activity (GO:0004497), and heme binding (GO:0020037) for MF (Table S5).

In P5CR vs. P5R, 2209, 2052, and 2294 DEGs were enriched in 259 GO terms in CC with 13 significantly enriched GO terms, 2007 GO terms in BP with 309 significantly enriched GO terms, and 653 GO terms in MF with 139 significantly enriched GO terms. The top three enriched GO terms were apoplast, intrinsic component of plasma membrane (GO:0031226), and integral component of plasma membrane (GO:0005887) for CC; secondary metabolic process, plant-type secondary CW biogenesis (GO:0009834), and phenylpropanoid metabolic process (GO:0009698) for BP; and oxidoreductase activity, oxidizing metal ions (GO:0016722), heme binding (GO:0020037), and transmembrane signaling receptor activity (GO:0004888) for MF (Table S6).

### 2.4. Validation of qRT-PCR

As shown in Figure S1, the expression patterns of the 32 DEGs obtained by RNA-Seq were well matched with the data obtained by qRT-PCR. A positive and significant correlation existed between the RNA-Seq and qRT-PCR data. Thus, the RNA-Seq data were reliable.

### 2.5. Root Metabolite Profiles and Differentially Abundant Metabolites (DAMs)

As shown in Table S7, 820 metabolites were detected in P5R, P3R, P5CR, and/or P3CR, including (class I) 88 amino acids (AAs) and AA derivatives (AADs), 101 lipids, 60 nucleotides and derivatives (NDs), 64 organic acids (OAs), 53 flavonoids, 161 phenolic acids (PAs), 79 alkaloids, 92 lignans and coumarins, 29 terpenoids, 4 quinones, and 89 other metabolites.

We observed good correlations (*r* ≥ 0.98) between any two biological replicates per treatment (Figure S2A), suggesting that our metabolome data were reliable. Principal component analysis (PCA) and HCA indicated that metabolites were highly separated among the samples—namely, P3R, P5R, P3CR, P5CR, and quality control (QC) sample (Mix)—but clustered together in the three biological replicates of each treatment (Figure S2B,C), suggesting that Cu toxicity and/or low pH had significant influences on metabolite abundances in roots.

**Figure 5:**
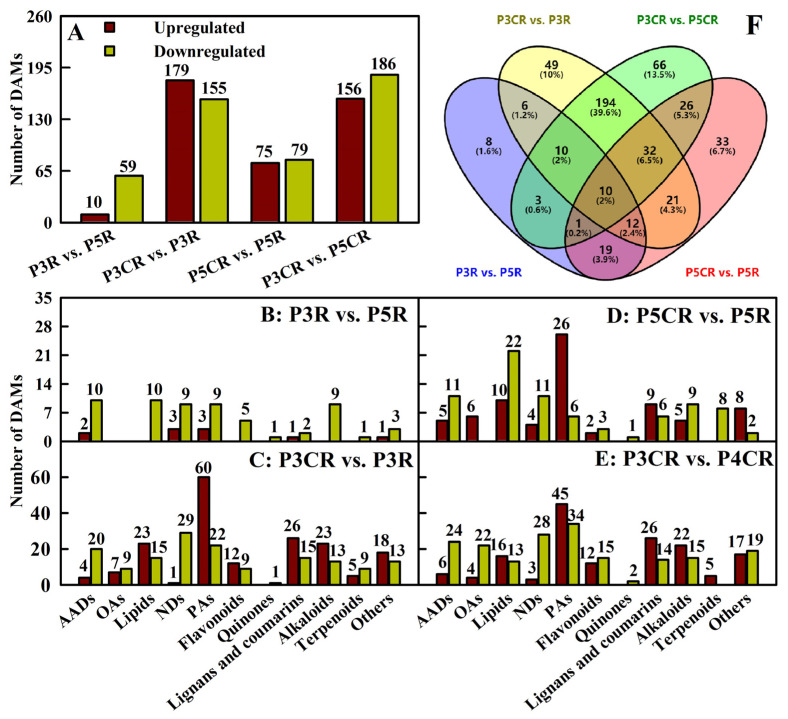
Upregulated and downregulated metabolites (**A**–**E**) and Venn diagram of DAMs (**F**) detected in P3R vs. P5R, P3CR vs. P3R, P5CR vs. P5R, and P3CR vs. P5CR.

The current study detected 10 upregulated [6 primary metabolites (PMs) and 4 SMs] and 59 downregulated (31 PMs and 28 SMs), 179 upregulated (46 PMs and 133 SMs) and 155 downregulated (80 PMs and 75 SMs), 75 upregulated (31 PMs and 44 SMs) and 79 downregulated (45 PMs and 34 SMs), and 156 upregulated (38 PMs and 118 SMs) and 186 downregulated (100 PMs and 86 SMs) metabolites in P3R vs. P5R, P3CR vs. P3R, P5CR vs. P5R, and P3CR vs. P5CR, respectively. These DAMs fell into the following categories (class I): AADs, OAs, lipids, NDs, PAs, flavonoids, quinones, lignans and coumarins, alkaloids, terpenoids, and others (Figure 5 and Table S8). HCA revealed that the DAMs were clustered together in three biological replicates of each treatment but were highly separated between the two treatments in each comparison group (Figure S2D–G).

A total of 490 DAMs were detected in P3R vs. P5R, P3CR vs. P3R, P5CR vs. P5R, and/or P3CR vs. P5CR, while only 10 common DAMs (mws0671, MWS4296, Qmgp082709, Qmgp082711, Qmgp082713, mws0884, Zmnn011624, pmb2940, pmp001270, and mws1409) were detected in the four comparison groups. A total of 8, 49, 33, and 66 DAMs were detected only in P3R vs. P5R, P3CR vs. P3R, P5CR vs. P5R, and P3CR vs. P5CR, respectively (Figure 5B; Table S8).

As shown in Tables S9 and S10, a total of 105 and 40 DAMs were annotated to 75 and 65 KEGG pathways, respectively, with 4 and 6 significantly enriched KEGG pathways at *p* < 0.05 in P3CR vs. P3R and P5CR vs. P5R, respectively. The top three enriched KEGG pathways were purine metabolism (ko00230), biosynthesis of cofactors (ko01240), and nucleotide metabolism (ko01232) for P3CR vs. P3R and carbon fixation in photosynthetic organisms (ko00710), carbapenem biosynthesis (ko00332), and porphyrin metabolism (ko00860) for P5CR vs. P5R.

### 2.6. An Integrated Analysis of Transcriptomic and Metabolomic Data

**Figure 6:**
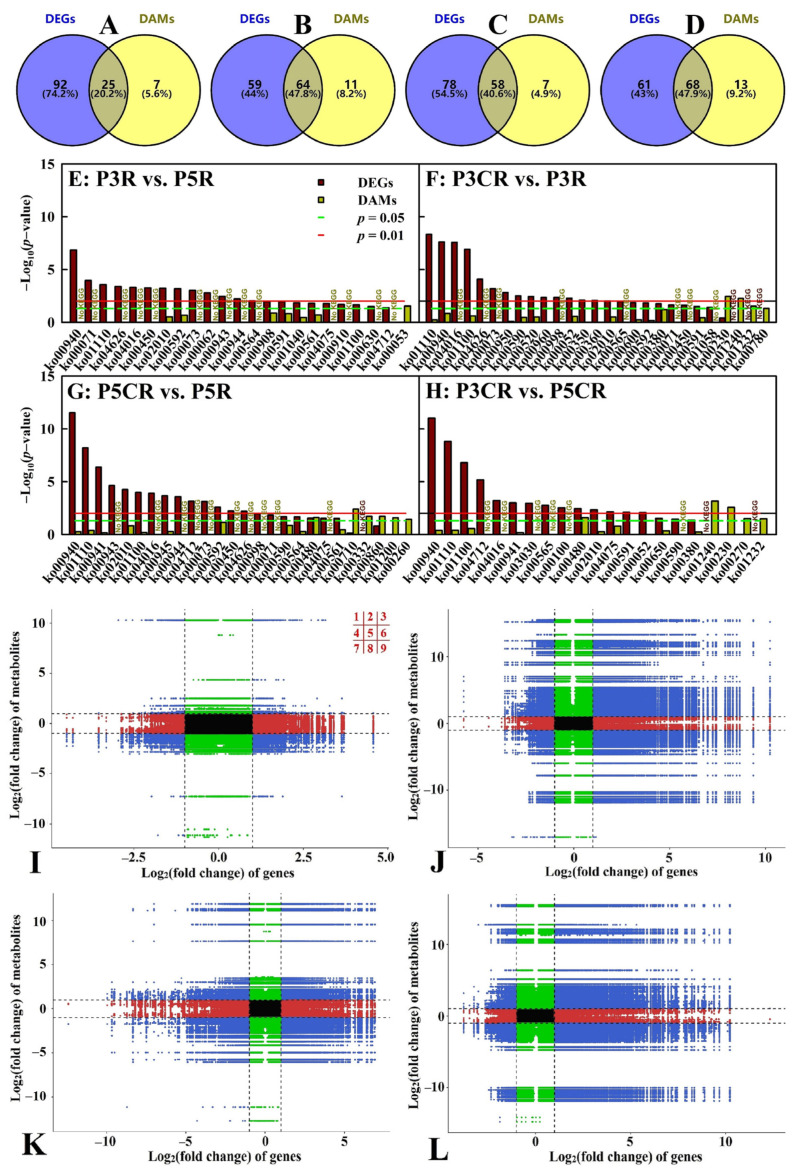
Venn diagrams of all enriched KEGG pathways for DEGs and DAMs (**A**–**D**), statistics of all enriched KEGG pathways for DEGs and/or DAMs with *p* < 0.05 (**E**–**H**), and an overview of transcriptomic and metabolomic variation (**I**–**L**) in P3R vs. P5R (**A**,**E**,**I**), P3CR vs. P3R (**B**,**F**,**J**), P5CR vs. P5R (**C**,**G**,**K**), and P3CR vs. P5CR (**D**,**H**,**L**). For the (**I-L**), black dotted lines represent the threshold values for DEGs and DAMs; within the lines, genes and/or metabolites were not significantly altered; outside of the lines, genes and/or metabolites were significantly altered; blue dots (quadrants 1, 3, 7 and 9) represent both DEGs and DAMs; black dots (quadrant 5) represent unaltered both genes and metabolites; green dots (quadrants 2 and 8) represent DAMs with unaltered genes; and red dots (quadrants 4 and 6) represent DEGs with unaltered metabolites.

The current study obtained more DEGs than DAMs (Figure 4 and Figure 5) and more enriched KEGG pathways for DEGs than for DAMs (Figure 6A–D) for P3R vs. P5R, P3CR vs. P3R, P5CR vs. P5R, and P3CR vs. P5CR. There were 25, 64, 58, and 68 commonly enriched KEGG pathways between DEGs and DAMs and 23, 27, 26, and 21 significantly enriched KEGG pathways, respectively, with *p* < 0.05 for DEGs and/or DAMs in P3R vs. P5R, P3CR vs. P3R, P5CR vs. P5R, and P3CR vs. P5CR, respectively. However, only one common significantly enriched pathway, glutathione metabolism (ko00480), was detected for the DEGs and DAMs in P5CR vs. PCR and P3CR vs. P5CR (Figure 6E–H). Cu–pH interactions had great impacts on the overall transcriptomic and metabolomic variation (Figure 6I–L).

Metabolite–gene networks were constructed to model the synthetic and regulatory characteristics of DEGs and DAMs. Figures S3 and S4 showed the metabolite-gene Pearson correlation network representing DEGs and DAMs related to phenylpropanoid biosynthesis, glutathione metabolism, and cysteine and methionine metabolism (ko00270) in P3CR vs. P3R and P5CR vs. P5R. Many factors associated with phenylpropanoid biosynthesis were activated in P3CR vs. P3R and P5CR vs. P5R, especially in the former.

### 2.7. Comparison of Cu–pH Interaction-Responsive Genes and Metabolites Between Leaves and Roots

In the corresponding comparison group, we obtained more total, upregulated, and downregulated DEGs (DAMs) and more enriched KEGG pathways (GO terms) in roots than in leaves (Figures S5 and S6). This difference might be due to the preferential accumulation of Cu in Cu-exposed roots [7]. There were 6 (1), 76 (10), 37 (2), and 90 (16) common DEGs (DAMs) between P3R vs. P5R and pH 3.0 + 0.5 μM Cu-treated leaves (P3L) vs. pH 4.8 + 0.5 μM Cu-treated leaves (P5L), P3CR vs. P3R and pH 3.0 + 300 μM Cu-treated leaves (P3CL) vs. P3L, P5CR vs. P5R and pH 4.8 + 300 μM Cu-treated leaves (P5CL) vs. P5L, and P3CR vs. P5CR and P3CL vs. P5CL, respectively. Among these common DEGs (DAMs), 2 (0), 30 (5), 25 (1), and 24 (4) DEGs (DAMs) exhibited opposite trends between P3R vs. P5R and P3L vs. P5L, P3CR vs. P3R and P3CL vs. P3L, P5CR vs. P5R and P5CL vs. P5L, and P3CR vs. P5CR and P3CL vs. P5CL, respectively (Figure S5).

There were 28 (6), 85 (31), 46 (18), and 78 (48) commonly enriched KEGG pathways for DEGs (DAMs) between P3R vs. P5R and P3L vs. P5L, P3CR vs. P3R and P3CL vs. P3L, P5CR vs. P5R and P5CL vs. P5L, and P3CR vs. P5CR and P3CL vs. P5CL, respectively. There were 336, 831, 504, and 958 commonly enriched GO terms in BP; 46, 102, 73, and 97 commonly enriched GO terms in CC; and 97, 291, 184, and 306 commonly enriched GO terms in MF between P3R vs. P5R and P3L vs. P5L, P3CR vs. P3R and P3CL vs. P3L, P5CR vs. P5R and P5CL vs. P5L, and P3CR vs. P5CR and P3CL vs. P5CL, respectively (Figure S6).

Additionally, Cu–pH interaction-responsive metabolites and genes exhibited other differences and similarities between leaves and roots. For example, we identified more DEGs in P5CR vs. P5R than in P3CR vs. P3R; more upregulated than downregulated DEGs in P3R vs. P5R, P3CR vs. P3R, P5CR vs. P5R, and P3CR vs. P5CR; and more upregulated than downregulated SMs in P3CR vs. P3R. However, the opposite was true for leaves. More upregulated (downregulated) than downregulated (upregulated) metabolites were detected in P3CR vs. P3R (P3CR vs. P5CR), but fewer upregulated (downregulated) than downregulated (upregulated) metabolites were detected in P3CL vs. P3L (P3CL vs. P5CL) (Figure S5). We detected more decreased than increased free fatty acids (FFAs), glycerol esters, AADs, and NDs in P5CR vs. P5R (Table S8), but only increased AADs and NDs were detected in P5CL vs. P5L [16].

Cu toxicity increased lignin accumulation in root and leaf CWs at pH 3.0 but not at pH 4.8 (Figure 3) [19]. Increasing the pH alleviated Cu toxicity-induced alterations in metabolite abundances, and Cu toxicity increased low–pH interaction-induced alterations in metabolite abundances and gene expression levels in roots and leaves (Figures S5 and S6). We detected more downregulated than upregulated PMs and more upregulated than downregulated SMs in P3CR vs. P5CR and P3CL vs. P5CL (Table S8) [19].

## 3. Discussion

### 3.1. Increasing the pH Conferred Root Cu Tolerance

The current study showed that increasing the pH decreased the toxic effects of Cu on root growth (Figure 1). Additionally, Cu toxicity had a smaller impact on most root parameters at pH 4.8 than at pH 3.0. Indeed, Cu toxicity significantly affected 22 out of 23 parameters in roots at pH 3.0 but only 14 parameters at pH 4.8 (Figure 1, Figure 2 and Figure 3). Our finding that Cu toxicity increased Cu levels in roots and root CWs more strongly at pH 3.0 than at pH 4.8 (Figure 1) might be responsible for the increased pH-mediated amelioration of Cu toxicity. The preferential immobilization of Cu in Cu-exposed root CWs may prevent Cu from entering more sensitive targets, thereby conferring plant Cu tolerance [26]. However, the increased pH-mediated amelioration of Cu toxicity could not be explained in this way alone since the Cu fraction in CWs was greater in P3CR than in P5CR. The increase in Cu immobilization in root CWs agreed with the increased Cu detoxification requirement since P3CR accumulated more Cu than P5CR (Figure 1). Additionally, we detected fewer Cu toxicity-responsive metabolites in roots at pH 4.8 than at pH 3.0 (Figure 5A). Taken together, these findings showed that increasing the pH decreased the toxic effects of Cu on roots. Interestingly, we screened more Cu toxicity-responsive genes in roots at pH 4.8 than at pH 3.0 (Figure 4A), implying that Cu toxicity led to more extensive gene reprogramming in roots at pH 4.8 than at pH 3.0, thus improving root Cu tolerance.

### 3.2. Roots Displayed a Greater Capacity to Maintain Cu Homeostasis at pH 4.8 Than at pH 3.0

In addition to improving Cu immobilization in root CWs, plants have developed different strategies to maintain Cu homeostasis [3,9,12]. In *Arabidopsis*, AtHMA5 has a role in excess Cu export from the cytosol across the plasma membrane (PM) and in loading Cu into the xylem for root-to-shoot Cu translocation or Cu detoxification in roots [27,28]. Li et al. [29] reported that Cu hypertolerance in *Silene vulgaris* was correlated with the upregulated expression of two HMA5 paralogs, *SvHMA5I* and *SvHMA5II*. Overexpression of one of the two genes conferred Cu tolerance in *Arabidopsis.* Shi et al. [30] reported that apple calli transformed with apple *HMA5 (MdHMA5)*-RNAi or overexpressing *MdHMA5* had decreased or increased Cu tolerance, respectively, accompanied by increased or reduced Cu concentrations in calli. In *Arabidopsis*, AtATX1 plays a vital role in Cu homeostasis by delivering Cu to AtHMA5 [31]. Dai et al. [32] showed that overexpression of the peanut YSL gene (*AhYSL3.1*) conferred Cu tolerance to tobacco and rice, accompanied by a reduction in the Cu concentration in Cu-exposed young leaves. Zn supplementation mitigated Cu toxicity in oat (*Avena sativa*), lettuce (*Lactuca sativa*) [33], and duckweed (*Spirodela polyrhiza*) [34] by decreasing Cu uptake. The upregulation of *ZIP1* (orange1.1t03274 and orange1.1t03275) in P5CR vs. P5R implied that increasing the pH improved Zn uptake, thus reducing Cu uptake in Cu-exposed seedlings and conferring citrus Cu tolerance [7]. Overall, we isolated 22 (30) upregulated and 5 (9) downregulated genes involved in Cu homeostasis in P3CR vs. P3R (P5CR vs. P5R) (Table S11). Increasing the pH improved the capacity of Cu-exposed roots to maintain Cu homeostasis (Figure 1), which could be explained at least partially by the upregulation of *HMA5* (Cs5g03780, Cs5g03790, and Cs5g03800), *YSL1* (Cs5g01560; the homolog of *Arabidopsis YSL3*), and *ZIP1* in P5CR vs. P5R and the downregulation of the *Cu transport protein ATX1* (Cs2g08850) in P3CR vs. P3R.

### 3.3. Increasing the pH Mitigated Cu Toxicity-Induced CW Impairment and Senescence of Roots

We found that Cu toxicity improved the CWM concentration in roots, thus increasing the Cu fraction in root CWs (Figure 1). The increase in the Cu fraction in Cu-exposed root CWs was consistent with the increased demand for Cu immobilization in CWs and hindered the entry of Cu into the more sensitive cytoplasm [1]. The Cu toxicity-induced increase in the CWM concentration was mainly due to elevated cellulose and lignin levels in P3CR vs. P3R and increased cellulose levels in P5CR vs. P5R (Figure 3). Our results suggested that the Cu toxicity-induced upregulation of cellulose resulted from elevated biosynthesis and decreased degradation in P3CR vs. P3R and from increased biosynthesis in P5CR vs. P5R (Table S12). The greater accumulation of cellulose in P3CR vs. P3R than in P5CR vs. P5R might be caused by decreased degradation and decreased dilution due to reduced growth because Cu toxicity decreased root growth more at pH 3.0 than at pH 4.8 (Figure 1). Cellulose synthase (CesA) and cellulose synthase-like (Csl) proteins are involved in the biosynthesis of cellulose and most hemicellulosic polysaccharides [35]. Transgenic *Arabidopsis* plants overexpressing CesA6-like genes displayed increased cellulose concentrations and CW thicknesses [36]. TRICHOME BIREFRINGENCE is necessary for the biosynthesis of cellulose in *Arabidopsis* [37]. Endoglucanase plays a role in cellulose hydrolysis [38]. The upregulation of CesA (Cs2g04590, Cs4g01990, and Cs5g29200), Csl (Cs5g01970, Cs9g08730, and Cs9g08760), and protein trichome birefringence-like (Cs6g08690) genes and the downregulation of *endoglucanase 8* (Cs2g17090) in P3CR vs. P3R and the upregulation of CesA (Cs2g04590, Cs4g01990, and Cs5g29200), Csl (Cs3g09000, Cs4g08560, Cs5g25090, Cs9g08730, Cs9g08750, and Cs9g08760), and protein trichome birefringence-like (Cs2g15690 and Cs6g08690) genes in P5CR vs. P5R might contribute to the Cu toxicity-induced accumulation of cellulose in roots, thereby increasing CW thickness.

In *Elsholtzia splendens*, the higher Cu adsorption capacity of pectin in CWs has been shown to be responsible for the greater Cu tolerance [11]. Silencing of a rice wall-associated kinase gene (*OsWAK11*) by RNAi decreased Cu tolerance by reducing Cu accumulation in CW pectin and hemicellulose due to an enhanced degree of pectin methyl esterification (DPM) and an elevated Cu accumulation in the cytoplasm [39]. DPM in *Arabidopsis* was negatively correlated with the activity of pectin methylesterase (PME) [40]. We isolated one upregulated (orange1.1t00214) and three downregulated (Cs9g14450, Cs5g33410, and orange1.1t01727) *PMEs* in P3CR vs. P3R and four upregulated (Cs1g16560, Cs2g16380, Cs6g11440, and orange1.1t01085) and one downregulated (Cs4g06670) *PME* in P5CR vs. P5R (Table S12), implying that Cu toxicity improved and lessened DPM in P3CR vs. P3R and P5CR vs. P5R, respectively. Colzi et al. observed that in *Silene paradoxa*, the tolerant population had a greater DPM than the sensitive population, which might reduce Cu binding to the root CWs, thereby ensuring a low concentration of apoplastic Cu and possibly reducing symplastic Cu uptake by root cells [41]. Taken together, these findings showed that Cu toxicity led to reduced pectin and hemicellulose concentrations (Figure 3G–J) and elevated pectin methyl esterification in roots treated with a pH of 3.0, thus decreasing Cu tolerance.

Overexpression of two maize R2R3-MYB transcription factor genes (*ZmMYB31* and *ZmMYB42*) in *A. thaliana* and maize led to a decrease in lignin concentration in transgenic plants due to downregulated expression of caffeic acid 3-O-methyltransferase (COMT) genes [42]. Cinnamoyl CoA reductase (CCR) is involved in regulating carbon flux into lignin. Transgenic tobacco plants with greatly reduced CCR activity displayed a strong decrease in lignin concentration [43]. The lignin concentration was reduced by 15% in *shikimate O-hydroxycinnamoyltransferase* (*HCT*)-silenced *Nicotiana benthamiana* stems [44]. 4-Coumarate:CoA ligase (4CL) plays a vital role in controlling carbon flow into different branch pathways of phenylpropanoid metabolism. At4CL1 and At4CL2 function in the biosynthesis of lignin and in the formation of phenolic compounds other than flavonoids [45]. The lignin concentration was higher in transgenic soybean lines overexpressing the cinnamyl alcohol dehydrogenase (CAD) gene from wild soybean than in wild-type plants [46]. In plants, LAC participates in the formation of lignin polymers. Upregulation of *CsiLAC4* in *C. sinensis* led to an increase in the level of lignin in the xylem CWs [47]. Peroxidase (PER) catalyzes the final step in lignin biosynthesis. A transgenic carrot line overexpressing a rice cationic peroxidase gene (*OsPrx114*) exhibited increased lignin biosynthesis in the outer periderm tissues of tap roots [48]. Thus, the upregulation of sixteen COMT, three CCR, five HCT, one 4CL, seventeen CAD, twelve LAC, and four PER genes in P3CR vs. P3R might contribute to the Cu toxicity-induced accumulation of lignin in pH 3.0-treated roots (Figure 3L and Table S12). The increased accumulation of lignin in P3CR vs. P3R could also be caused by decreased dilution due to decreased root growth (Figure 1). Additionally, we screened three (six) upregulated and one (five) downregulated gene related to CW thickening (GO:0052386) in P3CR vs. P3R (P5CR vs. P5R) (Table S12). These results suggested that Cu toxicity led to increased CW thickness and hardness, thus inhibiting root growth at pH 3.0 but not at pH 4.8 (Figure 1) [49].

Zhu et al. [50] reported that a *xyloglucan endotransglucosylase/hydrolase protein 31* (*XTH31)* T-DNA insertional mutant of *Arabidopsis* had reduced root growth and hemicellulose concentration. As shown in Table S12, we identified eight upregulated and one downregulated *XTH* in P5CR vs. P5R and two upregulated and one downregulated *XTH* in P3CR vs. P3R. The upregulation of *XTHs* in P5CR vs. P5R might contribute to the elevated pH-mediated alleviation of root growth inhibition and hemicellulose reduction caused by Cu toxicity.

To conclude, we obtained more DEGs related to CW metabolism in P5CR vs. P5R than in P3CR vs. P3R (Table S12). Increasing the pH might alleviate the toxic effects of Cu on root CW and growth by reprogramming genes related to CW metabolism more extensively. These findings agreed with our result that Cu toxicity might accelerate root senescence at pH 3.0 but not at pH 4.8 (Table S13).

### 3.4. Increasing the pH Mitigated the Toxic Effects of Cu on Primary Metabolism in Roots

The effects of Cu toxicity on the concentrations of NCs (Figure 3A–E) and the abundances of OAs, saccharides, and alcohols (Table S8) in roots were greater at 3.0 than at pH 4.8. However, we obtained 33 (44) upregulated and 18 (35) downregulated genes related to starch and sucrose metabolism (ko00500), tricarboxylic acid (TCA) cycle (ko00020), glycolysis/gluconeogenesis (ko00010), pyruvate metabolism (ko00620), and pentose phosphate (Pi) pathway (PPP, ko00030) in P3CR vs. P3R (P5CR vs. P5R) (Table S14). Our findings indicated that increasing the pH prevented Cu toxicity-triggered alterations in carbohydrate abundances in roots by reprogramming genes related to carbohydrate metabolism more extensively.

Stressed plants often suffer from energy deficit. Carbohydrates can serve as energy sources and antioxidants [22]. Yusuf et al. [51] reported that glucose-mediated alleviation of cucumber Cu toxicity involved improved nutrient uptake and reduced oxidative damage. TCA cycle participates in the oxidation of respiratory substrates to drive ATP biosynthesis. PPP can provide NADPH to yield GSH and ascorbate (ASC), which are involved in ROS scavenging [52]. Additionally, we identified eleven (three) upregulated and four (eight) downregulated genes in P3CR vs. P3R (P5CR vs. P5R) involved in oxidative phosphorylation (ko00190) and ATP biosynthetic process (GO:0006754) (Table S14). Taken together, these findings suggested that energy (ATP) formation might be increased in P3CR vs. P3R but not in P5CR vs. P5R. The greater increase in total NCs (Figure 3 and Table S8) and ATP production in P3CR vs. P3R was consistent with the increased need for energy and ROS removal.

Cu-triggered release of OAs by roots is considered an adaptive mechanism of plants to Cu toxicity by boosting Cu immobilization in roots and lowering Cu uptake and phytotoxicity [5,53]. In *C. sinensis* seedlings, Yang et al. [54] indicated that increasing the pH boosted the levels of OAs in roots and subsequently promoted their release by roots, thus alleviating aluminum (Al) toxicity. The increased accumulation of OAs in P5CR vs. P5R implied that increasing the pH might increase the Cu toxicity-induced exudation of OAs by roots, thereby mitigating Cu toxicity.

Macromolecules in senescing organs are degraded, and nutrients are redistributed to nutrient-demanding young organs. We found that excess Cu upregulated the degradation of N-containing compounds in roots at pH 3.0 but less at pH 4.8 (Table S15). These findings agreed with the results that 41 (24) upregulated and 76 (35) downregulated N-containing compounds were identified in P3CR vs. P3R (P5CR vs. P5R) (Table S16). The downregulation of N compounds in P3CR vs. P3R and P5CR vs. P5R implied that Cu toxicity improved N remobilization efficiency (NRE) to address Cu toxicity-induced N reduction, especially at pH 3.0 [7]. Our results suggested that increasing the pH prevented Cu toxicity-induced increases in protein and AA degradation and decreases in protein and AA biosynthesis (Table S15), thereby improving protein and AA levels in roots (Figure 2B and Table S8). In conclusion, Cu toxicity increased NRE in roots to cope with the Cu toxicity-induced reduction in N uptake, especially at low pH. An increase in pH increased the levels of N, proteins, and AAs in Cu-exposed roots.

Our results suggested that the upregulation of FFAs in P3CR vs. P3R [sixteen upregulated (five saturated + eleven unsaturated) FAs and five downregulated unsaturated FAs; Table S8] might be caused by decreased dilution due to reduced growth (Figure 1) and/or increased biosynthesis, as indicated by eleven upregulated and nine downregulated genes involved in FA biosynthetic process (GO:0006633; Table S17), and that the downregulation of unsaturated FAs in P5CR vs. P5R [four upregulated (three saturated + one unsaturated) FAs and twelve downregulated unsaturated FAs; Table S8] might result from reduced biosynthesis, as indicated by four downregulated genes involved in unsaturated FA biosynthetic process (GO:0006636) and elevated degradation (oxidation), as indicated by fifteen upregulated and three downregulated genes involved in FA degradation (ko00071) and six upregulated and three downregulated genes involved in FA oxidation (GO:0019395; Table S17). Additionally, we detected one upregulated and one downregulated glycerol ester and six upregulated (five lysophosphatidylcholines (LysoPCs) and one lysophosphatidylethanolamine (LysoPE)) and nine downregulated (seven LysoPCs + two LysoPEs) phospholipids (PLs) in P3CR vs. P3R, and one upregulated and seven downregulated glycerol esters and five upregulated and three downregulated LysoPCs in P5CR vs. P5R (Table S8). The downregulation of glycerol ester in P5CR vs. P5R was caused by increased oxidation, as indicated by six upregulated and four downregulated genes involved in lipid oxidation (GO:0034440; Table S17), while the downregulation of PLs in P3CR vs. P3R was caused by increased catabolism (oxidation), as indicated by eighteen upregulated and four downregulated genes involved in lipid catabolic process (GO:0016042) and three upregulated and one downregulated gene involved in lipid oxidation (GO:0034440; Table S17). Lipid peroxidation is partly due to an increase in the FFA substrate for lipoxygenase [55]. The increased pH-mediated alleviation of oxidative damage in Cu-exposed roots [2] might involve reduced FFA accumulation.

Cu toxicity reduced the P level in *C. sinensis* roots, and the P level in Cu-exposed roots was greater at pH 4.8 than at pH 3.0 [7]. In plants, P remobilization of organic P (PLs) contributes to the maintenance of P homeostasis [56]. As shown in Tables S8 and S16, we detected fewer upregulated than downregulated P compounds (PLs) in P3CR vs. P3R, but the reverse was the case in P5CR vs. P5R, suggesting that more organic P was converted into available Pi to maintain Pi homeostasis in Cu-exposed roots at pH 3.0 but not at pH 4.8. These findings agreed with the report that the P level in Cu-toxic roots was much lower at pH 3.0 than at pH 4.8 [7].

Our results indicated that elevating the pH decreased the Cu toxicity-induced decreases in NDs (Table S8). Interestingly, we identified more upregulated genes than downregulated genes or similar upregulated and downregulated genes involved in nucleotide biosynthetic process (GO:0009165) but fewer upregulated genes than downregulated genes or similar upregulated and downregulated genes involved in nucleotide catabolic process (GO:0009166) in P3CR vs. P3R and P5CR vs. P5R (Table S18). These findings suggested that Cu toxicity-induced alterations in ND abundances could not be regulated at the transcriptional level.

### 3.5. Increasing the pH Decreased the Toxic Effects of Cu on SMs in Roots

Our results indicated that increasing the pH mitigated the increase in the biosynthesis and abundances of root SMs, phenylpropanoids, phenolic compounds, and lignans and coumarins caused by Cu toxicity (Figure S3; Tables S8 and S19–S21). Notably, SA was upregulated in P3CR vs. P3R but not in P5CR vs. P5R (Table S8). This finding agreed with the result obtained by targeted metabolomics, in which the level of SA was upregulated in P3CR vs. P3R but not in P5CR vs. P5R [15]. Zhang et al. reported that the overexpression of *protein SAR DEFICIENT 1* (*SARD1*) led to increased levels of free SA and total SA in *Arabidopsis*, suggesting that *SARD1* plays a key role in SA biosynthesis by regulating the expression of *SID2*, which encodes isochorismate synthase [57]. The upregulation of SA in P3CR vs. P3R might be caused by increased biosynthesis, as indicated by upregulated *SARD1* (Cs7g27120) involved in SA biosynthetic process (GO:0009697; Table S19), and decreased dilution due to reduced growth (Figure 1), rather than by reduced catabolism, as indicated by five upregulated genes involved in SA catabolic process (GO:0046244; Table S19). Additionally, we identified 21 (16) upregulated and 4 (6) downregulated genes involved in the biosynthesis of various alkaloids (ko00960, ko00950, and ko0090; Table S19) and 23 (5) upregulated and 13 (9) downregulated alkaloids in P3CR vs. P3R (P5CR vs. P5R) (Table S8), suggesting that Cu toxicity increased the biosynthesis of alkaloids and their accumulation in P3CR vs. P3R but not in P5CR vs. P5R. A Cu toxicity-induced increase in alkaloids has been observed in *C. grandis* roots [12]. Similarly, increasing the pH decreased the toxic effects of Cu on terpenoids, as indicated by five (zero) upregulated and nine (eight) downregulated terpenoids in P3CR vs. P3R (P5CR vs. P5R) (Table S8). The downregulation of terpenoids might be caused by increased degradation in P3CR vs. P3R, as indicated by 2 upregulated genes involved in terpenoid catabolic process (GO:0016115; Table S19), and by decreased biosynthesis in P5CR vs. P5R, as indicated by 17 upregulated and 21 downregulated genes involved in terpenoid biosynthetic process (GO:0016114; Table S19).

In addition to scavenging ROS, SMs participate in the detoxification of Cu toxicity through the chelation (immobilization) of Cu [58]. The endophytic *Bacillus altitudinis* WR10 enhanced wheat tolerance to excess Cu by upregulating phenylpropanoid biosynthesis and antioxidant capacity and reducing H_2_O_2_ accumulation in roots [4]. Increased accumulation of lignin in roots leads to increased CW thickness, thus forming a physical barrier to hinder the adverse impacts of excess Cu. Overexpression of *caffeoyl-CoA O-methyltransferase* (*CCoAOMT*), *LAC10*, and *peroxidase 7* conferred Cu tolerance on rice [59], tobacco [60], and *Arabidopsis* [61], respectively, by improving the immobilization of Cu in CWs and/or reducing Cu uptake under Cu toxicity due to increased lignin biosynthesis and accumulation. Mostofa and Fujita [62] indicated that SA-mediated alleviation of rice Cu toxicity involved increased immobilization of Cu in roots and decreased oxidative damage in leaves and roots. Chen et al. [63] reported that exogenous putrescine enhanced *Populus cathayana* tolerance to Cu toxicity by reducing foliar Cu concentrations and oxidative damage. Shad et al. [64] reported that exogenous coumarin alleviated manganese (Mn) toxicity-induced decrease in growth in *Sesamum indicum* plants by reducing Mn concentrations, electrolyte leakage, and the accumulation of ROS and MDA in roots and leaves. The foliar application of betaine increased maize tolerance to cadmium (Cd) toxicity by repressing Cd uptake and translocation to shoots and mitigating oxidative damage in roots and shoots [65]. A recent study suggested that the Cu-induced release of phenolic compounds was involved in *C. sinensis* Cu tolerance [5]. The increased accumulation of SMs implied that Cu toxicity might trigger the release of SMs by roots, especially at low pH.

Taken together, these results indicated that the observed greater accumulation of SMs, especially PAs (SA), alkaloids (betaine and putrescine), lignin, and coumarins, in P3CR vs. P3R might be an adaptive strategy to Cu toxicity to cope with the elevated need for Cu and ROS detoxification. Increasing the pH decreased the Cu toxicity-induced alterations of SMs in the roots.

### 3.6. Increasing the pH Mitigated Cu Toxicity-Induced Oxidative Damage in Roots

Fe is a component of several antioxidant enzymes, including Fe-SOD, catalase (CAT), and ASC peroxidase (APX). Fe deficiency increases plant vulnerability to oxidative damage [66]. A study showed that Cu toxicity reduced Fe uptake by roots and root-to-shoot Fe translocation in citrus seedlings [67]. Transcription factor FER-LIKE IRON DEFICIENCY-INDUCED TRANSCRIPTION FACTOR (FIT or BHLH29) plays a vital role in controlling Fe uptake. In *Arabidopsis*, Fe levels in roots and shoots were lower in *fit1* mutant plants than in wild-type plants [68]. Durrett et al. [69] indicated that FRD3-mediated transport of citrate into the root vasculature was key for the translocation of Fe to shoots and that overexpression of *FRD3* conferred *Arabidopsis* Al tolerance by increasing Al-stimulated secretion of citrate by roots. The upregulation of *FIT* (Cs8g15600) and *FRD3* (Cs7g01770) in P5CR vs. P5R agreed with our reports that under Cu toxicity, increasing the pH improved Fe uptake and translocation to shoots [7], as well as the activities of SOD, CAT, and APX in roots [2]; our findings that Cu toxicity downregulated the expression of *SOD [Fe] chloroplastic* (*FSD*; Cs7g19250) in roots at pH 3.0 but not at pH 4.8; and the above inference that increasing the pH improved Cu toxicity-stimulated exudation of OAs. Protein disulfide isomerase (PDI) and protein disulfide isomerase-like protein (PDIL) catalyze the reduction, formation, or isomerization of disulfide bonds. PDI also has Cu-binding activity. Overexpression of a PDIL gene conferred rice mercury (Hg) tolerance through alleviating Hg-induced oxidative damage due to reduced ROS formation and elevated activities of antioxidant enzymes, concentrations of GSH, and ratios of GSH/oxiglutathione (GSSG) in roots and leaves [70]. The upregulation of *protein disulfide-isomerase 5-4* (orange1.1t04754) in P5CR vs. P5R agreed with our finding that GSH abundance was reduced in P3CR vs. P3R but not in P5CR vs. P5R (Table S8). Overexpression of an *Arabidopsis Nudix hydrolase* (*AtNUDX2*) conferred tolerance to oxidative stress in stressed *Arabidopsis* cells [71]. A study showed that increasing the pH alleviated excess Cu-induced oxidative damage and decrease in the GSH/GSSG ratio by decreasing the ROS formation and improving the antioxidant capacity of leaves and roots [2]. Therefore, the upregulation of *protein disulfide-isomerase 5-4*, *NUDX2* (Cs2g19230), *FRD3*, and *FIT* in P5CR vs. P5R (Table S22) might confer citrus Cu tolerance by reducing Cu uptake, phytotoxicity, and translocation to shoots; increasing Fe uptake and translocation to shoots; and alleviating Cu toxicity-induced oxidative damage in roots. GSTs are involved in the quenching of reactive molecules by the addition of GSH and the detoxification of Cu. Overexpression of *GST* (*NT107*) conferred Cu tolerance to *Dianthus superplants* by increasing phytochelatin (PC) biosynthesis and Cu accumulation. A higher level of PC biosynthesis might contribute to the sequestration and detoxification of excess Cu [72]. Overexpression of *GST/glutathione peroxidase* enhanced the growth of transgenic tobacco plants under stress but decreased the GSH/GSSG ratio [73]. We identified ten (nine) upregulated and two (twelve) downregulated genes related to the GST activity in P3CR vs. P3R (P5CR vs. P5R) (Table S22), which agreed with our findings that the GSH/GSSG ratio was decreased in P3CR vs. P3R but not in P5CR vs. P3R (Table S8) and our previous report that Cu toxicity increased the root PC concentration more at pH 3.0 than at pH 4.8 and that Cu toxicity reduced the root GSH/GSSG ratio at pH 3.0 but not at pH 4.8 [2]. The upregulation of *GSTs* in P3CR vs. P3R agreed with the increased need for Cu sequestration and detoxification (Figure 1B).

Through the biosynthesis of sulfur (S)-containing compounds (GSH and cysteine), S metabolism plays a vital role in the tolerance of plants to Cu toxicity and oxidative stress [2,74]. We found that increasing the pH alleviated the Cu toxicity-induced reduction in S compounds (Table S16), thereby conferring root Cu tolerance. This agreed with the report that increasing the pH mitigated the Cu toxicity-induced decrease in the activities of eight S metabolism-related enzymes and the concentration of GSH in roots [2]. Notably, L-cysteine was downregulated in P5CR vs. P5R but unaltered in P3CR vs. P3R (Table S8). In plants, cysteine synthase (CS) catalyzes the final step in the biosynthesis of L-cysteine, a precursor for the biosynthesis of different S-containing compounds. Transgenic soybean plants overexpressing *CS* displayed increased accumulation of cysteine in seeds [75]. Choi et al. indicated that CS could protect plants against toxic O_3_ gas, probably by overaccumulating S-rich antioxidants (cysteine and GSH) [76]. A study from our laboratory indicated that CS activity was reduced in P3CR vs. P3R but not in P5CR vs. P5R and that CS activity in Cu-exposed roots decreased with decreasing pH [2]. These findings suggested that cysteine biosynthesis might decrease and increase in P3CR vs. P3R and P5CR vs. P5R, respectively. Thus, the decrease in cysteine in P5CR vs. P5R was caused by increased utilization due to increased biosynthesis of the other S-containing compounds rather than by decreased biosynthesis, while the unaltered cysteine level in P3CR vs. P3R might be caused by decreased utilization due to decreased biosynthesis of the other S-containing compounds and/or decreased dilution due to decreased growth (Figure 1). Interestingly, we identified one (Cs3g11490) and two (Cs3g11490 and Cs3g11520) upregulated CS genes in P3CR vs. P3R and P5CR vs. P5R, respectively (Table S22), implying that posttranscriptional regulation might influence CS activity in roots.

Vitamins have good antioxidant potential. We detected three upregulated (acitretin, N-(β-D-glucosyl) nicotinate, and pyridoxine-5′-phosphate) and four downregulated (nicotinamide, D-pantothenic acid, biotin, and pyridoxal) vitamins in P3CR vs. P3R and one downregulated (biotin) vitamin in P5CR vs. P5R (Table S8). A study showed that Cu toxicity increased the ASC and dehydroascorbate (DHA) concentrations and had no significant impact on the ASC/DHA ratio in roots at pH 4.8, but it decreased these three parameters at pH 3.0 [2]. Obviously, increasing the pH prevented the toxic effects of Cu on ASC and other vitamin metabolisms, thereby maintaining vitamin homeostasis. Tahjib-Ul-Arif et al. [74] reported that GSH and ASC lessened the toxic effects of Cu on rice growth and photosynthetic pigments by increasing N and water uptake and lowering Cu uptake and oxidative damage.

In conclusion, increasing the pH caused more extensive reprogramming of genes related to ROS detoxification and cell redox homeostasis in Cu-exposed roots (Table S22) to cope with oxidative stress. The increased pH-mediated alleviation of oxidative damage in Cu-exposed roots involved reduced Cu accumulation and ROS production and an enhanced ability to detoxify ROS and maintain cell redox homeostasis.

## 4. Materials and Methods

### 4.1. Plant Materials

The culture and Cu–pH treatments of ‘Xuegan’ [*Citrus sinensis* (L.) Osbeck] seedlings were performed according to the previous methods [19]. Six weeks after seed germination, uniform seedlings were transferred to 6 L plots (two plants per pot) containing sand. Seedlings were cultivated in a greenhouse under natural conditions at Fujian Agriculture and Forestry University, Fuzhou, China (119°14′ E, 26°5′ N). Starting from the seventh week after transplanting, each pot was supplied six times weekly with ~500 mL of freshly prepared nutrient solution at a CuCl_2_ concentration of 300 μM (Cu toxicity) or 0.5 μM (control or non-Cu toxicity) and a pH of 4.8 (high) or 3.0 (low) until leaking. The nutrient solution was adjusted to pH 4.8 or 3.0 with 1 M HCl. In this study, 300 μM Cu was used as the Cu toxicity treatment because it led to a significant but not excessive inhibition of ‘Xuegan’ seedling growth at pH 3.0, while it did not significantly inhibit seedling growth at pH 4.8 [7]. Yuda and Okamoto [77] reported that citrus plants were insensitive to acidic soils. A study from our laboratory showed that pH 3.0 slightly decreased the growth of ‘Xuegan’ seedlings; pH 4.0 hardly decreased seedling growth; and seedling growth reached its maximum at pH 5.0 [78]. The high-pH setting of 4.8 was to prevent Cu precipitation. Twenty pots per treatment were assigned to a completely randomized design. Seventeen weeks after the pH–Cu treatments, approximately 0.5 cm long white root apexes were harvested at noon, immediately frozen in liquid N_2_, and subsequently stored in a −80 °C freezer until the extraction of metabolites and RNA. The nonsampled seedlings were subjected to Cu and CWM assays.

### 4.2. Assays of NCs, TFAAs, TSPs, CWM Extraction, CWCs, and Cu

Soluble sugars were extracted thrice with 80% (*v*/*v*) ethanol at 80 °C. Glucose, fructose, and sucrose in the supernatant and starch in the pellet were measured spectrophotometrically via an enzymatic method [22].

TFAAs were determined by the ninhydrin colorimetric method [5].

TSPs were assayed according to the methods of Bradford [79] after extraction with 50 mM phosphate buffer (pH 7.0).

Extraction of CWMs and assay of CWCs (pectin, HC1, HC2, lignin, and cellulose) were performed according to Zhang et al. [19].

Cu concentrations in roots and root CWs were assayed with a PinAAcle 900 F atomic absorption spectrometer (PerkinElmer Singapore Pte Ltd., Singapore).

### 4.3. Root RNA-Seq and qRT-PCR Validation

Equal amounts of frozen roots of four seedlings from four pots were pooled as one biological replicate. Twelve samples were subsequently sent to Wuhan MetWare Biotechnology Co., Ltd. (Wuhan, Hubei, China), for RNA extraction and RNA-Seq using the HiSeq Illumina platform (Illumina Inc., San Diego, CA, USA) as described previously [19]. High-quality clean reads, filtered by fastp v0.19.3, were mapped to the reference genome of *C. sinensi*s v1.0 (http://citrus.hzau.edu.cn/orange/download/index.php, accessed on 7 May 2022) by HISAT v2.1.0. Transcript assembly, functional annotation, and screening of DEGs were performed according to the previous methods [17]. The screening criteria for DEGs were |log_2_(fold change)| > 1 and a false discovery rate (FDR) < 0.05 using DESeq2 v1.22.1.

Thirty-two DEGs were randomly selected for qRT-PCR validation. Forward and reverse primers were designed using Primer PREMIER version 5.0 (Premier Biosoft International, CA, USA) (Table S23). qRT-PCR was performed in two technical replicates × three biological replicates using *U4/U6 small nuclear ribonucleoprotein PRP31* (*PRPF31*; Cs7g08440) and *actin* (Cs1g05000) as internal standards.

### 4.4. Widely Targeted Metabolome in Roots

Twelve samples were sent to Wuhan MetWare Biotechnology Co., Ltd. (Wuhan, Hubei, China) for widely targeted metabolome via the UPLC-ESI-MS/MS system (UPLC, SHIMADZU Nexera X2, www.shimadzu.com.cn/; MS, Applied Biosystems 4500 Q TRAP, www.appliedbiosystems.com.cn/, accessed on 7 May 2022). The screening criteria for DAMs were a |log_2_(fold change)| > 1 and a variable importance in projection (VIP) > 1 in the orthogonal projections to latent structures discriminant analysis (OPLS-DA). The functions of the DAMs were annotated using the KEGG compound database (http://www.kegg.jp/kegg/compound/, accessed on 2 June 2022) and MetWare metabolite database [20].

### 4.5. Integrated Analysis of Metabolome and Transcriptome

An integrated analysis was performed as described previously [20] after the two datasets were converted to log_2_ values. Pearson correlation coefficients (PCCs) > 0.80 and corresponding *p-*values (PCCPs) < 0.05 were used to screen the data for the integrated analysis.

### 4.6. Statistical Analysis

All data were subjected to two-way ANOVA (two (Cu levels) × two (pH)) and LSD test at *p* < 0.05 using a DPS of 7.05 (Hangzhou RuiFeng Information Technology Co. Ltd., Hangzhou, China). PCA, OPLS-DA, and HCA were performed using R (base package, version 3.5.0), R (MetaboAnalystR; version 1.0.1), and R (ComplexHeatmap; version 2.8.0), respectively, after the data were normalized [20].

## 5. Conclusions

**Figure 7:**
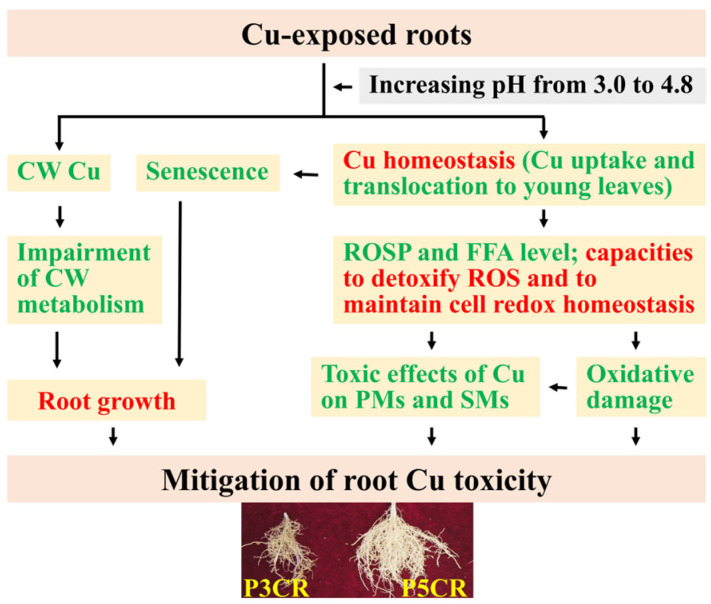
A model for the high-pH-mediated mitigation of Cu toxicity in *C. sinensis* roots. ROSP, ROS production; red, upregulation; green, downregulation.

Our results demonstrated that increasing the pH reduced the toxic effects of Cu on the abundances of PMs and SMs in roots. This difference was related to the following several factors: (*a*) increasing the pH improved the capacity of Cu-exposed seedlings (roots) to maintain Cu homeostasis by reducing Cu uptake and Cu translocation to young leaves; (*b*) increasing the pH decreased Cu toxicity-triggered oxidative damage by reducing ROS formation and the abundances of FFAs and enhancing the ability to detoxify ROS and maintain cell redox homeostasis; and (*c*) increasing the pH decreased the Cu toxicity-induced impairment of CW metabolism and senescence by decreasing the Cu levels in the roots and root CWs, thereby promoting root growth (Figure 7). Several genes (*HMA5*, *YSL1, ZIP1*, *protein disulfide-isomerase 5-4*, *NUDX2*, *FRD3*, and *BHLH29*), metabolites (SA, betaine, putrescine, coumarins, GSH, ASC, and lignin), and/or metabolic pathways (phenylpropanoid biosynthesis) might play a role in root Cu tolerance. Additionally, we observed some differences and similarities in Cu–pH interaction-responsive genes and metabolites between leaves and roots. To conclude, this study may provide novel information on the mechanisms underlying the elevated pH-mediated alleviation of Cu toxicity in roots and provide a theoretical basis for the development of new technologies to reduce Cu toxicity in plants.

## Data Availability

RNA-Seq data were deposited in NCBI database with SRA accession number PRJNA915596 (https://www.ncbi.nlm.nih.gov/search/all/?term=PRJNA915596, accessed on 1 October 2024). Data are archived in L-S Chen’s lab and available upon request.

## Supplementary Materials

The following supporting information can be downloaded at https://www.mdpi.com/article/10.3390/plants13213054/s1, Figure S1: qRT-PCR validation of 32 DEGs detected in P3R vs. P5R (A,E), P3CR vs. P3R (B,F), P3CR vs. P5CR (C,G), and P5CR vs. P5R (D,H) using *actin* (A–D) and *PRPF31* (E-H) as internal standards, and the correlation analysis of RNA-Seq data and qRT-PCR results using *actin* (I) and *PRPF31* (J) as internal standards; Figure S2: Pearson correlation coefficient matrix (A) and 2 D PCA plot (B) of P3R, P5R, P3CR, P3CR, and quality control sample (Mix), HCA of total metabolites identified in P3R, P5R, P3CR, and/or P3CR (C), and HCA of DEGs (D–G) for P3R vs. P5R, P3CR vs. P3R, P5CR vs. P5R, and P3CR vs. P5CR, respectively; Figure S3: Metabolite–gene Pearson correlation network between DAMs and DEGs involved in phenylpropanoid biosynthesis (ko00940) in P3CR vs. P3R (A) and P5CR vs. P5R (B); Figure S4: Metabolite–gene Pearson correlation network between DAMs and DEGs involved in glutathione metabolism (A–B) and cysteine and methionine metabolism (C–D) in P3CR vs. P3R (A,C) and P5CR vs. P5R (B,D); Figure S5: Venn analysis of total (A–D), upregulated (E–H), and downregulated (I–L) DEGs, as well as total (M–P), upregulated (Q–T), and downregulated (U–X) DAMs, identified in P3R vs. P5R and P3L vs. P5L (A, E, I, M, Q and U), P3CR vs. P3R and P3CL vs. P3L (B, F, J, N, R and V), P5CR vs. P5R and P5CL vs. P5L (C, G, K, O, S and W), and P3CR vs. P5CR and P3CL vs. P5CL (D, H, K, P, T and X); Figure S6: Venn analysis of enriched KEGG pathways for DEGs (A–D) and DAMs (E–H) as well as enriched GO terms in BP (I–L), CC (M–P), and MF (Q–T) for DEGs identified in P3R vs. P5R and P3L vs. P5L (A, E, I, M and Q), P3CR vs. P3R and P3CL vs. P3L (B, F, J, N and R), P5CR vs. P5R and P5CL vs. P5L (C, G, K, O and S), and P3CR vs. P5CR and P3CL vs. P5CL (D, H, K, P and T); Table S1: Summary of the RNA-Seq data collected from control and Cu-toxic roots of *C. sinensis* seedlings; Table S2: List of known and novel genes identified in P3R, P5R, P3CR, and/or P5CR; Table S3: List of the enriched KEGG for DEGs identified in P3CR vs. P3R; Table S4: List of the enriched KEGG for DEGs identified in P5CR vs. P5R; Table S5: List of the enriched GO terms for DEGs identified in P3CR vs. P3R; Table S6: List of the enriched GO terms for DEGs identified in P5CR vs. P5R; Table S7: List of all metabolites identified in P3R, P5R, P3CR, and/or P5CR; Table S8: List of DAMs detected in P3R vs. P5R, P3CR vs. P3R, P5CR vs. P5R, and/or P3CR vs. P5CR; Table S9: List of the enriched KEGG for DAMs identified in P3CR vs. P3R; Table S10: List of the enriched KEGG for DAMs identified in P5CR vs. P5R; Table S11: DEGs related to Cu homeostasis in P3CR vs. P3R and/or P5CR vs. P5R; Table S12: DEGs related to cell wall metabolism in P3CR vs. P3R and/or P5CR vs. P5R; Table S13: DEGs related to plant organ senescence in P3CR vs. P3R and/or P5CR vs. P5R; Table S14: DEGs and DAMs related to carbohydrate and energy metabolisms in P3CR vs. P3R and/or P5CR vs. P5R; Table S15: DEGs and DAMs related to N, protein, and AA metabolisms in P3CR vs. P3R and/or P5CR vs. P5R; Table S16: Classification of DAMs based on whether they contain N, P, or S; Table S17: DEGs related to lipid metabolism in P3CR vs. P3R and/or P5CR vs. P5R; Table S18: DEGs related to nucleotide metabolism in P3CR vs. P3R and/or P5CR vs. P5R; Table S19: DEGs and DAMs related to biosynthesis and catabolism of SMs in P3CR vs. P3R and/or P5CR vs. P5R; Table S20: Differentially abundant phenylpropanoids detected in P3CR vs. P3R and/or P5CR vs. P5R; Table S21: Differentially abundant phenolic compounds detected in P3CR vs. P3R and/or P5CR vs. P5R; Table S22: DEGs and DAMs related to ROS detoxification and cell redox homeostasis in P3CR vs. P3R and/or P5CR vs. P5R; Table S23: Specific primer pairs used for qRT-PCR expression analysis.
