# Supplementary material for: Mechanisms by Which Increased pH Ameliorates Copper Excess in Citrus sinensis Roots: Insight from a Combined Analysis of Physiology, Transcriptome, and Metabolome
Source: Plants (Basel). 2024 Oct 31;13(21):3054. doi: 10.3390/plants13213054 (PMC11548300; doi:10.3390/plants13213054)
Supplement: Supplementary file 1 [file plants-13-03054-s001.zip › Fig. S1SecondRevision.pdf]

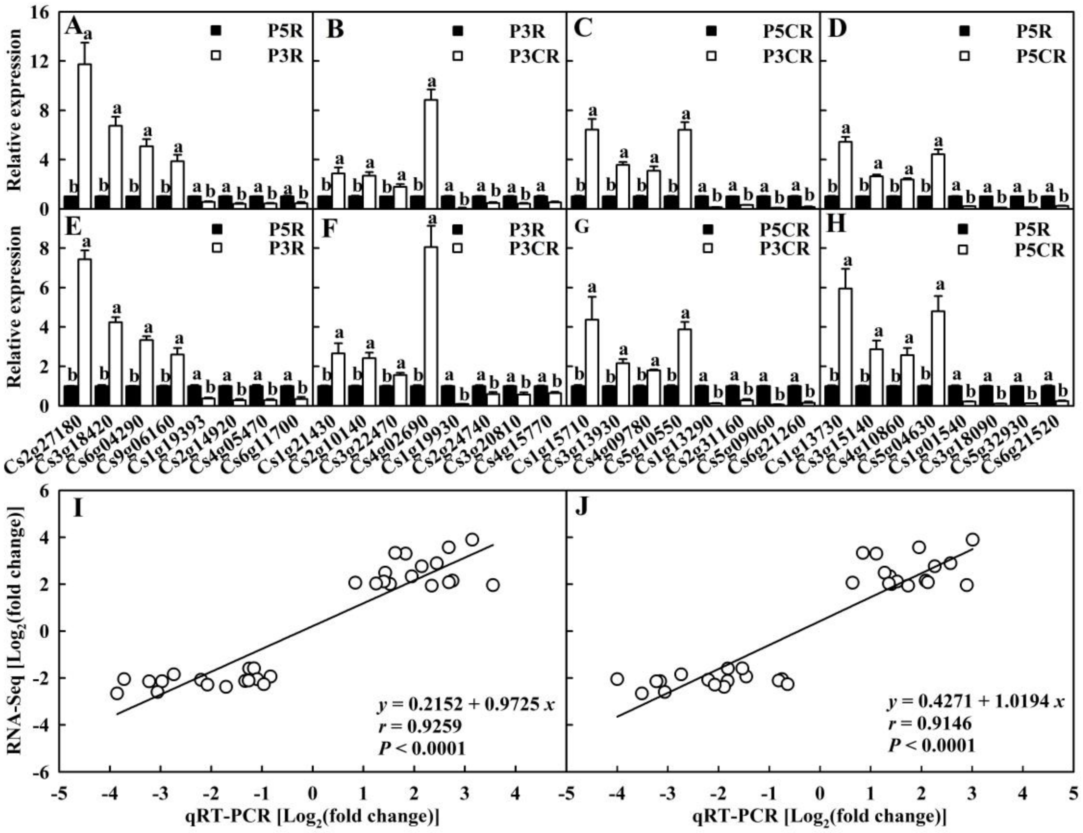

Figure S1. qRT-PCR validation of 32 DEGs detected in P3R vs. P5R (A,E), P3CR vs. P3R (B,F), P3CR vs. P5CR (C,G), and P5CR vs. P5R (D,H) using *actin* (A–D) and *PRPF31* (E-H) as internal standards, and the correlation analysis of RNA-Seq data and qRT-PCR results using *actin* (I) and *PRPF31* (J) as internal standards. Bars represent means ± SE of two technical and three biological replicates. For the same genes, different letters above the bars indicate a significant difference at $p < 0.05$.
