# Supplementary material for: Mechanisms by Which Increased pH Ameliorates Copper Excess in Citrus sinensis Roots: Insight from a Combined Analysis of Physiology, Transcriptome, and Metabolome
Source: Plants (Basel). 2024 Oct 31;13(21):3054. doi: 10.3390/plants13213054 (PMC11548300; doi:10.3390/plants13213054)
Supplement: Supplementary file 1 [file plants-13-03054-s001.zip › Fig. S2SecondRevision.pdf]

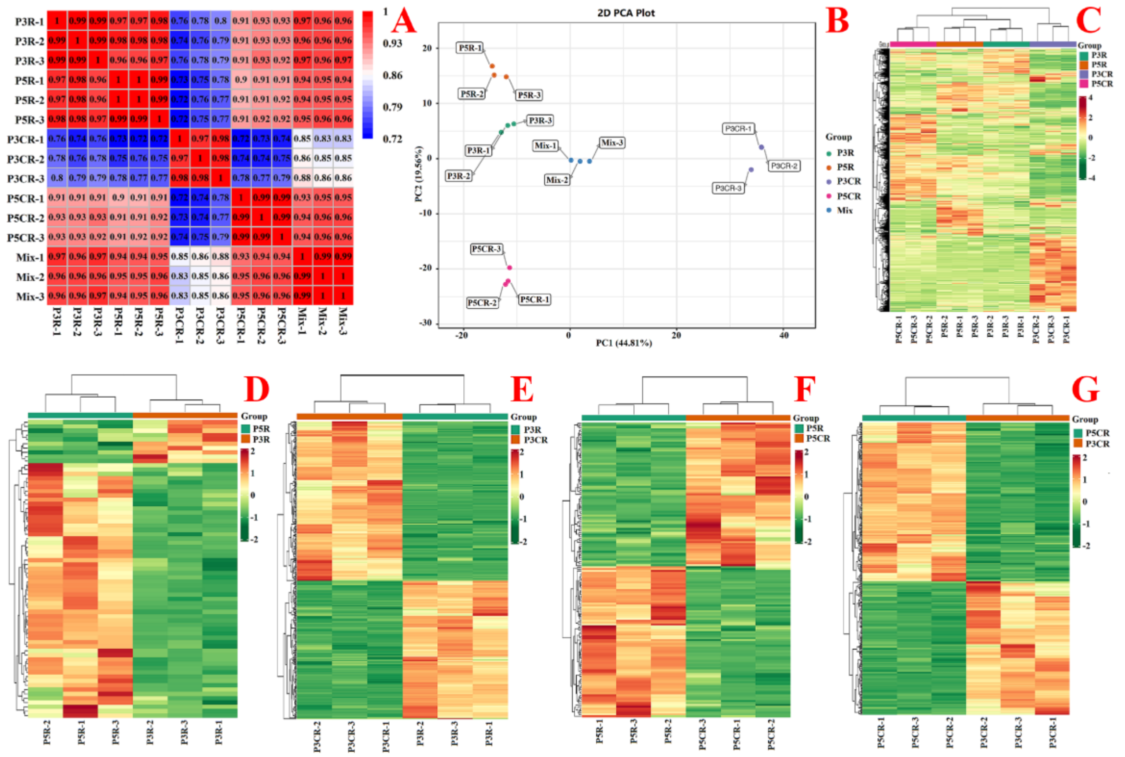

Figure S2. Pearson correlation coefficient matrix (A) and 2 D PCA plot (B) of P3R, P5R, P3CR, P3CR, and quality control sample (Mix), HCA of total metabolites identified in P3R, P5R, P3CR, and/or P3CR (C), and HCA of DEGs (D–G) for P3R vs. P5R, P3CR vs. P3R, P5CR vs. P5R, and P3CR vs. P5CR, respectively
