# Supplementary material for: Mechanisms by Which Increased pH Ameliorates Copper Excess in Citrus sinensis Roots: Insight from a Combined Analysis of Physiology, Transcriptome, and Metabolome
Source: Plants (Basel). 2024 Oct 31;13(21):3054. doi: 10.3390/plants13213054 (PMC11548300; doi:10.3390/plants13213054)
Supplement: Supplementary file 1 [file plants-13-03054-s001.zip › Fig. S3SecondRevision.pdf]

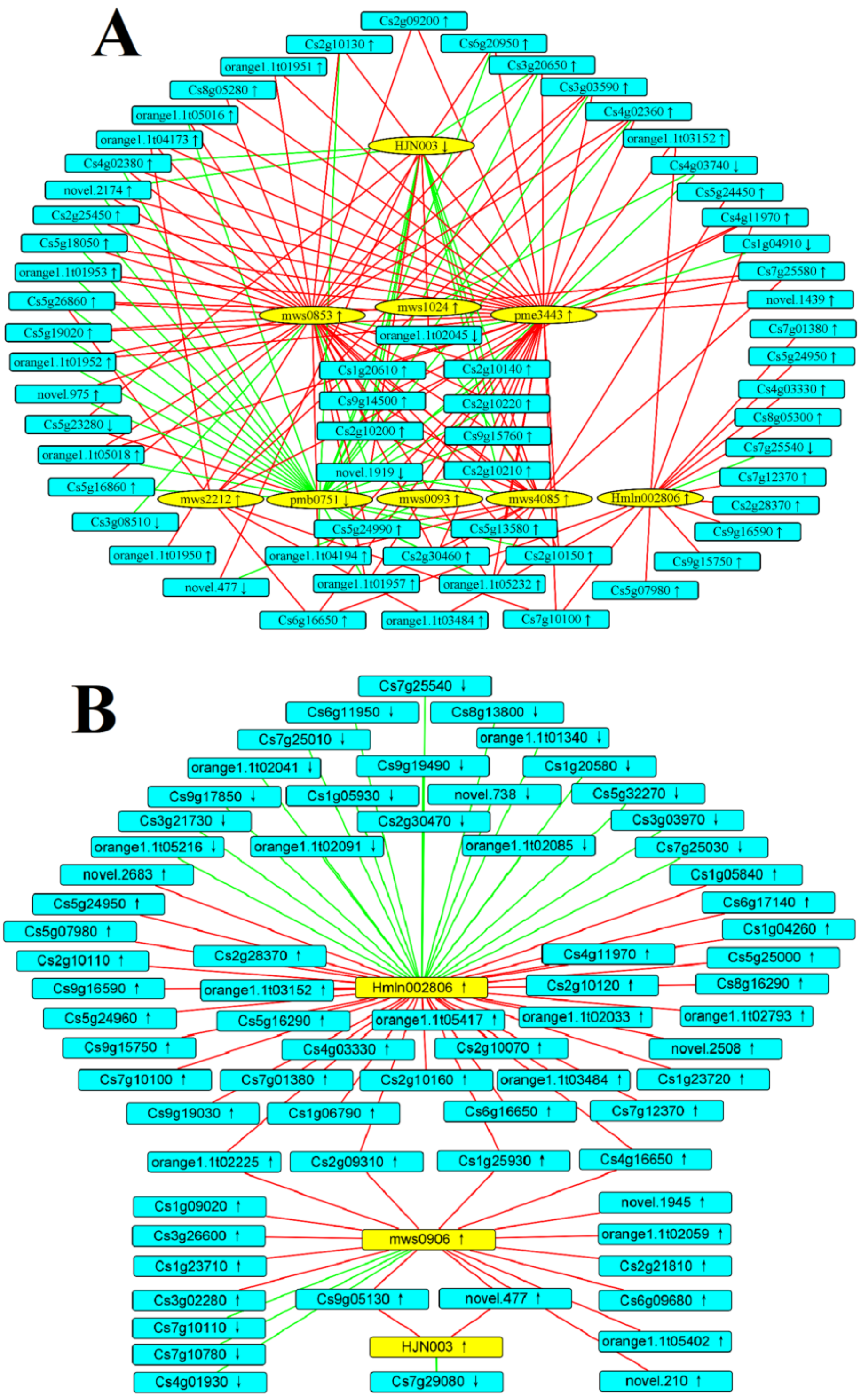

Figure S3. Metabolite–gene Pearson correlation network between DAMs and DEGs involved in phenylpropanoid biosynthesis (ko00940) in P3CR vs. P3R (A) and P5CR vs. P5R (B). Metabolite-gene pairs were connected by edges; red and green lines represent positive and negative correlation, respectively; yellow and blue nodes represent DAMs and DEGs, respectively. ↓, downregulation; ↑, upregulation.
