# Supplementary material for: Mechanisms by Which Increased pH Ameliorates Copper Excess in Citrus sinensis Roots: Insight from a Combined Analysis of Physiology, Transcriptome, and Metabolome
Source: Plants (Basel). 2024 Oct 31;13(21):3054. doi: 10.3390/plants13213054 (PMC11548300; doi:10.3390/plants13213054)
Supplement: Supplementary file 1 [file plants-13-03054-s001.zip › Fig. S4SecondRevision.pdf]

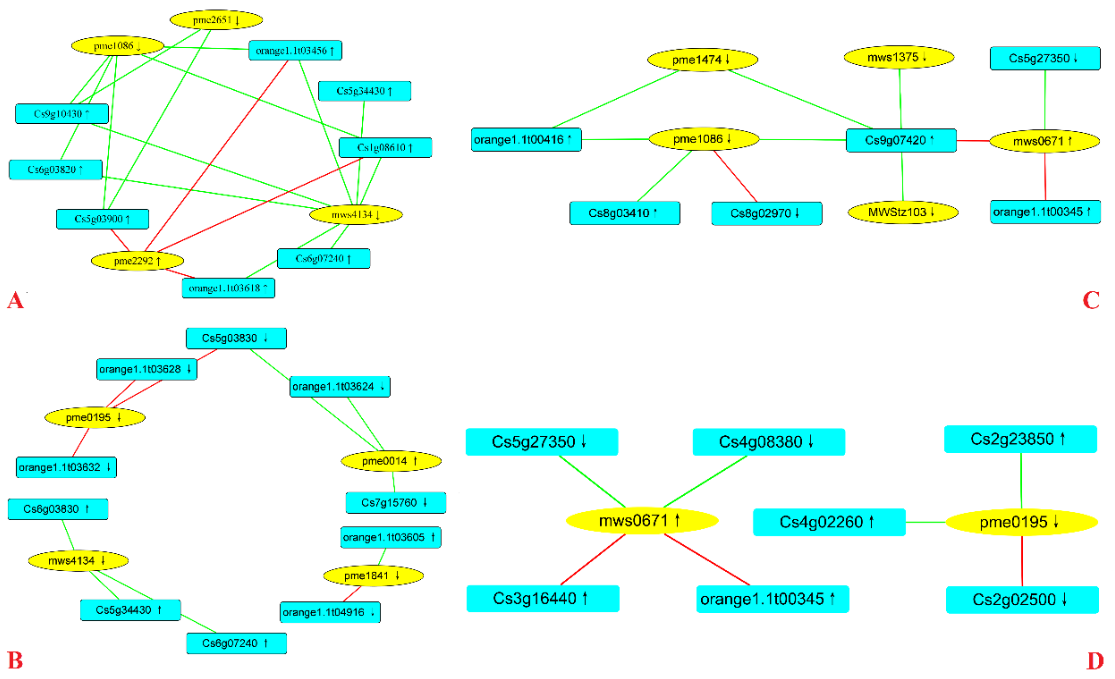

Figure S4. Metabolite–gene Pearson correlation network between DAMs and DEGs involved in glutathione metabolism (A–B) and cysteine and methionine metabolism (C–D) in P3CR vs. P3R (A,C) and P5CR vs. P5R (B,D). Metabolite-gene pairs were connected by edges; red and green lines represent positive and negative correlation, respectively; yellow and blue nodes represent DAMs and DEGs, respectively. ↓, downregulation; ↑, upregulation.
