# Supplementary material for: Mechanisms by Which Increased pH Ameliorates Copper Excess in Citrus sinensis Roots: Insight from a Combined Analysis of Physiology, Transcriptome, and Metabolome
Source: Plants (Basel). 2024 Oct 31;13(21):3054. doi: 10.3390/plants13213054 (PMC11548300; doi:10.3390/plants13213054)
Supplement: Supplementary file 1 [file plants-13-03054-s001.zip › Fig. S5SecondRevision.pdf]

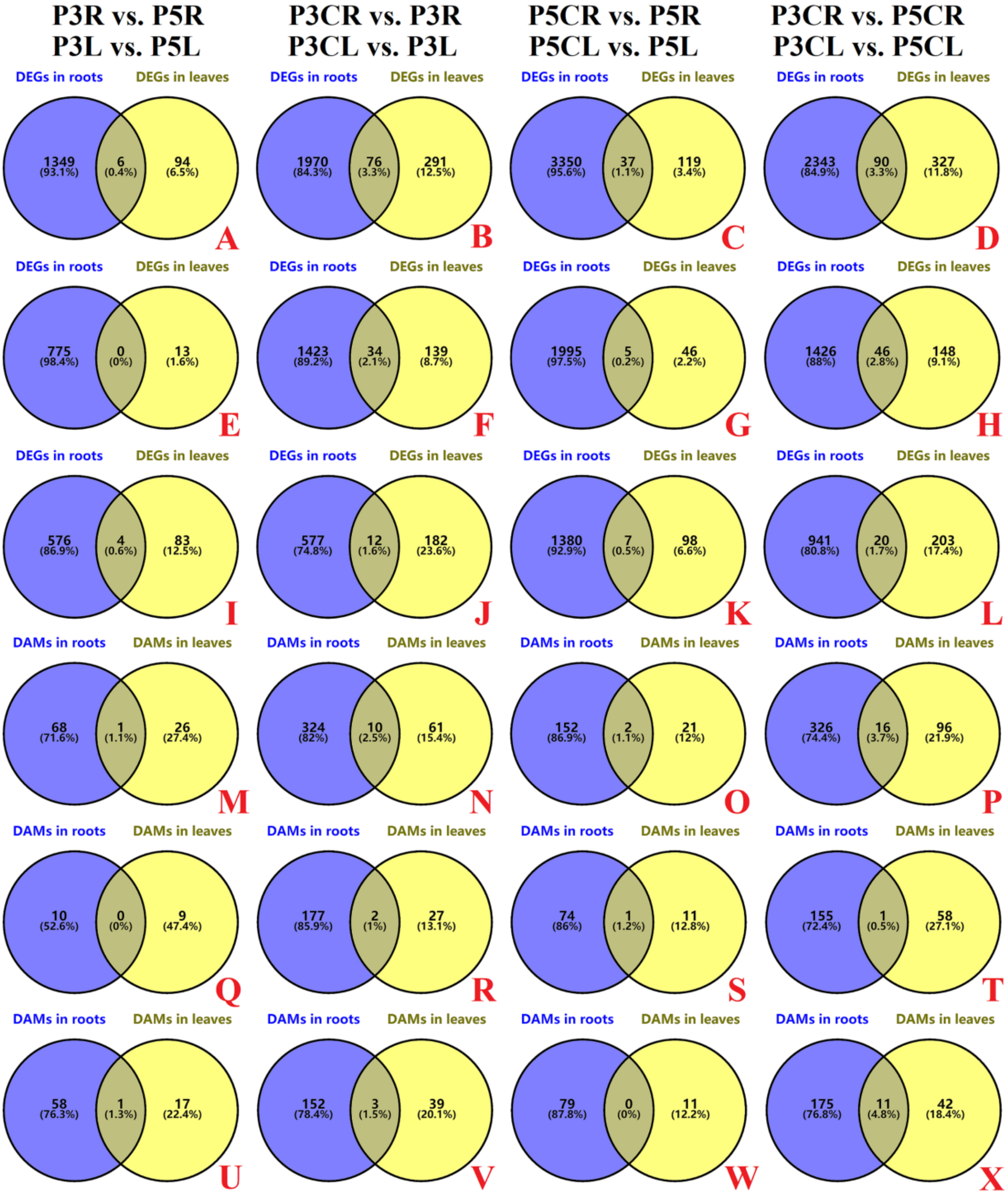

Figure S5. Venn analysis of total (A–D), upregulated (E–H), and downregulated (I–L) DEGs, as well as total (M–P), upregulated (Q–T), and downregulated (U–X) DAMs, identified in P3R vs. P5R and P3L vs. P5L (A, E, I, M, Q and U), P3CR vs. P3R and P3CL vs. P3L (B, F, J, N, R and V), P5CR vs. P5R and P5CL vs. P5L (C, G, K, O, S and W), and P3CR vs. P5CR and P3CL vs. P5CL (D, H, K, P, T and X).
