# Supplementary material for: Mechanisms by Which Increased pH Ameliorates Copper Excess in Citrus sinensis Roots: Insight from a Combined Analysis of Physiology, Transcriptome, and Metabolome
Source: Plants (Basel). 2024 Oct 31;13(21):3054. doi: 10.3390/plants13213054 (PMC11548300; doi:10.3390/plants13213054)
Supplement: Supplementary file 1 [file plants-13-03054-s001.zip › Fig. S6SecondRevision.pdf]

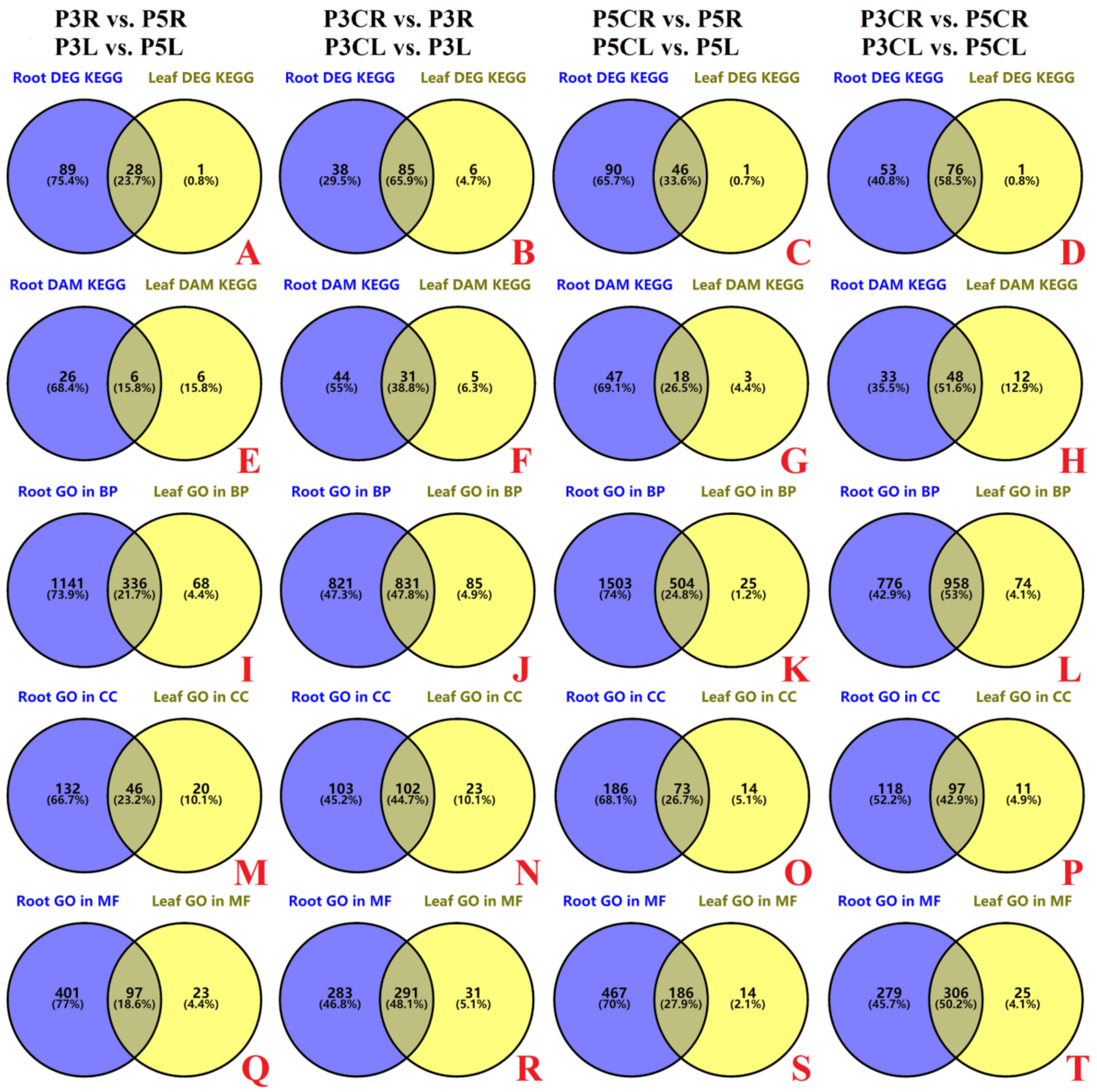

Figure S6. Venn analysis of enriched KEGG pathways for DEGs (A–D) and DAMs (E–H) as well as enriched GO terms in BP (I–L), CC (M–P), and MF (Q–T) for DEGs identified in P3R vs. P5R and P3L vs. P5L (A, E, I, M and Q), P3CR vs. P3R and P3CL vs. P3L (B, F, J, N and R), P5CR vs. P5R and P5CL vs. P5L (C, G, K, O and S), and P3CR vs. P5CR and P3CL vs. P5CL (D, H, K, P and T).
