# Supplementary material for: Mechanisms by Which Increased pH Ameliorates Copper Excess in Citrus sinensis Roots: Insight from a Combined Analysis of Physiology, Transcriptome, and Metabolome
Source: Plants (Basel). 2024 Oct 31;13(21):3054. doi: 10.3390/plants13213054 (PMC11548300; doi:10.3390/plants13213054)
Supplement: Supplementary file 1 [file plants-13-03054-s001.zip › Table S11SecondRevision.pdf]

Table S11. DEGs related to Cu homeostasis in P3CR vs. P3R and/or P5CR vs. P5R

**Accession No.**	**KEGG**	**Swissprot (KOG)**	**Log$_2$(FC)**	
			P3CR vs. P3R	P5CR vs. P5R
Copper ion binding [GO:0005507; P = 0.0002 (P3CR vs. P3R) and 0.0007 (P5CR vs. P5R)				
Cs1g01800	K00423 L-ascorbate oxidase [EC:1.10.3.3] | (RefSeq) L-ascorbate oxidase-like (A)	L-ascorbate oxidase; ASO; Ascorbase; EC=1.10.3.3; Flags: Precursor (At4g39830)		1.402
Cs1g14690	K05909 laccase [EC:1.10.3.2] | (RefSeq) LOC109742875; laccase-19-like isoform X1 (A)	Multicopper oxidase LPR1; EC=1.-.-.-; Protein LOW PHOSPHATE ROOT 1; Flags: Precursor (At1g23010)	1.012	2.441
Cs1g21320	K06123 1-acylglycerone phosphate reductase [EC:1.1.1.101] | (RefSeq) hypothetical protein (A)	Short-chain dehydrogenase reductase 4; AtSDR4; EC=1.1.1.- (At5g10050)	1.601	
Cs1g23100	K19791 iron transport multicopper oxidase | (RefSeq) L-ascorbate oxidase homolog (A)	L-ascorbate oxidase homolog; EC=1.10.3.-; Flags: Precursor (At4g37160)		1.974
Cs1g24250	K05909 laccase [EC:1.10.3.2] | (RefSeq) laccase-11-like (A)	Laccase-11; EC=1.10.3.2; Benzenediol:oxygen oxidoreductase 11; Diphenol oxidase 11; Urishiol oxidase 11; Flags: Precursor (At5g03260)	3.444	6.447
Cs2g23190	K04077 chaperonin GroEL | (RefSeq) chaperonin CPN60-2, mitochondrial-like (A)	Chaperonin CPN60, mitochondrial; HSP60; Flags: Precursor (At3g23990)		1.064
Cs2g29390	K04078 chaperonin GroES | (RefSeq) hypothetical protein (A)	10 kDa chaperonin, mitochondrial; Chaperonin 10; CPN10; Protein groES; Flags: Precursor (At1g14980)	-1.412	-1.526
Cs5g03780	K17686 Cu+-exporting ATPase [EC:3.6.3.54] | (RefSeq) probable copper-transporting ATPase HMA5 (A)	Probable copper-transporting ATPase HMA5; EC=3.6.3.54; Probable copper-transporting ATPase 3; Protein HEAVY METAL ATPASE 5 (At1g63440)		1.463
Cs5g03790	K17686 Cu+-exporting ATPase [EC:3.6.3.54] | (RefSeq) probable copper-transporting ATPase HMA5 (A)	Probable copper-transporting ATPase HMA5; EC=3.6.3.54; Probable copper-transporting ATPase 3; Protein HEAVY METAL ATPASE 5 (At1g63440)		1.060
Cs5g03800	K17686 Cu+-exporting ATPase [EC:3.6.3.54] | (RefSeq) probable copper-transporting ATPase HMA5 (A)	Probable copper-transporting ATPase HMA5; EC=3.6.3.54; Probable copper-transporting ATPase 3; Protein HEAVY METAL ATPASE 5 (At1g63440)		1.130
Cs5g09470	K00276 primary-amine oxidase [EC:1.4.3.21] | (RefSeq) primary amine oxidase-like (A)	Primary amine oxidase; EC=1.4.3.21 {ECO:0000250|UniProtKB:P46883}; Amine oxidase [copper-containing]; Flags: Precursor (At4g12290)		1.008
Cs5g19640	K08738 cytochrome c | (RefSeq) hypothetical protein (A)	Cytochrome c-2 {ECO:0000305}; Cytochrome c At4g10040 (At4g10040)		-1.113

Cs6g06880	K05909 laccase [EC:1.10.3.2] | (RefSeq) hypothetical protein (A)	Laccase-17; EC=1.10.3.2; Benzenediol:oxygen oxidoreductase 17; Diphenol oxidase 17; Urishiol oxidase 17; Flags: Precursor (At5g60020)		4.438
Cs6g06920	K05909 laccase [EC:1.10.3.2] | (RefSeq) laccase-17-like (A)	Laccase-17; EC=1.10.3.2; Benzenediol:oxygen oxidoreductase 17; Diphenol oxidase 17; Urishiol oxidase 17; Flags: Precursor (At5g60020)	1.652	3.497
Cs6g07400	K05909 laccase [EC:1.10.3.2] | (RefSeq) laccase-7-like (A)	Laccase-7; EC=1.10.3.2; Benzenediol:oxygen oxidoreductase 7; Diphenol oxidase 7; Urishiol oxidase 7; Flags: Precursor (At3g09220)	1.881	
Cs6g07410	K05909 laccase [EC:1.10.3.2] | (RefSeq) hypothetical protein (A)	Laccase-7; EC=1.10.3.2; Benzenediol:oxygen oxidoreductase 7; Diphenol oxidase 7; Urishiol oxidase 7; Flags: Precursor (At3g09220)	1.498	2.464
Cs6g07450	K05909 laccase [EC:1.10.3.2] | (RefSeq) laccase-7-like (A)	Laccase-7; EC=1.10.3.2; Benzenediol:oxygen oxidoreductase 7; Diphenol oxidase 7; Urishiol oxidase 7; Flags: Precursor (At3g09220)		2.442
Cs6g07800	K05909 laccase [EC:1.10.3.2] | (RefSeq) hypothetical protein (A)	Laccase-4; EC=1.10.3.2; Benzenediol:oxygen oxidoreductase 4; Diphenol oxidase 4; Protein IRREGULAR XYLEM 12; Urishiol oxidase 4; Flags: Precursor (At2g38080)	1.504	3.815
Cs6g09690	K14455 aspartate aminotransferase, mitochondrial [EC:2.6.1.1] | (RefSeq) hypothetical protein (A)	Aspartate aminotransferase, mitochondrial; EC=2.6.1.1; Transaminase A; Flags: Precursor (At2g30970)	-1.627	
Cs6g11860	K05909 laccase [EC:1.10.3.2] | (RefSeq) hypothetical protein (A)	Laccase-11; EC=1.10.3.2; Benzenediol:oxygen oxidoreductase 11; Diphenol oxidase 11; Urishiol oxidase 11; Flags: Precursor (At5g03260)	2.724	5.299
Cs7g11700	K09564 peptidyl-prolyl isomerase E (cyclophilin E) [EC:5.2.1.8] | (RefSeq) peptidyl-prolyl cis-trans isomerase E (A)	Glycine-rich RNA-binding protein 3, mitochondrial; AtGR-RBP3; AtRBG3; Mitochondrial RNA-binding protein 2a; At-mRBP2a; Organelle RRM domain-containing protein 3 {ECO:0000303|PubMed:25800738}; Flags: Precursor (At1g13690)		-1.204
Cs7g18550	K00799 glutathione S-transferase [EC:2.5.1.18] | (RefSeq) glutathione S-transferase F9-like (A)	Glutathione S-transferase F9; AtGSTF9; EC=2.5.1.18; AtGSTF7; GST class-phi member 9 (At2g30860)	1.427	3.342
Cs7g19160	K00261 glutamate dehydrogenase (NAD(P)+) [EC:1.4.1.3] | (RefSeq) hypothetical protein (A)	Glutamate dehydrogenase 2; GDH 2; EC=1.4.1.3 (At5g07440)		-1.412
Cs7g21880	K19791 iron transport multicopper oxidase | (RefSeq) monocopper oxidase-like protein SKU5 (A)	Monocopper oxidase-like protein SKU5; Skewed roots; Flags: Precursor (At4g12420)	1.963	-7.098
Cs7g21890	K19791 iron transport multicopper oxidase | (RefSeq) monocopper oxidase-like protein SKU5 (A)	Monocopper oxidase-like protein SKU5; Skewed roots; Flags: Precursor (At5g48450)	2.325	-3.812
Cs7g23490	K05909 laccase [EC:1.10.3.2] | (RefSeq) hypothetical protein (A)	Laccase-17; EC=1.10.3.2; Benzenediol:oxygen oxidoreductase 17; Diphenol oxidase 17; Urishiol oxidase 17; Flags: Precursor (At5g60020)		3.109
Cs7g30410	K05909 laccase [EC:1.10.3.2] | (RefSeq) hypothetical protein (A)	Laccase-5; EC=1.10.3.2; Benzenediol:oxygen oxidoreductase 5; Diphenol oxidase 5; Urishiol oxidase 5; Flags: Precursor (At2g40370)	1.431	4.293

Cs7g31620	K05909 laccase [EC:1.10.3.2] | (RefSeq) hypothetical protein (A)	Laccase-22; EC=1.10.3.2; Benzenediol:oxygen oxidoreductase 22; Diphenol oxidase 22; Urishiol oxidase 22; Flags: Precursor (At2g38080)	1.349	2.990
Cs8g05800	K00069 15-hydroxyprostaglandin dehydrogenase (NAD) [EC:1.1.1.141] | (RefSeq) 15-hydroxyprostaglandin dehydrogenase [NAD(+)]-like (A)	3-oxoacyl-[acyl-carrier-protein] reductase, chloroplastic; EC=1.1.1.100; 3-ketoacyl-acyl carrier protein reductase; Flags: Precursor (At1g49670_1)	-1.452	-1.487
Cs8g11710	K05909 laccase [EC:1.10.3.2] | (RefSeq) laccase-13-like (A)	Laccase-3; EC=1.10.3.2; Benzenediol:oxygen oxidoreductase 3; Diphenol oxidase 3; Urishiol oxidase 3; Flags: Precursor (At2g30210)	2.670	3.781
Cs8g17630	K05909 laccase [EC:1.10.3.2] | (RefSeq) hypothetical protein (A)	Laccase-17; EC=1.10.3.2; Benzenediol:oxygen oxidoreductase 17; Diphenol oxidase 17; Urishiol oxidase 17; Flags: Precursor (At5g60020)	2.374	5.969
Cs8g18800	K05909 laccase [EC:1.10.3.2] | (RefSeq) laccase-17 (A)	Laccase-17; EC=1.10.3.2; Benzenediol:oxygen oxidoreductase 17; Diphenol oxidase 17; Urishiol oxidase 17; Flags: Precursor (At5g60020)	3.255	3.790
Cs8g19850	K05909 laccase [EC:1.10.3.2] | (RefSeq) hypothetical protein (A)	Laccase-4; EC=1.10.3.2; Benzenediol:oxygen oxidoreductase 4; Diphenol oxidase 4; Protein IRREGULAR XYLEM 12; Urishiol oxidase 4; Flags: Precursor (At2g38080)	1.651	3.446
Cs9g06700	K00276 primary-amine oxidase [EC:1.4.3.21] | (RefSeq) hypothetical protein (A)	Primary amine oxidase 2; EC=1.4.3.21 {ECO:0000250|UniProtKB:P12807}; Amine oxidase [copper-containing] 2; Flags: Precursor (At1g31670)	7.325	
Cs9g06710	K00276 primary-amine oxidase [EC:1.4.3.21] | (RefSeq) hypothetical protein (A)	Primary amine oxidase; EC=1.4.3.21 {ECO:0000250|UniProtKB:P46883}; Amine oxidase [copper-containing]; Flags: Precursor; Fragment (At1g31690)	5.304	
Cs9g06770	K00276 primary-amine oxidase [EC:1.4.3.21] | (RefSeq) hypothetical protein (A)	Primary amine oxidase; EC=1.4.3.21 {ECO:0000250|UniProtKB:P46883}; Amine oxidase [copper-containing]; Flags: Precursor; Fragment (At1g31700)	1.992	
orange1.1t00518	K05909 laccase [EC:1.10.3.2] | (RefSeq) hypothetical protein (A)	Laccase-6; EC=1.10.3.2; Benzenediol:oxygen oxidoreductase 6; Diphenol oxidase 6; Urishiol oxidase 6; Flags: Precursor (At2g46570)		1.676
orange1.1t04018		Heavy metal-associated isoprenylated plant protein 5 {ECO:0000303|PubMed:21072340, ECO:0000303|PubMed:23368984}; AtHIP05 {ECO:0000303|PubMed:21072340, ECO:0000303|PubMed:23368984}; Farnesylated protein 2 {ECO:0000303|PubMed:8837031}; AtFP2 {ECO:0000303|PubMed:8837031}; Flags: Precursor (At2g36950)	1.469	
orange1.1t04786	K05909 laccase [EC:1.10.3.2] | (RefSeq) laccase-19-like (A)	Multicopper oxidase LPR1; EC=1.-.-.-; Protein LOW PHOSPHATE ROOT 1; Flags: Precursor (At1g23010)		1.064

Copper chaperone				
Cs2g08850	K07213 copper chaperone | (RefSeq) copper transport protein ATX1-like (A)	Copper transport protein ATX1; Copper chaperone ATX1 (At3g56240)	-1.193	
Cs4g16810	K07213 copper chaperone | (RefSeq) protein SODIUM POTASSIUM ROOT DEFECTIVE 1-like (A)	Protein SODIUM POTASSIUM ROOT DEFECTIVE 1 {ECO:0000303|PubMed:21193571}; NaKR1 {ECO:0000303|PubMed:21193571}; Heavy metal-associated plant protein 2 {ECO:0000303|PubMed:23368984}; AtHPP02 {ECO:0000303|PubMed:23368984}; Nuclear-enriched phloem companion cell gene 6 {ECO:0000303|PubMed:18354040}; NPCC6 {ECO:0000303|PubMed:18354040} (At3g24450)		1.623
Cs5g16220	K07213 copper chaperone | (RefSeq) copper transport protein ATX1-like (A)	Heavy metal-associated isoprenylated plant protein 9 {ECO:0000303|PubMed:21072340, ECO:0000303|PubMed:23368984}; AtHIP09 {ECO:0000303|PubMed:21072340, ECO:0000303|PubMed:23368984}; Flags: Precursor (At5g24580)	-1.273	-1.565
Cs5g16230	K07213 copper chaperone | (RefSeq) hypothetical protein (A)	Protein SODIUM POTASSIUM ROOT DEFECTIVE 2 {ECO:0000303|PubMed:21193571}; NaKR2 {ECO:0000303|PubMed:21193571}; Heavy metal-associated plant protein 3 {ECO:0000303|PubMed:23368984}; AtHPP03 {ECO:0000303|PubMed:23368984} (At4g27590)		-4.283
Copper ion transport [GO:0006825; P = 0.3782 (P3CR vs. P3R) and 0.1609 (P5CR vs. P5R)]				
Cs2g08850	K07213 copper chaperone | (RefSeq) copper transport protein ATX1-like (A)	Copper transport protein ATX1; Copper chaperone ATX1 (At3g56240)	-1.193	
Cs5g03780	K17686 Cu+-exporting ATPase [EC:3.6.3.54] | (RefSeq) probable copper-transporting ATPase HMA5 (A)	Probable copper-transporting ATPase HMA5; EC=3.6.3.54; Probable copper-transporting ATPase 3; Protein HEAVY METAL ATPASE 5 (At1g63440)		1.463
Cs5g03790	K17686 Cu+-exporting ATPase [EC:3.6.3.54] | (RefSeq) probable copper-transporting ATPase HMA5 (A)	Probable copper-transporting ATPase HMA5; EC=3.6.3.54; Probable copper-transporting ATPase 3; Protein HEAVY METAL ATPASE 5 (At1g63440)		1.060
Cs5g03800	K17686 Cu+-exporting ATPase [EC:3.6.3.54] | (RefSeq) probable copper-transporting ATPase HMA5 (A)	Probable copper-transporting ATPase HMA5; EC=3.6.3.54; Probable copper-transporting ATPase 3; Protein HEAVY METAL ATPASE 5 (At1g63440)		1.130
Cs8g19460	K14686 solute carrier family 31 (copper transporter), member 1 | (RefSeq) copper transporter 1-like (A)	Copper transporter 1; AtCOPT1 (At5g59030)	2.228	1.607

Others related to Cu homeostasis				
Cs5g01560	K23490 cytochrome b5 | (RefSeq) metal-nicotianamine transporter YSL3-like (A)	Metal-nicotianamine transporter YSL1; Protein YELLOW STRIPE LIKE 1; AtYSL1 (At5g53550)		3.894
orange1.1t03274	K14709 solute carrier family 39 (zinc transporter), member 1/2/3 | (RefSeq) hypothetical protein (A)	Zinc transporter 1; ZRT/IRT-like protein 1; Flags: Precursor (At3g12750)		1.292
orange1.1t03275	K14709 solute carrier family 39 (zinc transporter), member 1/2/3 | (RefSeq) hypothetical protein (A)	Zinc transporter 1; ZRT/IRT-like protein 1; Flags: Precursor (At3g12750)		1.311
