# Supplementary material for: Mechanisms by Which Increased pH Ameliorates Copper Excess in Citrus sinensis Roots: Insight from a Combined Analysis of Physiology, Transcriptome, and Metabolome
Source: Plants (Basel). 2024 Oct 31;13(21):3054. doi: 10.3390/plants13213054 (PMC11548300; doi:10.3390/plants13213054)
Supplement: Supplementary file 1 [file plants-13-03054-s001.zip › Table S12SecondRevision.pdf]

Table S12. DEGs related to cell wall metabolism in P3CR vs. P3R and/or P5CR vs. P5R

Accession No.	KEGG	Swissprot (KOG)	Log2(FC)	
			P3CR vs. P3R	P5CR vs. P5R
Cell wall part [GO:0044426; P = 0.0922 (P3CR vs. P3R) and 0.0012 (P5CR vs. P5R)]				
Cs2g10950	K13420 LRR receptor-like serine/threonine-protein kinase FLS2 [EC:2.7.11.1] | (RefSeq) hypothetical protein (A)	LRR receptor-like serine/threonine-protein kinase GSO1 {ECO:0000303|PubMed:18088309}; EC=2.7.11.1 {ECO:0000255|PROSITE-ProRule:PRU00159}; Protein GASSHO 1 {ECO:0000303|PubMed:18088309}; Protein SCHENGEN 3 {ECO:0000303|PubMed:25233277}; Flags: Precursor (At4g20140)		1.586
Cs2g11910	K13420 LRR receptor-like serine/threonine-protein kinase FLS2 [EC:2.7.11.1] | (RefSeq) LRR receptor-like serine/threonine-protein kinase FLS2 (A)	LRR receptor-like serine/threonine-protein kinase GSO1 {ECO:0000303|PubMed:18088309}; EC=2.7.11.1 {ECO:0000255|PROSITE-ProRule:PRU00159}; Protein GASSHO 1 {ECO:0000303|PubMed:18088309}; Protein SCHENGEN 3 {ECO:0000303|PubMed:25233277}; Flags: Precursor (At4g20140)	1.313	1.890
Cs4g13170	K09422 transcription factor MYB, plant | (RefSeq) transcription factor RAX2-like (A)	Transcription factor MYB36 {ECO:0000303|PubMed:9839469}; Myb-related protein 36 {ECO:0000303|PubMed:9839469}; AtMYB36 {ECO:0000303|PubMed:9839469} (At5g65790)	2.853	
Cs6g10250	K04733 interleukin-1 receptor-associated kinase 4 [EC:2.7.11.1] | (RefSeq) hypothetical protein (A)	Receptor-like protein kinase FERONIA; EC=2.7.11.1; Protein SIRENE; Flags: Precursor (At3g51550)		1.116
Cs6g16450	K04733 interleukin-1 receptor-associated kinase 4 [EC:2.7.11.1] | (RefSeq) hypothetical protein (A)	Receptor-like protein kinase FERONIA; EC=2.7.11.1; Protein SIRENE; Flags: Precursor (At3g51550)		1.144
Cs6g19610	K04733 interleukin-1 receptor-associated kinase 4 [EC:2.7.11.1] | (RefSeq) hypothetical protein (A)	Receptor-like protein kinase FERONIA; EC=2.7.11.1; Protein SIRENE; Flags: Precursor (At3g51550)		2.250
Cs8g07490		Protein CASPARIAN STRIP INTEGRITY FACTOR 1 {ECO:0000303|PubMed:28104889}; Flags: Precursor (At4g34600)		1.150
orange1.1t02709	K04733 interleukin-1 receptor-associated kinase 4 [EC:2.7.11.1] | (RefSeq) hypothetical protein (A)	Receptor-like protein kinase FERONIA; EC=2.7.11.1; Protein SIRENE; Flags: Precursor (At3g51550)	2.648	1.833
orange1.1t02713	K04733 interleukin-1 receptor-associated kinase 4 [EC:2.7.11.1] | (RefSeq) hypothetical protein (A)	Receptor-like protein kinase FERONIA; EC=2.7.11.1; Protein SIRENE; Flags: Precursor (At3g51550)	2.755	3.129
orange1.1t02715	K04733 interleukin-1 receptor-associated kinase 4 [EC:2.7.11.1] | (RefSeq) hypothetical protein (A)	Receptor-like protein kinase FERONIA; EC=2.7.11.1; Protein SIRENE; Flags: Precursor (At3g51550)		1.069

Plant-type cell wall [GO:0009505; P = 0.0127 (P3CR vs. P3R) and 0.0275 (P5CR vs. P5R)]				
Cs1g13910	K04733 interleukin-1 receptor-associated kinase 4 [EC:2.7.11.1] | (RefSeq) wall-associated receptor kinase-like 10 isoform X1 (A)	Wall-associated receptor kinase-like 2; EC=2.7.11.-; Flags: Precursor (At1g79680)	1.393	
Cs1g17350	K15333 tRNA guanosine-2'-O-methyltransferase [EC:2.1.1.34] | (RefSeq) uncharacterized protein LOC104224633 (A)	Subtilisin-like protease SBT1.4 {ECO:0000303|PubMed:16193095}; EC=3.4.21.- {ECO:0000255|PROSITE-ProRule:PRU10082}; Subtilase subfamily 1 member 4 {ECO:0000303|PubMed:16193095}; AtSBT1.4 {ECO:0000303|PubMed:16193095}; Flags: Precursor (At3g14067)	1.240	
Cs1g25930	K00430 peroxidase [EC:1.11.1.7] | (RefSeq) peroxidase 64-like (A)	Peroxidase 64; Atperox P64; EC=1.11.1.7; ATP17a; PRXR4; Flags: Precursor (At5g42180)		2.616
Cs2g03340	K19891 glucan endo-1,3-beta-glucosidase 1/2/3 [EC:3.2.1.39] | (RefSeq) hypothetical protein (A)	Glucan endo-1,3-beta-glucosidase 11; EC=3.2.1.39; (1->3)-beta-glucan endohydrolase 11; (1->3)-beta-glucanase 11; Beta-1,3-endoglucanase 11; Beta-1,3-glucanase 11; Flags: Precursor (At1g30080)	1.102	-1.069
Cs2g05220	K00430 peroxidase [EC:1.11.1.7] | (RefSeq) peroxidase 19 (A)	Peroxidase 19; Atperox P19; EC=1.11.1.7; ATP51; Flags: Precursor (At2g34060)		1.187
Cs2g05840		Protein RALF-like 33; Flags: Precursor (At1g28270)	1.972	
Cs2g09510	K19891 glucan endo-1,3-beta-glucosidase 1/2/3 [EC:3.2.1.39] | (RefSeq) hypothetical protein (A)	Glucan endo-1,3-beta-glucosidase 13; EC=3.2.1.39; (1->3)-beta-glucan endohydrolase 13; (1->3)-beta-glucanase 13; Beta-1,3-endoglucanase 13; Beta-1,3-glucanase 13; Flags: Precursor (At4g05430)		-2.165
Cs2g10950	K13420 LRR receptor-like serine/threonine-protein kinase FLS2 [EC:2.7.11.1] | (RefSeq) hypothetical protein (A)	LRR receptor-like serine/threonine-protein kinase GSO1 {ECO:0000303|PubMed:18088309}; EC=2.7.11.1 {ECO:0000255|PROSITE-ProRule:PRU00159}; Protein GASSHO 1 {ECO:0000303|PubMed:18088309}; Protein SCHENGEN 3 {ECO:0000303|PubMed:25233277}; Flags: Precursor (At4g20140)		1.586
Cs2g11910	K13420 LRR receptor-like serine/threonine-protein kinase FLS2 [EC:2.7.11.1] | (RefSeq) LRR receptor-like serine/threonine-protein kinase FLS2 (A)	LRR receptor-like serine/threonine-protein kinase GSO1 {ECO:0000303|PubMed:18088309}; EC=2.7.11.1 {ECO:0000255|PROSITE-ProRule:PRU00159}; Protein GASSHO 1 {ECO:0000303|PubMed:18088309}; Protein SCHENGEN 3 {ECO:0000303|PubMed:25233277}; Flags: Precursor (At4g20140)	1.313	1.890
Cs2g12290	K07407 alpha-galactosidase [EC:3.2.1.22] | (RefSeq) alpha-galactosidase 1-like (A)	Alpha-galactosidase 1 {ECO:0000303|PubMed:15034167}; AtAGAL1 {ECO:0000303|PubMed:15034167}; EC=3.2.1.22 {ECO:0000250|UniProtKB:Q9FXT4}; Alpha-D-galactoside galactohydrolase 1 {ECO:0000305}; Melibiase 1 {ECO:0000305}; Flags: Precursor (At5g08380)	-1.333	-2.188

Cs2g14530	K01191 alpha-mannosidase [EC:3.2.1.24] | (RefSeq) alpha-mannosidase-like (A)	Alpha-mannosidase At3g26720 {ECO:0000305}; EC=3.2.1.24 {ECO:0000269|PubMed:16233119}; Flags: Precursor (At3g26720)		1.577
Cs2g21660	K19891 glucan endo-1,3-beta-glucosidase 1/2/3 [EC:3.2.1.39] | (RefSeq) hypothetical protein (A)	Glucan endo-1,3-beta-glucosidase 11; EC=3.2.1.39; (1->3)-beta-glucan endohydrolase 11; (1->3)-beta-glucanase 11; Beta-1,3-endoglucanase 11; Beta-1,3-glucanase 11; Flags: Precursor (At2g05790)		-2.259
Cs2g21810	K00430 peroxidase [EC:1.11.1.7] | (RefSeq) hypothetical protein (A)	Peroxidase 27; Atperox P27; EC=1.11.1.7; ATP12a; PRXR7; Flags: Precursor (At3g01190)		1.538
Cs2g23510	K03006 DNA-directed RNA polymerase II subunit RPB1 [EC:2.7.7.6] | (RefSeq) leucine-rich repeat extensin-like protein 1 (A)	Leucine-rich repeat extensin-like protein 4; AtLRX4; LRR/EXTENSIN4; Cell wall hydroxyproline-rich glycoprotein; Flags: Precursor (At4g28380)		1.223
Cs3g10420	K19882 O-palmitoleoyl-L-serine hydrolase [EC:3.1.1.98] | (RefSeq) hypothetical protein (A)	Pectin acetylesterase 8 {ECO:0000303|PubMed:25115560}; EC=3.1.1.- {ECO:0000305}; Flags: Precursor (At5g45280)	1.766	
Cs3g10430	K19882 O-palmitoleoyl-L-serine hydrolase [EC:3.1.1.98] | (RefSeq) pectin acetylesterase 7-like (A)	Pectin acetylesterase 8 {ECO:0000303|PubMed:25115560}; EC=3.1.1.- {ECO:0000305}; Flags: Precursor (At5g45280)	1.572	
Cs3g14000	K04733 interleukin-1 receptor-associated kinase 4 [EC:2.7.11.1] | (RefSeq) non-functional pseudokinase ZED1-like (A)	Wall-associated receptor kinase-like 2; EC=2.7.11.-; Flags: Precursor (At3g57730)	1.205	
Cs3g15800	K15333 tRNA guanosine-2'-O-methyltransferase [EC:2.1.1.34] | (RefSeq) uncharacterized protein LOC104224633 (A)	Subtilisin-like protease SBT1.7 {ECO:0000303|PubMed:16193095}; EC=3.4.21.- {ECO:0000305}; Cucumisin-like serine protease; Subtilase subfamily 1 member 7 {ECO:0000303|PubMed:16193095}; AtSBT1.7 {ECO:0000303|PubMed:16193095}; Subtilisin-like serine protease 1 {ECO:0000303|PubMed:12702015}; At-SLP1 {ECO:0000303|PubMed:12702015}; Flags: Precursor (At5g67360)		1.848
Cs3g18070	K00020 3-hydroxyisobutyrate dehydrogenase [EC:1.1.1.31] | (RefSeq) 6-phosphogluconate dehydrogenase-like protein (A)	Protein EXORDIUM; Flags: Precursor (At4g08950)		1.172
Cs3g21730	K00430 peroxidase [EC:1.11.1.7] | (RefSeq) peroxidase 12 (A)	Peroxidase 12; Atperox P12; EC=1.11.1.7; ATP4a; PRXR6; Flags: Precursor (At1g71695)		-1.066
Cs3g23350	K15333 tRNA guanosine-2'-O-methyltransferase [EC:2.1.1.34] | (RefSeq) uncharacterized protein LOC104224633 (A)	Subtilisin-like protease SBT1.7 {ECO:0000303|PubMed:16193095}; EC=3.4.21.- {ECO:0000305}; Cucumisin-like serine protease; Subtilase subfamily 1 member 7 {ECO:0000303|PubMed:16193095}; AtSBT1.7 {ECO:0000303|PubMed:16193095}; Subtilisin-like serine protease 1 {ECO:0000303|PubMed:12702015}; At-SLP1 {ECO:0000303|PubMed:12702015}; Flags: Precursor (At5g67360)	1.291	-7.781

Cs3g24860	K04733 interleukin-1 receptor-associated kinase 4 [EC:2.7.11.1] | (RefSeq) probable LRR receptor-like serine/threonine-protein kinase At2g16250 (A)	Probable LRR receptor-like serine/threonine-protein kinase At2g16250; EC=2.7.11.1; Flags: Precursor (At2g16250)		1.135
Cs3g26600	K00430 peroxidase [EC:1.11.1.7] | (RefSeq) peroxidase 64 (A)	Peroxidase 64; Atperox P64; EC=1.11.1.7; ATP17a; PRXR4; Flags: Precursor (At5g42180)		1.204
Cs4g09340	K04733 interleukin-1 receptor-associated kinase 4 [EC:2.7.11.1] | (RefSeq) probable inactive receptor kinase At2g26730 (A)	Probable inactive receptor kinase At3g08680; Flags: Precursor (At3g08680)		-1.605
Cs4g09780	K19891 glucan endo-1,3-beta-glucosidase 1/2/3 [EC:3.2.1.39] | (RefSeq) hypothetical protein (A)	Glucan endo-1,3-beta-glucosidase 13; EC=3.2.1.39; (1->3)-beta-glucan endohydrolase 13; (1->3)-beta-glucanase 13; Beta-1,3-endoglucanase 13; Beta-1,3-glucanase 13; Flags: Precursor (At5g55180)		-2.309
Cs4g11970	K01188 beta-glucosidase [EC:3.2.1.21] | (RefSeq) putative beta-glucosidase 41 (A)	Putative beta-glucosidase 41; AtBGLU41; EC=3.2.1.21; Flags: Precursor (At5g54570)	2.732	3.222
Cs4g13170	K09422 transcription factor MYB, plant | (RefSeq) transcription factor RAX2-like (A)	Transcription factor MYB36 {ECO:0000303|PubMed:9839469}; Myb-related protein 36 {ECO:0000303|PubMed:9839469}; AtMYB36 {ECO:0000303|PubMed:9839469} (At5g65790)	2.853	
Cs4g17860	K00430 peroxidase [EC:1.11.1.7] | (RefSeq) peroxidase 25 (A)	Peroxidase 25; Atperox P25; EC=1.11.1.7; Flags: Precursor (At2g41480)		-1.000
Cs5g12400	K04733 interleukin-1 receptor-associated kinase 4 [EC:2.7.11.1] | (RefSeq) G-type lectin S-receptor-like serine/threonine-protein kinase SD2-5 (A)	EP1-like glycoprotein 3 {ECO:0000303|PubMed:15276441}; Curculin-like (Mannose-binding) lectin family protein {ECO:0000303|PubMed:23738689}; Putative receptor-like protein kinase-like protein {ECO:0000303|PubMed:12833529}; Flags: Precursor (At1g78850)	-1.844	
Cs5g12420	K04733 interleukin-1 receptor-associated kinase 4 [EC:2.7.11.1] | (RefSeq) G-type lectin S-receptor-like serine/threonine-protein kinase SD2-5 (A)	EP1-like glycoprotein 3 {ECO:0000303|PubMed:15276441}; Curculin-like (Mannose-binding) lectin family protein {ECO:0000303|PubMed:23738689}; Putative receptor-like protein kinase-like protein {ECO:0000303|PubMed:12833529}; Flags: Precursor (At1g78850)	1.580	
Cs5g23280	K00430 peroxidase [EC:1.11.1.7] | (RefSeq) hypothetical protein (A)	Probable peroxidase 61; Atperox P61; EC=1.11.1.7; Flags: Precursor (At5g24070)	-1.229	
Cs5g32270	K00430 peroxidase [EC:1.11.1.7] | (RefSeq) peroxidase 3 (A)	Peroxidase 3; Atperox P3; EC=1.11.1.7; ATPRC; RCI3A; Rare cold-inducible protein; Flags: Precursor (At1g05260)		-1.201
Cs5g32900	K19765 heat shock factor-binding protein 1 | (RefSeq) hypothetical protein (A)	Protein RALF-like 33; Flags: Precursor (At1g02900)		1.449
Cs6g02700	K04733 interleukin-1 receptor-associated kinase 4 [EC:2.7.11.1] | (RefSeq) wall-associated receptor	Wall-associated receptor kinase-like 2; EC=2.7.11.-; Flags: Precursor (At1g16130)	3.081	4.760

	kinase-like 1 (A)			
Cs6g06530	K20628 expansin | (RefSeq) expansin-A4 (A)	Expansin-A4; AtEXPA4; Alpha-expansin-4; At-EXP4; AtEx4; Ath-ExpAlpha-1.6; Flags: Precursor (At2g39700)	-1.153	
Cs6g16270	K01209 alpha-N-arabinofuranosidase [EC:3.2.1.55] | (RefSeq) alpha-L-arabinofuranosidase 1-like (A)	Alpha-L-arabinofuranosidase 1; AtASD1; EC=3.2.1.55; Beta-D-xylosidase; EC=3.2.1.-; Flags: Precursor (At3g10740)		2.031
Cs6g17850	K05350 beta-glucosidase [EC:3.2.1.21] | (RefSeq) hypothetical protein (A)	Beta-glucosidase 44; AtBGLU44; EC=3.2.1.21; Flags: Precursor (At3g18080)		-2.746
Cs7g10500	K04733 interleukin-1 receptor-associated kinase 4 [EC:2.7.11.1] | (RefSeq) receptor protein kinase-like protein ZAR1 (A)	Receptor protein kinase-like protein ZAR1 {ECO:0000303|PubMed:27014878}; EC=2.7.11.1 {ECO:0000305}; Protein ZYGOTIC ARREST 1 {ECO:0000303|PubMed:27014878}; Flags: Precursor (At1g67510)		1.783
Cs7g21880	K19791 iron transport multicopper oxidase | (RefSeq) monocopper oxidase-like protein SKU5 (A)	Monocopper oxidase-like protein SKU5; Skewed roots; Flags: Precursor (At4g12420)	1.963	-7.098
Cs7g21890	K19791 iron transport multicopper oxidase | (RefSeq) monocopper oxidase-like protein SKU5 (A)	Monocopper oxidase-like protein SKU5; Skewed roots; Flags: Precursor (At5g48450)	2.325	-3.812
Cs8g02110	K20628 expansin | (RefSeq) expansin-B15-like (A)	Expansin-like A3; At-EXPL3; AtEXLA3; AtEXPL3; Ath-ExpBeta-2.3; Flags: Precursor (At3g45960)	-1.938	-1.158
Cs8g06080	K15333 tRNA guanosine-2'-O-methyltransferase [EC:2.1.1.34] | (RefSeq) uncharacterized protein LOC104224633 (A)	Subtilisin-like protease SBT1.7 {ECO:0000303|PubMed:16193095}; EC=3.4.21.- {ECO:0000305}; Cucumisin-like serine protease; Subtilase subfamily 1 member 7 {ECO:0000303|PubMed:16193095}; AtSBT1.7 {ECO:0000303|PubMed:16193095}; Subtilisin-like serine protease 1 {ECO:0000303|PubMed:12702015}; At-SLP1 {ECO:0000303|PubMed:12702015}; Flags: Precursor (At5g67360)	1.927	-5.188
Cs8g07490		Protein CASPARIAN STRIP INTEGRITY FACTOR 1 {ECO:0000303|PubMed:28104889}; Flags: Precursor (At4g34600)		1.150
Cs8g09520	K04733 interleukin-1 receptor-associated kinase 4 [EC:2.7.11.1] | (RefSeq) G-type lectin S-receptor-like serine/threonine-protein kinase SD2-5 (A)	EP1-like glycoprotein 4 {ECO:0000305}; Curculin-like (Mannose-binding) lectin family protein {ECO:0000303|PubMed:23738689}; Flags: Precursor (At1g78860)	-1.149	-1.801
Cs9g02570	K15920 xylan 1,4-beta-xylosidase [EC:3.2.1.37] | (RefSeq) beta-D-xylosidase 1-like (A)	Beta-D-xylosidase 1; AtBXL1; EC=3.2.1.-; Alpha-L-arabinofuranosidase; EC=3.2.1.55; Flags: Precursor (At5g49360)	3.107	1.508
Cs9g05130	K00430 peroxidase [EC:1.11.1.7] | (RefSeq) peroxidase 3-like (A)	Peroxidase 3; Atperox P3; EC=1.11.1.7; ATPRC; RCI3A; Rare cold-inducible protein; Flags: Precursor (At1g05260)		1.261
Cs9g14540	K04733 interleukin-1 receptor-associated kinase 4 [EC:2.7.11.1] | (RefSeq) hypothetical protein (A)	Wall-associated receptor kinase-like 2; EC=2.7.11.-; Flags: Precursor (At1g16130)	1.558	1.728

Cs9g16590	K00430 peroxidase [EC:1.11.1.7] | (RefSeq) peroxidase 66 (A)	Peroxidase 66; Atperox P66; EC=1.11.1.7; ATP27a; Flags: Precursor (At5g51890)	2.440	5.362
novel.1682	K04733 interleukin-1 receptor-associated kinase 4 [EC:2.7.11.1] | (RefSeq) G-type lectin S-receptor-like serine/threonine-protein kinase SD2-5 (A)	EP1L4_ARATH EP1-like glycoprotein 4 {ECO:0000305}; Curculin-like (Mannose-binding) lectin family protein {ECO:0000303|PubMed:23738689}; Flags: Precursor (At1g78860)		-2.884
novel.2027	K19891 glucan endo-1,3-beta-glucosidase 1/2/3 [EC:3.2.1.39] | (RefSeq) glucan endo-1,3-beta-glucosidase 3-like (A)	E1310_ARATH Glucan endo-1,3-beta-glucosidase 10; EC=3.2.1.39; (1->3)-beta-glucan endohydrolase 10; (1->3)-beta-glucanase 10; Beta-1,3-endoglucanase 10; Beta-1,3-glucanase 10; Putative plasmodesmal associated protein {ECO:0000303|PubMed:17270015}; AtBG_ppap {ECO:0000303|PubMed:17270015}; Flags: Precursor (At2g05790)		-1.014
novel.2352		ELD1_ARATH Glycosyltransferase-like KOBITO 1 {ECO:0000303|PubMed:12215501, ECO:0000303|PubMed:22457425}; EC=2.4.1.- {ECO:0000305|PubMed:22457425}; Protein ABA INSENSITIVE 8 {ECO:0000303|PubMed:14742875}; Protein ELONGATION DEFECTIVE 1 {ECO:0000303|PubMed:10859181}; Flags: Precursor (At3g08550)		-1.008
orange1.1t00281	K15333 tRNA guanosine-2'-O-methyltransferase [EC:2.1.1.34] | (RefSeq) uncharacterized LOC105043884 (A)	Subtilisin-like protease SBT4.14 {ECO:0000303|PubMed:16193095}; EC=3.4.21.- {ECO:0000305}; Cucumisin-like protein; Subtilase subfamily 4 member 14 {ECO:0000303|PubMed:16193095}; AtSBT4.14 {ECO:0000303|PubMed:16193095}; Xylem serine proteinase 1 {ECO:0000303|PubMed:10889267}; AtXSP1 {ECO:0000303|PubMed:10889267}; Flags: Precursor (At4g00230)	2.335	2.890
orange1.1t00617	K00384 thioredoxin reductase (NADPH) [EC:1.8.1.9] | (RefSeq) thioredoxin reductase 1-like (A)	Germin-like protein subfamily 2 member 4; Flags: Precursor (At3g62020)	2.088	4.418
orange1.1t01614	K04733 interleukin-1 receptor-associated kinase 4 [EC:2.7.11.1] | (RefSeq) EP1-like glycoprotein 2 (A)	EP1-like glycoprotein 2 {ECO:0000305}; Curculin-like (Mannose-binding) lectin family protein {ECO:0000303|PubMed:23738689}; Flags: Precursor (At1g78830)	-1.016	
orange1.1t01658	K15920 xylan 1,4-beta-xylosidase [EC:3.2.1.37] | (RefSeq) probable beta-D-xylosidase 2 (A)	Probable beta-D-xylosidase 2; AtBXL2; EC=3.2.1.-; Flags: Precursor (At1g02640)		1.297
orange1.1t03602	K19882 O-palmitoleoyl-L-serine hydrolase [EC:3.1.1.98] | (RefSeq) pectin acetylesterase 8-like (A)	Pectin acetylesterase 8 {ECO:0000303|PubMed:25115560}; EC=3.1.1.- {ECO:0000305}; Flags: Precursor (At5g45280)	1.516	
orange1.1t03603	K19882 O-palmitoleoyl-L-serine hydrolase [EC:3.1.1.98] | (RefSeq) hypothetical protein (A)	Pectin acetylesterase 8 {ECO:0000303|PubMed:25115560}; EC=3.1.1.- {ECO:0000305}; Flags: Precursor (At5g26665)	1.503	
Plant-type cell wall organization or biogenesis [GO:0071669; P < 0.0001 (P3CR vs. P3R) and 0.0001 (P5CR vs. P5R)]				

Cs1g05500	K20890 xylan alpha-glucuronosyltransferase [EC:2.4.1.-] | (RefSeq) UDP-glucuronate:xylan alpha-glucuronosyltransferase 1 (A)	UDP-glucuronate:xylan alpha-glucuronosyltransferase 1; UDP-GlcA:xylan glucuronyltransferase 1; EC=2.4.1.-; Glycogenin-like protein 1; Plant glycogenin-like starch initiation protein 1; Protein GLUCURONIC ACID SUBSTITUTION OF XYLAN 1; AtGUX1 (At3g18660)	1.889	3.881
Cs1g07100	K20628 expansin | (RefSeq) expansin-A4-like (A)	Expansin-A12; AtEXPA12; Alpha-expansin-12; At-EXP12; AtEx12; Ath-ExpAlpha-1.24; Expansin-S2; Flags: Precursor (At3g15370)		-1.275
Cs1g25930	K00430 peroxidase [EC:1.11.1.7] | (RefSeq) peroxidase 64-like (A)	Peroxidase 64; Atperox P64; EC=1.11.1.7; ATP17a; PRXR4; Flags: Precursor (At5g42180)		2.616
Cs2g01800	K22733 magnesium transporter | (RefSeq) microtubule-associated protein 70-5-like (A)	Microtubule-associated protein 70-5; AtMAP70-5; 70 kDa microtubule-associated protein 5 (At4g17220)		1.892
Cs2g04590	K10999 cellulose synthase A [EC:2.4.1.12] | (RefSeq) cellulose synthase A catalytic subunit 8 [UDP-forming] (A)	Cellulose synthase A catalytic subunit 8 [UDP-forming]; AtCesA8; EC=2.4.1.12; Protein IRREGULAR XYLEM 1; AtIRX1; Protein LEAF WILTING 2 (At4g18780)	1.721	4.523
Cs2g05220	K00430 peroxidase [EC:1.11.1.7] | (RefSeq) peroxidase 19 (A)	Peroxidase 19; Atperox P19; EC=1.11.1.7; ATP51; Flags: Precursor (At2g34060)		1.187
Cs2g05490		NAC domain-containing protein 73 {ECO:0000303|PubMed:15029955}; ANAC073 {ECO:0000303|PubMed:15029955}; Protein SECONDARY WALL-ASSOCIATED NAC DOMAIN PROTEIN 2 {ECO:0000303|PubMed:18952777} (At4g28500)	1.261	2.000
Cs2g16190		NAC domain-containing protein 73 {ECO:0000303|PubMed:15029955}; ANAC073 {ECO:0000303|PubMed:15029955}; Protein SECONDARY WALL-ASSOCIATED NAC DOMAIN PROTEIN 2 {ECO:0000303|PubMed:18952777} (At4g28500)	2.055	4.527
Cs2g19510	K15400 omega-hydroxypalmitate O-feruloyl transferase [EC:2.3.1.188] | (RefSeq) omega-hydroxypalmitate O-feruloyl transferase-like (A)	Omega-hydroxypalmitate O-feruloyl transferase; EC=2.3.1.188; Omega-hydroxyacid hydroxycinnamoyltransferase; Protein ALIPHATIC SUBERIN FERULOYL TRANSFERASE (At5g41040)		3.666
Cs2g21100	K10891 fanconi anemia group D2 protein | (RefSeq) Fanconi anemia group D2 protein homolog (A)	Cellulose synthase-like protein B4; AtCslB4; EC=2.4.1.- (At2g32540)		-1.449
Cs2g21810	K00430 peroxidase [EC:1.11.1.7] | (RefSeq) hypothetical protein (A)	Peroxidase 27; Atperox P27; EC=1.11.1.7; ATP12a; PRXR7; Flags: Precursor (At3g01190)		1.538
Cs2g25720	K15400 omega-hydroxypalmitate O-feruloyl transferase [EC:2.3.1.188] | (RefSeq) omega-hydroxypalmitate O-feruloyl transferase (A)	Omega-hydroxypalmitate O-feruloyl transferase; EC=2.3.1.188; Omega-hydroxyacid hydroxycinnamoyltransferase; Protein ALIPHATIC SUBERIN FERULOYL TRANSFERASE (At5g41040)		2.867
Cs3g09000	K20924 cellulose synthase-like protein [EC:2.4.1.-] |	Cellulose synthase-like protein D3; AtCslD3; EC=2.4.1.-; Protein KOJAK		1.847

	(RefSeq) hypothetical protein (A)	(At3g03050)		
Cs3g16440	K00826 branched-chain amino acid aminotransferase [EC:2.6.1.42] | (RefSeq) branched-chain-amino-acid aminotransferase 2, chloroplastic (A)	Branched-chain-amino-acid aminotransferase 2, chloroplastic; Atbcat-2; EC=2.6.1.42; Flags: Precursor (At1g10070)		1.092
Cs3g20590	K09422 transcription factor MYB, plant | (RefSeq) myb-related protein 308-like (A)	Transcription factor MYB52 {ECO:0000303|PubMed:9839469}; Myb-related protein 52 {ECO:0000303|PubMed:9839469}; AtMYB52 {ECO:0000303|PubMed:9839469}; Protein ABA-HYPERSENSITIVE 1 {ECO:0000303|PubMed:21399993} (At1g17950)	1.675	4.918
Cs3g20910	K20890 xylan alpha-glucuronosyltransferase [EC:2.4.1.-] | (RefSeq) putative UDP-glucuronate:xylan alpha-glucuronosyltransferase 3 (A)	Putative UDP-glucuronate:xylan alpha-glucuronosyltransferase 3; UDP-GlcA:xylan glucuronyltransferase 3; EC=2.4.1.-; Glycogenin-like protein 3; Plant glycogenin-like starch initiation protein 2; Protein GLUCURONIC ACID SUBSTITUTION OF XYLAN 3; AtGUX3 (At1g77130)		1.189
Cs3g21730	K00430 peroxidase [EC:1.11.1.7] | (RefSeq) peroxidase 12 (A)	Peroxidase 12; Atperox P12; EC=1.11.1.7; ATP4a; PRXR6; Flags: Precursor (At1g71695)		-1.066
Cs3g23600	K20890 xylan alpha-glucuronosyltransferase [EC:2.4.1.-] | (RefSeq) UDP-glucuronate:xylan alpha-glucuronosyltransferase 2 (A)	UDP-glucuronate:xylan alpha-glucuronosyltransferase 2; UDP-GlcA:xylan glucuronyltransferase 2; EC=2.4.1.-; Glycogenin-like protein 2; Plant glycogenin-like starch initiation protein 3; Protein GLUCURONIC ACID SUBSTITUTION OF XYLAN 2; AtGUX2 (At4g33330)	1.620	4.416
Cs3g26600	K00430 peroxidase [EC:1.11.1.7] | (RefSeq) peroxidase 64 (A)	Peroxidase 64; Atperox P64; EC=1.11.1.7; ATP17a; PRXR4; Flags: Precursor (At5g42180)		1.204
Cs3g26610	K20628 expansin | (RefSeq) expansin-A4-like (A)	Expansin-A1; Alpha-expansin-1; OsEXP1; OsEXPA1; OsaEXPa1.16; RiExA; Flags: Precursor (At1g20190)	-1.740	-1.742
Cs4g01990	K10999 cellulose synthase A [EC:2.4.1.12] | (RefSeq) cellulose synthase A catalytic subunit 7 [UDP-forming] (A)	Cellulose synthase A catalytic subunit 7 [UDP-forming]; AtCesA7; EC=2.4.1.12; Protein FRAGILE FIBER 5; Protein IRREGULAR XYLEM 3; AtIRX3 (At5g17420)	1.503	3.892
Cs4g08540	K10999 cellulose synthase A [EC:2.4.1.12] | (RefSeq) cellulose synthase A catalytic subunit 7 [UDP-forming]-like (A)	Cellulose synthase-like protein E1; AtCslE1; EC=2.4.1.- (At1g55850)	-2.353	
Cs4g08560	K20923 mixed-linked glucan synthase [EC:2.4.1.-] | (RefSeq) probable mixed-linked glucan synthase 6 (A)	Cellulose synthase-like protein E1; AtCslE1; EC=2.4.1.- (At1g55850)		1.596
Cs5g01350	K00521 ferric-chelate reductase [EC:1.16.1.7] | (RefSeq) ferric reduction oxidase 7, chloroplastic-like (A)	NAC domain-containing protein 12 {ECO:0000303|PubMed:15029955}; ANAC012 {ECO:0000303|PubMed:15029955}; Protein NAC SECONDARY WALL THICKENING PROMOTING 3 {ECO:0000303|PubMed:17237351};	1.426	4.287

		Protein SECONDARY WALL-ASSOCIATED NAC DOMAIN PROTEIN 1 {ECO:0000303|PubMed:17114348} (At1g32770)		
Cs5g01970	K10999 cellulose synthase A [EC:2.4.1.12] | (RefSeq) cellulose synthase A catalytic subunit 7 [UDP-forming]-like isoform X1 (A)	Cellulose synthase-like protein G3; AtCslG3; EC=2.4.1.- (At4g23990)	1.617	
Cs5g03130	K09422 transcription factor MYB, plant | (RefSeq) myb-related protein Myb4 (A)	Transcription factor MYB26 {ECO:0000305}; Myb-related protein 26 {ECO:0000305}; AtMYB26 {ECO:0000303|PubMed:11597504}; Protein MALE STERILE 35 {ECO:0000303|PubMed:12753590} (At1g63910)	1.519	2.426
Cs5g09360	K01051 pectinesterase [EC:3.1.1.11] | (RefSeq) pectinesterase 3-like (A)	Pectinesterase inhibitor 10 {ECO:0000305}; Pectin methylesterase inhibitor 10 {ECO:0000303|PubMed:28082716}; AtPMEI10 {ECO:0000303|PubMed:28082716}; Flags: Precursor (At1g62763)	-1.021	-2.490
Cs5g10820	K20628 expansin | (RefSeq) hypothetical protein (A)	Expansin-A1; AtEXPA1; Alpha-expansin-1; At-EXP1; AtEx1; Ath-ExpAlpha-1.2; Flags: Precursor (At1g69530)		-2.035
Cs5g12220	K17964 leucine-rich PPR motif-containing protein, mitochondrial | (RefSeq) pentatricopeptide repeat-containing protein At3g18110, chloroplastic-like (A)	COBRA-like protein 4; Flags: Precursor (At5g15630)	1.039	3.198
Cs5g12230	K17964 leucine-rich PPR motif-containing protein, mitochondrial | (RefSeq) pentatricopeptide repeat-containing protein At3g18110, chloroplastic-like (A)	COBRA-like protein 4; Flags: Precursor (At5g15630)		2.418
Cs5g16420	K09422 transcription factor MYB, plant | (RefSeq) hypothetical protein (A)	Transcription factor MYB52 {ECO:0000303|PubMed:9839469}; Myb-related protein 52 {ECO:0000303|PubMed:9839469}; AtMYB52 {ECO:0000303|PubMed:9839469}; Protein ABA-HYPERSENSITIVE 1 {ECO:0000303|PubMed:21399993} (At1g17950)	1.560	3.254
Cs5g18940	K00521 ferric-chelate reductase [EC:1.16.1.7] | (RefSeq) ferric reduction oxidase 7, chloroplastic-like (A)	Protein BEARSKIN2; NAC domain-containing protein 70; ANAC070 (At4g10350)	-1.411	
Cs5g23280	K00430 peroxidase [EC:1.11.1.7] | (RefSeq) hypothetical protein (A)	Probable peroxidase 61; Atperox P61; EC=1.11.1.7; Flags: Precursor (At5g24070)	-1.229	
Cs5g25090	K20924 cellulose synthase-like protein [EC:2.4.1.-] | (RefSeq) hypothetical protein (A)	Cellulose synthase-like protein D3; AtCslD3; EC=2.4.1.-; Protein KOJAK (At3g03050)		2.184
Cs5g28040	K14963 COMPASS component SWD3 | (RefSeq) transcriptional corepressor LEUNIG_HOMOLOG-like (A)	Transcriptional corepressor LEUNIG_HOMOLOG; Protein MUCILAGE-MODIFIED 1 {ECO:0000303|PubMed:11706181} (At2g32700)		1.712

Cs5g29200	K10999 cellulose synthase A [EC:2.4.1.12] | (RefSeq) cellulose synthase A catalytic subunit 4 [UDP-forming] (A)	Cellulose synthase A catalytic subunit 4 [UDP-forming]; AtCesA4; EC=2.4.1.12; Protein IRREGULAR XYLEM 5; AtIRX5 (At5g17420)	1.736	4.872
Cs5g32270	K00430 peroxidase [EC:1.11.1.7] | (RefSeq) peroxidase 3 (A)	Peroxidase 3; Atperox P3; EC=1.11.1.7; ATPRC; RCI3A; Rare cold-inducible protein; Flags: Precursor (At1g05260)		-1.201
Cs6g02570		CRIB domain-containing protein RIC4; ROP-interactive CRIB motif-containing protein 4; Target of ROP protein RIC4 (At5g16490)		1.512
Cs6g04310	K19519 transforming growth factor-beta-induced protein | (RefSeq) fasciclin-like arabinogalactan protein 12 (A)	Fasciclin-like arabinogalactan protein 11; Flags: Precursor (At5g03170)	1.066	2.802
Cs6g04870	K20869 putative beta-1,4-xylosyltransferase IRX9 [EC:2.4.2.-] | (RefSeq) probable beta-1,4-xylosyltransferase IRX9 (A)	Probable beta-1,4-xylosyltransferase IRX9; EC=2.4.2.-; Protein IRREGULAR XYLEM 9; Xylan xylosyltransferase IRX9 (At2g37090)	1.695	3.472
Cs6g06530	K20628 expansin | (RefSeq) expansin-A4 (A)	Expansin-A4; AtEXPA4; Alpha-expansin-4; At-EXP4; AtEx4; Ath-ExpAlpha-1.6; Flags: Precursor (At2g39700)	-1.153	
Cs6g07800	K05909 laccase [EC:1.10.3.2] | (RefSeq) hypothetical protein (A)	Laccase-4; EC=1.10.3.2; Benzenediol:oxygen oxidoreductase 4; Diphenol oxidase 4; Protein IRREGULAR XYLEM 12; Urishiol oxidase 4; Flags: Precursor (At2g38080)	1.504	3.815
Cs6g08690	K16573 gamma-tubulin complex component 6 | (RefSeq) uncharacterized LOC103722329 (A)	Protein trichome birefringence-like 3 (At5g01360)	1.007	2.548
Cs6g17170	K16573 gamma-tubulin complex component 6 | (RefSeq) uncharacterized LOC103722329 (A)	Protein trichome birefringence-like 33 (At2g40320)		-1.021
Cs7g01800	K09422 transcription factor MYB, plant | (RefSeq) transcription factor MYB46 (A)	Transcription factor MYB83 {ECO:0000303|PubMed:11597504}; Myb-related protein 83 {ECO:0000303|PubMed:11597504}; AtMYB83 {ECO:0000303|PubMed:11597504} (At3g08500)		1.661
Cs7g09030	K01213 galacturan 1,4-alpha-galacturonidase [EC:3.2.1.67] | (RefSeq) polygalacturonase At1g48100 (A)	Polygalacturonase At1g48100; PG; EC=3.2.1.15; Pectinase At1g48100; Flags: Precursor (At3g26610)	1.069	1.504
Cs7g29830	K23518 O-acetyl-ADP-ribose deacetylase [EC:3.1.1.106] | (RefSeq) hypothetical protein (A)	Expansin-A8; AtEXPA8; Alpha-expansin-8; At-EXP8; AtEx8; Ath-ExpAlpha-1.11; Flags: Precursor (At2g40610)		-1.251
Cs7g30510	K16573 gamma-tubulin complex component 6 | (RefSeq) uncharacterized LOC103722329 (A)	Protein trichome birefringence-like 33 (At2g40320)	1.388	2.970
Cs8g03410	K00826 branched-chain amino acid aminotransferase [EC:2.6.1.42] | (RefSeq) hypothetical protein (A)	Branched-chain-amino-acid aminotransferase 2, chloroplastic; Atbcat-2; EC=2.6.1.42; Flags: Precursor (At1g10070)	1.282	

Cs8g05250	K18801 glucuronoxylan 4-O-methyltransferase [EC:2.1.1.112] | (RefSeq) hypothetical protein (A)	Protein IRX15-LIKE (At5g67210)		-1.292
Cs8g09160		Proline-rich protein 2; AtPRP2; Flags: Precursor (At2g21140)	1.181	1.844
Cs8g10780	K20870 putative beta-1,4-xylosyltransferase IRX10 [EC:2.4.2.-] | (RefSeq) probable beta-1,4-xylosyltransferase IRX10 (A)	Probable beta-1,4-xylosyltransferase IRX10; EC=2.4.2.-; Glucuronoxylan glucuronosyltransferase 1; AtGUT1; Glucuronoxylan glucuronosyltransferase 2; Protein IRREGULAR XYLEM 10; Xylan xylosyltransferase IRX10 (At1g27440)	1.084	2.225
Cs8g15710	K20889 probable glucuronoxylan glucuronosyltransferase IRX7 [EC2.4.1.-] | (RefSeq) probable glucuronoxylan glucuronosyltransferase IRX7 (A)	Probable glucuronoxylan glucuronosyltransferase IRX7; EC=2.4.1.-; Protein FRAGILE FIBER 8; Protein IRREGULAR XYLEM 7 (At2g28110)		1.396
Cs8g16840	K19519 transforming growth factor-beta-induced protein | (RefSeq) fasciclin-like arabinogalactan protein 12 (A)	Fasciclin-like arabinogalactan protein 12; Flags: Precursor (At5g60490)		2.345
Cs8g19850	K05909 laccase [EC:1.10.3.2] | (RefSeq) hypothetical protein (A)	Laccase-4; EC=1.10.3.2; Benzenediol:oxygen oxidoreductase 4; Diphenol oxidase 4; Protein IRREGULAR XYLEM 12; Urishiol oxidase 4; Flags: Precursor (At2g38080)	1.651	3.446
Cs8g21030	K20642 Rho GTPase-activating protein 22/24/25 | (RefSeq) rho GTPase-activating protein 7-like (A)	Rho GTPase-activating protein 7; Rho-type GTPase-activating protein 7 (At3g13000)		1.296
Cs8g21040	K15400 omega-hydroxypalmitate O-feruloyl transferase [EC:2.3.1.188] | (RefSeq) omega-hydroxypalmitate O-feruloyl transferase-like (A)	Omega-hydroxypalmitate O-feruloyl transferase; EC=2.3.1.188; Omega-hydroxyacid hydroxycinnamoyltransferase; Protein ALIPHATIC SUBERIN FERULOYL TRANSFERASE (At1g78990)	1.742	2.780
Cs9g01630	K01213 galacturan 1,4-alpha-galacturonidase [EC:3.2.1.67] | (RefSeq) polygalacturonase At1g48100-like (A)	Polygalacturonase At1g48100; PG; EC=3.2.1.15; Pectinase At1g48100; Flags: Precursor (At1g48100)		1.625
Cs9g05130	K00430 peroxidase [EC:1.11.1.7] | (RefSeq) peroxidase 3-like (A)	Peroxidase 3; Atperox P3; EC=1.11.1.7; ATPRC; RCI3A; Rare cold-inducible protein; Flags: Precursor (At1g05260)		1.261
Cs9g08730	K10999 cellulose synthase A [EC:2.4.1.12] | (RefSeq) cellulose synthase A catalytic subunit 8 [UDP-forming] (A)	Cellulose synthase-like protein G2; AtCslG2; EC=2.4.1.- (At4g24000)	1.748	1.562
Cs9g08750	K10999 cellulose synthase A [EC:2.4.1.12] | (Kazusa) Lj5g3v1119490.1; - (A)	Cellulose synthase-like protein E1; EC=2.4.1.-; OsCslE1 (At4g24000)	1.204	
Cs9g08760	K10999 cellulose synthase A [EC:2.4.1.12] | (RefSeq) cellulose synthase A catalytic subunit 7 [UDP-forming]-like (A)	Cellulose synthase-like protein E6; EC=2.4.1.-; OsCslE6 (At4g24000)	1.054	1.806
Cs9g14460	K00521 ferric-chelate reductase [EC:1.16.1.7] | (RefSeq)	NAC domain-containing protein 7 {ECO:0000303|PubMed:15029955};		1.588

	ferric reduction oxidase 7, chloroplastic-like (A)	ANAC007 {ECO:0000303|PubMed:15029955}; Protein EMBRYO DEFECTIVE 2749 {ECO:0000303|Ref.7}; Protein VASCULAR RELATED NAC-DOMAIN 4 {ECO:0000303|PubMed:16103214} (At1g12260)		
Cs9g16590	K00430 peroxidase [EC:1.11.1.7] | (RefSeq) peroxidase 66 (A)	Peroxidase 66; Atperox P66; EC=1.11.1.7; ATP27a; Flags: Precursor (At5g51890)	2.440	5.362
novel.1550	K13648 alpha-1,4-galacturonosyltransferase [EC:2.4.1.43] | (RefSeq) hypothetical protein (A)	GAUT8_ARATH Galacturonosyltransferase 8; EC=2.4.1.-; Glycosyltransferase QUASIMODO1 (At3g25140)	-1.020	
novel.2352		ELD1_ARATH Glycosyltransferase-like KOBITO 1 {ECO:0000303|PubMed:12215501, ECO:0000303|PubMed:22457425}; EC=2.4.1.- {ECO:0000305|PubMed:22457425}; Protein ABA INSENSITIVE 8 {ECO:0000303|PubMed:14742875}; Protein ELONGATION DEFECTIVE 1 {ECO:0000303|PubMed:10859181}; Flags: Precursor (At3g08550)		-1.008
orange1.1t00561	K00521 ferric-chelate reductase [EC:1.16.1.7] | (RefSeq) ferric reduction oxidase 7, chloroplastic-like (A)	NAC domain-containing protein 43; ANAC043; Protein EMBRYO DEFECTIVE 2301; Protein NAC SECONDARY WALL THICKENING PROMOTING FACTOR 1 (At2g46770)		3.429
orange1.1t00615	K20628 expansin | (RefSeq) expansin-A4-like (A)	Putative expansin-A17; AtEXPA17; Alpha-expansin-17; At-EXP17; AtEx17; Ath-ExpAlpha-1.13; Flags: Precursor (At4g01630)	-2.165	-1.155
orange1.1t01738	K01213 galacturan 1,4-alpha-galacturonidase [EC:3.2.1.67] | (RefSeq) hypothetical protein (A)	Polygalacturonase At1g48100; PG; EC=3.2.1.15; Pectinase At1g48100; Flags: Precursor (At1g02460)	1.103	1.253
orange1.1t02619	K15400 omega-hydroxypalmitate O-feruloyl transferase [EC:2.3.1.188] | (RefSeq) hypothetical protein (A)	Omega-hydroxypalmitate O-feruloyl transferase; EC=2.3.1.188; Omega-hydroxyacid hydroxycinnamoyltransferase; Protein ALIPHATIC SUBERIN FERULOYL TRANSFERASE (At5g41040)	2.639	5.667
orange1.1t03392	K20870 putative beta-1,4-xylosyltransferase IRX10 [EC:2.4.2.-] | (RefSeq) uncharacterized protein LOC103936878 (A)	Probable beta-1,4-xylosyltransferase IRX10; EC=2.4.2.-; Glucuronoxylan glucuronosyltransferase 1; AtGUT1; Glucuronoxylan glucuronosyltransferase 2; Protein IRREGULAR XYLEM 10; Xylan xylosyltransferase IRX10 (At3g57630)		1.540
orange1.1t04064	K18801 glucuronoxylan 4-O-methyltransferase [EC:2.1.1.112] | (RefSeq) hypothetical protein (A)	Protein IRX15-LIKE (At5g67210)	1.609	3.639
Cell wall biogenesis [GO:0042546; P < 0.0001 (P3CR vs. P3R) and 0.0001 (P5CR vs. P5R)]				
Cs1g05500	K20890 xylan alpha-glucuronosyltransferase [EC:2.4.1.-] | (RefSeq) UDP-glucuronate:xylan alpha-glucuronosyltransferase 1 (A)	UDP-glucuronate:xylan alpha-glucuronosyltransferase 1; UDP-GlcA:xylan glucuronyltransferase 1; EC=2.4.1.-; Glycogenin-like protein 1; Plant glycogenin-like starch initiation protein 1; Protein GLUCURONIC ACID SUBSTITUTION OF XYLAN 1; AtGUX1 (At3g18660)	1.889	3.881

Cs1g21130	K08235 xyloglucan:xyloglucosyl transferase [EC:2.4.1.207] | (RefSeq) hypothetical protein (A)	Probable xyloglucan endotransglucosylase/hydrolase protein 6; At-XTH6; XTH-6; EC=2.4.1.207; Flags: Precursor (At5g65730)		-1.344
Cs2g01800	K22733 magnesium transporter | (RefSeq) microtubule-associated protein 70-5-like (A)	Microtubule-associated protein 70-5; AtMAP70-5; 70 kDa microtubule-associated protein 5 (At4g17220)		1.892
Cs2g04590	K10999 cellulose synthase A [EC:2.4.1.12] | (RefSeq) cellulose synthase A catalytic subunit 8 [UDP-forming] (A)	Cellulose synthase A catalytic subunit 8 [UDP-forming]; AtCesA8; EC=2.4.1.12; Protein IRREGULAR XYLEM 1; AtIRX1; Protein LEAF WILTING 2 (At4g18780)	1.721	4.523
Cs2g05490		NAC domain-containing protein 73 {ECO:0000303|PubMed:15029955}; ANAC073 {ECO:0000303|PubMed:15029955}; Protein SECONDARY WALL-ASSOCIATED NAC DOMAIN PROTEIN 2 {ECO:0000303|PubMed:18952777} (At4g28500)	1.261	2.000
Cs2g14920	K08235 xyloglucan:xyloglucosyl transferase [EC:2.4.1.207] | (RefSeq) hypothetical protein (A)	Probable xyloglucan endotransglucosylase/hydrolase protein 5; At-XTH5; XTH-5; EC=2.4.1.207; Flags: Precursor (At5g13870)	-1.016	
Cs2g16190		NAC domain-containing protein 73 {ECO:0000303|PubMed:15029955}; ANAC073 {ECO:0000303|PubMed:15029955}; Protein SECONDARY WALL-ASSOCIATED NAC DOMAIN PROTEIN 2 {ECO:0000303|PubMed:18952777} (At4g28500)	2.055	4.527
Cs2g17920	K08235 xyloglucan:xyloglucosyl transferase [EC:2.4.1.207] | (RefSeq) probable xyloglucan endotransglucosylase/hydrolase protein 28 (A)	Probable xyloglucan endotransglucosylase/hydrolase protein 28; At-XTH28; XTH-28; EC=2.4.1.207; Flags: Precursor (At1g14720)		1.202
Cs2g19510	K15400 omega-hydroxypalmitate O-feruloyl transferase [EC:2.3.1.188] | (RefSeq) omega-hydroxypalmitate O-feruloyl transferase-like (A)	Omega-hydroxypalmitate O-feruloyl transferase; EC=2.3.1.188; Omega-hydroxyacid hydroxycinnamoyltransferase; Protein ALIPHATIC SUBERIN FERULOYL TRANSFERASE (At5g41040)		3.666
Cs2g21100	K10891 fanconi anemia group D2 protein | (RefSeq) Fanconi anemia group D2 protein homolog (A)	Cellulose synthase-like protein B4; AtCslB4; EC=2.4.1.- (At2g32540)		-1.449
Cs2g25390	K18801 glucuronoxylan 4-O-methyltransferase [EC:2.1.1.112] | (RefSeq) glucuronoxylan 4-O-methyltransferase-like protein (DUF579) (A)	Probable methyltransferase At1g27930; EC=2.1.1.- {ECO:0000305} (At1g27930)	-1.027	
Cs2g25720	K15400 omega-hydroxypalmitate O-feruloyl transferase [EC:2.3.1.188] | (RefSeq) omega-hydroxypalmitate O-feruloyl transferase (A)	Omega-hydroxypalmitate O-feruloyl transferase; EC=2.3.1.188; Omega-hydroxyacid hydroxycinnamoyltransferase; Protein ALIPHATIC SUBERIN FERULOYL TRANSFERASE (At5g41040)		2.867
Cs3g03080	K18801 glucuronoxylan 4-O-methyltransferase [EC:2.1.1.112] | (RefSeq) glucuronoxylan 4-O-methyltransferase 1 (A)	Glucuronoxylan 4-O-methyltransferase 1; EC=2.1.1.112 (At1g09610)	1.160	3.145

Cs3g08950	K08235 xyloglucan:xyloglucosyl transferase [EC:2.4.1.207] | (RefSeq) hypothetical protein (A)	Probable xyloglucan endotransglucosylase/hydrolase protein 30; At-XTH30; XTH-30; EC=2.4.1.207; Flags: Precursor (At1g32170)		1.305
Cs3g20590	K09422 transcription factor MYB, plant | (RefSeq) myb-related protein 308-like (A)	Transcription factor MYB52 {ECO:0000303|PubMed:9839469}; Myb-related protein 52 {ECO:0000303|PubMed:9839469}; AtMYB52 {ECO:0000303|PubMed:9839469}; Protein ABA-HYPERSENSITIVE 1 {ECO:0000303|PubMed:21399993} (At1g17950)	1.675	4.918
Cs3g20910	K20890 xylan alpha-glucuronosyltransferase [EC:2.4.1.-] | (RefSeq) putative UDP-glucuronate:xylan alpha-glucuronosyltransferase 3 (A)	Putative UDP-glucuronate:xylan alpha-glucuronosyltransferase 3; UDP-GlcA:xylan glucuronyltransferase 3; EC=2.4.1.-; Glycogenin-like protein 3; Plant glycogenin-like starch initiation protein 2; Protein GLUCURONIC ACID SUBSTITUTION OF XYLAN 3; AtGUX3 (At1g77130)		1.189
Cs3g23600	K20890 xylan alpha-glucuronosyltransferase [EC:2.4.1.-] | (RefSeq) UDP-glucuronate:xylan alpha-glucuronosyltransferase 2 (A)	UDP-glucuronate:xylan alpha-glucuronosyltransferase 2; UDP-GlcA:xylan glucuronyltransferase 2; EC=2.4.1.-; Glycogenin-like protein 2; Plant glycogenin-like starch initiation protein 3; Protein GLUCURONIC ACID SUBSTITUTION OF XYLAN 2; AtGUX2 (At4g33330)	1.620	4.416
Cs4g01990	K10999 cellulose synthase A [EC:2.4.1.12] | (RefSeq) cellulose synthase A catalytic subunit 7 [UDP-forming] (A)	Cellulose synthase A catalytic subunit 7 [UDP-forming]; AtCesA7; EC=2.4.1.12; Protein FRAGILE FIBER 5; Protein IRREGULAR XYLEM 3; AtIRX3 (At5g17420)	1.503	3.892
Cs4g03050	K14504 xyloglucan:xyloglucosyl transferase TCH4 [EC:2.4.1.207] | (RefSeq) probable xyloglucan endotransglucosylase/hydrolase protein 23 (A)	Probable xyloglucan endotransglucosylase/hydrolase protein 23; At-XTH23; XTH-23; EC=2.4.1.207; Flags: Precursor (At4g25810)		1.738
Cs4g03060	K14504 xyloglucan:xyloglucosyl transferase TCH4 [EC:2.4.1.207] | (RefSeq) probable xyloglucan endotransglucosylase/hydrolase protein 23 (A)	Probable xyloglucan endotransglucosylase/hydrolase protein 23; At-XTH23; XTH-23; EC=2.4.1.207; Flags: Precursor (At4g25810)		1.400
Cs4g03070	K14504 xyloglucan:xyloglucosyl transferase TCH4 [EC:2.4.1.207] | (RefSeq) probable xyloglucan endotransglucosylase/hydrolase protein 23 (A)	Probable xyloglucan endotransglucosylase/hydrolase protein 23; At-XTH23; XTH-23; EC=2.4.1.207; Flags: Precursor (At4g25810)		1.428
Cs4g03130	K14504 xyloglucan:xyloglucosyl transferase TCH4 [EC:2.4.1.207] | (RefSeq) hypothetical protein (A)	Probable xyloglucan endotransglucosylase/hydrolase protein 23; At-XTH23; XTH-23; EC=2.4.1.207; Flags: Precursor (At4g25810)	1.252	1.931
Cs4g03140	K14504 xyloglucan:xyloglucosyl transferase TCH4 [EC:2.4.1.207] | (RefSeq) hypothetical protein (A)	Probable xyloglucan endotransglucosylase/hydrolase protein 23; At-XTH23; XTH-23; EC=2.4.1.207; Flags: Precursor (At4g25810)		1.602
Cs4g03200	K14504 xyloglucan:xyloglucosyl transferase TCH4 [EC:2.4.1.207] | (RefSeq) probable xyloglucan endotransglucosylase/hydrolase protein 23 (A)	Probable xyloglucan endotransglucosylase/hydrolase protein 23; At-XTH23; XTH-23; EC=2.4.1.207; Flags: Precursor (At4g25810)		1.214

Cs4g08540	K10999 cellulose synthase A [EC:2.4.1.12] | (RefSeq) cellulose synthase A catalytic subunit 7 [UDP-forming]-like (A)	Cellulose synthase-like protein E1; AtCslE1; EC=2.4.1.- (At1g55850)	-2.353	
Cs4g08560	K20923 mixed-linked glucan synthase [EC:2.4.1.-] | (RefSeq) probable mixed-linked glucan synthase 6 (A)	Cellulose synthase-like protein E1; AtCslE1; EC=2.4.1.- (At1g55850)		1.596
Cs4g12320	K20867 galacturonosyltransferase 12/13/14/15 [EC:2.4.1.-] | (RefSeq) probable galacturonosyltransferase 12 (A)	Probable galacturonosyltransferase 12; EC=2.4.1.-; Like glycosyl transferase 6; Protein IRREGULAR XYLEM 8 (At5g54690)	1.384	2.469
Cs5g01350	K00521 ferric-chelate reductase [EC:1.16.1.7] | (RefSeq) ferric reduction oxidase 7, chloroplastic-like (A)	NAC domain-containing protein 12 {ECO:0000303|PubMed:15029955}; ANAC012 {ECO:0000303|PubMed:15029955}; Protein NAC SECONDARY WALL THICKENING PROMOTING 3 {ECO:0000303|PubMed:17237351}; Protein SECONDARY WALL-ASSOCIATED NAC DOMAIN PROTEIN 1 {ECO:0000303|PubMed:17114348} (At1g32770)	1.426	4.287
Cs5g01970	K10999 cellulose synthase A [EC:2.4.1.12] | (RefSeq) cellulose synthase A catalytic subunit 7 [UDP-forming]-like isoform X1 (A)	Cellulose synthase-like protein G3; AtCslG3; EC=2.4.1.- (At4g23990)	1.617	
Cs5g03130	K09422 transcription factor MYB, plant | (RefSeq) myb-related protein Myb4 (A)	Transcription factor MYB26 {ECO:0000305}; Myb-related protein 26 {ECO:0000305}; AtMYB26 {ECO:0000303|PubMed:11597504}; Protein MALE STERILE 35 {ECO:0000303|PubMed:12753590} (At1g63910)	1.519	2.426
Cs5g12220	K17964 leucine-rich PPR motif-containing protein, mitochondrial | (RefSeq) pentatricopeptide repeat-containing protein At3g18110, chloroplastic-like (A)	COBRA-like protein 4; Flags: Precursor (At5g15630)	1.039	3.198
Cs5g12230	K17964 leucine-rich PPR motif-containing protein, mitochondrial | (RefSeq) pentatricopeptide repeat-containing protein At3g18110, chloroplastic-like (A)	COBRA-like protein 4; Flags: Precursor (At5g15630)		2.418
Cs5g16420	K09422 transcription factor MYB, plant | (RefSeq) hypothetical protein (A)	Transcription factor MYB52 {ECO:0000303|PubMed:9839469}; Myb-related protein 52 {ECO:0000303|PubMed:9839469}; AtMYB52 {ECO:0000303|PubMed:9839469}; Protein ABA-HYPERSENSITIVE 1 {ECO:0000303|PubMed:21399993} (At1g17950)	1.560	3.254
Cs5g18370	K18801 glucuronoxylan 4-O-methyltransferase [EC:2.1.1.112] | (RefSeq) glucuronoxylan 4-O-methyltransferase 3 (A)	Glucuronoxylan 4-O-methyltransferase 3; EC=2.1.1.112 (At1g33800)		2.166

Cs5g18940	K00521 ferric-chelate reductase [EC:1.16.1.7] | (RefSeq) ferric reduction oxidase 7, chloroplastic-like (A)	Protein BEARSKIN2; NAC domain-containing protein 70; ANAC070 (At4g10350)	-1.411	
Cs5g29200	K10999 cellulose synthase A [EC:2.4.1.12] | (RefSeq) cellulose synthase A catalytic subunit 4 [UDP-forming] (A)	Cellulose synthase A catalytic subunit 4 [UDP-forming]; AtCesA4; EC=2.4.1.12; Protein IRREGULAR XYLEM 5; AtIRX5 (At5g17420)	1.736	4.872
Cs6g02570		CRIB domain-containing protein RIC4; ROP-interactive CRIB motif-containing protein 4; Target of ROP protein RIC4 (At5g16490)		1.512
Cs6g04310	K19519 transforming growth factor-beta-induced protein | (RefSeq) fasciclin-like arabinogalactan protein 12 (A)	Fasciclin-like arabinogalactan protein 11; Flags: Precursor (At5g03170)	1.066	2.802
Cs6g04870	K20869 putative beta-1,4-xylosyltransferase IRX9 [EC:2.4.2.-] | (RefSeq) probable beta-1,4-xylosyltransferase IRX9 (A)	Probable beta-1,4-xylosyltransferase IRX9; EC=2.4.2.-; Protein IRREGULAR XYLEM 9; Xylan xylosyltransferase IRX9 (At2g37090)	1.695	3.472
Cs6g07800	K05909 laccase [EC:1.10.3.2] | (RefSeq) hypothetical protein (A)	Laccase-4; EC=1.10.3.2; Benzenediol:oxygen oxidoreductase 4; Diphenol oxidase 4; Protein IRREGULAR XYLEM 12; Urishiol oxidase 4; Flags: Precursor (At2g38080)	1.504	3.815
Cs6g08690	K16573 gamma-tubulin complex component 6 | (RefSeq) uncharacterized LOC103722329 (A)	Protein trichome birefringence-like 3 (At5g01360)	1.007	2.548
Cs6g09180	K16573 gamma-tubulin complex component 6 | (RefSeq) uncharacterized LOC103722329 (A)	Protein trichome birefringence-like 34 (At2g38320)		1.999
Cs6g17170	K16573 gamma-tubulin complex component 6 | (RefSeq) uncharacterized LOC103722329 (A)	Protein trichome birefringence-like 33 (At2g40320)		-1.021
Cs7g01800	K09422 transcription factor MYB, plant | (RefSeq) transcription factor MYB46 (A)	Transcription factor MYB83 {ECO:0000303|PubMed:11597504}; Myb-related protein 83 {ECO:0000303|PubMed:11597504}; AtMYB83 {ECO:0000303|PubMed:11597504} (At3g08500)		1.661
Cs7g30510	K16573 gamma-tubulin complex component 6 | (RefSeq) uncharacterized LOC103722329 (A)	Protein trichome birefringence-like 33 (At2g40320)	1.388	2.970
Cs7g30960	K16573 gamma-tubulin complex component 6 | (RefSeq) uncharacterized LOC103722329 (A)	Protein ESKIMO 1; Protein trichome birefringence-like 29 (At3g55990)		2.314
Cs8g05250	K18801 glucuronoxylan 4-O-methyltransferase [EC:2.1.1.112] | (RefSeq) hypothetical protein (A)	Protein IRX15-LIKE (At5g67210)		-1.292
Cs8g10780	K20870 putative beta-1,4-xylosyltransferase IRX10 [EC:2.4.2.-] | (RefSeq) probable beta-1,4-xylosyltransferase IRX10 (A)	Probable beta-1,4-xylosyltransferase IRX10; EC=2.4.2.-; Glucuronoxylan glucuronosyltransferase 1; AtGUT1; Glucuronoxylan glucuronosyltransferase 2; Protein IRREGULAR XYLEM 10; Xylan xylosyltransferase IRX10 (At1g27440)	1.084	2.225

Cs8g15710	K20889 probable glucuronoxylan glucuronosyltransferase IRX7 [EC2.4.1.-] | (RefSeq) probable glucuronoxylan glucuronosyltransferase IRX7 (A)	Probable glucuronoxylan glucuronosyltransferase IRX7; EC=2.4.1.-; Protein FRAGILE FIBER 8; Protein IRREGULAR XYLEM 7 (At2g28110)		1.396
Cs8g15720	K08235 xyloglucan:xyloglucosyl transferase [EC:2.4.1.207] | (RefSeq) xyloglucan endotransglucosylase/hydrolase protein 31-like (A)	Probable xyloglucan endotransglucosylase/hydrolase protein 32; At-XTH32; XTH-32; EC=2.4.1.207; Flags: Precursor (At2g36870)	1.919	
Cs8g16840	K19519 transforming growth factor-beta-induced protein | (RefSeq) fasciclin-like arabinogalactan protein 12 (A)	Fasciclin-like arabinogalactan protein 12; Flags: Precursor (At5g60490)		2.345
Cs8g19850	K05909 laccase [EC:1.10.3.2] | (RefSeq) hypothetical protein (A)	Laccase-4; EC=1.10.3.2; Benzenediol:oxygen oxidoreductase 4; Diphenol oxidase 4; Protein IRREGULAR XYLEM 12; Urishiol oxidase 4; Flags: Precursor (At2g38080)	1.651	3.446
Cs8g21030	K20642 Rho GTPase-activating protein 22/24/25 | (RefSeq) rho GTPase-activating protein 7-like (A)	Rho GTPase-activating protein 7; Rho-type GTPase-activating protein 7 (At3g13000)		1.296
Cs8g21040	K15400 omega-hydroxypalmitate O-feruloyl transferase [EC:2.3.1.188] | (RefSeq) omega-hydroxypalmitate O-feruloyl transferase-like (A)	Omega-hydroxypalmitate O-feruloyl transferase; EC=2.3.1.188; Omega-hydroxyacid hydroxycinnamoyltransferase; Protein ALIPHATIC SUBERIN FERULOYL TRANSFERASE (At1g78990)	1.742	2.780
Cs9g08730	K10999 cellulose synthase A [EC:2.4.1.12] | (RefSeq) cellulose synthase A catalytic subunit 8 [UDP-forming] (A)	Cellulose synthase-like protein G2; AtCslG2; EC=2.4.1.- (At4g24000)	1.748	1.562
Cs9g08750	K10999 cellulose synthase A [EC:2.4.1.12] | (Kazusa) Lj5g3v1119490.1; - (A)	Cellulose synthase-like protein E1; EC=2.4.1.-; OsCslE1 (At4g24000)	1.204	
Cs9g08760	K10999 cellulose synthase A [EC:2.4.1.12] | (RefSeq) cellulose synthase A catalytic subunit 7 [UDP-forming]-like (A)	Cellulose synthase-like protein E6; EC=2.4.1.-; OsCslE6 (At4g24000)	1.054	1.806
Cs9g14460	K00521 ferric-chelate reductase [EC:1.16.1.7] | (RefSeq) ferric reduction oxidase 7, chloroplastic-like (A)	NAC domain-containing protein 7 {ECO:0000303|PubMed:15029955}; ANAC007 {ECO:0000303|PubMed:15029955}; Protein EMBRYO DEFECTIVE 2749 {ECO:0000303|Ref.7}; Protein VASCULAR RELATED NAC-DOMAIN 4 {ECO:0000303|PubMed:16103214} (At1g12260)		1.588
novel.1550	K13648 alpha-1,4-galacturonosyltransferase [EC:2.4.1.43] | (RefSeq) hypothetical protein (A)	GAUT8_ARATH Galacturonosyltransferase 8; EC=2.4.1.-; Glycosyltransferase QUASIMODO1 (At3g25140)	-1.020	
novel.2352		ELD1_ARATH Glycosyltransferase-like KOBITO 1 {ECO:0000303|PubMed:12215501, ECO:0000303|PubMed:22457425}; EC=2.4.1.- {ECO:0000305|PubMed:22457425}; Protein ABA INSENSITIVE 8 {ECO:0000303|PubMed:14742875}; Protein ELONGATION DEFECTIVE		-1.008

		1 {ECO:0000303|PubMed:10859181}; Flags: Precursor (At3g08550)		
orange1.1t00561	K00521 ferric-chelate reductase [EC:1.16.1.7] | (RefSeq) ferric reduction oxidase 7, chloroplastic-like (A)	NAC domain-containing protein 43; ANAC043; Protein EMBRYO DEFECTIVE 2301; Protein NAC SECONDARY WALL THICKENING PROMOTING FACTOR 1 (At2g46770)		3.429
orange1.1t02619	K15400 omega-hydroxypalmitate O-feruloyl transferase [EC:2.3.1.188] | (RefSeq) hypothetical protein (A)	Omega-hydroxypalmitate O-feruloyl transferase; EC=2.3.1.188; Omega-hydroxyacid hydroxycinnamoyltransferase; Protein ALIPHATIC SUBERIN FERULOYL TRANSFERASE (At5g41040)	2.639	5.667
orange1.1t03392	K20870 putative beta-1,4-xylosyltransferase IRX10 [EC:2.4.2.-] | (RefSeq) uncharacterized protein LOC103936878 (A)	Probable beta-1,4-xylosyltransferase IRX10; EC=2.4.2.-; Glucuronoxylan glucuronosyltransferase 1; AtGUT1; Glucuronoxylan glucuronosyltransferase 2; Protein IRREGULAR XYLEM 10; Xylan xylosyltransferase IRX10 (At3g57630)		1.540
orange1.1t04064	K18801 glucuronoxylan 4-O-methyltransferase [EC:2.1.1.112] | (RefSeq) hypothetical protein (A)	Protein IRX15-LIKE (At5g67210)	1.609	3.639
Cell wall modification [GO:0042545; P = 0.1722 (P3CR vs. P3R) and 0.0475 (P5CR vs. P5R)]				
Cs1g02580	K13422 transcription factor MYC2 | (RefSeq) transcription factor MYC2-like (A)	Transcription factor bHLH91; Basic helix-loop-helix protein 91; AtbHLH91; bHLH 91; Transcription factor EN 25; bHLH transcription factor bHLH091 (At2g31220)		1.917
Cs1g15710	K05681 ATP-binding cassette, subfamily G (WHITE), member 2 | (RefSeq) hypothetical protein (A)	ABC transporter G family member 36; ABC transporter ABCG.36; AtABCG36; Pleiotropic drug resistance protein 8; Protein PENETRATION 3 (At1g59870)		-2.119
Cs1g16560	K01051 pectinesterase [EC:3.1.1.11] | (RefSeq) pectinesterase 3 (A)	Pectinesterase 3; PE 3; EC=3.1.1.11; Pectin methylesterase 3; Flags: Precursor (At1g53840)		1.217
Cs2g16380	K01051 pectinesterase [EC:3.1.1.11] | (RefSeq) putative pectinesterase 11 (A)	Putative pectinesterase 11; PE 11; EC=3.1.1.11; Pectin methylesterase 11; AtPME11 (At2g21610)		1.477
Cs2g17090	K01179 endoglucanase [EC:3.2.1.4] | (RefSeq) endoglucanase 8-like (A)	Endoglucanase 8; EC=3.2.1.4; Cellulase 1; AtCEL1; Endo-1,4-beta glucanase 8; Flags: Precursor (At1g70710)	-1.018	
Cs3g21550	K20617 cytochrome P450 family 71 subfamily A | (RefSeq) hypothetical protein (A)	Cytochrome P450 83B1; EC=1.14.14.45 {ECO:0000269|PubMed:12970475}; Protein ALTERED TRYPTOPHAN REGULATION 4; Protein RED ELONGATED 1; Protein SUPERROOT 2 (At4g31500)		2.005
Cs3g25780	K20617 cytochrome P450 family 71 subfamily A | (RefSeq) cytochrome P450 71A1-like (A)	Cytochrome P450 83B1; EC=1.14.14.45 {ECO:0000269|PubMed:12970475}; Protein ALTERED TRYPTOPHAN REGULATION 4; Protein RED ELONGATED 1; Protein SUPERROOT 2	3.636	

		(At4g31500)		
Cs4g06630	K01051 pectinesterase [EC:3.1.1.11] | (RefSeq) PME4; putative thermostable pectinesterase (A)	Pectinesterase 3; PE 3; EC=3.1.1.11; Pectin methylesterase 3; Flags: Precursor (At3g14310)		
Cs4g06670	K01051 pectinesterase [EC:3.1.1.11] | (RefSeq) probable pectinesterase/pectinesterase inhibitor 12 (A)	Probable pectinesterase/pectinesterase inhibitor 12; Includes: Pectinesterase inhibitor 12; Pectin methylesterase inhibitor 12; Includes: Pectinesterase 12; PE 12; EC=3.1.1.11; Pectin methylesterase 12; AtPME12; Flags: Precursor (At2g26440)		-1.091
Cs5g23130	K13420 LRR receptor-like serine/threonine-protein kinase FLS2 [EC:2.7.11.1] | (RefSeq) LRR receptor-like serine/threonine-protein kinase FLS2 (A)	LRR receptor-like serine/threonine-protein kinase FLS2; EC=2.7.11.1; Protein FLAGELLIN-SENSING 2; Protein FLAGELLIN-SENSITIVE 2; Flags: Precursor (At5g46330)		-1.088
Cs5g25060	K20617 cytochrome P450 family 71 subfamily A | (RefSeq) cytochrome P450 71A1-like (A)	Cytochrome P450 83B1; EC=1.14.14.45 {ECO:0000269|PubMed:12970475}; Protein ALTERED TRYPTOPHAN REGULATION 4; Protein RED ELONGATED 1; Protein SUPERROOT 2 (At4g31500)		-1.027
Cs5g25070	K20617 cytochrome P450 family 71 subfamily A | (RefSeq) cytochrome P450 71A1-like (A)	Cytochrome P450 83B1; EC=1.14.14.45 {ECO:0000269|PubMed:12970475}; Protein ALTERED TRYPTOPHAN REGULATION 4; Protein RED ELONGATED 1; Protein SUPERROOT 2 (At4g31500)		2.331
Cs5g28040	K14963 COMPASS component SWD3 | (RefSeq) transcriptional corepressor LEUNIG_HOMOLOG-like (A)	Transcriptional corepressor LEUNIG_HOMOLOG; Protein MUCILAGE-MODIFIED 1 {ECO:0000303|PubMed:11706181} (At2g32700)		1.712
Cs5g33410	K01051 pectinesterase [EC:3.1.1.11] | (RefSeq) probable pectinesterase/pectinesterase inhibitor 20 (A)	Probable pectinesterase/pectinesterase inhibitor 41; Includes: Pectinesterase inhibitor 41; Pectin methylesterase inhibitor 41; Includes: Pectinesterase 41; PE 41; EC=3.1.1.11; AtPMEpcrB; Pectin methylesterase 41; AtPME41; Flags: Precursor (At4g02330)	-1.001	
Cs6g08690	K16573 gamma-tubulin complex component 6 | (RefSeq) uncharacterized LOC103722329 (A)	Protein trichome birefringence-like 3 (At5g01360)	1.007	2.548
Cs6g11440	K01051 pectinesterase [EC:3.1.1.11] | (RefSeq) probable pectinesterase 8 (A)	Probable pectinesterase 8; PE 8; EC=3.1.1.11; Pectin methylesterase 2; AtPME2; Pectin methylesterase 8; AtPME8; Flags: Precursor (At1g05310)		5.569
Cs6g17580	K14190 GDP-L-galactose phosphorylase [EC:2.7.7.69] | (RefSeq) hypothetical protein (A)	GDP-L-galactose phosphorylase 1; EC=2.7.7.69; Protein VITAMIN C DEFECTIVE 2 (At5g55120)	1.157	2.069
Cs7g08110	K11820 N-hydroxythioamide S-beta-glucosyltransferase [EC:2.4.1.195] | (RefSeq) UDP-glycosyltransferase 74B1 (A)	UDP-glycosyltransferase 74B1; EC=2.4.1.-; N-hydroxythioamide S-beta-glucosyltransferase; EC=2.4.1.195; Thiohydroximate S-glucosyltransferase (At1g24100)	-1.156	-2.258

Cs7g09030	K01213 galacturan 1,4-alpha-galacturonidase [EC:3.2.1.67] | (RefSeq) polygalacturonase At1g48100 (A)	Polygalacturonase At1g48100; PG; EC=3.2.1.15; Pectinase At1g48100; Flags: Precursor (At3g26610)	1.069	1.504
Cs7g21940	K01728 pectate lyase [EC:4.2.2.2] | (RefSeq) hypothetical protein (A)	Probable pectate lyase 13; EC=4.2.2.2; Powdery mildew susceptibility protein; Powdery mildew-resistant mutant 6; Flags: Precursor (At5g04310)		
Cs9g01630	K01213 galacturan 1,4-alpha-galacturonidase [EC:3.2.1.67] | (RefSeq) polygalacturonase At1g48100-like (A)	Polygalacturonase At1g48100; PG; EC=3.2.1.15; Pectinase At1g48100; Flags: Precursor (At1g48100)		1.625
Cs9g14450	K01051 pectinesterase [EC:3.1.1.11] | (RefSeq) pectinesterase 2-like (A)	Pectinesterase 2; PE 2; EC=3.1.1.11; Pectin methylesterase; Flags: Precursor (At2g45220)	-1.167	
novel.2352		ELD1_ARATH Glycosyltransferase-like KOBITO 1 {ECO:0000303|PubMed:12215501, ECO:0000303|PubMed:22457425}; EC=2.4.1.- {ECO:0000305|PubMed:22457425}; Protein ABA INSENSITIVE 8 {ECO:0000303|PubMed:14742875}; Protein ELONGATION DEFECTIVE 1 {ECO:0000303|PubMed:10859181}; Flags: Precursor (At3g08550)		-1.008
orange1.1t00214	K01051 pectinesterase [EC:3.1.1.11] | (RefSeq) hypothetical protein (A)	Pectinesterase 2; PE 2; EC=3.1.1.11; Pectin methylesterase; Flags: Precursor (At2g45220)	1.028	
orange1.1t00615	K20628 expansin | (RefSeq) expansin-A4-like (A)	Putative expansin-A17; AtEXPA17; Alpha-expansin-17; At-EXP17; AtEx17; Ath-ExpAlpha-1.13; Flags: Precursor (At4g01630)	-2.165	-1.155
orange1.1t00806	K11000 callose synthase [EC:2.4.1.-] | (RefSeq) callose synthase 12 (A)	Callose synthase 12; EC=2.4.1.34; 1,3-beta-glucan synthase; Protein GLUCAN SYNTHASE-LIKE 5; Protein POWDERY MILDEW RESISTANT 4 (At4g03550)		1.145
orange1.1t01085	K01051 pectinesterase [EC:3.1.1.11] | (RefSeq) hypothetical protein (A)	Probable pectinesterase 15; PE 15; EC=3.1.1.11; Pectin methylesterase 15; AtPME15; Flags: Precursor (At2g36710)		2.963
orange1.1t01727	K01051 pectinesterase [EC:3.1.1.11] | (RefSeq) probable pectinesterase 53 (A)	Probable pectinesterase 53; PE 53; EC=3.1.1.11; Pectin methylesterase 53; AtPME53; Flags: Precursor (At5g19730)	-1.455	
orange1.1t01738	K01213 galacturan 1,4-alpha-galacturonidase [EC:3.2.1.67] | (RefSeq) hypothetical protein (A)	Polygalacturonase At1g48100; PG; EC=3.2.1.15; Pectinase At1g48100; Flags: Precursor (At1g02460)	1.103	1.253
orange1.1t02087	K20617 cytochrome P450 family 71 subfamily A | (RefSeq) cytochrome P450 71A1-like (A)	Cytochrome P450 83B1; EC=1.14.14.45 {ECO:0000269|PubMed:12970475}; Protein ALTERED TRYPTOPHAN REGULATION 4; Protein RED ELONGATED 1; Protein SUPERROOT 2 (At4g31500)		-2.078
orange1.1t02796	K20617 cytochrome P450 family 71 subfamily A | (RefSeq) cytochrome P450 71A1-like (A)	Cytochrome P450 83B1; EC=1.14.14.45 {ECO:0000269|PubMed:12970475}; Protein ALTERED TRYPTOPHAN	2.393	1.485

		REGULATION 4; Protein RED ELONGATED 1; Protein SUPERROOT 2 (At4g31500)		
Cell wall thickening [GO:0052386; P = 0.3451 (P3CR vs. P3R) and 0.0073 (P5CR vs. P5R)]				
Cs1g02580	K13422 transcription factor MYC2 | (RefSeq) transcription factor MYC2-like (A)	Transcription factor bHLH91; Basic helix-loop-helix protein 91; AtbHLH91; bHLH 91; Transcription factor EN 25; bHLH transcription factor bHLH091 (At2g31220)		1.917
Cs1g15710	K05681 ATP-binding cassette, subfamily G (WHITE), member 2 | (RefSeq) hypothetical protein (A)	ABC transporter G family member 36; ABC transporter ABCG.36; AtABCG36; Pleiotropic drug resistance protein 8; Protein PENETRATION 3 (At1g59870)		-2.119
Cs3g21550	K20617 cytochrome P450 family 71 subfamily A | (RefSeq) hypothetical protein (A)	Cytochrome P450 83B1; EC=1.14.14.45 {ECO:0000269|PubMed:12970475}; Protein ALTERED TRYPTOPHAN REGULATION 4; Protein RED ELONGATED 1; Protein SUPERROOT 2 (At4g31500)		2.005
Cs3g25780	K20617 cytochrome P450 family 71 subfamily A | (RefSeq) cytochrome P450 71A1-like (A)	Cytochrome P450 83B1; EC=1.14.14.45 {ECO:0000269|PubMed:12970475}; Protein ALTERED TRYPTOPHAN REGULATION 4; Protein RED ELONGATED 1; Protein SUPERROOT 2 (At4g31500)	3.636	
Cs5g23130	K13420 LRR receptor-like serine/threonine-protein kinase FLS2 [EC:2.7.11.1] | (RefSeq) LRR receptor-like serine/threonine-protein kinase FLS2 (A)	LRR receptor-like serine/threonine-protein kinase FLS2; EC=2.7.11.1; Protein FLAGELLIN-SENSING 2; Protein FLAGELLIN-SENSITIVE 2; Flags: Precursor (At5g46330)		-1.088
Cs5g25060	K20617 cytochrome P450 family 71 subfamily A | (RefSeq) cytochrome P450 71A1-like (A)	Cytochrome P450 83B1; EC=1.14.14.45 {ECO:0000269|PubMed:12970475}; Protein ALTERED TRYPTOPHAN REGULATION 4; Protein RED ELONGATED 1; Protein SUPERROOT 2 (At4g31500)		-1.027
Cs5g25070	K20617 cytochrome P450 family 71 subfamily A | (RefSeq) cytochrome P450 71A1-like (A)	Cytochrome P450 83B1; EC=1.14.14.45 {ECO:0000269|PubMed:12970475}; Protein ALTERED TRYPTOPHAN REGULATION 4; Protein RED ELONGATED 1; Protein SUPERROOT 2 (At4g31500)		2.331
Cs6g17580	K14190 GDP-L-galactose phosphorylase [EC:2.7.7.69] | (RefSeq) hypothetical protein (A)	GDP-L-galactose phosphorylase 1; EC=2.7.7.69; Protein VITAMIN C DEFECTIVE 2 (At5g55120)	1.157	2.069
Cs7g08110	K11820 N-hydroxythioamide S-beta-glucosyltransferase [EC:2.4.1.195] | (RefSeq) UDP-glycosyltransferase 74B1 (A)	UDP-glycosyltransferase 74B1; EC=2.4.1.-; N-hydroxythioamide S-beta-glucosyltransferase; EC=2.4.1.195; Thiohydroximate S-glucosyltransferase (At1g24100)	-1.156	-2.258

orange1.1t00806	K11000 callose synthase [EC:2.4.1.-] | (RefSeq) callose synthase 12 (A)	Callose synthase 12; EC=2.4.1.34; 1,3-beta-glucan synthase; Protein GLUCAN SYNTHASE-LIKE 5; Protein POWDERY MILDEW RESISTANT 4 (At4g03550)		1.145
orange1.1t02087	K20617 cytochrome P450 family 71 subfamily A | (RefSeq) cytochrome P450 71A1-like (A)	Cytochrome P450 83B1; EC=1.14.14.45 {ECO:0000269|PubMed:12970475}; Protein ALTERED TRYPTOPHAN REGULATION 4; Protein RED ELONGATED 1; Protein SUPERROOT 2 (At4g31500)		-2.078
orange1.1t02796	K20617 cytochrome P450 family 71 subfamily A | (RefSeq) cytochrome P450 71A1-like (A)	Cytochrome P450 83B1; EC=1.14.14.45 {ECO:0000269|PubMed:12970475}; Protein ALTERED TRYPTOPHAN REGULATION 4; Protein RED ELONGATED 1; Protein SUPERROOT 2 (At4g31500)	2.393	1.485
Cell wall macromolecule biosynthetic process [GO:0044038; P = 0.0011 (P3CR vs. P3R) and < 0.0001 (P5CR vs. P5R)]				
Cs1g05500	K20890 xylan alpha-glucuronosyltransferase [EC:2.4.1.-] | (RefSeq) UDP-glucuronate:xylan alpha-glucuronosyltransferase 1 (A)	UDP-glucuronate:xylan alpha-glucuronosyltransferase 1; UDP-GlcA:xylan glucuronyltransferase 1; EC=2.4.1.-; Glycogenin-like protein 1; Plant glycogenin-like starch initiation protein 1; Protein GLUCURONIC ACID SUBSTITUTION OF XYLAN 1; AtGUX1 (At3g18660)	1.889	3.881
Cs2g19510	K15400 omega-hydroxypalmitate O-feruloyl transferase [EC:2.3.1.188] | (RefSeq) omega-hydroxypalmitate O-feruloyl transferase-like (A)	Omega-hydroxypalmitate O-feruloyl transferase; EC=2.3.1.188; Omega-hydroxyacid hydroxycinnamoyltransferase; Protein ALIPHATIC SUBERIN FERULOYL TRANSFERASE (At5g41040)		3.666
Cs2g25390	K18801 glucuronoxylan 4-O-methyltransferase [EC:2.1.1.112] | (RefSeq) glucuronoxylan 4-O-methyltransferase-like protein (DUF579) (A)	Probable methyltransferase At1g27930; EC=2.1.1.- {ECO:0000305} (At1g27930)	-1.027	
Cs2g25720	K15400 omega-hydroxypalmitate O-feruloyl transferase [EC:2.3.1.188] | (RefSeq) omega-hydroxypalmitate O-feruloyl transferase (A)	Omega-hydroxypalmitate O-feruloyl transferase; EC=2.3.1.188; Omega-hydroxyacid hydroxycinnamoyltransferase; Protein ALIPHATIC SUBERIN FERULOYL TRANSFERASE (At5g41040)		2.867
Cs3g03080	K18801 glucuronoxylan 4-O-methyltransferase [EC:2.1.1.112] | (RefSeq) glucuronoxylan 4-O-methyltransferase 1 (A)	Glucuronoxylan 4-O-methyltransferase 1; EC=2.1.1.112 (At1g09610)	1.160	3.145
Cs3g20910	K20890 xylan alpha-glucuronosyltransferase [EC:2.4.1.-] | (RefSeq) putative UDP-glucuronate:xylan alpha-glucuronosyltransferase 3 (A)	Putative UDP-glucuronate:xylan alpha-glucuronosyltransferase 3; UDP-GlcA:xylan glucuronyltransferase 3; EC=2.4.1.-; Glycogenin-like protein 3; Plant glycogenin-like starch initiation protein 2; Protein GLUCURONIC ACID SUBSTITUTION OF XYLAN 3; AtGUX3 (At1g77130)		1.189

Cs3g23600	K20890 xylan alpha-glucuronosyltransferase [EC:2.4.1.-] | (RefSeq) UDP-glucuronate:xylan alpha-glucuronosyltransferase 2 (A)	UDP-glucuronate:xylan alpha-glucuronosyltransferase 2; UDP-GlcA:xylan glucuronyltransferase 2; EC=2.4.1.-; Glycogenin-like protein 2; Plant glycogenin-like starch initiation protein 3; Protein GLUCURONIC ACID SUBSTITUTION OF XYLAN 2; AtGUX2 (At4g33330)	1.620	4.416
Cs4g12320	K20867 galacturonosyltransferase 12/13/14/15 [EC:2.4.1.-] | (RefSeq) probable galacturonosyltransferase 12 (A)	Probable galacturonosyltransferase 12; EC=2.4.1.-; Like glycosyl transferase 6; Protein IRREGULAR XYLEM 8 (At5g54690)	1.384	2.469
Cs5g18370	K18801 glucuronoxylan 4-O-methyltransferase [EC:2.1.1.112] | (RefSeq) glucuronoxylan 4-O-methyltransferase 3 (A)	Glucuronoxylan 4-O-methyltransferase 3; EC=2.1.1.112 (At1g33800)		2.166
Cs6g04870	K20869 putative beta-1,4-xylosyltransferase IRX9 [EC:2.4.2.-] | (RefSeq) probable beta-1,4-xylosyltransferase IRX9 (A)	Probable beta-1,4-xylosyltransferase IRX9; EC=2.4.2.-; Protein IRREGULAR XYLEM 9; Xylan xylosyltransferase IRX9 (At2g37090)	1.695	3.472
Cs6g08690	K16573 gamma-tubulin complex component 6 | (RefSeq) uncharacterized LOC103722329 (A)	Protein trichome birefringence-like 3 (At5g01360)	1.007	2.548
Cs6g09180	K16573 gamma-tubulin complex component 6 | (RefSeq) uncharacterized LOC103722329 (A)	Protein trichome birefringence-like 34 (At2g38320)		1.999
Cs7g30960	K16573 gamma-tubulin complex component 6 | (RefSeq) uncharacterized LOC103722329 (A)	Protein ESKIMO 1; Protein trichome birefringence-like 29 (At3g55990)		2.314
Cs8g05250	K18801 glucuronoxylan 4-O-methyltransferase [EC:2.1.1.112] | (RefSeq) hypothetical protein (A)	Protein IRX15-LIKE (At5g67210)		-1.292
Cs8g10780	K20870 putative beta-1,4-xylosyltransferase IRX10 [EC:2.4.2.-] | (RefSeq) probable beta-1,4-xylosyltransferase IRX10 (A)	Probable beta-1,4-xylosyltransferase IRX10; EC=2.4.2.-; Glucuronoxylan glucuronosyltransferase 1; AtGUT1; Glucuronoxylan glucuronosyltransferase 2; Protein IRREGULAR XYLEM 10; Xylan xylosyltransferase IRX10 (At1g27440)	1.084	2.225
Cs8g15710	K20889 probable glucuronoxylan glucuronosyltransferase IRX7 [EC2.4.1.-] | (RefSeq) probable glucuronoxylan glucuronosyltransferase IRX7 (A)	Probable glucuronoxylan glucuronosyltransferase IRX7; EC=2.4.1.-; Protein FRAGILE FIBER 8; Protein IRREGULAR XYLEM 7 (At2g28110)		1.396
Cs8g21040	K15400 omega-hydroxypalmitate O-feruloyl transferase [EC:2.3.1.188] | (RefSeq) omega-hydroxypalmitate O-feruloyl transferase-like (A)	Omega-hydroxypalmitate O-feruloyl transferase; EC=2.3.1.188; Omega-hydroxyacid hydroxycinnamoyltransferase; Protein ALIPHATIC SUBERIN FERULOYL TRANSFERASE (At1g78990)	1.742	2.780
novel.1550	K13648 alpha-1,4-galacturonosyltransferase [EC:2.4.1.43] | (RefSeq) hypothetical protein (A)	GAUT8_ARATH Galacturonosyltransferase 8; EC=2.4.1.-; Glycosyltransferase QUASIMODO1 (At3g25140)	-1.020	
novel.2352		ELD1_ARATH Glycosyltransferase-like KOBITO 1		-1.008

		{ECO:0000303|PubMed:12215501, ECO:0000303|PubMed:22457425}; EC=2.4.1.- {ECO:0000305|PubMed:22457425}; Protein ABA INSENSITIVE 8 {ECO:0000303|PubMed:14742875}; Protein ELONGATION DEFECTIVE 1 {ECO:0000303|PubMed:10859181}; Flags: Precursor (At3g08550)		
orange1.1t02619	K15400 omega-hydroxypalmitate O-feruloyl transferase [EC:2.3.1.188] | (RefSeq) hypothetical protein (A)	Omega-hydroxypalmitate O-feruloyl transferase; EC=2.3.1.188; Omega-hydroxyacid hydroxycinnamoyltransferase; Protein ALIPHATIC SUBERIN FERULOYL TRANSFERASE (At5g41040)	2.639	5.667
orange1.1t03392	K20870 putative beta-1,4-xylosyltransferase IRX10 [EC:2.4.2.-] | (RefSeq) uncharacterized protein LOC103936878 (A)	Probable beta-1,4-xylosyltransferase IRX10; EC=2.4.2.-; Glucuronoxylan glucuronosyltransferase 1; AtGUT1; Glucuronoxylan glucuronosyltransferase 2; Protein IRREGULAR XYLEM 10; Xylan xylosyltransferase IRX10 (At3g57630)		1.540
orange1.1t04064	K18801 glucuronoxylan 4-O-methyltransferase [EC:2.1.1.112] | (RefSeq) hypothetical protein (A)	Protein IRX15-LIKE (At5g67210)	1.609	3.639
Cellulose biosynthetic process [GO:0030244; P = 0.0367 (P3CR vs. P3R) and 0.0154 (P5CR vs. P5R)]				
Cs2g04590	K10999 cellulose synthase A [EC:2.4.1.12] | (RefSeq) cellulose synthase A catalytic subunit 8 [UDP-forming] (A)	Cellulose synthase A catalytic subunit 8 [UDP-forming]; AtCesA8; EC=2.4.1.12; Protein IRREGULAR XYLEM 1; AtIRX1; Protein LEAF WILTING 2 (At4g18780)	1.721	4.523
Cs2g15690	K16573 gamma-tubulin complex component 6 | (RefSeq) uncharacterized LOC103722329 (A)	Protein trichome birefringence-like 1 (At3g12060)		1.563
Cs2g21100	K10891 fanconi anemia group D2 protein | (RefSeq) Fanconi anemia group D2 protein homolog (A)	Cellulose synthase-like protein B4; AtCslB4; EC=2.4.1.- (At2g32540)		-1.449
Cs3g09000	K20924 cellulose synthase-like protein [EC:2.4.1.-] | (RefSeq) hypothetical protein (A)	Cellulose synthase-like protein D3; AtCslD3; EC=2.4.1.-; Protein KOJAK (At3g03050)		1.847
Cs3g09730	K01187 alpha-glucosidase [EC:3.2.1.20] | (RAP-DB) Os07g0421300; Similar to Alpha glucosidase-like protein. (A)	Probable glucan 1,3-alpha-glucosidase; EC=3.2.1.84; Glucosidase II subunit alpha; Protein PRIORITY IN SWEET LIFE 5; Protein RADIAL SWELLING 3; Flags: Precursor (At3g23640)		1.120
Cs4g01990	K10999 cellulose synthase A [EC:2.4.1.12] | (RefSeq) cellulose synthase A catalytic subunit 7 [UDP-forming] (A)	Cellulose synthase A catalytic subunit 7 [UDP-forming]; AtCesA7; EC=2.4.1.12; Protein FRAGILE FIBER 5; Protein IRREGULAR XYLEM 3; AtIRX3 (At5g17420)	1.503	3.892
Cs4g08540	K10999 cellulose synthase A [EC:2.4.1.12] | (RefSeq) cellulose synthase A catalytic subunit 7 [UDP-forming]-like (A)	Cellulose synthase-like protein E1; AtCslE1; EC=2.4.1.- (At1g55850)	-2.353	
Cs4g08560	K20923 mixed-linked glucan synthase [EC:2.4.1.-] |	Cellulose synthase-like protein E1; AtCslE1; EC=2.4.1.- (At1g55850)		1.596

	(RefSeq) probable mixed-linked glucan synthase 6 (A)			
Cs5g01970	K10999 cellulose synthase A [EC:2.4.1.12] | (RefSeq) cellulose synthase A catalytic subunit 7 [UDP-forming]-like isoform X1 (A)	Cellulose synthase-like protein G3; AtCslG3; EC=2.4.1.- (At4g23990)	1.617	
Cs5g25090	K20924 cellulose synthase-like protein [EC:2.4.1.-] | (RefSeq) hypothetical protein (A)	Cellulose synthase-like protein D3; AtCslD3; EC=2.4.1.-; Protein KOJAK (At3g03050)		2.184
Cs5g29200	K10999 cellulose synthase A [EC:2.4.1.12] | (RefSeq) cellulose synthase A catalytic subunit 4 [UDP-forming] (A)	Cellulose synthase A catalytic subunit 4 [UDP-forming]; AtCesA4; EC=2.4.1.12; Protein IRREGULAR XYLEM 5; AtIRX5 (At5g17420)	1.736	4.872
Cs6g08690	K16573 gamma-tubulin complex component 6 | (RefSeq) uncharacterized LOC103722329 (A)	Protein trichome birefringence-like 3 (At5g01360)	1.007	2.548
Cs9g08730	K10999 cellulose synthase A [EC:2.4.1.12] | (RefSeq) cellulose synthase A catalytic subunit 8 [UDP-forming] (A)	Cellulose synthase-like protein G2; AtCslG2; EC=2.4.1.- (At4g24000)	1.748	1.562
Cs9g08750	K10999 cellulose synthase A [EC:2.4.1.12] | (Kazusa) Lj5g3v1119490.1; - (A)	Cellulose synthase-like protein E1; EC=2.4.1.-; OsCslE1 (At4g24000)	1.204	
Cs9g08760	K10999 cellulose synthase A [EC:2.4.1.12] | (RefSeq) cellulose synthase A catalytic subunit 7 [UDP-forming]-like (A)	Cellulose synthase-like protein E6; EC=2.4.1.-; OsCslE6 (At4g24000)	1.054	1.806
novel.2352		ELD1_ARATH Glycosyltransferase-like KOBITO 1 {ECO:0000303|PubMed:12215501, ECO:0000303|PubMed:22457425}; EC=2.4.1.- {ECO:0000305|PubMed:22457425}; Protein ABA INSENSITIVE 8 {ECO:0000303|PubMed:14742875}; Protein ELONGATION DEFECTIVE 1 {ECO:0000303|PubMed:10859181}; Flags: Precursor (At3g08550)		-1.008
orange1.1t01105	K01187 alpha-glucosidase [EC:3.2.1.20] | (RefSeq) alpha-glucosidase 2-like (A)	Probable glucan 1,3-alpha-glucosidase; EC=3.2.1.84; Glucosidase II subunit alpha; Protein PRIORITY IN SWEET LIFE 5; Protein RADIAL SWELLING 3; Flags: Precursor (At3g23640)		1.095
Lignin biosynthetic process [GO:0009809; P = 0.0001 (P3CR vs. P3R) and 0.0003 (P5CR vs. P5R)]				
Cs1g03780	K13066 caffeic acid 3-O-methyltransferase [EC:2.1.1.68] | (RefSeq) caffeic acid 3-O-methyltransferase-like isoform X1 (A)	Caffeic acid 3-O-methyltransferase; CAOMT; COMT; EC=2.1.1.68; S-adenosysl-L-methionine:caffeic acid 3-O-methyltransferase (At5g54160)		1.270
Cs1g04910	K00083 cinnamyl-alcohol dehydrogenase [EC:1.1.1.195] | (RefSeq) hypothetical protein (A)	Probable cinnamyl alcohol dehydrogenase 9; AtCAD9; EC=1.1.1.195 (At4g39330)	-2.173	

Cs2g09210	K00430 peroxidase [EC:1.11.1.7] | (RefSeq) peroxidase P7-like (A)	Peroxidase 52; Atperox P52; EC=1.11.1.7; ATP49; Flags: Precursor (At5g05340)		1.669
Cs2g30460	K09753 cinnamoyl-CoA reductase [EC:1.2.1.44] | (RefSeq) cinnamoyl-CoA reductase 1-like isoform X1 (A)	Cinnamoyl-CoA reductase 1; AtCCR1; EC=1.2.1.44; Protein IRREGULAR XYLEM 4 (At5g19440)	2.798	
Cs2g30470	K09753 cinnamoyl-CoA reductase [EC:1.2.1.44] | (RefSeq) cinnamoyl-CoA reductase 2-like (A)	Cinnamoyl-CoA reductase 1; AtCCR1; EC=1.2.1.44; Protein IRREGULAR XYLEM 4 (At1g51410)		-1.535
Cs3g20650	K13066 caffeic acid 3-O-methyltransferase [EC:2.1.1.68] | (RefSeq) caffeic acid 3-O-methyltransferase (A)	Caffeic acid 3-O-methyltransferase; CAOMT; COMT; EC=2.1.1.68; S-adenosysl-L-methionine:caffeic acid 3-O-methyltransferase (At5g54160)	3.127	
Cs4g18140	K03006 DNA-directed RNA polymerase II subunit RPB1 [EC:2.7.7.6] | (RefSeq) early nodulin-like protein 2 (A)	Blue copper protein; Blue copper-binding protein; AtBCB; Phytocyanin 1; Stellacyanin; Flags: Precursor (At5g20230)		-1.225
Cs5g13580	K13066 caffeic acid 3-O-methyltransferase [EC:2.1.1.68] | (RefSeq) caffeic acid 3-O-methyltransferase-like (A)	Caffeic acid 3-O-methyltransferase; CAOMT; COMT; EC=2.1.1.68; S-adenosysl-L-methionine:caffeic acid 3-O-methyltransferase (At5g54160)	3.547	
Cs5g16290	K13066 caffeic acid 3-O-methyltransferase [EC:2.1.1.68] | (RefSeq) caffeic acid 3-O-methyltransferase (A)	Caffeic acid 3-O-methyltransferase; CAOMT; COMT; EC=2.1.1.68; S-adenosysl-L-methionine:caffeic acid 3-O-methyltransferase (At5g54160)		1.277
Cs5g18050	K13066 caffeic acid 3-O-methyltransferase [EC:2.1.1.68] | (RefSeq) caffeic acid 3-O-methyltransferase-like isoform X1 (A)	Caffeic acid 3-O-methyltransferase; CAOMT; COMT; EC=2.1.1.68; S-adenosysl-L-methionine:caffeic acid 3-O-methyltransferase (At5g54160)	2.004	
Cs5g19020	K13066 caffeic acid 3-O-methyltransferase [EC:2.1.1.68] | (RefSeq) hypothetical protein (A)	Caffeic acid 3-O-methyltransferase; CAOMT; COMT; EC=2.1.1.68; S-adenosysl-L-methionine:caffeic acid 3-O-methyltransferase (At5g54160)	1.778	1.136
Cs5g25000	K13066 caffeic acid 3-O-methyltransferase [EC:2.1.1.68] | (RefSeq) caffeic acid 3-O-methyltransferase-like (A)	Caffeic acid 3-O-methyltransferase; CAOMT; COMT; EC=2.1.1.68; S-adenosysl-L-methionine:caffeic acid 3-O-methyltransferase (At5g54160)		1.383
Cs6g06880	K05909 laccase [EC:1.10.3.2] | (RefSeq) hypothetical protein (A)	Laccase-17; EC=1.10.3.2; Benzenediol:oxygen oxidoreductase 17; Diphenol oxidase 17; Urishiol oxidase 17; Flags: Precursor (At5g60020)		4.438
Cs6g06920	K05909 laccase [EC:1.10.3.2] | (RefSeq) laccase-17-like (A)	Laccase-17; EC=1.10.3.2; Benzenediol:oxygen oxidoreductase 17; Diphenol oxidase 17; Urishiol oxidase 17; Flags: Precursor (At5g60020)	1.652	3.497
Cs6g07800	K05909 laccase [EC:1.10.3.2] | (RefSeq) hypothetical protein (A)	Laccase-4; EC=1.10.3.2; Benzenediol:oxygen oxidoreductase 4; Diphenol oxidase 4; Protein IRREGULAR XYLEM 12; Urishiol oxidase 4; Flags: Precursor (At2g38080)	1.504	3.815
Cs6g16650	K09754 coumaroylquinate(coumaroylshikimate) 3'-monooxygenase [EC:1.14.13.36] | (RefSeq) cytochrome P450 98A2 (A)	Cytochrome P450 98A2; EC=1.14.-.- (At2g40890)	1.097	1.588
Cs7g23490	K05909 laccase [EC:1.10.3.2] | (RefSeq) hypothetical protein (A)	Laccase-17; EC=1.10.3.2; Benzenediol:oxygen oxidoreductase 17; Diphenol oxidase 17; Urishiol oxidase 17; Flags: Precursor (At5g60020)		3.109

Cs7g25010	K13066 caffeic acid 3-O-methyltransferase [EC:2.1.1.68] | (RefSeq) COMT1-2; caffeic acid O-methyltransferase (A)	Caffeic acid 3-O-methyltransferase; CAOMT; COMT; EC=2.1.1.68; S-adenosysl-L-methionine:caffeic acid 3-O-methyltransferase (At3g53140)		-1.955
Cs7g25540	K13066 caffeic acid 3-O-methyltransferase [EC:2.1.1.68] | (RefSeq) COMT1-2; caffeic acid O-methyltransferase (A)	Caffeic acid 3-O-methyltransferase; CAOMT; COMT; EC=2.1.1.68; S-adenosysl-L-methionine:caffeic acid 3-O-methyltransferase (At3g53140)	-1.256	-1.457
Cs7g25580	K13066 caffeic acid 3-O-methyltransferase [EC:2.1.1.68] | (RefSeq) caffeic acid 3-O-methyltransferase-like (A)	Caffeic acid 3-O-methyltransferase; CAOMT; COMT; EC=2.1.1.68; S-adenosysl-L-methionine:caffeic acid 3-O-methyltransferase (At5g54160)	4.994	
Cs8g17630	K05909 laccase [EC:1.10.3.2] | (RefSeq) hypothetical protein (A)	Laccase-17; EC=1.10.3.2; Benzenediol:oxygen oxidoreductase 17; Diphenol oxidase 17; Urishiol oxidase 17; Flags: Precursor (At5g60020)	2.374	5.969
Cs8g18800	K05909 laccase [EC:1.10.3.2] | (RefSeq) laccase-17 (A)	Laccase-17; EC=1.10.3.2; Benzenediol:oxygen oxidoreductase 17; Diphenol oxidase 17; Urishiol oxidase 17; Flags: Precursor (At5g60020)	3.255	3.790
Cs8g19850	K05909 laccase [EC:1.10.3.2] | (RefSeq) hypothetical protein (A)	Laccase-4; EC=1.10.3.2; Benzenediol:oxygen oxidoreductase 4; Diphenol oxidase 4; Protein IRREGULAR XYLEM 12; Urishiol oxidase 4; Flags: Precursor (At2g38080)	1.651	3.446
novel.2285	K13066 caffeic acid 3-O-methyltransferase [EC:2.1.1.68] | (RefSeq) COMT1-2; caffeic acid O-methyltransferase (A)	COMT2_OCIBA Caffeic acid 3-O-methyltransferase 2; CAOMT-2; COMT-2; EC=2.1.1.68; S-adenosysl-L-methionine:caffeic acid 3-O-methyltransferase 2 (At3g53140)		-1.310
novel.2508	K13066 caffeic acid 3-O-methyltransferase [EC:2.1.1.68] | (RefSeq) anthranilate N-methyltransferase-like (A)	COMT1_MEDSA Caffeic acid 3-O-methyltransferase; CAOMT; COMT; EC=2.1.1.68; S-adenosysl-L-methionine:caffeic acid 3-O-methyltransferase (At5g54160)		2.654
novel.738	K13065 shikimate O-hydroxycinnamoyltransferase [EC:2.3.1.133] | (RefSeq) shikimate O-hydroxycinnamoyltransferase (A)	HST_ARATH Shikimate O-hydroxycinnamoyltransferase; EC=2.3.1.133; Hydroxycinnamoyl transferase; Hydroxycinnamoyl-Coenzyme A shikimate/quinate hydroxycinnamoyltransferase (At5g48930)		-1.997
novel.739	K13065 shikimate O-hydroxycinnamoyltransferase [EC:2.3.1.133] | (RefSeq) shikimate O-hydroxycinnamoyltransferase-like (A)	HST_ARATH Shikimate O-hydroxycinnamoyltransferase; EC=2.3.1.133; Hydroxycinnamoyl transferase; Hydroxycinnamoyl-Coenzyme A shikimate/quinate hydroxycinnamoyltransferase (At5g48930)		-1.375
novel.975	K13066 caffeic acid 3-O-methyltransferase [EC:2.1.1.68] | (RefSeq) hypothetical protein (A)	COMT1_MEDSA Caffeic acid 3-O-methyltransferase; CAOMT; COMT; EC=2.1.1.68; S-adenosysl-L-methionine:caffeic acid 3-O-methyltransferase (At5g54160)	2.726	
orange1.1t01084	K13066 caffeic acid 3-O-methyltransferase [EC:2.1.1.68] | (Kazusa) Lj2g3v3339490.1; - (A)	Caffeic acid 3-O-methyltransferase; CAOMT; COMT; EC=2.1.1.68; S-adenosysl-L-methionine:caffeic acid 3-O-methyltransferase (At3g53140)	-1.168	-1.088
orange1.1t01768	K20240 spermidine dicoumaroyl transferase [EC:2.3.1.249] | (RefSeq) acyl transferase 5 (A)	Acyl transferase 4 {ECO:0000305}; OsAT4 {ECO:0000303|PubMed:23391577}; EC=2.3.1.- {ECO:0000305}; p-coumaroyl-CoA monolignol transferase {ECO:0000305}; OsPMT {ECO:0000303|PubMed:22267741} (At3g62160)	2.871	5.366

orange1.1t02085	K13066 caffeic acid 3-O-methyltransferase [EC:2.1.1.68] | (RefSeq) caffeic acid 3-O-methyltransferase-like isoform X1 (A)	Caffeic acid 3-O-methyltransferase 1; CAOMT-1; COMT-1; EC=2.1.1.68; S-adenosysl-L-methionine:caffeic acid 3-O-methyltransferase 1 (At5g54160)		-2.085
orange1.1t02091	K13066 caffeic acid 3-O-methyltransferase [EC:2.1.1.68] | (RefSeq) hypothetical protein (A)	Caffeic acid 3-O-methyltransferase 1; CAOMT-1; COMT-1; EC=2.1.1.68; S-adenosysl-L-methionine:caffeic acid 3-O-methyltransferase 1 (At5g54160)		-2.365
orange1.1t03987	K13066 caffeic acid 3-O-methyltransferase [EC:2.1.1.68] | (RefSeq) caffeic acid 3-O-methyltransferase-like (A)	Caffeic acid 3-O-methyltransferase; CAOMT; COMT; EC=2.1.1.68; S-adenosysl-L-methionine:caffeic acid 3-O-methyltransferase (At5g54160)	6.849	
orange1.1t04194	K13066 caffeic acid 3-O-methyltransferase [EC:2.1.1.68] | (RefSeq) caffeic acid 3-O-methyltransferase-like (A)	Caffeic acid 3-O-methyltransferase; CAOMT; COMT; EC=2.1.1.68; S-adenosysl-L-methionine:caffeic acid 3-O-methyltransferase (At5g54160)	3.478	
orange1.1t05016	K13066 caffeic acid 3-O-methyltransferase [EC:2.1.1.68] | (RefSeq) caffeic acid 3-O-methyltransferase-like isoform X2 (A)	Caffeic acid 3-O-methyltransferase; CAOMT; COMT; EC=2.1.1.68; S-adenosysl-L-methionine:caffeic acid 3-O-methyltransferase (At5g54160)	1.113	1.584
orange1.1t05018	K13066 caffeic acid 3-O-methyltransferase [EC:2.1.1.68] | (RefSeq) caffeic acid 3-O-methyltransferase-like isoform X2 (A)	Caffeic acid 3-O-methyltransferase; CAOMT; COMT; EC=2.1.1.68; S-adenosysl-L-methionine:caffeic acid 3-O-methyltransferase (At5g54160)	1.136	1.021
orange1.1t05216	K13066 caffeic acid 3-O-methyltransferase [EC:2.1.1.68] | (RefSeq) caffeic acid 3-O-methyltransferase-like (A)	Flavone 3'-O-methyltransferase 1; AtOMT1; EC=2.1.1.42; Acetylserotonin O-methyltransferase OMT1 {ECO:0000305}; EC=2.1.1.4 {ECO:0000269|PubMed:25039887}; Caffeate O-methyltransferase 1; EC=2.1.1.68; Quercetin 3'-O-methyltransferase 1 (At3g53140)		-1.581
orange1.1t05232	K13066 caffeic acid 3-O-methyltransferase [EC:2.1.1.68] | (RefSeq) anthranilate N-methyltransferase-like (A)	Caffeic acid 3-O-methyltransferase; CAOMT; COMT; EC=2.1.1.68; S-adenosysl-L-methionine:caffeic acid 3-O-methyltransferase (At5g54160)	5.886	
orange1.1t05402	K00430 peroxidase [EC:1.11.1.7] | (RefSeq) peroxidase P7-like (A)	Peroxidase 52; Atperox P52; EC=1.11.1.7; ATP49; Flags: Precursor (At5g05340)		1.615
Pectin biosynthetic process [GO:0045489; P = 0.1408 (P3CR vs. P3R) and 0.1255 (P5CR vs. P5R)]				
Cs2g01430	K13648 alpha-1,4-galacturonosyltransferase [EC:2.4.1.43] | (RefSeq) probable galacturonosyltransferase 4 (A)	Probable galacturonosyltransferase 4; EC=2.4.1.-; Like glycosyl transferase 3 (At5g47780)		1.247
Cs2g19510	K15400 omega-hydroxypalmitate O-feruloyl transferase [EC:2.3.1.188] | (RefSeq) omega-hydroxypalmitate O-feruloyl transferase-like (A)	Omega-hydroxypalmitate O-feruloyl transferase; EC=2.3.1.188; Omega-hydroxyacid hydroxycinnamoyltransferase; Protein ALIPHATIC SUBERIN FERULOYL TRANSFERASE (At5g41040)		3.666
Cs2g25720	K15400 omega-hydroxypalmitate O-feruloyl transferase [EC:2.3.1.188] | (RefSeq) omega-hydroxypalmitate O-feruloyl transferase (A)	Omega-hydroxypalmitate O-feruloyl transferase; EC=2.3.1.188; Omega-hydroxyacid hydroxycinnamoyltransferase; Protein ALIPHATIC SUBERIN FERULOYL TRANSFERASE (At5g41040)		2.867

Cs4g12320	K20867 galacturonosyltransferase 12/13/14/15 [EC:2.4.1.-] | (RefSeq) probable galacturonosyltransferase 12 (A)	Probable galacturonosyltransferase 12; EC=2.4.1.-; Like glycosyl transferase 6; Protein IRREGULAR XYLEM 8 (At5g54690)	1.384	2.469
Cs5g28040	K14963 COMPASS component SWD3 | (RefSeq) transcriptional corepressor LEUNIG_HOMOLOG-like (A)	Transcriptional corepressor LEUNIG_HOMOLOG; Protein MUCILAGE-MODIFIED 1 {ECO:0000303|PubMed:11706181} (At2g32700)		1.712
Cs5g30310	K13648 alpha-1,4-galacturonosyltransferase [EC:2.4.1.43] | (RefSeq) hypothetical protein (A)	Probable galacturonosyltransferase 6; EC=2.4.1.- (At1g06780)	1.165	1.128
Cs6g08690	K16573 gamma-tubulin complex component 6 | (RefSeq) uncharacterized LOC103722329 (A)	Protein trichome birefringence-like 3 (At5g01360)	1.007	2.548
Cs8g21040	K15400 omega-hydroxypalmitate O-feruloyl transferase [EC:2.3.1.188] | (RefSeq) omega-hydroxypalmitate O-feruloyl transferase-like (A)	Omega-hydroxypalmitate O-feruloyl transferase; EC=2.3.1.188; Omega-hydroxyacid hydroxycinnamoyltransferase; Protein ALIPHATIC SUBERIN FERULOYL TRANSFERASE (At1g78990)	1.742	2.780
novel.1550	K13648 alpha-1,4-galacturonosyltransferase [EC:2.4.1.43] | (RefSeq) hypothetical protein (A)	GAUT8_ARATH Galacturonosyltransferase 8; EC=2.4.1.-; Glycosyltransferase QUASIMODO1 (At3g25140)	-1.020	
orange1.1t02619	K15400 omega-hydroxypalmitate O-feruloyl transferase [EC:2.3.1.188] | (RefSeq) hypothetical protein (A)	Omega-hydroxypalmitate O-feruloyl transferase; EC=2.3.1.188; Omega-hydroxyacid hydroxycinnamoyltransferase; Protein ALIPHATIC SUBERIN FERULOYL TRANSFERASE (At5g41040)	2.639	5.667
Cell wall macromolecule catabolic process [GO:0016998; P < 0.0001 (P3CR vs. P3R) and = 0.0702 (P5CR vs. P5R)]				
Cs1g24390	K19355 mannan endo-1,4-beta-mannosidase [EC:3.2.1.78] | (RefSeq) mannan endo-1,4-beta-mannosidase 7 (A)	Mannan endo-1,4-beta-mannosidase 7; EC=3.2.1.78; Beta-mannanase 7; Endo-beta-1,4-mannanase 7; AtMAN7; Flags: Precursor (At5g66460)		-1.878
Cs5g21860	K01183 chitinase [EC:3.2.1.14] | (RefSeq) hypothetical protein (A)	Chitinase 4; EC=3.2.1.14; OsChia2b; Pathogenesis related (PR)-3 chitinase 4; Flags: Precursor (At3g54420)	6.039	1.472
Cs5g21870	K01183 chitinase [EC:3.2.1.14] | (RefSeq) hypothetical protein (A)	Endochitinase EP3 {ECO:0000303|PubMed:11525512}; EC=3.2.1.14; Chitinase class IV {ECO:0000303|PubMed:11525512}; AtchitIV {ECO:0000303|PubMed:11525512}; Protein HOMOLOG OF CARROT EP3-3 CHITINASE {ECO:0000303|PubMed:11525512}; AtEP3 {ECO:0000303|PubMed:11525512}; Flags: Precursor (At3g54420)	3.265	
Cs6g10240	K19355 mannan endo-1,4-beta-mannosidase [EC:3.2.1.78] | (RefSeq) hypothetical protein (A)	Mannan endo-1,4-beta-mannosidase 6; EC=3.2.1.78; Beta-mannanase 6; Endo-beta-1,4-mannanase 6; AtMAN6; Flags: Precursor (At5g01930)	2.492	2.770
Cs8g01810	K20547 basic endochitinase B [EC:3.2.1.14] | (RefSeq) endochitinase-like (A)	Endochitinase A; CHN-A; EC=3.2.1.14; Flags: Precursor (At3g12500)	-2.339	-1.171

Cs8g01840	K20547 basic endochitinase B [EC:3.2.1.14] | (RefSeq) hypothetical protein (A)	Endochitinase CH25; EC=3.2.1.14; Flags: Precursor (At3g12500)	2.616	
Cs8g15720	K08235 xyloglucan:xyloglucosyl transferase [EC:2.4.1.207] | (RefSeq) xyloglucan endotransglucosylase/hydrolase protein 31-like (A)	Probable xyloglucan endotransglucosylase/hydrolase protein 32; At-XTH32; XTH-32; EC=2.4.1.207; Flags: Precursor (At2g36870)	1.919	
Cs9g02520	K20547 basic endochitinase B [EC:3.2.1.14] | (RefSeq) endochitinase CH25 (A)	Chitinase-like protein 2; AtCTL2; Flags: Precursor (At3g16920)		3.006
novel.1070	K01183 chitinase [EC:3.2.1.14] | (RefSeq) hypothetical protein (A)	CHIA_MAIZE Endochitinase A {ECO:0000303|PubMed:1551872}; EC=3.2.1.14 {ECO:0000269|PubMed:1551872, ECO:0000269|PubMed:1740436}; Seed chitinase A {ECO:0000303|PubMed:1551872}; Flags: Precursor (At3g54420)	7.442	
novel.1071	K01183 chitinase [EC:3.2.1.14] | (RefSeq) hypothetical protein (A)	CHI4_ORYSJ Chitinase 4; EC=3.2.1.14; OsChia2b; Pathogenesis related (PR)-3 chitinase 4; Flags: Precursor (At3g54420)	5.499	
orange1.1t03118	K01183 chitinase [EC:3.2.1.14] | (RefSeq) hypothetical protein (A)	Chitinase 4; EC=3.2.1.14; OsChia2b; Pathogenesis related (PR)-3 chitinase 4; Flags: Precursor (At3g54420)	5.505	
orange1.1t05124	K20547 basic endochitinase B [EC:3.2.1.14] | (RefSeq) endochitinase-like (A)	Endochitinase A; CHN-A; EC=3.2.1.14; Flags: Precursor (At3g12500)	-1.854	-1.018
Cellulose catabolic process [GO:0030245; P = 0.8181 (P3CR vs. P3R) and 0.3895 (P5CR vs. P5R)]				
Cs1g21260	K01179 endoglucanase [EC:3.2.1.4] | (RefSeq) endoglucanase 2-like (A)	Endoglucanase 24; EC=3.2.1.4; Endo-1,4-beta glucanase 24; Flags: Precursor (At1g19940)		-2.171
Cs2g12180	K01179 endoglucanase [EC:3.2.1.4] | (RefSeq) endoglucanase 12 (A)	Endoglucanase 12; EC=3.2.1.4; Endo-1,4-beta glucanase 12; OsGLU3 (At1g65610)		1.162
Cs2g17090	K01179 endoglucanase [EC:3.2.1.4] | (RefSeq) endoglucanase 8-like (A)	Endoglucanase 8; EC=3.2.1.4; Cellulase 1; AtCEL1; Endo-1,4-beta glucanase 8; Flags: Precursor (At1g70710)	-1.018	
Cs5g01400	K01179 endoglucanase [EC:3.2.1.4] | (RefSeq) endoglucanase CX (A)	Endoglucanase 1; EC=3.2.1.4; Abscission cellulase 1; Endo-1,4-beta-glucanase 1; Flags: Precursor (At1g02800)		3.825
Cs5g33460	K01179 endoglucanase [EC:3.2.1.4] | (RefSeq) endoglucanase 17 (A)	Endoglucanase 17; EC=3.2.1.4; Endo-1,4-beta glucanase 17; Flags: Precursor (At4g02290)		-1.318
Cs7g13990	K01179 endoglucanase [EC:3.2.1.4] | (RefSeq) endoglucanase 5 (A)	Endoglucanase 5; EC=3.2.1.4; Endo-1,4-beta glucanase 5; Flags: Precursor (At1g48930)		1.257
Cs9g14430	K01179 endoglucanase [EC:3.2.1.4] | (RefSeq) endoglucanase 4-like (A)	Endoglucanase 4; EC=3.2.1.4; Endo-1,4-beta glucanase 4; Flags: Precursor (At1g23210)	-1.165	

novel.301	K01179 endoglucanase [EC:3.2.1.4] | (RefSeq) endoglucanase 12 (A)	GUN7_ARATH Endoglucanase 7; EC=3.2.1.4; Endo-1,4-beta glucanase 7 (At1g65610)		1.225
Lignin catabolic process [GO:0046274; P < 0.0001 (P3CR vs. P3R) and 0.0001 (P5CR vs. P5R]				
Cs1g24250	K05909 laccase [EC:1.10.3.2] | (RefSeq) laccase-11-like (A)	Laccase-11; EC=1.10.3.2; Benzenediol:oxygen oxidoreductase 11; Diphenol oxidase 11; Urishiol oxidase 11; Flags: Precursor (At5g03260)	3.444	6.447
Cs6g06880	K05909 laccase [EC:1.10.3.2] | (RefSeq) hypothetical protein (A)	Laccase-17; EC=1.10.3.2; Benzenediol:oxygen oxidoreductase 17; Diphenol oxidase 17; Urishiol oxidase 17; Flags: Precursor (At5g60020)		4.438
Cs6g06920	K05909 laccase [EC:1.10.3.2] | (RefSeq) laccase-17-like (A)	Laccase-17; EC=1.10.3.2; Benzenediol:oxygen oxidoreductase 17; Diphenol oxidase 17; Urishiol oxidase 17; Flags: Precursor (At5g60020)	1.652	3.497
Cs6g07400	K05909 laccase [EC:1.10.3.2] | (RefSeq) laccase-7-like (A)	Laccase-7; EC=1.10.3.2; Benzenediol:oxygen oxidoreductase 7; Diphenol oxidase 7; Urishiol oxidase 7; Flags: Precursor (At3g09220)	1.881	
Cs6g07410	K05909 laccase [EC:1.10.3.2] | (RefSeq) hypothetical protein (A)	Laccase-7; EC=1.10.3.2; Benzenediol:oxygen oxidoreductase 7; Diphenol oxidase 7; Urishiol oxidase 7; Flags: Precursor (At3g09220)	1.498	2.464
Cs6g07450	K05909 laccase [EC:1.10.3.2] | (RefSeq) laccase-7-like (A)	Laccase-7; EC=1.10.3.2; Benzenediol:oxygen oxidoreductase 7; Diphenol oxidase 7; Urishiol oxidase 7; Flags: Precursor (At3g09220)		2.442
Cs6g07800	K05909 laccase [EC:1.10.3.2] | (RefSeq) hypothetical protein (A)	Laccase-4; EC=1.10.3.2; Benzenediol:oxygen oxidoreductase 4; Diphenol oxidase 4; Protein IRREGULAR XYLEM 12; Urishiol oxidase 4; Flags: Precursor (At2g38080)	1.504	3.815
Cs6g11860	K05909 laccase [EC:1.10.3.2] | (RefSeq) hypothetical protein (A)	Laccase-11; EC=1.10.3.2; Benzenediol:oxygen oxidoreductase 11; Diphenol oxidase 11; Urishiol oxidase 11; Flags: Precursor (At5g03260)	2.724	5.299
Cs7g23490	K05909 laccase [EC:1.10.3.2] | (RefSeq) hypothetical protein (A)	Laccase-17; EC=1.10.3.2; Benzenediol:oxygen oxidoreductase 17; Diphenol oxidase 17; Urishiol oxidase 17; Flags: Precursor (At5g60020)		3.109
Cs7g30410	K05909 laccase [EC:1.10.3.2] | (RefSeq) hypothetical protein (A)	Laccase-5; EC=1.10.3.2; Benzenediol:oxygen oxidoreductase 5; Diphenol oxidase 5; Urishiol oxidase 5; Flags: Precursor (At2g40370)	1.431	4.293
Cs7g31620	K05909 laccase [EC:1.10.3.2] | (RefSeq) hypothetical protein (A)	Laccase-22; EC=1.10.3.2; Benzenediol:oxygen oxidoreductase 22; Diphenol oxidase 22; Urishiol oxidase 22; Flags: Precursor (At2g38080)	1.349	2.990
Cs8g11710	K05909 laccase [EC:1.10.3.2] | (RefSeq) laccase-13-like (A)	Laccase-3; EC=1.10.3.2; Benzenediol:oxygen oxidoreductase 3; Diphenol oxidase 3; Urishiol oxidase 3; Flags: Precursor (At2g30210)	2.670	3.781
Cs8g17630	K05909 laccase [EC:1.10.3.2] | (RefSeq) hypothetical protein (A)	Laccase-17; EC=1.10.3.2; Benzenediol:oxygen oxidoreductase 17; Diphenol oxidase 17; Urishiol oxidase 17; Flags: Precursor (At5g60020)	2.374	5.969
Cs8g18800	K05909 laccase [EC:1.10.3.2] | (RefSeq) laccase-17 (A)	Laccase-17; EC=1.10.3.2; Benzenediol:oxygen oxidoreductase 17; Diphenol oxidase 17; Urishiol oxidase 17; Flags: Precursor (At5g60020)	3.255	3.790

Cs8g19850	K05909 laccase [EC:1.10.3.2] | (RefSeq) hypothetical protein (A)	Laccase-4; EC=1.10.3.2; Benzenediol:oxygen oxidoreductase 4; Diphenol oxidase 4; Protein IRREGULAR XYLEM 12; Urishiol oxidase 4; Flags: Precursor (At2g38080)	1.651	3.446
orange1.1t00518	K05909 laccase [EC:1.10.3.2] | (RefSeq) hypothetical protein (A)	Laccase-6; EC=1.10.3.2; Benzenediol:oxygen oxidoreductase 6; Diphenol oxidase 6; Urishiol oxidase 6; Flags: Precursor (At2g46570)		1.676
Pectin catabolic process [GO:0045490; P = 0.5857 (P3CR vs. P3R) and 0.8134 (P5CR vs. P5R)]				
Cs1g16560	K01051 pectinesterase [EC:3.1.1.11] | (RefSeq) pectinesterase 3 (A)	Pectinesterase 3; PE 3; EC=3.1.1.11; Pectin methylesterase 3; Flags: Precursor (At1g53840)		1.217
Cs2g16380	K01051 pectinesterase [EC:3.1.1.11] | (RefSeq) putative pectinesterase 11 (A)	Putative pectinesterase 11; PE 11; EC=3.1.1.11; Pectin methylesterase 11; AtPME11 (At2g21610)		1.477
Cs2g23970	K01728 pectate lyase [EC:4.2.2.2] | (RefSeq) hypothetical protein (A)	Probable pectate lyase P18; EC=4.2.2.2; Style development-specific protein 9612; Flags: Precursor (At3g27400)	1.733	2.124
Cs4g06670	K01051 pectinesterase [EC:3.1.1.11] | (RefSeq) probable pectinesterase/pectinesterase inhibitor 12 (A)	Probable pectinesterase/pectinesterase inhibitor 12; Includes: Pectinesterase inhibitor 12; Pectin methylesterase inhibitor 12; Includes: Pectinesterase 12; PE 12; EC=3.1.1.11; Pectin methylesterase 12; AtPME12; Flags: Precursor (At2g26440)		-1.091
Cs5g33410	K01051 pectinesterase [EC:3.1.1.11] | (RefSeq) probable pectinesterase/pectinesterase inhibitor 20 (A)	Probable pectinesterase/pectinesterase inhibitor 41; Includes: Pectinesterase inhibitor 41; Pectin methylesterase inhibitor 41; Includes: Pectinesterase 41; PE 41; EC=3.1.1.11; AtPMEpcrB; Pectin methylesterase 41; AtPME41; Flags: Precursor (At4g02330)	-1.001	
Cs5g34710	K01728 pectate lyase [EC:4.2.2.2] | (RefSeq) hypothetical protein (A)	Probable pectate lyase 10; EC=4.2.2.2; Flags: Precursor (At3g24670)		-1.154
Cs6g11440	K01051 pectinesterase [EC:3.1.1.11] | (RefSeq) probable pectinesterase 8 (A)	Probable pectinesterase 8; PE 8; EC=3.1.1.11; Pectin methylesterase 2; AtPME2; Pectin methylesterase 8; AtPME8; Flags: Precursor (At1g05310)		5.569
Cs7g09860	K01728 pectate lyase [EC:4.2.2.2] | (RefSeq) probable pectate lyase 5 (A)	Probable pectate lyase 5; EC=4.2.2.2; Flags: Precursor (At1g67750)	-1.969	-2.841
Cs9g14450	K01051 pectinesterase [EC:3.1.1.11] | (RefSeq) pectinesterase 2-like (A)	Pectinesterase 2; PE 2; EC=3.1.1.11; Pectin methylesterase; Flags: Precursor (At2g45220)	-1.167	
orange1.1t00214	K01051 pectinesterase [EC:3.1.1.11] | (RefSeq) hypothetical protein (A)	Pectinesterase 2; PE 2; EC=3.1.1.11; Pectin methylesterase; Flags: Precursor (At2g45220)	1.028	
orange1.1t01085	K01051 pectinesterase [EC:3.1.1.11] | (RefSeq) hypothetical protein (A)	Probable pectinesterase 15; PE 15; EC=3.1.1.11; Pectin methylesterase 15; AtPME15; Flags: Precursor (At2g36710)		2.963

orange1.1t01727	K01051 pectinesterase [EC:3.1.1.11] | (RefSeq) probable pectinesterase 53 (A)	Probable pectinesterase 53; PE 53; EC=3.1.1.11; Pectin methylesterase 53; AtPME53; Flags: Precursor (At5g19730)	-1.455	
Hemicellulose metabolic process [GO:0010410; P = 0.0115 (P3CR vs. P3R) and < 0.0001 (P5CR vs. P5R)]				
Cs1g05500	K20890 xylan alpha-glucuronosyltransferase [EC:2.4.1.-] | (RefSeq) UDP-glucuronate:xylan alpha-glucuronosyltransferase 1 (A)	UDP-glucuronate:xylan alpha-glucuronosyltransferase 1; UDP-GlcA:xylan glucuronyltransferase 1; EC=2.4.1.-; Glycogenin-like protein 1; Plant glycogenin-like starch initiation protein 1; Protein GLUCURONIC ACID SUBSTITUTION OF XYLAN 1; AtGUX1 (At3g18660)	1.889	3.881
Cs1g21130	K08235 xyloglucan:xyloglucosyl transferase [EC:2.4.1.207] | (RefSeq) hypothetical protein (A)	Probable xyloglucan endotransglucosylase/hydrolase protein 6; At-XTH6; XTH-6; EC=2.4.1.207; Flags: Precursor (At5g65730)		-1.344
Cs2g14920	K08235 xyloglucan:xyloglucosyl transferase [EC:2.4.1.207] | (RefSeq) hypothetical protein (A)	Probable xyloglucan endotransglucosylase/hydrolase protein 5; At-XTH5; XTH-5; EC=2.4.1.207; Flags: Precursor (At5g13870)	-1.016	
Cs2g17920	K08235 xyloglucan:xyloglucosyl transferase [EC:2.4.1.207] | (RefSeq) probable xyloglucan endotransglucosylase/hydrolase protein 28 (A)	Probable xyloglucan endotransglucosylase/hydrolase protein 28; At-XTH28; XTH-28; EC=2.4.1.207; Flags: Precursor (At1g14720)		1.202
Cs2g25390	K18801 glucuronoxylan 4-O-methyltransferase [EC:2.1.1.112] | (RefSeq) glucuronoxylan 4-O-methyltransferase-like protein (DUF579) (A)	Probable methyltransferase At1g27930; EC=2.1.1.- {ECO:0000305} (At1g27930)	-1.027	
Cs3g03080	K18801 glucuronoxylan 4-O-methyltransferase [EC:2.1.1.112] | (RefSeq) glucuronoxylan 4-O-methyltransferase 1 (A)	Glucuronoxylan 4-O-methyltransferase 1; EC=2.1.1.112 (At1g09610)	1.160	3.145
Cs3g08950	K08235 xyloglucan:xyloglucosyl transferase [EC:2.4.1.207] | (RefSeq) hypothetical protein (A)	Probable xyloglucan endotransglucosylase/hydrolase protein 30; At-XTH30; XTH-30; EC=2.4.1.207; Flags: Precursor (At1g32170)		1.305
Cs3g20910	K20890 xylan alpha-glucuronosyltransferase [EC:2.4.1.-] | (RefSeq) putative UDP-glucuronate:xylan alpha-glucuronosyltransferase 3 (A)	Putative UDP-glucuronate:xylan alpha-glucuronosyltransferase 3; UDP-GlcA:xylan glucuronyltransferase 3; EC=2.4.1.-; Glycogenin-like protein 3; Plant glycogenin-like starch initiation protein 2; Protein GLUCURONIC ACID SUBSTITUTION OF XYLAN 3; AtGUX3 (At1g77130)		1.189
Cs3g23600	K20890 xylan alpha-glucuronosyltransferase [EC:2.4.1.-] | (RefSeq) UDP-glucuronate:xylan alpha-glucuronosyltransferase 2 (A)	UDP-glucuronate:xylan alpha-glucuronosyltransferase 2; UDP-GlcA:xylan glucuronyltransferase 2; EC=2.4.1.-; Glycogenin-like protein 2; Plant glycogenin-like starch initiation protein 3; Protein GLUCURONIC ACID SUBSTITUTION OF XYLAN 2; AtGUX2 (At4g33330)	1.620	4.416
Cs4g03050	K14504 xyloglucan:xyloglucosyl transferase TCH4 [EC:2.4.1.207] | (RefSeq) probable xyloglucan	Probable xyloglucan endotransglucosylase/hydrolase protein 23; At-XTH23; XTH-23; EC=2.4.1.207; Flags: Precursor (At4g25810)		1.738

	endotransglucosylase/hydrolase protein 23 (A)			
Cs4g03060	K14504 xyloglucan:xyloglucosyl transferase TCH4 [EC:2.4.1.207] | (RefSeq) probable xyloglucan endotransglucosylase/hydrolase protein 23 (A)	Probable xyloglucan endotransglucosylase/hydrolase protein 23; At-XTH23; XTH-23; EC=2.4.1.207; Flags: Precursor (At4g25810)		1.400
Cs4g03070	K14504 xyloglucan:xyloglucosyl transferase TCH4 [EC:2.4.1.207] | (RefSeq) probable xyloglucan endotransglucosylase/hydrolase protein 23 (A)	Probable xyloglucan endotransglucosylase/hydrolase protein 23; At-XTH23; XTH-23; EC=2.4.1.207; Flags: Precursor (At4g25810)		1.428
Cs4g03130	K14504 xyloglucan:xyloglucosyl transferase TCH4 [EC:2.4.1.207] | (RefSeq) hypothetical protein (A)	Probable xyloglucan endotransglucosylase/hydrolase protein 23; At-XTH23; XTH-23; EC=2.4.1.207; Flags: Precursor (At4g25810)	1.252	1.931
Cs4g03140	K14504 xyloglucan:xyloglucosyl transferase TCH4 [EC:2.4.1.207] | (RefSeq) hypothetical protein (A)	Probable xyloglucan endotransglucosylase/hydrolase protein 23; At-XTH23; XTH-23; EC=2.4.1.207; Flags: Precursor (At4g25810)		1.602
Cs4g03200	K14504 xyloglucan:xyloglucosyl transferase TCH4 [EC:2.4.1.207] | (RefSeq) probable xyloglucan endotransglucosylase/hydrolase protein 23 (A)	Probable xyloglucan endotransglucosylase/hydrolase protein 23; At-XTH23; XTH-23; EC=2.4.1.207; Flags: Precursor (At4g25810)		1.214
Cs4g12320	K20867 galacturonosyltransferase 12/13/14/15 [EC:2.4.1.-] | (RefSeq) probable galacturonosyltransferase 12 (A)	Probable galacturonosyltransferase 12; EC=2.4.1.-; Like glycosyl transferase 6; Protein IRREGULAR XYLEM 8 (At5g54690)	1.384	2.469
Cs5g18370	K18801 glucuronoxylan 4-O-methyltransferase [EC:2.1.1.112] | (RefSeq) glucuronoxylan 4-O-methyltransferase 3 (A)	Glucuronoxylan 4-O-methyltransferase 3; EC=2.1.1.112 (At1g33800)		2.166
Cs5g29020	K20888 xyloglucan galactosyltransferase MUR3 [EC:2.4.1.-] | (RefSeq) xyloglucan galactosyltransferase KATAMARI1 (A)	Xyloglucan galactosyltransferase MUR3 {ECO:0000305}; EC=2.4.1.- {ECO:0000305}; Protein KATAMARI {ECO:0000303|PubMed:15863516}; Protein MURUS 3 {ECO:0000303|PubMed:12837954}; AtMUR3 {ECO:0000303|PubMed:12837954}; Protein SHORT ROOT IN SALT MEDIUM 3 {ECO:0000303|PubMed:23571490} (At2g20370)		1.430
Cs6g04870	K20869 putative beta-1,4-xylosyltransferase IRX9 [EC:2.4.2.-] | (RefSeq) probable beta-1,4-xylosyltransferase IRX9 (A)	Probable beta-1,4-xylosyltransferase IRX9; EC=2.4.2.-; Protein IRREGULAR XYLEM 9; Xylan xylosyltransferase IRX9 (At2g37090)	1.695	3.472
Cs6g08690	K16573 gamma-tubulin complex component 6 | (RefSeq) uncharacterized LOC103722329 (A)	Protein trichome birefringence-like 3 (At5g01360)	1.007	2.548
Cs6g09180	K16573 gamma-tubulin complex component 6 | (RefSeq) uncharacterized LOC103722329 (A)	Protein trichome birefringence-like 34 (At2g38320)		1.999
Cs6g16270	K01209 alpha-N-arabinofuranosidase [EC:3.2.1.55] | (RefSeq) alpha-L-arabinofuranosidase 1-like (A)	Alpha-L-arabinofuranosidase 1; AtASD1; EC=3.2.1.55; Beta-D-xylosidase; EC=3.2.1.-; Flags: Precursor (At3g10740)		2.031

Cs6g17170	K16573 gamma-tubulin complex component 6 | (RefSeq) uncharacterized LOC103722329 (A)	Protein trichome birefringence-like 33 (At2g40320)		-1.021
Cs7g30510	K16573 gamma-tubulin complex component 6 | (RefSeq) uncharacterized LOC103722329 (A)	Protein trichome birefringence-like 33 (At2g40320)	1.388	2.970
Cs7g30960	K16573 gamma-tubulin complex component 6 | (RefSeq) uncharacterized LOC103722329 (A)	Protein ESKIMO 1; Protein trichome birefringence-like 29 (At3g55990)		2.314
Cs8g05250	K18801 glucuronoxylan 4-O-methyltransferase [EC:2.1.1.112] | (RefSeq) hypothetical protein (A)	Protein IRX15-LIKE (At5g67210)		-1.292
Cs8g10780	K20870 putative beta-1,4-xylosyltransferase IRX10 [EC:2.4.2.-] | (RefSeq) probable beta-1,4-xylosyltransferase IRX10 (A)	Probable beta-1,4-xylosyltransferase IRX10; EC=2.4.2.-; Glucuronoxylan glucuronosyltransferase 1; AtGUT1; Glucuronoxylan glucuronosyltransferase 2; Protein IRREGULAR XYLEM 10; Xylan xylosyltransferase IRX10 (At1g27440)	1.084	2.225
Cs8g13800	K05349 beta-glucosidase [EC:3.2.1.21] | (RefSeq) beta-glucosidase BoGH3B-like (A)	Beta-xylosidase/alpha-L-arabinofuranosidase 2 {ECO:0000303|PubMed:17615411, ECO:0000312|EMBL:ABQ45228.1}; Xylan 1,4-beta-xylosidase/Alpha-L-arabinofuranosidase 2; MsXyl2 {ECO:0000303|PubMed:17615411}; Includes: Beta-xylosidase; EC=3.2.1.37; 1,4-beta-D-xylan xylohydrolase {ECO:0000250|UniProtKB:P48792}; Xylan 1,4-beta-xylosidase; Includes: Alpha-L-arabinofuranosidase; Arabinosidase {ECO:0000250|UniProtKB:P48792}; EC=3.2.1.55; Flags: Precursor (At5g20950)		-1.094
Cs8g15710	K20889 probable glucuronoxylan glucuronosyltransferase IRX7 [EC2.4.1.-] | (RefSeq) probable glucuronoxylan glucuronosyltransferase IRX7 (A)	Probable glucuronoxylan glucuronosyltransferase IRX7; EC=2.4.1.-; Protein FRAGILE FIBER 8; Protein IRREGULAR XYLEM 7 (At2g28110)		1.396
Cs8g15720	K08235 xyloglucan:xyloglucosyl transferase [EC:2.4.1.207] | (RefSeq) xyloglucan endotransglucosylase/hydrolase protein 31-like (A)	Probable xyloglucan endotransglucosylase/hydrolase protein 32; At-XTH32; XTH-32; EC=2.4.1.207; Flags: Precursor (At2g36870)	1.919	
orange1.1t03392	K20870 putative beta-1,4-xylosyltransferase IRX10 [EC:2.4.2.-] | (RefSeq) uncharacterized protein LOC103936878 (A)	Probable beta-1,4-xylosyltransferase IRX10; EC=2.4.2.-; Glucuronoxylan glucuronosyltransferase 1; AtGUT1; Glucuronoxylan glucuronosyltransferase 2; Protein IRREGULAR XYLEM 10; Xylan xylosyltransferase IRX10 (At3g57630)		1.540
orange1.1t04064	K18801 glucuronoxylan 4-O-methyltransferase [EC:2.1.1.112] | (RefSeq) hypothetical protein (A)	Protein IRX15-LIKE (At5g67210)	1.609	3.639
