# Supplementary material for: Mechanisms by Which Increased pH Ameliorates Copper Excess in Citrus sinensis Roots: Insight from a Combined Analysis of Physiology, Transcriptome, and Metabolome
Source: Plants (Basel). 2024 Oct 31;13(21):3054. doi: 10.3390/plants13213054 (PMC11548300; doi:10.3390/plants13213054)
Supplement: Supplementary file 1 [file plants-13-03054-s001.zip › Table S13SecondRevision.pdf]

Table S13. DEGs related to plant organ senescence in P3CR vs. P3R and/or P5CR vs. P5R

**Accession No.**	**KEGG**	**Swissprot (KOG)**	**Log$_2$(FC)**	
			P3CR vs. P3R	P5CR vs. P5R
Plant organ senescence [GO:0090693; P = 0.1518 (P3CR vs. P3R) and 0.1928 (P5CR vs. P5R)]				
Cs1g03720	K22048 mechanosensitive ion channel protein 4/5/6/7/8/9/10 | (RefSeq) mechanosensitive ion channel protein 10-like (A)	Mechanosensitive ion channel protein 10; Mechanosensitive channel of small conductance-like 10; MscS-Like protein 10; AtMSL10 (At5g12080)	1.255	
Cs1g09630	K00521 ferric-chelate reductase [EC:1.16.1.7] | (RefSeq) ferric reduction oxidase 7, chloroplastic-like (A)	NAC domain-containing protein 53 {ECO:0000303|PubMed:15029955}; ANAC053 {ECO:0000303|PubMed:15029955}; Protein ANTHER INDEHISCENCE FACTOR {ECO:0000303|PubMed:24323506}; Protein NTM1-like 4 {ECO:0000303|PubMed:17158162} (At3g10500)	1.215	
Cs1g09670	K00521 ferric-chelate reductase [EC:1.16.1.7] | (RefSeq) ferric reduction oxidase 7, chloroplastic-like (A)	NAC domain-containing protein 92 {ECO:0000303|PubMed:15029955}; ANAC092 {ECO:0000303|PubMed:15029955}; AtNAC2 {ECO:0000303|PubMed:16359384}; AtNAC6 {ECO:0000303|PubMed:21511905}; Protein ORESARA 1 {ECO:0000303|PubMed:9351240} (At3g18400)	1.261	1.377
Cs1g12310	K18054 2'-deoxymugineic-acid 2'-dioxygenase / mugineic-acid 3-dioxygenase [EC:1.14.11.24 1.14.11.25] | (RefSeq) 2'-deoxymugineic-acid 2'-dioxygenase-like (A)	Protein DMR6-LIKE OXYGENASE 1 {ECO:0000303|PubMed:25376907}; EC=1.14.11.- {ECO:0000255|PROSITE-ProRule:PRU00805}; 2-oxoglutarate (2OG)-Fe(II) oxygenase-like protein DLO1 {ECO:0000303|PubMed:25376907}; Protein SENESCENCE-ASSOCIATED GENE 108 {ECO:0000303|PubMed:23959884}; Salicylate 3-hydroxylase DLO1 {ECO:0000303|PubMed:23959884}; S3H DLO1 {ECO:0000303|PubMed:23959884}; SA 3-hydroxylase DLO1 {ECO:0000303|PubMed:23959884}; Salicylic acid 3-hydroxylase DLO1 {ECO:0000303|PubMed:23959884}; EC=1.14.13.- {ECO:0000269|PubMed:23959884} (At4g10500)		-1.609
Cs1g17350	K15333 tRNA guanosine-2'-O-methyltransferase [EC:2.1.1.34] | (RefSeq) uncharacterized protein LOC104224633 (A)	Subtilisin-like protease SBT1.4 {ECO:0000303|PubMed:16193095}; EC=3.4.21.- {ECO:0000255|PROSITE-ProRule:PRU10082}; Subtilase subfamily 1 member 4 {ECO:0000303|PubMed:16193095}; AtSBT1.4 {ECO:0000303|PubMed:16193095}; Flags: Precursor (At3g14067)	1.240	

Cs2g04090	K05658 ATP-binding cassette, subfamily B (MDR/TAP), member 1 [EC:7.6.2.2] | (RefSeq) uncharacterized protein LOC111781709 (A)	Protein SRG1; AtSRG1; Protein SENESCENCE-RELATED GENE 1 (At1g17020)	-1.331	-1.569
Cs2g04110	K05658 ATP-binding cassette, subfamily B (MDR/TAP), member 1 [EC:7.6.2.2] | (RefSeq) uncharacterized protein LOC111781709 (A)	Protein SRG1; AtSRG1; Protein SENESCENCE-RELATED GENE 1 (At1g78550)	-1.502	-1.096
Cs2g04940	K01365 cathepsin L [EC:3.4.22.15] | (RefSeq) hypothetical protein (A)	Senescence-specific cysteine protease SAG39 {ECO:0000305}; EC=3.4.22.- {ECO:0000305}; Cysteine proteinase SAG39 {ECO:0000305}; Protein SENESCENCE-ASSOCIATED GENE 39 {ECO:0000250|UniProtKB:Q7XWK5}; Flags: Precursor (At5g45890)	2.537	-5.958
Cs2g06670		Endonuclease 1; AtENDO1; EC=3.1.30.1; Bifunctional nuclease I; AtBFN1; Deoxyribonuclease ENDO1; Single-stranded-nucleate endonuclease ENDO1; Flags: Precursor (At1g11190)	1.691	3.814
Cs2g15480	K16292 KDEL-tailed cysteine endopeptidase [EC:3.4.22.-] | (RefSeq) senescence-specific cysteine protease SAG39-like (A)	Senescence-specific cysteine protease SAG39 {ECO:0000305}; EC=3.4.22.- {ECO:0000305}; Cysteine proteinase SAG39 {ECO:0000305}; Protein SENESCENCE-ASSOCIATED GENE 39 {ECO:0000250|UniProtKB:Q7XWK5}; Flags: Precursor (At5g45890)		-5.160
Cs2g15490	K16292 KDEL-tailed cysteine endopeptidase [EC:3.4.22.-] | (RefSeq) senescence-specific cysteine protease SAG39-like (A)	Senescence-specific cysteine protease SAG39 {ECO:0000305}; EC=3.4.22.- {ECO:0000305}; Cysteine proteinase SAG39 {ECO:0000305}; Protein SENESCENCE-ASSOCIATED GENE 39 {ECO:0000250|UniProtKB:Q7XWK5}; Flags: Precursor (At5g45890)	1.346	-7.852
Cs2g15700	K16292 KDEL-tailed cysteine endopeptidase [EC:3.4.22.-] | (RefSeq) senescence-specific cysteine protease SAG39 (A)	Senescence-specific cysteine protease SAG39 {ECO:0000305}; EC=3.4.22.- {ECO:0000305}; Cysteine proteinase SAG39 {ECO:0000305}; Protein SENESCENCE-ASSOCIATED GENE 39 {ECO:0000303|PubMed:20439547}; Flags: Precursor (At5g45890)	2.123	-3.210
Cs2g27790	K01365 cathepsin L [EC:3.4.22.15] | (RefSeq) zingipain-1-like (A)	Senescence-specific cysteine protease SAG12 {ECO:0000305}; EC=3.4.22.- {ECO:0000305}; Cysteine proteinase SAG12 {ECO:0000305}; Protein SENESCENCE-ASSOCIATED GENE 12 {ECO:0000303|Ref.6}; Flags: Precursor (At3g49340)		-1.325
Cs5g16310	K05278 flavonol synthase [EC:1.14.11.23] | (RefSeq) flavonol synthase/flavanone 3-hydroxylase (A)	Protein DOWNY MILDEW RESISTANCE 6 {ECO:0000303|PubMed:15986928}; AtDMR6 {ECO:0000303|PubMed:15986928}; EC=1.14.11.- {ECO:0000255|PROSITE-ProRule:PRU00805}; 2-oxoglutarate (2OG)-Fe(II) oxygenase-like protein DMR6		-1.118

		{ECO:0000303|PubMed:18248595}; Salicylate 3-hydroxylase DMR6 {ECO:0000305}; S3H DMR6 {ECO:0000305}; SA 3-hydroxylase DMR6 {ECO:0000305}; Salicylic acid 3-hydroxylase DMR6 {ECO:0000305}; EC=1.14.13.- {ECO:0000250|UniProtKB:Q9ZSA8} (At5g24530)		
Cs5g22560	K00521 ferric-chelate reductase [EC:1.16.1.7] | (RefSeq) ferric reduction oxidase 7, chloroplastic-like (A)	NAC domain-containing protein 83 {ECO:0000303|PubMed:15029955}; ANAC083 {ECO:0000303|PubMed:15029955}; Protein VND-INTERACTING 2 {ECO:0000303|PubMed:20388856} (At5g13180)		-4.953
Cs5g28710	K05282 gibberellin-44 dioxygenase [EC:1.14.11.12] | (RefSeq) gibberellin 20 oxidase 1-D-like (A)	Protein DMR6-LIKE OXYGENASE 1 {ECO:0000303|PubMed:25376907}; EC=1.14.11.- {ECO:0000255|PROSITE-ProRule:PRU00805}; 2-oxoglutarate (2OG)-Fe(II) oxygenase-like protein DLO1 {ECO:0000303|PubMed:25376907}; Protein SENESCENCE-ASSOCIATED GENE 108 {ECO:0000303|PubMed:23959884}; Salicylate 3-hydroxylase DLO1 {ECO:0000303|PubMed:23959884}; S3H DLO1 {ECO:0000303|PubMed:23959884}; SA 3-hydroxylase DLO1 {ECO:0000303|PubMed:23959884}; Salicylic acid 3-hydroxylase DLO1 {ECO:0000303|PubMed:23959884}; EC=1.14.13.- {ECO:0000269|PubMed:23959884} (At3g19000)	2.636	
Cs6g02660	K05658 ATP-binding cassette, subfamily B (MDR/TAP), member 1 [EC:7.6.2.2] | (RefSeq) uncharacterized protein LOC111781709 (A)	Protein SRG1; AtSRG1; Protein SENESCENCE-RELATED GENE 1 (At1g17020)		1.094
Cs6g10120	K13459 disease resistance protein RPS2 | (RefSeq) disease resistance protein RPS2 (A)	Probable WRKY transcription factor 70; WRKY DNA-binding protein 70 (At3g56400)		-1.503
Cs7g02970	K15382 solute carrier family 50 (sugar transporter) | (RefSeq) hypothetical protein (A)	Bidirectional sugar transporter SWEET15 {ECO:0000303|PubMed:21107422}; AtSWEET15 {ECO:0000303|PubMed:21107422}; Protein SUGARS WILL EVENTUALLY BE EXPORTED TRANSPORTERS 15 {ECO:0000303|PubMed:21107422}; Senescence-associated protein 29 {ECO:0000303|PubMed:20963606} (At5g13170)		3.300
Cs7g07830	K08869 aarF domain-containing kinase | (RefSeq) predicted protein (A)	Transcription factor KUA1 {ECO:0000303|PubMed:24806884}; Myb-related protein H {ECO:0000303|PubMed:23888064}; AtMYBH {ECO:0000303|PubMed:23888064}; AtMYBS3	-1.281	-1.251

		{ECO:0000303|PubMed:25920996}; MYBS3-homolg protein {ECO:0000303|PubMed:25920996}; Protein KUODA1 {ECO:0000303|PubMed:24806884} (At1g70000)		
Cs7g15850	K22048 mechanosensitive ion channel protein 4/5/6/7/8/9/10 | (RefSeq) mechanosensitive ion channel protein 10-like (A)	Mechanosensitive ion channel protein 10; Mechanosensitive channel of small conductance-like 10; MscS-Like protein 10; AtMSL10 (At5g12080)		1.491
Cs7g17600	K00521 ferric-chelate reductase [EC:1.16.1.7] | (RefSeq) ferric reduction oxidase 7, chloroplastic-like (A)	Transcription factor JUNGBRUNNEN 1; NAC domain-containing protein 42; ANAC042 (At2g43000)	1.057	1.759
Cs7g22760	K14638 solute carrier family 15 (peptide/histidine transporter), member 3/4 | (RefSeq) hypothetical protein (A)	Protein NRT1/ PTR FAMILY 7.3; AtNPF7.3; Nitrate transporter 1.5 (At1g32450)		1.871
Cs7g27330	K08472 mlo protein | (RefSeq) hypothetical protein (A)	MLO-like protein 12; AtMlo12; AtMlo18 (At1g11310)		1.563
Cs7g31460	K05658 ATP-binding cassette, subfamily B (MDR/TAP), member 1 [EC:7.6.2.2] | (RefSeq) uncharacterized protein LOC111781709 (A)	Protein SRG1; AtSRG1; Protein SENESCENCE-RELATED GENE 1 (At3g21420)		-4.960
Cs7g32220	K00699 glucuronosyltransferase [EC:2.4.1.17] | (RefSeq) UDP-glycosyltransferase 76G1-like (A)	UDP-glycosyltransferase 76B1 {ECO:0000305}; EC=2.4.1.- {ECO:0000305} (At3g11340)		-2.117
Cs8g03950	K05278 flavonol synthase [EC:1.14.11.23] | (RefSeq) flavonol synthase/flavanone 3-hydroxylase-like (A)	Protein SRG1; AtSRG1; Protein SENESCENCE-RELATED GENE 1 (At1g49390)		-1.217
Cs8g16870	K00521 ferric-chelate reductase [EC:1.16.1.7] | (RefSeq) ferric reduction oxidase 7, chloroplastic-like (A)	NAC transcription factor 29; AtNAC029; Protein NAC-LIKE, ACTIVATED BY AP3/PI; AtNAP (At1g69490)		-6.441
Cs8g18300	K00699 glucuronosyltransferase [EC:2.4.1.17] | (RefSeq) UDP-glycosyltransferase 76G1-like (A)	UDP-glycosyltransferase 76B1 {ECO:0000305}; EC=2.4.1.- {ECO:0000305} (At3g11340)	-1.264	-1.334
Cs8g18310	K00699 glucuronosyltransferase [EC:2.4.1.17] | (RefSeq) UDP-glycosyltransferase 76G1-like (A)	UDP-glycosyltransferase 76B1 {ECO:0000305}; EC=2.4.1.- {ECO:0000305} (At3g11340)	-1.040	-3.150
Cs9g04630	K05658 ATP-binding cassette, subfamily B (MDR/TAP), member 1 [EC:7.6.2.2] | (RefSeq) uncharacterized protein LOC111781709 (A)	Protein SRG1; AtSRG1; Protein SENESCENCE-RELATED GENE 1 (At3g21420)	1.345	1.562
Cs9g05270	K01240 uridine nucleosidase [EC:3.2.2.3] | (RefSeq) uridine nucleosidase 1-like (A)	Probable uridine nucleosidase 2; EC=3.2.2.3; Uridine ribohydrolase 2 (At5g18860)		1.915
Cs9g09290	K05658 ATP-binding cassette, subfamily B (MDR/TAP), member 1 [EC:7.6.2.2] | (RefSeq) uncharacterized protein LOC111781709 (A)	Protein SRG1; AtSRG1; Protein SENESCENCE-RELATED GENE 1 (At1g17020)	2.091	

Cs9g13580	K01365 cathepsin L [EC:3.4.22.15] | (RefSeq) zingipain-2-like (A)	Senescence-specific cysteine protease SAG12 {ECO:0000305}; EC=3.4.22.- {ECO:0000305}; Cysteine proteinase SAG12 {ECO:0000305}; Protein SENESCENCE-ASSOCIATED GENE 12 {ECO:0000303|Ref.6}; Flags: Precursor (At3g49340)	7.122	
Cs9g16150		CDGSH iron-sulfur domain-containing protein NEET; At-NEET (At5g51720)	-1.199	-1.008
orange1.1t00587	K00521 ferric-chelate reductase [EC:1.16.1.7] | (RefSeq) ferric reduction oxidase 7, chloroplastic-like (A)	NAC domain-containing protein 53 {ECO:0000303|PubMed:15029955}; ANAC053 {ECO:0000303|PubMed:15029955}; Protein ANTHER INDEHISCENCE FACTOR {ECO:0000303|PubMed:24323506}; Protein NTM1-like 4 {ECO:0000303|PubMed:17158162} (At3g10500)	1.675	
orange1.1t00806	K11000 callose synthase [EC:2.4.1.-] | (RefSeq) callose synthase 12 (A)	Callose synthase 12; EC=2.4.1.34; 1,3-beta-glucan synthase; Protein GLUCAN SYNTHASE-LIKE 5; Protein POWDERY MILDEW RESISTANT 4 (At4g03550)		1.145
orange1.1t01032	K06892 feruloyl-CoA ortho-hydroxylase [EC:1.14.11.-] | (RefSeq) 2-oxoglutarate-iron(II)-dependent oxygenase (A)	Protein DMR6-LIKE OXYGENASE 1 {ECO:0000303|PubMed:25376907}; EC=1.14.11.- {ECO:0000255|PROSITE-ProRule:PRU00805}; 2-oxoglutarate (2OG)-Fe(II) oxygenase-like protein DLO1 {ECO:0000303|PubMed:25376907}; Protein SENESCENCE-ASSOCIATED GENE 108 {ECO:0000303|PubMed:23959884}; Salicylate 3-hydroxylase DLO1 {ECO:0000303|PubMed:23959884}; S3H DLO1 {ECO:0000303|PubMed:23959884}; SA 3-hydroxylase DLO1 {ECO:0000303|PubMed:23959884}; Salicylic acid 3-hydroxylase DLO1 {ECO:0000303|PubMed:23959884}; EC=1.14.13.- {ECO:0000269|PubMed:23959884} (At2g36690)		1.348
orange1.1t02662	K20604 mitogen-activated protein kinase kinase 9 [EC:2.7.12.2] | (RefSeq) mitogen-activated protein kinase kinase 9 (A)	Mitogen-activated protein kinase kinase 9; AtMKK9; MAP kinase kinase 9; EC=2.7.12.2 (At1g73500)	-1.280	-1.159
