# Supplementary material for: Mechanisms by Which Increased pH Ameliorates Copper Excess in Citrus sinensis Roots: Insight from a Combined Analysis of Physiology, Transcriptome, and Metabolome
Source: Plants (Basel). 2024 Oct 31;13(21):3054. doi: 10.3390/plants13213054 (PMC11548300; doi:10.3390/plants13213054)
Supplement: Supplementary file 1 [file plants-13-03054-s001.zip › Table S14SecondRevision.pdf]

Table S14. DEGs and DAMs related to carbohydrate and energy metabolisms in P3CR vs. P3R and/or P5CR vs. P5R

**Accession No.**	**KEGG**	**Swissprot (KOG)**	**Log2(FC)**	
			P3CR vs. P3R	P5CR vs. P5R
Starch and sucrose metabolism [ko00500; P = 0.5074 (P3CR vs. P3R) and 0.3170 (P5CR vs. P5R)]				
Cs1g01210	K01210 glucan 1,3-beta-glucosidase [EC:3.2.1.58] | (RefSeq) probable glucan 1,3-beta-glucosidase A (A)	(YOR190w)		1.066
Cs1g01330	K01087 trehalose 6-phosphate phosphatase [EC:3.1.3.12] | (RefSeq) probable trehalose-phosphate phosphatase J (A)	Probable trehalose-phosphate phosphatase J; AtTPPJ; EC=3.1.3.12; Trehalose 6-phosphate phosphatase (At5g65140)	-1.651	-1.272
Cs1g06790	K05350 beta-glucosidase [EC:3.2.1.21] | (RefSeq) beta-glucosidase 18-like (A)	Beta-glucosidase 18; Os4bglu18; EC=3.2.1.21; Flags: Precursor (At4g21760)		1.146
Cs1g11480	K01193 beta-fructofuranosidase [EC:3.2.1.26] | (RefSeq) LOC109747879; beta-fructofuranosidase, insoluble isoenzyme 3-like (A)	Putative UPF0481 protein At3g02645 (At3g02650_1)	2.078	
Cs1g15840	K00844 hexokinase [EC:2.7.1.1] | (RefSeq) hexokinase-1-like (A)	Hexokinase-1; EC=2.7.1.1; NtHxK1 (At4g29130)		-1.611
Cs1g18220	K01193 beta-fructofuranosidase [EC:3.2.1.26] | (RefSeq) beta-fructofuranosidase, insoluble isoenzyme CWINV1 (A)	Beta-fructofuranosidase, insoluble isoenzyme CWINV1; EC=3.2.1.26; Cell wall beta-fructosidase 1; AtbetaFRUCT1; Cell wall invertase 1; AtcwINV1; Sucrose hydrolase 1; Flags: Precursor (At3g13790)		-1.274
Cs1g18240	K01193 beta-fructofuranosidase [EC:3.2.1.26] | (RefSeq) beta-fructofuranosidase, insoluble isoenzyme CWINV1-like (A)	Fructan 6-exohydrolase {ECO:0000312|EMBL:CAD48404.1}; EC=3.2.1.154; Flags: Precursor (At3g13790)	1.373	
Cs1g21260	K01179 endoglucanase [EC:3.2.1.4] | (RefSeq) endoglucanase 2-like (A)	Endoglucanase 24; EC=3.2.1.4; Endo-1,4-beta glucanase 24; Flags: Precursor (At1g19940)		-2.171
Cs2g01100	K01193 beta-fructofuranosidase [EC:3.2.1.26] | (RefSeq) LOC109747879; beta-fructofuranosidase, insoluble isoenzyme 3-like (A)	UPF0481 protein At3g47200 (At4g31980_2)	2.886	
Cs2g01120	K01193 beta-fructofuranosidase [EC:3.2.1.26] | (RefSeq) LOC109747879; beta-fructofuranosidase, insoluble isoenzyme 3-like (A)	UPF0481 protein At3g47200 (At4g31980_2)	2.275	
Cs2g01150	K01193 beta-fructofuranosidase [EC:3.2.1.26] | (RefSeq) LOC109747879; beta-fructofuranosidase, insoluble isoenzyme 3-like (A)	UPF0481 protein At3g47200 (At4g31980_2)	2.645	2.218

Cs2g03340	K19891 glucan endo-1,3-beta-glucosidase 1/2/3 [EC:3.2.1.39] | (RefSeq) hypothetical protein (A)	Glucan endo-1,3-beta-glucosidase 11; EC=3.2.1.39; (1->3)-beta-glucan endohydrolase 11; (1->3)-beta-glucanase 11; Beta-1,3-endoglucanase 11; Beta-1,3-glucanase 11; Flags: Precursor (At1g30080)	1.102	-1.069
Cs2g03760	K19891 glucan endo-1,3-beta-glucosidase 1/2/3 [EC:3.2.1.39] | (RefSeq) hypothetical protein (A)	Glucan endo-1,3-beta-D-glucosidase; EC=3.2.1.39; Major pollen allergen Ole e 9; AltName: Allergen=Ole e 9; Flags: Precursor (At1g78520)	5.025	
Cs2g09510	K19891 glucan endo-1,3-beta-glucosidase 1/2/3 [EC:3.2.1.39] | (RefSeq) hypothetical protein (A)	Glucan endo-1,3-beta-glucosidase 13; EC=3.2.1.39; (1->3)-beta-glucan endohydrolase 13; (1->3)-beta-glucanase 13; Beta-1,3-endoglucanase 13; Beta-1,3-glucanase 13; Flags: Precursor (At4g05430)		-2.165
Cs2g11050	K01187 alpha-glucosidase [EC:3.2.1.20] | (RefSeq) alpha-glucosidase (A)	Alpha-glucosidase; EC=3.2.1.20; Maltase; Flags: Precursor (At5g11720)	1.942	
Cs2g12180	K01179 endoglucanase [EC:3.2.1.4] | (RefSeq) endoglucanase 12 (A)	Endoglucanase 12; EC=3.2.1.4; Endo-1,4-beta glucanase 12; OsGLU3 (At1g65610)		1.162
Cs2g17090	K01179 endoglucanase [EC:3.2.1.4] | (RefSeq) endoglucanase 8-like (A)	Endoglucanase 8; EC=3.2.1.4; Cellulase 1; AtCEL1; Endo-1,4-beta glucanase 8; Flags: Precursor (At1g70710)	-1.018	
Cs2g21660	K19891 glucan endo-1,3-beta-glucosidase 1/2/3 [EC:3.2.1.39] | (RefSeq) hypothetical protein (A)	Glucan endo-1,3-beta-glucosidase 11; EC=3.2.1.39; (1->3)-beta-glucan endohydrolase 11; (1->3)-beta-glucanase 11; Beta-1,3-endoglucanase 11; Beta-1,3-glucanase 11; Flags: Precursor (At2g05790)		-2.259
Cs2g22040	K01177 beta-amylase [EC:3.2.1.2] | (RefSeq) hypothetical protein (A)	Beta-amylase; EC=3.2.1.2; 1,4-alpha-D-glucan maltohydrolase (At4g15210)	1.575	1.347
Cs2g28150	K01087 trehalose 6-phosphate phosphatase [EC:3.1.3.12] | (RefSeq) trehalose-phosphate phosphatase A-like (A)	Protein KINESIN LIGHT CHAIN-RELATED 2 {ECO:0000303|PubMed:23204523}; Kinesin light chain-like protein 1 {ECO:0000303|Ref.1}; AtKLC1 {ECO:0000303|Ref.1} (At3g27960)		1.090
Cs3g07670	K01193 beta-fructofuranosidase [EC:3.2.1.26] | (RefSeq) LOC109747879; beta-fructofuranosidase, insoluble isoenzyme 3-like (A)	UPF0481 protein At3g47200 (At2g36430)		1.219
Cs3g09730	K01187 alpha-glucosidase [EC:3.2.1.20] | (RAP-DB) Os07g0421300; Similar to Alpha glucosidase-like protein. (A)	Probable glucan 1,3-alpha-glucosidase; EC=3.2.1.84; Glucosidase II subunit alpha; Protein PRIORITY IN SWEET LIFE 5; Protein RADIAL SWELLING 3; Flags: Precursor (At3g23640)		1.120
Cs3g09750	K01187 alpha-glucosidase [EC:3.2.1.20] | (RefSeq)			1.470

	alpha-glucosidase 2-like (A)			
Cs3g09800	K16055 trehalose 6-phosphate synthase/phosphatase [EC:2.4.1.15 3.1.3.12] | (RefSeq) alpha,alpha-trehalose-phosphate synthase [UDP-forming] 5 (A)	Alpha,alpha-trehalose-phosphate synthase [UDP-forming] 5; EC=2.4.1.15; Trehalose-6-phosphate synthase 5; AtTPS5 (At4g17770)		1.137
Cs3g18450	K01087 trehalose 6-phosphate phosphatase [EC:3.1.3.12] | (RefSeq) probable trehalose-phosphate phosphatase D (A)	Probable trehalose-phosphate phosphatase D; AtTPPD; EC=3.1.3.12; Trehalose 6-phosphate phosphatase (At1g35910)		1.440
Cs3g18560	K01210 glucan 1,3-beta-glucosidase [EC:3.2.1.58] | (RefSeq) probable glucan 1,3-beta-glucosidase A (A)	(YOR190w)		-1.860
Cs3g18600	K01210 glucan 1,3-beta-glucosidase [EC:3.2.1.58] | (RefSeq) probable glucan 1,3-beta-glucosidase A (A)	(YOR190w)		-1.325
Cs3g23560	K01176 alpha-amylase [EC:3.2.1.1] | (RefSeq) hypothetical protein (A)	Alpha-amylase; EC=3.2.1.1 {ECO:0000250|UniProtKB:P00693}; 1,4-alpha-D-glucan glucanohydrolase; Flags: Precursor (At4g25000)	1.160	
Cs3g27220	K00700 1,4-alpha-glucan branching enzyme [EC:2.4.1.18] | (RefSeq) 1,4-alpha-glucan-branching enzyme 1, chloroplastic/amyloplastic-like (A)	1,4-alpha-glucan-branching enzyme 1, chloroplastic/amyloplastic; EC=2.4.1.18; Starch branching enzyme I; Flags: Precursor; Fragment (At5g03650)	1.861	
Cs3g27500	K19891 glucan endo-1,3-beta-glucosidase 1/2/3 [EC:3.2.1.39] | (RefSeq) glucan endo-1,3-beta-glucosidase 3-like (A)	Glucan endo-1,3-beta-glucosidase 12; EC=3.2.1.39; (1->3)-beta-glucan endohydrolase 12; (1->3)-beta-glucanase 12; Beta-1,3-endoglucanase 12; Beta-1,3-glucanase 12; Flags: Precursor (At4g29360)		1.498
Cs4g04430	K01214 isoamylase [EC:3.2.1.68] | (RefSeq) isoamylase 1, chloroplastic (A)	Isoamylase 1, chloroplastic; AtISA1; EC=3.2.1.68; Flags: Precursor (At2g39930)		1.220
Cs4g08530	K19892 glucan endo-1,3-beta-glucosidase 4 [EC:3.2.1.39] | (RefSeq) glucan endo-1,3-beta-glucosidase 4-like (A)	Glucan endo-1,3-beta-glucosidase 7; EC=3.2.1.39; (1->3)-beta-glucan endohydrolase 7; (1->3)-beta-glucanase 7; Beta-1,3-endoglucanase 7; Beta-1,3-glucanase 7; Flags: Precursor (At5g53600)		1.964
Cs4g08550	K19892 glucan endo-1,3-beta-glucosidase 4 [EC:3.2.1.39] | (RefSeq) glucan endo-1,3-beta-glucosidase 4-like (A)	Major pollen allergen Ole e 10; AltName: Allergen=Ole e 10; Flags: Precursor (At1g66870)	-1.291	-5.245
Cs4g09780	K19891 glucan endo-1,3-beta-glucosidase 1/2/3 [EC:3.2.1.39] | (RefSeq) hypothetical protein (A)	Glucan endo-1,3-beta-glucosidase 13; EC=3.2.1.39; (1->3)-beta-glucan endohydrolase 13; (1->3)-beta-glucanase 13; Beta-1,3-endoglucanase 13; Beta-1,3-glucanase 13; Flags: Precursor (At5g55180)		-2.309
Cs4g11970	K01188 beta-glucosidase [EC:3.2.1.21] | (RefSeq) putative beta-glucosidase 41 (A)	Putative beta-glucosidase 41; AtBGLU41; EC=3.2.1.21; Flags: Precursor (At5g54570)	2.732	3.222

Cs4g18340	K01193 beta-fructofuranosidase [EC:3.2.1.26] | (RefSeq) beta-fructofuranosidase, insoluble isoenzyme CWINV1 (A)	Beta-fructofuranosidase, insoluble isoenzyme CWINV1; EC=3.2.1.26; Cell wall beta-fructosidase 1; AtbetaFRUCT1; Cell wall invertase 1; AtcwINV1; Sucrose hydrolase 1; Flags: Precursor (At3g13790)		3.456
Cs5g01400	K01179 endoglucanase [EC:3.2.1.4] | (RefSeq) endoglucanase CX (A)	Endoglucanase 1; EC=3.2.1.4; Abscission cellulase 1; Endo-1,4-beta-glucanase 1; Flags: Precursor (At1g02800)		3.825
Cs5g08850	K00750 glycogenin [EC:2.4.1.186] | (RefSeq) putative glucuronosyltransferase PGSIP8 (A)	Putative glucuronosyltransferase PGSIP7; EC=2.4.1.-; Glycogenin-like protein 7; Plant glycogenin-like starch initiation protein 7 (At4g16600)		1.243
Cs5g19060	K00696 sucrose-phosphate synthase [EC:2.4.1.14] | (RefSeq) probable sucrose-phosphate synthase 4 (A)	Probable sucrose-phosphate synthase 4; EC=2.4.1.14; Sucrose phosphate synthase 4F; AtSPS4F; UDP-glucose-fructose-phosphate glucosyltransferase (At4g10120)		1.416
Cs5g22270	K01193 beta-fructofuranosidase [EC:3.2.1.26] | (RefSeq) LOC109747879; beta-fructofuranosidase, insoluble isoenzyme 3-like (A)	UPF0481 protein At3g47200 (At4g31980_2)		1.063
Cs5g22910	K00847 fructokinase [EC:2.7.1.4] | (RefSeq) probable fructokinase-4 (A)	Probable fructokinase-4; EC=2.7.1.4 (At3g59480)	-1.093	
Cs5g26860	K05349 beta-glucosidase [EC:3.2.1.21] | (RefSeq) beta-glucosidase BoGH3B-like (A)	Beta-D-xylosidase 3; AtBXL3; EC=3.2.1.-; Alpha-L-arabinofuranosidase; EC=3.2.1.55; Flags: Precursor (At5g20950)	2.891	2.238
Cs5g33460	K01179 endoglucanase [EC:3.2.1.4] | (RefSeq) endoglucanase 17 (A)	Endoglucanase 17; EC=3.2.1.4; Endo-1,4-beta glucanase 17; Flags: Precursor (At4g02290)		-1.318
Cs5g33470	K00695 sucrose synthase [EC:2.4.1.13] | (RefSeq) sucrose synthase 2 (A)	Sucrose synthase 2; EC=2.4.1.13; Sucrose-UDP glucosyltransferase 2 (At4g02280)		1.247
Cs6g16770	K19893 glucan endo-1,3-beta-glucosidase 5/6 [EC:3.2.1.39] | (RefSeq) glucan endo-1,3-beta-glucosidase 6-like (A)	Glucan endo-1,3-beta-glucosidase; EC=3.2.1.39; (1->3)-beta-glucan endohydrolase; (1->3)-beta-glucanase; Beta-1,3-endoglucanase; Flags: Precursor (At3g57270)	3.620	1.955
Cs6g17850	K05350 beta-glucosidase [EC:3.2.1.21] | (RefSeq) hypothetical protein (A)	Beta-glucosidase 44; AtBGLU44; EC=3.2.1.21; Flags: Precursor (At3g18080)		-2.746
Cs6g21370	K00703 starch synthase [EC:2.4.1.21] | (RefSeq) granule-bound starch synthase 2, chloroplastic/amyloplastic (A)	Starch synthase 2, chloroplastic/amyloplastic; AtSS2; EC=2.4.1.21; Soluble starch synthase II; Flags: Precursor (At3g01180)	1.011	
Cs7g01380	K01188 beta-glucosidase [EC:3.2.1.21] | (RefSeq) beta-glucosidase 17-like (A)	Beta-glucosidase 17; AtBGLU17; EC=3.2.1.21; Flags: Precursor (At2g44480)	1.090	1.679
Cs7g05690	K00696 sucrose-phosphate synthase [EC:2.4.1.14] | (RefSeq)	Probable sucrose-phosphate synthase 1; EC=2.4.1.14;	1.268	

	probable sucrose-phosphate synthase 1 (A)	UDP-glucose-fructose-phosphate glucosyltransferase 1 (At5g11110)		
Cs7g08390	K16055 trehalose 6-phosphate synthase/phosphatase [EC:2.4.1.15 3.1.3.12] | (RefSeq) hypothetical protein (A)	Probable alpha,alpha-trehalose-phosphate synthase [UDP-forming] 9; EC=2.4.1.15; Trehalose-6-phosphate synthase 9; AtTPS9 (At1g23870)		-1.922
Cs7g09680	K01179 endoglucanase [EC:3.2.1.4] | (RefSeq) uncharacterized protein LOC104710406 (A)	(At1g13130)	2.183	1.866
Cs7g13990	K01179 endoglucanase [EC:3.2.1.4] | (RefSeq) endoglucanase 5 (A)	Endoglucanase 5; EC=3.2.1.4; Endo-1,4-beta glucanase 5; Flags: Precursor (At1g48930)		1.257
Cs7g23850	K07024 sucrose-6-phosphatase [EC:3.1.3.24] | (RefSeq) hypothetical protein (A)	Sucrose-phosphatase 2; NtSPP2; EC=3.1.3.24 (At1g51420)		1.006
Cs8g11370	K01193 beta-fructofuranosidase [EC:3.2.1.26] | (RefSeq) LOC109747879; beta-fructofuranosidase, insoluble isoenzyme 3-like (A)	UPF0481 protein At3g47200 (At4g31980_2)	2.993	2.516
Cs8g11500	K01193 beta-fructofuranosidase [EC:3.2.1.26] | (RefSeq) LOC109747879; beta-fructofuranosidase, insoluble isoenzyme 3-like (A)	UPF0481 protein At3g47200 (At4g31980_2)	1.141	
Cs8g13800	K05349 beta-glucosidase [EC:3.2.1.21] | (RefSeq) beta-glucosidase BoGH3B-like (A)	Beta-xylosidase/alpha-L-arabinofuranosidase 2 {ECO:0000303|PubMed:17615411, ECO:0000312|EMBL:ABQ45228.1}; Xylan 1,4-beta-xylosidase/Alpha-L-arabinofuranosidase 2; MsXyl2 {ECO:0000303|PubMed:17615411}; Includes: Beta-xylosidase; EC=3.2.1.37; 1,4-beta-D-xylan xylohydrolase {ECO:0000250|UniProtKB:P48792}; Xylan 1,4-beta-xylosidase; Includes: Alpha-L-arabinofuranosidase; Arabinosidase {ECO:0000250|UniProtKB:P48792}; EC=3.2.1.55; Flags: Precursor (At5g20950)		-1.094
Cs8g19800	K00688 glycogen phosphorylase [EC:2.4.1.1] | (RefSeq) hypothetical protein (A)	Alpha-glucan phosphorylase 2, cytosolic; AtPHS2; EC=2.4.1.1; Alpha-glucan phosphorylase, H isozyme; Starch phosphorylase H (At3g46970)		1.288
Cs9g04980	K01177 beta-amylase [EC:3.2.1.2] | (RefSeq) inactive beta-amylase 9-like isoform X1 (A)	Inactive beta-amylase 9; 1,4-alpha-D-glucan maltohydrolase; Inactive beta-amylase 3 (At5g18670)		-1.594
Cs9g14300	K16055 trehalose 6-phosphate synthase/phosphatase [EC:2.4.1.15 3.1.3.12] | (RefSeq) alpha,alpha-trehalose-phosphate synthase	Alpha,alpha-trehalose-phosphate synthase [UDP-forming] 6; EC=2.4.1.15; Trehalose-6-phosphate synthase 6; AtTPS6		-1.578

	[UDP-forming] 6 (A)	(At1g68020)		
Cs9g14430	K01179 endoglucanase [EC:3.2.1.4] | (RefSeq) endoglucanase 4-like (A)	Endoglucanase 4; EC=3.2.1.4; Endo-1,4-beta glucanase 4; Flags: Precursor (At1g23210)	-1.165	
Cs9g14590	K01193 beta-fructofuranosidase [EC:3.2.1.26] | (RefSeq) Cs-bFruct2; beta-fructofuranosidase, soluble isoenzyme I-like (A)	Beta-fructofuranosidase, soluble isoenzyme I; EC=3.2.1.26; Invertase; Saccharase; Sucrose hydrolase; Flags: Precursor (At1g12240)		-2.684
Cs9g14600	K01087 trehalose 6-phosphate phosphatase [EC:3.1.3.12] | (RefSeq) trehalose-phosphate phosphatase A (A)	Trehalose-phosphate phosphatase A; AtTPPA; EC=3.1.3.12; Trehalose 6-phosphate phosphatase (At5g51460)		1.012
Cs9g16550	K00847 fructokinase [EC:2.7.1.4] | (RefSeq) hypothetical protein (A)	Probable fructokinase-7; EC=2.7.1.4 (At5g51830)	-1.148	
Cs9g18290	K01087 trehalose 6-phosphate phosphatase [EC:3.1.3.12] | (RefSeq) probable trehalose-phosphate phosphatase C (A)	Trehalose 6-phosphate phosphatase RA3 {ECO:0000305}; EC=3.1.3.12 {ECO:0000269|PubMed:16688177}; Protein RAMOSA 3 {ECO:0000303|PubMed:16688177} (At1g78090)	1.386	1.065
novel.1919	K05349 beta-glucosidase [EC:3.2.1.21] | (RefSeq) lysosomal beta glucosidase-like (A)	POLR2_ARATH Retrovirus-related Pol polyprotein from transposon RE2; Retro element 2 {ECO:0000303|PubMed:10689195}; AtRE2 {ECO:0000303|PubMed:10689195}; Includes: Protease RE2; EC=3.4.23.-; Includes: Reverse transcriptase RE2; EC=2.7.7.49; Includes: Endonuclease RE2 (At4g16020)	-1.420	
novel.2027	K19891 glucan endo-1,3-beta-glucosidase 1/2/3 [EC:3.2.1.39] | (RefSeq) glucan endo-1,3-beta-glucosidase 3-like (A)	E1310_ARATH Glucan endo-1,3-beta-glucosidase 10; EC=3.2.1.39; (1->3)-beta-glucan endohydrolase 10; (1->3)-beta-glucanase 10; Beta-1,3-endoglucanase 10; Beta-1,3-glucanase 10; Putative plasmodesmal associated protein {ECO:0000303|PubMed:17270015}; AtBG_ppap {ECO:0000303|PubMed:17270015}; Flags: Precursor (At2g05790)		-1.014
novel.301	K01179 endoglucanase [EC:3.2.1.4] | (RefSeq) endoglucanase 12 (A)	GUN7_ARATH Endoglucanase 7; EC=3.2.1.4; Endo-1,4-beta glucanase 7 (At1g65610)		1.225
orange1.1t00277	K00847 fructokinase [EC:2.7.1.4] | (RefSeq) hypothetical protein (A)	Probable fructokinase-7; EC=2.7.1.4 (At5g51830)	-1.072	
orange1.1t00484	K01513 ectonucleotide pyrophosphatase/phosphodiesterase family member 1/3 [EC:3.1.4.1 3.6.1.9] | (RefSeq) uncharacterized protein LOC109769794 (A)	(At2g46420)		-1.935
orange1.1t00633	K19891 glucan endo-1,3-beta-glucosidase 1/2/3 [EC:3.2.1.39] | (RefSeq) glucan endo-1,3-beta-glucosidase 3-like (A)	Glucan endo-1,3-beta-glucosidase, basic isoform; EC=3.2.1.39; (1->3)-beta-glucan endohydrolase; (1->3)-beta-glucanase;		-3.217

		Beta-1,3-endoglucanase; PpGns1; Flags: Precursor (At3g57270)		
orange1.1t00634	K19891 glucan endo-1,3-beta-glucosidase 1/2/3 [EC:3.2.1.39] | (RefSeq) glucan endo-1,3-beta-glucosidase 3-like (A)	Glucan endo-1,3-beta-glucosidase, basic isoform; EC=3.2.1.39; (1->3)-beta-glucan endohydrolase; (1->3)-beta-glucanase; Beta-1,3-endoglucanase; PpGns1; Flags: Precursor (At3g57270)	1.969	
orange1.1t00635	K19893 glucan endo-1,3-beta-glucosidase 5/6 [EC:3.2.1.39] | (RefSeq) glucan endo-1,3-beta-glucosidase 6-like isoform X1 (A)	Glucan endo-1,3-beta-glucosidase; EC=3.2.1.39; (1->3)-beta-glucan endohydrolase; (1->3)-beta-glucanase; Beta-1,3-endoglucanase; Flags: Precursor (At3g57270)	3.569	
orange1.1t00636	K19891 glucan endo-1,3-beta-glucosidase 1/2/3 [EC:3.2.1.39] | (RefSeq) glucan endo-1,3-beta-glucosidase 3-like (A)	Glucan endo-1,3-beta-glucosidase; EC=3.2.1.39; (1->3)-beta-glucan endohydrolase; (1->3)-beta-glucanase; Beta-1,3-endoglucanase; Flags: Precursor (At3g57270)	4.225	
orange1.1t00643	K19891 glucan endo-1,3-beta-glucosidase 1/2/3 [EC:3.2.1.39] | (RefSeq) glucan endo-1,3-beta-glucosidase 3-like (A)	Glucan endo-1,3-beta-glucosidase, basic isoform; EC=3.2.1.39; (1->3)-beta-glucan endohydrolase; (1->3)-beta-glucanase; Beta-1,3-endoglucanase; PpGns1; Flags: Precursor (At3g57270)		-1.670
orange1.1t01105	K01187 alpha-glucosidase [EC:3.2.1.20] | (RefSeq) alpha-glucosidase 2-like (A)	Probable glucan 1,3-alpha-glucosidase; EC=3.2.1.84; Glucosidase II subunit alpha; Protein PRIORITY IN SWEET LIFE 5; Protein RADIAL SWELLING 3; Flags: Precursor (At3g23640)		1.095
orange1.1t02061	K01193 beta-fructofuranosidase [EC:3.2.1.26] | (RefSeq) LOC109747879; beta-fructofuranosidase, insoluble isoenzyme 3-like (A)	UPF0481 protein At3g47200 (At4g31980_2)		1.416
Citrate cycle (TCA cycle) [ko00020; P = 0.6034 (P3CR vs. P3R) and 0.9416 (P5CR vs. P5R]				
Cs1g20920	K01610 phosphoenolpyruvate carboxykinase (ATP) [EC:4.1.1.49] | (RefSeq) phosphoenolpyruvate carboxykinase [ATP]-like (A)	Phosphoenolpyruvate carboxykinase (ATP); PEP carboxykinase; PEPCK; EC=4.1.1.49 (At4g37870)		1.085
Cs1g25560	K00025 malate dehydrogenase [EC:1.1.1.37] | (RefSeq) malate dehydrogenase, cytoplasmic-like (A)	Oligopeptide transporter 7; AtOPT7 (At4g10770)	-1.049	
Cs3g16700	K01610 phosphoenolpyruvate carboxykinase (ATP) [EC:4.1.1.49] | (RefSeq) phosphoenolpyruvate carboxykinase [ATP]-like (A)	Phosphoenolpyruvate carboxykinase (ATP); PEP carboxykinase; PEPCK; EC=4.1.1.49 (At4g37870)		2.281
Cs3g16870	K00025 malate dehydrogenase [EC:1.1.1.37] | (RefSeq) malate dehydrogenase, cytoplasmic-like (A)	Oligopeptide transporter 4; AtOPT4 (At5g64410)	1.016	
Cs4g08360	K00025 malate dehydrogenase [EC:1.1.1.37] | (RefSeq) malate dehydrogenase, cytoplasmic-like (A)	Oligopeptide transporter 1; AtOPT1 (At5g55930)	1.141	
Cs4g08370	K00025 malate dehydrogenase [EC:1.1.1.37] | (RefSeq) malate dehydrogenase, cytoplasmic-like (A)	Oligopeptide transporter 1; AtOPT1 (At5g55930)		-1.145

Cs4g08380	K00025 malate dehydrogenase [EC:1.1.1.37] | (RefSeq) malate dehydrogenase, cytoplasmic-like (A)	Oligopeptide transporter 1; AtOPT1 (At5g55930)		-1.837
Cs8g02970	K00025 malate dehydrogenase [EC:1.1.1.37] | (RefSeq) malate dehydrogenase, cytoplasmic-like (A)	Oligopeptide transporter 4; AtOPT4 (At5g64410)	-1.428	
novel.2012	K00234 succinate dehydrogenase (ubiquinone) flavoprotein subunit [EC:1.3.5.1] | (RefSeq) succinate dehydrogenase [ubiquinone] flavoprotein subunit, mitochondrial-like (A)	(At2g18950)	1.093	1.829
Glycolysis / Gluconeogenesis [ko00010; P = 0.8110 (P3CR vs. P3R) and 0.9620 (P5CR vs. P5R)]				
Cs1g15840	K00844 hexokinase [EC:2.7.1.1] | (RefSeq) hexokinase-1-like (A)	Hexokinase-1; EC=2.7.1.1; NtHxK1 (At4g29130)		-1.611
Cs1g15970	K00850 6-phosphofructokinase 1 [EC:2.7.1.11] | (RefSeq) ATP-dependent 6-phosphofructokinase 3-like (A)	ATP-dependent 6-phosphofructokinase 3 {ECO:0000255|HAMAP-Rule:MF_03186}; ATP-PFK 3 {ECO:0000255|HAMAP-Rule:MF_03186}; Phosphofructokinase 3 {ECO:0000255|HAMAP-Rule:MF_03186}; EC=2.7.1.11 {ECO:0000255|HAMAP-Rule:MF_03186}; Phosphohexokinase 3 {ECO:0000255|HAMAP-Rule:MF_03186} (At4g29220)	1.064	
Cs1g20920	K01610 phosphoenolpyruvate carboxykinase (ATP) [EC:4.1.1.49] | (RefSeq) phosphoenolpyruvate carboxykinase [ATP]-like (A)	Phosphoenolpyruvate carboxykinase (ATP); PEP carboxykinase; PEPCK; EC=4.1.1.49 (At4g37870)		1.085
Cs1g24850	K00128 aldehyde dehydrogenase (NAD+) [EC:1.2.1.3] | (RefSeq) aldehyde dehydrogenase family 3 member F1 (A)	Aldehyde dehydrogenase family 3 member F1; EC=1.2.1.3 (At4g36250)	1.544	
Cs2g05050	K01568 pyruvate decarboxylase [EC:4.1.1.1] | (RefSeq) hypothetical protein (A)	Pyruvate decarboxylase 1; PDC; EC=4.1.1.1 (At5g54960)	-1.150	
Cs2g14890	K01689 enolase [EC:4.2.1.11] | (RefSeq) enolase 1, chloroplastic isoform X1 (A)	(At5g47920)		1.457
Cs2g29670	K00895 diphosphate-dependent phosphofructokinase [EC:2.7.1.90] | (RefSeq) pyrophosphatefructose 6-phosphate 1-phosphotransferase subunit alpha-like (A)	(At1g50570_2)	1.233	1.301
Cs3g14390	K00128 aldehyde dehydrogenase (NAD+) [EC:1.2.1.3] | (RefSeq) aldehyde dehydrogenase 5, mitochondrial-like (A)	Translationally-controlled tumor protein homolog; TCTP (At3g16640)		-2.145
Cs3g16700	K01610 phosphoenolpyruvate carboxykinase (ATP) [EC:4.1.1.49] | (RefSeq) phosphoenolpyruvate carboxykinase [ATP]-like (A)	Phosphoenolpyruvate carboxykinase (ATP); PEP carboxykinase; PEPCK; EC=4.1.1.49 (At4g37870)		2.281
Cs3g17650	K00850 6-phosphofructokinase 1 [EC:2.7.1.11] | (RefSeq) ATP-dependent 6-phosphofructokinase 6-like (A)	Purine permease 1 {ECO:0000303|PubMed:10662864}; AtPUP1 {ECO:0000303|PubMed:10662864} (At1g28230)		-1.500

Cs3g17940	K00121 S-(hydroxymethyl)glutathione dehydrogenase / alcohol dehydrogenase [EC:1.1.1.284 1.1.1.1] | (RefSeq) alcohol dehydrogenase-like 2 (A)	Alcohol dehydrogenase-like 2; EC=1.1.1.1 {ECO:0000250|UniProtKB:P06525} (At1g22440)		1.096
Cs3g20350	K01913 acetate-CoA ligase [EC:6.2.1.1] | (RefSeq) acetate/butyrateCoA ligase AAE7, peroxisomal-like isoform X1 (A)	Probable acyl-activating enzyme 6; EC=6.2.1.-; AMP-binding protein 6; AtAMPBP6 (At5g16340)		-1.085
Cs3g20800	K18857 alcohol dehydrogenase class-P [EC:1.1.1.1] | (RefSeq) alcohol dehydrogenase-like (A)	Alcohol dehydrogenase; EC=1.1.1.1 {ECO:0000250|UniProtKB:P06525} (At1g77120)	-1.541	
Cs3g20810	K18857 alcohol dehydrogenase class-P [EC:1.1.1.1] | (RefSeq) alcohol dehydrogenase (A)	Alcohol dehydrogenase; EC=1.1.1.1 {ECO:0000250|UniProtKB:P06525} (At1g77120)	-2.108	
Cs3g21280	K03841 fructose-1,6-bisphosphatase I [EC:3.1.3.11] | (RefSeq) fructose-1,6-bisphosphatase, cytosolic (A)	Fructose-1,6-bisphosphatase, cytosolic; FBPase; EC=3.1.3.11; CY-F1; D-fructose-1,6-bisphosphate 1-phosphohydrolase (At1g43670)		1.036
Cs4g13070	K00850 6-phosphofructokinase 1 [EC:2.7.1.11] | (RefSeq) ATP-dependent 6-phosphofructokinase 3-like (A)	ATP-dependent 6-phosphofructokinase 3 {ECO:0000255|HAMAP-Rule:MF_03186}; ATP-PFK 3 {ECO:0000255|HAMAP-Rule:MF_03186}; Phosphofructokinase 3 {ECO:0000255|HAMAP-Rule:MF_03186}; EC=2.7.1.11 {ECO:0000255|HAMAP-Rule:MF_03186}; Phosphohexokinase 3 {ECO:0000255|HAMAP-Rule:MF_03186} (At5g56630)	-1.258	
Cs4g14970	K01913 acetate-CoA ligase [EC:6.2.1.1] | (RefSeq) acetate/butyrateCoA ligase AAE7, peroxisomal-like (A)	Probable acyl-activating enzyme 6; EC=6.2.1.-; AMP-binding protein 6; AtAMPBP6 (At5g16340)		-1.048
Cs4g18980	K00850 6-phosphofructokinase 1 [EC:2.7.1.11] | (RefSeq) hypothetical protein (A)	ATP-dependent 6-phosphofructokinase 3 {ECO:0000255|HAMAP-Rule:MF_03186}; ATP-PFK 3 {ECO:0000255|HAMAP-Rule:MF_03186}; Phosphofructokinase 3 {ECO:0000255|HAMAP-Rule:MF_03186}; EC=2.7.1.11 {ECO:0000255|HAMAP-Rule:MF_03186}; Phosphohexokinase 3 {ECO:0000255|HAMAP-Rule:MF_03186} (At4g32840)	-1.602	-1.466
Cs6g08840	K01568 pyruvate decarboxylase [EC:4.1.1.1] | (RefSeq) hypothetical protein (A)	Pyruvate decarboxylase 1; AtPDC1; EC=4.1.1.1 (At4g33070)	-1.459	
Cs7g14420	K00121 S-(hydroxymethyl)glutathione dehydrogenase / alcohol dehydrogenase [EC:1.1.1.284 1.1.1.1] | (RefSeq) alcohol dehydrogenase-like 2 (A)	Alcohol dehydrogenase-like 2; EC=1.1.1.1 {ECO:0000250|UniProtKB:P06525} (At1g22440)		1.229
Cs8g08710	K01623 fructose-bisphosphate aldolase, class I [EC:4.1.2.13] | (RefSeq) fructose-bisphosphate aldolase 1, chloroplastic (A)	Fructose-bisphosphate aldolase 2, chloroplastic {ECO:0000305}; AtFBA2 {ECO:0000303|PubMed:22561114}; EC=4.1.2.13 {ECO:0000305}; Flags: Precursor (At2g01140)	1.927	

Cs9g02540	K01913 acetate-CoA ligase [EC:6.2.1.1] | (RefSeq) acetate/butyrateCoA ligase AAE7, peroxisomal-like (A)	Acetate/butyrateCoA ligase AAE7, peroxisomal; EC=6.2.1.1; EC=6.2.1.2; AMP-binding protein 7; AtAMPBP7; Acetyl-CoA synthetase; Acyl-activating enzyme 7; Butyryl-CoA synthetase; Protein ACETATE NON-UTILIZING 1 (At3g16910)		-1.628
Cs9g02550	K01913 acetate-CoA ligase [EC:6.2.1.1] | (RefSeq) hypothetical protein (A)	Acetate/butyrateCoA ligase AAE7, peroxisomal; EC=6.2.1.1; EC=6.2.1.2; AMP-binding protein 7; AtAMPBP7; Acetyl-CoA synthetase; Acyl-activating enzyme 7; Butyryl-CoA synthetase; Protein ACETATE NON-UTILIZING 1 (At3g16910)	1.479	
Cs9g17270	K01623 fructose-bisphosphate aldolase, class I [EC:4.1.2.13] | (RefSeq) fructose-bisphosphate aldolase 6, cytosolic-like (A)	Heavy metal-associated isoprenylated plant protein 39 {ECO:0000303|PubMed:21072340, ECO:0000303|PubMed:23368984}; AtHIP39 {ECO:0000303|PubMed:21072340, ECO:0000303|PubMed:23368984}; Flags: Precursor (At1g01490)		-1.134
Cs9g18120	K00873 pyruvate kinase [EC:2.7.1.40] | (RefSeq) hypothetical protein (A)	Plastidial pyruvate kinase 4, chloroplastic; PKp4; EC=2.7.1.40; Flags: Precursor (At3g49160)	-1.526	-1.586
orange1.1t04629	K00121 S-(hydroxymethyl)glutathione dehydrogenase / alcohol dehydrogenase [EC:1.1.1.284 1.1.1.1] | (RefSeq) alcohol dehydrogenase-like 2 (A)	Alcohol dehydrogenase-like 2; EC=1.1.1.1 {ECO:0000250|UniProtKB:P06525} (At2g07550)		-6.311
Pyruvate metabolism [ko00620; P = 0.5550 (P3CR vs. P3R) and 0.6851 (P5CR vs. P5R)]				
Cs1g06250	K00028 malate dehydrogenase (decarboxylating) [EC:1.1.1.39] | (RefSeq) probable F-box protein At4g22060 (A)	Putative F-box protein At3g25750 (At1g65740)	-2.332	
Cs1g20920	K01610 phosphoenolpyruvate carboxykinase (ATP) [EC:4.1.1.49] | (RefSeq) phosphoenolpyruvate carboxykinase [ATP]-like (A)	Phosphoenolpyruvate carboxykinase (ATP); PEP carboxykinase; PEPCK; EC=4.1.1.49 (At4g37870)		1.085
Cs1g24850	K00128 aldehyde dehydrogenase (NAD+) [EC:1.2.1.3] | (RefSeq) aldehyde dehydrogenase family 3 member F1 (A)	Aldehyde dehydrogenase family 3 member F1; EC=1.2.1.3 (At4g36250)	1.544	
Cs1g25560	K00025 malate dehydrogenase [EC:1.1.1.37] | (RefSeq) malate dehydrogenase, cytoplasmic-like (A)	Oligopeptide transporter 7; AtOPT7 (At4g10770)	-1.049	
Cs3g14390	K00128 aldehyde dehydrogenase (NAD+) [EC:1.2.1.3] | (RefSeq) aldehyde dehydrogenase 5, mitochondrial-like (A)	Translationally-controlled tumor protein homolog; TCTP (At3g16640)		-2.145
Cs3g16700	K01610 phosphoenolpyruvate carboxykinase (ATP) [EC:4.1.1.49] | (RefSeq) phosphoenolpyruvate carboxykinase [ATP]-like (A)	Phosphoenolpyruvate carboxykinase (ATP); PEP carboxykinase; PEPCK; EC=4.1.1.49 (At4g37870)		2.281
Cs3g16870	K00025 malate dehydrogenase [EC:1.1.1.37] | (RefSeq) malate dehydrogenase, cytoplasmic-like (A)	Oligopeptide transporter 4; AtOPT4 (At5g64410)	1.016	

Cs3g18630	K11262 acetyl-CoA carboxylase / biotin carboxylase 1 [EC:6.4.1.2 6.3.4.14 2.1.3.15] | (RefSeq) acetyl-CoA carboxylase 1-like (A)	Acetyl-CoA carboxylase 1; AtACC1; EC=6.4.1.2 {ECO:0000269|PubMed:9008389}; Protein EMBRYO DEFECTIVE 22; Protein GURKE; Protein PASTICCINO 3; Includes: Biotin carboxylase; EC=6.3.4.14 (At1g36160)		1.119
Cs3g20350	K01913 acetate-CoA ligase [EC:6.2.1.1] | (RefSeq) acetate/butyrateCoA ligase AAE7, peroxisomal-like isoform X1 (A)	Probable acyl-activating enzyme 6; EC=6.2.1.-; AMP-binding protein 6; AtAMPBP6 (At5g16340)		-1.085
Cs4g08360	K00025 malate dehydrogenase [EC:1.1.1.37] | (RefSeq) malate dehydrogenase, cytoplasmic-like (A)	Oligopeptide transporter 1; AtOPT1 (At5g55930)	1.141	
Cs4g08370	K00025 malate dehydrogenase [EC:1.1.1.37] | (RefSeq) malate dehydrogenase, cytoplasmic-like (A)	Oligopeptide transporter 1; AtOPT1 (At5g55930)		-1.145
Cs4g08380	K00025 malate dehydrogenase [EC:1.1.1.37] | (RefSeq) malate dehydrogenase, cytoplasmic-like (A)	Oligopeptide transporter 1; AtOPT1 (At5g55930)		-1.837
Cs4g14970	K01913 acetate-CoA ligase [EC:6.2.1.1] | (RefSeq) acetate/butyrateCoA ligase AAE7, peroxisomal-like (A)	Probable acyl-activating enzyme 6; EC=6.2.1.-; AMP-binding protein 6; AtAMPBP6 (At5g16340)		-1.048
Cs8g02970	K00025 malate dehydrogenase [EC:1.1.1.37] | (RefSeq) malate dehydrogenase, cytoplasmic-like (A)	Oligopeptide transporter 4; AtOPT4 (At5g64410)	-1.428	
Cs9g02540	K01913 acetate-CoA ligase [EC:6.2.1.1] | (RefSeq) acetate/butyrateCoA ligase AAE7, peroxisomal-like (A)	Acetate/butyrateCoA ligase AAE7, peroxisomal; EC=6.2.1.1; EC=6.2.1.2; AMP-binding protein 7; AtAMPBP7; Acetyl-CoA synthetase; Acyl-activating enzyme 7; Butyryl-CoA synthetase; Protein ACETATE NON-UTILIZING 1 (At3g16910)		-1.628
Cs9g02550	K01913 acetate-CoA ligase [EC:6.2.1.1] | (RefSeq) hypothetical protein (A)	Acetate/butyrateCoA ligase AAE7, peroxisomal; EC=6.2.1.1; EC=6.2.1.2; AMP-binding protein 7; AtAMPBP7; Acetyl-CoA synthetase; Acyl-activating enzyme 7; Butyryl-CoA synthetase; Protein ACETATE NON-UTILIZING 1 (At3g16910)	1.479	
Cs9g04200	K00028 malate dehydrogenase (decarboxylating) [EC:1.1.1.39] | (RefSeq) probable F-box protein At4g22060 (A)	F-box protein At2g17036 (At2g17030_3)		-1.898
Cs9g09130	K01759 lactoylglutathione lyase [EC:4.4.1.5] | (RefSeq) probable lactoylglutathione lyase, chloroplastic (A)	Protein SRC2 {ECO:0000305}; Protein SOYBEAN GENE REGULATED BY COLD 2 {ECO:0000303|Ref.1} (At2g33320)		1.392
Cs9g18120	K00873 pyruvate kinase [EC:2.7.1.40] | (RefSeq) hypothetical protein (A)	Plastidial pyruvate kinase 4, chloroplastic; PKp4; EC=2.7.1.40; Flags: Precursor (At3g49160)	-1.526	-1.586
Pentose phosphate pathway [ko00030; P = 0.8023 (P3CR vs. P3R) and 0.9919 (P5CR vs. P5R)]				
Cs1g15970	K00850 6-phosphofructokinase 1 [EC:2.7.1.11] | (RefSeq)	ATP-dependent 6-phosphofructokinase 3	1.064	

	ATP-dependent 6-phosphofructokinase 3-like (A)	{ECO:0000255|HAMAP-Rule:MF_03186}; ATP-PFK 3 {ECO:0000255|HAMAP-Rule:MF_03186}; Phosphofructokinase 3 {ECO:0000255|HAMAP-Rule:MF_03186}; EC=2.7.1.11 {ECO:0000255|HAMAP-Rule:MF_03186}; Phosphohexokinase 3 {ECO:0000255|HAMAP-Rule:MF_03186} (At4g29220)		
Cs2g29670	K00895 diphosphate-dependent phosphofructokinase [EC:2.7.1.90] | (RefSeq) pyrophosphatefructose 6-phosphate 1-phosphotransferase subunit alpha-like (A)	(At1g50570_2)	1.233	1.301
Cs3g17650	K00850 6-phosphofructokinase 1 [EC:2.7.1.11] | (RefSeq) ATP-dependent 6-phosphofructokinase 6-like (A)	Purine permease 1 {ECO:0000303|PubMed:10662864}; AtPUP1 {ECO:0000303|PubMed:10662864} (At1g28230)		-1.500
Cs3g21280	K03841 fructose-1,6-bisphosphatase I [EC:3.1.3.11] | (RefSeq) fructose-1,6-bisphosphatase, cytosolic (A)	Fructose-1,6-bisphosphatase, cytosolic; FBPase; EC=3.1.3.11; CY-F1; D-fructose-1,6-bisphosphate 1-phosphohydrolase (At1g43670)		1.036
Cs4g13070	K00850 6-phosphofructokinase 1 [EC:2.7.1.11] | (RefSeq) ATP-dependent 6-phosphofructokinase 3-like (A)	ATP-dependent 6-phosphofructokinase 3 {ECO:0000255|HAMAP-Rule:MF_03186}; ATP-PFK 3 {ECO:0000255|HAMAP-Rule:MF_03186}; Phosphofructokinase 3 {ECO:0000255|HAMAP-Rule:MF_03186}; EC=2.7.1.11 {ECO:0000255|HAMAP-Rule:MF_03186}; Phosphohexokinase 3 {ECO:0000255|HAMAP-Rule:MF_03186} (At5g56630)	-1.258	
Cs4g18980	K00850 6-phosphofructokinase 1 [EC:2.7.1.11] | (RefSeq) hypothetical protein (A)	ATP-dependent 6-phosphofructokinase 3 {ECO:0000255|HAMAP-Rule:MF_03186}; ATP-PFK 3 {ECO:0000255|HAMAP-Rule:MF_03186}; Phosphofructokinase 3 {ECO:0000255|HAMAP-Rule:MF_03186}; EC=2.7.1.11 {ECO:0000255|HAMAP-Rule:MF_03186}; Phosphohexokinase 3 {ECO:0000255|HAMAP-Rule:MF_03186} (At4g32840)	-1.602	-1.466
Cs7g24920	K00615 transketolase [EC:2.2.1.1] | (RefSeq) transketolase, chloroplastic-like (A)	Transketolase-1, chloroplastic; TK; EC=2.2.1.1; Flags: Precursor (At2g45290)	1.474	-1.189
Cs8g08710	K01623 fructose-bisphosphate aldolase, class I [EC:4.1.2.13] | (RefSeq) fructose-bisphosphate aldolase 1, chloroplastic (A)	Fructose-bisphosphate aldolase 2, chloroplastic {ECO:0000305}; AtFBA2 {ECO:0000303|PubMed:22561114}; EC=4.1.2.13 {ECO:0000305}; Flags: Precursor (At2g01140)	1.927	
Cs9g17270	K01623 fructose-bisphosphate aldolase, class I [EC:4.1.2.13] | (RefSeq) fructose-bisphosphate aldolase 6, cytosolic-like (A)	Heavy metal-associated isoprenylated plant protein 39 {ECO:0000303|PubMed:21072340, ECO:0000303|PubMed:23368984}; AtHIP39 {ECO:0000303|PubMed:21072340,		-1.134

		ECO:0000303|PubMed:23368984}; Flags: Precursor (At1g01490)		
	Oxidative phosphorylation [ko00190; P = 0.7299 (P3CR vs. P3R) and 0.9997 (P5CR vs. P5R)]			
Cs1g15410	K03946 NADH dehydrogenase (ubiquinone) 1 alpha subcomplex subunit 2 | (RefSeq) NADH dehydrogenase [ubiquinone] 1 alpha subcomplex subunit 2-like (A)	NADH dehydrogenase [ubiquinone] 1 alpha subcomplex subunit 2 (At5g47890)		-1.209
Cs1g16160	K01535 H+-transporting ATPase [EC:3.6.3.6] | (RefSeq) hypothetical protein (A)	ATPase 10, plasma membrane-type; EC=3.6.3.6; Proton pump 10 (At1g17260)		2.076
Cs1g21090	K02266 cytochrome c oxidase subunit 6a | (RefSeq) cytochrome c oxidase subunit 6a, mitochondrial (A)	Cytochrome c oxidase subunit 6a, mitochondrial; AtCOX6a; Flags: Precursor (At4g37830)		-1.055
Cs5g08370	K01535 H+-transporting ATPase [EC:3.6.3.6] | (RefSeq) hypothetical protein (A)	ATPase 11, plasma membrane-type; EC=3.6.3.6; Proton pump 11 (At5g62670)		-3.477
Cs6g03430	K01535 H+-transporting ATPase [EC:3.6.3.6] | (RefSeq) plasma membrane ATPase 1-like (A)	ATPase 7, plasma membrane-type; EC=3.6.3.6; Proton pump 7 (At3g60330)	2.160	
Cs6g03440	K01535 H+-transporting ATPase [EC:3.6.3.6] | (RefSeq) hypothetical protein (A)	Plasma membrane ATPase 1; EC=3.6.3.6; Proton pump 1 (At5g62670)	2.437	
Cs6g03445	K01535 H+-transporting ATPase [EC:3.6.3.6] | (RefSeq) hypothetical protein (A)	ATPase 4, plasma membrane-type; EC=3.6.3.6; Proton pump 4 (At1g80660)	2.470	
Cs6g03470	K01535 H+-transporting ATPase [EC:3.6.3.6] | (RefSeq) ATPase 4, plasma membrane-type-like (A)	Putative ATPase, plasma membrane-like (At4g11730)	1.173	
Cs6g03480	K01535 H+-transporting ATPase [EC:3.6.3.6] | (RefSeq) plasma membrane ATPase 1-like (A)	Plasma membrane ATPase 1; EC=3.6.3.6; Proton pump 1 (At5g62670)	2.445	
Cs6g03490	K01535 H+-transporting ATPase [EC:3.6.3.6] | (RefSeq) hypothetical protein (A)	ATPase 4, plasma membrane-type; EC=3.6.3.6; Proton pump 4 (At5g62670)	2.662	
Cs6g15750	K03942 NADH dehydrogenase (ubiquinone) flavoprotein 1 [EC:1.6.5.3 1.6.99.3] | (RefSeq) NADH dehydrogenase [ubiquinone] flavoprotein 1, mitochondrial-like (A)	(At1g67020)		1.214
Cs6g20570	K01535 H+-transporting ATPase [EC:3.6.3.6] | (RefSeq) plasma membrane ATPase 1-like (A)	Plasma membrane ATPase 1; EC=3.6.3.6; Proton pump 1 (At3g47950)	1.223	
Cs8g03160	K01507 inorganic pyrophosphatase [EC:3.6.1.1] | (RefSeq) soluble inorganic pyrophosphatase 6, chloroplastic (A)	Soluble inorganic pyrophosphatase 6, chloroplastic {ECO:0000303|PubMed:15135060}; EC=3.6.1.1 {ECO:0000269|PubMed:15135060}; Inorganic pyrophosphatase 6; Pyrophosphate phospho-hydrolase 6	-1.001	

		{ECO:0000303|PubMed:15135060}; PPase 6 {ECO:0000303|PubMed:15135060}; Flags: Precursor (At5g09650)		
novel.2012	K00234 succinate dehydrogenase (ubiquinone) flavoprotein subunit [EC:1.3.5.1] | (RefSeq) succinate dehydrogenase [ubiquinone] flavoprotein subunit, mitochondrial-like (A)	(At2g18950)	1.093	1.829
ATP biosynthetic process [GO:0006754; P = 0.0383 (P3CR vs. P3R) and 0.9435 (P5CR vs. P5R)]				
Cs1g15840	K00844 hexokinase [EC:2.7.1.1] | (RefSeq) hexokinase-1-like (A)	Hexokinase-1; EC=2.7.1.1; NtHxK1 (At4g29130)		-1.611
Cs1g15970	K00850 6-phosphofructokinase 1 [EC:2.7.1.11] | (RefSeq) ATP-dependent 6-phosphofructokinase 3-like (A)	ATP-dependent 6-phosphofructokinase 3 {ECO:0000255|HAMAP-Rule:MF_03186}; ATP-PFK 3 {ECO:0000255|HAMAP-Rule:MF_03186}; Phosphofructokinase 3 {ECO:0000255|HAMAP-Rule:MF_03186}; EC=2.7.1.11 {ECO:0000255|HAMAP-Rule:MF_03186}; Phosphohexokinase 3 {ECO:0000255|HAMAP-Rule:MF_03186} (At4g29220)	1.064	
Cs1g16160	K01535 H+-transporting ATPase [EC:3.6.3.6] | (RefSeq) hypothetical protein (A)	ATPase 10, plasma membrane-type; EC=3.6.3.6; Proton pump 10 (At1g17260)		2.076
Cs4g13070	K00850 6-phosphofructokinase 1 [EC:2.7.1.11] | (RefSeq) ATP-dependent 6-phosphofructokinase 3-like (A)	ATP-dependent 6-phosphofructokinase 3 {ECO:0000255|HAMAP-Rule:MF_03186}; ATP-PFK 3 {ECO:0000255|HAMAP-Rule:MF_03186}; Phosphofructokinase 3 {ECO:0000255|HAMAP-Rule:MF_03186}; EC=2.7.1.11 {ECO:0000255|HAMAP-Rule:MF_03186}; Phosphohexokinase 3 {ECO:0000255|HAMAP-Rule:MF_03186} (At5g56630)	-1.258	
Cs4g18980	K00850 6-phosphofructokinase 1 [EC:2.7.1.11] | (RefSeq) hypothetical protein (A)	ATP-dependent 6-phosphofructokinase 3 {ECO:0000255|HAMAP-Rule:MF_03186}; ATP-PFK 3 {ECO:0000255|HAMAP-Rule:MF_03186}; Phosphofructokinase 3 {ECO:0000255|HAMAP-Rule:MF_03186}; EC=2.7.1.11 {ECO:0000255|HAMAP-Rule:MF_03186}; Phosphohexokinase 3 {ECO:0000255|HAMAP-Rule:MF_03186} (At4g32840)	-1.602	-1.466
Cs5g08370	K01535 H+-transporting ATPase [EC:3.6.3.6] | (RefSeq) hypothetical protein (A)	ATPase 11, plasma membrane-type; EC=3.6.3.6; Proton pump 11 (At5g62670)		-3.477
Cs6g03430	K01535 H+-transporting ATPase [EC:3.6.3.6] | (RefSeq) plasma membrane ATPase 1-like (A)	ATPase 7, plasma membrane-type; EC=3.6.3.6; Proton pump 7 (At3g60330)	2.160	
Cs6g03440	K01535 H+-transporting ATPase [EC:3.6.3.6] | (RefSeq) hypothetical protein (A)	Plasma membrane ATPase 1; EC=3.6.3.6; Proton pump 1 (At5g62670)	2.437	

Cs6g03445	K01535 H+-transporting ATPase [EC:3.6.3.6] | (RefSeq) hypothetical protein (A)	ATPase 4, plasma membrane-type; EC=3.6.3.6; Proton pump 4 (At1g80660)	2.470	
Cs6g03480	K01535 H+-transporting ATPase [EC:3.6.3.6] | (RefSeq) plasma membrane ATPase 1-like (A)	Plasma membrane ATPase 1; EC=3.6.3.6; Proton pump 1 (At5g62670)	2.445	
Cs6g03490	K01535 H+-transporting ATPase [EC:3.6.3.6] | (RefSeq) hypothetical protein (A)	ATPase 4, plasma membrane-type; EC=3.6.3.6; Proton pump 4 (At5g62670)	2.662	
Cs6g20570	K01535 H+-transporting ATPase [EC:3.6.3.6] | (RefSeq) plasma membrane ATPase 1-like (A)	Plasma membrane ATPase 1; EC=3.6.3.6; Proton pump 1 (At3g47950)	1.223	
Cs6g20790	K09285 AP2-like factor, ANT lineage | (RefSeq) AP2-like ethylene-responsive transcription factor At1g16060 (A)	Ethylene-responsive transcription factor WRI1; Protein ACTIVATOR OF SPORAMIN::LUC 1; Protein WRINKLED 1 (At1g16060)	1.439	-2.381
Cs7g04300	K09285 AP2-like factor, ANT lineage | (RefSeq) AP2-like ethylene-responsive transcription factor At1g79700 (A)	Ethylene-responsive transcription factor WRI1; Protein ACTIVATOR OF SPORAMIN::LUC 1; Protein WRINKLED 1 (At5g17430)		-5.678
Cs8g08710	K01623 fructose-bisphosphate aldolase, class I [EC:4.1.2.13] | (RefSeq) fructose-bisphosphate aldolase 1, chloroplastic (A)	Fructose-bisphosphate aldolase 2, chloroplastic {ECO:0000305}; AtFBA2 {ECO:0000303|PubMed:22561114}; EC=4.1.2.13 {ECO:0000305}; Flags: Precursor (At2g01140)	1.927	
Cs9g18120	K00873 pyruvate kinase [EC:2.7.1.40] | (RefSeq) hypothetical protein (A)	Plastidial pyruvate kinase 4, chloroplastic; PKp4; EC=2.7.1.40; Flags: Precursor (At3g49160)	-1.526	-1.586
**Index**	**Compounds**		**Log_2(fold change)**	
			P3CR vs. P3R	P5CR vs. P5R
Starch and sucrose metabolism [ko00500; P = 0.4275 (P3CR vs. P3R) and 0.1167 (P5CR vs. P5R)]				
mws1090	Glucose-1-phosphate		-1.032	
mws2523	Trehalose 6-phosphate			1.198
mws4170	D-Glucose		1.172	
pmb2922	Uridine 5'-diphospho-D-glucose		-4.189	
pme3313	D-Fructose 6-phosphate		-1.146	1.037
Zmyn000083	D-Glucose 1,6-bisphosphate			1.216
Citrate cycle (TCA cycle) [ko00020; P = 0.9362 (P3CR vs. P3R)]				
Zmyn000453	Isocitric acid		-2.158	

Glycolysis / Gluconeogenesis* [ko00010; *P = 0.2567 (P3CR vs. P3R) and 0.5319 (P5CR vs. P5R)]				
pme3472	Arbutin		-1.328	
mws1090	Glucose-1-phosphate		-1.032	
mws4170	D-Glucose		1.172	
Zmzn000078	Dihydroxyacetone phosphate			1.353
Pentose phosphate pathway [ko00030; P = 0.7102 (P3CR vs. P3R) and 0.1987 (P5CR vs. P5R)]				
mws4170	D-Glucose		1.172	
pma6455	Ribulose-5-phosphate			1.628
pmb2507	2-Deoxyribose-1-phosphate		-1.659	
Zmzn000079	D-Erythrose-4-phosphate			1.247
Oxidative phosphorylation [ko00190; P = 0.2567 (P3CR vs. P3R) and 0.0900 (P3CR vs. P5CR)]				
mws0192	succinic acid			
MWS5083	Flavin Single Nucleotide(FMN)		-1.351	
pmb0530	Nicotinic acid adenine dinucleotide		-4.606	
pme2117	Adenosine 5'-diphosphate		-1.571	
