# Supplementary material for: Mechanisms by Which Increased pH Ameliorates Copper Excess in Citrus sinensis Roots: Insight from a Combined Analysis of Physiology, Transcriptome, and Metabolome
Source: Plants (Basel). 2024 Oct 31;13(21):3054. doi: 10.3390/plants13213054 (PMC11548300; doi:10.3390/plants13213054)
Supplement: Supplementary file 1 [file plants-13-03054-s001.zip › Table S15SecondRevision.pdf]

Table S15. DEGs and DAMs related to N, protein, and AA metabolisms in P3CR vs. P3R and/or P5CR vs. P5R

Accession No.	KEGG	Swissprot (KOG)	Log_2(FC)	
			P3CR vs. P3R	P5CR vs. P5R
Nitrogen metabolism [ko00910; P = (P3CR vs. P3R) and 0.1829 (P5CR vs. P5R)]				
Cs3g17310	K01673 carbonic anhydrase [EC:4.2.1.1] | (RefSeq) beta carbonic anhydrase 5, chloroplastic-like (A)	Beta carbonic anhydrase 5, chloroplastic; AtbCA5; AtbetaCA5; EC=4.2.1.1; Beta carbonate dehydratase 5; Flags: Precursor (At1g58180)		-1.536
Cs3g19060	K10534 nitrate reductase (NAD(P)H) [EC:1.7.1.1 1.7.1.2 1.7.1.3] | (RefSeq) nitrate reductase [NAD(P)H]-like (A)	Nitrate reductase [NAD(P)H]; NR; EC=1.7.1.2 (At1g37130_1)	-1.532	
Cs5g05410	K01673 carbonic anhydrase [EC:4.2.1.1] | (RefSeq) carbonic anhydrase 2-like (A)	Carbonic anhydrase, chloroplastic; EC=4.2.1.1; Carbonate dehydratase; Flags: Precursor (At5g14740)		-4.075
Cs7g19160	K00261 glutamate dehydrogenase (NAD(P)+) [EC:1.4.1.3] | (RefSeq) hypothetical protein (A)	Glutamate dehydrogenase 2; GDH 2; EC=1.4.1.3 (At5g07440)		-1.412
Cs8g16000	K02575 MFS transporter, NNP family, nitrate/nitrite transporter | (RefSeq) high affinity nitrate transporter 2.6-like (A)	High affinity nitrate transporter 2.6; AtNRT2:6 (At3g45060)	2.956	
Cs8g16010	K02575 MFS transporter, NNP family, nitrate/nitrite transporter | (RefSeq) high affinity nitrate transporter 2.4-like (A)	High affinity nitrate transporter 2.4; AtNRT2:4 (At5g60770)		3.094
Cs8g16250	K01674 carbonic anhydrase [EC:4.2.1.1] | (RefSeq) alpha carbonic anhydrase 7-like (A)	Alpha carbonic anhydrase 7; AtaCA7; AtalphaCA7; EC=4.2.1.1; Alpha carbonate dehydratase 7; Flags: Precursor (At1g08080)		1.044
novel.1786	K02575 MFS transporter, NNP family, nitrate/nitrite transporter | (RefSeq) high affinity nitrate transporter 2.4-like (A)	NRT24_ARATH High affinity nitrate transporter 2.4; AtNRT2:4 (At5g60770)		2.755
Cellular nitrogen compound catabolic process [GO:0044270; P = 0.9162 (P3CR vs. P3R) and 0.9821 (P5CR vs. P5R)]				
Cs1g15710	K05681 ATP-binding cassette, subfamily G (WHITE), member 2 | (RefSeq) hypothetical protein (A)	ABC transporter G family member 36; ABC transporter ABCG.36; AtABCG36; Pleiotropic drug resistance protein 8; Protein PENETRATION 3 (At1g59870)		-2.119
Cs1g15840	K00844 hexokinase [EC:2.7.1.1] | (RefSeq) hexokinase-1-like (A)	Hexokinase-1; EC=2.7.1.1; NtHxK1 (At4g29130)		-1.611
Cs1g15970	K00850 6-phosphofructokinase 1 [EC:2.7.1.11] | (RefSeq) ATP-dependent 6-phosphofructokinase 3-like (A)	ATP-dependent 6-phosphofructokinase 3 {ECO:0000255|HAMAP-Rule:MF_03186}; ATP-PFK 3 {ECO:0000255|HAMAP-Rule:MF_03186}; Phosphofructokinase 3 {ECO:0000255|HAMAP-Rule:MF_03186}; EC=2.7.1.11	1.064	

		{ECO:0000255|HAMAP-Rule:MF_03186}; Phosphohexokinase 3 {ECO:0000255|HAMAP-Rule:MF_03186} (At4g29220)		
Cs1g22680	K13545 red chlorophyll catabolite reductase [EC:1.3.7.12] | (RefSeq) red chlorophyll catabolite reductase-like (A)	Red chlorophyll catabolite reductase, chloroplastic; AtRCCR; RCC reductase; EC=1.3.7.12 {ECO:0000269|PubMed:10743659}; Accelerated cell death protein 2; Flags: Precursor (At4g37000)	1.740	
Cs2g05830	K14409 protein SMG7 | (RefSeq) protein SMG7L-like isoform X1 (A)	Protein SMG7L; Protetin SMG7-like (At1g28260)		1.123
Cs2g06670		Endonuclease 1; AtENDO1; EC=3.1.30.1; Bifunctional nuclease I; AtBFN1; Deoxyribonuclease ENDO1; Single-stranded-nucleate endonuclease ENDO1; Flags: Precursor (At1g11190)	1.691	3.814
Cs2g08400	K17943 pumilio RNA-binding family | (RefSeq) pumilio homolog 5 (A)	Pumilio homolog 5; APUM-5; AtPUM5 (At3g20250)	1.143	
Cs2g12650		Endonuclease 2; AtENDO2; EC=3.1.30.1; Deoxyribonuclease ENDO2; Single-stranded-nucleate endonuclease ENDO2; Flags: Precursor (At1g68290)		-2.089
Cs2g13240	K18753 butyrate response factor | (RefSeq) hypothetical protein (A)	Zinc finger CCCH domain-containing protein 15; AtC3H15 (At1g68200)		1.504
Cs4g13070	K00850 6-phosphofructokinase 1 [EC:2.7.1.11] | (RefSeq) ATP-dependent 6-phosphofructokinase 3-like (A)	ATP-dependent 6-phosphofructokinase 3 {ECO:0000255|HAMAP-Rule:MF_03186}; ATP-PFK 3 {ECO:0000255|HAMAP-Rule:MF_03186}; Phosphofructokinase 3 {ECO:0000255|HAMAP-Rule:MF_03186}; EC=2.7.1.11 {ECO:0000255|HAMAP-Rule:MF_03186}; Phosphohexokinase 3 {ECO:0000255|HAMAP-Rule:MF_03186} (At5g56630)	-1.258	
Cs4g18980	K00850 6-phosphofructokinase 1 [EC:2.7.1.11] | (RefSeq) hypothetical protein (A)	ATP-dependent 6-phosphofructokinase 3 {ECO:0000255|HAMAP-Rule:MF_03186}; ATP-PFK 3 {ECO:0000255|HAMAP-Rule:MF_03186}; Phosphofructokinase 3 {ECO:0000255|HAMAP-Rule:MF_03186}; EC=2.7.1.11 {ECO:0000255|HAMAP-Rule:MF_03186}; Phosphohexokinase 3 {ECO:0000255|HAMAP-Rule:MF_03186} (At4g32840)	-1.602	-1.466
Cs5g03020	K10878 meiotic recombination protein SPO11 | (RefSeq) hypothetical protein (A)	Meiotic recombination protein SPO11-2; AtSPO11-2; EC=5.99.1.3 {ECO:0000305} (At1g63990)	1.403	1.274
Cs6g12480	K12581 CCR4-NOT transcription complex subunit 7/8 | (RefSeq) probable CCR4-associated factor 1 homolog 11 (A)	Probable CCR4-associated factor 1 homolog 9; EC=3.1.13.4 (At3g44260)	1.119	
Cs6g13950	K12581 CCR4-NOT transcription complex subunit 7/8 |	Probable CCR4-associated factor 1 homolog 11; EC=3.1.13.4		-1.142

	(RefSeq) probable CCR4-associated factor 1 homolog 11 (A)	(At5g22250)		
Cs6g15100	K01240 uridine nucleosidase [EC:3.2.2.3] | (RefSeq) uridine nucleosidase 1 (A)	Uridine nucleosidase 1; EC=3.2.2.3; Uridine ribohydrolase 1 (At2g36310)		-1.188
Cs6g20790	K09285 AP2-like factor, ANT lineage | (RefSeq) AP2-like ethylene-responsive transcription factor At1g16060 (A)	Ethylene-responsive transcription factor WRI1; Protein ACTIVATOR OF SPORAMIN::LUC 1; Protein WRINKLED 1 (At1g16060)	1.439	-2.381
Cs7g04300	K09285 AP2-like factor, ANT lineage | (RefSeq) AP2-like ethylene-responsive transcription factor At1g79700 (A)	Ethylene-responsive transcription factor WRI1; Protein ACTIVATOR OF SPORAMIN::LUC 1; Protein WRINKLED 1 (At5g17430)		-5.678
Cs7g06230	K12581 CCR4-NOT transcription complex subunit 7/8 | (RefSeq) hypothetical protein (A)	Probable CCR4-associated factor 1 homolog 7; EC=3.1.13.4 (At1g80780)		-1.056
Cs7g15290	K19036 ATP-dependent RNA/DNA helicase IGHMBP2 [EC:3.6.4.12 3.6.4.13] | (RefSeq) DNA-binding protein SMUBP-2 isoform X1 (A)	Regulator of nonsense transcripts 1 homolog; EC=3.6.4.-; ATP-dependent helicase UPF1 (At5g35970)		1.277
Cs8g08710	K01623 fructose-bisphosphate aldolase, class I [EC:4.1.2.13] | (RefSeq) fructose-bisphosphate aldolase 1, chloroplastic (A)	Fructose-bisphosphate aldolase 2, chloroplastic {ECO:0000305}; AtFBA2 {ECO:0000303|PubMed:22561114}; EC=4.1.2.13 {ECO:0000305}; Flags: Precursor (At2g01140)	1.927	
Cs8g13100	K13035 beta-cyano-L-alanine hydratase/nitrilase [EC:3.5.5.4 4.2.1.65] | (RefSeq) bifunctional nitrilase/nitrile hydratase NIT4A-like (A)	Bifunctional nitrilase/nitrile hydratase NIT4A; LaNIT4A; EC=3.5.5.4; EC=4.2.1.65; 3-cyanoalanine hydratase; Cyanoalanine nitrilase A (At5g22300)		1.336
Cs9g05270	K01240 uridine nucleosidase [EC:3.2.2.3] | (RefSeq) uridine nucleosidase 1-like (A)	Probable uridine nucleosidase 2; EC=3.2.2.3; Uridine ribohydrolase 2 (At5g18860)		1.915
Cs9g18120	K00873 pyruvate kinase [EC:2.7.1.40] | (RefSeq) hypothetical protein (A)	Plastidial pyruvate kinase 4, chloroplastic; PKp4; EC=2.7.1.40; Flags: Precursor (At3g49160)	-1.526	-1.586
orange1.1t00584	K11592 endoribonuclease Dicer [EC:3.1.26.-] | (RefSeq) endoribonuclease Dicer homolog 1 (A)	Endoribonuclease Dicer homolog 1; EC=3.1.26.-; Dicer-like protein 1; AtDCL1; Protein ABNORMAL SUSPENSOR 1; Protein CARPEL FACTORY; Protein SHORT INTEGUMENTS 1; Protein SUSPENSOR 1 (At1g01040)		1.186
orange1.1t02678	K15308 tristetraprolin | (RefSeq) tristetraprolin-like (A)	Zinc finger CCCH domain-containing protein 39; OsC3H39;(Hs4507961)	2.060	
orange1.1t04212	K17710 pentatricopeptide repeat domain-containing protein 1 | (RefSeq) hypothetical protein (A)	Pentatricopeptide repeat-containing protein MRL1, chloroplastic; Protein MATURATION OF RBCL 1; AtMRL1; Flags: Precursor (At4g34830)		1.340
orange1.1t04402	K03012 DNA-directed RNA polymerase II subunit RPB4 | (RefSeq) DNA-directed RNA polymerase II subunit 4-like (A)	DNA-directed RNA polymerase II subunit 4; 15.9 kDa subunit of RNA polymerase II; DNA-directed RNA polymerase II subunit D (At5g09920)	-2.184	-2.017

Senescence-associated vacuole [GO:0010282; P = 0.0710 (P3CR vs. P3R) and 0.1127 (P5CR vs. P5R)]				
Cs2g04940	K01365 cathepsin L [EC:3.4.22.15] | (RefSeq) hypothetical protein (A)	Senescence-specific cysteine protease SAG39 {ECO:0000305}; EC=3.4.22.- {ECO:0000305}; Cysteine proteinase SAG39 {ECO:0000305}; Protein SENESCENCE-ASSOCIATED GENE 39 {ECO:0000250|UniProtKB:Q7XWK5}; Flags: Precursor (At5g45890)	2.537	-5.958
Cs2g15480	K16292 KDEL-tailed cysteine endopeptidase [EC:3.4.22.-] | (RefSeq) senescence-specific cysteine protease SAG39-like (A)	Senescence-specific cysteine protease SAG39 {ECO:0000305}; EC=3.4.22.- {ECO:0000305}; Cysteine proteinase SAG39 {ECO:0000305}; Protein SENESCENCE-ASSOCIATED GENE 39 {ECO:0000250|UniProtKB:Q7XWK5}; Flags: Precursor (At5g45890)		-5.160
Cs2g15490	K16292 KDEL-tailed cysteine endopeptidase [EC:3.4.22.-] | (RefSeq) senescence-specific cysteine protease SAG39-like (A)	Senescence-specific cysteine protease SAG39 {ECO:0000305}; EC=3.4.22.- {ECO:0000305}; Cysteine proteinase SAG39 {ECO:0000305}; Protein SENESCENCE-ASSOCIATED GENE 39 {ECO:0000250|UniProtKB:Q7XWK5}; Flags: Precursor (At5g45890)	1.346	-7.852
Cs2g15700	K16292 KDEL-tailed cysteine endopeptidase [EC:3.4.22.-] | (RefSeq) senescence-specific cysteine protease SAG39 (A)	Senescence-specific cysteine protease SAG39 {ECO:0000305}; EC=3.4.22.- {ECO:0000305}; Cysteine proteinase SAG39 {ECO:0000305}; Protein SENESCENCE-ASSOCIATED GENE 39 {ECO:0000303|PubMed:20439547}; Flags: Precursor (At5g45890)	2.123	-3.210
Cs2g27790	K01365 cathepsin L [EC:3.4.22.15] | (RefSeq) zingipain-1-like (A)	Senescence-specific cysteine protease SAG12 {ECO:0000305}; EC=3.4.22.- {ECO:0000305}; Cysteine proteinase SAG12 {ECO:0000305}; Protein SENESCENCE-ASSOCIATED GENE 12 {ECO:0000303|Ref.6}; Flags: Precursor (At3g49340)		-1.325
Cs9g13580	K01365 cathepsin L [EC:3.4.22.15] | (RefSeq) zingipain-2-like (A)	Senescence-specific cysteine protease SAG12 {ECO:0000305}; EC=3.4.22.- {ECO:0000305}; Cysteine proteinase SAG12 {ECO:0000305}; Protein SENESCENCE-ASSOCIATED GENE 12 {ECO:0000303|Ref.6}; Flags: Precursor (At3g49340)	7.122	
Autophagy [GO:0006914; P = 0.9815 (P3CR vs. P3R)]				
Cs4g05020	K20296 vacuolar protein sorting-associated protein 51 | (RefSeq) hypothetical protein (A)	Vacuolar protein sorting-associated protein 51 homolog {ECO:0000303|PubMed:24757006}; Protein UNHINGED {ECO:0000303|PubMed:24757006} (At4g02030)		1.026
Cs6g09420	K13424 WRKY transcription factor 33 | (RefSeq) hypothetical protein (A)	Probable WRKY transcription factor 33; WRKY DNA-binding protein 33 (At2g38470)	1.908	1.729
Cs7g08800	K00914 phosphatidylinositol 3-kinase [EC:2.7.1.137] |	Phosphatidylinositol 3-kinase, nodule isoform; PI3-kinase; PI3K;		1.162

	(RefSeq) phosphatidylinositol 3-kinase, nodule isoform (A)	PtdIns-3-kinase; EC=2.7.1.137; SPI3K-1 (At1g60490)		
Protein ubiquitination [GO:0016567; P = 0.9967 (P3CR vs. P3R) and 0.9775 (P5CR vs. P5R)]				
Cs1g10740	K15503 serine/threonine-protein phosphatase 6 regulatory ankyrin repeat subunit B | (RefSeq) protein ACCELERATED CELL DEATH 6 (A)	Protein ACCELERATED CELL DEATH 6 {ECO:0000303|PubMed:10488236} (At1g03670)	1.841	1.928
Cs1g21880	K03063 26S proteasome regulatory subunit T3 | (RefSeq) 26S protease regulatory subunit 6B homolog (A)	BTB/POZ domain-containing protein SR1IP1 {ECO:0000305}; Protein ATSR1-INTERACTION PROTEIN 1 {ECO:0000303|PubMed:24528504} (At3g49970)	1.508	
Cs1g23960		F-box protein At5g49610 (At5g49610)	1.906	1.660
Cs2g15840	K19038 E3 ubiquitin-protein ligase ATL41 [EC:2.3.2.27] | (RefSeq) E3 ubiquitin-protein ligase ATL41-like (A)	E3 ubiquitin-protein ligase ATL41; EC=2.3.2.27 {ECO:0000269|PubMed:15644464, ECO:0000269|PubMed:16339806}; RING-H2 finger protein ATL41; RING-type E3 ubiquitin transferase ATL41 {ECO:0000305} (At2g42360)		1.014
Cs3g19900	K19045 E3 ubiquitin-protein ligase BIG BROTHER and related proteins [EC:2.3.2.27] | (RefSeq) E3 ubiquitin ligase BIG BROTHER-related-like (A)	E3 ubiquitin ligase BIG BROTHER-related; AtBBR; EC=2.3.2.27 {ECO:0000250|UniProtKB:Q8L649}; RING-type E3 ubiquitin transferase BIG BROTHER-related {ECO:0000305} (At3g47180)	-1.274	
Cs4g10410	K15503 serine/threonine-protein phosphatase 6 regulatory ankyrin repeat subunit B | (RefSeq) protein ACCELERATED CELL DEATH 6-like (A)	Protein ACCELERATED CELL DEATH 6 {ECO:0000303|PubMed:10488236} (At4g03500)		1.874
Cs4g10500	K15503 serine/threonine-protein phosphatase 6 regulatory ankyrin repeat subunit B | (RefSeq) protein ACCELERATED CELL DEATH 6-like (A)	Protein ACCELERATED CELL DEATH 6 {ECO:0000303|PubMed:10488236} (At4g03500)		1.649
Cs4g14600	K14508 regulatory protein NPR1 | (RefSeq) regulatory protein NPR1 (A)	Regulatory protein NPR1; BTB/POZ domain-containing protein NPR1; Non-inducible immunity protein 1; Nim1; Nonexpresser of PR genes 1; Salicylic acid insensitive 1; Sai1 (At1g64280)		1.055
Cs4g17630	K10443 kelch-like protein 2/3 | (RefSeq) F-box/kelch-repeat protein At1g16250-like isoform X1 (A)	F-box/kelch-repeat protein At1g55270 (At1g55270)		1.116
Cs5g03280	K16281 RING-H2 zinc finger protein RHA1 | (RefSeq) hypothetical protein (A)	E3 ubiquitin-protein ligase RHA1B {ECO:0000305}; EC=2.3.2.27; RING-H2 finger A1b {ECO:0000303|PubMed:9781696}; RING-H2 zinc finger protein RHA1b {ECO:0000305}; RING-type E3 ubiquitin transferase RHA1B {ECO:0000305} (At3g61460)	-1.059	-1.083
Cs5g04420	K16276 zinc finger protein-like protein | (RefSeq) uncharacterized LOC102613268 (A)	Zinc finger protein BRUTUS-like At1g74770 {ECO:0000305} (At1g18920)	1.352	2.402

Cs5g05990	K00276 primary-amine oxidase [EC:1.4.3.21] | (RefSeq) hypothetical protein (A)	U-box domain-containing protein 12; EC=2.3.2.27; Plant U-box protein 12; OsPUB12; RING-type E3 ubiquitin transferase PUB12 {ECO:0000305} (At3g26600)	1.336	1.395
Cs5g23550	K11975 E3 ubiquitin-protein ligase RNF144 [EC:2.3.2.31] | (RefSeq) probable E3 ubiquitin-protein ligase RNF217 (A)	Putative E3 ubiquitin-protein ligase ARI4; EC=2.3.2.27 {ECO:0000250|UniProtKB:Q9Y4X5}; ARIADNE-like protein ARI4; Protein ariadne homolog 4; RING-type E3 ubiquitin transferase ARI4 {ECO:0000305} (At3g53690)	1.062	1.428
Cs5g27990	K03063 26S proteasome regulatory subunit T3 | (RefSeq) 26S protease regulatory subunit 6B homolog (A)	BTB/POZ domain-containing protein At5g48800 (At5g48800)	1.067	1.376
Cs5g33100	K16275 E3 ubiquitin-protein ligase BAH [EC:2.3.2.27] | (RefSeq) hypothetical protein (A)	Probable E3 ubiquitin-protein ligase BAH1-like 1; EC=2.3.2.27 {ECO:0000305}; RING-type E3 ubiquitin transferase BAH1-like 1 {ECO:0000305} (At1g02860)	1.198	1.406
Cs6g12970	K12830 splicing factor 3B subunit 3 | (RefSeq) uncharacterized LOC102624787 (A)	DNA damage-binding protein 1; High pigmentation protein 1; UV-damaged DNA-binding protein 1 (At3g11960)		1.100
Cs7g04220	K10573 ubiquitin-conjugating enzyme E2 A [EC:2.3.2.23] | (RefSeq) hypothetical protein (A)	Ubiquitin-conjugating enzyme E2 2; EC=2.3.2.23; E2 ubiquitin-conjugating enzyme 2; Ubiquitin carrier protein 2; Ubiquitin-conjugating enzyme E2-17 kDa 2; Ubiquitin-protein ligase 2 (At2g02760)	-3.437	-2.891
Cs7g05010	K08332 vacuolar protein 8 | (RefSeq) U-box domain-containing protein 13-like (A)	U-box domain-containing protein 11; EC=2.3.2.27; Plant U-box protein 11; RING-type E3 ubiquitin transferase PUB11 {ECO:0000305} (At1g23030)		-5.019
Cs7g05390	K14515 EIN3-binding F-box protein | (RefSeq) EIN3-binding F-box protein 1 (A)	EIN3-binding F-box protein 1; F-box/LRR-repeat protein 6 (At2g25490)		1.135
Cs7g07200	K10443 kelch-like protein 2/3 | (RefSeq) F-box/kelch-repeat protein At1g16250-like isoform X1 (A)	F-box protein AFR; Protein ATTENUATED FAR-RED RESPONSE; SKP1-interacting partner 29 (At2g24540)		-1.567
Cs7g09390	K03063 26S proteasome regulatory subunit T3 | (RefSeq) 26S protease regulatory subunit 6B homolog (A)	BTB/POZ domain-containing protein At1g67900 (At1g67900)		1.589
Cs7g19610	K10638 E3 ubiquitin-protein ligase UHRF1 [EC:2.3.2.27] | (RefSeq) E3 ubiquitin-protein ligase ORTHRUS 2-like (A)	E3 ubiquitin-protein ligase ORTHRUS 2; EC=2.3.2.27; Protein VARIANT IN METHYLATION 1; RING-type E3 ubiquitin transferase ORTHRUS 2 {ECO:0000305} (At1g57820)		1.055
Cs7g27090	K16276 zinc finger protein-like protein | (RefSeq) uncharacterized LOC102610804 (A)	Zinc finger protein BRUTUS {ECO:0000303|PubMed:20675571}; Protein EMBRYO DEFECTIVE 2454 {ECO:0000303|PubMed:20675571} (At3g18290)		1.397
Cs8g06440		BTB/POZ domain-containing protein At1g63850 (At1g63850)		-2.142

Cs8g15610	K10144 RING finger and CHY zinc finger domain-containing protein 1 [EC:2.3.2.27] | (RefSeq) E3 ubiquitin-protein ligase MIEL1 (A)	E3 ubiquitin-protein ligase RZFP34 {ECO:0000305}; EC=2.3.2.27 {ECO:0000269|PubMed:26508764}; CHY zinc-finger and RING protein 1 {ECO:0000303|PubMed:26508764}; RING zinc-finger protein 34 {ECO:0000305}; RZFP34 protein homolog {ECO:0000303|PubMed:25002225}; AtRZPF34 {ECO:0000303|PubMed:25002225} (At5g22920)	-1.632	
Cs8g18240	K03283 heat shock 70kDa protein 1/2/6/8 | (RefSeq) probable mediator of RNA polymerase II transcription subunit 37c (A)	Probable mediator of RNA polymerase II transcription subunit 37c; Heat shock 70 kDa protein 4; Heat shock cognate 70 kDa protein 4; Heat shock cognate protein 70-4; AtHsc70-4; Heat shock protein 70-4; AtHsp70-4 (At3g12580)		1.617
Cs9g08200	K15503 serine/threonine-protein phosphatase 6 regulatory ankyrin repeat subunit B | (RefSeq) uncharacterized protein LOC110673220 (A)	Protein ACCELERATED CELL DEATH 6 {ECO:0000303|PubMed:10488236} (At4g03500)		1.263
Cs9g10770	K04733 interleukin-1 receptor-associated kinase 4 [EC:2.7.11.1] | (RefSeq) probable serine/threonine-protein kinase PBL11 (A)	F-box protein PP2-B10; Protein PHLOEM PROTEIN 2-LIKE B10; AtPP2-B10 (At2g02360)	1.008	1.547
novel.181	K10664 E3 ubiquitin-protein ligase ATL6/9/15/31/42/55 [EC:2.3.2.27] | (RefSeq) E3 ubiquitin-protein ligase ATL6-like (A)	ATL6_ARATH E3 ubiquitin-protein ligase ATL6; EC=2.3.2.27 {ECO:0000269|PubMed:15644464}; RING-H2 finger protein ATL6; RING-type E3 ubiquitin transferase ATL6 {ECO:0000305}; Flags: Precursor (At3g05200)	-1.711	-1.481
novel.1870	K22378 E3 ubiquitin-protein ligase RNF181 [EC:2.3.2.27] | (RefSeq) probable E3 ubiquitin-protein ligase RHY1A (A)	RNG1L_ARATH E3 ubiquitin-protein ligase RING1-like; EC=2.3.2.27; RING finger protein 1; RING-type E3 ubiquitin transferase RING1-like {ECO:0000305} (Hs22068658)		-2.374
novel.269	K04733 interleukin-1 receptor-associated kinase 4 [EC:2.7.11.1] | (RefSeq) LOC109787390; U-box domain-containing protein 12-like (A)	PUB8_ARATH U-box domain-containing protein 8; EC=2.3.2.27; Plant U-box protein 8; RING-type E3 ubiquitin transferase PUB8 {ECO:0000305} (At4g21350)		-1.142
novel.436		FK113_ARATH F-box/kelch-repeat protein At5g26960 (At5g26960)		-1.029
novel.725	K19037 E3 ubiquitin-protein ligase ATL23 [EC:2.3.2.27] | (RefSeq) E3 ubiquitin-protein ligase ATL23 (A)	ATL23_ARATH E3 ubiquitin-protein ligase ATL23; EC=2.3.2.27 {ECO:0000269|PubMed:15644464, ECO:0000269|PubMed:16339806}; RING-H2 finger protein ATL23; RING-type E3 ubiquitin transferase ATL23 {ECO:0000305} (At5g42200)	-1.079	
novel.894	K10664 E3 ubiquitin-protein ligase ATL6/9/15/31/42/55 [EC:2.3.2.27] | (RefSeq) putative RING-H2 finger protein ATL12 (A)	ATL42_ARATH E3 ubiquitin-protein ligase ATL42; EC=2.3.2.27 {ECO:0000269|PubMed:15644464}; RING-H2 finger protein ATL42; RING-type E3 ubiquitin transferase ATL42 {ECO:0000305}; Flags:		1.915

		Precursor (At4g28890)		
orange1.1t00304	K03188 urease accessory protein | (RefSeq) uncharacterized protein LOC108224146 isoform X1 (A)	F-box protein PP2-A13; Protein PHLOEM PROTEIN 2-LIKE A13; AtPP2-A13; SKP1-interacting partner 9 (At3g61060)		-1.394
orange1.1t00397	K16281 RING-H2 zinc finger protein RHA1 | (RefSeq) hypothetical protein (A)	E3 ubiquitin-protein ligase RHA1B {ECO:0000305}; EC=2.3.2.27; RING-H2 finger A1b {ECO:0000303|PubMed:9781696}; RING-H2 zinc finger protein RHA1b {ECO:0000305}; RING-type E3 ubiquitin transferase RHA1B {ECO:0000305} (At3g61460)		-1.272
orange1.1t02067	K03094 S-phase kinase-associated protein 1 | (RefSeq) hypothetical protein (A)	SKP1-like protein 11; AtSK11 (At4g34210)	1.002	3.273
orange1.1t03837	K10664 E3 ubiquitin-protein ligase ATL6/9/15/31/42/55 [EC:2.3.2.27] | (RefSeq) E3 ubiquitin-protein ligase ATL42-like (A)	E3 ubiquitin-protein ligase ATL42; EC=2.3.2.27 {ECO:0000269|PubMed:15644464}; RING-H2 finger protein ATL42; RING-type E3 ubiquitin transferase ATL42 {ECO:0000305}; Flags: Precursor (At4g28890)		1.114
orange1.1t04176	K03188 urease accessory protein | (RefSeq) uncharacterized protein LOC108224146 isoform X1 (A)	F-box protein PP2-B10; Protein PHLOEM PROTEIN 2-LIKE B10; AtPP2-B10 (At2g02360)		-1.676
Protein processing in endoplasmic reticulum [ko04141; P = 0.9756 (P3CR vs. P3R) and 0.1829 (P5CR vs. P5R)]				
Cs1g16780	K13993 HSP20 family protein | (RefSeq) 17.4 kDa class III heat shock protein (A)	17.4 kDa class III heat shock protein; 17.4 kDa heat shock protein 2; AtHsp17.4B (At1g54050)	1.083	
Cs2g08140	K10950 ERO1-like protein alpha [EC:1.8.4.-] | (RefSeq) endoplasmic reticulum oxidoreductin-2-like (A)	Endoplasmic reticulum oxidoreductin-1; EC=1.8.4.-; Flags: Precursor (At1g72280)	-1.031	
Cs2g20930	K14016 ubiquitin fusion degradation protein 1 | (RefSeq) uncharacterized protein LOC107496751 (A)	(At4g15420_2)		-1.921
Cs2g28910	K13993 HSP20 family protein | (RefSeq) hypothetical protein (A)	Protein RESTRICTED TEV MOVEMENT 2; Alpha-crystallin domains containing protein 41.3; AtAcd41.3; Restricted tobacco etch virus movement protein 2 (At2g27140)		-1.189
Cs3g04490	K03094 S-phase kinase-associated protein 1 | (RefSeq) uncharacterized LOC107176092 (A)			1.586
Cs3g14220	K09490 heat shock 70kDa protein 5 | (RefSeq) hypothetical protein (A)	Mediator of RNA polymerase II transcription subunit 37f; Heat shock 70 kDa protein 12; Heat shock protein 70-12; AtHsp70-12; Luminal-binding protein 2; AtBP2; BiP2; Flags: Precursor (At5g42020)		1.479
Cs3g18580	K03283 heat shock 70kDa protein 1/2/6/8 | (RefSeq) heat shock 70 kDa protein 18-like (A)	(At2g16050)		1.114

Cs4g05880	K13993 HSP20 family protein | (RefSeq) 17.7 kDa class II heat shock protein (A)	17.7 kDa class II heat shock protein; 17.7 kDa heat shock protein; AtHsp17.7 (At5g12030)		2.404
Cs5g03150	K04079 molecular chaperone HtpG | (RefSeq) heat shock protein 83-like (A)	Heat shock protein 83 (At5g52640)	1.176	
Cs5g05840	K13525 transitional endoplasmic reticulum ATPase | (RefSeq) cell division cycle protein 48 homolog (A)	Cell division cycle protein 48 homolog; Valosin-containing protein homolog; VCP (At5g03340)	1.624	
Cs5g08400	K03347 cullin 1 | (RefSeq) cullin-1 (A)	RING-H2 finger protein ATL22; EC=2.3.2.27 {ECO:0000305}; RING-type E3 ubiquitin transferase ATL22 {ECO:0000305}; Flags: Precursor (At5g53110)	2.485	
Cs5g18500	K13993 HSP20 family protein | (RefSeq) 22.0 kDa heat shock protein (A)	22.0 kDa heat shock protein; AtHsp22.0; Flags: Precursor (At4g10250)	2.028	1.313
Cs5g23960	K03094 S-phase kinase-associated protein 1 | (Kazusa) Lj2g3v3084320.1; - (A)	Protein FEZ; NAC domain-containing protein 9; ANAC009 (At1g26870)	1.289	1.083
Cs5g26940	K13993 HSP20 family protein | (RefSeq) HSP20-like chaperones superfamily protein (A)	Protein RESTRICTED TEV MOVEMENT 2; Alpha-crystallin domains containing protein 41.3; AtAcd41.3; Restricted tobacco etch virus movement protein 2 (At1g54400)		-1.313
Cs6g08940	K09555 BCL2-associated athanogene 1 | (RefSeq) BAG family molecular chaperone regulator 4-like isoform X1 (A)	BAG family molecular chaperone regulator 4; Bcl-2-associated athanogene 4 (At3g51780)		-1.103
Cs6g21270	K10661 E3 ubiquitin-protein ligase MARCH6 [EC:2.3.2.27] | (RefSeq) probable E3 ubiquitin ligase SUD1 (A)	Probable E3 ubiquitin ligase SUD1; EC=2.3.2.27; Protein ECERIFERUM 9; Protein SUPPRESSOR OF DRY2 DEFFECTS 1; AtSUD1; RING-type E3 ubiquitin transferase SUD1 {ECO:0000305}; RING/U-box domain-containing protein (At2g02960)		-1.401
Cs7g13460	K03868 RING-box protein 1 [EC:2.3.2.32] | (RefSeq) uncharacterized LOC100792815 (A)	GEM-like protein 4 (At5g08350)	-1.645	-4.260
Cs7g13465	K03868 RING-box protein 1 [EC:2.3.2.32] | (RefSeq) uncharacterized LOC100792815 (A)	GEM-like protein 4 (At5g08350)		-2.013
Cs7g23870	K13993 HSP20 family protein | (RefSeq) hypothetical protein (A)	Inactive protein RESTRICTED TEV MOVEMENT 2; Inactive restricted tobacco etch virus movement protein 2 (At5g04890)	-1.179	-2.214
Cs7g23890	K13993 HSP20 family protein | (RefSeq) inactive protein RESTRICTED TEV MOVEMENT 2-like (A)	Protein RESTRICTED TEV MOVEMENT 2; Alpha-crystallin domains containing protein 41.3; AtAcd41.3; Restricted tobacco etch virus movement protein 2 (At5g04890)		-1.170
Cs7g29020	K03283 heat shock 70kDa protein 1/2/6/8 | (RefSeq) hypothetical protein (A)	Heat shock cognate 70 kDa protein (At5g02500)		1.521

Cs7g29030	K03283 heat shock 70kDa protein 1/2/6/8 | (RefSeq) hypothetical protein (A)	(At2g16050)	1.503	1.007
Cs7g29040	K03283 heat shock 70kDa protein 1/2/6/8 | (RefSeq) heat shock 70 kDa protein 18-like (A)	Heat shock cognate 70 kDa protein (At5g02500)	1.029	
Cs7g29170	K03283 heat shock 70kDa protein 1/2/6/8 | (RefSeq) heat shock cognate 70 kDa protein 2-like isoform X1 (A)	Probable nucleoredoxin 1-2; OsNrx1-2; EC=1.8.1.8 (At2g16050)	2.726	2.927
Cs7g29190	K03283 heat shock 70kDa protein 1/2/6/8 | (RefSeq) heat shock cognate 70 kDa protein 2-like isoform X1 (A)	Probable nucleoredoxin 1-2; OsNrx1-2; EC=1.8.1.8 (At2g16050)	4.565	
Cs7g32260	K13993 HSP20 family protein | (RefSeq) 18.1 kDa class I heat shock protein-like (A)	18.1 kDa class I heat shock protein; 18.1 kDa heat shock protein; AtHsp18.1 (At5g59720)		-4.082
Cs8g18020	K13993 HSP20 family protein | (RefSeq) hypothetical protein (A)	17.6 kDa class I heat shock protein 3; 17.6 kDa heat shock protein 3; AtHsp17.6C (At1g53540)	1.720	1.980
Cs8g18240	K03283 heat shock 70kDa protein 1/2/6/8 | (RefSeq) probable mediator of RNA polymerase II transcription subunit 37c (A)	Probable mediator of RNA polymerase II transcription subunit 37c; Heat shock 70 kDa protein 4; Heat shock cognate 70 kDa protein 4; Heat shock cognate protein 70-4; AtHsc70-4; Heat shock protein 70-4; AtHsp70-4 (At3g12580)		1.617
Cs8g18360	K13993 HSP20 family protein | (RefSeq) hypothetical protein (A)	17.4 kDa class I heat shock protein; 17.4 kDa heat shock protein 1; AtHsp17.4A (At3g46230)	2.109	1.307
Cs9g02500	K07953 GTP-binding protein SAR1 [EC:3.6.5.-] | (RefSeq) GTP-binding protein SAR1A-like (A)	GTP-binding protein SAR1A (At4g02080)	1.173	
novel.1609	K03283 heat shock 70kDa protein 1/2/6/8 | (RefSeq) heat shock 70 kDa protein 18-like (A)	HSP70_SOYBN Heat shock 70 kDa protein (At1g56410)		1.504
novel.2184	K03094 S-phase kinase-associated protein 1 | (RefSeq) SKP1-like protein 14 (A)	Y3720_ARATH UPF0481 protein At3g47200 (At4g31980_2)		1.665
novel.2282	K03283 heat shock 70kDa protein 1/2/6/8 | (RefSeq) heat shock 70 kDa protein (A)	HSP70_SOYBN Heat shock 70 kDa protein (At1g16030)	2.899	1.100
novel.2575	K04079 molecular chaperone HtpG | (RefSeq) heat shock cognate protein HSP 90-beta-like (A)	POLX_TOBAC Retrovirus-related Pol polyprotein from transposon TNT 1-94; Includes: Protease; EC=3.4.23.-; Includes: Reverse transcriptase; EC=2.7.7.49; Includes: Endonuclease (At4g03810)		1.801
novel.2837	K03283 heat shock 70kDa protein 1/2/6/8 | (RefSeq) heat shock 70 kDa protein (A)	RNHX1_ARATH Putative ribonuclease H protein At1g65750; EC=3.1.26.4 (At1g17390)		1.493
novel.789	K13719 ubiquitin thioesterase OTU1 [EC:3.1.2.-] | (RefSeq) ubiquitin thioesterase OTU1 (A)			1.645

orange1.1t02067	K03094 S-phase kinase-associated protein 1 | (RefSeq) hypothetical protein (A)	SKP1-like protein 11; AtSK11 (At4g34210)	1.002	3.273
orange1.1t02795	K03283 heat shock 70kDa protein 1/2/6/8 | (RefSeq) heat shock 70 kDa protein 5-like (A)	Heat shock 70 kDa protein 5; Heat shock protein 70-5; AtHsp70-5; Heat shock protein 70b (At1g16030)	1.466	2.190
orange1.1t03606	K13525 transitional endoplasmic reticulum ATPase | (RefSeq) probable 2-oxoacid dependent dioxygenase (A)	1-aminocyclopropane-1-carboxylate oxidase homolog; Protein E8 (At2g25450)		1.019
orange1.1t04042	K03283 heat shock 70kDa protein 1/2/6/8 | (RefSeq) heat shock cognate 70 kDa protein 2-like isoform X1 (A)	(At1g20990)	1.318	
orange1.1t04546	K04374 cyclic AMP-dependent transcription factor ATF-4 | (RefSeq) basic leucine zipper 61-like (A)	Basic leucine zipper 61 {ECO:0000303|PubMed:11906833}; AtbZIP61 {ECO:0000303|PubMed:11906833}; bZIP protein 61 (At3g58120)		
orange1.1t05692	K09503 DnaJ homolog subfamily A member 2 | (RefSeq) molecular chaperone DnaJ (A)	Chaperone protein dnaJ C76, chloroplastic {ECO:0000305}; atDjC76 {ECO:0000303|PubMed:23894646}; AtDjC17 {ECO:0000303|PubMed:25339971}; AtJ17 {ECO:0000305}; Flags: Precursor (At5g23240)		5.982
Proteasome-mediated ubiquitin-dependent protein catabolic process [GO:0043161; P = (P3CR vs. P3R) and 0.9657 (P5CR vs. P5R)]				
Cs1g14060	K04733 interleukin-1 receptor-associated kinase 4 [EC:2.7.11.1] | (RefSeq) probable receptor-like protein kinase At1g30570 (A)	F-box/kelch-repeat protein At1g15670 (At1g15670)	-2.562	-1.267
Cs2g10710	K19042 E3 ubiquitin-protein ligase BOI and related proteins [EC:2.3.2.27] | (RefSeq) BOI-related E3 ubiquitin-protein ligase 1 (A)	BOI-related E3 ubiquitin-protein ligase 1; EC=2.3.2.27 {ECO:0000269|PubMed:20921156}; RING-type E3 ubiquitin transferase BRG1 {ECO:0000305} (At5g45100)		-1.292
Cs2g15840	K19038 E3 ubiquitin-protein ligase ATL41 [EC:2.3.2.27] | (RefSeq) E3 ubiquitin-protein ligase ATL41-like (A)	E3 ubiquitin-protein ligase ATL41; EC=2.3.2.27 {ECO:0000269|PubMed:15644464, ECO:0000269|PubMed:16339806}; RING-H2 finger protein ATL41; RING-type E3 ubiquitin transferase ATL41 {ECO:0000305} (At2g42360)		1.014
Cs2g24740		Kelch repeat-containing protein At3g27220 (At3g27220)	-2.054	
Cs2g28510	K19041 E3 ubiquitin-protein ligase RNF38/44 [EC:2.3.2.27] | (RefSeq) RING-H2 finger protein ATL46 isoform X1 (A)	RING-H2 finger protein ATL46; EC=2.3.2.27 {ECO:0000305}; RING-type E3 ubiquitin transferase ATL46 {ECO:0000305} (At5g40250)	1.009	
Cs3g19900	K19045 E3 ubiquitin-protein ligase BIG BROTHER and related proteins [EC:2.3.2.27] | (RefSeq) E3 ubiquitin ligase BIG BROTHER-related-like (A)	E3 ubiquitin ligase BIG BROTHER-related; AtBBR; EC=2.3.2.27 {ECO:0000250|UniProtKB:Q8L649}; RING-type E3 ubiquitin transferase BIG BROTHER-related {ECO:0000305} (At3g47180)	-1.274	

Cs3g26440	K19042 E3 ubiquitin-protein ligase BOI and related proteins [EC:2.3.2.27] | (RefSeq) probable BOI-related E3 ubiquitin-protein ligase 2 (A)	BOI-related E3 ubiquitin-protein ligase 1; EC=2.3.2.27 {ECO:0000269|PubMed:20921156}; RING-type E3 ubiquitin transferase BRG1 {ECO:0000305} (At4g35070)	-2.165	
Cs4g01190	K22378 E3 ubiquitin-protein ligase RNF181 [EC:2.3.2.27] | (RefSeq) zinc finger family protein (A)	Probable E3 ubiquitin-protein ligase RHY1A {ECO:0000305}; EC=2.3.2.27; RING-H2 finger Y1a {ECO:0000303|PubMed:9781696}; RING-H2 zinc finger protein RHY1a {ECO:0000305}; RING-type E3 ubiquitin transferase RHY1A {ECO:0000305} (At2g15580)	1.691	
Cs4g07940	K10523 speckle-type POZ protein | (RefSeq) BTB/POZ domain-containing protein At1g55760 (A)	BTB/POZ domain-containing protein At1g55760 (At1g55760)		1.109
Cs5g02250	K19041 E3 ubiquitin-protein ligase RNF38/44 [EC:2.3.2.27] | (RefSeq) RING-H2 finger protein ATL20-like (A)	RING-H2 finger protein ATL22; EC=2.3.2.27 {ECO:0000305}; RING-type E3 ubiquitin transferase ATL22 {ECO:0000305}; Flags: Precursor (At5g53110)		2.318
Cs5g02260	K19041 E3 ubiquitin-protein ligase RNF38/44 [EC:2.3.2.27] | (RefSeq) RING-H2 finger protein ATL20-like (A)	Putative RING-H2 finger protein ATL21A; EC=2.3.2.27 {ECO:0000305}; RING-type E3 ubiquitin transferase ATL21A {ECO:0000305}; Flags: Precursor (At5g07040)	1.619	1.969
Cs5g03280	K16281 RING-H2 zinc finger protein RHA1 | (RefSeq) hypothetical protein (A)	E3 ubiquitin-protein ligase RHA1B {ECO:0000305}; EC=2.3.2.27; RING-H2 finger A1b {ECO:0000303|PubMed:9781696}; RING-H2 zinc finger protein RHA1b {ECO:0000305}; RING-type E3 ubiquitin transferase RHA1B {ECO:0000305} (At3g61460)	-1.059	-1.083
Cs5g04060	K04733 interleukin-1 receptor-associated kinase 4 [EC:2.7.11.1] | (RefSeq) probable receptor-like protein kinase At1g30570 (A)	F-box/kelch-repeat protein SKIP11; SKP1-interacting partner 11 (At2g02870)	-1.566	
Cs5g08400	K03347 cullin 1 | (RefSeq) cullin-1 (A)	RING-H2 finger protein ATL22; EC=2.3.2.27 {ECO:0000305}; RING-type E3 ubiquitin transferase ATL22 {ECO:0000305}; Flags: Precursor (At5g53110)	2.485	
Cs5g23550	K11975 E3 ubiquitin-protein ligase RNF144 [EC:2.3.2.31] | (RefSeq) probable E3 ubiquitin-protein ligase RNF217 (A)	Putative E3 ubiquitin-protein ligase ARI4; EC=2.3.2.27 {ECO:0000250|UniProtKB:Q9Y4X5}; ARIADNE-like protein ARI4; Protein ariadne homolog 4; RING-type E3 ubiquitin transferase ARI4 {ECO:0000305} (At3g53690)	1.062	1.428
Cs5g29440	K16275 E3 ubiquitin-protein ligase BAH [EC:2.3.2.27] | (RefSeq) hypothetical protein (A)	E3 ubiquitin-protein ligase BAH1; EC=2.3.2.27 {ECO:0000305}; Protein BENZOIC ACID HYPERSENSITIVE 1; Protein NITROGEN LIMITATION ADAPTATION; RING-type E3 ubiquitin transferase BAH1 {ECO:0000305} (At1g02860)		1.226
Cs5g29920	K19041 E3 ubiquitin-protein ligase RNF38/44 [EC:2.3.2.27] |	RING-H2 finger protein ATL16; EC=2.3.2.27 {ECO:0000305};	-1.449	-1.378

	(RefSeq) RING-H2 finger protein ATL16-like (A)	RING-type E3 ubiquitin transferase ATL16 {ECO:0000305} (At5g43420)		
Cs5g33380	K19041 E3 ubiquitin-protein ligase RNF38/44 [EC:2.3.2.27] | (Kazusa) Lj6g3v1468220.1; - (A)	RING-H2 finger protein ATL64; EC=2.3.2.27 {ECO:0000305}; RING-type E3 ubiquitin transferase ATL64 {ECO:0000305} (At2g47560)	-1.005	
Cs6g09980	K19040 E3 ubiquitin-protein ligase ATL10/75/76/77/78 [EC:2.3.2.27] | (RefSeq) RING-H2 finger protein ATL74-like (A)	RING-H2 finger protein ATL74; EC=2.3.2.27 {ECO:0000305}; RING-type E3 ubiquitin transferase ATL74 {ECO:0000305} (At5g01880)	-1.043	
Cs6g12970	K12830 splicing factor 3B subunit 3 | (RefSeq) uncharacterized LOC102624787 (A)	DNA damage-binding protein 1; High pigmentation protein 1; UV-damaged DNA-binding protein 1 (At3g11960)		1.100
Cs6g13530	K19041 E3 ubiquitin-protein ligase RNF38/44 [EC:2.3.2.27] | (RefSeq) RING-H2 finger protein ATL70-like (A)	Putative RING-H2 finger protein ATL71; EC=2.3.2.27 {ECO:0000305}; RING-type E3 ubiquitin transferase ATL71 {ECO:0000305} (At5g06490)	-1.017	
Cs6g21270	K10661 E3 ubiquitin-protein ligase MARCH6 [EC:2.3.2.27] | (RefSeq) probable E3 ubiquitin ligase SUD1 (A)	Probable E3 ubiquitin ligase SUD1; EC=2.3.2.27; Protein ECERIFERUM 9; Protein SUPPRESSOR OF DRY2 DEFFECTS 1; AtSUD1; RING-type E3 ubiquitin transferase SUD1 {ECO:0000305}; RING/U-box domain-containing protein (At2g02960)		-1.401
Cs7g08960		F-box/kelch-repeat protein At1g23390 (At1g23390)		-2.637
Cs7g09440	K16285 RING/U-box domain-containing protein [EC:2.3.2.27] | (RefSeq) hypothetical protein (A)	Probable E3 ubiquitin-protein ligase XERICO; EC=2.3.2.27; RING-type E3 ubiquitin transferase XERICO (At2g04240)	1.393	1.416
Cs7g14590	K15030 translation initiation factor 3 subunit M | (RefSeq) uncharacterized LOC103856145 (A)	F-box protein CPR1; Protein CONSTITUTIVE EXPRESSER OF PR GENES 1; Protein CONSTITUTIVE EXPRESSER OF PR GENES 30 {ECO:0000303|PubMed:19682297} (At4g12560)		-1.003
Cs7g21290	K10523 speckle-type POZ protein | (RefSeq) BTB/POZ domain-containing protein At3g56230-like (A)	BTB/POZ domain-containing protein At3g56230 (At3g56230)		-2.108
Cs7g30840	K19038 E3 ubiquitin-protein ligase ATL41 [EC:2.3.2.27] | (RefSeq) E3 ubiquitin-protein ligase ATL41-like (A)	RING-H2 finger protein ATL66; EC=2.3.2.27 {ECO:0000305}; RING-type E3 ubiquitin transferase ATL66 {ECO:0000305} (At3g11110)		-2.823
Cs9g18010	K19041 E3 ubiquitin-protein ligase RNF38/44 [EC:2.3.2.27] | (RefSeq) RING-H2 finger protein ATL20-like (A)	Putative RING-H2 finger protein ATL21A; EC=2.3.2.27 {ECO:0000305}; RING-type E3 ubiquitin transferase ATL21A {ECO:0000305}; Flags: Precursor (At5g53110)		1.514
novel.181	K10664 E3 ubiquitin-protein ligase ATL6/9/15/31/42/55 [EC:2.3.2.27] | (RefSeq) E3 ubiquitin-protein ligase ATL6-like (A)	ATL6_ARATH E3 ubiquitin-protein ligase ATL6; EC=2.3.2.27 {ECO:0000269|PubMed:15644464}; RING-H2 finger protein ATL6; RING-type E3 ubiquitin transferase ATL6 {ECO:0000305}; Flags:	-1.711	-1.481

		Precursor (At3g05200)		
novel.1832		FK126_ARATH F-box/kelch-repeat protein At1g15670 (At1g15670)		-4.013
novel.1870	K22378 E3 ubiquitin-protein ligase RNF181 [EC:2.3.2.27] | (RefSeq) probable E3 ubiquitin-protein ligase RHY1A (A)	RNG1L_ARATH E3 ubiquitin-protein ligase RING1-like; EC=2.3.2.27; RING finger protein 1; RING-type E3 ubiquitin transferase RING1-like {ECO:0000305} (Hs22068658)		-2.374
novel.2729	K10664 E3 ubiquitin-protein ligase ATL6/9/15/31/42/55 [EC:2.3.2.27] | (RefSeq) hypothetical protein (A)	ATL31_ARATH E3 ubiquitin-protein ligase ATL31; EC=2.3.2.27 {ECO:0000269|PubMed:19702666}; Protein CARBON/NITROGEN INSENSITIVE 1; Protein SUPER SURVIVAL 1; RING-H2 finger protein ATL31; RING-type E3 ubiquitin transferase ATL31 {ECO:0000305}; Flags: Precursor (At5g27420)	-1.413	-1.732
novel.35	K19040 E3 ubiquitin-protein ligase ATL10/75/76/77/78 [EC:2.3.2.27] | (RefSeq) RING-H2 finger protein ATL10-like (A)	ATL78_ARATH RING-H2 finger protein ATL78; EC=2.3.2.27 {ECO:0000305}; RING-type E3 ubiquitin transferase ATL78 {ECO:0000305} (At1g49230)		-1.028
novel.659	K19041 E3 ubiquitin-protein ligase RNF38/44 [EC:2.3.2.27] | (RefSeq) RING-H2 finger protein ATL54-like (A)	ATL54_ARATH RING-H2 finger protein ATL54; EC=2.3.2.27 {ECO:0000305}; RING-type E3 ubiquitin transferase ATL54 {ECO:0000305} (At1g72220)	1.752	3.520
novel.725	K19037 E3 ubiquitin-protein ligase ATL23 [EC:2.3.2.27] | (RefSeq) E3 ubiquitin-protein ligase ATL23 (A)	ATL23_ARATH E3 ubiquitin-protein ligase ATL23; EC=2.3.2.27 {ECO:0000269|PubMed:15644464, ECO:0000269|PubMed:16339806}; RING-H2 finger protein ATL23; RING-type E3 ubiquitin transferase ATL23 {ECO:0000305} (At5g42200)	-1.079	
novel.894	K10664 E3 ubiquitin-protein ligase ATL6/9/15/31/42/55 [EC:2.3.2.27] | (RefSeq) putative RING-H2 finger protein ATL12 (A)	ATL42_ARATH E3 ubiquitin-protein ligase ATL42; EC=2.3.2.27 {ECO:0000269|PubMed:15644464}; RING-H2 finger protein ATL42; RING-type E3 ubiquitin transferase ATL42 {ECO:0000305}; Flags: Precursor (At4g28890)		1.915
novel.911	K19041 E3 ubiquitin-protein ligase RNF38/44 [EC:2.3.2.27] | (RefSeq) RING-H2 finger protein ATL20-like (A)	ATL69_ARATH Putative RING-H2 finger protein ATL69; EC=2.3.2.27 {ECO:0000305}; RING-type E3 ubiquitin transferase ATL69 {ECO:0000305} (At5g07040)	1.230	1.277
orange1.1t00397	K16281 RING-H2 zinc finger protein RHA1 | (RefSeq) hypothetical protein (A)	E3 ubiquitin-protein ligase RHA1B {ECO:0000305}; EC=2.3.2.27; RING-H2 finger A1b {ECO:0000303|PubMed:9781696}; RING-H2 zinc finger protein RHA1b {ECO:0000305}; RING-type E3 ubiquitin transferase RHA1B {ECO:0000305} (At3g61460)		-1.272
orange1.1t01964	K19041 E3 ubiquitin-protein ligase RNF38/44 [EC:2.3.2.27] | (RefSeq) E3 ubiquitin-protein ligase RING1 (A)	RING-H2 finger protein ATL54; EC=2.3.2.27 {ECO:0000305}; RING-type E3 ubiquitin transferase ATL54 {ECO:0000305} (At1g72220)		-1.235

orange1.1t03837	K10664 E3 ubiquitin-protein ligase ATL6/9/15/31/42/55 [EC:2.3.2.27] | (RefSeq) E3 ubiquitin-protein ligase ATL42-like (A)	E3 ubiquitin-protein ligase ATL42; EC=2.3.2.27 {ECO:0000269|PubMed:15644464}; RING-H2 finger protein ATL42; RING-type E3 ubiquitin transferase ATL42 {ECO:0000305}; Flags: Precursor (At4g28890)		1.114
Ubiquitin-dependent protein catabolic process [GO:0006511; P = 0.9350 (P3CR vs. P3R) and 0.9971 (P5CR vs. P5R)]				
Cs1g05020	K19040 E3 ubiquitin-protein ligase ATL10/75/76/77/78 [EC:2.3.2.27] | (RefSeq) hypothetical protein (A)	RING-H2 finger protein ATL78; EC=2.3.2.27 {ECO:0000305}; RING-type E3 ubiquitin transferase ATL78 {ECO:0000305} (At1g49230)		1.848
Cs1g05090	K19040 E3 ubiquitin-protein ligase ATL10/75/76/77/78 [EC:2.3.2.27] | (RefSeq) hypothetical protein (A)	RING-H2 finger protein ATL75; EC=2.3.2.27 {ECO:0000305}; RING-type E3 ubiquitin transferase ATL75 {ECO:0000305} (At1g49200)		
Cs1g14060	K04733 interleukin-1 receptor-associated kinase 4 [EC:2.7.11.1] | (RefSeq) probable receptor-like protein kinase At1g30570 (A)	F-box/kelch-repeat protein At1g15670 (At1g15670)	-2.562	-1.267
Cs2g06250		E3 ubiquitin-protein ligase AIRP2 {ECO:0000305}; EC=2.3.2.27 {ECO:0000269|PubMed:21969385}; Protein ABA INSENSITIVE RING PROTEIN 2 {ECO:0000303|PubMed:21969385}; AtAIRP2 {ECO:0000303|PubMed:21969385}; RING-type E3 ubiquitin transferase AIRP2 {ECO:0000305} (At3g47160)		-1.730
Cs2g10710	K19042 E3 ubiquitin-protein ligase BOI and related proteins [EC:2.3.2.27] | (RefSeq) BOI-related E3 ubiquitin-protein ligase 1 (A)	BOI-related E3 ubiquitin-protein ligase 1; EC=2.3.2.27 {ECO:0000269|PubMed:20921156}; RING-type E3 ubiquitin transferase BRG1 {ECO:0000305} (At5g45100)		-1.292
Cs2g11740	K02728 20S proteasome subunit alpha 3 [EC:3.4.25.1] | (RefSeq) hypothetical protein (A)	Proteasome subunit alpha type-4; EC=3.4.25.1; 20S proteasome alpha subunit C; 20S proteasome subunit alpha-3; Proteasome 27 kDa subunit (At3g22110)	-1.829	
Cs2g15840	K19038 E3 ubiquitin-protein ligase ATL41 [EC:2.3.2.27] | (RefSeq) E3 ubiquitin-protein ligase ATL41-like (A)	E3 ubiquitin-protein ligase ATL41; EC=2.3.2.27 {ECO:0000269|PubMed:15644464, ECO:0000269|PubMed:16339806}; RING-H2 finger protein ATL41; RING-type E3 ubiquitin transferase ATL41 {ECO:0000305} (At2g42360)		1.014
Cs2g20930	K14016 ubiquitin fusion degradation protein 1 | (RefSeq) uncharacterized protein LOC107496751 (A)	(At4g15420_2)		-1.921
Cs2g24740		Kelch repeat-containing protein At3g27220 (At3g27220)	-2.054	
Cs2g28510	K19041 E3 ubiquitin-protein ligase RNF38/44 [EC:2.3.2.27] |	RING-H2 finger protein ATL46; EC=2.3.2.27 {ECO:0000305};	1.009	

	(RefSeq) RING-H2 finger protein ATL46 isoform X1 (A)	RING-type E3 ubiquitin transferase ATL46 {ECO:0000305} (At5g40250)		
Cs3g19900	K19045 E3 ubiquitin-protein ligase BIG BROTHER and related proteins [EC:2.3.2.27] | (RefSeq) E3 ubiquitin ligase BIG BROTHER-related-like (A)	E3 ubiquitin ligase BIG BROTHER-related; AtBBR; EC=2.3.2.27 {ECO:0000250|UniProtKB:Q8L649}; RING-type E3 ubiquitin transferase BIG BROTHER-related {ECO:0000305} (At3g47180)	-1.274	
Cs3g26440	K19042 E3 ubiquitin-protein ligase BOI and related proteins [EC:2.3.2.27] | (RefSeq) probable BOI-related E3 ubiquitin-protein ligase 2 (A)	BOI-related E3 ubiquitin-protein ligase 1; EC=2.3.2.27 {ECO:0000269|PubMed:20921156}; RING-type E3 ubiquitin transferase BRG1 {ECO:0000305} (At4g35070)	-2.165	
Cs4g01190	K22378 E3 ubiquitin-protein ligase RNF181 [EC:2.3.2.27] | (RefSeq) zinc finger family protein (A)	Probable E3 ubiquitin-protein ligase RHY1A {ECO:0000305}; EC=2.3.2.27; RING-H2 finger Y1a {ECO:0000303|PubMed:9781696}; RING-H2 zinc finger protein RHY1a {ECO:0000305}; RING-type E3 ubiquitin transferase RHY1A {ECO:0000305} (At2g15580)	1.691	
Cs4g07940	K10523 speckle-type POZ protein | (RefSeq) BTB/POZ domain-containing protein At1g55760 (A)	BTB/POZ domain-containing protein At1g55760 (At1g55760)		1.109
Cs4g11160	K08770 ubiquitin C | (RefSeq) polyubiquitin 11 (A)	Polyubiquitin 11; Contains: Ubiquitin; Flags: Precursor (At4g05050)		1.888
Cs5g02250	K19041 E3 ubiquitin-protein ligase RNF38/44 [EC:2.3.2.27] | (RefSeq) RING-H2 finger protein ATL20-like (A)	RING-H2 finger protein ATL22; EC=2.3.2.27 {ECO:0000305}; RING-type E3 ubiquitin transferase ATL22 {ECO:0000305}; Flags: Precursor (At5g53110)		2.318
Cs5g02260	K19041 E3 ubiquitin-protein ligase RNF38/44 [EC:2.3.2.27] | (RefSeq) RING-H2 finger protein ATL20-like (A)	Putative RING-H2 finger protein ATL21A; EC=2.3.2.27 {ECO:0000305}; RING-type E3 ubiquitin transferase ATL21A {ECO:0000305}; Flags: Precursor (At5g07040)	1.619	1.969
Cs5g03280	K16281 RING-H2 zinc finger protein RHA1 | (RefSeq) hypothetical protein (A)	E3 ubiquitin-protein ligase RHA1B {ECO:0000305}; EC=2.3.2.27; RING-H2 finger A1b {ECO:0000303|PubMed:9781696}; RING-H2 zinc finger protein RHA1b {ECO:0000305}; RING-type E3 ubiquitin transferase RHA1B {ECO:0000305} (At3g61460)	-1.059	-1.083
Cs5g04060	K04733 interleukin-1 receptor-associated kinase 4 [EC:2.7.11.1] | (RefSeq) probable receptor-like protein kinase At1g30570 (A)	F-box/kelch-repeat protein SKIP11; SKP1-interacting partner 11 (At2g02870)	-1.566	
Cs5g08270	K05609 ubiquitin carboxyl-terminal hydrolase L3 [EC:3.4.19.12] | (RefSeq) ubiquitin carboxyl-terminal hydrolase isozyme L3-like (A)	Ubiquitin carboxyl-terminal hydrolase 3 {ECO:0000303|PubMed:17559514}; EC=3.4.19.12 {ECO:0000305} (At4g17510)		-1.608
Cs5g08400	K03347 cullin 1 | (RefSeq) cullin-1 (A)	RING-H2 finger protein ATL22; EC=2.3.2.27 {ECO:0000305}; RING-type E3 ubiquitin transferase ATL22 {ECO:0000305}; Flags: Precursor (At5g53110)	2.485	

Cs5g23550	K11975 E3 ubiquitin-protein ligase RNF144 [EC:2.3.2.31] | (RefSeq) probable E3 ubiquitin-protein ligase RNF217 (A)	Putative E3 ubiquitin-protein ligase ARI4; EC=2.3.2.27 {ECO:0000250|UniProtKB:Q9Y4X5}; ARIADNE-like protein ARI4; Protein ariadne homolog 4; RING-type E3 ubiquitin transferase ARI4 {ECO:0000305} (At3g53690)	1.062	1.428
Cs5g29440	K16275 E3 ubiquitin-protein ligase BAH [EC:2.3.2.27] | (RefSeq) hypothetical protein (A)	E3 ubiquitin-protein ligase BAH1; EC=2.3.2.27 {ECO:0000305}; Protein BENZOIC ACID HYPERSENSITIVE 1; Protein NITROGEN LIMITATION ADAPTATION; RING-type E3 ubiquitin transferase BAH1 {ECO:0000305} (At1g02860)		1.226
Cs5g29920	K19041 E3 ubiquitin-protein ligase RNF38/44 [EC:2.3.2.27] | (RefSeq) RING-H2 finger protein ATL16-like (A)	RING-H2 finger protein ATL16; EC=2.3.2.27 {ECO:0000305}; RING-type E3 ubiquitin transferase ATL16 {ECO:0000305} (At5g43420)	-1.449	-1.378
Cs5g33380	K19041 E3 ubiquitin-protein ligase RNF38/44 [EC:2.3.2.27] | (Kazusa) Lj6g3v1468220.1; - (A)	RING-H2 finger protein ATL64; EC=2.3.2.27 {ECO:0000305}; RING-type E3 ubiquitin transferase ATL64 {ECO:0000305} (At2g47560)	-1.005	
Cs6g07180	K06700 proteasome inhibitor subunit 1 (PI31) | (RefSeq) probable proteasome inhibitor (A)	Probable proteasome inhibitor (At3g53970)		-1.172
Cs6g09980	K19040 E3 ubiquitin-protein ligase ATL10/75/76/77/78 [EC:2.3.2.27] | (RefSeq) RING-H2 finger protein ATL74-like (A)	RING-H2 finger protein ATL74; EC=2.3.2.27 {ECO:0000305}; RING-type E3 ubiquitin transferase ATL74 {ECO:0000305} (At5g01880)	-1.043	
Cs6g12970	K12830 splicing factor 3B subunit 3 | (RefSeq) uncharacterized LOC102624787 (A)	DNA damage-binding protein 1; High pigmentation protein 1; UV-damaged DNA-binding protein 1 (At3g11960)		1.100
Cs6g13530	K19041 E3 ubiquitin-protein ligase RNF38/44 [EC:2.3.2.27] | (RefSeq) RING-H2 finger protein ATL70-like (A)	Putative RING-H2 finger protein ATL71; EC=2.3.2.27 {ECO:0000305}; RING-type E3 ubiquitin transferase ATL71 {ECO:0000305} (At5g06490)	-1.017	
Cs6g21270	K10661 E3 ubiquitin-protein ligase MARCH6 [EC:2.3.2.27] | (RefSeq) probable E3 ubiquitin ligase SUD1 (A)	Probable E3 ubiquitin ligase SUD1; EC=2.3.2.27; Protein ECERIFERUM 9; Protein SUPPRESSOR OF DRY2 DEFFECTS 1; AtSUD1; RING-type E3 ubiquitin transferase SUD1 {ECO:0000305}; RING/U-box domain-containing protein (At2g02960)		-1.401
Cs7g05390	K14515 EIN3-binding F-box protein | (RefSeq) EIN3-binding F-box protein 1 (A)	EIN3-binding F-box protein 1; F-box/LRR-repeat protein 6 (At2g25490)		1.135
Cs7g08960		F-box/kelch-repeat protein At1g23390 (At1g23390)		-2.637
Cs7g09440	K16285 RING/U-box domain-containing protein [EC:2.3.2.27] | (RefSeq) hypothetical protein (A)	Probable E3 ubiquitin-protein ligase XERICO; EC=2.3.2.27; RING-type E3 ubiquitin transferase XERICO (At2g04240)	1.393	1.416
Cs7g14590	K15030 translation initiation factor 3 subunit M | (RefSeq)	F-box protein CPR1; Protein CONSTITUTIVE EXPRESSER OF PR		-1.003

	uncharacterized LOC103856145 (A)	GENES 1; Protein CONSTITUTIVE EXPRESSER OF PR GENES 30 {ECO:0000303|PubMed:19682297} (At4g12560)		
Cs7g21290	K10523 speckle-type POZ protein | (RefSeq) BTB/POZ domain-containing protein At3g56230-like (A)	BTB/POZ domain-containing protein At3g56230 (At3g56230)		-2.108
Cs7g30840	K19038 E3 ubiquitin-protein ligase ATL41 [EC:2.3.2.27] | (RefSeq) E3 ubiquitin-protein ligase ATL41-like (A)	RING-H2 finger protein ATL66; EC=2.3.2.27 {ECO:0000305}; RING-type E3 ubiquitin transferase ATL66 {ECO:0000305} (At3g11110)		-2.823
Cs8g20100	K03320 ammonium transporter, Amt family | (RefSeq) ammonium transporter 1 member 3-like (A)	E3 ubiquitin-protein ligase AIRP2 {ECO:0000305}; EC=2.3.2.27 {ECO:0000269|PubMed:21969385}; Protein ABA INSENSITIVE RING PROTEIN 2 {ECO:0000303|PubMed:21969385}; AtAIRP2 {ECO:0000303|PubMed:21969385}; RING-type E3 ubiquitin transferase AIRP2 {ECO:0000305} (At3g47160)	-1.609	
Cs9g18010	K19041 E3 ubiquitin-protein ligase RNF38/44 [EC:2.3.2.27] | (RefSeq) RING-H2 finger protein ATL20-like (A)	Putative RING-H2 finger protein ATL21A; EC=2.3.2.27 {ECO:0000305}; RING-type E3 ubiquitin transferase ATL21A {ECO:0000305}; Flags: Precursor (At5g53110)		1.514
novel.181	K10664 E3 ubiquitin-protein ligase ATL6/9/15/31/42/55 [EC:2.3.2.27] | (RefSeq) E3 ubiquitin-protein ligase ATL6-like (A)	ATL6_ARATH E3 ubiquitin-protein ligase ATL6; EC=2.3.2.27 {ECO:0000269|PubMed:15644464}; RING-H2 finger protein ATL6; RING-type E3 ubiquitin transferase ATL6 {ECO:0000305}; Flags: Precursor (At3g05200)	-1.711	-1.481
novel.1832		FK126_ARATH F-box/kelch-repeat protein At1g15670 (At1g15670)		-4.013
novel.1870	K22378 E3 ubiquitin-protein ligase RNF181 [EC:2.3.2.27] | (RefSeq) probable E3 ubiquitin-protein ligase RHY1A (A)	RNG1L_ARATH E3 ubiquitin-protein ligase RING1-like; EC=2.3.2.27; RING finger protein 1; RING-type E3 ubiquitin transferase RING1-like {ECO:0000305} (Hs22068658)		-2.374
novel.2729	K10664 E3 ubiquitin-protein ligase ATL6/9/15/31/42/55 [EC:2.3.2.27] | (RefSeq) hypothetical protein (A)	ATL31_ARATH E3 ubiquitin-protein ligase ATL31; EC=2.3.2.27 {ECO:0000269|PubMed:19702666}; Protein CARBON/NITROGEN INSENSITIVE 1; Protein SUPER SURVIVAL 1; RING-H2 finger protein ATL31; RING-type E3 ubiquitin transferase ATL31 {ECO:0000305}; Flags: Precursor (At5g27420)	-1.413	-1.732
novel.35	K19040 E3 ubiquitin-protein ligase ATL10/75/76/77/78 [EC:2.3.2.27] | (RefSeq) RING-H2 finger protein ATL10-like (A)	ATL78_ARATH RING-H2 finger protein ATL78; EC=2.3.2.27 {ECO:0000305}; RING-type E3 ubiquitin transferase ATL78 {ECO:0000305} (At1g49230)		-1.028
novel.659	K19041 E3 ubiquitin-protein ligase RNF38/44 [EC:2.3.2.27] | (RefSeq) RING-H2 finger protein ATL54-like (A)	ATL54_ARATH RING-H2 finger protein ATL54; EC=2.3.2.27 {ECO:0000305}; RING-type E3 ubiquitin transferase ATL54 {ECO:0000305} (At1g72220)	1.752	3.520

novel.725	K19037 E3 ubiquitin-protein ligase ATL23 [EC:2.3.2.27] | (RefSeq) E3 ubiquitin-protein ligase ATL23 (A)	ATL23_ARATH E3 ubiquitin-protein ligase ATL23; EC=2.3.2.27 {ECO:0000269|PubMed:15644464, ECO:0000269|PubMed:16339806}; RING-H2 finger protein ATL23; RING-type E3 ubiquitin transferase ATL23 {ECO:0000305} (At5g42200)	-1.079	
novel.894	K10664 E3 ubiquitin-protein ligase ATL6/9/15/31/42/55 [EC:2.3.2.27] | (RefSeq) putative RING-H2 finger protein ATL12 (A)	ATL42_ARATH E3 ubiquitin-protein ligase ATL42; EC=2.3.2.27 {ECO:0000269|PubMed:15644464}; RING-H2 finger protein ATL42; RING-type E3 ubiquitin transferase ATL42 {ECO:0000305}; Flags: Precursor (At4g28890)		1.915
novel.911	K19041 E3 ubiquitin-protein ligase RNF38/44 [EC:2.3.2.27] | (RefSeq) RING-H2 finger protein ATL20-like (A)	ATL69_ARATH Putative RING-H2 finger protein ATL69; EC=2.3.2.27 {ECO:0000305}; RING-type E3 ubiquitin transferase ATL69 {ECO:0000305} (At5g07040)	1.230	1.277
orange1.1t00397	K16281 RING-H2 zinc finger protein RHA1 | (RefSeq) hypothetical protein (A)	E3 ubiquitin-protein ligase RHA1B {ECO:0000305}; EC=2.3.2.27; RING-H2 finger A1b {ECO:0000303|PubMed:9781696}; RING-H2 zinc finger protein RHA1b {ECO:0000305}; RING-type E3 ubiquitin transferase RHA1B {ECO:0000305} (At3g61460)		-1.272
orange1.1t01964	K19041 E3 ubiquitin-protein ligase RNF38/44 [EC:2.3.2.27] | (RefSeq) E3 ubiquitin-protein ligase RING1 (A)	RING-H2 finger protein ATL54; EC=2.3.2.27 {ECO:0000305}; RING-type E3 ubiquitin transferase ATL54 {ECO:0000305} (At1g72220)		-1.235
orange1.1t02067	K03094 S-phase kinase-associated protein 1 | (RefSeq) hypothetical protein (A)	SKP1-like protein 11; AtSK11 (At4g34210)	1.002	3.273
orange1.1t03837	K10664 E3 ubiquitin-protein ligase ATL6/9/15/31/42/55 [EC:2.3.2.27] | (RefSeq) E3 ubiquitin-protein ligase ATL42-like (A)	E3 ubiquitin-protein ligase ATL42; EC=2.3.2.27 {ECO:0000269|PubMed:15644464}; RING-H2 finger protein ATL42; RING-type E3 ubiquitin transferase ATL42 {ECO:0000305}; Flags: Precursor (At4g28890)		1.114
orange1.1t04179	K10523 speckle-type POZ protein | (RefSeq) MATH domain and coiled-coil domain-containing protein At3g58270-like (A)	Ubiquitin carboxyl-terminal hydrolase 13; EC=3.4.19.12; Deubiquitinating enzyme 13; AtUBP13; Ubiquitin thioesterase 13; Ubiquitin-specific-processing protease 13 (At3g17380)	1.427	1.344
orange1.1t05146	K10523 speckle-type POZ protein | (RefSeq) MATH domain and coiled-coil domain-containing protein At3g58270-like (A)	Ubiquitin carboxyl-terminal hydrolase 13; EC=3.4.19.12; Deubiquitinating enzyme 13; AtUBP13; Ubiquitin thioesterase 13; Ubiquitin-specific-processing protease 13 (At3g17380)	2.658	
Ubiquitin mediated proteolysis [ko04120, P = 0.9717 (P3CR vs. P3R) and 0.9993 (P5CR vs. P5R)]				
Cs3g04490	K03094 S-phase kinase-associated protein 1 | (RefSeq) uncharacterized LOC107176092 (A)			1.586

orange1.1t02067	K03094 S-phase kinase-associated protein 1 | (RefSeq) hypothetical protein (A)	SKP1-like protein 11; AtSK11 (At4g34210)	1.002	3.273
Cs7g13460	K03868 RING-box protein 1 [EC:2.3.2.32] | (RefSeq) uncharacterized LOC100792815 (A)	GEM-like protein 4 (At5g08350)	-1.645	-4.260
Cs6g15970	K10615 E3 ubiquitin-protein ligase HERC4 [EC:2.3.2.26] | (RefSeq) probable E3 ubiquitin-protein ligase HERC4 isoform X1 (A)	PH, RCC1 and FYVE domains-containing protein 1 {ECO:0000303|PubMed:11563980} Protein Praf4 {ECO:0000303|PubMed:15358268} (At1g65920)		-1.145
Cs5g23960	K03094 S-phase kinase-associated protein 1 | (Kazusa) Lj2g3v3084320.1; - (A)	Protein FEZ; NAC domain-containing protein 9; ANAC009 (At1g26870)	1.289	1.083
Cs5g08400	K03347 cullin 1 | (RefSeq) cullin-1 (A)	RING-H2 finger protein ATL22; EC=2.3.2.27 {ECO:0000305}; RING-type E3 ubiquitin transferase ATL22 {ECO:0000305}; Flags: Precursor (At5g53110)	2.485	
orange1.1t04938	K03350 anaphase-promoting complex subunit 3 | (RefSeq) hypothetical protein (A)	(At1g52140)	1.604	
Cs5g04910	K03362 F-box and WD-40 domain protein 1/11 | (RefSeq) protein JINGUBANG-like (A)	Protein JINGUBANG {ECO:0000303|PubMed:27468890} (At5g50120)	-1.859	-2.345
Cs8g15610	K10144 RING finger and CHY zinc finger domain-containing protein 1 [EC:2.3.2.27] | (RefSeq) E3 ubiquitin-protein ligase MIEL1 (A)	E3 ubiquitin-protein ligase RZFP34 {ECO:0000305}; EC=2.3.2.27 {ECO:0000269|PubMed:26508764}; CHY zinc-finger and RING protein 1 {ECO:0000303|PubMed:26508764}; RING zinc-finger protein 34 {ECO:0000305}; RZFP34 protein homolog {ECO:0000303|PubMed:25002225}; AtRZPF34 {ECO:0000303|PubMed:25002225} (At5g22920)	-1.632	
Cs7g04220	K10573 ubiquitin-conjugating enzyme E2 A [EC:2.3.2.23] | (RefSeq) hypothetical protein (A)	Ubiquitin-conjugating enzyme E2 2; EC=2.3.2.23; E2 ubiquitin-conjugating enzyme 2; Ubiquitin carrier protein 2; Ubiquitin-conjugating enzyme E2-17 kDa 2; Ubiquitin-protein ligase 2 (At2g02760)	-3.437	-2.891
Cs2g06030	K10581 ubiquitin-conjugating enzyme E2 O [EC:2.3.2.24] | (RefSeq) probable ubiquitin-conjugating enzyme E2 24 (A)	Probable ubiquitin-conjugating enzyme E2 24 {ECO:0000303|PubMed:16339806}; EC=2.3.2.23 {ECO:0000305}; AtPHO2 {ECO:0000303|PubMed:16679424}; E2 ubiquitin-conjugating enzyme 24 {ECO:0000303|PubMed:16339806}; Ubiquitin carrier protein 24 {ECO:0000303|PubMed:16339806}; Ubiquitin-protein ligase 24 {ECO:0000303|PubMed:16339806} (At2g16920)	1.191	
novel.60	K10581 ubiquitin-conjugating enzyme E2 O [EC:2.3.2.24] | (RefSeq) uncharacterized LOC105047412 (A)	(At4g21700)	2.570	1.205

Cs4g19400	K10614 E3 ubiquitin-protein ligase HERC3 [EC:2.3.2.26] | (RefSeq) ultraviolet-B receptor UVR8-like (A)	Ultraviolet-B receptor UVR8; Protein UV-B RESISTANCE 8; RCC1 domain-containing protein UVR8 (At5g11580)	1.514	
novel.2184	K03094 S-phase kinase-associated protein 1 | (RefSeq) SKP1-like protein 14 (A)	Y3720_ARATH UPF0481 protein At3g47200 (At4g31980_2)		1.665
Cs5g18350	K03354 anaphase-promoting complex subunit 7 | (RefSeq) uncharacterized LOC102623835 (A)	(At4g10000)		1.215
Cs9g16630	K03363 cell division cycle 20, cofactor of APC complex | (RefSeq) transducin family protein/WD-40 repeat protein (A)	(At5g62150)		1.095
Cs7g13465	K03868 RING-box protein 1 [EC:2.3.2.32] | (RefSeq) uncharacterized LOC100792815 (A)	GEM-like protein 4 (At5g08350)		-2.013
Cs9g17160	K10143 E3 ubiquitin-protein ligase RFWD2 [EC:2.3.2.27] | (RefSeq) WD repeat-containing protein RUP2 (A)	WD repeat-containing protein RUP2; Protein EARLY FLOWERING BY OVEREXPRESSION 2; Protein EPRESSOR OF UV-B PHOTOMORPHOGENESIS 2 (At5g23730)		-2.621
Cs4g17150	K10581 ubiquitin-conjugating enzyme E2 O [EC:2.3.2.24] | (RefSeq) uncharacterized LOC105047412 (A)	(At1g52780)		2.069
Cs1g06920	K10614 E3 ubiquitin-protein ligase HERC3 [EC:2.3.2.26] | (RefSeq) ultraviolet-B receptor UVR8-like (A)	Ultraviolet-B receptor UVR8; Protein UV-B RESISTANCE 8; RCC1 domain-containing protein UVR8 (At3g15430)		1.090
Protein folding [GO:0006457; P = 0.9979 (P3CR vs. P3R) and 1.0000 (P5CR vs. P5R)]				
Cs1g18140	K04077 chaperonin GroEL | (RefSeq) chaperonin CPN60-like 2, mitochondrial (A)	Chaperonin CPN60-like 2, mitochondrial; HSP60-like 2; Flags: Precursor (At3g13860)	-1.065	
Cs2g08140	K10950 ERO1-like protein alpha [EC:1.8.4.-] | (RefSeq) endoplasmic reticulum oxidoreductin-2-like (A)	Endoplasmic reticulum oxidoreductin-1; EC=1.8.4.-; Flags: Precursor (At1g72280)	-1.031	
Cs2g23190	K04077 chaperonin GroEL | (RefSeq) chaperonin CPN60-2, mitochondrial-like (A)	Chaperonin CPN60, mitochondrial; HSP60; Flags: Precursor (At3g23990)		1.064
Cs2g29390	K04078 chaperonin GroES | (RefSeq) hypothetical protein (A)	10 kDa chaperonin, mitochondrial; Chaperonin 10; CPN10; Protein groES; Flags: Precursor (At1g14980)	-1.412	-1.526
Cs4g05880	K13993 HSP20 family protein | (RefSeq) 17.7 kDa class II heat shock protein (A)	17.7 kDa class II heat shock protein; 17.7 kDa heat shock protein; AtHsp17.7 (At5g12030)		2.404
Cs5g03150	K04079 molecular chaperone HtpG | (RefSeq) heat shock protein 83-like (A)	Heat shock protein 83 (At5g52640)	1.176	
Cs6g21710		BAG family molecular chaperone regulator 8, chloroplastic; Bcl-2-associated athanogene 8; Flags: Precursor (At5g62390)		-1.012

Cs9g12770	K12737 peptidyl-prolyl cis-trans isomerase SDCCAG10 [EC:5.2.1.8] | (RefSeq) peptidyl-prolyl cis-trans isomerase CYP57 (A)	Peptidyl-prolyl cis-trans isomerase CYP57; PPIase CYP57; EC=5.2.1.8; Cyclophilin of 57 kDa; Cyclophilin-57 (At4g33060)	2.092	
novel.1859		BAG7_ARATH BAG family molecular chaperone regulator 7; Bcl-2-associated athanogene 7 (At5g62390)		-1.383
novel.2500	K03687 molecular chaperone GrpE | (RefSeq) protein GrpE (A)			1.221
Ribosome biogenesis [GO:0042254; P = 1.0000 (P3CR vs. P3R) and 1.0000 (P5CR vs. P5R)]				
Cs2g08210	K15032 mTERF domain-containing protein, mitochondrial | (RefSeq) uncharacterized LOC102624544 (A)	Transcription termination factor MTEF1, chloroplastic {ECO:0000305}; Mitochondrial transcription termination factor 1 {ECO:0000303|PubMed:23087700}; Protein EMBRYO DEFECTIVE 93 {ECO:0000305}; Protein SINGLET OXYGEN-LINKED DEATH ACTIVATOR 10 {ECO:0000303|PubMed:19563435}; Flags: Precursor (At2g34620)		-1.555
Cs2g23270	K02875 large subunit ribosomal protein L14e | (RefSeq) hypothetical protein (A)	60S ribosomal protein L14-2 (At4g27090)	-2.292	-1.556
Cs3g21570	K02925 large subunit ribosomal protein L3e | (RefSeq) 60S ribosomal protein L3-2-like (A)	60S ribosomal protein L3-2 (At1g61580)	-1.249	-1.351
Cs6g07530	K02917 large subunit ribosomal protein L35Ae | (RefSeq) hypothetical protein (A)	60S ribosomal protein L35a-1 (At1g07070)		-1.006
Cs9g02410	K11797 PH-interacting protein | (RefSeq) PH-interacting protein (A)	Notchless protein homolog {ECO:0000303|PubMed:23572950} (At5g49430)		1.646
Cs9g06870	K02974 small subunit ribosomal protein S24e | (RefSeq) hypothetical protein (A)	40S ribosomal protein S24-2 (At5g28060)	3.649	
novel.2075	K02917 large subunit ribosomal protein L35Ae | (RefSeq) 60S ribosomal protein L35a-1 (A)	R35A3_ARATH 60S ribosomal protein L35a-3 (At1g74270)	-1.576	
Translation initiation factor activity [GO:0003743, P = 0.9828 (P5CR vs. P5R)]				
Cs1g02930	K03260 translation initiation factor 4G | (RefSeq) eukaryotic translation initiation factor 4G (A)	Eukaryotic translation initiation factor 4G; eIF-4G; eIF4G; Protein cucumovirus multiplication 2; Protein synthesis initiation factor 4G (At3g60240_1)		1.133
Cs2g04120	K03259 translation initiation factor 4E | (RefSeq) eukaryotic	Eukaryotic translation initiation factor 4E-1; eIF-4E-1; eIF4E-1; Protein		-1.236

	translation initiation factor 4E-1-like (A)	cucumovirus multiplication 1; eIF-4F 25 kDa subunit; eIF-4F p26 subunit; mRNA cap-binding protein (At4g18040)		
Cs3g15510	K03243 translation initiation factor 5B | (RefSeq) LOW QUALITY PROTEIN: eukaryotic translation initiation factor 5B-like (A)	Translation initiation factor IF-2, chloroplastic (At1g76810)		1.606
Biosynthesis of amino acids [ko01230; P = 0.8553 (P3CR vs. P3R) and 0.9997 (P5CR vs. P5R)]				
Cs1g08010	K00815 tyrosine aminotransferase [EC:2.6.1.5] | (RefSeq) hypothetical protein (A)	Probable aminotransferase TAT2; EC=2.6.1.-; Tyrosine aminotransferase 2 (At5g53970)	1.218	
Cs1g15970	K00850 6-phosphofructokinase 1 [EC:2.7.1.11] | (RefSeq) ATP-dependent 6-phosphofructokinase 3-like (A)	ATP-dependent 6-phosphofructokinase 3 {ECO:0000255|HAMAP-Rule:MF_03186}; ATP-PFK 3 {ECO:0000255|HAMAP-Rule:MF_03186}; Phosphofructokinase 3 {ECO:0000255|HAMAP-Rule:MF_03186}; EC=2.7.1.11 {ECO:0000255|HAMAP-Rule:MF_03186}; Phosphohexokinase 3 {ECO:0000255|HAMAP-Rule:MF_03186} (At4g29220)	1.064	
Cs2g07700	K01696 tryptophan synthase beta chain [EC:4.2.1.20] | (RefSeq) tryptophan synthase beta chain 1 (A)	Tryptophan synthase beta chain 2, chloroplastic; EC=4.2.1.20; Flags: Precursor (At4g27070)	-1.338	-1.506
Cs2g13960	K00891 shikimate kinase [EC:2.7.1.71] | (RefSeq) probable inactive shikimate kinase like 1, chloroplastic (A)	Probable inactive shikimate kinase like 1, chloroplastic; AtSKL1; Flags: Precursor (At3g26900)		-1.076
Cs2g14460	K00928 aspartate kinase [EC:2.7.2.4] | (RefSeq) aspartokinase 2, chloroplastic-like (A)	Aspartokinase 3, chloroplastic; EC=2.7.2.4; Aspartate kinase 3; Flags: Precursor (At3g02020)	-1.045	
Cs2g14890	K01689 enolase [EC:4.2.1.11] | (RefSeq) enolase 1, chloroplastic isoform X1 (A)	(At5g47920)		1.457
Cs2g23840	K00815 tyrosine aminotransferase [EC:2.6.1.5] | (RefSeq) hypothetical protein (A)	Probable aminotransferase TAT2; EC=2.6.1.-; Tyrosine aminotransferase 2 (At5g53970)		1.425
Cs2g23850	K00815 tyrosine aminotransferase [EC:2.6.1.5] | (RefSeq) hypothetical protein (A)	Probable aminotransferase TAT2; EC=2.6.1.-; Tyrosine aminotransferase 2 (At5g53970)		1.042
Cs2g28930	K00549 5-methyltetrahydropteroyltriglutamatehomocysteine methyltransferase [EC:2.1.1.14] | (RefSeq) hypothetical protein (A)	5-methyltetrahydropteroyltriglutamatehomocysteine methyltransferase; EC=2.1.1.14; Cobalamin-independent methionine synthase isozyme; Vitamin-B12-independent methionine synthase isozyme (At5g17920)		1.292
Cs3g03620	K01695 tryptophan synthase alpha chain [EC:4.2.1.20] | (RefSeq) tryptophan synthase alpha chain-like (A)	Tryptophan synthase alpha chain; EC=4.2.1.20; Indole synthase; Indole-3-glycerol-phosphate lyase; EC=4.1.2.8 (At4g02610)	5.206	
Cs3g11490	K01738 cysteine synthase A [EC:2.5.1.47] | (RefSeq)	Cysteine synthase, chloroplastic/chromoplastic; EC=2.5.1.47; CSase B;	1.049	1.627

	hypothetical protein (A)	CS-B; O-acetylserine (thiol)-lyase; O-acetylserine sulfhydrylase; OAS-TL B; Flags: Precursor (At2g43750)		
Cs3g11520	K01738 cysteine synthase A [EC:2.5.1.47] | (RefSeq) cysteine synthase-like (A)	Cysteine synthase {ECO:0000303|PubMed:8280125}; CSase; EC=2.5.1.47 {ECO:0000269|PubMed:8280125, ECO:0000269|PubMed:9013806}; Beta-PA/CSase; EC=2.5.1.51 {ECO:0000269|PubMed:8280125, ECO:0000269|PubMed:9013806}; Beta-pyrazolylalanine synthase {ECO:0000303|PubMed:8280125}; EC=4.2.1.50 {ECO:0000269|PubMed:8280125, ECO:0000269|PubMed:9013806}; L-mimosine synthase; EC=2.5.1.52 {ECO:0000269|PubMed:9013806}; O-acetylserine (thiol)-lyase; OAS-TL; O-acetylserine sulfhydrylase (At5g28020)		1.745
Cs3g16440	K00826 branched-chain amino acid aminotransferase [EC:2.6.1.42] | (RefSeq) branched-chain-amino-acid aminotransferase 2, chloroplastic (A)	Branched-chain-amino-acid aminotransferase 2, chloroplastic; Atbcat-2; EC=2.6.1.42; Flags: Precursor (At1g10070)		1.092
Cs3g17650	K00850 6-phosphofructokinase 1 [EC:2.7.1.11] | (RefSeq) ATP-dependent 6-phosphofructokinase 6-like (A)	Purine permease 1 {ECO:0000303|PubMed:10662864}; AtPUP1 {ECO:0000303|PubMed:10662864} (At1g28230)		-1.500
Cs4g05200	K15227 arogenate dehydrogenase (NADP+), plant [EC:1.3.1.78] | (RefSeq) hypothetical protein (A)	Arogenate dehydrogenase 1, chloroplastic; EC=1.3.1.78; TYRATC; TyrAAT1; Flags: Precursor (At5g34930)		1.176
Cs4g13070	K00850 6-phosphofructokinase 1 [EC:2.7.1.11] | (RefSeq) ATP-dependent 6-phosphofructokinase 3-like (A)	ATP-dependent 6-phosphofructokinase 3 {ECO:0000255|HAMAP-Rule:MF_03186}; ATP-PFK 3 {ECO:0000255|HAMAP-Rule:MF_03186}; Phosphofructokinase 3 {ECO:0000255|HAMAP-Rule:MF_03186}; EC=2.7.1.11 {ECO:0000255|HAMAP-Rule:MF_03186}; Phosphohexokinase 3 {ECO:0000255|HAMAP-Rule:MF_03186} (At5g56630)	-1.258	
Cs4g18980	K00850 6-phosphofructokinase 1 [EC:2.7.1.11] | (RefSeq) hypothetical protein (A)	ATP-dependent 6-phosphofructokinase 3 {ECO:0000255|HAMAP-Rule:MF_03186}; ATP-PFK 3 {ECO:0000255|HAMAP-Rule:MF_03186}; Phosphofructokinase 3 {ECO:0000255|HAMAP-Rule:MF_03186}; EC=2.7.1.11 {ECO:0000255|HAMAP-Rule:MF_03186}; Phosphohexokinase 3 {ECO:0000255|HAMAP-Rule:MF_03186} (At4g32840)	-1.602	-1.466
Cs5g04240	K00549 5-methyltetrahydropteroyltriglutamatehomocysteine methyltransferase [EC:2.1.1.14] | (RefSeq) protein QUIRKY-like (A)	Protein QUIRKY {ECO:0000303|PubMed:19180193} (At1g74720)		1.383
Cs5g05650	K00766 anthranilate phosphoribosyltransferase [EC:2.4.2.18] |	Anthranilate phosphoribosyltransferase, chloroplastic; EC=2.4.2.18;	-1.039	

	(RefSeq) anthranilate phosphoribosyltransferase, chloroplastic (A)	Flags: Precursor (At5g17990)		
Cs5g27350	K00549 5-methyltetrahydropteroyltriglutamatehomocysteine methyltransferase [EC:2.1.1.14] | (RefSeq) protein QUIRKY-like (A)	Protein QUIRKY {ECO:0000303|PubMed:19180193} (At3g03680)	-1.466	-1.678
Cs6g09690	K14455 aspartate aminotransferase, mitochondrial [EC:2.6.1.1] | (RefSeq) hypothetical protein (A)	Aspartate aminotransferase, mitochondrial; EC=2.6.1.1; Transaminase A; Flags: Precursor (At2g30970)	-1.627	
Cs7g24920	K00615 transketolase [EC:2.2.1.1] | (RefSeq) transketolase, chloroplastic-like (A)	Transketolase-1, chloroplastic; TK; EC=2.2.1.1; Flags: Precursor (At2g45290)	1.474	-1.189
Cs8g01250	K14677 aminoacylase [EC:3.5.1.14] | (RefSeq) hypothetical protein (A)	(At4g38220)	1.180	
Cs8g03410	K00826 branched-chain amino acid aminotransferase [EC:2.6.1.42] | (RefSeq) hypothetical protein (A)	Branched-chain-amino-acid aminotransferase 2, chloroplastic; Atbcat-2; EC=2.6.1.42; Flags: Precursor (At1g10070)	1.282	
Cs8g08710	K01623 fructose-bisphosphate aldolase, class I [EC:4.1.2.13] | (RefSeq) fructose-bisphosphate aldolase 1, chloroplastic (A)	Fructose-bisphosphate aldolase 2, chloroplastic {ECO:0000305}; AtFBA2 {ECO:0000303|PubMed:22561114}; EC=4.1.2.13 {ECO:0000305}; Flags: Precursor (At2g01140)	1.927	
Cs8g12640	K01620 threonine aldolase [EC:4.1.2.48] | (RefSeq) probable low-specificity L-threonine aldolase 1 (A)	Probable low-specificity L-threonine aldolase 1; EC=4.1.2.48; Threonine aldolase 1 (At1g08630)	1.132	
Cs9g10010	K05359 arogenate/prephenate dehydratase [EC:4.2.1.91 4.2.1.51] | (RefSeq) arogenate dehydratase/prephenate dehydratase 1, chloroplastic-like (A)	Arogenate dehydratase/prephenate dehydratase 1, chloroplastic; AtADT1; AtPDT1; EC=4.2.1.51; EC=4.2.1.91; Flags: Precursor (At1g11790)	1.603	2.050
Cs9g17270	K01623 fructose-bisphosphate aldolase, class I [EC:4.1.2.13] | (RefSeq) fructose-bisphosphate aldolase 6, cytosolic-like (A)	Heavy metal-associated isoprenylated plant protein 39 {ECO:0000303|PubMed:21072340, ECO:0000303|PubMed:23368984}; AtHIP39 {ECO:0000303|PubMed:21072340, ECO:0000303|PubMed:23368984}; Flags: Precursor (At1g01490)		-1.134
Cs9g18120	K00873 pyruvate kinase [EC:2.7.1.40] | (RefSeq) hypothetical protein (A)	Plastidial pyruvate kinase 4, chloroplastic; PKp4; EC=2.7.1.40; Flags: Precursor (At3g49160)	-1.526	-1.586
novel.25	K00815 tyrosine aminotransferase [EC:2.6.1.5] | (RefSeq) LOW QUALITY PROTEIN: tyrosine aminotransferase-like (A)	(At2g06190)	-1.347	
novel.424	K01778 diaminopimelate epimerase [EC:5.1.1.7] | (RefSeq) hypothetical protein (A)	DAPF_ARATH Diaminopimelate epimerase, chloroplastic; DAP epimerase; EC=5.1.1.7; Flags: Precursor (At3g53580)		-1.524
orange1.1t00222	K01476 arginase [EC:3.5.3.1] | (RefSeq) hypothetical protein (A)	Arginase 1, mitochondrial; EC=3.5.3.1 {ECO:0000250|UniProtKB:P05089}; Agmatinase ARGAH1		1.387

		{ECO:0000305}; EC=3.5.3.11 {ECO:0000305|PubMed:28716421}; Arginine amidohydrolase 1; Flags: Precursor (At4g08900)		
orange1.1t00345	K00549 5-methyltetrahydropteroyltriglutamatehomocysteine methyltransferase [EC:2.1.1.14] | (RefSeq) protein QUIRKY-like (A)	FT-interacting protein 1 {ECO:0000303|PubMed:22529749} (At4g11610)	1.167	1.418
orange1.1t04496	K00058 D-3-phosphoglycerate dehydrogenase / 2-oxoglutarate reductase [EC:1.1.1.95 1.1.1.399] | (RefSeq) D-3-phosphoglycerate dehydrogenase 2, chloroplastic-like (A)	D-3-phosphoglycerate dehydrogenase 2, chloroplastic; PGDH; EC=1.1.1.95 {ECO:0000269|PubMed:24368794, ECO:0000269|PubMed:9867856}; Flags: Precursor (At1g17740)		1.349
Cellular amino acid catabolic process [GO:0009063; P = 0.3565 (P3CR vs. P3R) and 0.4110 (P5CR vs. P5R)]				
Cs1g08010	K00815 tyrosine aminotransferase [EC:2.6.1.5] | (RefSeq) hypothetical protein (A)	Probable aminotransferase TAT2; EC=2.6.1.-; Tyrosine aminotransferase 2 (At5g53970)	1.218	
Cs1g24170	K22369 epoxide hydrolase 4 [EC:3.3.-.-] | (RefSeq) uncharacterized protein LOC104235966 (A)	Protein PHYLLO, chloroplastic; Includes: Inactive isochorismate synthase; MENF; Includes: 2-succinyl-5-enolpyruvyl-6-hydroxy-3-cyclohexene-1-carboxylate synthase; EC=2.2.1.9; MEND; Includes: o-succinylbenzoate synthase; EC=4.2.1.113; MENC; Includes: 2-succinyl-6-hydroxy-2,4-cyclohexadiene-1-carboxylate synthase; EC=4.2.99.20; MENH; Flags: Precursor (At4g36610)	2.034	4.801
Cs2g23840	K00815 tyrosine aminotransferase [EC:2.6.1.5] | (RefSeq) hypothetical protein (A)	Probable aminotransferase TAT2; EC=2.6.1.-; Tyrosine aminotransferase 2 (At5g53970)		1.425
Cs2g23850	K00815 tyrosine aminotransferase [EC:2.6.1.5] | (RefSeq) hypothetical protein (A)	Probable aminotransferase TAT2; EC=2.6.1.-; Tyrosine aminotransferase 2 (At5g53970)		1.042
Cs4g06620	K14157 alpha-aminoadipic semialdehyde synthase [EC:1.5.1.8 1.5.1.9] | (RefSeq) alpha-aminoadipic semialdehyde synthase (A)	Alpha-aminoadipic semialdehyde synthase; cAt-LKR/SDH; LKR/SDH; Includes: Lysine ketoglutarate reductase; LKR; EC=1.5.1.8; Includes: Saccharopine dehydrogenase; EC=1.5.1.9; cAt-SDH; SDH (At4g33150)	1.865	
Cs5g01510	K01761 methionine-gamma-lyase [EC:4.4.1.11] | (RefSeq) methionine gamma-lyase (A)	Methionine gamma-lyase; AtMGL; EC=4.4.1.11; L-methioninase (At1g64660)		-1.687
Cs6g11950	K10775 phenylalanine ammonia-lyase [EC:4.3.1.24] | (RefSeq) phenylalanine ammonia-lyase (A)	Phenylalanine ammonia-lyase; EC=4.3.1.24 (At2g37040)		-1.149
Cs7g11970	K09716 D-aminoacyl-tRNA deacylase [EC:3.1.1.96] | (RefSeq) D-aminoacyl-tRNA deacylase (A)	D-aminoacyl-tRNA deacylase {ECO:0000303|PubMed:17251192}; EC=3.1.1.96 {ECO:0000269|PubMed:17251192}; Ethanol tolerance protein GEKO1 (At2g03800)	-1.056	
Cs7g22860	K02437 glycine cleavage system H protein | (RefSeq)	Glycine cleavage system H protein, mitochondrial; Flags: Precursor		-1.360

	hypothetical protein (A)	(At1g32470)		
Cs7g25030	K10775 phenylalanine ammonia-lyase [EC:4.3.1.24] | (RefSeq) phenylalanine ammonia-lyase (A)	Phenylalanine ammonia-lyase; EC=4.3.1.24; Flags: Fragment (At3g53260)		-2.319
Cs8g12640	K01620 threonine aldolase [EC:4.1.2.48] | (RefSeq) probable low-specificity L-threonine aldolase 1 (A)	Probable low-specificity L-threonine aldolase 1; EC=4.1.2.48; Threonine aldolase 1 (At1g08630)	1.132	
Cs8g16290	K10775 phenylalanine ammonia-lyase [EC:4.3.1.24] | (RefSeq) phenylalanine ammonia-lyase-like (A)	Phenylalanine ammonia-lyase 1; EC=4.3.1.24 (At2g37040)		2.899
orange1.1t00222	K01476 arginase [EC:3.5.3.1] | (RefSeq) hypothetical protein (A)	Arginase 1, mitochondrial; EC=3.5.3.1 {ECO:0000250|UniProtKB:P05089}; Agmatinase ARGAH1 {ECO:0000305}; EC=3.5.3.11 {ECO:0000305|PubMed:28716421}; Arginine amidohydrolase 1; Flags: Precursor (At4g08900)		1.387
orange1.1t04550	K00451 homogentisate 1,2-dioxygenase [EC:1.13.11.5] | (RefSeq) homogentisate 1,2-dioxygenase (A)	Homogentisate 1,2-dioxygenase; EC=1.13.11.5; Homogentisate oxygenase; Homogentisic acid oxidase; Homogentisicase (At5g54080)	1.095	
Tryptophan metabolism [ko00380; P = 0.0181 (P3CR vs. P3R) and 0.4809 (P5CR vs. P5R)]				
Cs1g01110	K01593 aromatic-L-amino-acid/L-tryptophan decarboxylase [EC:4.1.1.28 4.1.1.105] | (RefSeq) tyrosine decarboxylase 1-like (A)	Tyrosine/DOPA decarboxylase 3; Includes: DOPA decarboxylase; DDC; EC=4.1.1.28; Includes: Tyrosine decarboxylase; EC=4.1.1.25 (At2g20340)		-1.195
Cs1g24850	K00128 aldehyde dehydrogenase (NAD+) [EC:1.2.1.3] | (RefSeq) aldehyde dehydrogenase family 3 member F1 (A)	Aldehyde dehydrogenase family 3 member F1; EC=1.2.1.3 (At4g36250)	1.544	
Cs2g22660	K01593 aromatic-L-amino-acid/L-tryptophan decarboxylase [EC:4.1.1.28 4.1.1.105] | (RefSeq) hypothetical protein (A)	Aromatic-L-amino-acid decarboxylase; AADC; EC=4.1.1.28; DOPA decarboxylase; Tryptophan decarboxylase (At2g20340)	1.556	-1.894
Cs2g30610	K01593 aromatic-L-amino-acid/L-tryptophan decarboxylase [EC:4.1.1.28 4.1.1.105] | (RefSeq) tyrosine/DOPA decarboxylase 1-like (A)	Tyrosine/DOPA decarboxylase 2; Includes: DOPA decarboxylase; DDC; EC=4.1.1.28; Includes: Tyrosine decarboxylase; EC=4.1.1.25 (At2g20340)		-1.272
Cs3g13580	K01426 amidase [EC:3.5.1.4] | (RefSeq) hypothetical protein (A)	Amidase 1 {ECO:0000303|PubMed:16738862}; AtAMI1 {ECO:0000303|PubMed:12620340}; EC=3.5.1.4 {ECO:0000269|PubMed:12620340, ECO:0000269|PubMed:17555521, ECO:0000269|PubMed:27135507}; Translocon at the outer membrane of chloroplasts 64-I {ECO:0000305}; AtTOC64-I {ECO:0000303|PubMed:14741350} (At5g09420_1)		1.019
Cs3g14390	K00128 aldehyde dehydrogenase (NAD+) [EC:1.2.1.3] | (RefSeq) aldehyde dehydrogenase 5, mitochondrial-like (A)	Translationally-controlled tumor protein homolog; TCTP (At3g16640)		-2.145

Cs5g18710	K07408 cytochrome P450 family 1 subfamily A polypeptide 1 [EC:1.14.14.1] | (RefSeq) licodione synthase-like (A)	Licodione synthase; EC=1.14.13.87; (2S)-flavanone 2-hydroxylase; CYP GE-5; Cytochrome P450 93B1; Flavone synthase II (At5g06900)	1.108	
Cs5g28080	K01426 amidase [EC:3.5.1.4] | (RefSeq) LOW QUALITY PROTEIN: uncharacterized protein LOC105643773 (A)	Chemocyanin; Basic blue protein; Plantacyanin; Flags: Precursor (At2g02850)	-1.228	
Cs7g08110	K11820 N-hydroxythioamide S-beta-glucosyltransferase [EC:2.4.1.195] | (RefSeq) UDP-glycosyltransferase 74B1 (A)	UDP-glycosyltransferase 74B1; EC=2.4.1.-; N-hydroxythioamide S-beta-glucosyltransferase; EC=2.4.1.195; Thiohydroximate S-glucosyltransferase (At1g24100)	-1.156	-2.258
Cs7g09110	K16903 L-tryptophan-pyruvate aminotransferase [EC:2.6.1.99] | (RefSeq) hypothetical protein (A)	L-tryptophanpyruvate aminotransferase 1; EC=2.6.1.27; EC=2.6.1.99; Protein CYTOKININ INDUCED ROOT CURLING 1; Protein SHADE AVOIDANCE 3; Protein TRANSPORT INHIBITOR RESPONSE 2; Protein TRYPTOPHAN AMINOTRANSFERASE OF ARABIDOPSIS 1; Protein WEAK ETHYLENE INSENSITIVE 8; Tryptophan transaminase (At1g70560)		-2.122
Cs7g15500	K07409 cytochrome P450 family 1 subfamily A polypeptide 2 [EC:1.14.14.1] | (RefSeq) cytochrome P450 89A2-like (A)	Cytochrome P450 89A2; EC=1.14.-.-; ATH 6-1; CYPLXXXIX (At1g64940)	1.947	
Cs7g15540	K07409 cytochrome P450 family 1 subfamily A polypeptide 2 [EC:1.14.14.1] | (RefSeq) cytochrome P450 89A2-like (A)	Cytochrome P450 89A2; EC=1.14.-.-; ATH 6-1; CYPLXXXIX (At1g64940)		1.202
Cs7g20830	K01593 aromatic-L-amino-acid/L-tryptophan decarboxylase [EC:4.1.1.28 4.1.1.105] | (RefSeq) tyrosine/DOPA decarboxylase (A)	(At5g01130)	1.043	
Cs7g28480	K01593 aromatic-L-amino-acid/L-tryptophan decarboxylase [EC:4.1.1.28 4.1.1.105] | (RefSeq) tyrosine/DOPA decarboxylase (A)	(At5g01150)	1.596	
Cs7g28520	K01593 aromatic-L-amino-acid/L-tryptophan decarboxylase [EC:4.1.1.28 4.1.1.105] | (RefSeq) tyrosine/DOPA decarboxylase (A)	(At5g01150)	1.708	
Cs8g09330	K01426 amidase [EC:3.5.1.4] | (RefSeq) probable amidase At4g34880 (A)	Probable amidase At4g34880 {ECO:0000305}; EC=3.5.1.4 {ECO:0000250|UniProtKB:Q9FR37} (At4g34880)	1.502	2.204
Cs8g09340	K01426 amidase [EC:3.5.1.4] | (RefSeq) probable amidase At4g34880 isoform X1 (A)	Probable amidase At4g34880 {ECO:0000305}; EC=3.5.1.4 {ECO:0000250|UniProtKB:Q9FR37} (At4g34880)	-1.085	
Cs8g09350	K01426 amidase [EC:3.5.1.4] | (RefSeq) probable amidase At4g34880 (A)	Probable amidase At4g34880 {ECO:0000305}; EC=3.5.1.4 {ECO:0000250|UniProtKB:Q9FR37} (At4g34880)	-1.105	-2.507
novel.677	K11816 indole-3-pyruvate monooxygenase [EC:1.14.13.168] | (RefSeq) probable indole-3-pyruvate monooxygenase	CPPM_ARATH Carboxyvinyl-carboxyphosphonate phosphorylmutase, chloroplastic; EC=2.7.8.23; Carboxyphosphonoenolpyruvate	1.467	1.415

	YUCCA11 (A)	phosphonomutase; CPEP phosphonomutase; Flags: Precursor (At1g77060)		
orange1.1t00025	K07408 cytochrome P450 family 1 subfamily A polypeptide 1 [EC:1.14.14.1] | (RefSeq) cytochrome P450 71A26-like (A)	Cytochrome P450 71A22; EC=1.14.-.- (At4g13310)		-1.333
orange1.1t04380	K03781 catalase [EC:1.11.1.6] | (RefSeq) uncharacterized protein y4iK-like (A)	4-coumarateCoA ligase-like 2; EC=6.2.1.-;(Hs22051274)	2.250	-7.568
**Index**	**Compounds**		**Log_2(FC)**	
			P3CR vs. P3R	P5CR vs. P5R
Nitrogen metabolism [ko00910; P = 0.5791 (P3CR vs. P3R) and 0.2606 (P5CR vs. P5R)]				
pme0014	L-Glutamic acid			1.320
pme0193	L-Glutamine		-1.155	
Biosynthesis of amino acids [ko01230 P = 0.9990 (P3CR vs. P3R) and 0.0517 (P5CR vs. P5R)]				
mws0230	L-Threonine			1.615
mws0254	L-Histidine			-1.003
MWS0631	S-Sulfo-L-cysteine			-2.103
mws0671	L-Homoserine		2.706	2.457
MWStz103	S-(5'-Adenosyl)-L-methionine		-2.702	
pma6455	Ribulose-5-phosphate			1.628
pme0014	L-Glutamic acid			1.320
pme0026	L-Lysine		-1.155	
pme0193	L-Glutamine		-1.155	
pme0195	L-Cysteine			-1.032
pme1210	L-Methionine		-1.208	
Zmyn000453	Isocitric Acid		-2.158	
Zmzn000078	Dihydroxyacetone phosphate			1.353
Zmzn000079	D-Erythrose-4-phosphate			1.247
Tryptophan metabolism [ko00380; P = 0.0635 (P3CR vs. P3R)]				
ML10198935	3-(2-(Methylamino)ethyl)-1H-indol-5-ol		5.361	
NK10253223	2-Amino-3-methoxybenzoic Acid		-2.986	

pmb0818	Methoxyindoleacetic acid		1.693	
pmb1096	Indole		1.124	
pme1216	2-Picolinic acid		1.046	
