# Supplementary material for: Mechanisms by Which Increased pH Ameliorates Copper Excess in Citrus sinensis Roots: Insight from a Combined Analysis of Physiology, Transcriptome, and Metabolome
Source: Plants (Basel). 2024 Oct 31;13(21):3054. doi: 10.3390/plants13213054 (PMC11548300; doi:10.3390/plants13213054)
Supplement: Supplementary file 1 [file plants-13-03054-s001.zip › Table S16SecondRevision.pdf]

Table S16. Classification of DAMs based on whether they contain N, P, or S

Metabolites	P3CR vs. P3R		P5CR vs. P5R	
	Up-regulation	Down-regulation	Up-regulation	Downregulation
PMs				
Containing N	16	61	18	26
Containing P	10	27	15	5
Containing S	0	9	1	4
Total PMs	46	80	31	45
SMs				
Containing N	25	15	6	9
Containing P	1	0	0	0
Containing S	0	0	0	0
Total SMs	133	75	44	34
