# Supplementary material for: Mechanisms by Which Increased pH Ameliorates Copper Excess in Citrus sinensis Roots: Insight from a Combined Analysis of Physiology, Transcriptome, and Metabolome
Source: Plants (Basel). 2024 Oct 31;13(21):3054. doi: 10.3390/plants13213054 (PMC11548300; doi:10.3390/plants13213054)
Supplement: Supplementary file 1 [file plants-13-03054-s001.zip › Table S17SecondRevision.pdf]

Table S17. DEGs related to lipid metabolism in P3CR vs. P3R and/or P5CR vs. P5R

**Accession No.**	**KEGG**	**Swissprot (KOG)**	**Log2(FC)**	
			P3CR vs. P3R	P5CR vs. P5R
Fatty acid biosynthetic process [GO:0006633; P = (P3CR vs. P3R) and 0.0943 (P5CR vs. P5R)]				
Cs1g07440		Protein GRIM REAPER {ECO:0000303|PubMed:19279211}; Stigma-specific STIG1-like protein GRI {ECO:0000303|PubMed:19279211}; Contains: GRIp {ECO:0000303|PubMed:25398910}; Flags: Precursor (At1g53130)		1.169
Cs1g17380	K00454 lipoxygenase [EC:1.13.11.12] | (RefSeq) linoleate 13S-lipoxygenase 3-1, chloroplastic-like (A)	Linoleate 13S-lipoxygenase 3-1, chloroplastic; EC=1.13.11.12; Flags: Precursor (At1g17420)	1.679	2.275
Cs1g20370	K10703 very-long-chain (3R)-3-hydroxyacyl-CoA dehydratase [EC:4.2.1.134] | (RefSeq) very-long-chain (3R)-3-hydroxyacyl-CoA dehydratase PASTICCINO 2 (A)	Very-long-chain (3R)-3-hydroxyacyl-CoA dehydratase PASTICCINO 2 {ECO:0000305}; EC=4.2.1.134 {ECO:0000305|PubMed:18799749}; 3-hydroxyacyl-CoA dehydratase PASTICCINO 2; AtPAS2; HACD; HCD; Protein PEPINO; PEP; Protein tyrosine phosphatase-like protein (At5g10480)		1.075
Cs2g13450	K10781 fatty acyl-ACP thioesterase B [EC:3.1.2.14 3.1.2.21] | (RefSeq) hypothetical protein (A)	Palmitoyl-acyl carrier protein thioesterase, chloroplastic; EC=3.1.2.-; 16:0-acyl-carrier protein thioesterase; 16:0-ACP thioesterase; Acyl-[acyl-carrier-protein] hydrolase; PATE; Flags: Precursor (At1g08510)		-2.986
Cs2g19370	K07199 5'-AMP-activated protein kinase, regulatory beta subunit | (RefSeq) SNF1-related protein kinase regulatory subunit beta-2-like (A)	SNF1-related protein kinase regulatory subunit beta-2; AKIN subunit beta-2; AKINB2; AKINbeta2 (At4g16360)	1.171	
Cs2g21000	K10528 hydroperoxide lyase [EC:4.1.2.-] | (RefSeq) hypothetical protein (A)	Fatty acid hydroperoxide lyase, chloroplastic {ECO:0000303|PubMed:10859201}; LeHPL {ECO:0000303|PubMed:10859201}; EC=4.2.99.- {ECO:0000305}; Cytochrome P450 74B {ECO:0000305} (At4g15440)		-1.134
Cs2g28670	K10529 alpha-dioxygenase [EC:1.-.-.-] | (RefSeq) alpha-dioxygenase 1-like (A)	Alpha-dioxygenase 1; Alpha DOX1; EC=1.14.99.-; Fatty acid dioxygenase AlphaDOX1; Pathogen-induced oxygenase; Plant alpha dioxygenase 1; Flags: Precursor (At3g01420)	1.027	-2.590
Cs2g28680	K10529 alpha-dioxygenase [EC:1.-.-.-] | (RefSeq) alpha-dioxygenase 1-like (A)	Alpha-dioxygenase 1; Alpha DOX1; EC=1.14.99.-; Fatty acid dioxygenase AlphaDOX1; Pathogen-induced oxygenase; Plant alpha		-3.541

		dioxygenase 1; Flags: Precursor (At3g01420)		
Cs3g04040	K10249 elongation of very long chain fatty acids protein 4 [EC:2.3.1.199] | (RefSeq) elongation of fatty acids protein A-like (A)	Elongation of fatty acids protein 3-like; Protein ELO3-like; EC=2.3.1.-; Protein HIGH EXPRESSION OF OSMOTICALLY RESPONSIVE GENES 3; Very long-chain fatty acid condensing enzyme HOS3; VLCFA condensing enzyme HOS3 (At3g06470)		-2.061
Cs3g06080	K10525 allene oxide cyclase [EC:5.3.99.6] | (RefSeq) allene oxide cyclase 4, chloroplastic-like (A)	Allene oxide cyclase, chloroplastic {ECO:0000303|Ref.1, ECO:0000303|Ref.2}; OsAOC {ECO:0000303|Ref.1}; EC=5.3.99.6 {ECO:0000269|PubMed:23347338}; Protein COLEOPTILE PHOTOMORPHOGENESIS 2 {ECO:0000303|PubMed:23347338}; Protein HEBIBA {ECO:0000303|PubMed:14605232}; Flags: Precursor (At1g13280)	-1.814	-1.501
Cs3g13930	K15718 linoleate 9S-lipoxygenase [EC:1.13.11.58] | (RefSeq) hypothetical protein (A)	Probable linoleate 9S-lipoxygenase 5; EC=1.13.11.58; Leaf lipoxygenase (At1g55020)	1.229	
Cs3g15530	K03921 acyl-[acyl-carrier-protein] desaturase [EC:1.14.19.2 1.14.19.11 1.14.19.26] | (RefSeq) stearoyl-[acyl-carrier-protein] 9-desaturase 5, chloroplastic-like (A)	Stearoyl-[acyl-carrier-protein] 9-desaturase 5, chloroplastic; Stearoyl-ACP desaturase 5; EC=1.14.19.2 {ECO:0000269|PubMed:17072561}; Acyl-[acyl-carrier-protein] desaturase 5; Flags: Precursor (At3g02630)	-1.191	
Cs3g18630	K11262 acetyl-CoA carboxylase / biotin carboxylase 1 [EC:6.4.1.2 6.3.4.14 2.1.3.15] | (RefSeq) acetyl-CoA carboxylase 1-like (A)	Acetyl-CoA carboxylase 1; AtACC1; EC=6.4.1.2 {ECO:0000269|PubMed:9008389}; Protein EMBRYO DEFECTIVE 22; Protein GURKE; Protein PASTICCINO 3; Includes: Biotin carboxylase; EC=6.3.4.14 (At1g36160)		1.119
Cs3g20840	K03921 acyl-[acyl-carrier-protein] desaturase [EC:1.14.19.2 1.14.19.11 1.14.19.26] | (RefSeq) stearoyl-[acyl-carrier-protein] 9-desaturase 6, chloroplastic-like (A)	Stearoyl-[acyl-carrier-protein] 9-desaturase 6, chloroplastic; Stearoyl-ACP desaturase 6; EC=1.14.19.2 {ECO:0000250|UniProtKB:P22337}; Acyl-[acyl-carrier-protein] desaturase 6; Flags: Precursor (At1g43800)	-1.564	
Cs4g02210	K09285 AP2-like factor, ANT lineage | (RefSeq) AP2-like ethylene-responsive transcription factor At1g16060 (A)	AP2-like ethylene-responsive transcription factor At1g16060 (At1g16060)	1.475	
Cs5g28310	K01723 hydroperoxide dehydratase [EC:4.2.1.92] | (RefSeq) hypothetical protein (A)	Allene oxide synthase 3 {ECO:0000303|PubMed:12351632}; LeAOS3 {ECO:0000303|PubMed:12351632}; EC=4.2.1.92 {ECO:0000269|PubMed:12351632}; Allene oxide cyclase {ECO:0000303|PubMed:12351632}; EC=5.3.99.6 {ECO:0000269|PubMed:12351632}; Cytochrome P450 CYP74C3 {ECO:0000303|PubMed:12351632}; Cytochrome P450 CYP74D1 {ECO:0000303|PubMed:17073790} (At5g42650)		2.459

Cs6g16980	K00059 3-oxoacyl-[acyl-carrier protein] reductase [EC:1.1.1.100] | (RefSeq) uncharacterized protein LOC112507113 (A)	3-oxoacyl-[acyl-carrier-protein] reductase, chloroplastic; EC=1.1.1.100; 3-ketoacyl-acyl carrier protein reductase; Flags: Precursor (At3g46170)		-2.084
Cs7g13310	K15397 3-ketoacyl-CoA synthase [EC:2.3.1.199] | (RefSeq) hypothetical protein (A)	3-ketoacyl-CoA synthase 6 {ECO:0000303|PubMed:18465198}; KCS-6 {ECO:0000303|PubMed:18465198}; EC=2.3.1.199 {ECO:0000269|PubMed:10330468}; Cuticular protein 1 {ECO:0000303|PubMed:10330468}; Eceriferum 6 {ECO:0000303|PubMed:11041893}; Very long-chain fatty acid condensing enzyme 6 {ECO:0000303|PubMed:18465198}; VLCFA condensing enzyme 6 {ECO:0000303|PubMed:18465198} (At1g68530)	3.174	6.706
Cs7g13930	K15402 fatty acid omega-hydroxylase [EC:1.14.-.-] | (RefSeq) cytochrome P450 86B1 (A)	Cytochrome P450 86B1; EC=1.14.-.- (At5g23190)		3.197
Cs8g05800	K00069 15-hydroxyprostaglandin dehydrogenase (NAD) [EC:1.1.1.141] | (RefSeq) 15-hydroxyprostaglandin dehydrogenase [NAD(+)]-like (A)	3-oxoacyl-[acyl-carrier-protein] reductase, chloroplastic; EC=1.1.1.100; 3-ketoacyl-acyl carrier protein reductase; Flags: Precursor (At1g49670_1)	-1.452	-1.487
Cs8g17080	K16815 calcium-independent phospholipase A2-gamma | (RefSeq) hypothetical protein (A)	Patatin-like protein 2; AtPLP2; EC=3.1.1.-; Patatin-related phospholipase A IIalpha; pPLAIIa; Phospholipase A IIA; AtPLAIIA (At2g26560)		1.072
Cs8g17090	K16815 calcium-independent phospholipase A2-gamma | (RefSeq) hypothetical protein (A)	Patatin-like protein 2; AtPLP2; EC=3.1.1.-; Patatin-related phospholipase A IIalpha; pPLAIIa; Phospholipase A IIA; AtPLAIIA (At2g26560)		1.375
Cs9g05210	K07513 acetyl-CoA acyltransferase 1 [EC:2.3.1.16] | (RefSeq) hypothetical protein (A)	3-ketoacyl-CoA thiolase 2, peroxisomal; EC=2.3.1.16; Acetyl-CoA acyltransferase 2; Beta-ketothiolase 2; Peroxisomal 3-oxoacyl-CoA thiolase 2; Peroxisome defective protein 1; Flags: Precursor (At2g33150)	-1.826	
Cs9g06650	K15718 linoleate 9S-lipoxygenase [EC:1.13.11.58] | (RefSeq) hypothetical protein (A)	Linoleate 9S-lipoxygenase 5; EC=1.13.11.58; Lipoxygenase 5; AtLOX5 (At3g22400)		-1.358
Cs9g07970	K15397 3-ketoacyl-CoA synthase [EC:2.3.1.199] | (RefSeq) 3-ketoacyl-CoA synthase 11-like (A)	3-ketoacyl-CoA synthase 2 {ECO:0000303|PubMed:18465198}; KCS-2 {ECO:0000303|PubMed:18465198}; EC=2.3.1.199 {ECO:0000305}; Docosanoic acid synthase {ECO:0000303|PubMed:18786002}; Very long-chain fatty acid condensing enzyme 2 {ECO:0000303|PubMed:18465198}; VLCFA condensing enzyme 2 {ECO:0000303|PubMed:18465198} (At1g04220)	1.453	4.773
Cs9g16550	K00847 fructokinase [EC:2.7.1.4] | (RefSeq) hypothetical protein (A)	Probable fructokinase-7; EC=2.7.1.4 (At5g51830)	-1.148	
novel.2410	K00454 lipoxygenase [EC:1.13.11.12] | (RefSeq) hypothetical protein (A)	LOX21_SOLTU Linoleate 13S-lipoxygenase 2-1, chloroplastic; EC=1.13.11.12; Lipoxygenase 2-1; Flags: Precursor (At3g45140)		-1.693

novel.2411	K00454 lipoxygenase [EC:1.13.11.12] | (RefSeq) hypothetical protein (A)	LOX21_SOLTU Linoleate 13S-lipoxygenase 2-1, chloroplastic; EC=1.13.11.12; Lipoxygenase 2-1; Flags: Precursor (At3g45140)		-1.769
novel.431	K10529 alpha-dioxygenase [EC:1.-.-.-] | (RefSeq) alpha-dioxygenase 1-like (A)	DOX1_ARATH Alpha-dioxygenase 1; Alpha DOX1; EC=1.14.99.-; Fatty acid dioxygenase AlphaDOX1; Pathogen-induced oxygenase; Plant alpha dioxygenase 1; Flags: Precursor (At3g01420)		-2.296
novel.705		PLP2_ARATH Patatin-like protein 2; AtPLP2; EC=3.1.1.-; Patatin-related phospholipase A IIalpha; pPLAIIa; Phospholipase A IIA; AtPLAIIA (At2g26560)	1.440	2.989
orange1.1t00277	K00847 fructokinase [EC:2.7.1.4] | (RefSeq) hypothetical protein (A)	Probable fructokinase-7; EC=2.7.1.4 (At5g51830)	-1.072	
orange1.1t00556	K15397 3-ketoacyl-CoA synthase [EC:2.3.1.199] | (RefSeq) 3-ketoacyl-CoA synthase 1 (A)	3-ketoacyl-CoA synthase 1 {ECO:0000303|PubMed:10074711}; KCS-1 {ECO:0000303|PubMed:10074711}; EC=2.3.1.199 {ECO:0000269|PubMed:10074711, ECO:0000269|PubMed:16765910}; Very long-chain fatty acid condensing enzyme 1 {ECO:0000303|PubMed:10074711}; VLCFA condensing enzyme 1 {ECO:0000303|PubMed:10074711} (At1g01120)		1.797
orange1.1t01733		Nitrogen regulatory protein P-II homolog; Protein PII-like; Flags: Precursor (At4g01900)		-1.061
orange1.1t02607	K10781 fatty acyl-ACP thioesterase B [EC:3.1.2.14 3.1.2.21] | (RefSeq) palmitoyl-acyl carrier protein thioesterase, chloroplastic-like (A)	Palmitoyl-acyl carrier protein thioesterase, chloroplastic; EC=3.1.2.-; 16:0-acyl-carrier protein thioesterase; 16:0-ACP thioesterase; Acyl-[acyl-carrier-protein] hydrolase; Acyl-acyl carrier protein thioesterase B1 {ECO:0000303|PubMed:10859193}; AtFATB1 {ECO:0000303|PubMed:10859193}; Flags: Precursor (At1g08510)	1.540	3.023
orange1.1t03726	K05894 12-oxophytodienoic acid reductase [EC:1.3.1.42] | (RefSeq) putative 12-oxophytodienoate reductase 11 (A)	Putative 12-oxophytodienoate reductase 11; EC=1.3.1.-; OPDA-reductase 11; OsOPR11 (At1g76690)	4.152	
orange1.1t03727	K05894 12-oxophytodienoic acid reductase [EC:1.3.1.42] | (RefSeq) putative 12-oxophytodienoate reductase 11 (A)	Putative 12-oxophytodienoate reductase 11; EC=1.3.1.-; OPDA-reductase 11; OsOPR11 (At1g76690)		1.102
orange1.1t03770	K00454 lipoxygenase [EC:1.13.11.12] | (RefSeq) hypothetical protein (A)	Linoleate 13S-lipoxygenase 2-1, chloroplastic; EC=1.13.11.12; Lipoxygenase 2-1; Flags: Precursor (At3g45140)		-7.503
orange1.1t03775	K00454 lipoxygenase [EC:1.13.11.12] | (RefSeq) hypothetical protein (A)	Linoleate 13S-lipoxygenase 2-1, chloroplastic; EC=1.13.11.12; Lipoxygenase 2-1; Flags: Precursor (At3g45140)	-1.544	
orange1.1t04376	K00454 lipoxygenase [EC:1.13.11.12] | (RefSeq) linoleate 13S-lipoxygenase 2-1, chloroplastic (A)	Linoleate 13S-lipoxygenase 2-1, chloroplastic; EC=1.13.11.12; Lipoxygenase 2-1; Flags: Precursor (At3g45140)	-1.312	-2.168
orange1.1t055		Patatin-like protein 2; AtPLP2; EC=3.1.1.-; Patatin-related phospholipase	1.689	2.763

13		A IIalpha; pPLAIIa; Phospholipase A IIA; AtPLAIIA (At2g26560)		
Unsaturated fatty acid biosynthetic process [GO:0006636; P = 0.6310 (P3CR vs. P3R) and 0.4642 (P5CR vs. P5R)]				
Cs2g28670	K10529 alpha-dioxygenase [EC:1.-.-.-] | (RefSeq) alpha-dioxygenase 1-like (A)	Alpha-dioxygenase 1; Alpha DOX1; EC=1.14.99.-; Fatty acid dioxygenase AlphaDOX1; Pathogen-induced oxygenase; Plant alpha dioxygenase 1; Flags: Precursor (At3g01420)	1.027	-2.590
Cs2g28680	K10529 alpha-dioxygenase [EC:1.-.-.-] | (RefSeq) alpha-dioxygenase 1-like (A)	Alpha-dioxygenase 1; Alpha DOX1; EC=1.14.99.-; Fatty acid dioxygenase AlphaDOX1; Pathogen-induced oxygenase; Plant alpha dioxygenase 1; Flags: Precursor (At3g01420)		-3.541
Cs3g06080	K10525 allene oxide cyclase [EC:5.3.99.6] | (RefSeq) allene oxide cyclase 4, chloroplastic-like (A)	Allene oxide cyclase, chloroplastic {ECO:0000303|Ref.1, ECO:0000303|Ref.2}; OsAOC {ECO:0000303|Ref.1}; EC=5.3.99.6 {ECO:0000269|PubMed:23347338}; Protein COLEOPTILE PHOTOMORPHOGENESIS 2 {ECO:0000303|PubMed:23347338}; Protein HEBIBA {ECO:0000303|PubMed:14605232}; Flags: Precursor (At1g13280)	-1.814	-1.501
novel.431	K10529 alpha-dioxygenase [EC:1.-.-.-] | (RefSeq) alpha-dioxygenase 1-like (A)	DOX1_ARATH Alpha-dioxygenase 1; Alpha DOX1; EC=1.14.99.-; Fatty acid dioxygenase AlphaDOX1; Pathogen-induced oxygenase; Plant alpha dioxygenase 1; Flags: Precursor (At3g01420)		-2.296
Fatty acid degradation [ko00071; P = 0.0239 (P3CR vs. P3R) and 0.0150 (P5CR vs. P5R)]				
Cs1g01290	K00232 acyl-CoA oxidase [EC:1.3.3.6] | (RefSeq) hypothetical protein (A)	Acyl-coenzyme A oxidase 2, peroxisomal; AOX 2; EC=1.3.3.6; Long-chain acyl-CoA oxidase; AtCX2; Flags: Precursor (At5g65110)		1.830
Cs1g24850	K00128 aldehyde dehydrogenase (NAD+) [EC:1.2.1.3] | (RefSeq) aldehyde dehydrogenase family 3 member F1 (A)	Aldehyde dehydrogenase family 3 member F1; EC=1.2.1.3 (At4g36250)	1.544	
Cs3g14390	K00128 aldehyde dehydrogenase (NAD+) [EC:1.2.1.3] | (RefSeq) aldehyde dehydrogenase 5, mitochondrial-like (A)	Translationally-controlled tumor protein homolog; TCTP (At3g16640)		-2.145
Cs3g17940	K00121 S-(hydroxymethyl)glutathione dehydrogenase / alcohol dehydrogenase [EC:1.1.1.284 1.1.1.1] | (RefSeq) alcohol dehydrogenase-like 2 (A)	Alcohol dehydrogenase-like 2; EC=1.1.1.1 {ECO:0000250|UniProtKB:P06525} (At1g22440)		1.096
Cs3g20800	K18857 alcohol dehydrogenase class-P [EC:1.1.1.1] | (RefSeq) alcohol dehydrogenase-like (A)	Alcohol dehydrogenase; EC=1.1.1.1 {ECO:0000250|UniProtKB:P06525} (At1g77120)	-1.541	
Cs3g20810	K18857 alcohol dehydrogenase class-P [EC:1.1.1.1] | (RefSeq) alcohol dehydrogenase (A)	Alcohol dehydrogenase; EC=1.1.1.1 {ECO:0000250|UniProtKB:P06525} (At1g77120)	-2.108	

Cs4g06120	K07425 long-chain fatty acid omega-monooxygenase [EC:1.14.14.80] | (RefSeq) cytochrome P450 CYP72A219 (A)	Cytochrome P450 CYP72A219; EC=1.14.-.-; Cytochrome P450 CYP72A129 (At3g14690)	1.148	1.684
Cs5g14840	K07425 long-chain fatty acid omega-monooxygenase [EC:1.14.14.80] | (RefSeq) cytochrome P450 CYP72A219-like isoform X1 (A)	Cytochrome P450 CYP72A219; EC=1.14.-.-; Cytochrome P450 CYP72A129 (At3g14690)	-1.212	
Cs5g14880	K07425 long-chain fatty acid omega-monooxygenase [EC:1.14.14.80] | (RefSeq) cytochrome P450 CYP72A219-like (A)	Cytochrome P450 CYP72A219; EC=1.14.-.-; Cytochrome P450 CYP72A129 (At3g14690)		-2.798
Cs5g14970	K07425 long-chain fatty acid omega-monooxygenase [EC:1.14.14.80] | (RefSeq) cytochrome P450 CYP72A219-like isoform X1 (A)	Cytochrome P450 CYP72A219; EC=1.14.-.-; Cytochrome P450 CYP72A129 (At3g14690)	2.019	1.157
Cs5g28330	K01897 long-chain acyl-CoA synthetase [EC:6.2.1.3] | (RefSeq) long chain acyl-CoA synthetase 2 (A)	Long chain acyl-CoA synthetase 2; EC=6.2.1.3; Protein Botrytis resistant 1; Protein LATERAL ROOT DEVELOPMENT 2 (At1g49430)	1.162	4.187
Cs6g08640	K00232 acyl-CoA oxidase [EC:1.3.3.6] | (RefSeq) acyl-coenzyme A oxidase 4, peroxisomal-like (A)	Acyl-coenzyme A oxidase 4, peroxisomal; AOX 4; EC=1.3.3.6; G6p; Short-chain acyl-CoA oxidase; AtCX4; AtG6; SAOX (At3g51840)		1.863
Cs7g14420	K00121 S-(hydroxymethyl)glutathione dehydrogenase / alcohol dehydrogenase [EC:1.1.1.284 1.1.1.1] | (RefSeq) alcohol dehydrogenase-like 2 (A)	Alcohol dehydrogenase-like 2; EC=1.1.1.1 {ECO:0000250|UniProtKB:P06525} (At1g22440)		1.229
Cs8g19970	K15401 fatty acid omega-hydroxylase [EC:1.14.-.-] | (RefSeq) hypothetical protein (A)	Cytochrome P450 86A1; EC=1.14.13.205 {ECO:0000269|PubMed:9500987}; CYPLXXXVI; P450-dependent fatty acid omega-hydroxylase; Protein HYDROXYLASE OF ROOT SUBERIZED TISSUE (At5g58860)		3.935
Cs8g20390	K00232 acyl-CoA oxidase [EC:1.3.3.6] | (RefSeq) uncharacterized protein LOC107952742 isoform X1 (A)	Increased DNA methylation 1 {ECO:0000303|PubMed:22700931}; Histone H3 acetyltransferase IDM1 {ECO:0000305}; EC=2.3.1.- {ECO:0000255|PROSITE-ProRule:PRU00532}; Protein ROS4 {ECO:0000303|PubMed:22733760}; Repressor of silencing 4 {ECO:0000303|PubMed:22733760} (At5g58610_2)		1.155
Cs9g04000	K00249 acyl-CoA dehydrogenase [EC:1.3.8.7] | (RefSeq) probable acyl-CoA dehydrogenase IBR3 (A)	Probable acyl-CoA dehydrogenase IBR3 {ECO:0000305}; EC=1.3.99.- {ECO:0000305}; Protein INDOLE-3-BUTYRIC ACID RESPONSE 3 {ECO:0000303|PubMed:17277896} (At3g06810)		1.524
Cs9g05210	K07513 acetyl-CoA acyltransferase 1 [EC:2.3.1.16] | (RefSeq) hypothetical protein (A)	3-ketoacyl-CoA thiolase 2, peroxisomal; EC=2.3.1.16; Acetyl-CoA acyltransferase 2; Beta-ketothiolase 2; Peroxisomal 3-oxoacyl-CoA thiolase 2; Peroxisome defective protein 1; Flags: Precursor (At2g33150)	-1.826	

orange1.1t00260	K20495 long-chain fatty acid omega-monooxygenase [EC:1.14.14.80] | (RefSeq) cytochrome P450 704B1-like (A)	Cytochrome P450 704C1; EC=1.14.-.-; Cytochrome P450 CYPD (At2g45510)	1.259	1.429
orange1.1t00261	K20495 long-chain fatty acid omega-monooxygenase [EC:1.14.13.205] | (RefSeq) cytochrome P450 704B1 (A)	Cytochrome P450 704C1; EC=1.14.-.-; Cytochrome P450 CYPD (At2g45510)	1.378	1.381
orange1.1t04629	K00121 S-(hydroxymethyl)glutathione dehydrogenase / alcohol dehydrogenase [EC:1.1.1.284 1.1.1.1] | (RefSeq) alcohol dehydrogenase-like 2 (A)	Alcohol dehydrogenase-like 2; EC=1.1.1.1 {ECO:0000250|UniProtKB:P06525} (At1g22440)		-6.311
orange1.1t04726	K07425 long-chain fatty acid omega-monooxygenase [EC:1.14.14.80] | (RefSeq) cytochrome P450 CYP72A219-like isoform X1 (A)	Cytochrome P450 CYP72A219; EC=1.14.-.-; Cytochrome P450 CYP72A129 (At3g14690)	1.204	
orange1.1t04729	K07425 long-chain fatty acid omega-monooxygenase [EC:1.14.14.80] | (RefSeq) cytochrome P450 CYP72A219-like (A)	Cytochrome P450 CYP72A219; EC=1.14.-.-; Cytochrome P450 CYP72A129 (At3g14690)		1.731
orange1.1t04743	K07425 long-chain fatty acid omega-monooxygenase [EC:1.14.14.80] | (RefSeq) cytochrome P450 CYP72A219-like isoform X1 (A)	Cytochrome P450 CYP72A219; EC=1.14.-.-; Cytochrome P450 CYP72A129 (At3g14690)		1.165
orange1.1t04745	K07425 long-chain fatty acid omega-monooxygenase [EC:1.14.14.80] | (RefSeq) cytochrome P450 CYP72A219-like isoform X1 (A)	Cytochrome P450 CYP72A219; EC=1.14.-.-; Cytochrome P450 CYP72A129 (At1g42375)	1.030	1.470
Fatty acid catabolic process [GO:0009062; P = 0.8469 (P3CR vs. P3R) and 0.4564 (P5CR vs. P5R)]				
Cs1g01290	K00232 acyl-CoA oxidase [EC:1.3.3.6] | (RefSeq) hypothetical protein (A)	Acyl-coenzyme A oxidase 2, peroxisomal; AOX 2; EC=1.3.3.6; Long-chain acyl-CoA oxidase; AtCX2; Flags: Precursor (At5g65110)		1.830
Cs2g28670	K10529 alpha-dioxygenase [EC:1.-.-.-] | (RefSeq) alpha-dioxygenase 1-like (A)	Alpha-dioxygenase 1; Alpha DOX1; EC=1.14.99.-; Fatty acid dioxygenase AlphaDOX1; Pathogen-induced oxygenase; Plant alpha dioxygenase 1; Flags: Precursor (At3g01420)	1.027	-2.590
Cs2g28680	K10529 alpha-dioxygenase [EC:1.-.-.-] | (RefSeq) alpha-dioxygenase 1-like (A)	Alpha-dioxygenase 1; Alpha DOX1; EC=1.14.99.-; Fatty acid dioxygenase AlphaDOX1; Pathogen-induced oxygenase; Plant alpha dioxygenase 1; Flags: Precursor (At3g01420)		-3.541
Cs6g08640	K00232 acyl-CoA oxidase [EC:1.3.3.6] | (RefSeq) acyl-coenzyme A oxidase 4, peroxisomal-like (A)	Acyl-coenzyme A oxidase 4, peroxisomal; AOX 4; EC=1.3.3.6; G6p; Short-chain acyl-CoA oxidase; AtCX4; AtG6; SAOX (At3g51840)		1.863
Cs9g04000	K00249 acyl-CoA dehydrogenase [EC:1.3.8.7] | (RefSeq) probable acyl-CoA dehydrogenase IBR3 (A)	Probable acyl-CoA dehydrogenase IBR3 {ECO:0000305}; EC=1.3.99.- {ECO:0000305}; Protein INDOLE-3-BUTYRIC ACID RESPONSE 3 {ECO:0000303|PubMed:17277896} (At3g06810)		1.524

Cs9g05210	K07513 acetyl-CoA acyltransferase 1 [EC:2.3.1.16] | (RefSeq) hypothetical protein (A)	3-ketoacyl-CoA thiolase 2, peroxisomal; EC=2.3.1.16; Acetyl-CoA acyltransferase 2; Beta-ketothiolase 2; Peroxisomal 3-oxoacyl-CoA thiolase 2; Peroxisome defective protein 1; Flags: Precursor (At2g33150)	-1.826	
novel.431	K10529 alpha-dioxygenase [EC:1.-.-.-] | (RefSeq) alpha-dioxygenase 1-like (A)	DOX1_ARATH Alpha-dioxygenase 1; Alpha DOX1; EC=1.14.99.-; Fatty acid dioxygenase AlphaDOX1; Pathogen-induced oxygenase; Plant alpha dioxygenase 1; Flags: Precursor (At3g01420)		-2.296
Fatty acid oxidation [GO:0019395; P = 0.5252 (P3CR vs. P3R) and 0.1639 (P5CR vs. P5R)]				
Cs1g01290	K00232 acyl-CoA oxidase [EC:1.3.3.6] | (RefSeq) hypothetical protein (A)	Acyl-coenzyme A oxidase 2, peroxisomal; AOX 2; EC=1.3.3.6; Long-chain acyl-CoA oxidase; AtCX2; Flags: Precursor (At5g65110)		1.830
Cs2g28670	K10529 alpha-dioxygenase [EC:1.-.-.-] | (RefSeq) alpha-dioxygenase 1-like (A)	Alpha-dioxygenase 1; Alpha DOX1; EC=1.14.99.-; Fatty acid dioxygenase AlphaDOX1; Pathogen-induced oxygenase; Plant alpha dioxygenase 1; Flags: Precursor (At3g01420)	1.027	-2.590
Cs2g28680	K10529 alpha-dioxygenase [EC:1.-.-.-] | (RefSeq) alpha-dioxygenase 1-like (A)	Alpha-dioxygenase 1; Alpha DOX1; EC=1.14.99.-; Fatty acid dioxygenase AlphaDOX1; Pathogen-induced oxygenase; Plant alpha dioxygenase 1; Flags: Precursor (At3g01420)		-3.541
Cs5g10160	no KO assigned | (RefSeq) protein HOTHEAD (A)	Protein HOTHEAD; Protein ADHESION OF CALYX EDGES; Flags: Precursor (At1g12570)	2.459	3.883
Cs6g08640	K00232 acyl-CoA oxidase [EC:1.3.3.6] | (RefSeq) acyl-coenzyme A oxidase 4, peroxisomal-like (A)	Acyl-coenzyme A oxidase 4, peroxisomal; AOX 4; EC=1.3.3.6; G6p; Short-chain acyl-CoA oxidase; AtCX4; AtG6; SAOX (At3g51840)		1.863
Cs7g03710	K00108 choline dehydrogenase [EC:1.1.99.1] | (RefSeq) protein HOTHEAD-like (A)	Protein HOTHEAD; Protein ADHESION OF CALYX EDGES; Flags: Precursor (At1g14190)		1.518
Cs9g04000	K00249 acyl-CoA dehydrogenase [EC:1.3.8.7] | (RefSeq) probable acyl-CoA dehydrogenase IBR3 (A)	Probable acyl-CoA dehydrogenase IBR3 {ECO:0000305}; EC=1.3.99.- {ECO:0000305}; Protein INDOLE-3-BUTYRIC ACID RESPONSE 3 {ECO:0000303|PubMed:17277896} (At3g06810)		1.524
Cs9g05210	K07513 acetyl-CoA acyltransferase 1 [EC:2.3.1.16] | (RefSeq) hypothetical protein (A)	3-ketoacyl-CoA thiolase 2, peroxisomal; EC=2.3.1.16; Acetyl-CoA acyltransferase 2; Beta-ketothiolase 2; Peroxisomal 3-oxoacyl-CoA thiolase 2; Peroxisome defective protein 1; Flags: Precursor (At2g33150)	-1.826	
Cs9g16620	K21270 (R)-mandelonitrile oxidase [EC:1.1.3.49] | (RefSeq) protein HOTHEAD (A)	Protein HOTHEAD; Protein ADHESION OF CALYX EDGES; Flags: Precursor (At5g51950)	1.597	4.643
novel.431	K10529 alpha-dioxygenase [EC:1.-.-.-] | (RefSeq) alpha-dioxygenase 1-like (A)	DOX1_ARATH Alpha-dioxygenase 1; Alpha DOX1; EC=1.14.99.-; Fatty acid dioxygenase AlphaDOX1; Pathogen-induced oxygenase; Plant alpha dioxygenase 1; Flags: Precursor (At3g01420)		-2.296

Membrane lipid biosynthetic process [GO:0046467; P = 0.9962 (P3CR vs. P3R) and 0.9512 (P5CR vs. P5R)]				
Cs1g20370	K10703 very-long-chain (3R)-3-hydroxyacyl-CoA dehydratase [EC:4.2.1.134] | (RefSeq) very-long-chain (3R)-3-hydroxyacyl-CoA dehydratase PASTICCINO 2 (A)	Very-long-chain (3R)-3-hydroxyacyl-CoA dehydratase PASTICCINO 2 {ECO:0000305}; EC=4.2.1.134 {ECO:0000305|PubMed:18799749}; 3-hydroxyacyl-CoA dehydratase PASTICCINO 2; AtPAS2; HACD; HCD; Protein PEPINO; PEP; Protein tyrosine phosphatase-like protein (At5g10480)		1.075
Cs2g06240	K04712 sphingolipid 4-desaturase/C4-monooxygenase [EC:1.14.19.17 1.14.18.5] | (RefSeq) sphingolipid delta(4)-desaturase DES1-like (A)	Sphingolipid delta(4)-desaturase DES1-like; EC=1.14.19.17 {ECO:0000305|PubMed:18978071} (At4g04930)	-2.252	
Cs3g04040	K10249 elongation of very long chain fatty acids protein 4 [EC:2.3.1.199] | (RefSeq) elongation of fatty acids protein A-like (A)	Elongation of fatty acids protein 3-like; Protein ELO3-like; EC=2.3.1.-; Protein HIGH EXPRESSION OF OSMOTICALLY RESPONSIVE GENES 3; Very long-chain fatty acid condensing enzyme HOS3; VLCFA condensing enzyme HOS3 (At3g06470)		-2.061
Cs3g23660	K15728 phosphatidate phosphatase LPIN [EC:3.1.3.4] | (RefSeq) phosphatidate phosphatase PAH2 (A)	Phosphatidate phosphatase PAH2; EC=3.1.3.4; Phosphatidic acid phosphohydrolase 2; AtPAH2 (At5g42870)		1.656
Cs4g05250	K03715 1,2-diacylglycerol 3-beta-galactosyltransferase [EC:2.4.1.46] | (RefSeq) hypothetical protein (A)	Monogalactosyldiacylglycerol synthase 2, chloroplastic; AtMGD2; EC=2.4.1.46; MGDG synthase type B; Flags: Precursor (At5g20410)		1.204
Cs8g13690	K22697 sphingomyelin synthase-related protein 1 | (RefSeq) hypothetical protein (A)	Phosphatidylinositol:ceramide inositolphosphotransferase 2; EC=2.7.8.-; Inositol-phosphorylceramide synthase 2; AtIPCS2; IPC synthase 2; Protein ENHANCING RPW8-MEDIATED HR-LIKE CELL DEATH 1; Sphingolipid synthase 2 (At2g37940)		-2.071
Neutral lipid biosynthetic process [GO:0046460; P = 0.6844 (P3CR vs. P3R) and 0.1775 (P5CR vs. P5R)]				
Cs2g14090		Acyltransferase-like protein At3g26840, chloroplastic; EC=2.3.1.-; Flags: Precursor (At5g41120)		1.057
Cs2g14100	K10870 RAD51-like protein 2 | (RefSeq) DNA repair protein RAD51 homolog 3-like (A)	Acyltransferase-like protein At3g26840, chloroplastic; EC=2.3.1.-; Flags: Precursor (At3g26840)		1.512
Cs2g14120	K10870 RAD51-like protein 2 | (RefSeq) DNA repair protein RAD51 homolog 3-like (A)	Acyltransferase-like protein At3g26840, chloroplastic; EC=2.3.1.-; Flags: Precursor (At5g41120)		1.398
Cs6g20790	K09285 AP2-like factor, ANT lineage | (RefSeq) AP2-like ethylene-responsive transcription factor At1g16060 (A)	Ethylene-responsive transcription factor WRI1; Protein ACTIVATOR OF SPORAMIN::LUC 1; Protein WRINKLED 1 (At1g16060)	1.439	-2.381
Cs7g04300	K09285 AP2-like factor, ANT lineage | (RefSeq) AP2-like	Ethylene-responsive transcription factor WRI1; Protein ACTIVATOR OF		-5.678

	ethylene-responsive transcription factor At1g79700 (A)	SPORAMIN::LUC 1; Protein WRINKLED 1 (At5g17430)		
orange1.1t01771	K22849 diacylglycerol O-acyltransferase 3, plant [EC:2.3.1.20] | (RefSeq) hypothetical protein (A)	Diacylglycerol O-acyltransferase 3, cytosolic {ECO:0000305}; AtDGAT3 {ECO:0000303|PubMed:22760209}; EC=2.3.1.20 {ECO:0000305|PubMed:22760209} (At1g48300)	1.203	1.834
Phospholipid biosynthetic process [GO:0008654; P = 0.8446 (P3CR vs. P3R) and 0.7154 (P5CR vs. P5R)]				
Cs2g11760	K17807 mitochondrial translocator assembly and maintenance protein 41 | (RefSeq) phosphatidate cytidylyltransferase, mitochondrial (A)	(At3g47630)	-1.241	
Cs3g03640	K13789 geranylgeranyl diphosphate synthase, type II [EC:2.5.1.1 2.5.1.10 2.5.1.29] | (RefSeq) geranylgeranyl pyrophosphate synthase, chloroplastic-like (A)	Geranylgeranyl pyrophosphate synthase, chloroplastic; GGPP synthase; GGPS; EC=2.5.1.-; (2E,6E)-farnesyl diphosphate synthase; Dimethylallyltranstransferase; EC=2.5.1.1; Farnesyl diphosphate synthase; Farnesyltranstransferase; EC=2.5.1.29; Geranyltranstransferase; EC=2.5.1.10; Flags: Precursor (At4g36810)	3.498	
Cs3g23660	K15728 phosphatidate phosphatase LPIN [EC:3.1.3.4] | (RefSeq) phosphatidate phosphatase PAH2 (A)	Phosphatidate phosphatase PAH2; EC=3.1.3.4; Phosphatidic acid phosphohydrolase 2; AtPAH2 (At5g42870)		1.656
Cs4g10240	K13789 geranylgeranyl diphosphate synthase, type II [EC:2.5.1.1 2.5.1.10 2.5.1.29] | (RefSeq) hypothetical protein (A)	Geranylgeranyl pyrophosphate synthase, chloroplastic; GGPP synthase; GGPS; EC=2.5.1.-; (2E,6E)-farnesyl diphosphate synthase; Dimethylallyltranstransferase; EC=2.5.1.1; Farnesyl diphosphate synthase; Farnesyltranstransferase; EC=2.5.1.29; Geranyltranstransferase; EC=2.5.1.10; Flags: Precursor (At2g18620)	-1.409	-1.909
Cs4g10970	K13509 lysophosphatidate acyltransferase [EC:2.3.1.51] | (RefSeq) 1-acyl-sn-glycerol-3-phosphate acyltransferase (A)	1-acyl-sn-glycerol-3-phosphate acyltransferase; 1-AGP acyltransferase; 1-AGPAT; EC=2.3.1.51; Lysophosphatidic acid acyltransferase; LPAAT (YDL052c)		-1.282
Cs6g07920	K13508 glycerol-3-phosphate acyltransferase [EC:2.3.1.15 2.3.1.198] | (RefSeq) hypothetical protein (A)	Glycerol-3-phosphate 2-O-acyltransferase 6; AtGPAT6; EC=2.3.1.198; Glycerol-3-phosphate acyltransferase 6 (At2g38110)	1.642	4.893
Cs6g17510	K13789 geranylgeranyl diphosphate synthase, type II [EC:2.5.1.1 2.5.1.10 2.5.1.29] | (RefSeq) geranylgeranyl pyrophosphate synthase 7, chloroplastic-like (A)	Geranylgeranyl pyrophosphate synthase, chloroplastic; GGPP synthase; GGPS; EC=2.5.1.-; (2E,6E)-farnesyl diphosphate synthase; Dimethylallyltranstransferase; EC=2.5.1.1; Farnesyl diphosphate synthase; Farnesyltranstransferase; EC=2.5.1.29; Geranyltranstransferase; EC=2.5.1.10; Flags: Precursor (At4g36810)		-6.069
Cs6g20410	K13508 glycerol-3-phosphate acyltransferase [EC:2.3.1.15 2.3.1.198] | (RefSeq) glycerol-3-phosphate 2-O-acyltransferase 6 (A)	Glycerol-3-phosphate 2-O-acyltransferase 6; AtGPAT6; EC=2.3.1.198; Glycerol-3-phosphate acyltransferase 6 (At2g38110)		-7.533

Cs7g17090	K13508 glycerol-3-phosphate acyltransferase [EC:2.3.1.15 2.3.1.198] | (RefSeq) glycerol-3-phosphate acyltransferase 1 (A)	Glycerol-3-phosphate acyltransferase 1; AtGPAT1; EC=2.3.1.15 (At1g06520)		1.529
Cs7g31730	K13508 glycerol-3-phosphate acyltransferase [EC:2.3.1.15 2.3.1.198] | (RefSeq) hypothetical protein (A)	Glycerol-3-phosphate acyltransferase 7; AtGPAT7; EC=2.3.1.15 (At5g06090)	1.020	4.530
Cs8g06970	K13513 lysocardiolipin and lysophospholipid acyltransferase [EC:2.3.1.- 2.3.1.51] | (RefSeq) probable 1-acyl-sn-glycerol-3-phosphate acyltransferase 5 (A)	Probable 1-acyl-sn-glycerol-3-phosphate acyltransferase 5; EC=2.3.1.51; Lysophosphatidyl acyltransferase 5 (At3g18850)	-1.227	
Cs8g07030	K03527 4-hydroxy-3-methylbut-2-en-1-yl diphosphate reductase [EC:1.17.7.4] | (RefSeq) 4-hydroxy-3-methylbut-2-enyl diphosphate reductase, chloroplastic-like isoform X1 (A)	4-hydroxy-3-methylbut-2-enyl diphosphate reductase, chloroplastic; EC=1.17.7.4; Flags: Precursor (At4g34350)		-1.163
novel.1333	K13789 geranylgeranyl diphosphate synthase, type II [EC:2.5.1.1 2.5.1.10 2.5.1.29] | (RefSeq) geranylgeranyl pyrophosphate synthase, chloroplastic-like (A)	GGPPS_HEVBR Geranylgeranyl pyrophosphate synthase, chloroplastic; GGPP synthase; GGPS; EC=2.5.1.-; (2E,6E)-farnesyl diphosphate synthase; Dimethylallyltranstransferase; EC=2.5.1.1; Farnesyl diphosphate synthase; Farnesyltranstransferase; EC=2.5.1.29; Geranyltranstransferase; EC=2.5.1.10; Flags: Precursor (At4g36810)		-1.435
orange1.1t00198	K00981 phosphatidate cytidylyltransferase [EC:2.7.7.41] | (RefSeq) hypothetical protein (A)	Phosphatidate cytidylyltransferase 4, chloroplastic {ECO:0000303|PubMed:20442275}; EC=2.7.7.41 {ECO:0000269|PubMed:20442275}; CDP-DAG synthase 4; CDP-DG synthase 4; CDP-diacylglycerol synthase 4; CDS4; CDP-diglyceride pyrophosphorylase 4; CDP-diglyceride synthase 4; CTP:phosphatidate cytidylyltransferase 4; Flags: Precursor (At2g45150)		1.024
orange1.1t00375	K13508 glycerol-3-phosphate acyltransferase [EC:2.3.1.15 2.3.1.198] | (RefSeq) probable glycerol-3-phosphate acyltransferase 8 (A)	Probable glycerol-3-phosphate acyltransferase 8; EC=2.3.1.15 (At1g01610)	1.009	3.308
orange1.1t03552	K01613 phosphatidylserine decarboxylase [EC:4.1.1.65] | (RefSeq) phosphatidylserine decarboxylase proenzyme 2-like (A)	Phosphatidylserine decarboxylase proenzyme 2; EC=4.1.1.65 {ECO:0000255|HAMAP-Rule:MF_03209}; Contains: Phosphatidylserine decarboxylase 2 beta chain; Contains: Phosphatidylserine decarboxylase 2 alpha chain (At5g57190)		1.130
orange1.1t04957	K13789 geranylgeranyl diphosphate synthase, type II [EC:2.5.1.1 2.5.1.10 2.5.1.29] | (RefSeq) geranylgeranyl pyrophosphate synthase, chloroplastic/chromoplastic-like (A)	Geranylgeranyl pyrophosphate synthase, chloroplastic; GGPP synthase; GGPS; EC=2.5.1.-; (2E,6E)-farnesyl diphosphate synthase; Dimethylallyltranstransferase; EC=2.5.1.1; Farnesyl diphosphate synthase; Farnesyltranstransferase; EC=2.5.1.29; Geranyltranstransferase;		-3.818

		EC=2.5.1.10; Flags: Precursor (At4g36810)		
Lipid catabolic process [GO:0016042; P = 0.1831 (P3CR vs. P3R) and 0.0008 (P5CR vs. P5R)]				
Cs1g01290	K00232 acyl-CoA oxidase [EC:1.3.3.6] | (RefSeq) hypothetical protein (A)	Acyl-coenzyme A oxidase 2, peroxisomal; AOX 2; EC=1.3.3.6; Long-chain acyl-CoA oxidase; AtCX2; Flags: Precursor (At5g65110)		1.830
Cs1g01505	K05677 ATP-binding cassette, subfamily D (ALD), member 3 | (RefSeq) ABC transporter D family member 1 (A)	ABC transporter D family member 1; ABC transporter ABCD.1; AtABCD1; EC=3.6.3.47; Peroxisomal ABC transporter 1; AtPXA1; Protein ACETATE NON-UTILIZING 2; Protein COMATOSE; Protein PEROXISOME DEFECTIVE 3; Ped3p (At4g39850)		1.085
Cs1g16000	K00679 phospholipid:diacylglycerol acyltransferase [EC:2.3.1.158] | (RefSeq) hypothetical protein (A)	GDSL esterase/lipase APG; EC=3.1.1.-; Extracellular lipase APG; Flags: Precursor (At3g16370)		-1.270
Cs1g17310	K13806 sn1-specific diacylglycerol lipase [EC:3.1.1.-] | (RefSeq) sn1-specific diacylglycerol lipase beta-like (A)	(At3g14070_1)		1.382
Cs1g18410	K01115 phospholipase D1/2 [EC:3.1.4.4] | (RefSeq) phospholipase D alpha 4 (A)	Phospholipase D alpha 4 {ECO:0000303|PubMed:11891260}; AtPLDalpha4 {ECO:0000303|PubMed:11891260}; PLD alpha 4 {ECO:0000303|PubMed:11891260}; EC=3.1.4.4 {ECO:0000250|UniProtKB:Q38882}; PLDalpha3; Phospholipase D epsilon {ECO:0000303|PubMed:19143999}; AtPLDepsilon {ECO:0000303|PubMed:19143999}; PLD epsilon {ECO:0000303|PubMed:19143999} (At1g55180)	1.605	2.372
Cs1g22940		Patatin-like protein 1; EC=3.1.1.- (At4g37050)		3.398
Cs1g23250	K00679 phospholipid:diacylglycerol acyltransferase [EC:2.3.1.158] | (RefSeq) hypothetical protein (A)	GDSL esterase/lipase At2g23540; EC=3.1.1.-; Extracellular lipase At2g23540; Flags: Precursor (At2g23540)		3.813
Cs1g24480	K07437 cytochrome P450 family 26 subfamily A | (RefSeq) cytochrome P450 90A1-like (A)	Abscisic acid 8'-hydroxylase 3; ABA 8'-hydroxylase 3; EC=1.14.13.93; Cytochrome P450 707A7; OsABA8ox3 (At1g19630)		1.028
Cs1g25490	K07437 cytochrome P450 family 26 subfamily A | (RefSeq) cytochrome P450 90A1 (A)	Abscisic acid 8'-hydroxylase 1; ABA 8'-hydroxylase 1; EC=1.14.13.93; Cytochrome P450 707A1 (At1g19630)	3.253	
Cs2g05250	K10691 E3 ubiquitin-protein ligase UBR4 [EC:2.3.2.27] | (RefSeq) LOC109762802; auxin transport protein BIG (A)	GDSL esterase/lipase At5g45950; EC=3.1.1.-; Extracellular lipase At5g45950; Flags: Precursor (At5g45950)	-1.769	-1.729
Cs2g08310	K16818 phospholipase A1 [EC:3.1.1.32] | (RefSeq) hypothetical protein (A)	Phospholipase A1-Igamma1, chloroplastic; EC=3.1.1.-; Flags: Precursor (At1g30370)	1.088	
Cs2g11270	K16815 calcium-independent phospholipase A2-gamma | (RefSeq) hypothetical protein (A)	Patatin-like protein 1; EC=3.1.1.- (At2g26560)	1.612	

Cs2g20450	K01052 lysosomal acid lipase/cholesteryl ester hydrolase [EC:3.1.1.13] | (RefSeq) triacylglycerol lipase 2-like (A)	Triacylglycerol lipase 2; EC=3.1.1.3; Flags: Precursor (At5g14180)	1.326	
Cs2g20470	K01052 lysosomal acid lipase/cholesteryl ester hydrolase [EC:3.1.1.13] | (RefSeq) hypothetical protein (A)	Triacylglycerol lipase 2; EC=3.1.1.3; Flags: Precursor (At5g14180)	-1.827	
Cs2g20480	K01052 lysosomal acid lipase/cholesteryl ester hydrolase [EC:3.1.1.13] | (RefSeq) hypothetical protein (A)	Triacylglycerol lipase 2; EC=3.1.1.3; Flags: Precursor (At5g14180)		-6.743
Cs2g25040	K14452 gastric triacylglycerol lipase [EC:3.1.1.3] | (RefSeq) triacylglycerol lipase 2-like (A)	Triacylglycerol lipase 2; EC=3.1.1.3; Flags: Precursor (At5g14180)		-1.057
Cs2g28670	K10529 alpha-dioxygenase [EC:1.-.-.-] | (RefSeq) alpha-dioxygenase 1-like (A)	Alpha-dioxygenase 1; Alpha DOX1; EC=1.14.99.-; Fatty acid dioxygenase AlphaDOX1; Pathogen-induced oxygenase; Plant alpha dioxygenase 1; Flags: Precursor (At3g01420)	1.027	-2.590
Cs2g28680	K10529 alpha-dioxygenase [EC:1.-.-.-] | (RefSeq) alpha-dioxygenase 1-like (A)	Alpha-dioxygenase 1; Alpha DOX1; EC=1.14.99.-; Fatty acid dioxygenase AlphaDOX1; Pathogen-induced oxygenase; Plant alpha dioxygenase 1; Flags: Precursor (At3g01420)		-3.541
Cs2g29630	K01114 phospholipase C [EC:3.1.4.3] | (RefSeq) non-specific phospholipase C2 (A)	Non-specific phospholipase C2; EC=3.1.-.-; Flags: Precursor (At2g26870)		-1.356
Cs3g14430	K21026 acetylajmaline esterase [EC:3.1.1.80] | (RefSeq) hypothetical protein (A)	GDSL esterase/lipase At5g03980; EC=3.1.1.-; Extracellular lipase At5g03980; Flags: Precursor (At5g03980)		-1.493
Cs3g21210	K07437 cytochrome P450 family 26 subfamily A | (RefSeq) abscisic acid 8'-hydroxylase 1-like (A)	Abscisic acid 8'-hydroxylase 3; ABA 8'-hydroxylase 3; EC=1.14.13.93; Cytochrome P450 707A3 (At5g45340)	1.449	-3.358
Cs3g23440	K12863 protein CWC15 | (RefSeq) protein CWC15 (A)	Phospholipase A1-IIalpha; EC=3.1.1.- (At5g67050)		-1.334
Cs3g23530	K09843 (+)-abscisic acid 8'-hydroxylase [EC:1.14.13.93] | (RefSeq) hypothetical protein (A)	Abscisic acid 8'-hydroxylase 3; ABA 8'-hydroxylase 3; EC=1.14.13.93; Cytochrome P450 707A7; OsABA8ox3 (At3g19270)		-1.446
Cs3g26460	K14209 solute carrier family 36 (proton-coupled amino acid transporter) | (RefSeq) uncharacterized protein LOC109773794 (A)	GDSL esterase/lipase EXL3; EC=3.1.1.-; Family II extracellular lipase 3; Family II lipase EXL3; Flags: Precursor (At1g75900)		3.089
Cs3g26720	K01115 phospholipase D1/2 [EC:3.1.4.4] | (RefSeq) hypothetical protein (A)	Phospholipase D delta {ECO:0000303|PubMed:11891260}; AtPLDdelta {ECO:0000303|PubMed:11891260}; PLD delta {ECO:0000303|PubMed:11891260}; EC=3.1.4.4 {ECO:0000269|PubMed:11706190, ECO:0000269|PubMed:12397060} (At4g35790)	-1.036	
Cs4g06810	K00679 phospholipid:diacylglycerol acyltransferase [EC:2.3.1.158] | (RefSeq) hypothetical protein (A)	GDSL esterase/lipase 5; EC=3.1.1.-; Extracellular lipase 5; Flags: Precursor (At1g53920)	1.018	-5.873

Cs4g10140	K00679 phospholipid:diacylglycerol acyltransferase [EC:2.3.1.158] | (RefSeq) hypothetical protein (A)	GDSL esterase/lipase At5g55050; EC=3.1.1.-; Extracellular lipase At5g55050; Flags: Precursor (At5g55050)		-3.134
Cs4g19460	K17913 carlactone synthase / all-trans-10'-apo-beta-carotenal 13,14-cleaving dioxygenase [EC:1.13.11.69 1.13.11.70] | (RefSeq) hypothetical protein (A)	Carotenoid cleavage dioxygenase 8 homolog B, chloroplastic; OsCCD8b; EC=1.13.11.69 {ECO:0000269|PubMed:22422982}; EC=1.13.11.70 {ECO:0000269|PubMed:18637791}; Protein DWARF-10; Protein MAX4 homolog; Flags: Precursor (At4g32810)	1.942	1.251
Cs5g10750	K10691 E3 ubiquitin-protein ligase UBR4 [EC:2.3.2.27] | (RefSeq) LOC109762802; auxin transport protein BIG (A)	GDSL esterase/lipase At1g74460; EC=3.1.1.-; Extracellular lipase At1g74460; Flags: Precursor (At1g74460)	1.276	4.011
Cs5g19660	K17108 non-lysosomal glucosylceramidase [EC:3.2.1.45] | (RefSeq) non-lysosomal glucosylceramidase (A)	(At1g33700)	1.025	
Cs5g30350	K16818 phospholipase A1 [EC:3.1.1.32] | (RefSeq) phospholipase A(1) DAD1, chloroplastic (A)	Phospholipase A1-Igamma2, chloroplastic; EC=3.1.1.-; Flags: Precursor (At1g06800)		-1.286
Cs6g08640	K00232 acyl-CoA oxidase [EC:1.3.3.6] | (RefSeq) acyl-coenzyme A oxidase 4, peroxisomal-like (A)	Acyl-coenzyme A oxidase 4, peroxisomal; AOX 4; EC=1.3.3.6; G6p; Short-chain acyl-CoA oxidase; AtCX4; AtG6; SAOX (At3g51840)		1.863
Cs6g15160	K16280 E3 ubiquitin-protein ligase RGLG [EC:2.3.2.27] | (RefSeq) E3 ubiquitin-protein ligase RGLG2-like isoform X1 (A)	GDSL esterase/lipase At5g03610; EC=3.1.1.-; Extracellular lipase At5g03610; Flags: Precursor (At5g03610)		-2.099
Cs6g17290	K00679 phospholipid:diacylglycerol acyltransferase [EC:2.3.1.158] | (RefSeq) hypothetical protein (A)	GDSL esterase/lipase At5g37690; EC=3.1.1.-; Extracellular lipase At5g37690; Flags: Precursor (At1g74460)	1.333	4.012
Cs6g19380	K09843 (+)-abscisic acid 8'-hydroxylase [EC:1.14.13.93] | (RefSeq) CYP707A1; abscisic acid 8'-hydroxylase 1-like (A)	Abscisic acid 8'-hydroxylase 1; ABA 8'-hydroxylase 1; EC=1.14.13.93; Cytochrome P450 707A1 (At5g45340)		1.560
Cs6g19840	K00679 phospholipid:diacylglycerol acyltransferase [EC:2.3.1.158] | (RefSeq) hypothetical protein (A)	GDSL esterase/lipase 7; EC=3.1.1.-; Extracellular lipase 7; Flags: Precursor (At5g15720)		-4.352
Cs7g01700	K00465 carotenoid 9,10(9',10')-cleavage dioxygenase 1 [EC:1.13.11.-] | (RefSeq) carotenoid 9,10(9',10')-cleavage dioxygenase 1-like (A)	Carotenoid 9,10(9',10')-cleavage dioxygenase 1; EC=1.14.99.n4; AtCCD1; Neoxanthin cleavage enzyme NC1; AtNCED1 (At3g63520)		-1.066
Cs7g09490	K01206 alpha-L-fucosidase [EC:3.2.1.51] | (RefSeq) alpha-L-fucosidase 3-like (A)	GDSL esterase/lipase At3g26430; EC=3.1.1.-; Extracellular lipase At3g26430; Flags: Precursor (At3g26430)		-1.373
Cs7g15620	K01114 phospholipase C [EC:3.1.4.3] | (RefSeq) non-specific phospholipase C4-like (A)	Non-specific phospholipase C4; EC=3.1.4.3 (At3g03530)	1.786	2.198
Cs7g18030	K00679 phospholipid:diacylglycerol acyltransferase [EC:2.3.1.158] | (RefSeq) hypothetical protein (A)	GDSL esterase/lipase At5g22810; EC=3.1.1.-; Extracellular lipase At5g22810; Flags: Precursor (At5g22810)	2.079	6.407
Cs7g20810	K16280 E3 ubiquitin-protein ligase RGLG [EC:2.3.2.27] |	GDSL esterase/lipase At5g03610; EC=3.1.1.-; Extracellular lipase		5.090

	(RefSeq) E3 ubiquitin-protein ligase RGLG2-like isoform X1 (A)	At5g03610; Flags: Precursor (At5g03610)		
Cs7g24420	K00679 phospholipid:diacylglycerol acyltransferase [EC:2.3.1.158] | (RefSeq) hypothetical protein (A)	GDSL esterase/lipase At1g06990; EC=3.1.1.-; Extracellular lipase At1g06990; Flags: Precursor (At1g06990)		6.920
Cs7g26370	K01052 lysosomal acid lipase/cholesteryl ester hydrolase [EC:3.1.1.13] | (RefSeq) hypothetical protein (A)	Triacylglycerol lipase 2; EC=3.1.1.3; Flags: Precursor (At1g18460)		-1.478
Cs8g01950	K00679 phospholipid:diacylglycerol acyltransferase [EC:2.3.1.158] | (RefSeq) hypothetical protein (A)	GDSL esterase/lipase At5g37690; EC=3.1.1.-; Extracellular lipase At5g37690; Flags: Precursor (At5g55050)	1.269	-2.864
Cs8g04910	K01052 lysosomal acid lipase/cholesteryl ester hydrolase [EC:3.1.1.13] | (RefSeq) triacylglycerol lipase 1 (A)	Triacylglycerol lipase 1; EC=3.1.1.3; Flags: Precursor (At2g15230)		1.128
Cs8g05940	K09843 (+)-abscisic acid 8'-hydroxylase [EC:1.14.13.93] | (RefSeq) CYP707A3; abscisic acid 8'-hydroxylase 4-like (A)	Abscisic acid 8'-hydroxylase 4; ABA 8'-hydroxylase 4; EC=1.14.13.93; Cytochrome P450 707A4 (At3g19270)		-1.037
Cs8g17080	K16815 calcium-independent phospholipase A2-gamma | (RefSeq) hypothetical protein (A)	Patatin-like protein 2; AtPLP2; EC=3.1.1.-; Patatin-related phospholipase A IIalpha; pPLAIIa; Phospholipase A IIA; AtPLAIIA (At2g26560)		1.072
Cs8g17090	K16815 calcium-independent phospholipase A2-gamma | (RefSeq) hypothetical protein (A)	Patatin-like protein 2; AtPLP2; EC=3.1.1.-; Patatin-related phospholipase A IIalpha; pPLAIIa; Phospholipase A IIA; AtPLAIIA (At2g26560)		1.375
Cs8g20250	K05857 phosphatidylinositol phospholipase C, delta [EC:3.1.4.11] | (RefSeq) phosphoinositide phospholipase C 4-like (A)	Phosphoinositide phospholipase C 4; EC=3.1.4.11; Phosphoinositide phospholipase PLC4; AtPLC4; PI-PLC4 (At5g58700)		-1.439
Cs9g04000	K00249 acyl-CoA dehydrogenase [EC:1.3.8.7] | (RefSeq) probable acyl-CoA dehydrogenase IBR3 (A)	Probable acyl-CoA dehydrogenase IBR3 {ECO:0000305}; EC=1.3.99.- {ECO:0000305}; Protein INDOLE-3-BUTYRIC ACID RESPONSE 3 {ECO:0000303|PubMed:17277896} (At3g06810)		1.524
Cs9g05210	K07513 acetyl-CoA acyltransferase 1 [EC:2.3.1.16] | (RefSeq) hypothetical protein (A)	3-ketoacyl-CoA thiolase 2, peroxisomal; EC=2.3.1.16; Acetyl-CoA acyltransferase 2; Beta-ketothiolase 2; Peroxisomal 3-oxoacyl-CoA thiolase 2; Peroxisome defective protein 1; Flags: Precursor (At2g33150)	-1.826	
novel.431	K10529 alpha-dioxygenase [EC:1.-.-.-] | (RefSeq) alpha-dioxygenase 1-like (A)	DOX1_ARATH Alpha-dioxygenase 1; Alpha DOX1; EC=1.14.99.-; Fatty acid dioxygenase AlphaDOX1; Pathogen-induced oxygenase; Plant alpha dioxygenase 1; Flags: Precursor (At3g01420)		-2.296
novel.705		PLP2_ARATH Patatin-like protein 2; AtPLP2; EC=3.1.1.-; Patatin-related phospholipase A IIalpha; pPLAIIa; Phospholipase A IIA; AtPLAIIA (At2g26560)	1.440	2.989
orange1.1t00747	K14674 TAG lipase / steryl ester hydrolase / phospholipase A2 / LPA acyltransferase [EC:3.1.1.3 3.1.1.13 3.1.1.4 2.3.1.51] | (RefSeq) triacylglycerol lipase SDP1 (A)	Triacylglycerol lipase SDP1; EC=3.1.1.3; Protein SUGAR-DEPENDENT 1 (At5g04040)		-1.368

orange1.1t034 75	K16818 phospholipase A1 [EC:3.1.1.32] | (RefSeq) phospholipase A(1) DAD1, chloroplastic-like (A)	Phospholipase A(1) DAD1, chloroplastic; EC=3.1.1.32; Phospholipase A1-Ibeta1; Protein DEFECTIVE IN ANTHER DEHISCENCE 1; AtDAD1; Flags: Precursor (At4g13550)	1.937	
orange1.1t055 13		Patatin-like protein 2; AtPLP2; EC=3.1.1.-; Patatin-related phospholipase A IIalpha; pPLAIIa; Phospholipase A IIA; AtPLAIIA (At2g26560)	1.689	2.763
Lipid oxidation [GO:0034440; P = 0.6266 (P3CR vs. P3R) and 0.1628 (P5CR vs. P5R)]				
Cs1g01290	K00232 acyl-CoA oxidase [EC:1.3.3.6] | (RefSeq) hypothetical protein (A)	Acyl-coenzyme A oxidase 2, peroxisomal; AOX 2; EC=1.3.3.6; Long-chain acyl-CoA oxidase; AtCX2; Flags: Precursor (At5g65110)		1.830
Cs2g28670	K10529 alpha-dioxygenase [EC:1.-.-.-] | (RefSeq) alpha-dioxygenase 1-like (A)	Alpha-dioxygenase 1; Alpha DOX1; EC=1.14.99.-; Fatty acid dioxygenase AlphaDOX1; Pathogen-induced oxygenase; Plant alpha dioxygenase 1; Flags: Precursor (At3g01420)	1.027	-2.590
Cs2g28680	K10529 alpha-dioxygenase [EC:1.-.-.-] | (RefSeq) alpha-dioxygenase 1-like (A)	Alpha-dioxygenase 1; Alpha DOX1; EC=1.14.99.-; Fatty acid dioxygenase AlphaDOX1; Pathogen-induced oxygenase; Plant alpha dioxygenase 1; Flags: Precursor (At3g01420)		-3.541
Cs5g10160	no KO assigned | (RefSeq) protein HOTHEAD (A)	Protein HOTHEAD; Protein ADHESION OF CALYX EDGES; Flags: Precursor (At1g12570)	2.459	3.883
Cs6g08640	K00232 acyl-CoA oxidase [EC:1.3.3.6] | (RefSeq) acyl-coenzyme A oxidase 4, peroxisomal-like (A)	Acyl-coenzyme A oxidase 4, peroxisomal; AOX 4; EC=1.3.3.6; G6p; Short-chain acyl-CoA oxidase; AtCX4; AtG6; SAOX (At3g51840)		1.863
Cs7g03710	K00108 choline dehydrogenase [EC:1.1.99.1] | (RefSeq) protein HOTHEAD-like (A)	Protein HOTHEAD; Protein ADHESION OF CALYX EDGES; Flags: Precursor (At1g14190)		1.518
Cs9g04000	K00249 acyl-CoA dehydrogenase [EC:1.3.8.7] | (RefSeq) probable acyl-CoA dehydrogenase IBR3 (A)	Probable acyl-CoA dehydrogenase IBR3 {ECO:0000305}; EC=1.3.99.- {ECO:0000305}; Protein INDOLE-3-BUTYRIC ACID RESPONSE 3 {ECO:0000303|PubMed:17277896} (At3g06810)		1.524
Cs9g05210	K07513 acetyl-CoA acyltransferase 1 [EC:2.3.1.16] | (RefSeq) hypothetical protein (A)	3-ketoacyl-CoA thiolase 2, peroxisomal; EC=2.3.1.16; Acetyl-CoA acyltransferase 2; Beta-ketothiolase 2; Peroxisomal 3-oxoacyl-CoA thiolase 2; Peroxisome defective protein 1; Flags: Precursor (At2g33150)	-1.826	
Cs9g06650	K15718 linoleate 9S-lipoxygenase [EC:1.13.11.58] | (RefSeq) hypothetical protein (A)	Linoleate 9S-lipoxygenase 5; EC=1.13.11.58; Lipoxygenase 5; AtLOX5 (At3g22400)		-1.358
Cs9g16620	K21270 (R)-mandelonitrile oxidase [EC:1.1.3.49] | (RefSeq) protein HOTHEAD (A)	Protein HOTHEAD; Protein ADHESION OF CALYX EDGES; Flags: Precursor (At5g51950)	1.597	4.643
novel.431	K10529 alpha-dioxygenase [EC:1.-.-.-] | (RefSeq) alpha-dioxygenase 1-like (A)	DOX1_ARATH Alpha-dioxygenase 1; Alpha DOX1; EC=1.14.99.-; Fatty acid dioxygenase AlphaDOX1; Pathogen-induced oxygenase; Plant alpha		-2.296

		dioxygenase 1; Flags: Precursor (At3g01420)		
Phospholipid catabolic process [GO:0009395; P = 0.1601 (P3CR vs. P3R) and 0.1082 (P5CR vs. P5R)]				
Cs1g18410	K01115 phospholipase D1/2 [EC:3.1.4.4] | (RefSeq) phospholipase D alpha 4 (A)	Phospholipase D alpha 4 {ECO:0000303|PubMed:11891260}; AtPLDalpha4 {ECO:0000303|PubMed:11891260}; PLD alpha 4 {ECO:0000303|PubMed:11891260}; EC=3.1.4.4 {ECO:0000250|UniProtKB:Q38882}; PLDalpha3; Phospholipase D epsilon {ECO:0000303|PubMed:19143999}; AtPLDepsilon {ECO:0000303|PubMed:19143999}; PLD epsilon {ECO:0000303|PubMed:19143999} (At1g55180)	1.605	2.372
Cs2g29630	K01114 phospholipase C [EC:3.1.4.3] | (RefSeq) non-specific phospholipase C2 (A)	Non-specific phospholipase C2; EC=3.1.-.-; Flags: Precursor (At2g26870)		-1.356
Cs7g15620	K01114 phospholipase C [EC:3.1.4.3] | (RefSeq) non-specific phospholipase C4-like (A)	Non-specific phospholipase C4; EC=3.1.4.3 (At3g03530)	1.786	2.198
