# Supplementary material for: Mechanisms by Which Increased pH Ameliorates Copper Excess in Citrus sinensis Roots: Insight from a Combined Analysis of Physiology, Transcriptome, and Metabolome
Source: Plants (Basel). 2024 Oct 31;13(21):3054. doi: 10.3390/plants13213054 (PMC11548300; doi:10.3390/plants13213054)
Supplement: Supplementary file 1 [file plants-13-03054-s001.zip › Table S18SecondRevision.pdf]

Table S18. DEGs related to nucleotide metabolism in P3CR vs. P3R and/or P5CR vs. P5R

**Accession No.**	**KEGG**	**Swissprot (KOG)**	**Log$_2$(FC)**	
			P3CR vs. P3R	P5CR vs. P5R
Nucleotide biosynthetic process [GO:0009165; P = 0.3078 (P3CR vs. P3R) and 0.9844 (P5CR vs. P5R)]				
Cs1g13900	K04733 interleukin-1 receptor-associated kinase 4 [EC:2.7.11.1] | (RefSeq) wall-associated receptor kinase-like 10 isoform X1 (A)	Wall-associated receptor kinase-like 10; EC=2.7.11.-; Flags: Precursor (At1g79680)	1.844	
Cs1g15840	K00844 hexokinase [EC:2.7.1.1] | (RefSeq) hexokinase-1-like (A)	Hexokinase-1; EC=2.7.1.1; NtHxK1 (At4g29130)		-1.611
Cs1g15970	K00850 6-phosphofructokinase 1 [EC:2.7.1.11] | (RefSeq) ATP-dependent 6-phosphofructokinase 3-like (A)	ATP-dependent 6-phosphofructokinase 3 {ECO:0000255|HAMAP-Rule:MF_03186}; ATP-PFK 3 {ECO:0000255|HAMAP-Rule:MF_03186}; Phosphofructokinase 3 {ECO:0000255|HAMAP-Rule:MF_03186}; EC=2.7.1.11 {ECO:0000255|HAMAP-Rule:MF_03186}; Phosphohexokinase 3 {ECO:0000255|HAMAP-Rule:MF_03186} (At4g29220)	1.064	
Cs1g16160	K01535 H+-transporting ATPase [EC:3.6.3.6] | (RefSeq) hypothetical protein (A)	ATPase 10, plasma membrane-type; EC=3.6.3.6; Proton pump 10 (At1g17260)		2.076
Cs3g18630	K11262 acetyl-CoA carboxylase / biotin carboxylase 1 [EC:6.4.1.2 6.3.4.14 2.1.3.15] | (RefSeq) acetyl-CoA carboxylase 1-like (A)	Acetyl-CoA carboxylase 1; AtACC1; EC=6.4.1.2 {ECO:0000269|PubMed:9008389}; Protein EMBRYO DEFECTIVE 22; Protein GURKE; Protein PASTICCINO 3; Includes: Biotin carboxylase; EC=6.3.4.14 (At1g36160)		1.119
Cs4g13070	K00850 6-phosphofructokinase 1 [EC:2.7.1.11] | (RefSeq) ATP-dependent 6-phosphofructokinase 3-like (A)	ATP-dependent 6-phosphofructokinase 3 {ECO:0000255|HAMAP-Rule:MF_03186}; ATP-PFK 3 {ECO:0000255|HAMAP-Rule:MF_03186}; Phosphofructokinase 3 {ECO:0000255|HAMAP-Rule:MF_03186}; EC=2.7.1.11 {ECO:0000255|HAMAP-Rule:MF_03186}; Phosphohexokinase 3 {ECO:0000255|HAMAP-Rule:MF_03186} (At5g56630)	-1.258	
Cs4g18980	K00850 6-phosphofructokinase 1 [EC:2.7.1.11] | (RefSeq) hypothetical protein (A)	ATP-dependent 6-phosphofructokinase 3 {ECO:0000255|HAMAP-Rule:MF_03186}; ATP-PFK 3 {ECO:0000255|HAMAP-Rule:MF_03186}; Phosphofructokinase 3 {ECO:0000255|HAMAP-Rule:MF_03186}; EC=2.7.1.11	-1.602	-1.466

		{ECO:0000255|HAMAP-Rule:MF_03186}; Phosphohexokinase 3 {ECO:0000255|HAMAP-Rule:MF_03186} (At4g32840)		
Cs5g08370	K01535 H+-transporting ATPase [EC:3.6.3.6] | (RefSeq) hypothetical protein (A)	ATPase 11, plasma membrane-type; EC=3.6.3.6; Proton pump 11 (At5g62670)		-3.477
Cs6g03430	K01535 H+-transporting ATPase [EC:3.6.3.6] | (RefSeq) plasma membrane ATPase 1-like (A)	ATPase 7, plasma membrane-type; EC=3.6.3.6; Proton pump 7 (At3g60330)	2.160	
Cs6g03440	K01535 H+-transporting ATPase [EC:3.6.3.6] | (RefSeq) hypothetical protein (A)	Plasma membrane ATPase 1; EC=3.6.3.6; Proton pump 1 (At5g62670)	2.437	
Cs6g03445	K01535 H+-transporting ATPase [EC:3.6.3.6] | (RefSeq) hypothetical protein (A)	ATPase 4, plasma membrane-type; EC=3.6.3.6; Proton pump 4 (At1g80660)	2.470	
Cs6g03480	K01535 H+-transporting ATPase [EC:3.6.3.6] | (RefSeq) plasma membrane ATPase 1-like (A)	Plasma membrane ATPase 1; EC=3.6.3.6; Proton pump 1 (At5g62670)	2.445	
Cs6g03490	K01535 H+-transporting ATPase [EC:3.6.3.6] | (RefSeq) hypothetical protein (A)	ATPase 4, plasma membrane-type; EC=3.6.3.6; Proton pump 4 (At5g62670)	2.662	
Cs6g19330	K13800 UMP-CMP kinase [EC:2.7.4.14] | (RefSeq) UMP-CMP kinase 3-like (A)	UMP-CMP kinase 3 {ECO:0000255|HAMAP-Rule:MF_03172}; EC=2.7.4.14 {ECO:0000255|HAMAP-Rule:MF_03172}; Deoxycytidylate kinase {ECO:0000255|HAMAP-Rule:MF_03172}; CK {ECO:0000255|HAMAP-Rule:MF_03172}; dCMP kinase {ECO:0000255|HAMAP-Rule:MF_03172}; Uridine monophosphate/cytidine monophosphate kinase {ECO:0000255|HAMAP-Rule:MF_03172}; UMP/CMP kinase {ECO:0000255|HAMAP-Rule:MF_03172}; UMP/CMPK {ECO:0000255|HAMAP-Rule:MF_03172} (At3g60180)		-1.950
Cs6g20570	K01535 H+-transporting ATPase [EC:3.6.3.6] | (RefSeq) plasma membrane ATPase 1-like (A)	Plasma membrane ATPase 1; EC=3.6.3.6; Proton pump 1 (At3g47950)	1.223	
Cs6g20790	K09285 AP2-like factor, ANT lineage | (RefSeq) AP2-like ethylene-responsive transcription factor At1g16060 (A)	Ethylene-responsive transcription factor WRI1; Protein ACTIVATOR OF SPORAMIN::LUC 1; Protein WRINKLED 1 (At1g16060)	1.439	-2.381
Cs7g04300	K09285 AP2-like factor, ANT lineage | (RefSeq) AP2-like ethylene-responsive transcription factor At1g79700 (A)	Ethylene-responsive transcription factor WRI1; Protein ACTIVATOR OF SPORAMIN::LUC 1; Protein WRINKLED 1 (At5g17430)		-5.678
Cs7g12380	K00876 uridine kinase [EC:2.7.1.48] | (RefSeq) uridine-cytidine kinase C (A)	Uridine kinase-like protein 1, chloroplastic; Includes: Uridine kinase; UK; EC=2.7.1.48; Includes: Putative uracil phosphoribosyltransferase; UPRTase; EC=2.4.2.9; UMP pyrophosphorylase; Flags: Precursor (At1g26190)		1.837
Cs7g17090	K13508 glycerol-3-phosphate acyltransferase [EC:2.3.1.15	Glycerol-3-phosphate acyltransferase 1; AtGPAT1; EC=2.3.1.15		1.529

	2.3.1.198] | (RefSeq) glycerol-3-phosphate acyltransferase 1 (A)	(At1g06520)		
Cs7g31730	K13508 glycerol-3-phosphate acyltransferase [EC:2.3.1.15 2.3.1.198] | (RefSeq) hypothetical protein (A)	Glycerol-3-phosphate acyltransferase 7; AtGPAT7; EC=2.3.1.15 (At5g06090)	1.020	4.530
Cs8g06110	K00940 nucleoside-diphosphate kinase [EC:2.7.4.6] | (RefSeq) nucleoside diphosphate kinase 1-like (A)	Nucleoside diphosphate kinase 1; EC=2.7.4.6; Nucleoside diphosphate kinase I; NDK I; NDP kinase I; NDPK I (At4g09320)	-1.926	-1.763
Cs8g06970	K13513 lysocardiolipin and lysophospholipid acyltransferase [EC:2.3.1.- 2.3.1.51] | (RefSeq) probable 1-acyl-sn-glycerol-3-phosphate acyltransferase 5 (A)	Probable 1-acyl-sn-glycerol-3-phosphate acyltransferase 5; EC=2.3.1.51; Lysophosphatidyl acyltransferase 5 (At3g18850)	-1.227	
Cs8g07730	K00764 amidophosphoribosyltransferase [EC:2.4.2.14] | (RefSeq) amidophosphoribosyltransferase, chloroplastic-like (A)	Amidophosphoribosyltransferase 2, chloroplastic; AtATase2; AtPURF2; PRPP2; EC=2.4.2.14; Glutamine phosphoribosylpyrophosphate amidotransferase 2; AtGPRAT2; Protein CHLOROPLAST IMPORT APPARATUS 1; Protein DIFFERENTIAL DEVELOPMENT OF VASCULAR ASSOCIATED CELLS; Flags: Precursor (At4g34740)	1.316	
Cs8g08710	K01623 fructose-bisphosphate aldolase, class I [EC:4.1.2.13] | (RefSeq) fructose-bisphosphate aldolase 1, chloroplastic (A)	Fructose-bisphosphate aldolase 2, chloroplastic {ECO:0000305}; AtFBA2 {ECO:0000303|PubMed:22561114}; EC=4.1.2.13 {ECO:0000305}; Flags: Precursor (At2g01140)	1.927	
Cs9g18120	K00873 pyruvate kinase [EC:2.7.1.40] | (RefSeq) hypothetical protein (A)	Plastidial pyruvate kinase 4, chloroplastic; PKp4; EC=2.7.1.40; Flags: Precursor (At3g49160)	-1.526	-1.586
novel.1672	K00764 amidophosphoribosyltransferase [EC:2.4.2.14] | (RefSeq) hypothetical protein (A)	ASE1_ARATH Amidophosphoribosyltransferase 1, chloroplastic; AtATase1; PRPP1; EC=2.4.2.14; Glutamine phosphoribosylpyrophosphate amidotransferase 1; AtGPRAT1; Flags: Precursor (At2g16570)		1.033
novel.2787	K09680 type II pantothenate kinase [EC:2.7.1.33] | (RefSeq) hypothetical protein (A)	PANK2_ARATH Pantothenate kinase 2; AtPANK2; EC=2.7.1.33; Pantothenic acid kinase 2 (At4g32180_1)		1.437
orange1.1t00198	K00981 phosphatidate cytidylyltransferase [EC:2.7.7.41] | (RefSeq) hypothetical protein (A)	Phosphatidate cytidylyltransferase 4, chloroplastic {ECO:0000303|PubMed:20442275}; EC=2.7.7.41 {ECO:0000269|PubMed:20442275}; CDP-DAG synthase 4; CDP-DG synthase 4; CDP-diacylglycerol synthase 4; CDS4; CDP-diglyceride pyrophosphorylase 4; CDP-diglyceride synthase 4; CTP:phosphatidate cytidylyltransferase 4; Flags: Precursor (At2g45150)		1.024
orange1.1t00375	K13508 glycerol-3-phosphate acyltransferase [EC:2.3.1.15 2.3.1.198] | (RefSeq) probable glycerol-3-phosphate acyltransferase 8 (A)	Probable glycerol-3-phosphate acyltransferase 8; EC=2.3.1.15 (At1g01610)	1.009	3.308

orange1.1t04332		Nicotinamidase 1 {ECO:0000305}; AtNIC1 {ECO:0000303|PubMed:17335512}; EC=3.5.1.19 {ECO:0000269|PubMed:17335512}; Nicotinamide deamidase 1 (At2g22570)	1.020	
orange1.1t04553	K01956 carbamoyl-phosphate synthase small subunit [EC:6.3.5.5] | (RefSeq) hypothetical protein (A)	Carbamoyl-phosphate synthase small chain, chloroplastic; EC=6.3.5.5; Carbamoyl-phosphate synthetase glutamine chain; Protein VENOSA 6; Flags: Precursor (At3g27740)		-1.087
Nucleotide catabolic process [GO:0009166; P = 0.2660 (P3CR vs. P3R) and 0.8605 (P5CR vs. P5R)]				
Cs1g15840	K00844 hexokinase [EC:2.7.1.1] | (RefSeq) hexokinase-1-like (A)	Hexokinase-1; EC=2.7.1.1; NtHxK1 (At4g29130)		-1.611
Cs1g15970	K00850 6-phosphofructokinase 1 [EC:2.7.1.11] | (RefSeq) ATP-dependent 6-phosphofructokinase 3-like (A)	ATP-dependent 6-phosphofructokinase 3 {ECO:0000255|HAMAP-Rule:MF_03186}; ATP-PFK 3 {ECO:0000255|HAMAP-Rule:MF_03186}; Phosphofructokinase 3 {ECO:0000255|HAMAP-Rule:MF_03186}; EC=2.7.1.11 {ECO:0000255|HAMAP-Rule:MF_03186}; Phosphohexokinase 3 {ECO:0000255|HAMAP-Rule:MF_03186} (At4g29220)	1.064	
Cs4g13070	K00850 6-phosphofructokinase 1 [EC:2.7.1.11] | (RefSeq) ATP-dependent 6-phosphofructokinase 3-like (A)	ATP-dependent 6-phosphofructokinase 3 {ECO:0000255|HAMAP-Rule:MF_03186}; ATP-PFK 3 {ECO:0000255|HAMAP-Rule:MF_03186}; Phosphofructokinase 3 {ECO:0000255|HAMAP-Rule:MF_03186}; EC=2.7.1.11 {ECO:0000255|HAMAP-Rule:MF_03186}; Phosphohexokinase 3 {ECO:0000255|HAMAP-Rule:MF_03186} (At5g56630)	-1.258	
Cs4g18980	K00850 6-phosphofructokinase 1 [EC:2.7.1.11] | (RefSeq) hypothetical protein (A)	ATP-dependent 6-phosphofructokinase 3 {ECO:0000255|HAMAP-Rule:MF_03186}; ATP-PFK 3 {ECO:0000255|HAMAP-Rule:MF_03186}; Phosphofructokinase 3 {ECO:0000255|HAMAP-Rule:MF_03186}; EC=2.7.1.11 {ECO:0000255|HAMAP-Rule:MF_03186}; Phosphohexokinase 3 {ECO:0000255|HAMAP-Rule:MF_03186} (At4g32840)	-1.602	-1.466
Cs6g20790	K09285 AP2-like factor, ANT lineage | (RefSeq) AP2-like ethylene-responsive transcription factor At1g16060 (A)	Ethylene-responsive transcription factor WRI1; Protein ACTIVATOR OF SPORAMIN::LUC 1; Protein WRINKLED 1 (At1g16060)	1.439	-2.381
Cs7g04300	K09285 AP2-like factor, ANT lineage | (RefSeq) AP2-like ethylene-responsive transcription factor At1g79700 (A)	Ethylene-responsive transcription factor WRI1; Protein ACTIVATOR OF SPORAMIN::LUC 1; Protein WRINKLED 1 (At5g17430)		-5.678
Cs8g08710	K01623 fructose-bisphosphate aldolase, class I [EC:4.1.2.13] |	Fructose-bisphosphate aldolase 2, chloroplastic {ECO:0000305};	1.927	

	(RefSeq) fructose-bisphosphate aldolase 1, chloroplastic (A)	AtFBA2 {ECO:0000303|PubMed:22561114}; EC=4.1.2.13 {ECO:0000305}; Flags: Precursor (At2g01140)		
Cs9g18120	K00873 pyruvate kinase [EC:2.7.1.40] | (RefSeq) hypothetical protein (A)	Plastidial pyruvate kinase 4, chloroplastic; PKp4; EC=2.7.1.40; Flags: Precursor (At3g49160)	-1.526	-1.586
