# Supplementary material for: Mechanisms by Which Increased pH Ameliorates Copper Excess in Citrus sinensis Roots: Insight from a Combined Analysis of Physiology, Transcriptome, and Metabolome
Source: Plants (Basel). 2024 Oct 31;13(21):3054. doi: 10.3390/plants13213054 (PMC11548300; doi:10.3390/plants13213054)
Supplement: Supplementary file 1 [file plants-13-03054-s001.zip › Table S19SecondRevision.pdf]

Table S19. DEGs and DAMs related to biosynthesis and catabolism of SMs in P3CR vs. P3R and/or P5CR vs. P5R

**Accession No.**	**KEGG**	**Swissprot (KOG)**	**Log_2(FC)**	
			P3CR vs. P3R	P5CR vs. P5R
Biosynthesis of secondary metabolites [ko01110; P < 0.0001 (P3CR vs. P3R) and 0.0001 (P5CR vs. P5R)]				
Cs1g01110	K01593 aromatic-L-amino-acid/L-tryptophan decarboxylase [EC:4.1.1.28 4.1.1.105] | (RefSeq) tyrosine decarboxylase 1-like (A)	Tyrosine/DOPA decarboxylase 3; Includes: DOPA decarboxylase; DDC; EC=4.1.1.28; Includes: Tyrosine decarboxylase; EC=4.1.1.25 (At2g20340)		-1.195
Cs1g01290	K00232 acyl-CoA oxidase [EC:1.3.3.6] | (RefSeq) hypothetical protein (A)	Acyl-coenzyme A oxidase 2, peroxisomal; AOX 2; EC=1.3.3.6; Long-chain acyl-CoA oxidase; AtCX2; Flags: Precursor (At5g65110)		1.830
Cs1g01330	K01087 trehalose 6-phosphate phosphatase [EC:3.1.3.12] | (RefSeq) probable trehalose-phosphate phosphatase J (A)	Probable trehalose-phosphate phosphatase J; AtTPPJ; EC=3.1.3.12; Trehalose 6-phosphate phosphatase (At5g65140)	-1.651	-1.272
Cs1g02760	K15404 aldehyde decarbonylase [EC:4.1.99.5] | (RefSeq) protein ECERIFERUM 1 (A)	Protein ECERIFERUM 1 (At1g02190)	-1.004	
Cs1g03730	K12644 acridone synthase [EC:2.3.1.159] | (RefSeq) hypothetical protein (A)	Acridone synthase 2; EC=2.3.1.159; Acridone synthase II (At5g13930)	5.866	
Cs1g03780	K13066 caffeic acid 3-O-methyltransferase [EC:2.1.1.68] | (RefSeq) caffeic acid 3-O-methyltransferase-like isoform X1 (A)	Caffeic acid 3-O-methyltransferase; CAOMT; COMT; EC=2.1.1.68; S-adenosysl-L-methionine:caffeic acid 3-O-methyltransferase (At5g54160)		1.270
Cs1g03840	K22395 cinnamyl-alcohol dehydrogenase [EC:1.1.1.195] | (RefSeq) tetrahydrocannabinolic acid synthase-like isoform X1 (A)	Tetrahydrocannabinolic acid synthase; THCAS; EC=1.21.3.7; Delta(1)-tetrahydrocannabinolic acid synthase; THCA synthase; Flags: Precursor (At5g44440)		-1.588
Cs1g03950	K14975 codeine 3-O-demethylase [EC:1.14.11.32] | (RefSeq) codeine O-demethylase (A)	Codeine O-demethylase; EC=1.14.11.32 (At5g20400)		1.515
Cs1g04260	K13065 shikimate O-hydroxycinnamoyltransferase [EC:2.3.1.133] | (RefSeq) vinorine synthase-like (A)	Salutaridinol 7-O-acetyltransferase {ECO:0000303|PubMed:11404355}; salAT {ECO:0000303|PubMed:11404355}; EC=2.3.1.150 {ECO:0000269|PubMed:11404355, ECO:0000269|PubMed:8537369} (At3g26040)		2.369
Cs1g04290	K13065 shikimate O-hydroxycinnamoyltransferase [EC:2.3.1.133] | (RefSeq) POPTRDRAFT_818939; hypothetical protein (A)	Salutaridinol 7-O-acetyltransferase {ECO:0000303|PubMed:11404355}; salAT		1.260

		{ECO:0000303|PubMed:11404355}; EC=2.3.1.150 {ECO:0000269|PubMed:11404355, ECO:0000269|PubMed:8537369} (At3g26040)		
Cs1g04300	K13065 shikimate O-hydroxycinnamoyltransferase [EC:2.3.1.133] | (RefSeq) POPTRDRAFT_818939; hypothetical protein (A)	Salutaridinol 7-O-acetyltransferase {ECO:0000303|PubMed:11404355}; salAT {ECO:0000303|PubMed:11404355}; EC=2.3.1.150 {ECO:0000269|PubMed:11404355, ECO:0000269|PubMed:8537369} (At3g26040)		1.149
Cs1g04900	K09838 zeaxanthin epoxidase [EC:1.14.15.21] | (RefSeq) zeaxanthin epoxidase, chloroplastic-like (A)	Monooxygenase 2 {ECO:0000303|PubMed:10216258}; AtMO2 {ECO:0000303|PubMed:10216258}; EC=1.14.13.- (At4g38540)		-1.032
Cs1g04910	K00083 cinnamyl-alcohol dehydrogenase [EC:1.1.1.195] | (RefSeq) hypothetical protein (A)	Probable cinnamyl alcohol dehydrogenase 9; AtCAD9; EC=1.1.1.195 (At4g39330)	-2.173	
Cs1g05810	K13065 shikimate O-hydroxycinnamoyltransferase [EC:2.3.1.133] | (RefSeq) shikimate O-hydroxycinnamoyltransferase-like (A)	Shikimate O-hydroxycinnamoyltransferase; EC=2.3.1.133; Hydroxycinnamoyl transferase; Hydroxycinnamoyl-Coenzyme A shikimate/quinate hydroxycinnamoyltransferase (At5g48930)		-1.040
Cs1g05840	K13065 shikimate O-hydroxycinnamoyltransferase [EC:2.3.1.133] | (RefSeq) vinorine synthase-like (A)	Salutaridinol 7-O-acetyltransferase {ECO:0000303|PubMed:11404355}; salAT {ECO:0000303|PubMed:11404355}; EC=2.3.1.150 {ECO:0000269|PubMed:11404355, ECO:0000269|PubMed:8537369} (At3g26040)		1.514
Cs1g05930	K13065 shikimate O-hydroxycinnamoyltransferase [EC:2.3.1.133] | (RefSeq) vinorine synthase-like (A)	Salutaridinol 7-O-acetyltransferase {ECO:0000303|PubMed:11404355}; salAT {ECO:0000303|PubMed:11404355}; EC=2.3.1.150 {ECO:0000269|PubMed:11404355, ECO:0000269|PubMed:8537369} (At3g26040)		-1.445
Cs1g06680	K03183 demethylmenaquinone methyltransferase / 2-methoxy-6-polyprenyl-1,4-benzoquinol methylase [EC:2.1.1.163 2.1.1.201] | (RefSeq) 2-phytyl-1,4-beta-naphthoquinone methyltransferase, chloroplastic-like (A)	2-phytyl-1,4-beta-naphthoquinone methyltransferase, chloroplastic {ECO:0000255|HAMAP-Rule:MF_03192}; EC=2.1.1.329 {ECO:0000255|HAMAP-Rule:MF_03192}; Demethylphylloquinone methyltransferase {ECO:0000255|HAMAP-Rule:MF_03192}; Menaquinone biosynthesis methyltransferase ubiE-like protein {ECO:0000255|HAMAP-Rule:MF_03192}; Flags: Precursor (At5g54400)	-1.020	
Cs1g06790	K05350 beta-glucosidase [EC:3.2.1.21] | (RefSeq) beta-glucosidase 18-like (A)	Beta-glucosidase 18; Os4bglu18; EC=3.2.1.21; Flags: Precursor (At4g21760)		1.146

Cs1g08010	K00815 tyrosine aminotransferase [EC:2.6.1.5] | (RefSeq) hypothetical protein (A)	Probable aminotransferase TAT2; EC=2.6.1.-; Tyrosine aminotransferase 2 (At5g53970)	1.218	
Cs1g09020	K13065 shikimate O-hydroxycinnamoyltransferase [EC:2.3.1.133] | (RefSeq) vinorine synthase-like (A)	Salutaridinol 7-O-acetyltransferase {ECO:0000303|PubMed:11404355}; salAT {ECO:0000303|PubMed:11404355}; EC=2.3.1.150 {ECO:0000269|PubMed:11404355, ECO:0000269|PubMed:8537369} (At3g26040)		1.142
Cs1g11470	K08101 phytochromobilin:ferredoxin oxidoreductase [EC:1.3.7.4] | (RefSeq) phytochromobilin:ferredoxin oxidoreductase, chloroplastic-like isoform X1 (A)	(At1g71110)		-6.472
Cs1g11480	K01193 beta-fructofuranosidase [EC:3.2.1.26] | (RefSeq) LOC109747879; beta-fructofuranosidase, insoluble isoenzyme 3-like (A)	Putative UPF0481 protein At3g02645 (At3g02650_1)	2.078	
Cs1g12000	K09833 homogentisate phytyltransferase / homogentisate geranylgeranyltransferase [EC:2.5.1.115 2.5.1.116] | (RefSeq) homogentisate phytyltransferase 1, chloroplastic-like (A)	Coumarin 8-geranyltransferase 1b, chloroplastic; EC=2.5.1.138 {ECO:0000269|PubMed:25077796}; Prenyltransferase 1b {ECO:0000303|PubMed:25077796}; ClPT1ba {ECO:0000303|PubMed:25077796}; Flags: Precursor (At2g18950)		-8.298
Cs1g12860	K13496 UDP-glucosyl transferase 73C [EC:2.4.1.-] | (RefSeq) hypothetical protein (A)	UDP-glycosyltransferase 73C4; EC=2.4.1.- (At2g36770)	-1.144	
Cs1g13740	K04124 gibberellin 3-beta-dioxygenase [EC:1.14.11.15] | (RefSeq) gibberellin 3-beta-dioxygenase 1 (A)	Probable 2-oxoglutarate-dependent dioxygenase AOP1.2; EC=1.14.11.- (At1g52790)	1.835	1.472
Cs1g13750	K04124 gibberellin 3-beta-dioxygenase [EC:1.14.11.15] | (RefSeq) gibberellin 3-beta-dioxygenase 4 (A)	Probable 2-oxoglutarate-dependent dioxygenase AOP1.2; EC=1.14.11.- (At1g52800)	1.445	
Cs1g13770	K05282 gibberellin 20-oxidase [EC:1.14.11.12] | (RefSeq) gibberellin 20 oxidase 4 (A)	Probable 2-oxoglutarate-dependent dioxygenase AOP1.2; EC=1.14.11.- (At1g52800)	1.607	1.597
Cs1g13820	K04124 gibberellin 3-beta-dioxygenase [EC:1.14.11.15] | (RefSeq) gibberellin 3-beta-dioxygenase 3 (A)	Probable 2-oxoglutarate-dependent dioxygenase AOP1.2; EC=1.14.11.- (At1g52820)	2.198	2.712
Cs1g15840	K00844 hexokinase [EC:2.7.1.1] | (RefSeq) hexokinase-1-like (A)	Hexokinase-1; EC=2.7.1.1; NtHxK1 (At4g29130)		-1.611
Cs1g15970	K00850 6-phosphofructokinase 1 [EC:2.7.1.11] | (RefSeq) ATP-dependent 6-phosphofructokinase 3-like (A)	ATP-dependent 6-phosphofructokinase 3 {ECO:0000255|HAMAP-Rule:MF_03186}; ATP-PFK 3 {ECO:0000255|HAMAP-Rule:MF_03186}; Phosphofructokinase 3 {ECO:0000255|HAMAP-Rule:MF_03186}; EC=2.7.1.11 {ECO:0000255|HAMAP-Rule:MF_03186}; Phosphohexokinase 3 {ECO:0000255|HAMAP-Rule:MF_03186} (At4g29220)	1.064	

Cs1g17380	K00454 lipoxygenase [EC:1.13.11.12] | (RefSeq) linoleate 13S-lipoxygenase 3-1, chloroplastic-like (A)	Linoleate 13S-lipoxygenase 3-1, chloroplastic; EC=1.13.11.12; Flags: Precursor (At1g17420)	1.679	2.275
Cs1g17390	K20623 typhasterol/6-deoxotyphasterol 2alpha-hydroxylase | (RefSeq) cytochrome P450 71A1-like (A)	Cytochrome P450 71A1; EC=1.14.-.-; ARP-2; CYPLXXIA1 (At5g07990)	1.668	1.117
Cs1g17410	K20623 typhasterol/6-deoxotyphasterol 2alpha-hydroxylase | (RefSeq) hypothetical protein (A)	Cytochrome P450 71A1; EC=1.14.-.-; ARP-2; CYPLXXIA1 (At5g07990)	4.742	4.120
Cs1g18220	K01193 beta-fructofuranosidase [EC:3.2.1.26] | (RefSeq) beta-fructofuranosidase, insoluble isoenzyme CWINV1 (A)	Beta-fructofuranosidase, insoluble isoenzyme CWINV1; EC=3.2.1.26; Cell wall beta-fructosidase 1; AtbetaFRUCT1; Cell wall invertase 1; AtcwINV1; Sucrose hydrolase 1; Flags: Precursor (At3g13790)		-1.274
Cs1g18240	K01193 beta-fructofuranosidase [EC:3.2.1.26] | (RefSeq) beta-fructofuranosidase, insoluble isoenzyme CWINV1-like (A)	Fructan 6-exohydrolase {ECO:0000312|EMBL:CAD48404.1}; EC=3.2.1.154; Flags: Precursor (At3g13790)	1.373	
Cs1g18410	K01115 phospholipase D1/2 [EC:3.1.4.4] | (RefSeq) phospholipase D alpha 4 (A)	Phospholipase D alpha 4 {ECO:0000303|PubMed:11891260}; AtPLDalpha4 {ECO:0000303|PubMed:11891260}; PLD alpha 4 {ECO:0000303|PubMed:11891260}; EC=3.1.4.4 {ECO:0000250|UniProtKB:Q38882}; PLDalpha3; Phospholipase D epsilon {ECO:0000303|PubMed:19143999}; AtPLDepsilon {ECO:0000303|PubMed:19143999}; PLD epsilon {ECO:0000303|PubMed:19143999} (At1g55180)	1.605	2.372
Cs1g19360	K01772 protoporphyrin/coproporphyrin ferrochelatase [EC:4.99.1.1 4.99.1.9] | (RefSeq) ferrochelatase-2, chloroplastic (A)	Ferrochelatase-2, chloroplastic; EC=4.99.1.1; Ferrochelatase II; Heme synthase 2; Protoheme ferro-lyase 2; Flags: Precursor (At2g30390)		1.121
Cs1g19730	K21374 7-deoxyloganetin glucosyltransferase [EC:2.4.1.324] | (RefSeq) 7-deoxyloganetin glucosyltransferase-like (A)	Linamarin synthase 2 {ECO:0000305}; EC=2.4.1.63 {ECO:0000269|PubMed:21736650}; Cyanohydrin UDP-glucosyltransferase UGT85K5 {ECO:0000303|PubMed:21736650} (At1g22360)	1.022	
Cs1g19740	K21374 7-deoxyloganetin glucosyltransferase [EC:2.4.1.324] | (RefSeq) 7-deoxyloganetin glucosyltransferase-like (A)	Linamarin synthase 2 {ECO:0000305}; EC=2.4.1.63 {ECO:0000269|PubMed:21736650}; Cyanohydrin UDP-glucosyltransferase UGT85K5 {ECO:0000303|PubMed:21736650} (At1g22360)	1.151	
Cs1g19770	K21374 7-deoxyloganetin glucosyltransferase [EC:2.4.1.324] | (RefSeq) 7-deoxyloganetin glucosyltransferase-like (A)	Linamarin synthase 1 {ECO:0000305}; EC=2.4.1.63 {ECO:0000269|PubMed:21736650}; Cyanohydrin UDP-glucosyltransferase UGT85K4 {ECO:0000303|PubMed:21736650} (At1g22360)	1.266	

Cs1g20370	K10703 very-long-chain (3R)-3-hydroxyacyl-CoA dehydratase [EC:4.2.1.134] | (RefSeq) very-long-chain (3R)-3-hydroxyacyl-CoA dehydratase PASTICCINO 2 (A)	Very-long-chain (3R)-3-hydroxyacyl-CoA dehydratase PASTICCINO 2 {ECO:0000305}; EC=4.2.1.134 {ECO:0000305|PubMed:18799749}; 3-hydroxyacyl-CoA dehydratase PASTICCINO 2; AtPAS2; HACD; HCD; Protein PEPINO; PEP; Protein tyrosine phosphatase-like protein (At5g10480)		1.075
Cs1g20580	K00083 cinnamyl-alcohol dehydrogenase [EC:1.1.1.195] | (RefSeq) probable mannitol dehydrogenase (A)	Probable mannitol dehydrogenase; EC=1.1.1.255; NAD-dependent mannitol dehydrogenase (At4g37990)		-1.691
Cs1g20610	K00083 cinnamyl-alcohol dehydrogenase [EC:1.1.1.195] | (RefSeq) probable mannitol dehydrogenase (A)	Probable mannitol dehydrogenase; EC=1.1.1.255; NAD-dependent mannitol dehydrogenase (At4g37990)	1.280	
Cs1g20920	K01610 phosphoenolpyruvate carboxykinase (ATP) [EC:4.1.1.49] | (RefSeq) phosphoenolpyruvate carboxykinase [ATP]-like (A)	Phosphoenolpyruvate carboxykinase (ATP); PEP carboxykinase; PEPCK; EC=4.1.1.49 (At4g37870)		1.085
Cs1g22210	K13260 isoflavone/4'-methoxyisoflavone 2'-hydroxylase [EC:1.14.13.89 1.14.13.53] | (RefSeq) cytochrome P450 81E8-like (A)	Cytochrome P450 81E8 {ECO:0000303|PubMed:14617078}; EC=1.14.13.- {ECO:0000305} (At4g37370)		-1.971
Cs1g22230	K13260 isoflavone/4'-methoxyisoflavone 2'-hydroxylase [EC:1.14.13.89 1.14.13.53] | (RefSeq) cytochrome P450 81E8-like (A)	Cytochrome P450 81E8 {ECO:0000303|PubMed:14617078}; EC=1.14.13.- {ECO:0000305} (At4g37370)		-1.966
Cs1g22680	K13545 red chlorophyll catabolite reductase [EC:1.3.7.12] | (RefSeq) red chlorophyll catabolite reductase-like (A)	Red chlorophyll catabolite reductase, chloroplastic; AtRCCR; RCC reductase; EC=1.3.7.12 {ECO:0000269|PubMed:10743659}; Accelerated cell death protein 2; Flags: Precursor (At4g37000)	1.740	
Cs1g22960	K00430 peroxidase [EC:1.11.1.7] | (RefSeq) peroxidase 10-like (A)	Peroxidase 10; Atperox P10; EC=1.11.1.7; ATP5a; Flags: Precursor (At1g49570)		1.690
Cs1g23150	K20802 (R)-mandelonitrile lyase [EC:4.1.2.10] | (RefSeq) alpha-hydroxynitrile lyase (A)	Methylesterase 10; AtMES10; EC=3.1.1.- (At3g50440)		-1.319
Cs1g23190	K20802 (R)-mandelonitrile lyase [EC:4.1.2.10] | (RefSeq) alpha-hydroxynitrile lyase (A)	Salicylic acid-binding protein 2; NtSABP2; EC=3.1.1.-; Methyl salicylate esterase (At2g23610)		-1.562
Cs1g23200	K20802 (R)-mandelonitrile lyase [EC:4.1.2.10] | (RefSeq) MES5; methyl esterase 5 (A)	Salicylic acid-binding protein 2; NtSABP2; EC=3.1.1.-; Methyl salicylate esterase (At2g23620)		-2.712
Cs1g24850	K00128 aldehyde dehydrogenase (NAD+) [EC:1.2.1.3] | (RefSeq) aldehyde dehydrogenase family 3 member F1 (A)	Aldehyde dehydrogenase family 3 member F1; EC=1.2.1.3 (At4g36250)	1.544	
Cs1g25560	K00025 malate dehydrogenase [EC:1.1.1.37] | (RefSeq) malate dehydrogenase, cytoplasmic-like (A)	Oligopeptide transporter 7; AtOPT7 (At4g10770)	-1.049	
Cs1g25930	K00430 peroxidase [EC:1.11.1.7] | (RefSeq) peroxidase 64-like (A)	Peroxidase 64; Atperox P64; EC=1.11.1.7; ATP17a; PRXR4; Flags:		2.616

		Precursor (At5g42180)		
Cs2g01100	K01193 beta-fructofuranosidase [EC:3.2.1.26] | (RefSeq) LOC109747879; beta-fructofuranosidase, insoluble isoenzyme 3-like (A)	UPF0481 protein At3g47200 (At4g31980_2)	2.886	
Cs2g01120	K01193 beta-fructofuranosidase [EC:3.2.1.26] | (RefSeq) LOC109747879; beta-fructofuranosidase, insoluble isoenzyme 3-like (A)	UPF0481 protein At3g47200 (At4g31980_2)	2.275	
Cs2g01150	K01193 beta-fructofuranosidase [EC:3.2.1.26] | (RefSeq) LOC109747879; beta-fructofuranosidase, insoluble isoenzyme 3-like (A)	UPF0481 protein At3g47200 (At4g31980_2)	2.645	2.218
Cs2g01290	K01613 phosphatidylserine decarboxylase [EC:4.1.1.65] | (RefSeq) phosphatidylserine decarboxylase proenzyme 3-like (A)	C2 and GRAM domain-containing protein At1g03370 (At1g03370_2)		1.017
Cs2g02500	K05933 aminocyclopropanecarboxylate oxidase [EC:1.14.17.4] | (RefSeq) hypothetical protein (A)	1-aminocyclopropane-1-carboxylate oxidase; ACC oxidase; EC=1.14.17.4; Ethylene-forming enzyme; EFE (At1g05010)		-1.030
Cs2g05050	K01568 pyruvate decarboxylase [EC:4.1.1.1] | (RefSeq) hypothetical protein (A)	Pyruvate decarboxylase 1; PDC; EC=4.1.1.1 (At5g54960)	-1.150	
Cs2g05220	K00430 peroxidase [EC:1.11.1.7] | (RefSeq) peroxidase 19 (A)	Peroxidase 19; Atperox P19; EC=1.11.1.7; ATP51; Flags: Precursor (At2g34060)		1.187
Cs2g07170	K03809 NAD(P)H dehydrogenase (quinone) [EC:1.6.5.2] | (RefSeq) probable NAD(P)H dehydrogenase (quinone) FQR1-like 1 (A)	Probable NAD(P)H dehydrogenase (quinone) FQR1-like 1 {ECO:0000305}; EC=1.6.5.2 {ECO:0000250|UniProtKB:Q9LSQ5} (At5g54500)		1.104
Cs2g07700	K01696 tryptophan synthase beta chain [EC:4.2.1.20] | (RefSeq) tryptophan synthase beta chain 1 (A)	Tryptophan synthase beta chain 2, chloroplastic; EC=4.2.1.20; Flags: Precursor (At4g27070)	-1.338	-1.506
Cs2g08310	K16818 phospholipase A1 [EC:3.1.1.32] | (RefSeq) hypothetical protein (A)	Phospholipase A1-Igamma1, chloroplastic; EC=3.1.1.-; Flags: Precursor (At1g30370)	1.088	
Cs2g09200	K00430 peroxidase [EC:1.11.1.7] | (RefSeq) cationic peroxidase 1-like (A)	Cationic peroxidase 1; EC=1.11.1.7; PNPC1; Flags: Precursor (At5g05340)	5.690	
Cs2g09210	K00430 peroxidase [EC:1.11.1.7] | (RefSeq) peroxidase P7-like (A)	Peroxidase 52; Atperox P52; EC=1.11.1.7; ATP49; Flags: Precursor (At5g05340)		1.669
Cs2g09310	K00430 peroxidase [EC:1.11.1.7] | (RefSeq) peroxidase 4 (A)	Peroxidase 4 {ECO:0000250|UniProtKB:Q42578}; EC=1.11.1.7; Flags: Precursor (At5g58400)		1.590
Cs2g10070	K22395 cinnamyl-alcohol dehydrogenase [EC:1.1.1.195] | (RefSeq) flavin-dependent oxidoreductase FOX2 (A)	Berberine bridge enzyme-like 15 {ECO:0000303|PubMed:26037923}; AtBBE-like 15		2.378

		{ECO:0000303|PubMed:26037923}; EC=1.1.1.194 {ECO:0000269|PubMed:26037923}; EC=1.1.1.195 {ECO:0000269|PubMed:26037923}; Protein EMBRYO SAC DEVELOPMENT ARREST 28 {ECO:0000303|PubMed:15634699}; Protein MATERNAL EFFECT EMBRYO ARREST 23 {ECO:0000303|PubMed:15634699}; Flags: Precursor (At2g34790)		
Cs2g10110	K22395 cinnamyl-alcohol dehydrogenase [EC:1.1.1.195] | (RefSeq) tetrahydrocannabinolic acid synthase-like isoform X1 (A)	Cannabidiolic acid synthase-like 1; Flags: Precursor (At5g44440)		2.139
Cs2g10120	K22395 cinnamyl-alcohol dehydrogenase [EC:1.1.1.195] | (RefSeq) tetrahydrocannabinolic acid synthase-like isoform X1 (A)	Cannabidiolic acid synthase; EC=1.21.3.8; CBDA synthase; Flags: Precursor (At4g20820)		1.930
Cs2g10130	K22395 cinnamyl-alcohol dehydrogenase [EC:1.1.1.195] | (RefSeq) tetrahydrocannabinolic acid synthase-like (A)	Berberine bridge enzyme-like 28 {ECO:0000303|PubMed:26037923}; AtBBE-like 28 {ECO:0000303|PubMed:26037923}; EC=1.1.1.- {ECO:0000250|UniProtKB:O64743}; Flags: Precursor (At5g44440)	2.744	1.412
Cs2g10140	K22395 cinnamyl-alcohol dehydrogenase [EC:1.1.1.195] | (RefSeq) tetrahydrocannabinolic acid synthase-like isoform X1 (A)	Berberine bridge enzyme-like 18 {ECO:0000303|PubMed:26037923}; AtBBE-like 18 {ECO:0000303|PubMed:26037923}; EC=1.1.1.- {ECO:0000250|UniProtKB:O64743}; Flags: Precursor (At4g20820)	2.488	
Cs2g10150	K22395 cinnamyl-alcohol dehydrogenase [EC:1.1.1.195] | (RefSeq) tetrahydrocannabinolic acid synthase-like isoform X1 (A)	Berberine bridge enzyme-like 18 {ECO:0000303|PubMed:26037923}; AtBBE-like 18 {ECO:0000303|PubMed:26037923}; EC=1.1.1.- {ECO:0000250|UniProtKB:O64743}; Flags: Precursor (At4g20820)	6.179	
Cs2g10160	K22395 cinnamyl-alcohol dehydrogenase [EC:1.1.1.195] | (RefSeq) tetrahydrocannabinolic acid synthase-like isoform X1 (A)	Berberine bridge enzyme-like 18 {ECO:0000303|PubMed:26037923}; AtBBE-like 18 {ECO:0000303|PubMed:26037923}; EC=1.1.1.- {ECO:0000250|UniProtKB:O64743}; Flags: Precursor (At4g20820)		1.881
Cs2g10200	K22395 cinnamyl-alcohol dehydrogenase [EC:1.1.1.195] | (RefSeq) tetrahydrocannabinolic acid synthase-like isoform X1 (A)	Cannabidiolic acid synthase; EC=1.21.3.8; CBDA synthase; Flags: Precursor (At5g44440)	2.425	
Cs2g10210	K22395 cinnamyl-alcohol dehydrogenase [EC:1.1.1.195] | (RefSeq) tetrahydrocannabinolic acid synthase-like isoform X1 (A)	Cannabidiolic acid synthase-like 1; Flags: Precursor (At4g20820)	2.802	
Cs2g10220	K22395 cinnamyl-alcohol dehydrogenase [EC:1.1.1.195] | (RefSeq) berberine bridge enzyme-like 13 (A)	Berberine bridge enzyme-like 8 {ECO:0000303|PubMed:26037923}; AtBBE-like 8 {ECO:0000303|PubMed:26037923}; EC=1.1.1.- {ECO:0000250|UniProtKB:O64743}; Flags: Precursor (At1g30700)	2.826	

Cs2g10230	K22395 cinnamyl-alcohol dehydrogenase [EC:1.1.1.195] | (RefSeq) berberine bridge enzyme-like 26 (A)	Berberine bridge enzyme-like 14 {ECO:0000303|PubMed:26037923}; AtBBE-like 14 {ECO:0000303|PubMed:26037923}; EC=1.1.1.- {ECO:0000250|UniProtKB:O64743}; Flags: Precursor (At1g34570)	1.864	1.297
Cs2g11050	K01187 alpha-glucosidase [EC:3.2.1.20] | (RefSeq) alpha-glucosidase (A)	Alpha-glucosidase; EC=3.2.1.20; Maltase; Flags: Precursor (At5g11720)	1.942	
Cs2g11840	K01859 chalcone isomerase [EC:5.5.1.6] | (RefSeq) vacuolar-sorting receptor 1-like (A)	Vacuolar-sorting receptor 6; AtVSR6; BP80-like protein d; AtBP80d; Epidermal growth factor receptor-like protein 6; AtELP6; Flags: Precursor (At1g30900)		1.591
Cs2g12730	K10760 adenylate dimethylallyltransferase (cytokinin synthase) [EC:2.5.1.27 2.5.1.112] | (RefSeq) uncharacterized LOC104439822 (A)	(At1g68330)		-1.137
Cs2g13000	K17497 phosphomannomutase [EC:5.4.2.8] | (RefSeq) phosphomannomutase-like (A)	Probable non-specific lipid-transfer protein AKCS9; LTP; Flags: Precursor (At3g18280)	-1.344	-1.096
Cs2g13690	K08101 phytochromobilin:ferredoxin oxidoreductase [EC:1.3.7.4] | (RefSeq) phytochromobilin:ferredoxin oxidoreductase, chloroplastic-like isoform X1 (A)	(At1g71110)	1.305	1.009
Cs2g13960	K00891 shikimate kinase [EC:2.7.1.71] | (RefSeq) probable inactive shikimate kinase like 1, chloroplastic (A)	Probable inactive shikimate kinase like 1, chloroplastic; AtSKL1; Flags: Precursor (At3g26900)		-1.076
Cs2g13990	K09833 homogentisate phytyltransferase / homogentisate geranylgeranyltransferase [EC:2.5.1.115 2.5.1.116] | (RefSeq) probable homogentisate phytyltransferase 1, chloroplastic (A)	Coumarin 8-geranyltransferase 1b, chloroplastic; EC=2.5.1.138 {ECO:0000269|PubMed:25077796}; Prenyltransferase 1b {ECO:0000303|PubMed:25077796}; ClPT1ba {ECO:0000303|PubMed:25077796}; Flags: Precursor (At2g18950)	1.087	1.778
Cs2g14050	K09833 homogentisate phytyltransferase / homogentisate geranylgeranyltransferase [EC:2.5.1.115 2.5.1.116] | (RefSeq) homogentisate geranylgeranyltransferase, chloroplastic-like (A)	Coumarin 8-geranyltransferase 1b, chloroplastic; EC=2.5.1.138 {ECO:0000269|PubMed:25077796}; Prenyltransferase 1b {ECO:0000303|PubMed:25077796}; ClPT1ba {ECO:0000303|PubMed:25077796}; Flags: Precursor (At2g18950)	1.244	1.894
Cs2g14460	K00928 aspartate kinase [EC:2.7.2.4] | (RefSeq) aspartokinase 2, chloroplastic-like (A)	Aspartokinase 3, chloroplastic; EC=2.7.2.4; Aspartate kinase 3; Flags: Precursor (At3g02020)	-1.045	
Cs2g14890	K01689 enolase [EC:4.2.1.11] | (RefSeq) enolase 1, chloroplastic isoform X1 (A)	(At5g47920)		1.457
Cs2g16290	K21373 7-deoxyloganetic acid glucosyltransferase [EC:2.4.1.323] | (RefSeq) hypothetical protein (A)	7-deoxyloganetic acid glucosyltransferase; EC=2.4.1.323; UDP-glucose glucosyltransferase 8; CrUGT8; UDP-glycosyltransferase 709C2 (At1g22400)		1.501

Cs2g16510	K15397 3-ketoacyl-CoA synthase [EC:2.3.1.199] | (RefSeq) 3-ketoacyl-CoA synthase 17-like (A)	Transcription factor RADIALIS (At2g21650)		-4.124
Cs2g16840	K01742 thebaine synthase [EC:4.2.99.24] | (RefSeq) major latex protein 149-like (A)	MLP-like protein 31 (At1g70840)	-1.228	-3.845
Cs2g17520	K13065 shikimate O-hydroxycinnamoyltransferase [EC:2.3.1.133] | (RefSeq) vinorine synthase-like (A)	Salutaridinol 7-O-acetyltransferase {ECO:0000303|PubMed:11404355}; salAT {ECO:0000303|PubMed:11404355}; EC=2.3.1.150 {ECO:0000269|PubMed:11404355, ECO:0000269|PubMed:8537369} (At3g26040)		-8.742
Cs2g21000	K10528 hydroperoxide lyase [EC:4.1.2.-] | (RefSeq) hypothetical protein (A)	Fatty acid hydroperoxide lyase, chloroplastic {ECO:0000303|PubMed:10859201}; LeHPL {ECO:0000303|PubMed:10859201}; EC=4.2.99.- {ECO:0000305}; Cytochrome P450 74B {ECO:0000305} (At4g15440)		-1.134
Cs2g21810	K00430 peroxidase [EC:1.11.1.7] | (RefSeq) hypothetical protein (A)	Peroxidase 27; Atperox P27; EC=1.11.1.7; ATP12a; PRXR7; Flags: Precursor (At3g01190)		1.538
Cs2g22040	K01177 beta-amylase [EC:3.2.1.2] | (RefSeq) hypothetical protein (A)	Beta-amylase; EC=3.2.1.2; 1,4-alpha-D-glucan maltohydrolase (At4g15210)	1.575	1.347
Cs2g22090	K20979 geranyl diphosphate diphosphatase [EC:3.1.7.11] | (RefSeq) probable terpene synthase 9 (A)	Probable terpene synthase 9; RcSeTPS9; EC=4.2.3.- (At3g25830)		-2.685
Cs2g22180	K20979 geranyl diphosphate diphosphatase [EC:3.1.7.11] | (RefSeq) probable terpene synthase 11 (A)	Probable terpene synthase 11; RcSeTPS11; EC=4.2.3.- (At1g61680)		
Cs2g22660	K01593 aromatic-L-amino-acid/L-tryptophan decarboxylase [EC:4.1.1.28 4.1.1.105] | (RefSeq) hypothetical protein (A)	Aromatic-L-amino-acid decarboxylase; AADC; EC=4.1.1.28; DOPA decarboxylase; Tryptophan decarboxylase (At2g20340)	1.556	-1.894
Cs2g23200	K17744 L-galactose dehydrogenase [EC:1.1.1.316] | (RefSeq) hypothetical protein (A)	Probable voltage-gated potassium channel subunit beta; K(+) channel subunit beta; Potassium voltage beta 1; KV-beta1 (At1g04690)	-1.602	
Cs2g23840	K00815 tyrosine aminotransferase [EC:2.6.1.5] | (RefSeq) hypothetical protein (A)	Probable aminotransferase TAT2; EC=2.6.1.-; Tyrosine aminotransferase 2 (At5g53970)		1.425
Cs2g23850	K00815 tyrosine aminotransferase [EC:2.6.1.5] | (RefSeq) hypothetical protein (A)	Probable aminotransferase TAT2; EC=2.6.1.-; Tyrosine aminotransferase 2 (At5g53970)		1.042
Cs2g25450	K00430 peroxidase [EC:1.11.1.7] | (RefSeq) peroxidase 55-like (A)	Peroxidase 55; Atperox P55; EC=1.11.1.7; ATP20a; Flags: Precursor (At5g14130)	1.229	
Cs2g28150	K01087 trehalose 6-phosphate phosphatase [EC:3.1.3.12] | (RefSeq) trehalose-phosphate phosphatase A-like (A)	Protein KINESIN LIGHT CHAIN-RELATED 2 {ECO:0000303|PubMed:23204523}; Kinesin light chain-like protein		1.090

		1 {ECO:0000303|Ref.1}; AtKLC1 {ECO:0000303|Ref.1} (At3g27960)		
Cs2g28370	K23260 scopoletin glucosyltransferase [EC:2.4.1.128] | (RefSeq) scopoletin glucosyltransferase-like (A)	UDP-glycosyltransferase 90A1; EC=2.4.1.- (At2g16890)	1.726	1.956
Cs2g28930	K00549 5-methyltetrahydropteroyltriglutamatehomocysteine methyltransferase [EC:2.1.1.14] | (RefSeq) hypothetical protein (A)	5-methyltetrahydropteroyltriglutamatehomocysteine methyltransferase; EC=2.1.1.14; Cobalamin-independent methionine synthase isozyme; Vitamin-B12-independent methionine synthase isozyme (At5g17920)		1.292
Cs2g29430	K01640 hydroxymethylglutaryl-CoA lyase [EC:4.1.3.4] | (RefSeq) LOW QUALITY PROTEIN: hydroxymethylglutaryl-CoA lyase, mitochondrial (A)	F-box protein At2g32560 (At2g26850)		-1.966
Cs2g29630	K01114 phospholipase C [EC:3.1.4.3] | (RefSeq) non-specific phospholipase C2 (A)	Non-specific phospholipase C2; EC=3.1.-.-; Flags: Precursor (At2g26870)		-1.356
Cs2g29670	K00895 diphosphate-dependent phosphofructokinase [EC:2.7.1.90] | (RefSeq) pyrophosphatefructose 6-phosphate 1-phosphotransferase subunit alpha-like (A)	(At1g50570_2)	1.233	1.301
Cs2g30460	K09753 cinnamoyl-CoA reductase [EC:1.2.1.44] | (RefSeq) cinnamoyl-CoA reductase 1-like isoform X1 (A)	Cinnamoyl-CoA reductase 1; AtCCR1; EC=1.2.1.44; Protein IRREGULAR XYLEM 4 (At5g19440)	2.798	
Cs2g30470	K09753 cinnamoyl-CoA reductase [EC:1.2.1.44] | (RefSeq) cinnamoyl-CoA reductase 2-like (A)	Cinnamoyl-CoA reductase 1; AtCCR1; EC=1.2.1.44; Protein IRREGULAR XYLEM 4 (At1g51410)		-1.535
Cs2g30610	K01593 aromatic-L-amino-acid/L-tryptophan decarboxylase [EC:4.1.1.28 4.1.1.105] | (RefSeq) tyrosine/DOPA decarboxylase 1-like (A)	Tyrosine/DOPA decarboxylase 2; Includes: DOPA decarboxylase; DDC; EC=4.1.1.28; Includes: Tyrosine decarboxylase; EC=4.1.1.25 (At2g20340)		-1.272
Cs3g02270	K00430 peroxidase [EC:1.11.1.7] | (RefSeq) lignin-forming anionic peroxidase-like (A)	Lignin-forming anionic peroxidase; EC=1.11.1.7; Flags: Precursor (At1g14550)		1.775
Cs3g02280	K00430 peroxidase [EC:1.11.1.7] | (RefSeq) lignin-forming anionic peroxidase-like (A)	Lignin-forming anionic peroxidase; EC=1.11.1.7; Flags: Precursor (At1g14550)		1.108
Cs3g03590	K22395 cinnamyl-alcohol dehydrogenase [EC:1.1.1.195] | (RefSeq) inactive tetrahydrocannabinolic acid synthase-like (A)	Cannabidiolic acid synthase-like 2; Flags: Precursor (At4g20860)	2.989	2.112
Cs3g03620	K01695 tryptophan synthase alpha chain [EC:4.2.1.20] | (RefSeq) tryptophan synthase alpha chain-like (A)	Tryptophan synthase alpha chain; EC=4.2.1.20; Indole synthase; Indole-3-glycerol-phosphate lyase; EC=4.1.2.8 (At4g02610)	5.206	
Cs3g03640	K13789 geranylgeranyl diphosphate synthase, type II [EC:2.5.1.1 2.5.1.10 2.5.1.29] | (RefSeq) geranylgeranyl pyrophosphate synthase, chloroplastic-like (A)	Geranylgeranyl pyrophosphate synthase, chloroplastic; GGPP synthase; GGPS; EC=2.5.1.-; (2E,6E)-farnesyl diphosphate synthase; Dimethylallyltranstransferase; EC=2.5.1.1; Farnesyl diphosphate	3.498	

		synthase; Farnesyltranstransferase; EC=2.5.1.29; Geranyltranstransferase; EC=2.5.1.10; Flags: Precursor (At4g36810)		
Cs3g03970	K13065 shikimate O-hydroxycinnamoyltransferase [EC:2.3.1.133] | (RefSeq) hypothetical protein (A)	BAHD acyltransferase At5g47980; EC=2.3.1.- (At3g26040)		-1.270
Cs3g04040	K10249 elongation of very long chain fatty acids protein 4 [EC:2.3.1.199] | (RefSeq) elongation of fatty acids protein A-like (A)	Elongation of fatty acids protein 3-like; Protein ELO3-like; EC=2.3.1.-; Protein HIGH EXPRESSION OF OSMOTICALLY RESPONSIVE GENES 3; Very long-chain fatty acid condensing enzyme HOS3; VLCFA condensing enzyme HOS3 (At3g06470)		-2.061
Cs3g04700	K09833 homogentisate phytyltransferase / homogentisate geranylgeranyltransferase [EC:2.5.1.115 2.5.1.116] | (RefSeq) hypothetical protein (A)	Coumarin 8-geranyltransferase 1b, chloroplastic; EC=2.5.1.138 {ECO:0000269|PubMed:25077796}; Prenyltransferase 1b {ECO:0000303|PubMed:25077796}; ClPT1ba {ECO:0000303|PubMed:25077796}; Flags: Precursor (At2g18950)	2.365	-2.120
Cs3g06080	K10525 allene oxide cyclase [EC:5.3.99.6] | (RefSeq) allene oxide cyclase 4, chloroplastic-like (A)	Allene oxide cyclase, chloroplastic {ECO:0000303|Ref.1, ECO:0000303|Ref.2}; OsAOC {ECO:0000303|Ref.1}; EC=5.3.99.6 {ECO:0000269|PubMed:23347338}; Protein COLEOPTILE PHOTOMORPHOGENESIS 2 {ECO:0000303|PubMed:23347338}; Protein HEBIBA {ECO:0000303|PubMed:14605232}; Flags: Precursor (At1g13280)	-1.814	-1.501
Cs3g07670	K01193 beta-fructofuranosidase [EC:3.2.1.26] | (RefSeq) LOC109747879; beta-fructofuranosidase, insoluble isoenzyme 3-like (A)	UPF0481 protein At3g47200 (At2g36430)		1.219
Cs3g08290	K21568 pinoresinol/lariciresinol reductase [EC:1.23.1.1 1.23.1.2 1.23.1.3 1.23.1.4] | (RefSeq) hypothetical protein (A)	Bifunctional pinoresinol-lariciresinol reductase 2; PLR-Lu2; (+)-lariciresinol reductase; EC=1.23.1.2; (+)-pinoresinol reductase; EC=1.23.1.1 (At1g32100)	3.685	6.902
Cs3g08320	K21568 pinoresinol/lariciresinol reductase [EC:1.23.1.1 1.23.1.2 1.23.1.3 1.23.1.4] | (RefSeq) hypothetical protein (A)	Isoflavone reductase homolog; EC=1.3.1.- (At1g32100)	1.066	2.004
Cs3g08510	K01904 4-coumarate CoA ligase [EC:6.2.1.12] | (RefSeq) hypothetical protein (A)	4-coumarateCoA ligase-like 6; EC=6.2.1.-; 4-coumarateCoA ligase isoform 7; At4CL7 (At4g19010)	-1.625	
Cs3g09730	K01187 alpha-glucosidase [EC:3.2.1.20] | (RAP-DB) Os07g0421300; Similar to Alpha glucosidase-like protein. (A)	Probable glucan 1,3-alpha-glucosidase; EC=3.2.1.84; Glucosidase II subunit alpha; Protein PRIORITY IN SWEET LIFE 5; Protein RADIAL SWELLING 3; Flags: Precursor (At3g23640)		1.120
Cs3g09750	K01187 alpha-glucosidase [EC:3.2.1.20] | (RefSeq) alpha-glucosidase 2-like (A)			1.470
Cs3g09800	K16055 trehalose 6-phosphate synthase/phosphatase [EC:2.4.1.15	Alpha,alpha-trehalose-phosphate synthase [UDP-forming] 5;		1.137

	3.1.3.12] | (RefSeq) alpha,alpha-trehalose-phosphate synthase [UDP-forming] 5 (A)	EC=2.4.1.15; Trehalose-6-phosphate synthase 5; AtTPS5 (At4g17770)		
Cs3g10940	K01824 cholestenol Delta-isomerase [EC:5.3.3.5] | (RefSeq) hypothetical protein (A)	Probable 3-beta-hydroxysteroid-Delta(8),Delta(7)-isomerase; EC=5.3.3.5; Cholestenol Delta-isomerase; Delta(8)-Delta(7) sterol isomerase; D8-D7 sterol isomerase (At1g20050)		-1.149
Cs3g11060	K00514 zeta-carotene desaturase [EC:1.3.5.6] | (RefSeq) zeta-carotene desaturase, chloroplastic/chromoplastic-like (A)	Zeta-carotene desaturase, chloroplastic/chromoplastic; EC=1.3.5.6; 9,9'-di-cis-zeta-carotene desaturase; Carotene 7,8-desaturase; Flags: Precursor (At3g04870)		1.040
Cs3g11170	K00514 zeta-carotene desaturase [EC:1.3.5.6] | (RefSeq) CitZDS; ZDS (A)	Zeta-carotene desaturase, chloroplastic/chromoplastic; EC=1.3.5.6; 9,9'-di-cis-zeta-carotene desaturase; Carotene 7,8-desaturase; Flags: Precursor (At3g04870)	1.422	
Cs3g11490	K01738 cysteine synthase A [EC:2.5.1.47] | (RefSeq) hypothetical protein (A)	Cysteine synthase, chloroplastic/chromoplastic; EC=2.5.1.47; CSase B; CS-B; O-acetylserine (thiol)-lyase; O-acetylserine sulfhydrylase; OAS-TL B; Flags: Precursor (At2g43750)	1.049	1.627
Cs3g11520	K01738 cysteine synthase A [EC:2.5.1.47] | (RefSeq) cysteine synthase-like (A)	Cysteine synthase {ECO:0000303|PubMed:8280125}; CSase; EC=2.5.1.47 {ECO:0000269|PubMed:8280125, ECO:0000269|PubMed:9013806}; Beta-PA/CSase; EC=2.5.1.51 {ECO:0000269|PubMed:8280125, ECO:0000269|PubMed:9013806}; Beta-pyrazolylalanine synthase {ECO:0000303|PubMed:8280125}; EC=4.2.1.50 {ECO:0000269|PubMed:8280125, ECO:0000269|PubMed:9013806}; L-mimosine synthase; EC=2.5.1.52 {ECO:0000269|PubMed:9013806}; O-acetylserine (thiol)-lyase; OAS-TL; O-acetylserine sulfhydrylase (At5g28020)		1.745
Cs3g14390	K00128 aldehyde dehydrogenase (NAD+) [EC:1.2.1.3] | (RefSeq) aldehyde dehydrogenase 5, mitochondrial-like (A)	Translationally-controlled tumor protein homolog; TCTP (At3g16640)		-2.145
Cs3g14430	K21026 acetylajmaline esterase [EC:3.1.1.80] | (RefSeq) hypothetical protein (A)	GDSL esterase/lipase At5g03980; EC=3.1.1.-; Extracellular lipase At5g03980; Flags: Precursor (At5g03980)		-1.493
Cs3g14520	K22097 3-O-acetylpapaveroxine carboxylesterase [EC:3.1.1.105] | (RefSeq) carboxylesterase 1-like (A)	Probable carboxylesterase 15; AtCXE15; EC=3.1.1.1 (At5g06570)	1.890	
Cs3g16400	K01762 1-aminocyclopropane-1-carboxylate synthase [EC:4.4.1.14] | (RefSeq) 1-aminocyclopropane-1-carboxylate synthase 8 (A)	1-aminocyclopropane-1-carboxylate synthase 8; ACC synthase 8; EC=4.4.1.14; S-adenosyl-L-methionine methylthioadenosine-lyase 8 (At4g37770)		2.594
Cs3g16440	K00826 branched-chain amino acid aminotransferase [EC:2.6.1.42] |	Branched-chain-amino-acid aminotransferase 2, chloroplastic;		1.092

	(RefSeq) branched-chain-amino-acid aminotransferase 2, chloroplastic (A)	Atbcat-2; EC=2.6.1.42; Flags: Precursor (At1g10070)		
Cs3g16700	K01610 phosphoenolpyruvate carboxykinase (ATP) [EC:4.1.1.49] | (RefSeq) phosphoenolpyruvate carboxykinase [ATP]-like (A)	Phosphoenolpyruvate carboxykinase (ATP); PEP carboxykinase; PEPCK; EC=4.1.1.49 (At4g37870)		2.281
Cs3g16870	K00025 malate dehydrogenase [EC:1.1.1.37] | (RefSeq) malate dehydrogenase, cytoplasmic-like (A)	Oligopeptide transporter 4; AtOPT4 (At5g64410)	1.016	
Cs3g17650	K00850 6-phosphofructokinase 1 [EC:2.7.1.11] | (RefSeq) ATP-dependent 6-phosphofructokinase 6-like (A)	Purine permease 1 {ECO:0000303|PubMed:10662864}; AtPUP1 {ECO:0000303|PubMed:10662864} (At1g28230)		-1.500
Cs3g17940	K00121 S-(hydroxymethyl)glutathione dehydrogenase / alcohol dehydrogenase [EC:1.1.1.284 1.1.1.1] | (RefSeq) alcohol dehydrogenase-like 2 (A)	Alcohol dehydrogenase-like 2; EC=1.1.1.1 {ECO:0000250|UniProtKB:P06525} (At1g22440)		1.096
Cs3g18080	K21374 7-deoxyloganetin glucosyltransferase [EC:2.4.1.324] | (RefSeq) 7-deoxyloganetin glucosyltransferase-like (A)	7-deoxyloganetin glucosyltransferase; EC=2.4.1.324; Genipin glucosyltransferase; UDP-glucose glucosyltransferase 2; GjUGT2; UDP-glycosyltransferase 85A24 (At1g22360)		-2.298
Cs3g18100	K21374 7-deoxyloganetin glucosyltransferase [EC:2.4.1.324] | (RefSeq) 7-deoxyloganetin glucosyltransferase-like (A)	7-deoxyloganetin glucosyltransferase; EC=2.4.1.324; Genipin glucosyltransferase; UDP-glucose glucosyltransferase 2; GjUGT2; UDP-glycosyltransferase 85A24 (At1g22360)		-1.548
Cs3g18450	K01087 trehalose 6-phosphate phosphatase [EC:3.1.3.12] | (RefSeq) probable trehalose-phosphate phosphatase D (A)	Probable trehalose-phosphate phosphatase D; AtTPPD; EC=3.1.3.12; Trehalose 6-phosphate phosphatase (At1g35910)		1.440
Cs3g18630	K11262 acetyl-CoA carboxylase / biotin carboxylase 1 [EC:6.4.1.2 6.3.4.14 2.1.3.15] | (RefSeq) acetyl-CoA carboxylase 1-like (A)	Acetyl-CoA carboxylase 1; AtACC1; EC=6.4.1.2 {ECO:0000269|PubMed:9008389}; Protein EMBRYO DEFECTIVE 22; Protein GURKE; Protein PASTICCINO 3; Includes: Biotin carboxylase; EC=6.3.4.14 (At1g36160)		1.119
Cs3g19690	K13600 chlorophyllide a oxygenase [EC:1.14.13.122] | (RefSeq) chlorophyllide a oxygenase, chloroplastic (A)	Chlorophyllide a oxygenase, chloroplastic; Chlorophyll a oxygenase; EC=1.14.13.122; Chlorophyll b synthase; AtCAO; Flags: Precursor (At1g44446)	1.823	
Cs3g20330	K00660 chalcone synthase [EC:2.3.1.74] | (RefSeq) chalcone synthase-like (A)	Chalcone synthase; EC=2.3.1.74; Naringenin-chalcone synthase (At5g13930)		-1.250
Cs3g20350	K01913 acetate-CoA ligase [EC:6.2.1.1] | (RefSeq) acetate/butyrateCoA ligase AAE7, peroxisomal-like isoform X1 (A)	Probable acyl-activating enzyme 6; EC=6.2.1.-; AMP-binding protein 6; AtAMPBP6 (At5g16340)		-1.085
Cs3g20490	K00166 2-oxoisovalerate dehydrogenase E1 component alpha subunit [EC:1.2.4.4] | (RefSeq) 2-oxoisovalerate dehydrogenase subunit alpha 1, mitochondrial (A)	2-oxoisovalerate dehydrogenase subunit alpha 1, mitochondrial; EC=1.2.4.4; Branched-chain alpha-keto acid dehydrogenase E1 component alpha chain; BCKDE1A; BCKDH E1-alpha; Flags: Precursor (At1g21400)		-1.837

Cs3g20640	K00660 chalcone synthase [EC:2.3.1.74] | (RefSeq) chalcone synthase 2-like (A)	Chalcone synthase 2; EC=2.3.1.74; Naringenin-chalcone synthase 2 (At5g13930)	4.405	
Cs3g20650	K13066 caffeic acid 3-O-methyltransferase [EC:2.1.1.68] | (RefSeq) caffeic acid 3-O-methyltransferase (A)	Caffeic acid 3-O-methyltransferase; CAOMT; COMT; EC=2.1.1.68; S-adenosysl-L-methionine:caffeic acid 3-O-methyltransferase (At5g54160)	3.127	
Cs3g20680	K00660 chalcone synthase [EC:2.3.1.74] | (RefSeq) chalcone synthase 2-like (A)	Chalcone synthase 2; EC=2.3.1.74; Naringenin-chalcone synthase 2 (At5g13930)	3.814	
Cs3g20800	K18857 alcohol dehydrogenase class-P [EC:1.1.1.1] | (RefSeq) alcohol dehydrogenase-like (A)	Alcohol dehydrogenase; EC=1.1.1.1 {ECO:0000250|UniProtKB:P06525} (At1g77120)	-1.541	
Cs3g20810	K18857 alcohol dehydrogenase class-P [EC:1.1.1.1] | (RefSeq) alcohol dehydrogenase (A)	Alcohol dehydrogenase; EC=1.1.1.1 {ECO:0000250|UniProtKB:P06525} (At1g77120)	-2.108	
Cs3g21280	K03841 fructose-1,6-bisphosphatase I [EC:3.1.3.11] | (RefSeq) fructose-1,6-bisphosphatase, cytosolic (A)	Fructose-1,6-bisphosphatase, cytosolic; FBPase; EC=3.1.3.11; CY-F1; D-fructose-1,6-bisphosphate 1-phosphohydrolase (At1g43670)		1.036
Cs3g21730	K00430 peroxidase [EC:1.11.1.7] | (RefSeq) peroxidase 12 (A)	Peroxidase 12; Atperox P12; EC=1.11.1.7; ATP4a; PRXR6; Flags: Precursor (At1g71695)		-1.066
Cs3g23560	K01176 alpha-amylase [EC:3.2.1.1] | (RefSeq) hypothetical protein (A)	Alpha-amylase; EC=3.2.1.1 {ECO:0000250|UniProtKB:P00693}; 1,4-alpha-D-glucan glucanohydrolase; Flags: Precursor (At4g25000)	1.160	
Cs3g23660	K15728 phosphatidate phosphatase LPIN [EC:3.1.3.4] | (RefSeq) phosphatidate phosphatase PAH2 (A)	Phosphatidate phosphatase PAH2; EC=3.1.3.4; Phosphatidic acid phosphohydrolase 2; AtPAH2 (At5g42870)		1.656
Cs3g26600	K00430 peroxidase [EC:1.11.1.7] | (RefSeq) peroxidase 64 (A)	Peroxidase 64; Atperox P64; EC=1.11.1.7; ATP17a; PRXR4; Flags: Precursor (At5g42180)		1.204
Cs3g26720	K01115 phospholipase D1/2 [EC:3.1.4.4] | (RefSeq) hypothetical protein (A)	Phospholipase D delta {ECO:0000303|PubMed:11891260}; AtPLDdelta {ECO:0000303|PubMed:11891260}; PLD delta {ECO:0000303|PubMed:11891260}; EC=3.1.4.4 {ECO:0000269|PubMed:11706190, ECO:0000269|PubMed:12397060} (At4g35790)	-1.036	
Cs3g27120	K13065 shikimate O-hydroxycinnamoyltransferase [EC:2.3.1.133] | (RefSeq) vinorine synthase-like (A)	Vinorine synthase; EC=2.3.1.160 (At3g26040)	-1.216	-3.982
Cs3g27220	K00700 1,4-alpha-glucan branching enzyme [EC:2.4.1.18] | (RefSeq) 1,4-alpha-glucan-branching enzyme 1, chloroplastic/amyloplastic-like (A)	1,4-alpha-glucan-branching enzyme 1, chloroplastic/amyloplastic; EC=2.4.1.18; Starch branching enzyme I; Flags: Precursor; Fragment (At5g03650)	1.861	
Cs4g01930	K13065 shikimate O-hydroxycinnamoyltransferase [EC:2.3.1.133] | (RefSeq) shikimate O-hydroxycinnamoyltransferase-like (A)	Shikimate O-hydroxycinnamoyltransferase; EC=2.3.1.133; Hydroxycinnamoyl transferase; Hydroxycinnamoyl-Coenzyme A		-1.746

		shikimate/quinate hydroxycinnamoyltransferase (At5g48930)		
Cs4g02360	K13065 shikimate O-hydroxycinnamoyltransferase [EC:2.3.1.133] | (RefSeq) shikimate O-hydroxycinnamoyltransferase-like (A)	Shikimate O-hydroxycinnamoyltransferase; EC=2.3.1.133; Hydroxycinnamoyl transferase; Hydroxycinnamoyl-Coenzyme A shikimate/quinate hydroxycinnamoyltransferase (At5g48930)	1.711	
Cs4g02380	K13065 shikimate O-hydroxycinnamoyltransferase [EC:2.3.1.133] | (RefSeq) shikimate O-hydroxycinnamoyltransferase-like (A)	Shikimate O-hydroxycinnamoyltransferase; EC=2.3.1.133; Hydroxycinnamoyl transferase; Hydroxycinnamoyl-Coenzyme A shikimate/quinate hydroxycinnamoyltransferase (At5g48930)	1.299	
Cs4g03330	K01904 4-coumarateCoA ligase [EC:6.2.1.12] | (RefSeq) hypothetical protein (A)	4-coumarateCoA ligase 1; 4CL 1; EC=6.2.1.12; 4-coumaroyl-CoA synthase 1 (At3g21240)	1.132	3.074
Cs4g03740	K00430 peroxidase [EC:1.11.1.7] | (RefSeq) hypothetical protein (A)	Peroxidase 16; Atperox P16; EC=1.11.1.7; ATP22a; Flags: Precursor (At2g18980)	-1.821	
Cs4g04430	K01214 isoamylase [EC:3.2.1.68] | (RefSeq) isoamylase 1, chloroplastic (A)	Isoamylase 1, chloroplastic; AtISA1; EC=3.2.1.68; Flags: Precursor (At2g39930)		1.220
Cs4g04630	K01853 cycloartenol synthase [EC:5.4.99.8] | (RefSeq) hypothetical protein (A)	Cycloartenol synthase; RcCAS; EC=5.4.99.8 (At2g07050)	1.784	-2.809
Cs4g04640	K00511 squalene monooxygenase [EC:1.14.14.17] | (RefSeq) squalene epoxidase 3-like (A)	Squalene epoxidase 3; AtSQE3; EC=1.14.14.17 {ECO:0000269|PubMed:17426032} (At4g37760)	2.512	
Cs4g04730	K01853 cycloartenol synthase [EC:5.4.99.8] | (RefSeq) cycloartenol synthase-like (A)	Cycloartenol synthase; AtCYC; EC=5.4.99.8; 2,3-epoxysqualenecycloartenol cyclase (At2g07050)		1.060
Cs4g05200	K15227 arogenate dehydrogenase (NADP+), plant [EC:1.3.1.78] | (RefSeq) hypothetical protein (A)	Arogenate dehydrogenase 1, chloroplastic; EC=1.3.1.78; TYRATC; TyrAAT1; Flags: Precursor (At5g34930)		1.176
Cs4g06620	K14157 alpha-aminoadipic semialdehyde synthase [EC:1.5.1.8 1.5.1.9] | (RefSeq) alpha-aminoadipic semialdehyde synthase (A)	Alpha-aminoadipic semialdehyde synthase; cAt-LKR/SDH; LKR/SDH; Includes: Lysine ketoglutarate reductase; LKR; EC=1.5.1.8; Includes: Saccharopine dehydrogenase; EC=1.5.1.9; cAt-SDH; SDH (At4g33150)	1.865	
Cs4g08360	K00025 malate dehydrogenase [EC:1.1.1.37] | (RefSeq) malate dehydrogenase, cytoplasmic-like (A)	Oligopeptide transporter 1; AtOPT1 (At5g55930)	1.141	
Cs4g08370	K00025 malate dehydrogenase [EC:1.1.1.37] | (RefSeq) malate dehydrogenase, cytoplasmic-like (A)	Oligopeptide transporter 1; AtOPT1 (At5g55930)		-1.145
Cs4g08380	K00025 malate dehydrogenase [EC:1.1.1.37] | (RefSeq) malate dehydrogenase, cytoplasmic-like (A)	Oligopeptide transporter 1; AtOPT1 (At5g55930)		-1.837
Cs4g08990	K00640 serine O-acetyltransferase [EC:2.3.1.30] | (RefSeq) hypothetical protein (A)	Serine acetyltransferase 3, mitochondrial; AtSAT-3; AtSERAT2;2; SAT-m; EC=2.3.1.30; Flags: Precursor (At3g13110)		

Cs4g10160	K09833 homogentisate phytyltransferase / homogentisate geranylgeranyltransferase [EC:2.5.1.115 2.5.1.116] | (RefSeq) homogentisate phytyltransferase 1, chloroplastic-like (A)	Coumarin 8-geranyltransferase 1b, chloroplastic; EC=2.5.1.138 {ECO:0000269|PubMed:25077796}; Prenyltransferase 1b {ECO:0000303|PubMed:25077796}; ClPT1ba {ECO:0000303|PubMed:25077796}; Flags: Precursor (At3g11950)	2.444	
Cs4g10240	K13789 geranylgeranyl diphosphate synthase, type II [EC:2.5.1.1 2.5.1.10 2.5.1.29] | (RefSeq) hypothetical protein (A)	Geranylgeranyl pyrophosphate synthase, chloroplastic; GGPP synthase; GGPS; EC=2.5.1.-; (2E,6E)-farnesyl diphosphate synthase; Dimethylallyltranstransferase; EC=2.5.1.1; Farnesyl diphosphate synthase; Farnesyltranstransferase; EC=2.5.1.29; Geranyltranstransferase; EC=2.5.1.10; Flags: Precursor (At2g18620)	-1.409	-1.909
Cs4g10970	K13509 lysophosphatidate acyltransferase [EC:2.3.1.51] | (RefSeq) 1-acyl-sn-glycerol-3-phosphate acyltransferase (A)	1-acyl-sn-glycerol-3-phosphate acyltransferase; 1-AGP acyltransferase; 1-AGPAT; EC=2.3.1.51; Lysophosphatidic acid acyltransferase; LPAAT (YDL052c)		-1.282
Cs4g11970	K01188 beta-glucosidase [EC:3.2.1.21] | (RefSeq) putative beta-glucosidase 41 (A)	Putative beta-glucosidase 41; AtBGLU41; EC=3.2.1.21; Flags: Precursor (At5g54570)	2.732	3.222
Cs4g12540	K06892 feruloyl-CoA ortho-hydroxylase [EC:1.14.11.-] | (RefSeq) hypothetical protein (A)	Feruloyl CoA ortho-hydroxylase 1; EC=1.14.11.- (At3g12900)	1.199	2.220
Cs4g13070	K00850 6-phosphofructokinase 1 [EC:2.7.1.11] | (RefSeq) ATP-dependent 6-phosphofructokinase 3-like (A)	ATP-dependent 6-phosphofructokinase 3 {ECO:0000255|HAMAP-Rule:MF_03186}; ATP-PFK 3 {ECO:0000255|HAMAP-Rule:MF_03186}; Phosphofructokinase 3 {ECO:0000255|HAMAP-Rule:MF_03186}; EC=2.7.1.11 {ECO:0000255|HAMAP-Rule:MF_03186}; Phosphohexokinase 3 {ECO:0000255|HAMAP-Rule:MF_03186} (At5g56630)	-1.258	
Cs4g13490	K01762 1-aminocyclopropane-1-carboxylate synthase [EC:4.4.1.14] | (RefSeq) 1-aminocyclopropane-1-carboxylate synthase 7 (A)	1-aminocyclopropane-1-carboxylate synthase 7; ACC synthase 7; EC=4.4.1.14; S-adenosyl-L-methionine methylthioadenosine-lyase 7 (At4g26200)		1.634
Cs4g13640	K22395 cinnamyl-alcohol dehydrogenase [EC:1.1.1.195] | (RefSeq) uncharacterized LOC100817142 (A)	Protein EFFECTOR OF TRANSCRIPTION 2 {ECO:0000303|PubMed:17991462}; AtET2 {ECO:0000303|PubMed:17991462} (At5g56780)		1.033
Cs4g13870	K05933 aminocyclopropanecarboxylate oxidase [EC:1.14.17.4] | (RefSeq) hypothetical protein (A)	1-aminocyclopropane-1-carboxylate oxidase 1; ACC oxidase 1; AtACO1; EC=1.14.17.4 (At2g19590)	-1.124	
Cs4g14970	K01913 acetate-CoA ligase [EC:6.2.1.1] | (RefSeq) acetate/butyrateCoA ligase AAE7, peroxisomal-like (A)	Probable acyl-activating enzyme 6; EC=6.2.1.-; AMP-binding protein 6; AtAMPBP6 (At5g16340)		-1.048
Cs4g16860	K21371 crocetin glucosyltransferase [EC:2.4.1.271] | (RefSeq) crocetin glucosyltransferase, chloroplastic-like (A)	Probable strigolactone esterase DAD2; 3.1.-.-; Protein DECREASED APICAL DOMINANCE 2 (At3g24420)		-3.699

Cs4g17860	K00430 peroxidase [EC:1.11.1.7] | (RefSeq) peroxidase 25 (A)	Peroxidase 25; Atperox P25; EC=1.11.1.7; Flags: Precursor (At2g41480)		-1.000
Cs4g18340	K01193 beta-fructofuranosidase [EC:3.2.1.26] | (RefSeq) beta-fructofuranosidase, insoluble isoenzyme CWINV1 (A)	Beta-fructofuranosidase, insoluble isoenzyme CWINV1; EC=3.2.1.26; Cell wall beta-fructosidase 1; AtbetaFRUCT1; Cell wall invertase 1; AtcwINV1; Sucrose hydrolase 1; Flags: Precursor (At3g13790)		3.456
Cs4g18980	K00850 6-phosphofructokinase 1 [EC:2.7.1.11] | (RefSeq) hypothetical protein (A)	ATP-dependent 6-phosphofructokinase 3 {ECO:0000255|HAMAP-Rule:MF_03186}; ATP-PFK 3 {ECO:0000255|HAMAP-Rule:MF_03186}; Phosphofructokinase 3 {ECO:0000255|HAMAP-Rule:MF_03186}; EC=2.7.1.11 {ECO:0000255|HAMAP-Rule:MF_03186}; Phosphohexokinase 3 {ECO:0000255|HAMAP-Rule:MF_03186} (At4g32840)	-1.602	-1.466
Cs4g19000	K08241 jasmonate O-methyltransferase [EC:2.1.1.141] | (RefSeq) jasmonate O-methyltransferase-like (A)	Early nodulin-93; N-93 (At5g25940)		-1.094
Cs4g19460	K17913 carlactone synthase / all-trans-10'-apo-beta-carotenal 13,14-cleaving dioxygenase [EC:1.13.11.69 1.13.11.70] | (RefSeq) hypothetical protein (A)	Carotenoid cleavage dioxygenase 8 homolog B, chloroplastic; OsCCD8b; EC=1.13.11.69 {ECO:0000269|PubMed:22422982}; EC=1.13.11.70 {ECO:0000269|PubMed:18637791}; Protein DWARF-10; Protein MAX4 homolog; Flags: Precursor (At4g32810)	1.942	1.251
Cs5g01410	K17911 beta-carotene isomerase [EC:5.2.1.14] | (RefSeq) beta-carotene isomerase D27, chloroplastic-like isoform X1 (A)	Beta-carotene isomerase D27, chloroplastic; EC=5.2.1.14; Protein DWARF-27 homolog; AtD27; Flags: Precursor (At1g64680)		-1.707
Cs5g03060	K20772 1-aminocyclopropane-1-carboxylate synthase 1/2/6 [EC:4.4.1.14] | (RefSeq) hypothetical protein (A)	1-aminocyclopropane-1-carboxylate synthase; ACC synthase; EC=4.4.1.14; S-adenosyl-L-methionine methylthioadenosine-lyase (At4g11280)	1.327	1.903
Cs5g04240	K00549 5-methyltetrahydropteroyltriglutamatehomocysteine methyltransferase [EC:2.1.1.14] | (RefSeq) protein QUIRKY-like (A)	Protein QUIRKY {ECO:0000303|PubMed:19180193} (At1g74720)		1.383
Cs5g05650	K00766 anthranilate phosphoribosyltransferase [EC:2.4.2.18] | (RefSeq) anthranilate phosphoribosyltransferase, chloroplastic (A)	Anthranilate phosphoribosyltransferase, chloroplastic; EC=2.4.2.18; Flags: Precursor (At5g17990)	-1.039	
Cs5g05990	K00276 primary-amine oxidase [EC:1.4.3.21] | (RefSeq) hypothetical protein (A)	U-box domain-containing protein 12; EC=2.3.2.27; Plant U-box protein 12; OsPUB12; RING-type E3 ubiquitin transferase PUB12 {ECO:0000305} (At3g26600)	1.336	1.395
Cs5g07980	K13065 shikimate O-hydroxycinnamoyltransferase [EC:2.3.1.133] | (RefSeq) vinorine synthase-like (A)	Taxadien-5-alpha-ol O-acetyltransferase; EC=2.3.1.162; Taxa-4(20),11(12)-dien-5alpha-ol-O-acetyltransferase; Taxadienol acetyltransferase (At5g02890)	2.401	3.685
Cs5g08850	K00750 glycogenin [EC:2.4.1.186] | (RefSeq) putative	Putative glucuronosyltransferase PGSIP7; EC=2.4.1.-;		1.243

	glucuronosyltransferase PGSIP8 (A)	Glycogenin-like protein 7; Plant glycogenin-like starch initiation protein 7 (At4g16600)		
Cs5g09470	K00276 primary-amine oxidase [EC:1.4.3.21] | (RefSeq) primary amine oxidase-like (A)	Primary amine oxidase; EC=1.4.3.21 {ECO:0000250|UniProtKB:P46883}; Amine oxidase [copper-containing]; Flags: Precursor (At4g12290)		1.008
Cs5g10630	K15095 (+)-neomenthol dehydrogenase [EC:1.1.1.208] | (RefSeq) hypothetical protein (A)	Salutaridine reductase {ECO:0000303|PubMed:16968522}; EC=1.1.1.248 {ECO:0000269|PubMed:16968522, ECO:0000269|PubMed:19648114} (At3g61220)		-2.142
Cs5g11570	K00901 diacylglycerol kinase (ATP) [EC:2.7.1.107] | (RefSeq) diacylglycerol kinase 1 (A)	Diacylglycerol kinase 1; AtDGK1; DAG kinase 1; EC=2.7.1.107; Diglyceride kinase 1; DGK 1 (At5g07920)		1.043
Cs5g11730	K05280 flavonoid 3'-monooxygenase [EC:1.14.13.21] | (RefSeq) flavonoid 3'-monooxygenase (A)	Flavonoid 3'-monooxygenase; EC=1.14.13.21; Cytochrome P450 75B2; Flavonoid 3'-hydroxylase (At5g07990)	-1.621	-4.299
Cs5g12460	K01599 uroporphyrinogen decarboxylase [EC:4.1.1.37] | (RefSeq) hypothetical protein (A)	Peptide-N4-(N-acetyl-beta-glucosaminyl)asparagine amidase A; PNGase A; EC=3.5.1.52; Glycopeptide N-glycosidase; N-glycanase; Contains: Peptide-N4-(N-acetyl-beta-glucosaminyl)asparagine amidase A light chain; PNGase A small chain; PNGase A subunit B; Contains: Peptide-N4-(N-acetyl-beta-glucosaminyl)asparagine amidase A heavy chain; PNGase A large chain; PNGase A subunit A (At3g14920)		1.460
Cs5g13080	K05356 all-trans-nonaprenyl-diphosphate synthase [EC:2.5.1.84 2.5.1.85] | (RefSeq) hypothetical protein (A)	SH2 domain-containing protein B {ECO:0000303|PubMed:15063865}; AtSHB {ECO:0000303|PubMed:15063865}; STAT-type linker-SH2 domain factor B {ECO:0000303|PubMed:15073273} (At1g78540)		1.060
Cs5g13580	K13066 caffeic acid 3-O-methyltransferase [EC:2.1.1.68] | (RefSeq) caffeic acid 3-O-methyltransferase-like (A)	Caffeic acid 3-O-methyltransferase; CAOMT; COMT; EC=2.1.1.68; S-adenosysl-L-methionine:caffeic acid 3-O-methyltransferase (At5g54160)	3.547	
Cs5g14370	K09840 9-cis-epoxycarotenoid dioxygenase [EC:1.13.11.51] | (RefSeq) hypothetical protein (A)	9-cis-epoxycarotenoid dioxygenase NCED1, chloroplastic; EC=1.13.11.51; PvNCED1; Flags: Precursor (At3g14440)		-1.791
Cs5g14480	K04125 gibberellin 2-oxidase [EC:1.14.11.13] | (RefSeq) hypothetical protein (A)	Gibberellin 2-beta-dioxygenase 1; EC=1.14.11.13; GA 2-oxidase 1; Gibberellin 2-beta-hydroxylase 1; Gibberellin 2-oxidase 1; Protein SLENDER (At1g78440)		-2.992
Cs5g16290	K13066 caffeic acid 3-O-methyltransferase [EC:2.1.1.68] | (RefSeq) caffeic acid 3-O-methyltransferase (A)	Caffeic acid 3-O-methyltransferase; CAOMT; COMT; EC=2.1.1.68; S-adenosysl-L-methionine:caffeic acid 3-O-methyltransferase (At5g54160)		1.277

Cs5g16310	K05278 flavonol synthase [EC:1.14.11.23] | (RefSeq) flavonol synthase/flavanone 3-hydroxylase (A)	Protein DOWNY MILDEW RESISTANCE 6 {ECO:0000303|PubMed:15986928}; AtDMR6 {ECO:0000303|PubMed:15986928}; EC=1.14.11.- {ECO:0000255|PROSITE-ProRule:PRU00805}; 2-oxoglutarate (2OG)-Fe(II) oxygenase-like protein DMR6 {ECO:0000303|PubMed:18248595}; Salicylate 3-hydroxylase DMR6 {ECO:0000305}; S3H DMR6 {ECO:0000305}; SA 3-hydroxylase DMR6 {ECO:0000305}; Salicylic acid 3-hydroxylase DMR6 {ECO:0000305}; EC=1.14.13.- {ECO:0000250|UniProtKB:Q9ZSA8} (At5g24530)		-1.118
Cs5g16860	K13066 caffeic acid 3-O-methyltransferase [EC:2.1.1.68] | (RefSeq) anthranilate N-methyltransferase-like (A)	Anthranilate N-methyltransferase; RgANMT; EC=2.1.1.111 (At5g54160)	1.856	
Cs5g18050	K13066 caffeic acid 3-O-methyltransferase [EC:2.1.1.68] | (RefSeq) caffeic acid 3-O-methyltransferase-like isoform X1 (A)	Caffeic acid 3-O-methyltransferase; CAOMT; COMT; EC=2.1.1.68; S-adenosysl-L-methionine:caffeic acid 3-O-methyltransferase (At5g54160)	2.004	
Cs5g18930	K13065 shikimate O-hydroxycinnamoyltransferase [EC:2.3.1.133] | (RefSeq) shikimate O-hydroxycinnamoyltransferase-like (A)	Shikimate O-hydroxycinnamoyltransferase; EC=2.3.1.133; Hydroxycinnamoyl transferase; Hydroxycinnamoyl-Coenzyme A shikimate/quinate hydroxycinnamoyltransferase (At5g48930)	-1.479	
Cs5g19020	K13066 caffeic acid 3-O-methyltransferase [EC:2.1.1.68] | (RefSeq) hypothetical protein (A)	Caffeic acid 3-O-methyltransferase; CAOMT; COMT; EC=2.1.1.68; S-adenosysl-L-methionine:caffeic acid 3-O-methyltransferase (At5g54160)	1.778	1.136
Cs5g19060	K00696 sucrose-phosphate synthase [EC:2.4.1.14] | (RefSeq) probable sucrose-phosphate synthase 4 (A)	Probable sucrose-phosphate synthase 4; EC=2.4.1.14; Sucrose phosphate synthase 4F; AtSPS4F; UDP-glucose-fructose-phosphate glucosyltransferase (At4g10120)		1.416
Cs5g22270	K01193 beta-fructofuranosidase [EC:3.2.1.26] | (RefSeq) LOC109747879; beta-fructofuranosidase, insoluble isoenzyme 3-like (A)	UPF0481 protein At3g47200 (At4g31980_2)		1.063
Cs5g22910	K00847 fructokinase [EC:2.7.1.4] | (RefSeq) probable fructokinase-4 (A)	Probable fructokinase-4; EC=2.7.1.4 (At3g59480)	-1.093	
Cs5g23280	K00430 peroxidase [EC:1.11.1.7] | (RefSeq) hypothetical protein (A)	Probable peroxidase 61; Atperox P61; EC=1.11.1.7; Flags: Precursor (At5g24070)	-1.229	
Cs5g24450	K13065 shikimate O-hydroxycinnamoyltransferase [EC:2.3.1.133] | (RefSeq) vinorine synthase-like (A)	Vinorine synthase; EC=2.3.1.160 (At3g26040)	2.124	
Cs5g24470	K13065 shikimate O-hydroxycinnamoyltransferase [EC:2.3.1.133] |	Vinorine synthase; EC=2.3.1.160 (At3g26040)		1.489

	(RefSeq) vinorine synthase-like (A)			
Cs5g24640	K00699 glucuronosyltransferase [EC:2.4.1.17] | (RefSeq) UDP-glycosyltransferase 76C4-like (A)	UDP-glycosyltransferase 76F1; EC=2.4.1.- (At3g55700)	2.193	-1.003
Cs5g24950	K13066 caffeic acid 3-O-methyltransferase [EC:2.1.1.68] | (RefSeq) hypothetical protein (A)	Anthranilate N-methyltransferase; RgANMT; EC=2.1.1.111 (At5g54160)	1.386	1.722
Cs5g24960	K13066 caffeic acid 3-O-methyltransferase [EC:2.1.1.68] | (RefSeq) anthranilate N-methyltransferase-like (A)	Anthranilate N-methyltransferase; RgANMT; EC=2.1.1.111 (At5g54160)		2.259
Cs5g24990	K13066 caffeic acid 3-O-methyltransferase [EC:2.1.1.68] | (RefSeq) hypothetical protein (A)	Anthranilate N-methyltransferase; RgANMT; EC=2.1.1.111 (At5g54160)	2.543	
Cs5g25000	K13066 caffeic acid 3-O-methyltransferase [EC:2.1.1.68] | (RefSeq) caffeic acid 3-O-methyltransferase-like (A)	Caffeic acid 3-O-methyltransferase; CAOMT; COMT; EC=2.1.1.68; S-adenosysl-L-methionine:caffeic acid 3-O-methyltransferase (At5g54160)		1.383
Cs5g26860	K05349 beta-glucosidase [EC:3.2.1.21] | (RefSeq) beta-glucosidase BoGH3B-like (A)	Beta-D-xylosidase 3; AtBXL3; EC=3.2.1.-; Alpha-L-arabinofuranosidase; EC=3.2.1.55; Flags: Precursor (At5g20950)	2.891	2.238
Cs5g27350	K00549 5-methyltetrahydropteroyltriglutamatehomocysteine methyltransferase [EC:2.1.1.14] | (RefSeq) protein QUIRKY-like (A)	Protein QUIRKY {ECO:0000303|PubMed:19180193} (At3g03680)	-1.466	-1.678
Cs5g28310	K01723 hydroperoxide dehydratase [EC:4.2.1.92] | (RefSeq) hypothetical protein (A)	Allene oxide synthase 3 {ECO:0000303|PubMed:12351632}; LeAOS3 {ECO:0000303|PubMed:12351632}; EC=4.2.1.92 {ECO:0000269|PubMed:12351632}; Allene oxide cyclase {ECO:0000303|PubMed:12351632}; EC=5.3.99.6 {ECO:0000269|PubMed:12351632}; Cytochrome P450 CYP74C3 {ECO:0000303|PubMed:12351632}; Cytochrome P450 CYP74D1 {ECO:0000303|PubMed:17073790} (At5g42650)		2.459
Cs5g28710	K05282 gibberellin-44 dioxygenase [EC:1.14.11.12] | (RefSeq) gibberellin 20 oxidase 1-D-like (A)	Protein DMR6-LIKE OXYGENASE 1 {ECO:0000303|PubMed:25376907}; EC=1.14.11.- {ECO:0000255|PROSITE-ProRule:PRU00805}; 2-oxoglutarate (2OG)-Fe(II) oxygenase-like protein DLO1 {ECO:0000303|PubMed:25376907}; Protein SENESCENCE-ASSOCIATED GENE 108 {ECO:0000303|PubMed:23959884}; Salicylate 3-hydroxylase DLO1 {ECO:0000303|PubMed:23959884}; S3H DLO1 {ECO:0000303|PubMed:23959884}; SA 3-hydroxylase DLO1 {ECO:0000303|PubMed:23959884}; Salicylic acid 3-hydroxylase	2.636	

		DLO1 {ECO:0000303|PubMed:23959884}; EC=1.14.13.- {ECO:0000269|PubMed:23959884} (At3g19000)		
Cs5g28780	K05282 gibberellin-44 dioxygenase [EC:1.14.11.12] | (RefSeq) gibberellin 20 oxidase 1-like isoform X1 (A)	Protein DMR6-LIKE OXYGENASE 2 {ECO:0000303|PubMed:25376907}; EC=1.14.11.- {ECO:0000255|PROSITE-ProRule:PRU00805}; 2-oxoglutarate (2OG)-Fe(II) oxygenase-like protein DLO2 {ECO:0000303|PubMed:25376907}; Salicylate 3-hydroxylase DLO2 {ECO:0000305}; S3H DLO2 {ECO:0000305}; SA 3-hydroxylase DLO2 {ECO:0000305}; Salicylic acid 3-hydroxylase DLO2 {ECO:0000305}; EC=1.14.13.- {ECO:0000250|UniProtKB:Q9ZSA8} (At3g19000)		1.368
Cs5g30120	K08081 tropinone reductase I [EC:1.1.1.206] | (RefSeq) tropinone reductase homolog At5g06060-like (A)	Transcriptional regulator TAC1; Protein TELOMERASE ACTIVATOR1 (At3g09290)	1.113	
Cs5g30350	K16818 phospholipase A1 [EC:3.1.1.32] | (RefSeq) phospholipase A(1) DAD1, chloroplastic (A)	Phospholipase A1-Igamma2, chloroplastic; EC=3.1.1.-; Flags: Precursor (At1g06800)		-1.286
Cs5g32270	K00430 peroxidase [EC:1.11.1.7] | (RefSeq) peroxidase 3 (A)	Peroxidase 3; Atperox P3; EC=1.11.1.7; ATPRC; RCI3A; Rare cold-inducible protein; Flags: Precursor (At1g05260)		-1.201
Cs5g32360	K09841 xanthoxin dehydrogenase [EC:1.1.1.288] | (RefSeq) zerumbone synthase (A)	Short-chain dehydrogenase reductase 2a; AtSDR2a; EC=1.1.1.- (At4g03140)		1.272
Cs5g33470	K00695 sucrose synthase [EC:2.4.1.13] | (RefSeq) sucrose synthase 2 (A)	Sucrose synthase 2; EC=2.4.1.13; Sucrose-UDP glucosyltransferase 2 (At4g02280)		1.247
Cs5g35050	K13390 (R,S)-reticuline 7-O-methyltransferase [EC:2.1.1.291] | (RefSeq) (R,S)-reticuline 7-O-methyltransferase (A)	(R,S)-reticuline 7-O-methyltransferase {ECO:0000303|PubMed:14675446}; 7OMT {ECO:0000303|PubMed:14675446}; EC=2.1.1.291 {ECO:0000269|PubMed:14675446} (At5g54160)	2.523	
Cs5g35070	K13390 (R,S)-reticuline 7-O-methyltransferase [EC:2.1.1.291] | (RefSeq) (R,S)-reticuline 7-O-methyltransferase (A)	(R,S)-reticuline 7-O-methyltransferase {ECO:0000303|PubMed:14675446}; 7OMT {ECO:0000303|PubMed:14675446}; EC=2.1.1.291 {ECO:0000269|PubMed:14675446} (At5g54160)	1.712	1.292
Cs6g05110	K19269 phosphoglycolate phosphatase [EC:3.1.3.18 3.1.3.48] | (RefSeq) phosphoglycolate phosphatase 1B, chloroplastic (A)	Phosphoglycolate phosphatase 1B, chloroplastic; EC=3.1.3.18; Flags: Precursor (At5g36790)		-1.215
Cs6g05260	K15404 aldehyde decarbonylase [EC:4.1.99.5] | (RefSeq) hypothetical protein (A)	Protein ECERIFERUM 1 (At1g02190)	3.175	4.839
Cs6g06630	K08081 tropinone reductase I [EC:1.1.1.206] | (RefSeq) tropinone	Transcriptional regulator TAC1; Protein TELOMERASE	1.578	

	reductase homolog At5g06060-like (A)	ACTIVATOR1 (At3g09290)		
Cs6g07330	K21373 7-deoxyloganetic acid glucosyltransferase [EC:2.4.1.323] | (RefSeq) 7-deoxyloganetic acid glucosyltransferase-like (A)	7-deoxyloganetic acid glucosyltransferase; EC=2.4.1.323; UDP-glucose glucosyltransferase 8; CrUGT8; UDP-glycosyltransferase 709C2 (At1g22400)	1.060	1.522
Cs6g07920	K13508 glycerol-3-phosphate acyltransferase [EC:2.3.1.15 2.3.1.198] | (RefSeq) hypothetical protein (A)	Glycerol-3-phosphate 2-O-acyltransferase 6; AtGPAT6; EC=2.3.1.198; Glycerol-3-phosphate acyltransferase 6 (At2g38110)	1.642	4.893
Cs6g08500	K05277 leucoanthocyanidin dioxygenase [EC:1.14.11.19] | (RefSeq) leucoanthocyanidin dioxygenase-like (A)	Probable 2-oxoglutarate-dependent dioxygenase At5g05600 {ECO:0000305}; EC=1.14.11.- {ECO:0000305} (At5g05600)		-3.896
Cs6g08640	K00232 acyl-CoA oxidase [EC:1.3.3.6] | (RefSeq) acyl-coenzyme A oxidase 4, peroxisomal-like (A)	Acyl-coenzyme A oxidase 4, peroxisomal; AOX 4; EC=1.3.3.6; G6p; Short-chain acyl-CoA oxidase; AtCX4; AtG6; SAOX (At3g51840)		1.863
Cs6g08840	K01568 pyruvate decarboxylase [EC:4.1.1.1] | (RefSeq) hypothetical protein (A)	Pyruvate decarboxylase 1; AtPDC1; EC=4.1.1.1 (At4g33070)	-1.459	
Cs6g09640	K00827 alanine-glyoxylate transaminase / (R)-3-amino-2-methylpropionate-pyruvate transaminase [EC:2.6.1.44 2.6.1.40] | (RefSeq) hypothetical protein (A)	Alanineglyoxylate aminotransferase 2 homolog 3, mitochondrial; EC=2.6.1.44; Beta-alanine-pyruvate aminotransferase 3; Flags: Precursor (At3g08860)		-1.565
Cs6g09680	K00430 peroxidase [EC:1.11.1.7] | (RefSeq) cationic peroxidase 1-like (A)	Cationic peroxidase 1; EC=1.11.1.7; PNPC1; Flags: Precursor (At5g05340)		1.111
Cs6g09690	K14455 aspartate aminotransferase, mitochondrial [EC:2.6.1.1] | (RefSeq) hypothetical protein (A)	Aspartate aminotransferase, mitochondrial; EC=2.6.1.1; Transaminase A; Flags: Precursor (At2g30970)	-1.627	
Cs6g11950	K10775 phenylalanine ammonia-lyase [EC:4.3.1.24] | (RefSeq) phenylalanine ammonia-lyase (A)	Phenylalanine ammonia-lyase; EC=4.3.1.24 (At2g37040)		-1.149
Cs6g15630	K20618 cytochrome P450 family 76 subfamily A | (RefSeq) cytochrome P450 76A2-like (A)	Cytochrome P450 76A2; EC=1.14.-.-; CYPLXXVIA2; Cytochrome P-450EG7 (At3g52970)		2.087
Cs6g16650	K09754 coumaroylquinate(coumaroylshikimate) 3'-monooxygenase [EC:1.14.13.36] | (RefSeq) cytochrome P450 98A2 (A)	Cytochrome P450 98A2; EC=1.14.-.- (At2g40890)	1.097	1.588
Cs6g16980	K00059 3-oxoacyl-[acyl-carrier protein] reductase [EC:1.1.1.100] | (RefSeq) uncharacterized protein LOC112507113 (A)	3-oxoacyl-[acyl-carrier-protein] reductase, chloroplastic; EC=1.1.1.100; 3-ketoacyl-acyl carrier protein reductase; Flags: Precursor (At3g46170)		-2.084
Cs6g17140	K13065 shikimate O-hydroxycinnamoyltransferase [EC:2.3.1.133] | (RefSeq) hypothetical protein (A)	Vinorine synthase; EC=2.3.1.160 (At3g26040)		1.244
Cs6g17510	K13789 geranylgeranyl diphosphate synthase, type II [EC:2.5.1.1 2.5.1.10 2.5.1.29] | (RefSeq) geranylgeranyl pyrophosphate synthase 7, chloroplastic-like (A)	Geranylgeranyl pyrophosphate synthase, chloroplastic; GGPP synthase; GGPS; EC=2.5.1.-; (2E,6E)-farnesyl diphosphate synthase; Dimethylallyltranstransferase; EC=2.5.1.1; Farnesyl diphosphate		-6.069

		synthase; Farnesyltranstransferase; EC=2.5.1.29; Geranyltranstransferase; EC=2.5.1.10; Flags: Precursor (At4g36810)		
Cs6g17580	K14190 GDP-L-galactose phosphorylase [EC:2.7.7.69] | (RefSeq) hypothetical protein (A)	GDP-L-galactose phosphorylase 1; EC=2.7.7.69; Protein VITAMIN C DEFECTIVE 2 (At5g55120)	1.157	2.069
Cs6g17760	K21373 7-deoxyloganetic acid glucosyltransferase [EC:2.4.1.323] | (RefSeq) 7-deoxyloganetic acid glucosyltransferase-like (A)	Uncharacterized protein At1g66480 (At1g66480)		-1.615
Cs6g17850	K05350 beta-glucosidase [EC:3.2.1.21] | (RefSeq) hypothetical protein (A)	Beta-glucosidase 44; AtBGLU44; EC=3.2.1.21; Flags: Precursor (At3g18080)		-2.746
Cs6g18300	K18693 diacylglycerol diphosphate phosphatase / phosphatidate phosphatase [EC:3.1.3.81 3.1.3.4] | (RefSeq) protein PLASTID MOVEMENT IMPAIRED 2-like (A)	Protein PLASTID MOVEMENT IMPAIRED 2; Protein WEAK CHLOROPLAST MOVEMENT UNDER BLUE LIGHT 2; Protein WEB2 (At1g66840)	1.005	
Cs6g18680	K01602 ribulose-bisphosphate carboxylase small chain [EC:4.1.1.39] | (RefSeq) ribulose bisphosphate carboxylase small chain, chloroplastic-like (A)	Ribulose bisphosphate carboxylase small chain, chloroplastic; RuBisCO small subunit; EC=4.1.1.39; Flags: Precursor (At5g38410)		-1.626
Cs6g19090	K12639 cytochrome P450 family 724 subfamily B polypeptide 1 [EC:1.14.13.-] | (RefSeq) cytochrome P450 724B1-like (A)	Protein trichome birefringence-like 19 (At5g15900)		-1.594
Cs6g19760	K15889 prenylcysteine alpha-carboxyl methylesterase [EC:3.1.1.-] | (RefSeq) isoprenylcysteine alpha-carbonyl methylesterase ICME-like (A)	Major pollen allergen Lol p 11; Allergen Lol p XI; Allergen=Lol p 11 (At5g15780)		1.108
Cs6g20410	K13508 glycerol-3-phosphate acyltransferase [EC:2.3.1.15 2.3.1.198] | (RefSeq) glycerol-3-phosphate 2-O-acyltransferase 6 (A)	Glycerol-3-phosphate 2-O-acyltransferase 6; AtGPAT6; EC=2.3.1.198; Glycerol-3-phosphate acyltransferase 6 (At2g38110)		-7.533
Cs6g20950	K13066 caffeic acid 3-O-methyltransferase [EC:2.1.1.68] | (RefSeq) anthranilate N-methyltransferase-like (A)	Anthranilate N-methyltransferase; RgANMT; EC=2.1.1.111 (At5g54160)	5.384	2.730
Cs6g21370	K00703 starch synthase [EC:2.4.1.21] | (RefSeq) granule-bound starch synthase 2, chloroplastic/amyloplastic (A)	Starch synthase 2, chloroplastic/amyloplastic; AtSS2; EC=2.4.1.21; Soluble starch synthase II; Flags: Precursor (At3g01180)	1.011	
Cs7g01280	K14975 codeine 3-O-demethylase [EC:1.14.11.32] | (RefSeq) codeine O-demethylase (A)	Probable 2-oxoglutarate/Fe(II)-dependent dioxygenase; EC=1.14.11.- (At1g78550)		1.054
Cs7g01380	K01188 beta-glucosidase [EC:3.2.1.21] | (RefSeq) beta-glucosidase 17-like (A)	Beta-glucosidase 17; AtBGLU17; EC=3.2.1.21; Flags: Precursor (At2g44480)	1.090	1.679
Cs7g04930	K09841 xanthoxin dehydrogenase [EC:1.1.1.288] | (RefSeq) secoisolariciresinol dehydrogenase-like (A)	(-)-isopiperitenol/(-)-carveol dehydrogenase, mitochondrial; EC=1.1.1.223; EC=1.1.1.243; Flags: Precursor (At2g47140)		-1.401
Cs7g05690	K00696 sucrose-phosphate synthase [EC:2.4.1.14] | (RefSeq) probable sucrose-phosphate synthase 1 (A)	Probable sucrose-phosphate synthase 1; EC=2.4.1.14; UDP-glucose-fructose-phosphate glucosyltransferase 1 (At5g11110)	1.268	

Cs7g08110	K11820 N-hydroxythioamide S-beta-glucosyltransferase [EC:2.4.1.195] | (RefSeq) UDP-glycosyltransferase 74B1 (A)	UDP-glycosyltransferase 74B1; EC=2.4.1.-; N-hydroxythioamide S-beta-glucosyltransferase; EC=2.4.1.195; Thiohydroximate S-glucosyltransferase (At1g24100)	-1.156	-2.258
Cs7g08390	K16055 trehalose 6-phosphate synthase/phosphatase [EC:2.4.1.15 3.1.3.12] | (RefSeq) hypothetical protein (A)	Probable alpha,alpha-trehalose-phosphate synthase [UDP-forming] 9; EC=2.4.1.15; Trehalose-6-phosphate synthase 9; AtTPS9 (At1g23870)		-1.922
Cs7g10100	K13065 shikimate O-hydroxycinnamoyltransferase [EC:2.3.1.133] | (RefSeq) vinorine synthase (A)	Vinorine synthase; EC=2.3.1.160 (At1g24430)	2.509	4.511
Cs7g10110	K13065 shikimate O-hydroxycinnamoyltransferase [EC:2.3.1.133] | (RefSeq) BAHD acyltransferase At5g47980-like (A)	BAHD acyltransferase At5g47980; EC=2.3.1.- (At3g26040)		-1.475
Cs7g10780	K13065 shikimate O-hydroxycinnamoyltransferase [EC:2.3.1.133] | (RefSeq) shikimate O-hydroxycinnamoyltransferase-like (A)	(At1g27380)		-1.156
Cs7g11610	K13544 pheophorbidase [EC:3.1.1.82] | (RefSeq) probable pheophorbidase (A)	Putative methylesterase 13, chloroplastic; AtMES13; EC=3.1.1.-; Flags: Precursor (At3g29770)	2.136	1.689
Cs7g12080	K23260 scopoletin glucosyltransferase [EC:2.4.1.128] | (RefSeq) scopoletin glucosyltransferase-like (A)	UDP-glycosyltransferase 89A2; EC=2.4.1.- (At5g03490)		-1.248
Cs7g12370	K00430 peroxidase [EC:1.11.1.7] | (RefSeq) peroxidase 11 (A)	Peroxidase 11; Atperox P11; EC=1.11.1.7; ATP23a/ATP23b; Flags: Precursor (At1g68850)	1.028	4.241
Cs7g13290	K00993 ethanolaminephosphotransferase [EC:2.7.8.1] | (RefSeq) uncharacterized protein LOC106453259 (A)	(At1g13480)	1.161	1.506
Cs7g13310	K15397 3-ketoacyl-CoA synthase [EC:2.3.1.199] | (RefSeq) hypothetical protein (A)	3-ketoacyl-CoA synthase 6 {ECO:0000303|PubMed:18465198}; KCS-6 {ECO:0000303|PubMed:18465198}; EC=2.3.1.199 {ECO:0000269|PubMed:10330468}; Cuticular protein 1 {ECO:0000303|PubMed:10330468}; Eceriferum 6 {ECO:0000303|PubMed:11041893}; Very long-chain fatty acid condensing enzyme 6 {ECO:0000303|PubMed:18465198}; VLCFA condensing enzyme 6 {ECO:0000303|PubMed:18465198} (At1g68530)	3.174	6.706
Cs7g13320	K00547 homocysteine S-methyltransferase [EC:2.1.1.10] | (RefSeq) hypothetical protein (A)	Homocysteine S-methyltransferase 1; BoHMT1; EC=2.1.1.10 (At3g25900)		1.047
Cs7g14420	K00121 S-(hydroxymethyl)glutathione dehydrogenase / alcohol dehydrogenase [EC:1.1.1.284 1.1.1.1] | (RefSeq) alcohol dehydrogenase-like 2 (A)	Alcohol dehydrogenase-like 2; EC=1.1.1.1 {ECO:0000250|UniProtKB:P06525} (At1g22440)		1.229
Cs7g14940	K04125 gibberellin 2-oxidase [EC:1.14.11.13] | (RefSeq) gibberellin	Gibberellin 2-beta-dioxygenase 8; EC=1.14.11.13; GA 2-oxidase 8;		1.683

	2-beta-dioxygenase 8-like (A)	Gibberellin 2-beta-hydroxylase 8; Gibberellin 2-oxidase 8 (At4g21200)		
Cs7g15500	K07409 cytochrome P450 family 1 subfamily A polypeptide 2 [EC:1.14.14.1] | (RefSeq) cytochrome P450 89A2-like (A)	Cytochrome P450 89A2; EC=1.14.-.-; ATH 6-1; CYPLXXXIX (At1g64940)	1.947	
Cs7g15540	K07409 cytochrome P450 family 1 subfamily A polypeptide 2 [EC:1.14.14.1] | (RefSeq) cytochrome P450 89A2-like (A)	Cytochrome P450 89A2; EC=1.14.-.-; ATH 6-1; CYPLXXXIX (At1g64940)		1.202
Cs7g15620	K01114 phospholipase C [EC:3.1.4.3] | (RefSeq) non-specific phospholipase C4-like (A)	Non-specific phospholipase C4; EC=3.1.4.3 (At3g03530)	1.786	2.198
Cs7g17090	K13508 glycerol-3-phosphate acyltransferase [EC:2.3.1.15 2.3.1.198] | (RefSeq) glycerol-3-phosphate acyltransferase 1 (A)	Glycerol-3-phosphate acyltransferase 1; AtGPAT1; EC=2.3.1.15 (At1g06520)		1.529
Cs7g17370	K09833 homogentisate phytyltransferase / homogentisate geranylgeranyltransferase [EC:2.5.1.115 2.5.1.116] | (RefSeq) homogentisate phytyltransferase 1, chloroplastic-like (A)	Coumarin 8-geranyltransferase 1, chloroplastic; EC=2.5.1.138 {ECO:0000269|PubMed:25077796}; Prenyltransferase 1 {ECO:0000303|PubMed:25077796}; ClPT1 {ECO:0000303|PubMed:25077796}; Umbelliferone 8-C-geranyltransferase {ECO:0000303|PubMed:25077796}; U8GT {ECO:0000303|PubMed:25077796}; Flags: Precursor		2.169
Cs7g17390	K09833 homogentisate phytyltransferase / homogentisate geranylgeranyltransferase [EC:2.5.1.115 2.5.1.116] | (RefSeq) homogentisate phytyltransferase 1, chloroplastic-like (A)	Coumarin 8-geranyltransferase 1b, chloroplastic; EC=2.5.1.138 {ECO:0000269|PubMed:25077796}; Prenyltransferase 1b {ECO:0000303|PubMed:25077796}; ClPT1ba {ECO:0000303|PubMed:25077796}; Flags: Precursor (At2g18950)	2.268	1.567
Cs7g17410	K12153 phenylalanine N-monooxygenase [EC:1.14.14.40] | (RefSeq) phenylalanine N-monooxygenase-like (A)	Phenylalanine N-monooxygenase; EC=1.14.14.40 {ECO:0000269|PubMed:10799553}; Cytochrome P450 79A2; Phenylalanine N-hydroxylase (At5g05260)	9.372	
Cs7g19710	K03428 magnesium-protoporphyrin O-methyltransferase [EC:2.1.1.11] | (RefSeq) hypothetical protein (A)	Magnesium protoporphyrin IX methyltransferase, chloroplastic; EC=2.1.1.11; Flags: Precursor (At4g25080)	-1.203	-1.249
Cs7g20830	K01593 aromatic-L-amino-acid/L-tryptophan decarboxylase [EC:4.1.1.28 4.1.1.105] | (RefSeq) tyrosine/DOPA decarboxylase (A)	(At5g01130)	1.043	
Cs7g21790	K10526 OPC-8:0 CoA ligase 1 [EC:6.2.1.-] | (RefSeq) 4-coumarateCoA ligase-like 5 (A)	4-coumarateCoA ligase-like 5; EC=6.2.1.-; 4-coumarateCoA ligase isoform 9; At4CL9; Peroxisomal OPC-8:0-CoA ligase 1 (At1g20510)	1.656	-2.401
Cs7g22860	K02437 glycine cleavage system H protein | (RefSeq) hypothetical protein (A)	Glycine cleavage system H protein, mitochondrial; Flags: Precursor (At1g32470)		-1.360
Cs7g23850	K07024 sucrose-6-phosphatase [EC:3.1.3.24] | (RefSeq) hypothetical protein (A)	Sucrose-phosphatase 2; NtSPP2; EC=3.1.3.24 (At1g51420)		1.006

Cs7g24920	K00615 transketolase [EC:2.2.1.1] | (RefSeq) transketolase, chloroplastic-like (A)	Transketolase-1, chloroplastic; TK; EC=2.2.1.1; Flags: Precursor (At2g45290)	1.474	-1.189
Cs7g25010	K13066 caffeic acid 3-O-methyltransferase [EC:2.1.1.68] | (RefSeq) COMT1-2; caffeic acid O-methyltransferase (A)	Caffeic acid 3-O-methyltransferase; CAOMT; COMT; EC=2.1.1.68; S-adenosysl-L-methionine:caffeic acid 3-O-methyltransferase (At3g53140)		-1.955
Cs7g25030	K10775 phenylalanine ammonia-lyase [EC:4.3.1.24] | (RefSeq) phenylalanine ammonia-lyase (A)	Phenylalanine ammonia-lyase; EC=4.3.1.24; Flags: Fragment (At3g53260)		-2.319
Cs7g25540	K13066 caffeic acid 3-O-methyltransferase [EC:2.1.1.68] | (RefSeq) COMT1-2; caffeic acid O-methyltransferase (A)	Caffeic acid 3-O-methyltransferase; CAOMT; COMT; EC=2.1.1.68; S-adenosysl-L-methionine:caffeic acid 3-O-methyltransferase (At3g53140)	-1.256	-1.457
Cs7g25580	K13066 caffeic acid 3-O-methyltransferase [EC:2.1.1.68] | (RefSeq) caffeic acid 3-O-methyltransferase-like (A)	Caffeic acid 3-O-methyltransferase; CAOMT; COMT; EC=2.1.1.68; S-adenosysl-L-methionine:caffeic acid 3-O-methyltransferase (At5g54160)	4.994	
Cs7g28480	K01593 aromatic-L-amino-acid/L-tryptophan decarboxylase [EC:4.1.1.28 4.1.1.105] | (RefSeq) tyrosine/DOPA decarboxylase (A)	(At5g01150)	1.596	
Cs7g28520	K01593 aromatic-L-amino-acid/L-tryptophan decarboxylase [EC:4.1.1.28 4.1.1.105] | (RefSeq) tyrosine/DOPA decarboxylase (A)	(At5g01150)	1.708	
Cs7g29080	K13065 shikimate O-hydroxycinnamoyltransferase [EC:2.3.1.133] | (RefSeq) shikimate O-hydroxycinnamoyltransferase-like (A)	Shikimate O-hydroxycinnamoyltransferase; EC=2.3.1.133; Hydroxycinnamoyl transferase; Hydroxycinnamoyl-Coenzyme A shikimate/quinate hydroxycinnamoyltransferase (At5g48930)		-1.346
Cs7g29470	K13544 pheophorbidase [EC:3.1.1.82] | (RefSeq) pheophorbidase (A)	Methylesterase 17; AtMES17; EC=3.1.1.-; Methyl indole-3-acetic acid esterase (At3g10870)		1.465
Cs7g29760	K12153 phenylalanine N-monooxygenase [EC:1.14.14.40] | (RefSeq) hypothetical protein (A)	Phenylalanine N-monooxygenase; EC=1.14.14.40 {ECO:0000269|PubMed:10799553}; Cytochrome P450 79A2; Phenylalanine N-hydroxylase (At5g05260)		1.264
Cs7g29780	K01859 chalcone isomerase [EC:5.5.1.6] | (RefSeq) probable chalconeflavonone isomerase 3 (A)	Probable chalconeflavonone isomerase 3; Chalcone isomerase 3; EC=5.5.1.6; Chalcone isomerase-like 1 (At5g05270)		-1.305
Cs7g31010	K05277 leucoanthocyanidin dioxygenase [EC:1.14.11.19] | (RefSeq) leucoanthocyanidin dioxygenase (A)	Probable 2-oxoglutarate-dependent dioxygenase At5g05600 {ECO:0000305}; EC=1.14.11.- {ECO:0000305} (At5g05600)		-1.142
Cs7g31730	K13508 glycerol-3-phosphate acyltransferase [EC:2.3.1.15 2.3.1.198] | (RefSeq) hypothetical protein (A)	Glycerol-3-phosphate acyltransferase 7; AtGPAT7; EC=2.3.1.15 (At5g06090)	1.020	4.530
Cs7g31860	K08081 tropinone reductase I [EC:1.1.1.206] | (RefSeq) tropinone reductase homolog At5g06060-like (A)	Probable transcriptional regulator RABBIT EARS (At2g37740)		

Cs7g32210	K00699 glucuronosyltransferase [EC:2.4.1.17] | (RefSeq) UDP-glycosyltransferase 76C4-like (A)	UDP-glycosyltransferase 76F1; EC=2.4.1.- (At3g55700)		-2.226
Cs7g32220	K00699 glucuronosyltransferase [EC:2.4.1.17] | (RefSeq) UDP-glycosyltransferase 76G1-like (A)	UDP-glycosyltransferase 76B1 {ECO:0000305}; EC=2.4.1.- {ECO:0000305} (At3g11340)		-2.117
Cs8g01250	K14677 aminoacylase [EC:3.5.1.14] | (RefSeq) hypothetical protein (A)	(At4g38220)	1.180	
Cs8g02970	K00025 malate dehydrogenase [EC:1.1.1.37] | (RefSeq) malate dehydrogenase, cytoplasmic-like (A)	Oligopeptide transporter 4; AtOPT4 (At5g64410)	-1.428	
Cs8g03410	K00826 branched-chain amino acid aminotransferase [EC:2.6.1.42] | (RefSeq) hypothetical protein (A)	Branched-chain-amino-acid aminotransferase 2, chloroplastic; Atbcat-2; EC=2.6.1.42; Flags: Precursor (At1g10070)	1.282	
Cs8g03530	K00511 squalene monooxygenase [EC:1.14.14.17] | (RefSeq) squalene monooxygenase-like (A)	Squalene monooxygenase; EC=1.14.14.17 {ECO:0000250|UniProtKB:Q9SM02}; Squalene epoxidase; SE (At1g58440)		1.822
Cs8g03950	K05278 flavonol synthase [EC:1.14.11.23] | (RefSeq) flavonol synthase/flavanone 3-hydroxylase-like (A)	Protein SRG1; AtSRG1; Protein SENESCENCE-RELATED GENE 1 (At1g49390)		-1.217
Cs8g05280	K23260 scopoletin glucosyltransferase [EC:2.4.1.128] | (RefSeq) scopoletin glucosyltransferase-like (A)	Scopoletin glucosyltransferase; EC=2.4.1.128; Phenylpropanoid:glucosyltransferase 1 (At2g15480)	2.210	1.208
Cs8g05300	K23260 scopoletin glucosyltransferase [EC:2.4.1.128] | (RefSeq) scopoletin glucosyltransferase (A)	Scopoletin glucosyltransferase; EC=2.4.1.128; Phenylpropanoid:glucosyltransferase 1 (At2g15480)	1.013	
Cs8g06110	K00940 nucleoside-diphosphate kinase [EC:2.7.4.6] | (RefSeq) nucleoside diphosphate kinase 1-like (A)	Nucleoside diphosphate kinase 1; EC=2.7.4.6; Nucleoside diphosphate kinase I; NDK I; NDP kinase I; NDPK I (At4g09320)	-1.926	-1.763
Cs8g06970	K13513 lysocardiolipin and lysophospholipid acyltransferase [EC:2.3.1.- 2.3.1.51] | (RefSeq) probable 1-acyl-sn-glycerol-3-phosphate acyltransferase 5 (A)	Probable 1-acyl-sn-glycerol-3-phosphate acyltransferase 5; EC=2.3.1.51; Lysophosphatidyl acyltransferase 5 (At3g18850)	-1.227	
Cs8g07030	K03527 4-hydroxy-3-methylbut-2-en-1-yl diphosphate reductase [EC:1.17.7.4] | (RefSeq) 4-hydroxy-3-methylbut-2-enyl diphosphate reductase, chloroplastic-like isoform X1 (A)	4-hydroxy-3-methylbut-2-enyl diphosphate reductase, chloroplastic; EC=1.17.7.4; Flags: Precursor (At4g34350)		-1.163
Cs8g07730	K00764 amidophosphoribosyltransferase [EC:2.4.2.14] | (RefSeq) amidophosphoribosyltransferase, chloroplastic-like (A)	Amidophosphoribosyltransferase 2, chloroplastic; AtATase2; AtPURF2; PRPP2; EC=2.4.2.14; Glutamine phosphoribosylpyrophosphate amidotransferase 2; AtGPRAT2; Protein CHLOROPLAST IMPORT APPARATUS 1; Protein DIFFERENTIAL DEVELOPMENT OF VASCULAR ASSOCIATED CELLS; Flags: Precursor (At4g34740)	1.316	
Cs8g08710	K01623 fructose-bisphosphate aldolase, class I [EC:4.1.2.13] |	Fructose-bisphosphate aldolase 2, chloroplastic {ECO:0000305};	1.927	

	(RefSeq) fructose-bisphosphate aldolase 1, chloroplastic (A)	AtFBA2 {ECO:0000303|PubMed:22561114}; EC=4.1.2.13 {ECO:0000305}; Flags: Precursor (At2g01140)		
Cs8g09790	K13265 vestitone reductase [EC:1.1.1.348] | (RefSeq) vestitone reductase (A)	Vestitone reductase; EC=1.1.1.348 (At2g45400)		3.372
Cs8g09800	K00660 chalcone synthase [EC:2.3.1.74] | (RefSeq) chalcone synthase 2-like (A)	Chalcone synthase 2; EC=2.3.1.74; Naringenin-chalcone synthase 2 (At5g13930)		2.197
Cs8g09830	K00660 chalcone synthase [EC:2.3.1.74] | (RefSeq) chalcone synthase 2-like (A)	Chalcone synthase 2; EC=2.3.1.74; Naringenin-chalcone synthase 2 (At5g13930)		1.133
Cs8g11370	K01193 beta-fructofuranosidase [EC:3.2.1.26] | (RefSeq) LOC109747879; beta-fructofuranosidase, insoluble isoenzyme 3-like (A)	UPF0481 protein At3g47200 (At4g31980_2)	2.993	2.516
Cs8g11500	K01193 beta-fructofuranosidase [EC:3.2.1.26] | (RefSeq) LOC109747879; beta-fructofuranosidase, insoluble isoenzyme 3-like (A)	UPF0481 protein At3g47200 (At4g31980_2)	1.141	
Cs8g12640	K01620 threonine aldolase [EC:4.1.2.48] | (RefSeq) probable low-specificity L-threonine aldolase 1 (A)	Probable low-specificity L-threonine aldolase 1; EC=4.1.2.48; Threonine aldolase 1 (At1g08630)	1.132	
Cs8g13100	K13035 beta-cyano-L-alanine hydratase/nitrilase [EC:3.5.5.4 4.2.1.65] | (RefSeq) bifunctional nitrilase/nitrile hydratase NIT4A-like (A)	Bifunctional nitrilase/nitrile hydratase NIT4A; LaNIT4A; EC=3.5.5.4; EC=4.2.1.65; 3-cyanoalanine hydratase; Cyanoalanine nitrilase A (At5g22300)		1.336
Cs8g13770	K09842 abscisic-aldehyde oxidase [EC:1.2.3.14] | (RefSeq) indole-3-acetaldehyde oxidase-like (A)	Indole-3-acetaldehyde oxidase; IAA oxidase; EC=1.2.3.7; Aldehyde oxidase 1; AO-1; AtAO-1; AtAO1 (At5g20960)		1.072
Cs8g13780	K09842 abscisic-aldehyde oxidase [EC:1.2.3.14] | (RefSeq) abscisic-aldehyde oxidase-like (A)	Abscisic-aldehyde oxidase; EC=1.2.3.14; Aldehyde oxidase 3; AO-3; AtAO-3; AtAO4; Indole-3-acetaldehyde oxidase; IAA oxidase; EC=1.2.3.7 (At2g27150)		1.068
Cs8g13800	K05349 beta-glucosidase [EC:3.2.1.21] | (RefSeq) beta-glucosidase BoGH3B-like (A)	Beta-xylosidase/alpha-L-arabinofuranosidase 2 {ECO:0000303|PubMed:17615411, ECO:0000312|EMBL:ABQ45228.1}; Xylan 1,4-beta-xylosidase/Alpha-L-arabinofuranosidase 2; MsXyl2 {ECO:0000303|PubMed:17615411}; Includes: Beta-xylosidase; EC=3.2.1.37; 1,4-beta-D-xylan xylohydrolase {ECO:0000250|UniProtKB:P48792}; Xylan 1,4-beta-xylosidase; Includes: Alpha-L-arabinofuranosidase; Arabinosidase {ECO:0000250|UniProtKB:P48792}; EC=3.2.1.55; Flags: Precursor (At5g20950)		-1.094

Cs8g15140	K22395 cinnamyl-alcohol dehydrogenase [EC:1.1.1.195] | (RefSeq) tetrahydrocannabinolic acid synthase-like (A)	Berberine bridge enzyme-like 5 {ECO:0000303|PubMed:26037923}; AtBBE-like 5 {ECO:0000303|PubMed:26037923}; EC=1.1.1.- {ECO:0000250|UniProtKB:O64743}; Flavin-dependent oxidoreductase FOX3 {ECO:0000303|PubMed:26352477}; EC=1.-.-.- {ECO:0000305}; Flags: Precursor (At1g26400)		-3.348
Cs8g16290	K10775 phenylalanine ammonia-lyase [EC:4.3.1.24] | (RefSeq) phenylalanine ammonia-lyase-like (A)	Phenylalanine ammonia-lyase 1; EC=4.3.1.24 (At2g37040)		2.899
Cs8g18300	K00699 glucuronosyltransferase [EC:2.4.1.17] | (RefSeq) UDP-glycosyltransferase 76G1-like (A)	UDP-glycosyltransferase 76B1 {ECO:0000305}; EC=2.4.1.- {ECO:0000305} (At3g11340)	-1.264	-1.334
Cs8g18310	K00699 glucuronosyltransferase [EC:2.4.1.17] | (RefSeq) UDP-glycosyltransferase 76G1-like (A)	UDP-glycosyltransferase 76B1 {ECO:0000305}; EC=2.4.1.- {ECO:0000305} (At3g11340)	-1.040	-3.150
Cs8g19060	K08081 tropinone reductase I [EC:1.1.1.206] | (RefSeq) hypothetical protein (A)	Tropinone reductase homolog At2g29170 {ECO:0000305}; EC=1.1.1.- {ECO:0000305} (At2g29170)	1.296	1.801
Cs8g19070	K08081 tropinone reductase I [EC:1.1.1.206] | (RefSeq) tropinone reductase homolog At2g29170-like (A)	Tropinone reductase homolog At2g29170 {ECO:0000305}; EC=1.1.1.- {ECO:0000305} (At2g29170)		-1.055
Cs8g19105	K08081 tropinone reductase I [EC:1.1.1.206] | (RefSeq) tropinone reductase homolog At2g29170-like (A)	Tropinone reductase homolog At2g29170 {ECO:0000305}; EC=1.1.1.- {ECO:0000305} (At2g29170)	1.310	1.922
Cs8g19110	K08081 tropinone reductase I [EC:1.1.1.206] | (RefSeq) hypothetical protein (A)	Senescence-associated protein 13 {ECO:0000303|PubMed:9617813}; Tropinone reductase homolog SAG13 {ECO:0000250|UniProtKB:P50162}; EC=1.1.1.- {ECO:0000305} (At2g29350)		-2.568
Cs8g19800	K00688 glycogen phosphorylase [EC:2.4.1.1] | (RefSeq) hypothetical protein (A)	Alpha-glucan phosphorylase 2, cytosolic; AtPHS2; EC=2.4.1.1; Alpha-glucan phosphorylase, H isozyme; Starch phosphorylase H (At3g46970)		1.288
Cs8g20110	K11778 ditrans,polycis-polyprenyl diphosphate synthase [EC:2.5.1.87] | (RefSeq) dehydrodolichyl diphosphate synthase 2 (A)	Dehydrodolichyl diphosphate synthase 2; Dedol-PP synthase 2; EC=2.5.1.- (At5g58770)	-1.423	
Cs8g20390	K00232 acyl-CoA oxidase [EC:1.3.3.6] | (RefSeq) uncharacterized protein LOC107952742 isoform X1 (A)	Increased DNA methylation 1 {ECO:0000303|PubMed:22700931}; Histone H3 acetyltransferase IDM1 {ECO:0000305}; EC=2.3.1.- {ECO:0000255|PROSITE-ProRule:PRU00532}; Protein ROS4 {ECO:0000303|PubMed:22733760}; Repressor of silencing 4 {ECO:0000303|PubMed:22733760} (At5g58610_2)		1.155
Cs8g20950	K15813 beta-amyrin synthase [EC:5.4.99.39] | (RefSeq) beta-amyrin synthase-like (A)	Beta-amyrin synthase; EC=5.4.99.39 (At1g78950)	2.931	3.980

Cs9g01770	K04125 gibberellin 2-oxidase [EC:1.14.11.13] | (RefSeq) gibberellin 2-beta-dioxygenase 2-like (A)	Gibberellin 2-beta-dioxygenase 2; EC=1.14.11.13; GA 2-oxidase 2; Gibberellin 2-beta-hydroxylase 2; Gibberellin 2-oxidase 2 (At1g47990)		1.084
Cs9g02540	K01913 acetate-CoA ligase [EC:6.2.1.1] | (RefSeq) acetate/butyrateCoA ligase AAE7, peroxisomal-like (A)	Acetate/butyrateCoA ligase AAE7, peroxisomal; EC=6.2.1.1; EC=6.2.1.2; AMP-binding protein 7; AtAMPBP7; Acetyl-CoA synthetase; Acyl-activating enzyme 7; Butyryl-CoA synthetase; Protein ACETATE NON-UTILIZING 1 (At3g16910)		-1.628
Cs9g02550	K01913 acetate-CoA ligase [EC:6.2.1.1] | (RefSeq) hypothetical protein (A)	Acetate/butyrateCoA ligase AAE7, peroxisomal; EC=6.2.1.1; EC=6.2.1.2; AMP-binding protein 7; AtAMPBP7; Acetyl-CoA synthetase; Acyl-activating enzyme 7; Butyryl-CoA synthetase; Protein ACETATE NON-UTILIZING 1 (At3g16910)	1.479	
Cs9g02930	K06892 feruloyl-CoA ortho-hydroxylase [EC:1.14.11.-] | (RefSeq) feruloyl CoA ortho-hydroxylase 1 (A)	Feruloyl CoA ortho-hydroxylase 1; EC=1.14.11.- (At3g13610)	2.493	1.665
Cs9g02990	K13071 pheophorbide a oxygenase [EC:1.14.15.17] | (RefSeq) pheophorbide a oxygenase, chloroplastic (A)	Protochlorophyllide-dependent translocon component 52, chloroplastic; ACD1-like protein; Protein TIC 55-IV; Translocon at the inner envelope membrane of chloroplasts 55-IV; Flags: Precursor (At4g25650)		-1.328
Cs9g04000	K00249 acyl-CoA dehydrogenase [EC:1.3.8.7] | (RefSeq) probable acyl-CoA dehydrogenase IBR3 (A)	Probable acyl-CoA dehydrogenase IBR3 {ECO:0000305}; EC=1.3.99.- {ECO:0000305}; Protein INDOLE-3-BUTYRIC ACID RESPONSE 3 {ECO:0000303|PubMed:17277896} (At3g06810)		1.524
Cs9g04980	K01177 beta-amylase [EC:3.2.1.2] | (RefSeq) inactive beta-amylase 9-like isoform X1 (A)	Inactive beta-amylase 9; 1,4-alpha-D-glucan maltohydrolase; Inactive beta-amylase 3 (At5g18670)		-1.594
Cs9g05130	K00430 peroxidase [EC:1.11.1.7] | (RefSeq) peroxidase 3-like (A)	Peroxidase 3; Atperox P3; EC=1.11.1.7; ATPRC; RCI3A; Rare cold-inducible protein; Flags: Precursor (At1g05260)		1.261
Cs9g05210	K07513 acetyl-CoA acyltransferase 1 [EC:2.3.1.16] | (RefSeq) hypothetical protein (A)	3-ketoacyl-CoA thiolase 2, peroxisomal; EC=2.3.1.16; Acetyl-CoA acyltransferase 2; Beta-ketothiolase 2; Peroxisomal 3-oxoacyl-CoA thiolase 2; Peroxisome defective protein 1; Flags: Precursor (At2g33150)	-1.826	
Cs9g06010	K10760 adenylate dimethylallyltransferase (cytokinin synthase) [EC:2.5.1.27 2.5.1.112] | (RefSeq) hypothetical protein (A)	Adenylate isopentenyltransferase 5, chloroplastic; AtIPT5; EC=2.5.1.112; Adenylate dimethylallyltransferase 5; Cytokinin synthase 5; Flags: Precursor (At5g19040)	1.269	2.713
Cs9g06450	K13258 2-hydroxyisoflavanone dehydratase [EC:4.2.1.105] | (RefSeq) 2-hydroxyisoflavanone dehydratase-like (A)	2-hydroxyisoflavanone dehydratase; EC=3.1.1.1; EC=4.2.1.105; Carboxylesterase HIDM (At1g47480)		1.646
Cs9g06490	K13258 2-hydroxyisoflavanone dehydratase [EC:4.2.1.105] |	2-hydroxyisoflavanone dehydratase; EC=3.1.1.1; EC=4.2.1.105;	1.520	1.830

	(RefSeq) 2-hydroxyisoflavanone dehydratase (A)	Carboxylesterase HIDM (At3g48690)		
Cs9g06700	K00276 primary-amine oxidase [EC:1.4.3.21] | (RefSeq) hypothetical protein (A)	Primary amine oxidase 2; EC=1.4.3.21 {ECO:0000250|UniProtKB:P12807}; Amine oxidase [copper-containing] 2; Flags: Precursor (At1g31670)	7.325	
Cs9g06710	K00276 primary-amine oxidase [EC:1.4.3.21] | (RefSeq) hypothetical protein (A)	Primary amine oxidase; EC=1.4.3.21 {ECO:0000250|UniProtKB:P46883}; Amine oxidase [copper-containing]; Flags: Precursor; Fragment (At1g31690)	5.304	
Cs9g06770	K00276 primary-amine oxidase [EC:1.4.3.21] | (RefSeq) hypothetical protein (A)	Primary amine oxidase; EC=1.4.3.21 {ECO:0000250|UniProtKB:P46883}; Amine oxidase [copper-containing]; Flags: Precursor; Fragment (At1g31700)	1.992	
Cs9g07420	K00547 homocysteine S-methyltransferase [EC:2.1.1.10] | (RefSeq) homocysteine S-methyltransferase 3 (A)	Homocysteine S-methyltransferase 3; EC=2.1.1.10; S-methylmethionine:homocysteine methyltransferase 3; AtHMT-3; SMM:Hcy S-methyltransferase 3 (At3g22740)	1.233	
Cs9g07780	K13811 3'-phosphoadenosine 5'-phosphosulfate synthase [EC:2.7.7.4 2.7.1.25] | (RefSeq) hypothetical protein (A)	ATP sulfurylase 1, chloroplastic; AtPS1; EC=2.7.7.4; Flags: Precursor (At3g22890)	-1.997	-1.224
Cs9g07970	K15397 3-ketoacyl-CoA synthase [EC:2.3.1.199] | (RefSeq) 3-ketoacyl-CoA synthase 11-like (A)	3-ketoacyl-CoA synthase 2 {ECO:0000303|PubMed:18465198}; KCS-2 {ECO:0000303|PubMed:18465198}; EC=2.3.1.199 {ECO:0000305}; Docosanoic acid synthase {ECO:0000303|PubMed:18786002}; Very long-chain fatty acid condensing enzyme 2 {ECO:0000303|PubMed:18465198}; VLCFA condensing enzyme 2 {ECO:0000303|PubMed:18465198} (At1g04220)	1.453	4.773
Cs9g08970	K13065 shikimate O-hydroxycinnamoyltransferase [EC:2.3.1.133] | (RefSeq) salutaridinol 7-O-acetyltransferase-like (A)	Salutaridinol 7-O-acetyltransferase {ECO:0000303|PubMed:11404355}; salAT {ECO:0000303|PubMed:11404355}; EC=2.3.1.150 {ECO:0000269|PubMed:11404355, ECO:0000269|PubMed:8537369} (At3g26040)		1.363
Cs9g10010	K05359 arogenate/prephenate dehydratase [EC:4.2.1.91 4.2.1.51] | (RefSeq) arogenate dehydratase/prephenate dehydratase 1, chloroplastic-like (A)	Arogenate dehydratase/prephenate dehydratase 1, chloroplastic; AtADT1; AtPDT1; EC=4.2.1.51; EC=4.2.1.91; Flags: Precursor (At1g11790)	1.603	2.050
Cs9g11190	K00660 chalcone synthase [EC:2.3.1.74] | (RefSeq) chalcone synthase 1 (A)	Chalcone synthase 1; EC=2.3.1.74; Naringenin-chalcone synthase 1 (At5g13930)		-1.384
Cs9g11250	K07513 acetyl-CoA acyltransferase 1 [EC:2.3.1.16] | (RefSeq) hypothetical protein (A)	3-ketoacyl-CoA thiolase 2, peroxisomal; EC=2.3.1.16; Acetyl-CoA acyltransferase 2; Beta-ketothiolase 2; Peroxisomal 3-oxoacyl-CoA		

		thiolase 2; Peroxisome defective protein 1; Flags: Precursor (At2g33150)		
Cs9g14300	K16055 trehalose 6-phosphate synthase/phosphatase [EC:2.4.1.15 3.1.3.12] | (RefSeq) alpha,alpha-trehalose-phosphate synthase [UDP-forming] 6 (A)	Alpha,alpha-trehalose-phosphate synthase [UDP-forming] 6; EC=2.4.1.15; Trehalose-6-phosphate synthase 6; AtTPS6 (At1g68020)		-1.578
Cs9g14480	K00475 naringenin 3-dioxygenase [EC:1.14.11.9] | (RefSeq) naringenin,2-oxoglutarate 3-dioxygenase (A)	Protein DMR6-LIKE OXYGENASE 2 {ECO:0000303|PubMed:25376907}; EC=1.14.11.- {ECO:0000255|PROSITE-ProRule:PRU00805}; 2-oxoglutarate (2OG)-Fe(II) oxygenase-like protein DLO2 {ECO:0000303|PubMed:25376907}; Salicylate 3-hydroxylase DLO2 {ECO:0000305}; S3H DLO2 {ECO:0000305}; SA 3-hydroxylase DLO2 {ECO:0000305}; Salicylic acid 3-hydroxylase DLO2 {ECO:0000305}; EC=1.14.13.- {ECO:0000250|UniProtKB:Q9ZSA8} (At4g10490)	4.517	
Cs9g14500	K06892 feruloyl-CoA ortho-hydroxylase [EC:1.14.11.-] | (RefSeq) feruloyl CoA ortho-hydroxylase 1 (A)	Protein DMR6-LIKE OXYGENASE 2 {ECO:0000303|PubMed:25376907}; EC=1.14.11.- {ECO:0000255|PROSITE-ProRule:PRU00805}; 2-oxoglutarate (2OG)-Fe(II) oxygenase-like protein DLO2 {ECO:0000303|PubMed:25376907}; Salicylate 3-hydroxylase DLO2 {ECO:0000305}; S3H DLO2 {ECO:0000305}; SA 3-hydroxylase DLO2 {ECO:0000305}; Salicylic acid 3-hydroxylase DLO2 {ECO:0000305}; EC=1.14.13.- {ECO:0000250|UniProtKB:Q9ZSA8} (At4g10490)	5.040	
Cs9g14520	K05278 flavonol synthase [EC:1.14.11.23] | (RefSeq) flavonol synthase/flavanone 3-hydroxylase (A)	Protein DMR6-LIKE OXYGENASE 2 {ECO:0000303|PubMed:25376907}; EC=1.14.11.- {ECO:0000255|PROSITE-ProRule:PRU00805}; 2-oxoglutarate (2OG)-Fe(II) oxygenase-like protein DLO2 {ECO:0000303|PubMed:25376907}; Salicylate 3-hydroxylase DLO2 {ECO:0000305}; S3H DLO2 {ECO:0000305}; SA 3-hydroxylase DLO2 {ECO:0000305}; Salicylic acid 3-hydroxylase DLO2 {ECO:0000305}; EC=1.14.13.- {ECO:0000250|UniProtKB:Q9ZSA8} (At4g10490)	6.224	
Cs9g14590	K01193 beta-fructofuranosidase [EC:3.2.1.26] | (RefSeq) Cs-bFruct2; beta-fructofuranosidase, soluble isoenzyme I-like (A)	Beta-fructofuranosidase, soluble isoenzyme I; EC=3.2.1.26; Invertase; Saccharase; Sucrose hydrolase; Flags: Precursor		-2.684

		(At1g12240)		
Cs9g14600	K01087 trehalose 6-phosphate phosphatase [EC:3.1.3.12] | (RefSeq) trehalose-phosphate phosphatase A (A)	Trehalose-phosphate phosphatase A; AtTPPA; EC=3.1.3.12; Trehalose 6-phosphate phosphatase (At5g51460)		1.012
Cs9g15750	K22395 cinnamyl-alcohol dehydrogenase [EC:1.1.1.195] | (RefSeq) tetrahydrocannabinolic acid synthase-like (A)	Berberine bridge enzyme-like 15 {ECO:0000303|PubMed:26037923}; AtBBE-like 15 {ECO:0000303|PubMed:26037923}; EC=1.1.1.194 {ECO:0000269|PubMed:26037923}; EC=1.1.1.195 {ECO:0000269|PubMed:26037923}; Protein EMBRYO SAC DEVELOPMENT ARREST 28 {ECO:0000303|PubMed:15634699}; Protein MATERNAL EFFECT EMBRYO ARREST 23 {ECO:0000303|PubMed:15634699}; Flags: Precursor (At2g34790)	2.824	4.932
Cs9g15760	K22395 cinnamyl-alcohol dehydrogenase [EC:1.1.1.195] | (RefSeq) flavin-dependent oxidoreductase FOX2-like (A)	Berberine bridge enzyme-like 13 {ECO:0000303|PubMed:26037923}; AtBBE-like 13 {ECO:0000303|PubMed:26037923}; EC=1.1.1.194 {ECO:0000269|PubMed:26037923}; EC=1.1.1.195 {ECO:0000269|PubMed:26037923}; Flags: Precursor (At1g30760)	2.004	
Cs9g16460	K22097 3-O-acetylpapaveroxine carboxylesterase [EC:3.1.1.105] | (RefSeq) carboxylesterase 1-like (A)	Carboxylesterase 1; AeCXE1; EC=3.1.1.1 (At5g62180)		1.390
Cs9g16480	K22097 3-O-acetylpapaveroxine carboxylesterase [EC:3.1.1.105] | (RefSeq) carboxylesterase 1-like (A)	Carboxylesterase 1; AeCXE1; EC=3.1.1.1 (At5g62180)		1.350
Cs9g16490	K22097 3-O-acetylpapaveroxine carboxylesterase [EC:3.1.1.105] | (RefSeq) carboxylesterase 1-like (A)	Carboxylesterase 1; AeCXE1; EC=3.1.1.1 (At5g62180)	1.048	
Cs9g16520	K05282 gibberellin 20-oxidase [EC:1.14.11.12] | (RefSeq) gibberellin 20 oxidase 1 (A)	Gibberellin 20 oxidase 1; EC=1.14.11.-; GA 20-oxidase 1; AtGA20ox1; Gibberellin C-20 oxidase 1 (At4g25420)		-1.697
Cs9g16550	K00847 fructokinase [EC:2.7.1.4] | (RefSeq) hypothetical protein (A)	Probable fructokinase-7; EC=2.7.1.4 (At5g51830)	-1.148	
Cs9g16590	K00430 peroxidase [EC:1.11.1.7] | (RefSeq) peroxidase 66 (A)	Peroxidase 66; Atperox P66; EC=1.11.1.7; ATP27a; Flags: Precursor (At5g51890)	2.440	5.362
Cs9g17270	K01623 fructose-bisphosphate aldolase, class I [EC:4.1.2.13] | (RefSeq) fructose-bisphosphate aldolase 6, cytosolic-like (A)	Heavy metal-associated isoprenylated plant protein 39 {ECO:0000303|PubMed:21072340, ECO:0000303|PubMed:23368984}; AtHIP39 {ECO:0000303|PubMed:21072340, ECO:0000303|PubMed:23368984}; Flags: Precursor (At1g01490)		-1.134
Cs9g17840	K09833 homogentisate phytyltransferase / homogentisate	Coumarin 8-geranyltransferase 1b, chloroplastic; EC=2.5.1.138		1.987

	geranylgeranyltransferase [EC:2.5.1.115 2.5.1.116] | (RefSeq) homogentisate phytyltransferase 1, chloroplastic-like (A)	{ECO:0000269|PubMed:25077796}; Prenyltransferase 1b {ECO:0000303|PubMed:25077796}; ClPT1ba {ECO:0000303|PubMed:25077796}; Flags: Precursor (At3g11950)		
Cs9g17850	K22395 cinnamyl-alcohol dehydrogenase [EC:1.1.1.195] | (RefSeq) tetrahydrocannabinolic acid synthase-like isoform X1 (A)	Berberine bridge enzyme-like 5 {ECO:0000303|PubMed:26037923}; AtBBE-like 5 {ECO:0000303|PubMed:26037923}; EC=1.1.1.- {ECO:0000250|UniProtKB:O64743}; Flavin-dependent oxidoreductase FOX3 {ECO:0000303|PubMed:26352477}; EC=1.-.-.- {ECO:0000305}; Flags: Precursor (At1g26400)		-1.848
Cs9g18120	K00873 pyruvate kinase [EC:2.7.1.40] | (RefSeq) hypothetical protein (A)	Plastidial pyruvate kinase 4, chloroplastic; PKp4; EC=2.7.1.40; Flags: Precursor (At3g49160)	-1.526	-1.586
Cs9g18290	K01087 trehalose 6-phosphate phosphatase [EC:3.1.3.12] | (RefSeq) probable trehalose-phosphate phosphatase C (A)	Trehalose 6-phosphate phosphatase RA3 {ECO:0000305}; EC=3.1.3.12 {ECO:0000269|PubMed:16688177}; Protein RAMOSA 3 {ECO:0000303|PubMed:16688177} (At1g78090)	1.386	1.065
Cs9g18820	K22845 phlorizin synthase [EC:2.4.1.357] | (RefSeq) UDP-glycosyltransferase 88A1 (A)	Anthocyanidin 5,3-O-glucosyltransferase; EC=2.4.1.-; UDP-glucose: anthocyanidin 5,3-O-glucosyltransferase (At3g16520)		-2.140
Cs9g18830	K22845 phlorizin synthase [EC:2.4.1.357] | (RefSeq) UDP-glycosyltransferase 88A1 (A)	Anthocyanidin 5,3-O-glucosyltransferase; EC=2.4.1.-; UDP-glucose: anthocyanidin 5,3-O-glucosyltransferase (At3g16520)		-3.767
Cs9g19030	K13065 shikimate O-hydroxycinnamoyltransferase [EC:2.3.1.133] | (RefSeq) hypothetical protein (A)	BAHD acyltransferase At5g47980; EC=2.3.1.- (At3g26040)		1.030
Cs9g19270	K15746 beta-carotene 3-hydroxylase [EC:1.14.15.24] | (RefSeq) hypothetical protein (A)	Beta-carotene 3-hydroxylase, chloroplastic; GenCHYB; EC=1.14.15.24 {ECO:0000250|UniProtKB:Q9SZZ8}; Flags: Precursor (At5g52570)		-1.790
Cs9g19490	K22395 cinnamyl-alcohol dehydrogenase [EC:1.1.1.195] | (RefSeq) tetrahydrocannabinolic acid synthase-like isoform X1 (A)	Berberine bridge enzyme-like 5 {ECO:0000303|PubMed:26037923}; AtBBE-like 5 {ECO:0000303|PubMed:26037923}; EC=1.1.1.- {ECO:0000250|UniProtKB:O64743}; Flavin-dependent oxidoreductase FOX3 {ECO:0000303|PubMed:26352477}; EC=1.-.-.- {ECO:0000305}; Flags: Precursor (At1g26400)		-1.240
novel.1333	K13789 geranylgeranyl diphosphate synthase, type II [EC:2.5.1.1 2.5.1.10 2.5.1.29] | (RefSeq) geranylgeranyl pyrophosphate synthase, chloroplastic-like (A)	GGPPS_HEVBR Geranylgeranyl pyrophosphate synthase, chloroplastic; GGPP synthase; GGPS; EC=2.5.1.-; (2E,6E)-farnesyl diphosphate synthase; Dimethylallyltranstransferase; EC=2.5.1.1; Farnesyl diphosphate synthase; Farnesyltranstransferase; EC=2.5.1.29; Geranyltranstransferase; EC=2.5.1.10; Flags: Precursor		-1.435

		(At4g36810)		
novel.1439	K09753 cinnamoyl-CoA reductase [EC:1.2.1.44] | (RefSeq) cinnamoyl-CoA reductase 1-like isoform X1 (A)	POLR1_ARATH Retrovirus-related Pol polyprotein from transposon RE1; Retro element 1 {ECO:0000303|PubMed:10689195}; AtRE1 {ECO:0000303|PubMed:10689195}; Includes: Protease RE1; EC=3.4.23.-; Includes: Reverse transcriptase RE1; EC=2.7.7.49; Includes: Endonuclease RE1 (At1g44510)	2.066	2.170
novel.1553	K10258 very-long-chain enoyl-CoA reductase [EC:1.3.1.93] | (RefSeq) very-long-chain enoyl-CoA reductase (A)	DET2_GOSHI Steroid 5-alpha-reductase DET2; GhDET2; EC=1.3.1.22 (At5g16010)		1.023
novel.1672	K00764 amidophosphoribosyltransferase [EC:2.4.2.14] | (RefSeq) hypothetical protein (A)	ASE1_ARATH Amidophosphoribosyltransferase 1, chloroplastic; AtATase1; PRPP1; EC=2.4.2.14; Glutamine phosphoribosylpyrophosphate amidotransferase 1; AtGPRAT1; Flags: Precursor (At2g16570)		1.033
novel.1919	K05349 beta-glucosidase [EC:3.2.1.21] | (RefSeq) lysosomal beta glucosidase-like (A)	POLR2_ARATH Retrovirus-related Pol polyprotein from transposon RE2; Retro element 2 {ECO:0000303|PubMed:10689195}; AtRE2 {ECO:0000303|PubMed:10689195}; Includes: Protease RE2; EC=3.4.23.-; Includes: Reverse transcriptase RE2; EC=2.7.7.49; Includes: Endonuclease RE2 (At4g16020)	-1.420	
novel.1945	K22395 cinnamyl-alcohol dehydrogenase [EC:1.1.1.195] | (RefSeq) flavin-dependent oxidoreductase FOX2-like (A)	BBE15_ARATH Berberine bridge enzyme-like 15 {ECO:0000303|PubMed:26037923}; AtBBE-like 15 {ECO:0000303|PubMed:26037923}; EC=1.1.1.194 {ECO:0000269|PubMed:26037923}; EC=1.1.1.195 {ECO:0000269|PubMed:26037923}; Protein EMBRYO SAC DEVELOPMENT ARREST 28 {ECO:0000303|PubMed:15634699}; Protein MATERNAL EFFECT EMBRYO ARREST 23 {ECO:0000303|PubMed:15634699}; Flags: Precursor (At2g34790)		1.427
novel.2012	K00234 succinate dehydrogenase (ubiquinone) flavoprotein subunit [EC:1.3.5.1] | (RefSeq) succinate dehydrogenase [ubiquinone] flavoprotein subunit, mitochondrial-like (A)	(At1g35647)	1.093	1.829
novel.2174	K09753 cinnamoyl-CoA reductase [EC:1.2.1.44] | (RefSeq) cinnamoyl-CoA reductase 1-like isoform X1 (A)	POLR1_ARATH Retrovirus-related Pol polyprotein from transposon RE1; Retro element 1 {ECO:0000303|PubMed:10689195}; AtRE1 {ECO:0000303|PubMed:10689195}; Includes: Protease RE1; EC=3.4.23.-; Includes: Reverse transcriptase RE1; EC=2.7.7.49; Includes: Endonuclease RE1 (At1g27285)	4.246	
novel.2285	K13066 caffeic acid 3-O-methyltransferase [EC:2.1.1.68] | (RefSeq)	COMT2_OCIBA Caffeic acid 3-O-methyltransferase 2; CAOMT-2;		-1.310

	COMT1-2; caffeic acid O-methyltransferase (A)	COMT-2; EC=2.1.1.68; S-adenosysl-L-methionine:caffeic acid 3-O-methyltransferase 2 (At3g53140)		
novel.2409	K00454 lipoxygenase [EC:1.13.11.12] | (RefSeq) hypothetical protein (A)			-3.448
novel.2410	K00454 lipoxygenase [EC:1.13.11.12] | (RefSeq) hypothetical protein (A)	LOX21_SOLTU Linoleate 13S-lipoxygenase 2-1, chloroplastic; EC=1.13.11.12; Lipoxygenase 2-1; Flags: Precursor (At3g45140)		-1.693
novel.2411	K00454 lipoxygenase [EC:1.13.11.12] | (RefSeq) hypothetical protein (A)	LOX21_SOLTU Linoleate 13S-lipoxygenase 2-1, chloroplastic; EC=1.13.11.12; Lipoxygenase 2-1; Flags: Precursor (At3g45140)		-1.769
novel.25	K00815 tyrosine aminotransferase [EC:2.6.1.5] | (RefSeq) LOW QUALITY PROTEIN: tyrosine aminotransferase-like (A)	(At2g06190)	-1.347	
novel.2508	K13066 caffeic acid 3-O-methyltransferase [EC:2.1.1.68] | (RefSeq) anthranilate N-methyltransferase-like (A)	COMT1_MEDSA Caffeic acid 3-O-methyltransferase; CAOMT; COMT; EC=2.1.1.68; S-adenosysl-L-methionine:caffeic acid 3-O-methyltransferase (At5g54160)		2.654
novel.2683	K22395 cinnamyl-alcohol dehydrogenase [EC:1.1.1.195] | (RefSeq) tetrahydrocannabinolic acid synthase-like isoform X1 (A)	BBE18_ARATH Berberine bridge enzyme-like 18 {ECO:0000303|PubMed:26037923}; AtBBE-like 18 {ECO:0000303|PubMed:26037923}; EC=1.1.1.- {ECO:0000250|UniProtKB:O64743}; Flags: Precursor (At4g20820)		2.006
novel.2758	K13065 shikimate O-hydroxycinnamoyltransferase [EC:2.3.1.133] | (RefSeq) shikimate O-hydroxycinnamoyltransferase-like (A)	HST_TOBAC Shikimate O-hydroxycinnamoyltransferase; EC=2.3.1.133; Hydroxycinnamoyl transferase; Hydroxycinnamoyl-Coenzyme A shikimate/quinate hydroxycinnamoyltransferase (At5g48930)		-1.226
novel.2812	K05894 12-oxophytodienoic acid reductase [EC:1.3.1.42] | (RefSeq) putative 12-oxophytodienoate reductase 11 (A)		-3.142	-2.219
novel.2856	K09833 homogentisate phytyltransferase / homogentisate geranylgeranyltransferase [EC:2.5.1.115 2.5.1.116] | (RefSeq) hypothetical protein (A)		1.222	1.572
novel.320	K09833 homogentisate phytyltransferase / homogentisate geranylgeranyltransferase [EC:2.5.1.115 2.5.1.116] | (RefSeq) hypothetical protein (A)	CGT1A_CITLI Coumarin 8-geranyltransferase 1, chloroplastic; EC=2.5.1.138 {ECO:0000269|PubMed:25077796}; Prenyltransferase 1 {ECO:0000303|PubMed:25077796}; ClPT1 {ECO:0000303|PubMed:25077796}; Umbelliferone 8-C-geranyltransferase {ECO:0000303|PubMed:25077796}; U8GT {ECO:0000303|PubMed:25077796}; Flags: Precursor	1.066	
novel.424	K01778 diaminopimelate epimerase [EC:5.1.1.7] | (RefSeq) hypothetical protein (A)	DAPF_ARATH Diaminopimelate epimerase, chloroplastic; DAP epimerase; EC=5.1.1.7; Flags: Precursor (At3g53580)		-1.524

novel.477	K00430 peroxidase [EC:1.11.1.7] | (RefSeq) lignin-forming anionic peroxidase-like (A)	PERX_NICSY Lignin-forming anionic peroxidase; EC=1.11.1.7; Flags: Precursor (At1g14550)	-1.786	2.002
novel.738	K13065 shikimate O-hydroxycinnamoyltransferase [EC:2.3.1.133] | (RefSeq) shikimate O-hydroxycinnamoyltransferase (A)	HST_ARATH Shikimate O-hydroxycinnamoyltransferase; EC=2.3.1.133; Hydroxycinnamoyl transferase; Hydroxycinnamoyl-Coenzyme A shikimate/quinate hydroxycinnamoyltransferase (At5g48930)		-1.997
novel.739	K13065 shikimate O-hydroxycinnamoyltransferase [EC:2.3.1.133] | (RefSeq) shikimate O-hydroxycinnamoyltransferase-like (A)	HST_ARATH Shikimate O-hydroxycinnamoyltransferase; EC=2.3.1.133; Hydroxycinnamoyl transferase; Hydroxycinnamoyl-Coenzyme A shikimate/quinate hydroxycinnamoyltransferase (At5g48930)		-1.375
novel.975	K13066 caffeic acid 3-O-methyltransferase [EC:2.1.1.68] | (RefSeq) hypothetical protein (A)	COMT1_MEDSA Caffeic acid 3-O-methyltransferase; CAOMT; COMT; EC=2.1.1.68; S-adenosysl-L-methionine:caffeic acid 3-O-methyltransferase (At5g54160)	2.726	
orange1.1t00198	K00981 phosphatidate cytidylyltransferase [EC:2.7.7.41] | (RefSeq) hypothetical protein (A)	Phosphatidate cytidylyltransferase 4, chloroplastic {ECO:0000303|PubMed:20442275}; EC=2.7.7.41 {ECO:0000269|PubMed:20442275}; CDP-DAG synthase 4; CDP-DG synthase 4; CDP-diacylglycerol synthase 4; CDS4; CDP-diglyceride pyrophosphorylase 4; CDP-diglyceride synthase 4; CTP:phosphatidate cytidylyltransferase 4; Flags: Precursor (At2g45150)		1.024
orange1.1t00222	K01476 arginase [EC:3.5.3.1] | (RefSeq) hypothetical protein (A)	Arginase 1, mitochondrial; EC=3.5.3.1 {ECO:0000250|UniProtKB:P05089}; Agmatinase ARGAH1 {ECO:0000305}; EC=3.5.3.11 {ECO:0000305|PubMed:28716421}; Arginine amidohydrolase 1; Flags: Precursor (At4g08900)		1.387
orange1.1t00267	K22097 3-O-acetylpapaveroxine carboxylesterase [EC:3.1.1.105] | (RefSeq) carboxylesterase 1-like (A)	Probable carboxylesterase 8; AtCXE8; EC=3.1.1.1 (At2g45600)		-2.325
orange1.1t00271	K15919 hydroxypyruvate reductase 2 | (RefSeq) hypothetical protein (A)	Glyoxylate/hydroxypyruvate reductase HPR3; EC=1.1.1.79; EC=1.1.1.81; NAD(P)H-dependent hydroxypyruvate reductase 3; AtHPR3; HPR 3 (At2g45630)	-1.350	
orange1.1t00277	K00847 fructokinase [EC:2.7.1.4] | (RefSeq) hypothetical protein (A)	Probable fructokinase-7; EC=2.7.1.4 (At5g51830)	-1.072	
orange1.1t00345	K00549 5-methyltetrahydropteroyltriglutamatehomocysteine methyltransferase [EC:2.1.1.14] | (RefSeq) protein QUIRKY-like (A)	FT-interacting protein 1 {ECO:0000303|PubMed:22529749} (At4g11610)	1.167	1.418
orange1.1t003	K13508 glycerol-3-phosphate acyltransferase [EC:2.3.1.15	Probable glycerol-3-phosphate acyltransferase 8; EC=2.3.1.15	1.009	3.308

75	2.3.1.198] | (RefSeq) probable glycerol-3-phosphate acyltransferase 8 (A)	(At1g01610)		
orange1.1t00416	K20772 1-aminocyclopropane-1-carboxylate synthase 1/2/6 [EC:4.4.1.14] | (RefSeq) hypothetical protein (A)	1-aminocyclopropane-1-carboxylate synthase 1; ACC synthase 1; EC=4.4.1.14; S-adenosyl-L-methionine methylthioadenosine-lyase (At3g61510)	2.773	
orange1.1t00484	K01513 ectonucleotide pyrophosphatase/phosphodiesterase family member 1/3 [EC:3.1.4.1 3.6.1.9] | (RefSeq) uncharacterized protein LOC109769794 (A)	(At2g46420)		-1.935
orange1.1t00556	K15397 3-ketoacyl-CoA synthase [EC:2.3.1.199] | (RefSeq) 3-ketoacyl-CoA synthase 1 (A)	3-ketoacyl-CoA synthase 1 {ECO:0000303|PubMed:10074711}; KCS-1 {ECO:0000303|PubMed:10074711}; EC=2.3.1.199 {ECO:0000269|PubMed:10074711, ECO:0000269|PubMed:16765910}; Very long-chain fatty acid condensing enzyme 1 {ECO:0000303|PubMed:10074711}; VLCFA condensing enzyme 1 {ECO:0000303|PubMed:10074711} (At1g01120)		1.797
orange1.1t00577	K12643 anthranilate N-methyltransferase [EC:2.1.1.111] | (RefSeq) anthranilate N-methyltransferase-like (A)	Anthranilate N-methyltransferase; RgANMT; EC=2.1.1.111 (At5g54160)	1.373	
orange1.1t00747	K14674 TAG lipase / steryl ester hydrolase / phospholipase A2 / LPA acyltransferase [EC:3.1.1.3 3.1.1.13 3.1.1.4 2.3.1.51] | (RefSeq) triacylglycerol lipase SDP1 (A)	Triacylglycerol lipase SDP1; EC=3.1.1.3; Protein SUGAR-DEPENDENT 1 (At5g04040)		-1.368
orange1.1t01032	K06892 feruloyl-CoA ortho-hydroxylase [EC:1.14.11.-] | (RefSeq) 2-oxoglutarate-iron(II)-dependent oxygenase (A)	Protein DMR6-LIKE OXYGENASE 1 {ECO:0000303|PubMed:25376907}; EC=1.14.11.- {ECO:0000255|PROSITE-ProRule:PRU00805}; 2-oxoglutarate (2OG)-Fe(II) oxygenase-like protein DLO1 {ECO:0000303|PubMed:25376907}; Protein SENESCENCE-ASSOCIATED GENE 108 {ECO:0000303|PubMed:23959884}; Salicylate 3-hydroxylase DLO1 {ECO:0000303|PubMed:23959884}; S3H DLO1 {ECO:0000303|PubMed:23959884}; SA 3-hydroxylase DLO1 {ECO:0000303|PubMed:23959884}; Salicylic acid 3-hydroxylase DLO1 {ECO:0000303|PubMed:23959884}; EC=1.14.13.- {ECO:0000269|PubMed:23959884} (At2g36690)		1.348
orange1.1t01039	K08081 tropinone reductase I [EC:1.1.1.206] | (RefSeq) tropinone reductase homolog At5g06060-like (A)	Zinc finger protein 10 {ECO:0000305}; AtZFP10 {ECO:0000303|PubMed:12154136} (At4g17810)		-7.776

orange1.1t01084	K13066 caffeic acid 3-O-methyltransferase [EC:2.1.1.68] | (Kazusa) Lj2g3v3339490.1; - (A)	Caffeic acid 3-O-methyltransferase; CAOMT; COMT; EC=2.1.1.68; S-adenosysl-L-methionine:caffeic acid 3-O-methyltransferase (At3g53140)	-1.168	-1.088
orange1.1t01105	K01187 alpha-glucosidase [EC:3.2.1.20] | (RefSeq) alpha-glucosidase 2-like (A)	Probable glucan 1,3-alpha-glucosidase; EC=3.2.1.84; Glucosidase II subunit alpha; Protein PRIORITY IN SWEET LIFE 5; Protein RADIAL SWELLING 3; Flags: Precursor (At3g23640)		1.095
orange1.1t01340	K22395 cinnamyl-alcohol dehydrogenase [EC:1.1.1.195] | (RefSeq) berberine bridge enzyme-like 15 (A)	Berberine bridge enzyme-like 5 {ECO:0000303|PubMed:26037923}; AtBBE-like 5 {ECO:0000303|PubMed:26037923}; EC=1.1.1.- {ECO:0000250|UniProtKB:O64743}; Flavin-dependent oxidoreductase FOX3 {ECO:0000303|PubMed:26352477}; EC=1.-.-.- {ECO:0000305}; Flags: Precursor (At1g26400)		-2.274
orange1.1t01401	K14975 codeine 3-O-demethylase [EC:1.14.11.32] | (RefSeq) codeine O-demethylase (A)	Codeine O-demethylase; EC=1.14.11.32 (At1g49390)	1.295	
orange1.1t01456	K12153 phenylalanine N-monooxygenase [EC:1.14.14.40] | (RefSeq) phenylalanine N-monooxygenase-like (A)	Phenylalanine N-monooxygenase; EC=1.14.14.40 {ECO:0000269|PubMed:10799553}; Cytochrome P450 79A2; Phenylalanine N-hydroxylase (At5g05260)		1.163
orange1.1t01523	K21354 beta-D-glucosyl crocetin beta-1,6-glucosyltransferase [EC:2.4.1.330] | (RefSeq) hypothetical protein (A)	Beta-D-glucosyl crocetin beta-1,6-glucosyltransferase; EC=2.4.1.330 {ECO:0000269|PubMed:22569263}; UDP-glucose glucosyltransferase 9; GjUGT9; UDP-glycosyltransferase 94E5 (At5g65550)	4.195	1.974
orange1.1t01882	K22772 flavonol-3-O-glucoside L-rhamnosyltransferase [EC:2.4.1.159] | (RefSeq) 1,6-rhamnosyltransferase (A)	Anthocyanidin 3-O-glucosyltransferase; EC=2.4.1.115; Anthocyanin rhamnosyl transferase; Flavonol 3-O-glucosyltransferase; UDP-glucose flavonoid 3-O-glucosyltransferase (At5g54010)		-1.602
orange1.1t01950	K22395 cinnamyl-alcohol dehydrogenase [EC:1.1.1.195] | (RefSeq) tetrahydrocannabinolic acid synthase-like isoform X1 (A)	Berberine bridge enzyme-like 18 {ECO:0000303|PubMed:26037923}; AtBBE-like 18 {ECO:0000303|PubMed:26037923}; EC=1.1.1.- {ECO:0000250|UniProtKB:O64743}; Flags: Precursor (At4g20820)	3.214	
orange1.1t01951	K22395 cinnamyl-alcohol dehydrogenase [EC:1.1.1.195] | (RefSeq) tetrahydrocannabinolic acid synthase-like isoform X1 (A)	Berberine bridge enzyme-like 18 {ECO:0000303|PubMed:26037923}; AtBBE-like 18 {ECO:0000303|PubMed:26037923}; EC=1.1.1.- {ECO:0000250|UniProtKB:O64743}; Flags: Precursor (At4g20820)	2.936	
orange1.1t01952	K22395 cinnamyl-alcohol dehydrogenase [EC:1.1.1.195] | (RefSeq) tetrahydrocannabinolic acid synthase-like isoform X1 (A)	Berberine bridge enzyme-like 18 {ECO:0000303|PubMed:26037923}; AtBBE-like 18	3.091	2.022

		{ECO:0000303|PubMed:26037923}; EC=1.1.1.- {ECO:0000250|UniProtKB:O64743}; Flags: Precursor (At4g20820)		
orange1.1t01953	K22395 cinnamyl-alcohol dehydrogenase [EC:1.1.1.195] | (RefSeq) tetrahydrocannabinolic acid synthase-like isoform X1 (A)	Cannabidiolic acid synthase; EC=1.21.3.8; CBDA synthase; Flags: Precursor (At4g20820)	1.149	1.281
orange1.1t01955	K22395 cinnamyl-alcohol dehydrogenase [EC:1.1.1.195] | (RefSeq) tetrahydrocannabinolic acid synthase-like isoform X1 (A)	Cannabidiolic acid synthase-like 2; Flags: Precursor (At4g20820)		-1.161
orange1.1t01957	K22395 cinnamyl-alcohol dehydrogenase [EC:1.1.1.195] | (RefSeq) tetrahydrocannabinolic acid synthase-like isoform X1 (A)	Cannabidiolic acid synthase; EC=1.21.3.8; CBDA synthase; Flags: Precursor (At4g20820)	3.258	2.267
orange1.1t01981	K13496 UDP-glucosyl transferase 73C [EC:2.4.1.-] | (RefSeq) UGT2; UDP-glycosyltransferase 73C6-like (A)	UDP-glycosyltransferase 73C2; EC=2.4.1.- (At2g36760)	1.895	
orange1.1t01983	K13496 UDP-glucosyl transferase 73C [EC:2.4.1.-] | (RefSeq) UDP-glycosyltransferase 73C3-like (A)	UDP-glycosyltransferase 73C3; EC=2.4.1.- (At2g36780)		-1.299
orange1.1t02033	K00430 peroxidase [EC:1.11.1.7] | (RefSeq) peroxidase 59-like (A)	Peroxidase N; EC=1.11.1.7; Neutral peroxidase; Flags: Precursor (At5g19890)		2.207
orange1.1t02041	K00430 peroxidase [EC:1.11.1.7] | (RefSeq) peroxidase A2 (A)	Peroxidase C3; EC=1.11.1.7; Flags: Precursor (At5g06720)		-1.203
orange1.1t02045	K00430 peroxidase [EC:1.11.1.7] | (RefSeq) peroxidase 15-like (A)	Peroxidase 15 {ECO:0000303|PubMed:17936696}; Prx15 {ECO:0000303|PubMed:17936696}; EC=1.11.1.7; Anionic peroxidase {ECO:0000303|PubMed:17936696}; Flags: Precursor (At5g06720)	-1.018	
orange1.1t02059	K00430 peroxidase [EC:1.11.1.7] | (RefSeq) peroxidase A2 (A)	Lignin-forming anionic peroxidase; EC=1.11.1.7; TOPA; Flags: Precursor (At5g06720)		1.147
orange1.1t02061	K01193 beta-fructofuranosidase [EC:3.2.1.26] | (RefSeq) LOC109747879; beta-fructofuranosidase, insoluble isoenzyme 3-like (A)	UPF0481 protein At3g47200 (At4g31980_2)		1.416
orange1.1t02085	K13066 caffeic acid 3-O-methyltransferase [EC:2.1.1.68] | (RefSeq) caffeic acid 3-O-methyltransferase-like isoform X1 (A)	Caffeic acid 3-O-methyltransferase 1; CAOMT-1; COMT-1; EC=2.1.1.68; S-adenosysl-L-methionine:caffeic acid 3-O-methyltransferase 1 (At5g54160)		-2.085
orange1.1t02091	K13066 caffeic acid 3-O-methyltransferase [EC:2.1.1.68] | (RefSeq) hypothetical protein (A)	Caffeic acid 3-O-methyltransferase 1; CAOMT-1; COMT-1; EC=2.1.1.68; S-adenosysl-L-methionine:caffeic acid 3-O-methyltransferase 1 (At5g54160)		-2.365
orange1.1t02225	K00430 peroxidase [EC:1.11.1.7] | (RefSeq) LOW QUALITY PROTEIN: peroxidase A2-like (A)	Peroxidase 53; Atperox P53; EC=1.11.1.7; ATPA2; Flags: Precursor (At5g06720)		1.571

orange1.1t02279	K00228 coproporphyrinogen III oxidase [EC:1.3.3.3] | (RefSeq) oxygen-dependent coproporphyrinogen-III oxidase, chloroplastic (A)	Oxygen-dependent coproporphyrinogen-III oxidase, chloroplastic; Coprogen oxidase; Coproporphyrinogenase; EC=1.3.3.3; Flags: Precursor (At1g03475)	-1.642	
orange1.1t02409	K21374 7-deoxyloganetin glucosyltransferase [EC:2.4.1.324] | (RefSeq) hypothetical protein (A)	UDP-glycosyltransferase 86A1; EC=2.4.1.- (At2g36970)	2.482	1.998
orange1.1t02784	K20623 typhasterol/6-deoxotyphasterol 2alpha-hydroxylase | (RefSeq) hypothetical protein (A)	Cytochrome P450 71A1; EC=1.14.-.-; ARP-2; CYPLXXIA1 (At5g07990)	1.207	2.232
orange1.1t02793	K13065 shikimate O-hydroxycinnamoyltransferase [EC:2.3.1.133] | (RefSeq) shikimate O-hydroxycinnamoyltransferase (A)	Shikimate O-hydroxycinnamoyltransferase; EC=2.3.1.133; Hydroxycinnamoyl transferase; Hydroxycinnamoyl-Coenzyme A shikimate/quinate hydroxycinnamoyltransferase (At5g48930)		1.021
orange1.1t02914	K00006 glycerol-3-phosphate dehydrogenase (NAD+) [EC:1.1.1.8] | (RefSeq) glycerol-3-phosphate dehydrogenase [NAD(+)] GPDHC1, cytosolic (A)	Probable glycerol-3-phosphate dehydrogenase [NAD(+)] 1, cytosolic; EC=1.1.1.8 (At2g41540)		2.185
orange1.1t03034	K08081 tropinone reductase I [EC:1.1.1.206] | (RefSeq) tropinone reductase homolog (A)	Tropinone reductase homolog; EC=1.1.1.-; P29X (At5g06060)	1.610	
orange1.1t03042	K08081 tropinone reductase I [EC:1.1.1.206] | (RefSeq) tropinone reductase homolog isoform X1 (A)	Tropinone reductase homolog; EC=1.1.1.-; P29X (At2g29150)	2.066	
orange1.1t03152	K23260 scopoletin glucosyltransferase [EC:2.4.1.128] | (RefSeq) scopoletin glucosyltransferase (A)	UDP-glycosyltransferase 92A1; EC=2.4.1.- (At5g12890)	1.121	2.980
orange1.1t03432	K13496 UDP-glucosyl transferase 73C [EC:2.4.1.-] | (RefSeq) UDP-glycosyltransferase 73C5-like (A)	UDP-glycosyltransferase 87A2; EC=2.4.1.- (At2g30140)	-1.880	-1.902
orange1.1t03475	K16818 phospholipase A1 [EC:3.1.1.32] | (RefSeq) phospholipase A(1) DAD1, chloroplastic-like (A)	Phospholipase A(1) DAD1, chloroplastic; EC=3.1.1.32; Phospholipase A1-Ibeta1; Protein DEFECTIVE IN ANTHER DEHISCENCE 1; AtDAD1; Flags: Precursor (At4g13550)	1.937	
orange1.1t03484	K13066 caffeic acid 3-O-methyltransferase [EC:2.1.1.68] | (RefSeq) hypothetical protein (A)	Anthranilate N-methyltransferase; RgANMT; EC=2.1.1.111 (At5g54160)	1.202	1.252
orange1.1t03535	K01581 ornithine decarboxylase [EC:4.1.1.17] | (RefSeq) hypothetical protein (A)	Ornithine decarboxylase; ODC; EC=4.1.1.17 (YKL184w)		-1.815
orange1.1t03552	K01613 phosphatidylserine decarboxylase [EC:4.1.1.65] | (RefSeq) phosphatidylserine decarboxylase proenzyme 2-like (A)	Phosphatidylserine decarboxylase proenzyme 2; EC=4.1.1.65 {ECO:0000255|HAMAP-Rule:MF_03209}; Contains: Phosphatidylserine decarboxylase 2 beta chain; Contains: Phosphatidylserine decarboxylase 2 alpha chain (At5g57190)		1.130
orange1.1t03726	K05894 12-oxophytodienoic acid reductase [EC:1.3.1.42] | (RefSeq) putative 12-oxophytodienoate reductase 11 (A)	Putative 12-oxophytodienoate reductase 11; EC=1.3.1.-; OPDA-reductase 11; OsOPR11 (At1g76690)	4.152	

orange1.1t03727	K05894 12-oxophytodienoic acid reductase [EC:1.3.1.42] | (RefSeq) putative 12-oxophytodienoate reductase 11 (A)	Putative 12-oxophytodienoate reductase 11; EC=1.3.1.-; OPDA-reductase 11; OsOPR11 (At1g76690)		1.102
orange1.1t03770	K00454 lipoxygenase [EC:1.13.11.12] | (RefSeq) hypothetical protein (A)	Linoleate 13S-lipoxygenase 2-1, chloroplastic; EC=1.13.11.12; Lipoxygenase 2-1; Flags: Precursor (At3g45140)		-7.503
orange1.1t03775	K00454 lipoxygenase [EC:1.13.11.12] | (RefSeq) hypothetical protein (A)	Linoleate 13S-lipoxygenase 2-1, chloroplastic; EC=1.13.11.12; Lipoxygenase 2-1; Flags: Precursor (At3g45140)	-1.544	
orange1.1t03987	K13066 caffeic acid 3-O-methyltransferase [EC:2.1.1.68] | (RefSeq) caffeic acid 3-O-methyltransferase-like (A)	Caffeic acid 3-O-methyltransferase; CAOMT; COMT; EC=2.1.1.68; S-adenosysl-L-methionine:caffeic acid 3-O-methyltransferase (At5g54160)	6.849	
orange1.1t04173	K22395 cinnamyl-alcohol dehydrogenase [EC:1.1.1.195] | (RefSeq) tetrahydrocannabinolic acid synthase-like isoform X1 (A)	Cannabidiolic acid synthase-like 2; Flags: Precursor (At4g20820)	2.399	2.417
orange1.1t04194	K13066 caffeic acid 3-O-methyltransferase [EC:2.1.1.68] | (RefSeq) caffeic acid 3-O-methyltransferase-like (A)	Caffeic acid 3-O-methyltransferase; CAOMT; COMT; EC=2.1.1.68; S-adenosysl-L-methionine:caffeic acid 3-O-methyltransferase (At5g54160)	3.478	
orange1.1t04376	K00454 lipoxygenase [EC:1.13.11.12] | (RefSeq) linoleate 13S-lipoxygenase 2-1, chloroplastic (A)	Linoleate 13S-lipoxygenase 2-1, chloroplastic; EC=1.13.11.12; Lipoxygenase 2-1; Flags: Precursor (At3g45140)	-1.312	-2.168
orange1.1t04380	K03781 catalase [EC:1.11.1.6] | (RefSeq) uncharacterized protein y4iK-like (A)	4-coumarateCoA ligase-like 2; EC=6.2.1.- (Hs22051274)	2.250	-7.568
orange1.1t04496	K00058 D-3-phosphoglycerate dehydrogenase / 2-oxoglutarate reductase [EC:1.1.1.95 1.1.1.399] | (RefSeq) D-3-phosphoglycerate dehydrogenase 2, chloroplastic-like (A)	D-3-phosphoglycerate dehydrogenase 2, chloroplastic; PGDH; EC=1.1.1.95 {ECO:0000269|PubMed:24368794, ECO:0000269|PubMed:9867856}; Flags: Precursor (At1g17740)		1.349
orange1.1t04551	K09833 homogentisate phytyltransferase / homogentisate geranylgeranyltransferase [EC:2.5.1.115 2.5.1.116] | (RefSeq) homogentisate phytyltransferase 1, chloroplastic-like (A)	Coumarin 8-geranyltransferase 1b, chloroplastic; EC=2.5.1.138 {ECO:0000269|PubMed:25077796}; Prenyltransferase 1b {ECO:0000303|PubMed:25077796}; ClPT1ba {ECO:0000303|PubMed:25077796}; Flags: Precursor (At2g18950)		2.387
orange1.1t04629	K00121 S-(hydroxymethyl)glutathione dehydrogenase / alcohol dehydrogenase [EC:1.1.1.284 1.1.1.1] | (RefSeq) alcohol dehydrogenase-like 2 (A)	Alcohol dehydrogenase-like 2; EC=1.1.1.1 {ECO:0000250|UniProtKB:P06525} (At1g22440)		-6.311
orange1.1t04783	K21373 7-deoxyloganetic acid glucosyltransferase [EC:2.4.1.323] | (RefSeq) 7-deoxyloganetic acid glucosyltransferase-like (A)	Uncharacterized protein At1g66480 (At2g01340)		-1.196
orange1.1t04849	K09838 zeaxanthin epoxidase [EC:1.14.15.21] | (RefSeq) Zeaxanthin epoxidase, chloroplastic (A)	Zeaxanthin epoxidase, chloroplastic; AtZEP; EC=1.14.15.21; Protein ABA DEFICIENT 1; AtABA1; Protein IMPAIRED IN BABA-INDUCED STERILITY 3; Protein LOW EXPRESSION OF OSMOTIC STRESS-RESPONSIVE GENES 6; Protein	3.690	

		NON-PHOTOCHEMICAL QUENCHING 2; Flags: Precursor (At5g11330)		
orange1.1t04957	K13789 geranylgeranyl diphosphate synthase, type II [EC:2.5.1.1 2.5.1.10 2.5.1.29] | (RefSeq) geranylgeranyl pyrophosphate synthase, chloroplastic/chromoplastic-like (A)	Geranylgeranyl pyrophosphate synthase, chloroplastic; GGPP synthase; GGPS; EC=2.5.1.-; (2E,6E)-farnesyl diphosphate synthase; Dimethylallyltranstransferase; EC=2.5.1.1; Farnesyl diphosphate synthase; Farnesyltranstransferase; EC=2.5.1.29; Geranyltranstransferase; EC=2.5.1.10; Flags: Precursor (At4g36810)		-3.818
orange1.1t05016	K13066 caffeic acid 3-O-methyltransferase [EC:2.1.1.68] | (RefSeq) caffeic acid 3-O-methyltransferase-like isoform X2 (A)	Caffeic acid 3-O-methyltransferase; CAOMT; COMT; EC=2.1.1.68; S-adenosysl-L-methionine:caffeic acid 3-O-methyltransferase (At5g54160)	1.113	1.584
orange1.1t05018	K13066 caffeic acid 3-O-methyltransferase [EC:2.1.1.68] | (RefSeq) caffeic acid 3-O-methyltransferase-like isoform X2 (A)	Caffeic acid 3-O-methyltransferase; CAOMT; COMT; EC=2.1.1.68; S-adenosysl-L-methionine:caffeic acid 3-O-methyltransferase (At5g54160)	1.136	1.021
orange1.1t05125	K09838 zeaxanthin epoxidase [EC:1.14.15.21] | (RefSeq) Zeaxanthin epoxidase, chloroplastic (A)	Zeaxanthin epoxidase, chloroplastic; EC=1.14.15.21; Flags: Precursor (At5g11330)	3.049	
orange1.1t05216	K13066 caffeic acid 3-O-methyltransferase [EC:2.1.1.68] | (RefSeq) caffeic acid 3-O-methyltransferase-like (A)	Flavone 3'-O-methyltransferase 1; AtOMT1; EC=2.1.1.42; Acetylserotonin O-methyltransferase OMT1 {ECO:0000305}; EC=2.1.1.4 {ECO:0000269|PubMed:25039887}; Caffeate O-methyltransferase 1; EC=2.1.1.68; Quercetin 3'-O-methyltransferase 1 (At3g53140)		-1.581
orange1.1t05232	K13066 caffeic acid 3-O-methyltransferase [EC:2.1.1.68] | (RefSeq) anthranilate N-methyltransferase-like (A)	Caffeic acid 3-O-methyltransferase; CAOMT; COMT; EC=2.1.1.68; S-adenosysl-L-methionine:caffeic acid 3-O-methyltransferase (At5g54160)	5.886	
orange1.1t05352	K09833 homogentisate phytyltransferase / homogentisate geranylgeranyltransferase [EC:2.5.1.115 2.5.1.116] | (RefSeq) hypothetical protein (A)	Umbelliferone 6-dimethylallyltransferase, chloroplastic {ECO:0000303|PubMed:24354545}; EC=2.5.1.139 {ECO:0000269|PubMed:24354545}; Flags: Precursor (At2g18950)		-1.673
orange1.1t05402	K00430 peroxidase [EC:1.11.1.7] | (RefSeq) peroxidase P7-like (A)	Peroxidase 52; Atperox P52; EC=1.11.1.7; ATP49; Flags: Precursor (At5g05340)		1.615
orange1.1t05417	K22395 cinnamyl-alcohol dehydrogenase [EC:1.1.1.195] | (RefSeq) tetrahydrocannabinolic acid synthase-like isoform X1 (A)	Berberine bridge enzyme-like 18 {ECO:0000303|PubMed:26037923}; AtBBE-like 18 {ECO:0000303|PubMed:26037923}; EC=1.1.1.- {ECO:0000250|UniProtKB:O64743}; Flags: Precursor (At4g20820)		1.699
orange1.1t05430	K00660 chalcone synthase [EC:2.3.1.74] | (RefSeq) chalcone synthase 2-like (A)	Chalcone synthase 2; EC=2.3.1.74; Naringenin-chalcone synthase 2 (At5g13930)	4.664	

orange1.1t05518	K21374 7-deoxyloganetin glucosyltransferase [EC:2.4.1.324] | (RefSeq) 7-deoxyloganetin glucosyltransferase-like (A)	7-deoxyloganetin glucosyltransferase; EC=2.4.1.324; Genipin glucosyltransferase; UDP-glucose glucosyltransferase 2; GjUGT2; UDP-glycosyltransferase 85A24 (At1g22360)		-2.589
orange1.1t05631	K08081 tropinone reductase I [EC:1.1.1.206] | (RefSeq) tropinone reductase homolog At2g29170-like (A)	Tropinone reductase homolog At2g29360 {ECO:0000305}; EC=1.1.1.- {ECO:0000305} (At2g29360)		3.036
orange1.1t05709	K11517 (S)-2-hydroxy-acid oxidase [EC:1.1.3.15] | (RefSeq) hypothetical protein (A)	Peroxisomal (S)-2-hydroxy-acid oxidase GLO4; EC=1.1.3.15; Glycolate oxidase 4; AtGLO4; GOX 4; Short chain alpha-hydroxy acid oxidase GLO4 (At3g14150)	1.338	1.244
Phenylpropanoid biosynthesis [ko00940; P < 0.0001 (P3CR vs. P3R) and 0.0001 (P5CR vs. P5R)]				
Cs1g03780	K13066 caffeic acid 3-O-methyltransferase [EC:2.1.1.68] | (RefSeq) caffeic acid 3-O-methyltransferase-like isoform X1 (A)	Caffeic acid 3-O-methyltransferase; CAOMT; COMT; EC=2.1.1.68; S-adenosysl-L-methionine:caffeic acid 3-O-methyltransferase (At5g54160)		1.270
Cs1g03840	K22395 cinnamyl-alcohol dehydrogenase [EC:1.1.1.195] | (RefSeq) tetrahydrocannabinolic acid synthase-like isoform X1 (A)	Tetrahydrocannabinolic acid synthase; THCAS; EC=1.21.3.7; Delta(1)-tetrahydrocannabinolic acid synthase; THCA synthase; Flags: Precursor (At5g44440)		-1.588
Cs1g04260	K13065 shikimate O-hydroxycinnamoyltransferase [EC:2.3.1.133] | (RefSeq) vinorine synthase-like (A)	Salutaridinol 7-O-acetyltransferase {ECO:0000303|PubMed:11404355}; salAT {ECO:0000303|PubMed:11404355}; EC=2.3.1.150 {ECO:0000269|PubMed:11404355, ECO:0000269|PubMed:8537369} (At3g26040)		2.369
Cs1g04290	K13065 shikimate O-hydroxycinnamoyltransferase [EC:2.3.1.133] | (RefSeq) POPTRDRAFT_818939; hypothetical protein (A)	Salutaridinol 7-O-acetyltransferase {ECO:0000303|PubMed:11404355}; salAT {ECO:0000303|PubMed:11404355}; EC=2.3.1.150 {ECO:0000269|PubMed:11404355, ECO:0000269|PubMed:8537369} (At3g26040)		1.260
Cs1g04300	K13065 shikimate O-hydroxycinnamoyltransferase [EC:2.3.1.133] | (RefSeq) POPTRDRAFT_818939; hypothetical protein (A)	Salutaridinol 7-O-acetyltransferase {ECO:0000303|PubMed:11404355}; salAT {ECO:0000303|PubMed:11404355}; EC=2.3.1.150 {ECO:0000269|PubMed:11404355, ECO:0000269|PubMed:8537369} (At3g26040)		1.149
Cs1g04910	K00083 cinnamyl-alcohol dehydrogenase [EC:1.1.1.195] | (RefSeq) hypothetical protein (A)	Probable cinnamyl alcohol dehydrogenase 9; AtCAD9; EC=1.1.1.195 (At4g39330)	-2.173	
Cs1g05810	K13065 shikimate O-hydroxycinnamoyltransferase [EC:2.3.1.133] |	Shikimate O-hydroxycinnamoyltransferase; EC=2.3.1.133;		-1.040

	(RefSeq) shikimate O-hydroxycinnamoyltransferase-like (A)	Hydroxycinnamoyl transferase; Hydroxycinnamoyl-Coenzyme A shikimate/quinate hydroxycinnamoyltransferase (At5g48930)		
Cs1g05840	K13065 shikimate O-hydroxycinnamoyltransferase [EC:2.3.1.133] | (RefSeq) vinorine synthase-like (A)	Salutaridinol 7-O-acetyltransferase {ECO:0000303|PubMed:11404355}; salAT {ECO:0000303|PubMed:11404355}; EC=2.3.1.150 {ECO:0000269|PubMed:11404355, ECO:0000269|PubMed:8537369} (At3g26040)		1.514
Cs1g05930	K13065 shikimate O-hydroxycinnamoyltransferase [EC:2.3.1.133] | (RefSeq) vinorine synthase-like (A)	Salutaridinol 7-O-acetyltransferase {ECO:0000303|PubMed:11404355}; salAT {ECO:0000303|PubMed:11404355}; EC=2.3.1.150 {ECO:0000269|PubMed:11404355, ECO:0000269|PubMed:8537369} (At3g26040)		-1.445
Cs1g06790	K05350 beta-glucosidase [EC:3.2.1.21] | (RefSeq) beta-glucosidase 18-like (A)	Beta-glucosidase 18; Os4bglu18; EC=3.2.1.21; Flags: Precursor (At4g21760)		1.146
Cs1g09020	K13065 shikimate O-hydroxycinnamoyltransferase [EC:2.3.1.133] | (RefSeq) vinorine synthase-like (A)	Salutaridinol 7-O-acetyltransferase {ECO:0000303|PubMed:11404355}; salAT {ECO:0000303|PubMed:11404355}; EC=2.3.1.150 {ECO:0000269|PubMed:11404355, ECO:0000269|PubMed:8537369} (At3g26040)		1.142
Cs1g20580	K00083 cinnamyl-alcohol dehydrogenase [EC:1.1.1.195] | (RefSeq) probable mannitol dehydrogenase (A)	Probable mannitol dehydrogenase; EC=1.1.1.255; NAD-dependent mannitol dehydrogenase (At4g37990)		-1.691
Cs1g20610	K00083 cinnamyl-alcohol dehydrogenase [EC:1.1.1.195] | (RefSeq) probable mannitol dehydrogenase (A)	Probable mannitol dehydrogenase; EC=1.1.1.255; NAD-dependent mannitol dehydrogenase (At4g37990)	1.280	
Cs1g22960	K00430 peroxidase [EC:1.11.1.7] | (RefSeq) peroxidase 10-like (A)	Peroxidase 10; Atperox P10; EC=1.11.1.7; ATP5a; Flags: Precursor (At1g49570)		1.690
Cs1g23710	K12356 coniferyl-alcohol glucosyltransferase [EC:2.4.1.111] | (RefSeq) hypothetical protein (A)	Anthocyanidin 3-O-glucosyltransferase 5; EC=2.4.1.115; Flavonol 3-O-glucosyltransferase 5; UDP-glucose flavonoid 3-O-glucosyltransferase 5 (At2g18570)		1.267
Cs1g23720	K12356 coniferyl-alcohol glucosyltransferase [EC:2.4.1.111] | (RefSeq) hypothetical protein (A)	UDP-glycosyltransferase 72E1; EC=2.4.1.- (At5g66690)		1.614
Cs1g25930	K00430 peroxidase [EC:1.11.1.7] | (RefSeq) peroxidase 64-like (A)	Peroxidase 64; Atperox P64; EC=1.11.1.7; ATP17a; PRXR4; Flags: Precursor (At5g42180)		2.616
Cs2g05220	K00430 peroxidase [EC:1.11.1.7] | (RefSeq) peroxidase 19 (A)	Peroxidase 19; Atperox P19; EC=1.11.1.7; ATP51; Flags: Precursor (At2g34060)		1.187

Cs2g09200	K00430 peroxidase [EC:1.11.1.7] | (RefSeq) cationic peroxidase 1-like (A)	Cationic peroxidase 1; EC=1.11.1.7; PNPC1; Flags: Precursor (At5g05340)	5.690	
Cs2g09210	K00430 peroxidase [EC:1.11.1.7] | (RefSeq) peroxidase P7-like (A)	Peroxidase 52; Atperox P52; EC=1.11.1.7; ATP49; Flags: Precursor (At5g05340)		1.669
Cs2g09310	K00430 peroxidase [EC:1.11.1.7] | (RefSeq) peroxidase 4 (A)	Peroxidase 4 {ECO:0000250|UniProtKB:Q42578}; EC=1.11.1.7; Flags: Precursor (At5g58400)		1.590
Cs2g10070	K22395 cinnamyl-alcohol dehydrogenase [EC:1.1.1.195] | (RefSeq) flavin-dependent oxidoreductase FOX2 (A)	Berberine bridge enzyme-like 15 {ECO:0000303|PubMed:26037923}; AtBBE-like 15 {ECO:0000303|PubMed:26037923}; EC=1.1.1.194 {ECO:0000269|PubMed:26037923}; EC=1.1.1.195 {ECO:0000269|PubMed:26037923}; Protein EMBRYO SAC DEVELOPMENT ARREST 28 {ECO:0000303|PubMed:15634699}; Protein MATERNAL EFFECT EMBRYO ARREST 23 {ECO:0000303|PubMed:15634699}; Flags: Precursor (At2g34790)		2.378
Cs2g10110	K22395 cinnamyl-alcohol dehydrogenase [EC:1.1.1.195] | (RefSeq) tetrahydrocannabinolic acid synthase-like isoform X1 (A)	Cannabidiolic acid synthase-like 1; Flags: Precursor (At5g44440)		2.139
Cs2g10120	K22395 cinnamyl-alcohol dehydrogenase [EC:1.1.1.195] | (RefSeq) tetrahydrocannabinolic acid synthase-like isoform X1 (A)	Cannabidiolic acid synthase; EC=1.21.3.8; CBDA synthase; Flags: Precursor (At4g20820)		1.930
Cs2g10130	K22395 cinnamyl-alcohol dehydrogenase [EC:1.1.1.195] | (RefSeq) tetrahydrocannabinolic acid synthase-like (A)	Berberine bridge enzyme-like 28 {ECO:0000303|PubMed:26037923}; AtBBE-like 28 {ECO:0000303|PubMed:26037923}; EC=1.1.1.- {ECO:0000250|UniProtKB:O64743}; Flags: Precursor (At5g44440)	2.744	1.412
Cs2g10140	K22395 cinnamyl-alcohol dehydrogenase [EC:1.1.1.195] | (RefSeq) tetrahydrocannabinolic acid synthase-like isoform X1 (A)	Berberine bridge enzyme-like 18 {ECO:0000303|PubMed:26037923}; AtBBE-like 18 {ECO:0000303|PubMed:26037923}; EC=1.1.1.- {ECO:0000250|UniProtKB:O64743}; Flags: Precursor (At4g20820)	2.488	
Cs2g10150	K22395 cinnamyl-alcohol dehydrogenase [EC:1.1.1.195] | (RefSeq) tetrahydrocannabinolic acid synthase-like isoform X1 (A)	Berberine bridge enzyme-like 18 {ECO:0000303|PubMed:26037923}; AtBBE-like 18 {ECO:0000303|PubMed:26037923}; EC=1.1.1.- {ECO:0000250|UniProtKB:O64743}; Flags: Precursor (At4g20820)	6.179	
Cs2g10160	K22395 cinnamyl-alcohol dehydrogenase [EC:1.1.1.195] | (RefSeq) tetrahydrocannabinolic acid synthase-like isoform X1 (A)	Berberine bridge enzyme-like 18 {ECO:0000303|PubMed:26037923}; AtBBE-like 18 {ECO:0000303|PubMed:26037923}; EC=1.1.1.- {ECO:0000250|UniProtKB:O64743}; Flags: Precursor (At4g20820)		1.881

Cs2g10200	K22395 cinnamyl-alcohol dehydrogenase [EC:1.1.1.195] | (RefSeq) tetrahydrocannabinolic acid synthase-like isoform X1 (A)	Cannabidiolic acid synthase; EC=1.21.3.8; CBDA synthase; Flags: Precursor (At5g44440)	2.425	
Cs2g10210	K22395 cinnamyl-alcohol dehydrogenase [EC:1.1.1.195] | (RefSeq) tetrahydrocannabinolic acid synthase-like isoform X1 (A)	Cannabidiolic acid synthase-like 1; Flags: Precursor (At4g20820)	2.802	
Cs2g10220	K22395 cinnamyl-alcohol dehydrogenase [EC:1.1.1.195] | (RefSeq) berberine bridge enzyme-like 13 (A)	Berberine bridge enzyme-like 8 {ECO:0000303|PubMed:26037923}; AtBBE-like 8 {ECO:0000303|PubMed:26037923}; EC=1.1.1.- {ECO:0000250|UniProtKB:O64743}; Flags: Precursor (At1g30700)	2.826	
Cs2g10230	K22395 cinnamyl-alcohol dehydrogenase [EC:1.1.1.195] | (RefSeq) berberine bridge enzyme-like 26 (A)	Berberine bridge enzyme-like 14 {ECO:0000303|PubMed:26037923}; AtBBE-like 14 {ECO:0000303|PubMed:26037923}; EC=1.1.1.- {ECO:0000250|UniProtKB:O64743}; Flags: Precursor (At1g34570)	1.864	1.297
Cs2g17520	K13065 shikimate O-hydroxycinnamoyltransferase [EC:2.3.1.133] | (RefSeq) vinorine synthase-like (A)	Salutaridinol 7-O-acetyltransferase {ECO:0000303|PubMed:11404355}; salAT {ECO:0000303|PubMed:11404355}; EC=2.3.1.150 {ECO:0000269|PubMed:11404355, ECO:0000269|PubMed:8537369} (At3g26040)		-8.742
Cs2g21810	K00430 peroxidase [EC:1.11.1.7] | (RefSeq) hypothetical protein (A)	Peroxidase 27; Atperox P27; EC=1.11.1.7; ATP12a; PRXR7; Flags: Precursor (At3g01190)		1.538
Cs2g25450	K00430 peroxidase [EC:1.11.1.7] | (RefSeq) peroxidase 55-like (A)	Peroxidase 55; Atperox P55; EC=1.11.1.7; ATP20a; Flags: Precursor (At5g14130)	1.229	
Cs2g28370	K23260 scopoletin glucosyltransferase [EC:2.4.1.128] | (RefSeq) scopoletin glucosyltransferase-like (A)	UDP-glycosyltransferase 90A1; EC=2.4.1.- (At2g16890)	1.726	1.956
Cs2g30460	K09753 cinnamoyl-CoA reductase [EC:1.2.1.44] | (RefSeq) cinnamoyl-CoA reductase 1-like isoform X1 (A)	Cinnamoyl-CoA reductase 1; AtCCR1; EC=1.2.1.44; Protein IRREGULAR XYLEM 4 (At5g19440)	2.798	
Cs2g30470	K09753 cinnamoyl-CoA reductase [EC:1.2.1.44] | (RefSeq) cinnamoyl-CoA reductase 2-like (A)	Cinnamoyl-CoA reductase 1; AtCCR1; EC=1.2.1.44; Protein IRREGULAR XYLEM 4 (At1g51410)		-1.535
Cs3g02270	K00430 peroxidase [EC:1.11.1.7] | (RefSeq) lignin-forming anionic peroxidase-like (A)	Lignin-forming anionic peroxidase; EC=1.11.1.7; Flags: Precursor (At1g14550)		1.775
Cs3g02280	K00430 peroxidase [EC:1.11.1.7] | (RefSeq) lignin-forming anionic peroxidase-like (A)	Lignin-forming anionic peroxidase; EC=1.11.1.7; Flags: Precursor (At1g14550)		1.108
Cs3g03590	K22395 cinnamyl-alcohol dehydrogenase [EC:1.1.1.195] | (RefSeq) inactive tetrahydrocannabinolic acid synthase-like (A)	Cannabidiolic acid synthase-like 2; Flags: Precursor (At4g20860)	2.989	2.112
Cs3g03970	K13065 shikimate O-hydroxycinnamoyltransferase [EC:2.3.1.133] |	BAHD acyltransferase At5g47980; EC=2.3.1.- (At3g26040)		-1.270

	(RefSeq) hypothetical protein (A)			
Cs3g08510	K01904 4-coumarate CoA ligase [EC:6.2.1.12] | (RefSeq) hypothetical protein (A)	4-coumarateCoA ligase-like 6; EC=6.2.1.-; 4-coumarateCoA ligase isoform 7; At4CL7 (At4g19010)	-1.625	
Cs3g21730	K00430 peroxidase [EC:1.11.1.7] | (RefSeq) peroxidase 12 (A)	Peroxidase 12; Atperox P12; EC=1.11.1.7; ATP4a; PRXR6; Flags: Precursor (At1g71695)		-1.066
Cs3g26600	K00430 peroxidase [EC:1.11.1.7] | (RefSeq) peroxidase 64 (A)	Peroxidase 64; Atperox P64; EC=1.11.1.7; ATP17a; PRXR4; Flags: Precursor (At5g42180)		1.204
Cs3g27120	K13065 shikimate O-hydroxycinnamoyltransferase [EC:2.3.1.133] | (RefSeq) vinorine synthase-like (A)	Vinorine synthase; EC=2.3.1.160 (At3g26040)	-1.216	-3.982
Cs3g27130	K13065 shikimate O-hydroxycinnamoyltransferase [EC:2.3.1.133] | (RefSeq) vinorine synthase-like (A)	Vinorine synthase; EC=2.3.1.160 (At3g26040)		
Cs4g01930	K13065 shikimate O-hydroxycinnamoyltransferase [EC:2.3.1.133] | (RefSeq) shikimate O-hydroxycinnamoyltransferase-like (A)	Shikimate O-hydroxycinnamoyltransferase; EC=2.3.1.133; Hydroxycinnamoyl transferase; Hydroxycinnamoyl-Coenzyme A shikimate/quinate hydroxycinnamoyltransferase (At5g48930)		-1.746
Cs4g02360	K13065 shikimate O-hydroxycinnamoyltransferase [EC:2.3.1.133] | (RefSeq) shikimate O-hydroxycinnamoyltransferase-like (A)	Shikimate O-hydroxycinnamoyltransferase; EC=2.3.1.133; Hydroxycinnamoyl transferase; Hydroxycinnamoyl-Coenzyme A shikimate/quinate hydroxycinnamoyltransferase (At5g48930)	1.711	
Cs4g02380	K13065 shikimate O-hydroxycinnamoyltransferase [EC:2.3.1.133] | (RefSeq) shikimate O-hydroxycinnamoyltransferase-like (A)	Shikimate O-hydroxycinnamoyltransferase; EC=2.3.1.133; Hydroxycinnamoyl transferase; Hydroxycinnamoyl-Coenzyme A shikimate/quinate hydroxycinnamoyltransferase (At5g48930)	1.299	
Cs4g03330	K01904 4-coumarate CoA ligase [EC:6.2.1.12] | (RefSeq) hypothetical protein (A)	4-coumarateCoA ligase 1; 4CL 1; EC=6.2.1.12; 4-coumaroyl-CoA synthase 1 (At3g21240)	1.132	3.074
Cs4g03740	K00430 peroxidase [EC:1.11.1.7] | (RefSeq) hypothetical protein (A)	Peroxidase 16; Atperox P16; EC=1.11.1.7; ATP22a; Flags: Precursor (At2g18980)	-1.821	
Cs4g11970	K01188 beta-glucosidase [EC:3.2.1.21] | (RefSeq) putative beta-glucosidase 41 (A)	Putative beta-glucosidase 41; AtBGLU41; EC=3.2.1.21; Flags: Precursor (At5g54570)	2.732	3.222
Cs4g12540	K06892 feruloyl-CoA ortho-hydroxylase [EC:1.14.11.-] | (RefSeq) hypothetical protein (A)	Feruloyl CoA ortho-hydroxylase 1; EC=1.14.11.- (At3g12900)	1.199	2.220
Cs4g13640	K22395 cinnamyl-alcohol dehydrogenase [EC:1.1.1.195] | (RefSeq) uncharacterized LOC100817142 (A)	Protein EFFECTOR OF TRANSCRIPTION 2 {ECO:0000303|PubMed:17991462}; AtET2 {ECO:0000303|PubMed:17991462} (At5g56780)		1.033
Cs4g16650	K12355 coniferyl-aldehyde dehydrogenase [EC:1.2.1.68] | (RefSeq) aldehyde dehydrogenase family 2 member C4-like (A)	Aldehyde dehydrogenase family 2 member C4; EC=1.2.1.3; ALDH1a; Protein REDUCED EPIDERMAL FLUORESCENCE 1		1.128

		(At3g48000)		
Cs4g17860	K00430 peroxidase [EC:1.11.1.7] | (RefSeq) peroxidase 25 (A)	Peroxidase 25; Atperox P25; EC=1.11.1.7; Flags: Precursor (At2g41480)		-1.000
Cs5g07980	K13065 shikimate O-hydroxycinnamoyltransferase [EC:2.3.1.133] | (RefSeq) vinorine synthase-like (A)	Taxadien-5-alpha-ol O-acetyltransferase; EC=2.3.1.162; Taxa-4(20),11(12)-dien-5alpha-ol-O-acetyltransferase; Taxadienol acetyltransferase (At5g02890)	2.401	3.685
Cs5g16290	K13066 caffeic acid 3-O-methyltransferase [EC:2.1.1.68] | (RefSeq) caffeic acid 3-O-methyltransferase (A)	Caffeic acid 3-O-methyltransferase; CAOMT; COMT; EC=2.1.1.68; S-adenosysl-L-methionine:caffeic acid 3-O-methyltransferase (At5g54160)		1.277
Cs5g16860	K13066 caffeic acid 3-O-methyltransferase [EC:2.1.1.68] | (RefSeq) anthranilate N-methyltransferase-like (A)	Anthranilate N-methyltransferase; RgANMT; EC=2.1.1.111 (At5g54160)	1.856	
Cs5g18050	K13066 caffeic acid 3-O-methyltransferase [EC:2.1.1.68] | (RefSeq) caffeic acid 3-O-methyltransferase-like isoform X1 (A)	Caffeic acid 3-O-methyltransferase; CAOMT; COMT; EC=2.1.1.68; S-adenosysl-L-methionine:caffeic acid 3-O-methyltransferase (At5g54160)	2.004	
Cs5g18930	K13065 shikimate O-hydroxycinnamoyltransferase [EC:2.3.1.133] | (RefSeq) shikimate O-hydroxycinnamoyltransferase-like (A)	Shikimate O-hydroxycinnamoyltransferase; EC=2.3.1.133; Hydroxycinnamoyl transferase; Hydroxycinnamoyl-Coenzyme A shikimate/quinate hydroxycinnamoyltransferase (At5g48930)	-1.479	
Cs5g19020	K13066 caffeic acid 3-O-methyltransferase [EC:2.1.1.68] | (RefSeq) hypothetical protein (A)	Caffeic acid 3-O-methyltransferase; CAOMT; COMT; EC=2.1.1.68; S-adenosysl-L-methionine:caffeic acid 3-O-methyltransferase (At5g54160)	1.778	1.136
Cs5g23280	K00430 peroxidase [EC:1.11.1.7] | (RefSeq) hypothetical protein (A)	Probable peroxidase 61; Atperox P61; EC=1.11.1.7; Flags: Precursor (At5g24070)	-1.229	
Cs5g24450	K13065 shikimate O-hydroxycinnamoyltransferase [EC:2.3.1.133] | (RefSeq) vinorine synthase-like (A)	Vinorine synthase; EC=2.3.1.160 (At3g26040)	2.124	
Cs5g24470	K13065 shikimate O-hydroxycinnamoyltransferase [EC:2.3.1.133] | (RefSeq) vinorine synthase-like (A)	Vinorine synthase; EC=2.3.1.160 (At3g26040)		1.489
Cs5g24950	K13066 caffeic acid 3-O-methyltransferase [EC:2.1.1.68] | (RefSeq) hypothetical protein (A)	Anthranilate N-methyltransferase; RgANMT; EC=2.1.1.111 (At5g54160)	1.386	1.722
Cs5g24960	K13066 caffeic acid 3-O-methyltransferase [EC:2.1.1.68] | (RefSeq) anthranilate N-methyltransferase-like (A)	Anthranilate N-methyltransferase; RgANMT; EC=2.1.1.111 (At5g54160)		2.259
Cs5g24990	K13066 caffeic acid 3-O-methyltransferase [EC:2.1.1.68] | (RefSeq) hypothetical protein (A)	Anthranilate N-methyltransferase; RgANMT; EC=2.1.1.111 (At5g54160)	2.543	
Cs5g25000	K13066 caffeic acid 3-O-methyltransferase [EC:2.1.1.68] | (RefSeq)	Caffeic acid 3-O-methyltransferase; CAOMT; COMT; EC=2.1.1.68;		1.383

	caffeic acid 3-O-methyltransferase-like (A)	S-adenosysl-L-methionine:caffeic acid 3-O-methyltransferase (At5g54160)		
Cs5g26860	K05349 beta-glucosidase [EC:3.2.1.21] | (RefSeq) beta-glucosidase BoGH3B-like (A)	Beta-D-xylosidase 3; AtBXL3; EC=3.2.1.-; Alpha-L-arabinofuranosidase; EC=3.2.1.55; Flags: Precursor (At5g20950)	2.891	2.238
Cs5g32270	K00430 peroxidase [EC:1.11.1.7] | (RefSeq) peroxidase 3 (A)	Peroxidase 3; Atperox P3; EC=1.11.1.7; ATPRC; RCI3A; Rare cold-inducible protein; Flags: Precursor (At1g05260)		-1.201
Cs6g09680	K00430 peroxidase [EC:1.11.1.7] | (RefSeq) cationic peroxidase 1-like (A)	Cationic peroxidase 1; EC=1.11.1.7; PNPC1; Flags: Precursor (At5g05340)		1.111
Cs6g11950	K10775 phenylalanine ammonia-lyase [EC:4.3.1.24] | (RefSeq) phenylalanine ammonia-lyase (A)	Phenylalanine ammonia-lyase; EC=4.3.1.24 (At2g37040)		-1.149
Cs6g16650	K09754 coumaroylquinate(coumaroylshikimate) 3'-monooxygenase [EC:1.14.13.36] | (RefSeq) cytochrome P450 98A2 (A)	Cytochrome P450 98A2; EC=1.14.-.- (At2g40890)	1.097	1.588
Cs6g17140	K13065 shikimate O-hydroxycinnamoyltransferase [EC:2.3.1.133] | (RefSeq) hypothetical protein (A)	Vinorine synthase; EC=2.3.1.160 (At3g26040)		1.244
Cs6g17850	K05350 beta-glucosidase [EC:3.2.1.21] | (RefSeq) hypothetical protein (A)	Beta-glucosidase 44; AtBGLU44; EC=3.2.1.21; Flags: Precursor (At3g18080)		-2.746
Cs6g20950	K13066 caffeic acid 3-O-methyltransferase [EC:2.1.1.68] | (RefSeq) anthranilate N-methyltransferase-like (A)	Anthranilate N-methyltransferase; RgANMT; EC=2.1.1.111 (At5g54160)	5.384	2.730
Cs7g01380	K01188 beta-glucosidase [EC:3.2.1.21] | (RefSeq) beta-glucosidase 17-like (A)	Beta-glucosidase 17; AtBGLU17; EC=3.2.1.21; Flags: Precursor (At2g44480)	1.090	1.679
Cs7g10100	K13065 shikimate O-hydroxycinnamoyltransferase [EC:2.3.1.133] | (RefSeq) vinorine synthase (A)	Vinorine synthase; EC=2.3.1.160 (At1g24430)	2.509	4.511
Cs7g10110	K13065 shikimate O-hydroxycinnamoyltransferase [EC:2.3.1.133] | (RefSeq) BAHD acyltransferase At5g47980-like (A)	BAHD acyltransferase At5g47980; EC=2.3.1.- (At3g26040)		-1.475
Cs7g10780	K13065 shikimate O-hydroxycinnamoyltransferase [EC:2.3.1.133] | (RefSeq) shikimate O-hydroxycinnamoyltransferase-like (A)	(At1g27380)		-1.156
Cs7g12080	K23260 scopoletin glucosyltransferase [EC:2.4.1.128] | (RefSeq) scopoletin glucosyltransferase-like (A)	UDP-glycosyltransferase 89A2; EC=2.4.1.- (At5g03490)		-1.248
Cs7g12370	K00430 peroxidase [EC:1.11.1.7] | (RefSeq) peroxidase 11 (A)	Peroxidase 11; Atperox P11; EC=1.11.1.7; ATP23a/ATP23b; Flags: Precursor (At1g68850)	1.028	4.241
Cs7g25010	K13066 caffeic acid 3-O-methyltransferase [EC:2.1.1.68] | (RefSeq) COMT1-2; caffeic acid O-methyltransferase (A)	Caffeic acid 3-O-methyltransferase; CAOMT; COMT; EC=2.1.1.68; S-adenosysl-L-methionine:caffeic acid 3-O-methyltransferase		-1.955

		(At3g53140)		
Cs7g25030	K10775 phenylalanine ammonia-lyase [EC:4.3.1.24] | (RefSeq) phenylalanine ammonia-lyase (A)	Phenylalanine ammonia-lyase; EC=4.3.1.24; Flags: Fragment (At3g53260)		-2.319
Cs7g25540	K13066 caffeic acid 3-O-methyltransferase [EC:2.1.1.68] | (RefSeq) COMT1-2; caffeic acid O-methyltransferase (A)	Caffeic acid 3-O-methyltransferase; CAOMT; COMT; EC=2.1.1.68; S-adenosysl-L-methionine:caffeic acid 3-O-methyltransferase (At3g53140)	-1.256	-1.457
Cs7g29080	K13065 shikimate O-hydroxycinnamoyltransferase [EC:2.3.1.133] | (RefSeq) shikimate O-hydroxycinnamoyltransferase-like (A)	Shikimate O-hydroxycinnamoyltransferase; EC=2.3.1.133; Hydroxycinnamoyl transferase; Hydroxycinnamoyl-Coenzyme A shikimate/quinate hydroxycinnamoyltransferase (At5g48930)		-1.346
Cs8g05280	K23260 scopoletin glucosyltransferase [EC:2.4.1.128] | (RefSeq) scopoletin glucosyltransferase-like (A)	Scopoletin glucosyltransferase; EC=2.4.1.128; Phenylpropanoid:glucosyltransferase 1 (At2g15480)	2.210	1.208
Cs8g05300	K23260 scopoletin glucosyltransferase [EC:2.4.1.128] | (RefSeq) scopoletin glucosyltransferase (A)	Scopoletin glucosyltransferase; EC=2.4.1.128; Phenylpropanoid:glucosyltransferase 1 (At2g15480)	1.013	
Cs8g13800	K05349 beta-glucosidase [EC:3.2.1.21] | (RefSeq) beta-glucosidase BoGH3B-like (A)	Beta-xylosidase/alpha-L-arabinofuranosidase 2 {ECO:0000303|PubMed:17615411, ECO:0000312|EMBL:ABQ45228.1}; Xylan 1,4-beta-xylosidase/Alpha-L-arabinofuranosidase 2; MsXyl2 {ECO:0000303|PubMed:17615411}; Includes: Beta-xylosidase; EC=3.2.1.37; 1,4-beta-D-xylan xylohydrolase {ECO:0000250|UniProtKB:P48792}; Xylan 1,4-beta-xylosidase; Includes: Alpha-L-arabinofuranosidase; Arabinosidase {ECO:0000250|UniProtKB:P48792}; EC=3.2.1.55; Flags: Precursor (At5g20950)		-1.094
Cs8g15140	K22395 cinnamyl-alcohol dehydrogenase [EC:1.1.1.195] | (RefSeq) tetrahydrocannabinolic acid synthase-like (A)	Berberine bridge enzyme-like 5 {ECO:0000303|PubMed:26037923}; AtBBE-like 5 {ECO:0000303|PubMed:26037923}; EC=1.1.1.- {ECO:0000250|UniProtKB:O64743}; Flavin-dependent oxidoreductase FOX3 {ECO:0000303|PubMed:26352477}; EC=1.-.-.- {ECO:0000305}; Flags: Precursor (At1g26400)		-3.348
Cs8g16290	K10775 phenylalanine ammonia-lyase [EC:4.3.1.24] | (RefSeq) phenylalanine ammonia-lyase-like (A)	Phenylalanine ammonia-lyase 1; EC=4.3.1.24 (At2g37040)		2.899
Cs9g02930	K06892 feruloyl-CoA ortho-hydroxylase [EC:1.14.11.-] | (RefSeq) feruloyl CoA ortho-hydroxylase 1 (A)	Feruloyl CoA ortho-hydroxylase 1; EC=1.14.11.- (At3g13610)	2.493	1.665
Cs9g05130	K00430 peroxidase [EC:1.11.1.7] | (RefSeq) peroxidase 3-like (A)	Peroxidase 3; Atperox P3; EC=1.11.1.7; ATPRC; RCI3A; Rare		1.261

		cold-inducible protein; Flags: Precursor (At1g05260)		
Cs9g08970	K13065 shikimate O-hydroxycinnamoyltransferase [EC:2.3.1.133] | (RefSeq) salutaridinol 7-O-acetyltransferase-like (A)	Salutaridinol 7-O-acetyltransferase {ECO:0000303|PubMed:11404355}; salAT {ECO:0000303|PubMed:11404355}; EC=2.3.1.150 {ECO:0000269|PubMed:11404355, ECO:0000269|PubMed:8537369} (At3g26040)		1.363
Cs9g14500	K06892 feruloyl-CoA ortho-hydroxylase [EC:1.14.11.-] | (RefSeq) feruloyl CoA ortho-hydroxylase 1 (A)	Protein DMR6-LIKE OXYGENASE 2 {ECO:0000303|PubMed:25376907}; EC=1.14.11.- {ECO:0000255|PROSITE-ProRule:PRU00805}; 2-oxoglutarate (2OG)-Fe(II) oxygenase-like protein DLO2 {ECO:0000303|PubMed:25376907}; Salicylate 3-hydroxylase DLO2 {ECO:0000305}; S3H DLO2 {ECO:0000305}; SA 3-hydroxylase DLO2 {ECO:0000305}; Salicylic acid 3-hydroxylase DLO2 {ECO:0000305}; EC=1.14.13.- {ECO:0000250|UniProtKB:Q9ZSA8} (At4g10490)	5.040	
Cs9g15750	K22395 cinnamyl-alcohol dehydrogenase [EC:1.1.1.195] | (RefSeq) tetrahydrocannabinolic acid synthase-like (A)	Berberine bridge enzyme-like 15 {ECO:0000303|PubMed:26037923}; AtBBE-like 15 {ECO:0000303|PubMed:26037923}; EC=1.1.1.194 {ECO:0000269|PubMed:26037923}; EC=1.1.1.195 {ECO:0000269|PubMed:26037923}; Protein EMBRYO SAC DEVELOPMENT ARREST 28 {ECO:0000303|PubMed:15634699}; Protein MATERNAL EFFECT EMBRYO ARREST 23 {ECO:0000303|PubMed:15634699}; Flags: Precursor (At2g34790)	2.824	4.932
Cs9g15760	K22395 cinnamyl-alcohol dehydrogenase [EC:1.1.1.195] | (RefSeq) flavin-dependent oxidoreductase FOX2-like (A)	Berberine bridge enzyme-like 13 {ECO:0000303|PubMed:26037923}; AtBBE-like 13 {ECO:0000303|PubMed:26037923}; EC=1.1.1.194 {ECO:0000269|PubMed:26037923}; EC=1.1.1.195 {ECO:0000269|PubMed:26037923}; Flags: Precursor (At1g30760)	2.004	
Cs9g16590	K00430 peroxidase [EC:1.11.1.7] | (RefSeq) peroxidase 66 (A)	Peroxidase 66; Atperox P66; EC=1.11.1.7; ATP27a; Flags: Precursor (At5g51890)	2.440	5.362
Cs9g17850	K22395 cinnamyl-alcohol dehydrogenase [EC:1.1.1.195] | (RefSeq) tetrahydrocannabinolic acid synthase-like isoform X1 (A)	Berberine bridge enzyme-like 5 {ECO:0000303|PubMed:26037923}; AtBBE-like 5 {ECO:0000303|PubMed:26037923}; EC=1.1.1.- {ECO:0000250|UniProtKB:O64743}; Flavin-dependent		-1.848

		oxidoreductase FOX3 {ECO:0000303|PubMed:26352477}; EC=1.-.-.- {ECO:0000305}; Flags: Precursor (At1g26400)		
Cs9g19030	K13065 shikimate O-hydroxycinnamoyltransferase [EC:2.3.1.133] | (RefSeq) hypothetical protein (A)	BAHD acyltransferase At5g47980; EC=2.3.1.- (At3g26040)		1.030
Cs9g19490	K22395 cinnamyl-alcohol dehydrogenase [EC:1.1.1.195] | (RefSeq) tetrahydrocannabinolic acid synthase-like isoform X1 (A)	Berberine bridge enzyme-like 5 {ECO:0000303|PubMed:26037923}; AtBBE-like 5 {ECO:0000303|PubMed:26037923}; EC=1.1.1.- {ECO:0000250|UniProtKB:O64743}; Flavin-dependent oxidoreductase FOX3 {ECO:0000303|PubMed:26352477}; EC=1.-.-.- {ECO:0000305}; Flags: Precursor (At1g26400)		-1.240
novel.1439	K09753 cinnamoyl-CoA reductase [EC:1.2.1.44] | (RefSeq) cinnamoyl-CoA reductase 1-like isoform X1 (A)	POLR1_ARATH Retrovirus-related Pol polyprotein from transposon RE1; Retro element 1 {ECO:0000303|PubMed:10689195}; AtRE1 {ECO:0000303|PubMed:10689195}; Includes: Protease RE1; EC=3.4.23.-; Includes: Reverse transcriptase RE1; EC=2.7.7.49; Includes: Endonuclease RE1 (At1g44510)	2.066	2.170
novel.1919	K05349 beta-glucosidase [EC:3.2.1.21] | (RefSeq) lysosomal beta glucosidase-like (A)	POLR2_ARATH Retrovirus-related Pol polyprotein from transposon RE2; Retro element 2 {ECO:0000303|PubMed:10689195}; AtRE2 {ECO:0000303|PubMed:10689195}; Includes: Protease RE2; EC=3.4.23.-; Includes: Reverse transcriptase RE2; EC=2.7.7.49; Includes: Endonuclease RE2 (At4g16020)	-1.420	
novel.1945	K22395 cinnamyl-alcohol dehydrogenase [EC:1.1.1.195] | (RefSeq) flavin-dependent oxidoreductase FOX2-like (A)	BBE15_ARATH Berberine bridge enzyme-like 15 {ECO:0000303|PubMed:26037923}; AtBBE-like 15 {ECO:0000303|PubMed:26037923}; EC=1.1.1.194 {ECO:0000269|PubMed:26037923}; EC=1.1.1.195 {ECO:0000269|PubMed:26037923}; Protein EMBRYO SAC DEVELOPMENT ARREST 28 {ECO:0000303|PubMed:15634699}; Protein MATERNAL EFFECT EMBRYO ARREST 23 {ECO:0000303|PubMed:15634699}; Flags: Precursor (At2g34790)		1.427
novel.210	K12356 coniferyl-alcohol glucosyltransferase [EC:2.4.1.111] | (RefSeq) anthocyanidin 3-O-glucosyltransferase 5-like (A)	UFOG5_MANES Anthocyanidin 3-O-glucosyltransferase 5; EC=2.4.1.115; Flavonol 3-O-glucosyltransferase 5; UDP-glucose flavonoid 3-O-glucosyltransferase 5 (At3g50740)		1.644
novel.2174	K09753 cinnamoyl-CoA reductase [EC:1.2.1.44] | (RefSeq) cinnamoyl-CoA reductase 1-like isoform X1 (A)	POLR1_ARATH Retrovirus-related Pol polyprotein from transposon RE1; Retro element 1 {ECO:0000303|PubMed:10689195}; AtRE1 {ECO:0000303|PubMed:10689195}; Includes: Protease RE1;	4.246	

		EC=3.4.23.-; Includes: Reverse transcriptase RE1; EC=2.7.7.49; Includes: Endonuclease RE1 (At1g27285)		
novel.2285	K13066 caffeic acid 3-O-methyltransferase [EC:2.1.1.68] | (RefSeq) COMT1-2; caffeic acid O-methyltransferase (A)	COMT2_OCIBA Caffeic acid 3-O-methyltransferase 2; CAOMT-2; COMT-2; EC=2.1.1.68; S-adenosysl-L-methionine:caffeic acid 3-O-methyltransferase 2 (At3g53140)		-1.310
novel.2508	K13066 caffeic acid 3-O-methyltransferase [EC:2.1.1.68] | (RefSeq) anthranilate N-methyltransferase-like (A)	COMT1_MEDSA Caffeic acid 3-O-methyltransferase; CAOMT; COMT; EC=2.1.1.68; S-adenosysl-L-methionine:caffeic acid 3-O-methyltransferase (At5g54160)		2.654
novel.2683	K22395 cinnamyl-alcohol dehydrogenase [EC:1.1.1.195] | (RefSeq) tetrahydrocannabinolic acid synthase-like isoform X1 (A)	BBE18_ARATH Berberine bridge enzyme-like 18 {ECO:0000303|PubMed:26037923}; AtBBE-like 18 {ECO:0000303|PubMed:26037923}; EC=1.1.1.- {ECO:0000250|UniProtKB:O64743}; Flags: Precursor (At4g20820)		2.006
novel.2758	K13065 shikimate O-hydroxycinnamoyltransferase [EC:2.3.1.133] | (RefSeq) shikimate O-hydroxycinnamoyltransferase-like (A)	HST_TOBAC Shikimate O-hydroxycinnamoyltransferase; EC=2.3.1.133; Hydroxycinnamoyl transferase; Hydroxycinnamoyl-Coenzyme A shikimate/quinate hydroxycinnamoyltransferase (At5g48930)		-1.226
novel.477	K00430 peroxidase [EC:1.11.1.7] | (RefSeq) lignin-forming anionic peroxidase-like (A)	PERX_NICSY Lignin-forming anionic peroxidase; EC=1.11.1.7; Flags: Precursor (At1g14550)	-1.786	2.002
novel.738	K13065 shikimate O-hydroxycinnamoyltransferase [EC:2.3.1.133] | (RefSeq) shikimate O-hydroxycinnamoyltransferase (A)	HST_ARATH Shikimate O-hydroxycinnamoyltransferase; EC=2.3.1.133; Hydroxycinnamoyl transferase; Hydroxycinnamoyl-Coenzyme A shikimate/quinate hydroxycinnamoyltransferase (At5g48930)		-1.997
novel.739	K13065 shikimate O-hydroxycinnamoyltransferase [EC:2.3.1.133] | (RefSeq) shikimate O-hydroxycinnamoyltransferase-like (A)	HST_ARATH Shikimate O-hydroxycinnamoyltransferase; EC=2.3.1.133; Hydroxycinnamoyl transferase; Hydroxycinnamoyl-Coenzyme A shikimate/quinate hydroxycinnamoyltransferase (At5g48930)		-1.375
novel.975	K13066 caffeic acid 3-O-methyltransferase [EC:2.1.1.68] | (RefSeq) hypothetical protein (A)	COMT1_MEDSA Caffeic acid 3-O-methyltransferase; CAOMT; COMT; EC=2.1.1.68; S-adenosysl-L-methionine:caffeic acid 3-O-methyltransferase (At5g54160)	2.726	
orange1.1t01032	K06892 feruloyl-CoA ortho-hydroxylase [EC:1.14.11.-] | (RefSeq) 2-oxoglutarate-iron(II)-dependent oxygenase (A)	Protein DMR6-LIKE OXYGENASE 1 {ECO:0000303|PubMed:25376907}; EC=1.14.11.- {ECO:0000255|PROSITE-ProRule:PRU00805}; 2-oxoglutarate (2OG)-Fe(II) oxygenase-like protein DLO1 {ECO:0000303|PubMed:25376907}; Protein		1.348

		SENESCENCE-ASSOCIATED GENE 108 {ECO:0000303|PubMed:23959884}; Salicylate 3-hydroxylase DLO1 {ECO:0000303|PubMed:23959884}; S3H DLO1 {ECO:0000303|PubMed:23959884}; SA 3-hydroxylase DLO1 {ECO:0000303|PubMed:23959884}; Salicylic acid 3-hydroxylase DLO1 {ECO:0000303|PubMed:23959884}; EC=1.14.13.- {ECO:0000269|PubMed:23959884} (At2g36690)		
orange1.1t01084	K13066 caffeic acid 3-O-methyltransferase [EC:2.1.1.68] | (Kazusa) Lj2g3v3339490.1; - (A)	Caffeic acid 3-O-methyltransferase; CAOMT; COMT; EC=2.1.1.68; S-adenosysl-L-methionine:caffeic acid 3-O-methyltransferase (At3g53140)	-1.168	-1.088
orange1.1t01340	K22395 cinnamyl-alcohol dehydrogenase [EC:1.1.1.195] | (RefSeq) berberine bridge enzyme-like 15 (A)	Berberine bridge enzyme-like 5 {ECO:0000303|PubMed:26037923}; AtBBE-like 5 {ECO:0000303|PubMed:26037923}; EC=1.1.1.- {ECO:0000250|UniProtKB:O64743}; Flavin-dependent oxidoreductase FOX3 {ECO:0000303|PubMed:26352477}; EC=1.-.-.- {ECO:0000305}; Flags: Precursor (At1g26400)		-2.274
orange1.1t01950	K22395 cinnamyl-alcohol dehydrogenase [EC:1.1.1.195] | (RefSeq) tetrahydrocannabinolic acid synthase-like isoform X1 (A)	Berberine bridge enzyme-like 18 {ECO:0000303|PubMed:26037923}; AtBBE-like 18 {ECO:0000303|PubMed:26037923}; EC=1.1.1.- {ECO:0000250|UniProtKB:O64743}; Flags: Precursor (At4g20820)	3.214	
orange1.1t01951	K22395 cinnamyl-alcohol dehydrogenase [EC:1.1.1.195] | (RefSeq) tetrahydrocannabinolic acid synthase-like isoform X1 (A)	Berberine bridge enzyme-like 18 {ECO:0000303|PubMed:26037923}; AtBBE-like 18 {ECO:0000303|PubMed:26037923}; EC=1.1.1.- {ECO:0000250|UniProtKB:O64743}; Flags: Precursor (At4g20820)	2.936	
orange1.1t01952	K22395 cinnamyl-alcohol dehydrogenase [EC:1.1.1.195] | (RefSeq) tetrahydrocannabinolic acid synthase-like isoform X1 (A)	Berberine bridge enzyme-like 18 {ECO:0000303|PubMed:26037923}; AtBBE-like 18 {ECO:0000303|PubMed:26037923}; EC=1.1.1.- {ECO:0000250|UniProtKB:O64743}; Flags: Precursor (At4g20820)	3.091	2.022
orange1.1t01953	K22395 cinnamyl-alcohol dehydrogenase [EC:1.1.1.195] | (RefSeq) tetrahydrocannabinolic acid synthase-like isoform X1 (A)	Cannabidiolic acid synthase; EC=1.21.3.8; CBDA synthase; Flags: Precursor (At4g20820)	1.149	1.281
orange1.1t01955	K22395 cinnamyl-alcohol dehydrogenase [EC:1.1.1.195] | (RefSeq) tetrahydrocannabinolic acid synthase-like isoform X1 (A)	Cannabidiolic acid synthase-like 2; Flags: Precursor (At4g20820)		-1.161
orange1.1t01957	K22395 cinnamyl-alcohol dehydrogenase [EC:1.1.1.195] | (RefSeq) tetrahydrocannabinolic acid synthase-like isoform X1 (A)	Cannabidiolic acid synthase; EC=1.21.3.8; CBDA synthase; Flags: Precursor (At4g20820)	3.258	2.267

orange1.1t02033	K00430 peroxidase [EC:1.11.1.7] | (RefSeq) peroxidase 59-like (A)	Peroxidase N; EC=1.11.1.7; Neutral peroxidase; Flags: Precursor (At5g19890)		2.207
orange1.1t02041	K00430 peroxidase [EC:1.11.1.7] | (RefSeq) peroxidase A2 (A)	Peroxidase C3; EC=1.11.1.7; Flags: Precursor (At5g06720)		-1.203
orange1.1t02045	K00430 peroxidase [EC:1.11.1.7] | (RefSeq) peroxidase 15-like (A)	Peroxidase 15 {ECO:0000303|PubMed:17936696}; Prx15 {ECO:0000303|PubMed:17936696}; EC=1.11.1.7; Anionic peroxidase {ECO:0000303|PubMed:17936696}; Flags: Precursor (At5g06720)	-1.018	
orange1.1t02059	K00430 peroxidase [EC:1.11.1.7] | (RefSeq) peroxidase A2 (A)	Lignin-forming anionic peroxidase; EC=1.11.1.7; TOPA; Flags: Precursor (At5g06720)		1.147
orange1.1t02085	K13066 caffeic acid 3-O-methyltransferase [EC:2.1.1.68] | (RefSeq) caffeic acid 3-O-methyltransferase-like isoform X1 (A)	Caffeic acid 3-O-methyltransferase 1; CAOMT-1; COMT-1; EC=2.1.1.68; S-adenosysl-L-methionine:caffeic acid 3-O-methyltransferase 1 (At5g54160)		-2.085
orange1.1t02091	K13066 caffeic acid 3-O-methyltransferase [EC:2.1.1.68] | (RefSeq) hypothetical protein (A)	Caffeic acid 3-O-methyltransferase 1; CAOMT-1; COMT-1; EC=2.1.1.68; S-adenosysl-L-methionine:caffeic acid 3-O-methyltransferase 1 (At5g54160)		-2.365
orange1.1t02225	K00430 peroxidase [EC:1.11.1.7] | (RefSeq) LOW QUALITY PROTEIN: peroxidase A2-like (A)	Peroxidase 53; Atperox P53; EC=1.11.1.7; ATPA2; Flags: Precursor (At5g06720)		1.571
orange1.1t02793	K13065 shikimate O-hydroxycinnamoyltransferase [EC:2.3.1.133] | (RefSeq) shikimate O-hydroxycinnamoyltransferase (A)	Shikimate O-hydroxycinnamoyltransferase; EC=2.3.1.133; Hydroxycinnamoyl transferase; Hydroxycinnamoyl-Coenzyme A shikimate/quinate hydroxycinnamoyltransferase (At5g48930)		1.021
orange1.1t03152	K23260 scopoletin glucosyltransferase [EC:2.4.1.128] | (RefSeq) scopoletin glucosyltransferase (A)	UDP-glycosyltransferase 92A1; EC=2.4.1.- (At5g12890)	1.121	2.980
orange1.1t03484	K13066 caffeic acid 3-O-methyltransferase [EC:2.1.1.68] | (RefSeq) hypothetical protein (A)	Anthranilate N-methyltransferase; RgANMT; EC=2.1.1.111 (At5g54160)	1.202	1.252
orange1.1t03987	K13066 caffeic acid 3-O-methyltransferase [EC:2.1.1.68] | (RefSeq) caffeic acid 3-O-methyltransferase-like (A)	Caffeic acid 3-O-methyltransferase; CAOMT; COMT; EC=2.1.1.68; S-adenosysl-L-methionine:caffeic acid 3-O-methyltransferase (At5g54160)	6.849	
orange1.1t04173	K22395 cinnamyl-alcohol dehydrogenase [EC:1.1.1.195] | (RefSeq) tetrahydrocannabinolic acid synthase-like isoform X1 (A)	Cannabidiolic acid synthase-like 2; Flags: Precursor (At4g20820)	2.399	2.417
orange1.1t04194	K13066 caffeic acid 3-O-methyltransferase [EC:2.1.1.68] | (RefSeq) caffeic acid 3-O-methyltransferase-like (A)	Caffeic acid 3-O-methyltransferase; CAOMT; COMT; EC=2.1.1.68; S-adenosysl-L-methionine:caffeic acid 3-O-methyltransferase (At5g54160)	3.478	
orange1.1t050	K13066 caffeic acid 3-O-methyltransferase [EC:2.1.1.68] | (RefSeq)	Caffeic acid 3-O-methyltransferase; CAOMT; COMT; EC=2.1.1.68;	1.113	1.584

16	caffeic acid 3-O-methyltransferase-like isoform X2 (A)	S-adenosysl-L-methionine:caffeic acid 3-O-methyltransferase (At5g54160)		
orange1.1t05018	K13066 caffeic acid 3-O-methyltransferase [EC:2.1.1.68] | (RefSeq) caffeic acid 3-O-methyltransferase-like isoform X2 (A)	Caffeic acid 3-O-methyltransferase; CAOMT; COMT; EC=2.1.1.68; S-adenosysl-L-methionine:caffeic acid 3-O-methyltransferase (At5g54160)	1.136	1.021
orange1.1t05216	K13066 caffeic acid 3-O-methyltransferase [EC:2.1.1.68] | (RefSeq) caffeic acid 3-O-methyltransferase-like (A)	Flavone 3'-O-methyltransferase 1; AtOMT1; EC=2.1.1.42; Acetylserotonin O-methyltransferase OMT1 {ECO:0000305}; EC=2.1.1.4 {ECO:0000269|PubMed:25039887}; Caffeate O-methyltransferase 1; EC=2.1.1.68; Quercetin 3'-O-methyltransferase 1 (At3g53140)		-1.581
orange1.1t05232	K13066 caffeic acid 3-O-methyltransferase [EC:2.1.1.68] | (RefSeq) anthranilate N-methyltransferase-like (A)	Caffeic acid 3-O-methyltransferase; CAOMT; COMT; EC=2.1.1.68; S-adenosysl-L-methionine:caffeic acid 3-O-methyltransferase (At5g54160)	5.886	
orange1.1t05402	K00430 peroxidase [EC:1.11.1.7] | (RefSeq) peroxidase P7-like (A)	Peroxidase 52; Atperox P52; EC=1.11.1.7; ATP49; Flags: Precursor (At5g05340)		1.615
orange1.1t05417	K22395 cinnamyl-alcohol dehydrogenase [EC:1.1.1.195] | (RefSeq) tetrahydrocannabinolic acid synthase-like isoform X1 (A)	Berberine bridge enzyme-like 18 {ECO:0000303|PubMed:26037923}; AtBBE-like 18 {ECO:0000303|PubMed:26037923}; EC=1.1.1.- {ECO:0000250|UniProtKB:O64743}; Flags: Precursor (At4g20820)		1.699
Phenol-containing compound metabolic process [GO:0018958; P = 0.0261 (P3CR vs. P3R) and 0.0641 (P5CR vs. P5R)]				
Cs1g07440		Protein GRIM REAPER {ECO:0000303|PubMed:19279211}; Stigma-specific STIG1-like protein GRI {ECO:0000303|PubMed:19279211}; Contains: GRIp {ECO:0000303|PubMed:25398910}; Flags: Precursor (At1g53130)		1.169
Cs1g12300	K18054 2'-deoxymugineic-acid 2'-dioxygenase / mugineic-acid 3-dioxygenase [EC:1.14.11.24 1.14.11.25] | (RefSeq) 2'-deoxymugineic-acid 2'-dioxygenase-like (A)	Protein DMR6-LIKE OXYGENASE 2 {ECO:0000303|PubMed:25376907}; EC=1.14.11.- {ECO:0000255|PROSITE-ProRule:PRU00805}; 2-oxoglutarate (2OG)-Fe(II) oxygenase-like protein DLO2 {ECO:0000303|PubMed:25376907}; Salicylate 3-hydroxylase DLO2 {ECO:0000305}; S3H DLO2 {ECO:0000305}; SA 3-hydroxylase DLO2 {ECO:0000305}; Salicylic acid 3-hydroxylase DLO2 {ECO:0000305}; EC=1.14.13.- {ECO:0000250|UniProtKB:Q9ZSA8} (At4g10490)	2.697	

Cs1g12310	K18054 2'-deoxymugineic-acid 2'-dioxygenase / mugineic-acid 3-dioxygenase [EC:1.14.11.24 1.14.11.25] | (RefSeq) 2'-deoxymugineic-acid 2'-dioxygenase-like (A)	Protein DMR6-LIKE OXYGENASE 1 {ECO:0000303|PubMed:25376907}; EC=1.14.11.- {ECO:0000255|PROSITE-ProRule:PRU00805}; 2-oxoglutarate (2OG)-Fe(II) oxygenase-like protein DLO1 {ECO:0000303|PubMed:25376907}; Protein SENESCENCE-ASSOCIATED GENE 108 {ECO:0000303|PubMed:23959884}; Salicylate 3-hydroxylase DLO1 {ECO:0000303|PubMed:23959884}; S3H DLO1 {ECO:0000303|PubMed:23959884}; SA 3-hydroxylase DLO1 {ECO:0000303|PubMed:23959884}; Salicylic acid 3-hydroxylase DLO1 {ECO:0000303|PubMed:23959884}; EC=1.14.13.- {ECO:0000269|PubMed:23959884} (At4g10500)		-1.609
Cs1g16160	K01535 H+-transporting ATPase [EC:3.6.3.6] | (RefSeq) hypothetical protein (A)	ATPase 10, plasma membrane-type; EC=3.6.3.6; Proton pump 10 (At1g17260)		2.076
Cs2g05610	K01183 chitinase [EC:3.2.1.14] | (RefSeq) class III chitinase (A)	MACPF domain-containing protein NSL1; Protein NECROTIC SPOTTED LESIONS 1; Protein NSL1 (At1g28380)		1.321
Cs3g08290	K21568 pinoresinol/lariciresinol reductase [EC:1.23.1.1 1.23.1.2 1.23.1.3 1.23.1.4] | (RefSeq) hypothetical protein (A)	Bifunctional pinoresinol-lariciresinol reductase 2; PLR-Lu2; (+)-lariciresinol reductase; EC=1.23.1.2; (+)-pinoresinol reductase; EC=1.23.1.1 (At1g32100)	3.685	6.902
Cs5g16310	K05278 flavonol synthase [EC:1.14.11.23] | (RefSeq) flavonol synthase/flavanone 3-hydroxylase (A)	Protein DOWNY MILDEW RESISTANCE 6 {ECO:0000303|PubMed:15986928}; AtDMR6 {ECO:0000303|PubMed:15986928}; EC=1.14.11.- {ECO:0000255|PROSITE-ProRule:PRU00805}; 2-oxoglutarate (2OG)-Fe(II) oxygenase-like protein DMR6 {ECO:0000303|PubMed:18248595}; Salicylate 3-hydroxylase DMR6 {ECO:0000305}; S3H DMR6 {ECO:0000305}; SA 3-hydroxylase DMR6 {ECO:0000305}; Salicylic acid 3-hydroxylase DMR6 {ECO:0000305}; EC=1.14.13.- {ECO:0000250|UniProtKB:Q9ZSA8} (At5g24530)		-1.118
Cs5g18300	K01381 saccharopepsin [EC:3.4.23.25] | (RefSeq) aspartic proteinase CDR1-like (A)	Aspartic proteinase CDR1; EC=3.4.23.-; Protein CONSTITUTIVE DISEASE RESISTANCE 1; Flags: Precursor (At1g64830)	4.570	1.941
Cs5g21200	K13691 pathogen-inducible salicylic acid glucosyltransferase [EC:2.4.1.-] | (RefSeq) UDP-glycosyltransferase 74F2-like (A)	UDP-glycosyltransferase 74F2; EC=2.4.1.-; AtSGT1; Salicylic acid glucosyltransferase 1 (At2g43820)		-1.633
Cs5g28710	K05282 gibberellin-44 dioxygenase [EC:1.14.11.12] | (RefSeq)	Protein DMR6-LIKE OXYGENASE 1	2.636	

	gibberellin 20 oxidase 1-D-like (A)	{ECO:0000303|PubMed:25376907}; EC=1.14.11.- {ECO:0000255|PROSITE-ProRule:PRU00805}; 2-oxoglutarate (2OG)-Fe(II) oxygenase-like protein DLO1 {ECO:0000303|PubMed:25376907}; Protein SENESCENCE-ASSOCIATED GENE 108 {ECO:0000303|PubMed:23959884}; Salicylate 3-hydroxylase DLO1 {ECO:0000303|PubMed:23959884}; S3H DLO1 {ECO:0000303|PubMed:23959884}; SA 3-hydroxylase DLO1 {ECO:0000303|PubMed:23959884}; Salicylic acid 3-hydroxylase DLO1 {ECO:0000303|PubMed:23959884}; EC=1.14.13.- {ECO:0000269|PubMed:23959884} (At3g19000)		
Cs5g28780	K05282 gibberellin-44 dioxygenase [EC:1.14.11.12] | (RefSeq) gibberellin 20 oxidase 1-like isoform X1 (A)	Protein DMR6-LIKE OXYGENASE 2 {ECO:0000303|PubMed:25376907}; EC=1.14.11.- {ECO:0000255|PROSITE-ProRule:PRU00805}; 2-oxoglutarate (2OG)-Fe(II) oxygenase-like protein DLO2 {ECO:0000303|PubMed:25376907}; Salicylate 3-hydroxylase DLO2 {ECO:0000305}; S3H DLO2 {ECO:0000305}; SA 3-hydroxylase DLO2 {ECO:0000305}; Salicylic acid 3-hydroxylase DLO2 {ECO:0000305}; EC=1.14.13.- {ECO:0000250|UniProtKB:Q9ZSA8} (At3g19000)		1.368
Cs5g29440	K16275 E3 ubiquitin-protein ligase BAH [EC:2.3.2.27] | (RefSeq) hypothetical protein (A)	E3 ubiquitin-protein ligase BAH1; EC=2.3.2.27 {ECO:0000305}; Protein BENZOIC ACID HYPERSENSITIVE 1; Protein NITROGEN LIMITATION ADAPTATION; RING-type E3 ubiquitin transferase BAH1 {ECO:0000305} (At1g02860)		1.226
Cs6g12050	K04733 interleukin-1 receptor-associated kinase 4 [EC:2.7.11.1] | (RefSeq) probable serine/threonine-protein kinase PBL19 (A)	Serine/threonine-protein kinase PCRK1 {ECO:0000305}; EC=2.7.11.1 {ECO:0000269|PubMed:25711411}; Protein PTI-COMPROMISED RECEPTOR-LIKE CYTOPLASMIC KINASE 1 {ECO:0000303|PubMed:25711411} (At3g09830)		1.519
Cs7g15810	K01381 saccharopepsin [EC:3.4.23.25] | (RefSeq) aspartic proteinase CDR1-like (A)	Aspartic proteinase CDR1; EC=3.4.23.-; Protein CONSTITUTIVE DISEASE RESISTANCE 1; Flags: Precursor (At5g33340)	3.547	
Cs7g27120	K21989 calcium permeable stress-gated cation channel | (RefSeq) CSC1-like protein RXW8 (A)	Protein SAR DEFICIENT 1 {ECO:0000303|PubMed:20921422} (At1g73800)	2.272	1.128
Cs9g14480	K00475 naringenin 3-dioxygenase [EC:1.14.11.9] | (RefSeq) naringenin,2-oxoglutarate 3-dioxygenase (A)	Protein DMR6-LIKE OXYGENASE 2 {ECO:0000303|PubMed:25376907}; EC=1.14.11.-	4.517	

		{ECO:0000255|PROSITE-ProRule:PRU00805}; 2-oxoglutarate (2OG)-Fe(II) oxygenase-like protein DLO2 {ECO:0000303|PubMed:25376907}; Salicylate 3-hydroxylase DLO2 {ECO:0000305}; S3H DLO2 {ECO:0000305}; SA 3-hydroxylase DLO2 {ECO:0000305}; Salicylic acid 3-hydroxylase DLO2 {ECO:0000305}; EC=1.14.13.- {ECO:0000250|UniProtKB:Q9ZSA8} (At4g10490)		
Cs9g14500	K06892 feruloyl-CoA ortho-hydroxylase [EC:1.14.11.-] | (RefSeq) feruloyl CoA ortho-hydroxylase 1 (A)	Protein DMR6-LIKE OXYGENASE 2 {ECO:0000303|PubMed:25376907}; EC=1.14.11.- {ECO:0000255|PROSITE-ProRule:PRU00805}; 2-oxoglutarate (2OG)-Fe(II) oxygenase-like protein DLO2 {ECO:0000303|PubMed:25376907}; Salicylate 3-hydroxylase DLO2 {ECO:0000305}; S3H DLO2 {ECO:0000305}; SA 3-hydroxylase DLO2 {ECO:0000305}; Salicylic acid 3-hydroxylase DLO2 {ECO:0000305}; EC=1.14.13.- {ECO:0000250|UniProtKB:Q9ZSA8} (At4g10490)	5.040	
Cs9g14520	K05278 flavonol synthase [EC:1.14.11.23] | (RefSeq) flavonol synthase/flavanone 3-hydroxylase (A)	Protein DMR6-LIKE OXYGENASE 2 {ECO:0000303|PubMed:25376907}; EC=1.14.11.- {ECO:0000255|PROSITE-ProRule:PRU00805}; 2-oxoglutarate (2OG)-Fe(II) oxygenase-like protein DLO2 {ECO:0000303|PubMed:25376907}; Salicylate 3-hydroxylase DLO2 {ECO:0000305}; S3H DLO2 {ECO:0000305}; SA 3-hydroxylase DLO2 {ECO:0000305}; Salicylic acid 3-hydroxylase DLO2 {ECO:0000305}; EC=1.14.13.- {ECO:0000250|UniProtKB:Q9ZSA8} (At4g10490)	6.224	
orange1.1t01032	K06892 feruloyl-CoA ortho-hydroxylase [EC:1.14.11.-] | (RefSeq) 2-oxoglutarate-iron(II)-dependent oxygenase (A)	Protein DMR6-LIKE OXYGENASE 1 {ECO:0000303|PubMed:25376907}; EC=1.14.11.- {ECO:0000255|PROSITE-ProRule:PRU00805}; 2-oxoglutarate (2OG)-Fe(II) oxygenase-like protein DLO1 {ECO:0000303|PubMed:25376907}; Protein SENESCENCE-ASSOCIATED GENE 108 {ECO:0000303|PubMed:23959884}; Salicylate 3-hydroxylase DLO1 {ECO:0000303|PubMed:23959884}; S3H DLO1 {ECO:0000303|PubMed:23959884}; SA 3-hydroxylase DLO1		1.348

		{ECO:0000303|PubMed:23959884}; Salicylic acid 3-hydroxylase DLO1 {ECO:0000303|PubMed:23959884}; EC=1.14.13.- {ECO:0000269|PubMed:23959884} (At2g36690)		
orange1.1t04550	K00451 homogentisate 1,2-dioxygenase [EC:1.13.11.5] | (RefSeq) homogentisate 1,2-dioxygenase (A)	Homogentisate 1,2-dioxygenase; EC=1.13.11.5; Homogentisate oxygenase; Homogentisic acid oxidase; Homogentisicase (At5g54080)	1.095	
Lignan biosynthetic process [GO:0009807; P = 0.0196 (P3CR vs. P3R) and 0.5514 (P5CR vs. P5R)]				
Cs2g16220	K23050 phenylcoumaran benzylic ether reductase [EC:1.3.1.-] | (RefSeq) isoflavone reductase homolog PCBER-like isoform X1 (A)	Isoflavone reductase homolog PCBER {ECO:0000305}; EC=1.3.1.- {ECO:0000269|PubMed:10066819}; Phenylcoumaran benzylic ether reductase POP1 {ECO:0000303|PubMed:10066819}; PCBER-Pop1 {ECO:0000303|PubMed:10066819} (At4g39230)	1.170	
Cs2g16260	K23050 phenylcoumaran benzylic ether reductase [EC:1.3.1.-] | (RefSeq) isoflavone reductase homolog PCBER-like (A)	Isoflavone reductase homolog PCBER {ECO:0000305}; EC=1.3.1.- {ECO:0000269|PubMed:10066819}; Phenylcoumaran benzylic ether reductase POP1 {ECO:0000303|PubMed:10066819}; PCBER-Pop1 {ECO:0000303|PubMed:10066819} (At4g39230)	1.678	
Cs3g08290	K21568 pinoresinol/lariciresinol reductase [EC:1.23.1.1 1.23.1.2 1.23.1.3 1.23.1.4] | (RefSeq) hypothetical protein (A)	Bifunctional pinoresinol-lariciresinol reductase 2; PLR-Lu2; (+)-lariciresinol reductase; EC=1.23.1.2; (+)-pinoresinol reductase; EC=1.23.1.1 (At1g32100)	3.685	6.902
Cs3g25010	K23050 phenylcoumaran benzylic ether reductase [EC:1.3.1.-] | (RefSeq) isoflavone reductase homolog TP7-like (A)	Isoflavone reductase homolog TP7 {ECO:0000305}; EC=1.3.1.- {ECO:0000269|PubMed:12369619}; Phenylcoumaran benzylic ether reductase TP7 {ECO:0000305}; NtPCBER {ECO:0000305}; Protein TOBACCO PETAL 7 {ECO:0000305} (At1g75280)	1.233	2.386
Flavonoid biosynthesis [ko00941; P = 0.0692 (P3CR vs. P3R) and < 0.0001 (P5CR vs. P5R)]				
Cs1g04260	K13065 shikimate O-hydroxycinnamoyltransferase [EC:2.3.1.133] | (RefSeq) vinorine synthase-like (A)	Salutaridinol 7-O-acetyltransferase {ECO:0000303|PubMed:11404355}; salAT {ECO:0000303|PubMed:11404355}; EC=2.3.1.150 {ECO:0000269|PubMed:11404355, ECO:0000269|PubMed:8537369} (At3g26040)		2.369
Cs1g04290	K13065 shikimate O-hydroxycinnamoyltransferase [EC:2.3.1.133] | (RefSeq) POPTRDRAFT_818939; hypothetical protein (A)	Salutaridinol 7-O-acetyltransferase {ECO:0000303|PubMed:11404355}; salAT {ECO:0000303|PubMed:11404355}; EC=2.3.1.150 {ECO:0000269|PubMed:11404355,		1.260

		ECO:0000269|PubMed:8537369} (At3g26040)		
Cs1g04300	K13065 shikimate O-hydroxycinnamoyltransferase [EC:2.3.1.133] | (RefSeq) POPTRDRAFT_818939; hypothetical protein (A)	Salutaridinol 7-O-acetyltransferase {ECO:0000303|PubMed:11404355}; salAT {ECO:0000303|PubMed:11404355}; EC=2.3.1.150 {ECO:0000269|PubMed:11404355, ECO:0000269|PubMed:8537369} (At3g26040)		1.149
Cs1g05810	K13065 shikimate O-hydroxycinnamoyltransferase [EC:2.3.1.133] | (RefSeq) shikimate O-hydroxycinnamoyltransferase-like (A)	Shikimate O-hydroxycinnamoyltransferase; EC=2.3.1.133; Hydroxycinnamoyl transferase; Hydroxycinnamoyl-Coenzyme A shikimate/quinate hydroxycinnamoyltransferase (At5g48930)		-1.040
Cs1g05840	K13065 shikimate O-hydroxycinnamoyltransferase [EC:2.3.1.133] | (RefSeq) vinorine synthase-like (A)	Salutaridinol 7-O-acetyltransferase {ECO:0000303|PubMed:11404355}; salAT {ECO:0000303|PubMed:11404355}; EC=2.3.1.150 {ECO:0000269|PubMed:11404355, ECO:0000269|PubMed:8537369} (At3g26040)		1.514
Cs1g05930	K13065 shikimate O-hydroxycinnamoyltransferase [EC:2.3.1.133] | (RefSeq) vinorine synthase-like (A)	Salutaridinol 7-O-acetyltransferase {ECO:0000303|PubMed:11404355}; salAT {ECO:0000303|PubMed:11404355}; EC=2.3.1.150 {ECO:0000269|PubMed:11404355, ECO:0000269|PubMed:8537369} (At3g26040)		-1.445
Cs1g09020	K13065 shikimate O-hydroxycinnamoyltransferase [EC:2.3.1.133] | (RefSeq) vinorine synthase-like (A)	Salutaridinol 7-O-acetyltransferase {ECO:0000303|PubMed:11404355}; salAT {ECO:0000303|PubMed:11404355}; EC=2.3.1.150 {ECO:0000269|PubMed:11404355, ECO:0000269|PubMed:8537369} (At3g26040)		1.142
Cs2g11840	K01859 chalcone isomerase [EC:5.5.1.6] | (RefSeq) vacuolar-sorting receptor 1-like (A)	Vacuolar-sorting receptor 6; AtVSR6; BP80-like protein d; AtBP80d; Epidermal growth factor receptor-like protein 6; AtELP6; Flags: Precursor (At1g30900)		1.591
Cs2g17520	K13065 shikimate O-hydroxycinnamoyltransferase [EC:2.3.1.133] | (RefSeq) vinorine synthase-like (A)	Salutaridinol 7-O-acetyltransferase {ECO:0000303|PubMed:11404355}; salAT {ECO:0000303|PubMed:11404355}; EC=2.3.1.150 {ECO:0000269|PubMed:11404355, ECO:0000269|PubMed:8537369} (At3g26040)		-8.742
Cs3g03970	K13065 shikimate O-hydroxycinnamoyltransferase [EC:2.3.1.133] | (RefSeq) hypothetical protein (A)	BAHD acyltransferase At5g47980; EC=2.3.1.- (At3g26040)		-1.270

Cs3g20330	K00660 chalcone synthase [EC:2.3.1.74] | (RefSeq) chalcone synthase-like (A)	Chalcone synthase; EC=2.3.1.74; Naringenin-chalcone synthase (At5g13930)		-1.250
Cs3g20640	K00660 chalcone synthase [EC:2.3.1.74] | (RefSeq) chalcone synthase 2-like (A)	Chalcone synthase 2; EC=2.3.1.74; Naringenin-chalcone synthase 2 (At5g13930)	4.405	
Cs3g20680	K00660 chalcone synthase [EC:2.3.1.74] | (RefSeq) chalcone synthase 2-like (A)	Chalcone synthase 2; EC=2.3.1.74; Naringenin-chalcone synthase 2 (At5g13930)	3.814	
Cs3g27120	K13065 shikimate O-hydroxycinnamoyltransferase [EC:2.3.1.133] | (RefSeq) vinorine synthase-like (A)	Vinorine synthase; EC=2.3.1.160 (At3g26040)	-1.216	-3.982
Cs4g01930	K13065 shikimate O-hydroxycinnamoyltransferase [EC:2.3.1.133] | (RefSeq) shikimate O-hydroxycinnamoyltransferase-like (A)	Shikimate O-hydroxycinnamoyltransferase; EC=2.3.1.133; Hydroxycinnamoyl transferase; Hydroxycinnamoyl-Coenzyme A shikimate/quinate hydroxycinnamoyltransferase (At5g48930)		-1.746
Cs4g02360	K13065 shikimate O-hydroxycinnamoyltransferase [EC:2.3.1.133] | (RefSeq) shikimate O-hydroxycinnamoyltransferase-like (A)	Shikimate O-hydroxycinnamoyltransferase; EC=2.3.1.133; Hydroxycinnamoyl transferase; Hydroxycinnamoyl-Coenzyme A shikimate/quinate hydroxycinnamoyltransferase (At5g48930)	1.711	
Cs4g02380	K13065 shikimate O-hydroxycinnamoyltransferase [EC:2.3.1.133] | (RefSeq) shikimate O-hydroxycinnamoyltransferase-like (A)	Shikimate O-hydroxycinnamoyltransferase; EC=2.3.1.133; Hydroxycinnamoyl transferase; Hydroxycinnamoyl-Coenzyme A shikimate/quinate hydroxycinnamoyltransferase (At5g48930)	1.299	
Cs5g07980	K13065 shikimate O-hydroxycinnamoyltransferase [EC:2.3.1.133] | (RefSeq) vinorine synthase-like (A)	Taxadien-5-alpha-ol O-acetyltransferase; EC=2.3.1.162; Taxa-4(20),11(12)-dien-5alpha-ol-O-acetyltransferase; Taxadienol acetyltransferase (At5g02890)	2.401	3.685
Cs5g11730	K05280 flavonoid 3'-monooxygenase [EC:1.14.13.21] | (RefSeq) flavonoid 3'-monooxygenase (A)	Flavonoid 3'-monooxygenase; EC=1.14.13.21; Cytochrome P450 75B2; Flavonoid 3'-hydroxylase (At5g07990)	-1.621	-4.299
Cs5g16310	K05278 flavonol synthase [EC:1.14.11.23] | (RefSeq) flavonol synthase/flavanone 3-hydroxylase (A)	Protein DOWNY MILDEW RESISTANCE 6 {ECO:0000303|PubMed:15986928}; AtDMR6 {ECO:0000303|PubMed:15986928}; EC=1.14.11.- {ECO:0000255|PROSITE-ProRule:PRU00805}; 2-oxoglutarate (2OG)-Fe(II) oxygenase-like protein DMR6 {ECO:0000303|PubMed:18248595}; Salicylate 3-hydroxylase DMR6 {ECO:0000305}; S3H DMR6 {ECO:0000305}; SA 3-hydroxylase DMR6 {ECO:0000305}; Salicylic acid 3-hydroxylase DMR6 {ECO:0000305}; EC=1.14.13.- {ECO:0000250|UniProtKB:Q9ZSA8} (At5g24530)		-1.118
Cs5g18930	K13065 shikimate O-hydroxycinnamoyltransferase [EC:2.3.1.133] | (RefSeq) shikimate O-hydroxycinnamoyltransferase-like (A)	Shikimate O-hydroxycinnamoyltransferase; EC=2.3.1.133; Hydroxycinnamoyl transferase; Hydroxycinnamoyl-Coenzyme A	-1.479	

		shikimate/quinate hydroxycinnamoyltransferase (At5g48930)		
Cs5g24450	K13065 shikimate O-hydroxycinnamoyltransferase [EC:2.3.1.133] | (RefSeq) vinorine synthase-like (A)	Vinorine synthase; EC=2.3.1.160 (At3g26040)	2.124	
Cs5g24470	K13065 shikimate O-hydroxycinnamoyltransferase [EC:2.3.1.133] | (RefSeq) vinorine synthase-like (A)	Vinorine synthase; EC=2.3.1.160 (At3g26040)		1.489
Cs6g08500	K05277 leucoanthocyanidin dioxygenase [EC:1.14.11.19] | (RefSeq) leucoanthocyanidin dioxygenase-like (A)	Probable 2-oxoglutarate-dependent dioxygenase At5g05600 {ECO:0000305}; EC=1.14.11.- {ECO:0000305} (At5g05600)		-3.896
Cs6g16650	K09754 coumaroylquinate(coumaroylshikimate) 3'-monooxygenase [EC:1.14.13.36] | (RefSeq) cytochrome P450 98A2 (A)	Cytochrome P450 98A2; EC=1.14.-.- (At2g40890)	1.097	1.588
Cs6g17140	K13065 shikimate O-hydroxycinnamoyltransferase [EC:2.3.1.133] | (RefSeq) hypothetical protein (A)	Vinorine synthase; EC=2.3.1.160 (At3g26040)		1.244
Cs7g10100	K13065 shikimate O-hydroxycinnamoyltransferase [EC:2.3.1.133] | (RefSeq) vinorine synthase (A)	Vinorine synthase; EC=2.3.1.160 (At1g24430)	2.509	4.511
Cs7g10110	K13065 shikimate O-hydroxycinnamoyltransferase [EC:2.3.1.133] | (RefSeq) BAHD acyltransferase At5g47980-like (A)	BAHD acyltransferase At5g47980; EC=2.3.1.- (At3g26040)		-1.475
Cs7g10780	K13065 shikimate O-hydroxycinnamoyltransferase [EC:2.3.1.133] | (RefSeq) shikimate O-hydroxycinnamoyltransferase-like (A)	(At1g27380)		-1.156
Cs7g29080	K13065 shikimate O-hydroxycinnamoyltransferase [EC:2.3.1.133] | (RefSeq) shikimate O-hydroxycinnamoyltransferase-like (A)	Shikimate O-hydroxycinnamoyltransferase; EC=2.3.1.133; Hydroxycinnamoyl transferase; Hydroxycinnamoyl-Coenzyme A shikimate/quinate hydroxycinnamoyltransferase (At5g48930)		-1.346
Cs7g29780	K01859 chalcone isomerase [EC:5.5.1.6] | (RefSeq) probable chalconeflavonone isomerase 3 (A)	Probable chalconeflavonone isomerase 3; Chalcone isomerase 3; EC=5.5.1.6; Chalcone isomerase-like 1 (At5g05270)		-1.305
Cs7g31010	K05277 leucoanthocyanidin dioxygenase [EC:1.14.11.19] | (RefSeq) leucoanthocyanidin dioxygenase (A)	Probable 2-oxoglutarate-dependent dioxygenase At5g05600 {ECO:0000305}; EC=1.14.11.- {ECO:0000305} (At5g05600)		-1.142
Cs8g03950	K05278 flavonol synthase [EC:1.14.11.23] | (RefSeq) flavonol synthase/flavanone 3-hydroxylase-like (A)	Protein SRG1; AtSRG1; Protein SENESCENCE-RELATED GENE 1 (At1g49390)		-1.217
Cs8g09800	K00660 chalcone synthase [EC:2.3.1.74] | (RefSeq) chalcone synthase 2-like (A)	Chalcone synthase 2; EC=2.3.1.74; Naringenin-chalcone synthase 2 (At5g13930)		2.197
Cs8g09830	K00660 chalcone synthase [EC:2.3.1.74] | (RefSeq) chalcone synthase 2-like (A)	Chalcone synthase 2; EC=2.3.1.74; Naringenin-chalcone synthase 2 (At5g13930)		1.133
Cs9g08970	K13065 shikimate O-hydroxycinnamoyltransferase [EC:2.3.1.133] | (RefSeq) salutaridinol 7-O-acetyltransferase-like (A)	Salutaridinol 7-O-acetyltransferase {ECO:0000303|PubMed:11404355}; salAT {ECO:0000303|PubMed:11404355}; EC=2.3.1.150		1.363

		{ECO:0000269|PubMed:11404355, ECO:0000269|PubMed:8537369} (At3g26040)		
Cs9g11190	K00660 chalcone synthase [EC:2.3.1.74] | (RefSeq) chalcone synthase 1 (A)	Chalcone synthase 1; EC=2.3.1.74; Naringenin-chalcone synthase 1 (At5g13930)		-1.384
Cs9g14480	K00475 naringenin 3-dioxygenase [EC:1.14.11.9] | (RefSeq) naringenin,2-oxoglutarate 3-dioxygenase (A)	Protein DMR6-LIKE OXYGENASE 2 {ECO:0000303|PubMed:25376907}; EC=1.14.11.- {ECO:0000255|PROSITE-ProRule:PRU00805}; 2-oxoglutarate (2OG)-Fe(II) oxygenase-like protein DLO2 {ECO:0000303|PubMed:25376907}; Salicylate 3-hydroxylase DLO2 {ECO:0000305}; S3H DLO2 {ECO:0000305}; SA 3-hydroxylase DLO2 {ECO:0000305}; Salicylic acid 3-hydroxylase DLO2 {ECO:0000305}; EC=1.14.13.- {ECO:0000250|UniProtKB:Q9ZSA8} (At4g10490)	4.517	
Cs9g14520	K05278 flavonol synthase [EC:1.14.11.23] | (RefSeq) flavonol synthase/flavanone 3-hydroxylase (A)	Protein DMR6-LIKE OXYGENASE 2 {ECO:0000303|PubMed:25376907}; EC=1.14.11.- {ECO:0000255|PROSITE-ProRule:PRU00805}; 2-oxoglutarate (2OG)-Fe(II) oxygenase-like protein DLO2 {ECO:0000303|PubMed:25376907}; Salicylate 3-hydroxylase DLO2 {ECO:0000305}; S3H DLO2 {ECO:0000305}; SA 3-hydroxylase DLO2 {ECO:0000305}; Salicylic acid 3-hydroxylase DLO2 {ECO:0000305}; EC=1.14.13.- {ECO:0000250|UniProtKB:Q9ZSA8} (At4g10490)	6.224	
Cs9g18820	K22845 phlorizin synthase [EC:2.4.1.357] | (RefSeq) UDP-glycosyltransferase 88A1 (A)	Anthocyanidin 5,3-O-glucosyltransferase; EC=2.4.1.-; UDP-glucose: anthocyanidin 5,3-O-glucosyltransferase (At3g16520)		-2.140
Cs9g18830	K22845 phlorizin synthase [EC:2.4.1.357] | (RefSeq) UDP-glycosyltransferase 88A1 (A)	Anthocyanidin 5,3-O-glucosyltransferase; EC=2.4.1.-; UDP-glucose: anthocyanidin 5,3-O-glucosyltransferase (At3g16520)		-3.767
Cs9g19030	K13065 shikimate O-hydroxycinnamoyltransferase [EC:2.3.1.133] | (RefSeq) hypothetical protein (A)	BAHD acyltransferase At5g47980; EC=2.3.1.- (At3g26040)		1.030
novel.2758	K13065 shikimate O-hydroxycinnamoyltransferase [EC:2.3.1.133] | (RefSeq) shikimate O-hydroxycinnamoyltransferase-like (A)	HST_TOBAC Shikimate O-hydroxycinnamoyltransferase; EC=2.3.1.133; Hydroxycinnamoyl transferase; Hydroxycinnamoyl-Coenzyme A shikimate/quinate hydroxycinnamoyltransferase (At5g48930)		-1.226
novel.576	K13080 flavanone 7-O-glucoside 2"-O-beta-L-rhamnosyltransferase [EC:2.4.1.236] | (RefSeq) flavanone 7-O-glucoside	FLRT_CITMA Flavanone 7-O-glucoside 2"-O-beta-L-rhamnosyltransferase; EC=2.4.1.236	1.159	

	2''-O-beta-L-rhamnosyltransferase-like (A)	{ECO:0000269|PubMed:1939145}; 1,2 rhamnosyltransferase (At5g65550)		
novel.738	K13065 shikimate O-hydroxycinnamoyltransferase [EC:2.3.1.133] | (RefSeq) shikimate O-hydroxycinnamoyltransferase (A)	HST_ARATH Shikimate O-hydroxycinnamoyltransferase; EC=2.3.1.133; Hydroxycinnamoyl transferase; Hydroxycinnamoyl-Coenzyme A shikimate/quinate hydroxycinnamoyltransferase (At5g48930)		-1.997
novel.739	K13065 shikimate O-hydroxycinnamoyltransferase [EC:2.3.1.133] | (RefSeq) shikimate O-hydroxycinnamoyltransferase-like (A)	HST_ARATH Shikimate O-hydroxycinnamoyltransferase; EC=2.3.1.133; Hydroxycinnamoyl transferase; Hydroxycinnamoyl-Coenzyme A shikimate/quinate hydroxycinnamoyltransferase (At5g48930)		-1.375
orange1.1t02793	K13065 shikimate O-hydroxycinnamoyltransferase [EC:2.3.1.133] | (RefSeq) shikimate O-hydroxycinnamoyltransferase (A)	Shikimate O-hydroxycinnamoyltransferase; EC=2.3.1.133; Hydroxycinnamoyl transferase; Hydroxycinnamoyl-Coenzyme A shikimate/quinate hydroxycinnamoyltransferase (At5g48930)		1.021
orange1.1t05430	K00660 chalcone synthase [EC:2.3.1.74] | (RefSeq) chalcone synthase 2-like (A)	Chalcone synthase 2; EC=2.3.1.74; Naringenin-chalcone synthase 2 (At5g13930)	4.664	
Isoquinoline alkaloid biosynthesis [ko00950; P = 0.0016 (P3CR vs. P3R) and 0.3905 (P5CR vs. P5R)]				
Cs1g06680	K03183 demethylmenaquinone methyltransferase / 2-methoxy-6-polyprenyl-1,4-benzoquinol methylase [EC:2.1.1.163 2.1.1.201] | (RefSeq) 2-phytyl-1,4-beta-naphthoquinone methyltransferase, chloroplastic-like (A)	2-phytyl-1,4-beta-naphthoquinone methyltransferase, chloroplastic {ECO:0000255|HAMAP-Rule:MF_03192}; EC=2.1.1.329 {ECO:0000255|HAMAP-Rule:MF_03192}; Demethylphylloquinone methyltransferase {ECO:0000255|HAMAP-Rule:MF_03192}; Menaquinone biosynthesis methyltransferase ubiE-like protein {ECO:0000255|HAMAP-Rule:MF_03192}; Flags: Precursor (At5g54400)	-1.020	
Cs1g08010	K00815 tyrosine aminotransferase [EC:2.6.1.5] | (RefSeq) hypothetical protein (A)	Probable aminotransferase TAT2; EC=2.6.1.-; Tyrosine aminotransferase 2 (At5g53970)	1.218	
Cs1g12000	K09833 homogentisate phytyltransferase / homogentisate geranylgeranyltransferase [EC:2.5.1.115 2.5.1.116] | (RefSeq) homogentisate phytyltransferase 1, chloroplastic-like (A)	Coumarin 8-geranyltransferase 1b, chloroplastic; EC=2.5.1.138 {ECO:0000269|PubMed:25077796}; Prenyltransferase 1b {ECO:0000303|PubMed:25077796}; ClPT1ba {ECO:0000303|PubMed:25077796}; Flags: Precursor (At2g18950)		-8.298
Cs2g07170	K03809 NAD(P)H dehydrogenase (quinone) [EC:1.6.5.2] | (RefSeq) probable NAD(P)H dehydrogenase (quinone) FQR1-like 1 (A)	Probable NAD(P)H dehydrogenase (quinone) FQR1-like 1 {ECO:0000305}; EC=1.6.5.2 {ECO:0000250|UniProtKB:Q9LSQ5} (At5g54500)		1.104
Cs2g13990	K09833 homogentisate phytyltransferase / homogentisate	Coumarin 8-geranyltransferase 1b, chloroplastic; EC=2.5.1.138	1.087	1.778

	geranylgeranyltransferase [EC:2.5.1.115 2.5.1.116] | (RefSeq) probable homogentisate phytyltransferase 1, chloroplastic (A)	{ECO:0000269|PubMed:25077796}; Prenyltransferase 1b {ECO:0000303|PubMed:25077796}; ClPT1ba {ECO:0000303|PubMed:25077796}; Flags: Precursor (At2g18950)		
Cs2g14050	K09833 homogentisate phytyltransferase / homogentisate geranylgeranyltransferase [EC:2.5.1.115 2.5.1.116] | (RefSeq) homogentisate geranylgeranyltransferase, chloroplastic-like (A)	Coumarin 8-geranyltransferase 1b, chloroplastic; EC=2.5.1.138 {ECO:0000269|PubMed:25077796}; Prenyltransferase 1b {ECO:0000303|PubMed:25077796}; ClPT1ba {ECO:0000303|PubMed:25077796}; Flags: Precursor (At2g18950)	1.244	1.894
Cs2g23840	K00815 tyrosine aminotransferase [EC:2.6.1.5] | (RefSeq) hypothetical protein (A)	Probable aminotransferase TAT2; EC=2.6.1.-; Tyrosine aminotransferase 2 (At5g53970)		1.425
Cs2g23850	K00815 tyrosine aminotransferase [EC:2.6.1.5] | (RefSeq) hypothetical protein (A)	Probable aminotransferase TAT2; EC=2.6.1.-; Tyrosine aminotransferase 2 (At5g53970)		1.042
Cs3g04700	K09833 homogentisate phytyltransferase / homogentisate geranylgeranyltransferase [EC:2.5.1.115 2.5.1.116] | (RefSeq) hypothetical protein (A)	Coumarin 8-geranyltransferase 1b, chloroplastic; EC=2.5.1.138 {ECO:0000269|PubMed:25077796}; Prenyltransferase 1b {ECO:0000303|PubMed:25077796}; ClPT1ba {ECO:0000303|PubMed:25077796}; Flags: Precursor (At2g18950)	2.365	-2.120
Cs3g08510	K01904 4-coumarate CoA ligase [EC:6.2.1.12] | (RefSeq) hypothetical protein (A)	4-coumarateCoA ligase-like 6; EC=6.2.1.-; 4-coumarateCoA ligase isoform 7; At4CL7 (At4g19010)	-1.625	
Cs4g03330	K01904 4-coumarate CoA ligase [EC:6.2.1.12] | (RefSeq) hypothetical protein (A)	4-coumarateCoA ligase 1; 4CL 1; EC=6.2.1.12; 4-coumaroyl-CoA synthase 1 (At3g21240)	1.132	3.074
Cs4g10160	K09833 homogentisate phytyltransferase / homogentisate geranylgeranyltransferase [EC:2.5.1.115 2.5.1.116] | (RefSeq) homogentisate phytyltransferase 1, chloroplastic-like (A)	Coumarin 8-geranyltransferase 1b, chloroplastic; EC=2.5.1.138 {ECO:0000269|PubMed:25077796}; Prenyltransferase 1b {ECO:0000303|PubMed:25077796}; ClPT1ba {ECO:0000303|PubMed:25077796}; Flags: Precursor (At3g11950)	2.444	
Cs7g17370	K09833 homogentisate phytyltransferase / homogentisate geranylgeranyltransferase [EC:2.5.1.115 2.5.1.116] | (RefSeq) homogentisate phytyltransferase 1, chloroplastic-like (A)	Coumarin 8-geranyltransferase 1, chloroplastic; EC=2.5.1.138 {ECO:0000269|PubMed:25077796}; Prenyltransferase 1 {ECO:0000303|PubMed:25077796}; ClPT1 {ECO:0000303|PubMed:25077796}; Umbelliferone 8-C-geranyltransferase {ECO:0000303|PubMed:25077796}; U8GT {ECO:0000303|PubMed:25077796}; Flags: Precursor		2.169
Cs7g17390	K09833 homogentisate phytyltransferase / homogentisate geranylgeranyltransferase [EC:2.5.1.115 2.5.1.116] | (RefSeq) homogentisate phytyltransferase 1, chloroplastic-like (A)	Coumarin 8-geranyltransferase 1b, chloroplastic; EC=2.5.1.138 {ECO:0000269|PubMed:25077796}; Prenyltransferase 1b {ECO:0000303|PubMed:25077796}; ClPT1ba {ECO:0000303|PubMed:25077796}; Flags: Precursor (At2g18950)	2.268	1.567
Cs9g17840	K09833 homogentisate phytyltransferase / homogentisate	Coumarin 8-geranyltransferase 1b, chloroplastic; EC=2.5.1.138		1.987

	geranylgeranyltransferase [EC:2.5.1.115 2.5.1.116] | (RefSeq) homogentisate phytyltransferase 1, chloroplastic-like (A)	{ECO:0000269|PubMed:25077796}; Prenyltransferase 1b {ECO:0000303|PubMed:25077796}; ClPT1ba {ECO:0000303|PubMed:25077796}; Flags: Precursor (At3g11950)		
novel.25	K00815 tyrosine aminotransferase [EC:2.6.1.5] | (RefSeq) LOW QUALITY PROTEIN: tyrosine aminotransferase-like (A)	(At2g06190)	-1.347	
novel.2856	K09833 homogentisate phytyltransferase / homogentisate geranylgeranyltransferase [EC:2.5.1.115 2.5.1.116] | (RefSeq) hypothetical protein (A)		1.222	1.572
novel.320	K09833 homogentisate phytyltransferase / homogentisate geranylgeranyltransferase [EC:2.5.1.115 2.5.1.116] | (RefSeq) hypothetical protein (A)	CGT1A_CITLI Coumarin 8-geranyltransferase 1, chloroplastic; EC=2.5.1.138 {ECO:0000269|PubMed:25077796}; Prenyltransferase 1 {ECO:0000303|PubMed:25077796}; ClPT1 {ECO:0000303|PubMed:25077796}; Umbelliferone 8-C-geranyltransferase {ECO:0000303|PubMed:25077796}; U8GT {ECO:0000303|PubMed:25077796}; Flags: Precursor	1.066	
orange1.1t04551	K09833 homogentisate phytyltransferase / homogentisate geranylgeranyltransferase [EC:2.5.1.115 2.5.1.116] | (RefSeq) homogentisate phytyltransferase 1, chloroplastic-like (A)	Coumarin 8-geranyltransferase 1b, chloroplastic; EC=2.5.1.138 {ECO:0000269|PubMed:25077796}; Prenyltransferase 1b {ECO:0000303|PubMed:25077796}; ClPT1ba {ECO:0000303|PubMed:25077796}; Flags: Precursor (At2g18950)		2.387
orange1.1t05352	K09833 homogentisate phytyltransferase / homogentisate geranylgeranyltransferase [EC:2.5.1.115 2.5.1.116] | (RefSeq) hypothetical protein (A)	Umbelliferone 6-dimethylallyltransferase, chloroplastic {ECO:0000303|PubMed:24354545}; EC=2.5.1.139 {ECO:0000269|PubMed:24354545}; Flags: Precursor (At2g18950)		-1.673
Tropane, piperidine and pyridine alkaloid biosynthesis [ko00960; P = 0.0044 (P3CR vs. P3R) and 0.4808 (P5CR vs. P5R)]				
Cs1g08010	K00815 tyrosine aminotransferase [EC:2.6.1.5] | (RefSeq) hypothetical protein (A)	Probable aminotransferase TAT2; EC=2.6.1.-; Tyrosine aminotransferase 2 (At5g53970)	1.218	
Cs2g23840	K00815 tyrosine aminotransferase [EC:2.6.1.5] | (RefSeq) hypothetical protein (A)	Probable aminotransferase TAT2; EC=2.6.1.-; Tyrosine aminotransferase 2 (At5g53970)		1.425
Cs2g23850	K00815 tyrosine aminotransferase [EC:2.6.1.5] | (RefSeq) hypothetical protein (A)	Probable aminotransferase TAT2; EC=2.6.1.-; Tyrosine aminotransferase 2 (At5g53970)		1.042
Cs5g05990	K00276 primary-amine oxidase [EC:1.4.3.21] | (RefSeq) hypothetical protein (A)	U-box domain-containing protein 12; EC=2.3.2.27; Plant U-box protein 12; OsPUB12; RING-type E3 ubiquitin transferase PUB12 {ECO:0000305} (At3g26600)	1.336	1.395
Cs5g09470	K00276 primary-amine oxidase [EC:1.4.3.21] | (RefSeq) primary amine oxidase-like (A)	Primary amine oxidase; EC=1.4.3.21 {ECO:0000250|UniProtKB:P46883}; Amine oxidase		1.008

		[copper-containing]; Flags: Precursor (At4g12290)		
Cs5g30120	K08081 tropinone reductase I [EC:1.1.1.206] | (RefSeq) tropinone reductase homolog At5g06060-like (A)	Transcriptional regulator TAC1; Protein TELOMERASE ACTIVATOR1 (At3g09290)	1.113	
Cs6g06630	K08081 tropinone reductase I [EC:1.1.1.206] | (RefSeq) tropinone reductase homolog At5g06060-like (A)	Transcriptional regulator TAC1; Protein TELOMERASE ACTIVATOR1 (At3g09290)	1.578	
Cs6g09690	K14455 aspartate aminotransferase, mitochondrial [EC:2.6.1.1] | (RefSeq) hypothetical protein (A)	Aspartate aminotransferase, mitochondrial; EC=2.6.1.1; Transaminase A; Flags: Precursor (At2g30970)	-1.627	
Cs8g19060	K08081 tropinone reductase I [EC:1.1.1.206] | (RefSeq) hypothetical protein (A)	Tropinone reductase homolog At2g29170 {ECO:0000305}; EC=1.1.1.- {ECO:0000305} (At2g29170)	1.296	1.801
Cs8g19070	K08081 tropinone reductase I [EC:1.1.1.206] | (RefSeq) tropinone reductase homolog At2g29170-like (A)	Tropinone reductase homolog At2g29170 {ECO:0000305}; EC=1.1.1.- {ECO:0000305} (At2g29170)		-1.055
Cs8g19105	K08081 tropinone reductase I [EC:1.1.1.206] | (RefSeq) tropinone reductase homolog At2g29170-like (A)	Tropinone reductase homolog At2g29170 {ECO:0000305}; EC=1.1.1.- {ECO:0000305} (At2g29170)	1.310	1.922
Cs8g19110	K08081 tropinone reductase I [EC:1.1.1.206] | (RefSeq) hypothetical protein (A)	Senescence-associated protein 13 {ECO:0000303|PubMed:9617813}; Tropinone reductase homolog SAG13 {ECO:0000250|UniProtKB:P50162}; EC=1.1.1.- {ECO:0000305} (At2g29350)		-2.568
Cs9g06700	K00276 primary-amine oxidase [EC:1.4.3.21] | (RefSeq) hypothetical protein (A)	Primary amine oxidase 2; EC=1.4.3.21 {ECO:0000250|UniProtKB:P12807}; Amine oxidase [copper-containing] 2; Flags: Precursor (At1g31670)	7.325	
Cs9g06710	K00276 primary-amine oxidase [EC:1.4.3.21] | (RefSeq) hypothetical protein (A)	Primary amine oxidase; EC=1.4.3.21 {ECO:0000250|UniProtKB:P46883}; Amine oxidase [copper-containing]; Flags: Precursor; Fragment (At1g31690)	5.304	
Cs9g06770	K00276 primary-amine oxidase [EC:1.4.3.21] | (RefSeq) hypothetical protein (A)	Primary amine oxidase; EC=1.4.3.21 {ECO:0000250|UniProtKB:P46883}; Amine oxidase [copper-containing]; Flags: Precursor; Fragment (At1g31700)	1.992	
novel.25	K00815 tyrosine aminotransferase [EC:2.6.1.5] | (RefSeq) LOW QUALITY PROTEIN: tyrosine aminotransferase-like (A)	(At2g06190)	-1.347	
orange1.1t01039	K08081 tropinone reductase I [EC:1.1.1.206] | (RefSeq) tropinone reductase homolog At5g06060-like (A)	Zinc finger protein 10 {ECO:0000305}; AtZFP10 {ECO:0000303|PubMed:12154136} (At4g17810)		-7.776
orange1.1t03034	K08081 tropinone reductase I [EC:1.1.1.206] | (RefSeq) tropinone reductase homolog (A)	Tropinone reductase homolog; EC=1.1.1.-; P29X (At5g06060)	1.610	
orange1.1t030	K08081 tropinone reductase I [EC:1.1.1.206] | (RefSeq) tropinone	Tropinone reductase homolog; EC=1.1.1.-; P29X (At2g29150)	2.066	

42	reductase homolog isoform X1 (A)			
orange1.1t05631	K08081 tropinone reductase I [EC:1.1.1.206] | (RefSeq) tropinone reductase homolog At2g29170-like (A)	Tropinone reductase homolog At2g29360 {ECO:0000305}; EC=1.1.1.- {ECO:0000305} (At2g29360)		3.036
Acridone alkaloid biosynthesis [ko01058; P = 0.0389 (P3CR vs. P3R)]				
Cs1g03730	K12644 acridone synthase [EC:2.3.1.159] | (RefSeq) hypothetical protein (A)	Acridone synthase 2; EC=2.3.1.159; Acridone synthase II (At5g13930)	5.866	
orange1.1t00577	K12643 anthranilate N-methyltransferase [EC:2.1.1.111] | (RefSeq) anthranilate N-methyltransferase-like (A)	Anthranilate N-methyltransferase; RgANMT; EC=2.1.1.111 (At5g54160)	1.373	
Lignin biosynthetic process [GO:0009809; P = 0.0001 (P3CR vs. P3R) and 0.0003 (P5CR vs. P5R)]				
Cs1g03780	K13066 caffeic acid 3-O-methyltransferase [EC:2.1.1.68] | (RefSeq) caffeic acid 3-O-methyltransferase-like isoform X1 (A)	Caffeic acid 3-O-methyltransferase; CAOMT; COMT; EC=2.1.1.68; S-adenosysl-L-methionine:caffeic acid 3-O-methyltransferase (At5g54160)		1.270
Cs1g04910	K00083 cinnamyl-alcohol dehydrogenase [EC:1.1.1.195] | (RefSeq) hypothetical protein (A)	Probable cinnamyl alcohol dehydrogenase 9; AtCAD9; EC=1.1.1.195 (At4g39330)	-2.173	
Cs2g09210	K00430 peroxidase [EC:1.11.1.7] | (RefSeq) peroxidase P7-like (A)	Peroxidase 52; Atperox P52; EC=1.11.1.7; ATP49; Flags: Precursor (At5g05340)		1.669
Cs2g30460	K09753 cinnamoyl-CoA reductase [EC:1.2.1.44] | (RefSeq) cinnamoyl-CoA reductase 1-like isoform X1 (A)	Cinnamoyl-CoA reductase 1; AtCCR1; EC=1.2.1.44; Protein IRREGULAR XYLEM 4 (At5g19440)	2.798	
Cs2g30470	K09753 cinnamoyl-CoA reductase [EC:1.2.1.44] | (RefSeq) cinnamoyl-CoA reductase 2-like (A)	Cinnamoyl-CoA reductase 1; AtCCR1; EC=1.2.1.44; Protein IRREGULAR XYLEM 4 (At1g51410)		-1.535
Cs3g20650	K13066 caffeic acid 3-O-methyltransferase [EC:2.1.1.68] | (RefSeq) caffeic acid 3-O-methyltransferase (A)	Caffeic acid 3-O-methyltransferase; CAOMT; COMT; EC=2.1.1.68; S-adenosysl-L-methionine:caffeic acid 3-O-methyltransferase (At5g54160)	3.127	
Cs4g18140	K03006 DNA-directed RNA polymerase II subunit RPB1 [EC:2.7.7.6] | (RefSeq) early nodulin-like protein 2 (A)	Blue copper protein; Blue copper-binding protein; AtBCB; Phytocyanin 1; Stellacyanin; Flags: Precursor (At5g20230)		-1.225
Cs5g13580	K13066 caffeic acid 3-O-methyltransferase [EC:2.1.1.68] | (RefSeq) caffeic acid 3-O-methyltransferase-like (A)	Caffeic acid 3-O-methyltransferase; CAOMT; COMT; EC=2.1.1.68; S-adenosysl-L-methionine:caffeic acid 3-O-methyltransferase (At5g54160)	3.547	
Cs5g16290	K13066 caffeic acid 3-O-methyltransferase [EC:2.1.1.68] | (RefSeq) caffeic acid 3-O-methyltransferase (A)	Caffeic acid 3-O-methyltransferase; CAOMT; COMT; EC=2.1.1.68; S-adenosysl-L-methionine:caffeic acid 3-O-methyltransferase (At5g54160)		1.277

Cs5g18050	K13066 caffeic acid 3-O-methyltransferase [EC:2.1.1.68] | (RefSeq) caffeic acid 3-O-methyltransferase-like isoform X1 (A)	Caffeic acid 3-O-methyltransferase; CAOMT; COMT; EC=2.1.1.68; S-adenosysl-L-methionine:caffeic acid 3-O-methyltransferase (At5g54160)	2.004	
Cs5g19020	K13066 caffeic acid 3-O-methyltransferase [EC:2.1.1.68] | (RefSeq) hypothetical protein (A)	Caffeic acid 3-O-methyltransferase; CAOMT; COMT; EC=2.1.1.68; S-adenosysl-L-methionine:caffeic acid 3-O-methyltransferase (At5g54160)	1.778	1.136
Cs5g25000	K13066 caffeic acid 3-O-methyltransferase [EC:2.1.1.68] | (RefSeq) caffeic acid 3-O-methyltransferase-like (A)	Caffeic acid 3-O-methyltransferase; CAOMT; COMT; EC=2.1.1.68; S-adenosysl-L-methionine:caffeic acid 3-O-methyltransferase (At5g54160)		1.383
Cs6g06880	K05909 laccase [EC:1.10.3.2] | (RefSeq) hypothetical protein (A)	Laccase-17; EC=1.10.3.2; Benzenediol:oxygen oxidoreductase 17; Diphenol oxidase 17; Urishiol oxidase 17; Flags: Precursor (At5g60020)		4.438
Cs6g06920	K05909 laccase [EC:1.10.3.2] | (RefSeq) laccase-17-like (A)	Laccase-17; EC=1.10.3.2; Benzenediol:oxygen oxidoreductase 17; Diphenol oxidase 17; Urishiol oxidase 17; Flags: Precursor (At5g60020)	1.652	3.497
Cs6g07800	K05909 laccase [EC:1.10.3.2] | (RefSeq) hypothetical protein (A)	Laccase-4; EC=1.10.3.2; Benzenediol:oxygen oxidoreductase 4; Diphenol oxidase 4; Protein IRREGULAR XYLEM 12; Urishiol oxidase 4; Flags: Precursor (At2g38080)	1.504	3.815
Cs6g16650	K09754 coumaroylquinate(coumaroylshikimate) 3'-monooxygenase [EC:1.14.13.36] | (RefSeq) cytochrome P450 98A2 (A)	Cytochrome P450 98A2; EC=1.14.-.- (At2g40890)	1.097	1.588
Cs7g23490	K05909 laccase [EC:1.10.3.2] | (RefSeq) hypothetical protein (A)	Laccase-17; EC=1.10.3.2; Benzenediol:oxygen oxidoreductase 17; Diphenol oxidase 17; Urishiol oxidase 17; Flags: Precursor (At5g60020)		3.109
Cs7g25010	K13066 caffeic acid 3-O-methyltransferase [EC:2.1.1.68] | (RefSeq) COMT1-2; caffeic acid O-methyltransferase (A)	Caffeic acid 3-O-methyltransferase; CAOMT; COMT; EC=2.1.1.68; S-adenosysl-L-methionine:caffeic acid 3-O-methyltransferase (At3g53140)		-1.955
Cs7g25540	K13066 caffeic acid 3-O-methyltransferase [EC:2.1.1.68] | (RefSeq) COMT1-2; caffeic acid O-methyltransferase (A)	Caffeic acid 3-O-methyltransferase; CAOMT; COMT; EC=2.1.1.68; S-adenosysl-L-methionine:caffeic acid 3-O-methyltransferase (At3g53140)	-1.256	-1.457
Cs7g25580	K13066 caffeic acid 3-O-methyltransferase [EC:2.1.1.68] | (RefSeq) caffeic acid 3-O-methyltransferase-like (A)	Caffeic acid 3-O-methyltransferase; CAOMT; COMT; EC=2.1.1.68; S-adenosysl-L-methionine:caffeic acid 3-O-methyltransferase (At5g54160)	4.994	
Cs8g17630	K05909 laccase [EC:1.10.3.2] | (RefSeq) hypothetical protein (A)	Laccase-17; EC=1.10.3.2; Benzenediol:oxygen oxidoreductase 17; Diphenol oxidase 17; Urishiol oxidase 17; Flags: Precursor	2.374	5.969

		(At5g60020)		
Cs8g18800	K05909 laccase [EC:1.10.3.2] | (RefSeq) laccase-17 (A)	Laccase-17; EC=1.10.3.2; Benzenediol:oxygen oxidoreductase 17; Diphenol oxidase 17; Urishiol oxidase 17; Flags: Precursor (At5g60020)	3.255	3.790
Cs8g19850	K05909 laccase [EC:1.10.3.2] | (RefSeq) hypothetical protein (A)	Laccase-4; EC=1.10.3.2; Benzenediol:oxygen oxidoreductase 4; Diphenol oxidase 4; Protein IRREGULAR XYLEM 12; Urishiol oxidase 4; Flags: Precursor (At2g38080)	1.651	3.446
novel.2285	K13066 caffeic acid 3-O-methyltransferase [EC:2.1.1.68] | (RefSeq) COMT1-2; caffeic acid O-methyltransferase (A)	COMT2_OCIBA Caffeic acid 3-O-methyltransferase 2; CAOMT-2; COMT-2; EC=2.1.1.68; S-adenosysl-L-methionine:caffeic acid 3-O-methyltransferase 2 (At3g53140)		-1.310
novel.2508	K13066 caffeic acid 3-O-methyltransferase [EC:2.1.1.68] | (RefSeq) anthranilate N-methyltransferase-like (A)	COMT1_MEDSA Caffeic acid 3-O-methyltransferase; CAOMT; COMT; EC=2.1.1.68; S-adenosysl-L-methionine:caffeic acid 3-O-methyltransferase (At5g54160)		2.654
novel.738	K13065 shikimate O-hydroxycinnamoyltransferase [EC:2.3.1.133] | (RefSeq) shikimate O-hydroxycinnamoyltransferase (A)	HST_ARATH Shikimate O-hydroxycinnamoyltransferase; EC=2.3.1.133; Hydroxycinnamoyl transferase; Hydroxycinnamoyl-Coenzyme A shikimate/quinate hydroxycinnamoyltransferase (At5g48930)		-1.997
novel.739	K13065 shikimate O-hydroxycinnamoyltransferase [EC:2.3.1.133] | (RefSeq) shikimate O-hydroxycinnamoyltransferase-like (A)	HST_ARATH Shikimate O-hydroxycinnamoyltransferase; EC=2.3.1.133; Hydroxycinnamoyl transferase; Hydroxycinnamoyl-Coenzyme A shikimate/quinate hydroxycinnamoyltransferase (At5g48930)		-1.375
novel.975	K13066 caffeic acid 3-O-methyltransferase [EC:2.1.1.68] | (RefSeq) hypothetical protein (A)	COMT1_MEDSA Caffeic acid 3-O-methyltransferase; CAOMT; COMT; EC=2.1.1.68; S-adenosysl-L-methionine:caffeic acid 3-O-methyltransferase (At5g54160)	2.726	
orange1.1t01084	K13066 caffeic acid 3-O-methyltransferase [EC:2.1.1.68] | (Kazusa) Lj2g3v3339490.1; - (A)	Caffeic acid 3-O-methyltransferase; CAOMT; COMT; EC=2.1.1.68; S-adenosysl-L-methionine:caffeic acid 3-O-methyltransferase (At3g53140)	-1.168	-1.088
orange1.1t01768	K20240 spermidine dicoumaroyl transferase [EC:2.3.1.249] | (RefSeq) acyl transferase 5 (A)	Acyl transferase 4 {ECO:0000305}; OsAT4 {ECO:0000303|PubMed:23391577}; EC=2.3.1.- {ECO:0000305}; p-coumaroyl-CoA monolignol transferase {ECO:0000305}; OsPMT {ECO:0000303|PubMed:22267741} (At3g62160)	2.871	5.366
orange1.1t02085	K13066 caffeic acid 3-O-methyltransferase [EC:2.1.1.68] | (RefSeq) caffeic acid 3-O-methyltransferase-like isoform X1 (A)	Caffeic acid 3-O-methyltransferase 1; CAOMT-1; COMT-1; EC=2.1.1.68; S-adenosysl-L-methionine:caffeic acid 3-O-methyltransferase 1 (At5g54160)		-2.085

orange1.1t02091	K13066 caffeic acid 3-O-methyltransferase [EC:2.1.1.68] | (RefSeq) hypothetical protein (A)	Caffeic acid 3-O-methyltransferase 1; CAOMT-1; COMT-1; EC=2.1.1.68; S-adenosysl-L-methionine:caffeic acid 3-O-methyltransferase 1 (At5g54160)		-2.365
orange1.1t03987	K13066 caffeic acid 3-O-methyltransferase [EC:2.1.1.68] | (RefSeq) caffeic acid 3-O-methyltransferase-like (A)	Caffeic acid 3-O-methyltransferase; CAOMT; COMT; EC=2.1.1.68; S-adenosysl-L-methionine:caffeic acid 3-O-methyltransferase (At5g54160)	6.849	
orange1.1t04194	K13066 caffeic acid 3-O-methyltransferase [EC:2.1.1.68] | (RefSeq) caffeic acid 3-O-methyltransferase-like (A)	Caffeic acid 3-O-methyltransferase; CAOMT; COMT; EC=2.1.1.68; S-adenosysl-L-methionine:caffeic acid 3-O-methyltransferase (At5g54160)	3.478	
orange1.1t05016	K13066 caffeic acid 3-O-methyltransferase [EC:2.1.1.68] | (RefSeq) caffeic acid 3-O-methyltransferase-like isoform X2 (A)	Caffeic acid 3-O-methyltransferase; CAOMT; COMT; EC=2.1.1.68; S-adenosysl-L-methionine:caffeic acid 3-O-methyltransferase (At5g54160)	1.113	1.584
orange1.1t05018	K13066 caffeic acid 3-O-methyltransferase [EC:2.1.1.68] | (RefSeq) caffeic acid 3-O-methyltransferase-like isoform X2 (A)	Caffeic acid 3-O-methyltransferase; CAOMT; COMT; EC=2.1.1.68; S-adenosysl-L-methionine:caffeic acid 3-O-methyltransferase (At5g54160)	1.136	1.021
orange1.1t05216	K13066 caffeic acid 3-O-methyltransferase [EC:2.1.1.68] | (RefSeq) caffeic acid 3-O-methyltransferase-like (A)	Flavone 3'-O-methyltransferase 1; AtOMT1; EC=2.1.1.42; Acetylserotonin O-methyltransferase OMT1 {ECO:0000305}; EC=2.1.1.4 {ECO:0000269|PubMed:25039887}; Caffeate O-methyltransferase 1; EC=2.1.1.68; Quercetin 3'-O-methyltransferase 1 (At3g53140)		-1.581
orange1.1t05232	K13066 caffeic acid 3-O-methyltransferase [EC:2.1.1.68] | (RefSeq) anthranilate N-methyltransferase-like (A)	Caffeic acid 3-O-methyltransferase; CAOMT; COMT; EC=2.1.1.68; S-adenosysl-L-methionine:caffeic acid 3-O-methyltransferase (At5g54160)	5.886	
orange1.1t05402	K00430 peroxidase [EC:1.11.1.7] | (RefSeq) peroxidase P7-like (A)	Peroxidase 52; Atperox P52; EC=1.11.1.7; ATP49; Flags: Precursor (At5g05340)		1.615
Lignin catabolic process [GO:0046274; P < 0.0001 (P3CR vs. P3R) and 0.0001 (P5CR vs. P5R)]				
Cs1g24250	K05909 laccase [EC:1.10.3.2] | (RefSeq) laccase-11-like (A)	Laccase-11; EC=1.10.3.2; Benzenediol:oxygen oxidoreductase 11; Diphenol oxidase 11; Urishiol oxidase 11; Flags: Precursor (At5g03260)	3.444	6.447
Cs6g06880	K05909 laccase [EC:1.10.3.2] | (RefSeq) hypothetical protein (A)	Laccase-17; EC=1.10.3.2; Benzenediol:oxygen oxidoreductase 17; Diphenol oxidase 17; Urishiol oxidase 17; Flags: Precursor (At5g60020)		4.438
Cs6g06920	K05909 laccase [EC:1.10.3.2] | (RefSeq) laccase-17-like (A)	Laccase-17; EC=1.10.3.2; Benzenediol:oxygen oxidoreductase 17;	1.652	3.497

		Diphenol oxidase 17; Urishiol oxidase 17; Flags: Precursor (At5g60020)		
Cs6g07400	K05909 laccase [EC:1.10.3.2] | (RefSeq) laccase-7-like (A)	Laccase-7; EC=1.10.3.2; Benzenediol:oxygen oxidoreductase 7; Diphenol oxidase 7; Urishiol oxidase 7; Flags: Precursor (At3g09220)	1.881	
Cs6g07410	K05909 laccase [EC:1.10.3.2] | (RefSeq) hypothetical protein (A)	Laccase-7; EC=1.10.3.2; Benzenediol:oxygen oxidoreductase 7; Diphenol oxidase 7; Urishiol oxidase 7; Flags: Precursor (At3g09220)	1.498	2.464
Cs6g07450	K05909 laccase [EC:1.10.3.2] | (RefSeq) laccase-7-like (A)	Laccase-7; EC=1.10.3.2; Benzenediol:oxygen oxidoreductase 7; Diphenol oxidase 7; Urishiol oxidase 7; Flags: Precursor (At3g09220)		2.442
Cs6g07800	K05909 laccase [EC:1.10.3.2] | (RefSeq) hypothetical protein (A)	Laccase-4; EC=1.10.3.2; Benzenediol:oxygen oxidoreductase 4; Diphenol oxidase 4; Protein IRREGULAR XYLEM 12; Urishiol oxidase 4; Flags: Precursor (At2g38080)	1.504	3.815
Cs6g11860	K05909 laccase [EC:1.10.3.2] | (RefSeq) hypothetical protein (A)	Laccase-11; EC=1.10.3.2; Benzenediol:oxygen oxidoreductase 11; Diphenol oxidase 11; Urishiol oxidase 11; Flags: Precursor (At5g03260)	2.724	5.299
Cs7g23490	K05909 laccase [EC:1.10.3.2] | (RefSeq) hypothetical protein (A)	Laccase-17; EC=1.10.3.2; Benzenediol:oxygen oxidoreductase 17; Diphenol oxidase 17; Urishiol oxidase 17; Flags: Precursor (At5g60020)		3.109
Cs7g30410	K05909 laccase [EC:1.10.3.2] | (RefSeq) hypothetical protein (A)	Laccase-5; EC=1.10.3.2; Benzenediol:oxygen oxidoreductase 5; Diphenol oxidase 5; Urishiol oxidase 5; Flags: Precursor (At2g40370)	1.431	4.293
Cs7g31620	K05909 laccase [EC:1.10.3.2] | (RefSeq) hypothetical protein (A)	Laccase-22; EC=1.10.3.2; Benzenediol:oxygen oxidoreductase 22; Diphenol oxidase 22; Urishiol oxidase 22; Flags: Precursor (At2g38080)	1.349	2.990
Cs8g11710	K05909 laccase [EC:1.10.3.2] | (RefSeq) laccase-13-like (A)	Laccase-3; EC=1.10.3.2; Benzenediol:oxygen oxidoreductase 3; Diphenol oxidase 3; Urishiol oxidase 3; Flags: Precursor (At2g30210)	2.670	3.781
Cs8g17630	K05909 laccase [EC:1.10.3.2] | (RefSeq) hypothetical protein (A)	Laccase-17; EC=1.10.3.2; Benzenediol:oxygen oxidoreductase 17; Diphenol oxidase 17; Urishiol oxidase 17; Flags: Precursor (At5g60020)	2.374	5.969
Cs8g18800	K05909 laccase [EC:1.10.3.2] | (RefSeq) laccase-17 (A)	Laccase-17; EC=1.10.3.2; Benzenediol:oxygen oxidoreductase 17; Diphenol oxidase 17; Urishiol oxidase 17; Flags: Precursor	3.255	3.790

		(At5g60020)		
Cs8g19850	K05909 laccase [EC:1.10.3.2] | (RefSeq) hypothetical protein (A)	Laccase-4; EC=1.10.3.2; Benzenediol:oxygen oxidoreductase 4; Diphenol oxidase 4; Protein IRREGULAR XYLEM 12; Urishiol oxidase 4; Flags: Precursor (At2g38080)	1.651	3.446
orange1.1t00518	K05909 laccase [EC:1.10.3.2] | (RefSeq) hypothetical protein (A)	Laccase-6; EC=1.10.3.2; Benzenediol:oxygen oxidoreductase 6; Diphenol oxidase 6; Urishiol oxidase 6; Flags: Precursor (At2g46570)		1.676
Terpenoid biosynthetic process [GO:0016114; P = 0.1334 (P3CR vs. P3R) and 0.1822 (P5CR vs. P5R)]				
Cs1g05220	K04628 ceramide galactosyltransferase [EC:2.4.1.47] | (RefSeq) hypothetical protein (A)	UDP-glucose iridoid glucosyltransferase; EC=2.4.1.-; UDP-glucose glucosyltransferase 7; CrUGT7; UDP-glycosyltransferase 76A2 (At3g11340)	3.930	
Cs2g07240	K14175 (3S,6E)-nerolidol synthase [EC:4.2.3.48] | (RefSeq) (3S,6E)-nerolidol synthase 1 (A)	(3S,6E)-nerolidol synthase 1, chloroplastic; FvNES1; EC=4.2.3.48; Flags: Precursor (At1g61680)		-2.482
Cs2g07250	K14175 (3S,6E)-nerolidol synthase [EC:4.2.3.48] | (RefSeq) (3S,6E)-nerolidol synthase 1-like (A)	(3S,6E)-nerolidol synthase 2, chloroplastic/mitochondrial; FaNES2; EC=4.2.3.48; Flags: Precursor (At1g61680)	-1.915	-2.704
Cs2g07880	K07437 cytochrome P450 family 26 subfamily A | (RefSeq) taxadiene 5-alpha hydroxylase-like (A)	Taxadiene 5-alpha hydroxylase; EC=1.14.99.37 (At5g36110)	1.848	
Cs2g12700	K09422 transcription factor MYB, plant | (RefSeq) transcription factor MYB24 (A)	Transcription factor MYB62 {ECO:0000303|PubMed:9839469}; Myb-related protein 62 {ECO:0000303|PubMed:9839469}; AtMYB62 {ECO:0000303|PubMed:9839469} (At1g68320)		-3.323
Cs2g16290	K21373 7-deoxyloganetic acid glucosyltransferase [EC:2.4.1.323] | (RefSeq) hypothetical protein (A)	7-deoxyloganetic acid glucosyltransferase; EC=2.4.1.323; UDP-glucose glucosyltransferase 8; CrUGT8; UDP-glycosyltransferase 709C2 (At1g22400)		1.501
Cs3g03640	K13789 geranylgeranyl diphosphate synthase, type II [EC:2.5.1.1 2.5.1.10 2.5.1.29] | (RefSeq) geranylgeranyl pyrophosphate synthase, chloroplastic-like (A)	Geranylgeranyl pyrophosphate synthase, chloroplastic; GGPP synthase; GGPS; EC=2.5.1.-; (2E,6E)-farnesyl diphosphate synthase; Dimethylallyltranstransferase; EC=2.5.1.1; Farnesyl diphosphate synthase; Farnesyltranstransferase; EC=2.5.1.29; Geranyltranstransferase; EC=2.5.1.10; Flags: Precursor (At4g36810)	3.498	
Cs3g11060	K00514 zeta-carotene desaturase [EC:1.3.5.6] | (RefSeq) zeta-carotene desaturase, chloroplastic/chromoplastic-like (A)	Zeta-carotene desaturase, chloroplastic/chromoplastic; EC=1.3.5.6; 9,9'-di-cis-zeta-carotene desaturase; Carotene 7,8-desaturase; Flags: Precursor (At3g04870)		1.040
Cs3g11170	K00514 zeta-carotene desaturase [EC:1.3.5.6] | (RefSeq) CitZDS; ZDS (A)	Zeta-carotene desaturase, chloroplastic/chromoplastic; EC=1.3.5.6; 9,9'-di-cis-zeta-carotene desaturase; Carotene 7,8-desaturase; Flags:	1.422	

		Precursor (At3g04870)		
Cs3g17090	K14510 serine/threonine-protein kinase CTR1 [EC:2.7.11.1] | (RefSeq) serine/threonine-protein kinase CTR1 (A)	Serine/threonine-protein kinase CTR1 {ECO:0000303|PubMed:8431946}; EC=2.7.11.1 {ECO:0000305}; Protein CONSTITUTIVE TRIPLE RESPONSE1 {ECO:0000303|PubMed:8431946} (At4g24480)		1.077
Cs4g04630	K01853 cycloartenol synthase [EC:5.4.99.8] | (RefSeq) hypothetical protein (A)	Cycloartenol synthase; RcCAS; EC=5.4.99.8 (At2g07050)	1.784	-2.809
Cs4g04730	K01853 cycloartenol synthase [EC:5.4.99.8] | (RefSeq) cycloartenol synthase-like (A)	Cycloartenol synthase; AtCYC; EC=5.4.99.8; 2,3-epoxysqualenecycloartenol cyclase (At2g07050)		1.060
Cs4g05990	K04427 mitogen-activated protein kinase kinase kinase 7 [EC:2.7.11.25] | (RefSeq) serine/threonine-protein kinase STY46-like (A)	Serine/threonine-protein kinase CTR1 {ECO:0000303|PubMed:8431946}; EC=2.7.11.1 {ECO:0000305}; Protein CONSTITUTIVE TRIPLE RESPONSE1 {ECO:0000303|PubMed:8431946} (At5g03730)		-4.296
Cs4g10240	K13789 geranylgeranyl diphosphate synthase, type II [EC:2.5.1.1 2.5.1.10 2.5.1.29] | (RefSeq) hypothetical protein (A)	Geranylgeranyl pyrophosphate synthase, chloroplastic; GGPP synthase; GGPS; EC=2.5.1.-; (2E,6E)-farnesyl diphosphate synthase; Dimethylallyltranstransferase; EC=2.5.1.1; Farnesyl diphosphate synthase; Farnesyltranstransferase; EC=2.5.1.29; Geranyltranstransferase; EC=2.5.1.10; Flags: Precursor (At2g18620)	-1.409	-1.909
Cs4g12050	K15803 (-)-germacrene D synthase [EC:4.2.3.75] | (RefSeq) alpha-copaene synthase-like (A)	(-)-germacrene D synthase; EC=4.2.3.22; EC=4.2.3.75 (At5g23960)	1.032	2.535
Cs4g12450	K15803 (-)-germacrene D synthase [EC:4.2.3.75] | (RefSeq) (E)-beta-farnesene synthase-like (A)	(E)-beta-farnesene synthase; CjFS; EC=4.2.3.47; Terpene synthase 10 (At5g23960)		-1.024
Cs4g16860	K21371 crocetin glucosyltransferase [EC:2.4.1.271] | (RefSeq) crocetin glucosyltransferase, chloroplastic-like (A)	Probable strigolactone esterase DAD2; 3.1.-.-; Protein DECREASED APICAL DOMINANCE 2 (At3g24420)		-3.699
Cs4g18300	K20771 carlactone C-19 oxidase [EC:1.14.-.-] | (RefSeq) hypothetical protein (A)	Cytochrome P450 711A1; EC=1.14.-.-; Protein MORE AXILLARY BRANCHES 1 (At2g26170)	1.187	1.826
Cs4g19460	K17913 carlactone synthase / all-trans-10'-apo-beta-carotenal 13,14-cleaving dioxygenase [EC:1.13.11.69 1.13.11.70] | (RefSeq) hypothetical protein (A)	Carotenoid cleavage dioxygenase 8 homolog B, chloroplastic; OsCCD8b; EC=1.13.11.69 {ECO:0000269|PubMed:22422982}; EC=1.13.11.70 {ECO:0000269|PubMed:18637791}; Protein DWARF-10; Protein MAX4 homolog; Flags: Precursor (At4g32810)	1.942	1.251
Cs5g01410	K17911 beta-carotene isomerase [EC:5.2.1.14] | (RefSeq) beta-carotene isomerase D27, chloroplastic-like isoform X1 (A)	Beta-carotene isomerase D27, chloroplastic; EC=5.2.1.14; Protein DWARF-27 homolog; AtD27; Flags: Precursor (At1g64680)		-1.707
Cs5g07980	K13065 shikimate O-hydroxycinnamoyltransferase [EC:2.3.1.133] | (RefSeq) vinorine synthase-like (A)	Taxadien-5-alpha-ol O-acetyltransferase; EC=2.3.1.162; Taxa-4(20),11(12)-dien-5alpha-ol-O-acetyltransferase; Taxadienol	2.401	3.685

		acetyltransferase (At5g02890)		
Cs5g09460	K00512 steroid 17alpha-monooxygenase / 17alpha-hydroxyprogesterone deacetylase [EC:1.14.14.19 1.14.14.32] | (RefSeq) flavonoid 3'-monooxygenase-like (A)	Geraniol 8-hydroxylase; EC=1.14.13.152; Cytochrome P450 76B6; Geraniol 10-hydroxylase; CrG10H (At4g12300)	1.224	1.620
Cs5g09860	K20556 cytochrome P450 family 76 subfamily C | (RefSeq) hypothetical protein (A)	Geraniol 8-hydroxylase; EC=1.14.13.152; Cytochrome P450 76B6; Geraniol 10-hydroxylase; CrG10H (At2g45550)	2.759	
Cs5g09870	K20556 cytochrome P450 family 76 subfamily C | (RefSeq) hypothetical protein (A)	Geraniol 8-hydroxylase; EC=1.14.13.152; Cytochrome P450 76B6; Geraniol 10-hydroxylase; CrG10H (At2g45550)	2.636	
Cs5g14370	K09840 9-cis-epoxycarotenoid dioxygenase [EC:1.13.11.51] | (RefSeq) hypothetical protein (A)	9-cis-epoxycarotenoid dioxygenase NCED1, chloroplastic; EC=1.13.11.51; PvNCED1; Flags: Precursor (At3g14440)		-1.791
Cs5g14480	K04125 gibberellin 2-oxidase [EC:1.14.11.13] | (RefSeq) hypothetical protein (A)	Gibberellin 2-beta-dioxygenase 1; EC=1.14.11.13; GA 2-oxidase 1; Gibberellin 2-beta-hydroxylase 1; Gibberellin 2-oxidase 1; Protein SLENDER (At1g78440)		-2.992
Cs5g17030		NDR1/HIN1-like protein 6 {ECO:0000303|PubMed:26849212} (At1g54540)		1.780
Cs5g27570	K17961 cytochrome P450 family 82 subfamily G polypeptide 1 [EC:1.14.-.-] | (RefSeq) cytochrome P450 82G1-like (A)	Cytochrome P450 82G1; EC=1.14.-.- (At3g25180)		-2.621
Cs6g07330	K21373 7-deoxyloganetic acid glucosyltransferase [EC:2.4.1.323] | (RefSeq) 7-deoxyloganetic acid glucosyltransferase-like (A)	7-deoxyloganetic acid glucosyltransferase; EC=2.4.1.323; UDP-glucose glucosyltransferase 8; CrUGT8; UDP-glycosyltransferase 709C2 (At1g22400)	1.060	1.522
Cs6g17510	K13789 geranylgeranyl diphosphate synthase, type II [EC:2.5.1.1 2.5.1.10 2.5.1.29] | (RefSeq) geranylgeranyl pyrophosphate synthase 7, chloroplastic-like (A)	Geranylgeranyl pyrophosphate synthase, chloroplastic; GGPP synthase; GGPS; EC=2.5.1.-; (2E,6E)-farnesyl diphosphate synthase; Dimethylallyltranstransferase; EC=2.5.1.1; Farnesyl diphosphate synthase; Farnesyltranstransferase; EC=2.5.1.29; Geranyltranstransferase; EC=2.5.1.10; Flags: Precursor (At4g36810)		-6.069
Cs7g02690		Protein NEOXANTHIN-DEFICIENT 1 {ECO:0000303|PubMed:24506237} (At1g28100)	-1.075	
Cs7g04930	K09841 xanthoxin dehydrogenase [EC:1.1.1.288] | (RefSeq) secoisolariciresinol dehydrogenase-like (A)	(-)-isopiperitenol/(-)-carveol dehydrogenase, mitochondrial; EC=1.1.1.223; EC=1.1.1.243; Flags: Precursor (At2g47140)		-1.401
Cs7g09440	K16285 RING/U-box domain-containing protein [EC:2.3.2.27] | (RefSeq) hypothetical protein (A)	Probable E3 ubiquitin-protein ligase XERICO; EC=2.3.2.27; RING-type E3 ubiquitin transferase XERICO (At2g04240)	1.393	1.416
Cs7g14940	K04125 gibberellin 2-oxidase [EC:1.14.11.13] | (RefSeq) gibberellin 2-beta-dioxygenase 8-like (A)	Gibberellin 2-beta-dioxygenase 8; EC=1.14.11.13; GA 2-oxidase 8; Gibberellin 2-beta-hydroxylase 8; Gibberellin 2-oxidase 8 (At4g21200)		1.683

Cs7g30820	K15400 omega-hydroxypalmitate O-feruloyl transferase [EC:2.3.1.188] | (RefSeq) omega-hydroxypalmitate O-feruloyl transferase-like (A)	Taxadien-5-alpha-ol O-acetyltransferase; EC=2.3.1.162; Taxa-4(20),11(12)-dien-5alpha-ol-O-acetyltransferase; Taxadienol acetyltransferase (At2g40230)	-1.477	-2.357
Cs8g09390	K20556 cytochrome P450 family 76 subfamily C | (RefSeq) hypothetical protein (A)	Geraniol 8-hydroxylase; EC=1.14.13.152; Cytochrome P450 76B6; Geraniol 10-hydroxylase; CrG10H (At2g45550)	6.350	
Cs8g09720	K20556 cytochrome P450 family 76 subfamily C | (RefSeq) hypothetical protein (A)	Geraniol 8-hydroxylase; EC=1.14.13.152; Cytochrome P450 76B6; Geraniol 10-hydroxylase; CrG10H (At2g45550)	1.255	-1.498
Cs8g09780	K20556 cytochrome P450 family 76 subfamily C | (RefSeq) hypothetical protein (A)	Geraniol 8-hydroxylase; EC=1.14.13.152; Cytochrome P450 76B6; Geraniol 10-hydroxylase; CrG10H (At2g45550)	5.546	
Cs8g13770	K09842 abscisic-aldehyde oxidase [EC:1.2.3.14] | (RefSeq) indole-3-acetaldehyde oxidase-like (A)	Indole-3-acetaldehyde oxidase; IAA oxidase; EC=1.2.3.7; Aldehyde oxidase 1; AO-1; AtAO-1; AtAO1 (At5g20960)		1.072
Cs8g13780	K09842 abscisic-aldehyde oxidase [EC:1.2.3.14] | (RefSeq) abscisic-aldehyde oxidase-like (A)	Abscisic-aldehyde oxidase; EC=1.2.3.14; Aldehyde oxidase 3; AO-3; AtAO-3; AtAO4; Indole-3-acetaldehyde oxidase; IAA oxidase; EC=1.2.3.7 (At2g27150)		1.068
Cs8g19280	K04628 ceramide galactosyltransferase [EC:2.4.1.47] | (RefSeq) hypothetical protein (A)	UDP-glucose iridoid glucosyltransferase; EC=2.4.1.-; UDP-glucose glucosyltransferase 7; CrUGT7; UDP-glycosyltransferase 76A2 (At3g46670)		-1.023
Cs8g20950	K15813 beta-amyrin synthase [EC:5.4.99.39] | (RefSeq) beta-amyrin synthase-like (A)	Beta-amyrin synthase; EC=5.4.99.39 (At1g78950)	2.931	3.980
Cs9g01770	K04125 gibberellin 2-oxidase [EC:1.14.11.13] | (RefSeq) gibberellin 2-beta-dioxygenase 2-like (A)	Gibberellin 2-beta-dioxygenase 2; EC=1.14.11.13; GA 2-oxidase 2; Gibberellin 2-beta-hydroxylase 2; Gibberellin 2-oxidase 2 (At1g47990)		1.084
Cs9g16520	K05282 gibberellin 20-oxidase [EC:1.14.11.12] | (RefSeq) gibberellin 20 oxidase 1 (A)	Gibberellin 20 oxidase 1; EC=1.14.11.-; GA 20-oxidase 1; AtGA20ox1; Gibberellin C-20 oxidase 1 (At4g25420)		-1.697
Cs9g19270	K15746 beta-carotene 3-hydroxylase [EC:1.14.15.24] | (RefSeq) hypothetical protein (A)	Beta-carotene 3-hydroxylase, chloroplastic; GenCHYB; EC=1.14.15.24 {ECO:0000250|UniProtKB:Q9SZZ8}; Flags: Precursor (At5g52570)		-1.790
novel.1333	K13789 geranylgeranyl diphosphate synthase, type II [EC:2.5.1.1 2.5.1.10 2.5.1.29] | (RefSeq) geranylgeranyl pyrophosphate synthase, chloroplastic-like (A)	GGPPS_HEVBR Geranylgeranyl pyrophosphate synthase, chloroplastic; GGPP synthase; GGPS; EC=2.5.1.-; (2E,6E)-farnesyl diphosphate synthase; Dimethylallyltranstransferase; EC=2.5.1.1; Farnesyl diphosphate synthase; Farnesyltranstransferase; EC=2.5.1.29; Geranyltranstransferase; EC=2.5.1.10; Flags: Precursor (At4g36810)		-1.435
orange1.1t048	K09838 zeaxanthin epoxidase [EC:1.14.15.21] | (RefSeq)	Zeaxanthin epoxidase, chloroplastic; AtZEP; EC=1.14.15.21; Protein	3.690	

49	Zeaxanthin epoxidase, chloroplastic (A)	ABA DEFICIENT 1; AtABA1; Protein IMPAIRED IN BABA-INDUCED STERILITY 3; Protein LOW EXPRESSION OF OSMOTIC STRESS-RESPONSIVE GENES 6; Protein NON-PHOTOCHEMICAL QUENCHING 2; Flags: Precursor (At5g11330)		
orange1.1t04957	K13789 geranylgeranyl diphosphate synthase, type II [EC:2.5.1.1 2.5.1.10 2.5.1.29] | (RefSeq) geranylgeranyl pyrophosphate synthase, chloroplastic/chromoplastic-like (A)	Geranylgeranyl pyrophosphate synthase, chloroplastic; GGPP synthase; GGPS; EC=2.5.1.-; (2E,6E)-farnesyl diphosphate synthase; Dimethylallyltranstransferase; EC=2.5.1.1; Farnesyl diphosphate synthase; Farnesyltranstransferase; EC=2.5.1.29; Geranyltranstransferase; EC=2.5.1.10; Flags: Precursor (At4g36810)		-3.818
orange1.1t05125	K09838 zeaxanthin epoxidase [EC:1.14.15.21] | (RefSeq) Zeaxanthin epoxidase, chloroplastic (A)	Zeaxanthin epoxidase, chloroplastic; EC=1.14.15.21; Flags: Precursor (At5g11330)	3.049	
Terpenoid catabolic process [GO:0016115; P = 0.3242 (P3CR vs. P3R) and 0.0081 (P5CR vs. P5R)]				
Cs1g24480	K07437 cytochrome P450 family 26 subfamily A | (RefSeq) cytochrome P450 90A1-like (A)	Abscisic acid 8'-hydroxylase 3; ABA 8'-hydroxylase 3; EC=1.14.13.93; Cytochrome P450 707A7; OsABA8ox3 (At1g19630)		1.028
Cs1g25490	K07437 cytochrome P450 family 26 subfamily A | (RefSeq) cytochrome P450 90A1 (A)	Abscisic acid 8'-hydroxylase 1; ABA 8'-hydroxylase 1; EC=1.14.13.93; Cytochrome P450 707A1 (At1g19630)	3.253	
Cs3g21210	K07437 cytochrome P450 family 26 subfamily A | (RefSeq) abscisic acid 8'-hydroxylase 1-like (A)	Abscisic acid 8'-hydroxylase 3; ABA 8'-hydroxylase 3; EC=1.14.13.93; Cytochrome P450 707A3 (At5g45340)	1.449	-3.358
Cs3g23530	K09843 (+)-abscisic acid 8'-hydroxylase [EC:1.14.13.93] | (RefSeq) hypothetical protein (A)	Abscisic acid 8'-hydroxylase 3; ABA 8'-hydroxylase 3; EC=1.14.13.93; Cytochrome P450 707A7; OsABA8ox3 (At3g19270)		-1.446
Cs6g19380	K09843 (+)-abscisic acid 8'-hydroxylase [EC:1.14.13.93] | (RefSeq) CYP707A1; abscisic acid 8'-hydroxylase 1-like (A)	Abscisic acid 8'-hydroxylase 1; ABA 8'-hydroxylase 1; EC=1.14.13.93; Cytochrome P450 707A1 (At5g45340)		1.560
Cs7g01700	K00465 carotenoid 9,10(9',10')-cleavage dioxygenase 1 [EC:1.13.11.-] | (RefSeq) carotenoid 9,10(9',10')-cleavage dioxygenase 1-like (A)	Carotenoid 9,10(9',10')-cleavage dioxygenase 1; EC=1.14.99.n4; AtCCD1; Neoxanthin cleavage enzyme NC1; AtNCED1 (At3g63520)		-1.066
Cs8g05940	K09843 (+)-abscisic acid 8'-hydroxylase [EC:1.14.13.93] | (RefSeq) CYP707A3; abscisic acid 8'-hydroxylase 4-like (A)	Abscisic acid 8'-hydroxylase 4; ABA 8'-hydroxylase 4; EC=1.14.13.93; Cytochrome P450 707A4 (At3g19270)		-1.037
Salicylic acid biosynthetic process [GO:0009697; P = 0.5284 (P3CR vs. P3R) and 0.0238 (P5CR vs. P5R)]				

Cs7g27120	K21989 calcium permeable stress-gated cation channel | (RefSeq) CSC1-like protein RXW8 (A)	Protein SAR DEFICIENT 1 {ECO:0000303|PubMed:20921422} (At1g73800)	2.272	1.128
Cs6g12050	K04733 interleukin-1 receptor-associated kinase 4 [EC:2.7.11.1] | (RefSeq) probable serine/threonine-protein kinase PBL19 (A)	Serine/threonine-protein kinase PCRK1 {ECO:0000305}; EC=2.7.11.1 {ECO:0000269|PubMed:25711411}; Protein PTI-COMPROMISED RECEPTOR-LIKE CYTOPLASMIC KINASE 1 {ECO:0000303|PubMed:25711411} (At3g09830)		1.519
Cs5g29440	K16275 E3 ubiquitin-protein ligase BAH [EC:2.3.2.27] | (RefSeq) hypothetical protein (A)	E3 ubiquitin-protein ligase BAH1; EC=2.3.2.27 {ECO:0000305}; Protein BENZOIC ACID HYPERSENSITIVE 1; Protein NITROGEN LIMITATION ADAPTATION; RING-type E3 ubiquitin transferase BAH1 {ECO:0000305} (At1g02860)		1.226
Cs1g07440		Protein GRIM REAPER {ECO:0000303|PubMed:19279211}; Stigma-specific STIG1-like protein GRI {ECO:0000303|PubMed:19279211}; Contains: GRIp {ECO:0000303|PubMed:25398910}; Flags: Precursor (At1g53130)		1.169
Salicylic acid catabolic process [GO:0046244; P = 0.0097 (P3CR vs. P3R) and 0.1857 (P5CR vs. P5R)				
Cs9g14480	K00475 naringenin 3-dioxygenase [EC:1.14.11.9] | (RefSeq) naringenin,2-oxoglutarate 3-dioxygenase (A)	Protein DMR6-LIKE OXYGENASE 2 {ECO:0000303|PubMed:25376907}; EC=1.14.11.- {ECO:0000255|PROSITE-ProRule:PRU00805}; 2-oxoglutarate (2OG)-Fe(II) oxygenase-like protein DLO2 {ECO:0000303|PubMed:25376907}; Salicylate 3-hydroxylase DLO2 {ECO:0000305}; S3H DLO2 {ECO:0000305}; SA 3-hydroxylase DLO2 {ECO:0000305}; Salicylic acid 3-hydroxylase DLO2 {ECO:0000305}; EC=1.14.13.- {ECO:0000250|UniProtKB:Q9ZSA8} (At4g10490)	4.517	
Cs5g28710	K05282 gibberellin-44 dioxygenase [EC:1.14.11.12] | (RefSeq) gibberellin 20 oxidase 1-D-like (A)	Protein DMR6-LIKE OXYGENASE 1 {ECO:0000303|PubMed:25376907}; EC=1.14.11.- {ECO:0000255|PROSITE-ProRule:PRU00805}; 2-oxoglutarate (2OG)-Fe(II) oxygenase-like protein DLO1 {ECO:0000303|PubMed:25376907}; Protein SENESCENCE-ASSOCIATED GENE 108 {ECO:0000303|PubMed:23959884}; Salicylate 3-hydroxylase DLO1 {ECO:0000303|PubMed:23959884}; S3H DLO1 {ECO:0000303|PubMed:23959884}; SA 3-hydroxylase DLO1	2.636	

		{ECO:0000303|PubMed:23959884}; Salicylic acid 3-hydroxylase DLO1 {ECO:0000303|PubMed:23959884}; EC=1.14.13.- {ECO:0000269|PubMed:23959884} (At3g19000)		
Cs1g12300	K18054 2'-deoxymugineic-acid 2'-dioxygenase / mugineic-acid 3-dioxygenase [EC:1.14.11.24 1.14.11.25] | (RefSeq) 2'-deoxymugineic-acid 2'-dioxygenase-like (A)	Protein DMR6-LIKE OXYGENASE 2 {ECO:0000303|PubMed:25376907}; EC=1.14.11.- {ECO:0000255|PROSITE-ProRule:PRU00805}; 2-oxoglutarate (2OG)-Fe(II) oxygenase-like protein DLO2 {ECO:0000303|PubMed:25376907}; Salicylate 3-hydroxylase DLO2 {ECO:0000305}; S3H DLO2 {ECO:0000305}; SA 3-hydroxylase DLO2 {ECO:0000305}; Salicylic acid 3-hydroxylase DLO2 {ECO:0000305}; EC=1.14.13.- {ECO:0000250|UniProtKB:Q9ZSA8} (At4g10490)	2.697	
Cs9g14500	K06892 feruloyl-CoA ortho-hydroxylase [EC:1.14.11.-] | (RefSeq) feruloyl CoA ortho-hydroxylase 1 (A)	Protein DMR6-LIKE OXYGENASE 2 {ECO:0000303|PubMed:25376907}; EC=1.14.11.- {ECO:0000255|PROSITE-ProRule:PRU00805}; 2-oxoglutarate (2OG)-Fe(II) oxygenase-like protein DLO2 {ECO:0000303|PubMed:25376907}; Salicylate 3-hydroxylase DLO2 {ECO:0000305}; S3H DLO2 {ECO:0000305}; SA 3-hydroxylase DLO2 {ECO:0000305}; Salicylic acid 3-hydroxylase DLO2 {ECO:0000305}; EC=1.14.13.- {ECO:0000250|UniProtKB:Q9ZSA8} (At4g10490)	5.040	
Cs9g14520	K05278 flavonol synthase [EC:1.14.11.23] | (RefSeq) flavonol synthase/flavanone 3-hydroxylase (A)	Protein DMR6-LIKE OXYGENASE 2 {ECO:0000303|PubMed:25376907}; EC=1.14.11.- {ECO:0000255|PROSITE-ProRule:PRU00805}; 2-oxoglutarate (2OG)-Fe(II) oxygenase-like protein DLO2 {ECO:0000303|PubMed:25376907}; Salicylate 3-hydroxylase DLO2 {ECO:0000305}; S3H DLO2 {ECO:0000305}; SA 3-hydroxylase DLO2 {ECO:0000305}; Salicylic acid 3-hydroxylase DLO2 {ECO:0000305}; EC=1.14.13.- {ECO:0000250|UniProtKB:Q9ZSA8} (At4g10490)	6.224	
Cs5g28780	K05282 gibberellin-44 dioxygenase [EC:1.14.11.12] | (RefSeq) gibberellin 20 oxidase 1-like isoform X1 (A)	Protein DMR6-LIKE OXYGENASE 2 {ECO:0000303|PubMed:25376907}; EC=1.14.11.- {ECO:0000255|PROSITE-ProRule:PRU00805}; 2-oxoglutarate (2OG)-Fe(II) oxygenase-like protein DLO2		1.368

		{ECO:0000303|PubMed:25376907}; Salicylate 3-hydroxylase DLO2 {ECO:0000305}; S3H DLO2 {ECO:0000305}; SA 3-hydroxylase DLO2 {ECO:0000305}; Salicylic acid 3-hydroxylase DLO2 {ECO:0000305}; EC=1.14.13.- {ECO:0000250|UniProtKB:Q9ZSA8} (At3g19000)		
Cs5g16310	K05278 flavonol synthase [EC:1.14.11.23] | (RefSeq) flavonol synthase/flavanone 3-hydroxylase (A)	Protein DOWNY MILDEW RESISTANCE 6 {ECO:0000303|PubMed:15986928}; AtDMR6 {ECO:0000303|PubMed:15986928}; EC=1.14.11.- {ECO:0000255|PROSITE-ProRule:PRU00805}; 2-oxoglutarate (2OG)-Fe(II) oxygenase-like protein DMR6 {ECO:0000303|PubMed:18248595}; Salicylate 3-hydroxylase DMR6 {ECO:0000305}; S3H DMR6 {ECO:0000305}; SA 3-hydroxylase DMR6 {ECO:0000305}; Salicylic acid 3-hydroxylase DMR6 {ECO:0000305}; EC=1.14.13.- {ECO:0000250|UniProtKB:Q9ZSA8} (At5g24530)		-1.118
orange1.1t01032	K06892 feruloyl-CoA ortho-hydroxylase [EC:1.14.11.-] | (RefSeq) 2-oxoglutarate-iron(II)-dependent oxygenase (A)	Protein DMR6-LIKE OXYGENASE 1 {ECO:0000303|PubMed:25376907}; EC=1.14.11.- {ECO:0000255|PROSITE-ProRule:PRU00805}; 2-oxoglutarate (2OG)-Fe(II) oxygenase-like protein DLO1 {ECO:0000303|PubMed:25376907}; Protein SENESCENCE-ASSOCIATED GENE 108 {ECO:0000303|PubMed:23959884}; Salicylate 3-hydroxylase DLO1 {ECO:0000303|PubMed:23959884}; S3H DLO1 {ECO:0000303|PubMed:23959884}; SA 3-hydroxylase DLO1 {ECO:0000303|PubMed:23959884}; Salicylic acid 3-hydroxylase DLO1 {ECO:0000303|PubMed:23959884}; EC=1.14.13.- {ECO:0000269|PubMed:23959884} (At2g36690)		1.348
Cs1g12310	K18054 2'-deoxymugineic-acid 2'-dioxygenase / mugineic-acid 3-dioxygenase [EC:1.14.11.24 1.14.11.25] | (RefSeq) 2'-deoxymugineic-acid 2'-dioxygenase-like (A)	Protein DMR6-LIKE OXYGENASE 1 {ECO:0000303|PubMed:25376907}; EC=1.14.11.- {ECO:0000255|PROSITE-ProRule:PRU00805}; 2-oxoglutarate (2OG)-Fe(II) oxygenase-like protein DLO1 {ECO:0000303|PubMed:25376907}; Protein SENESCENCE-ASSOCIATED GENE 108 {ECO:0000303|PubMed:23959884}; Salicylate 3-hydroxylase		-1.609

		DLO1 {ECO:0000303|PubMed:23959884}; S3H DLO1 {ECO:0000303|PubMed:23959884}; SA 3-hydroxylase DLO1 {ECO:0000303|PubMed:23959884}; Salicylic acid 3-hydroxylase DLO1 {ECO:0000303|PubMed:23959884}; EC=1.14.13.- {ECO:0000269|PubMed:23959884} (At4g10500)		
**Index**	**Compounds**		**Log2(FC)**	
			P3CR vs. P3R	P5CR vs. P5R
Biosynthesis of secondary metabolites [ko01110; P = 0.5952 (P3CR vs. P3R) and 0.4188 (P5CR vs. P5R)]				
HJN003	1-O-Sinapoyl-D-glucose		-1.239	1.036
Hmln002806	5-O-Caffeoylshikimic acid		1.260	2.066
Lmbp000123	L-Homomethionine		-1.720	
Lmgn001670	Salicylic acid		1.882	
Lmmn001643	2-Hydroxycinnamic acid		1.044	
MA10107783	3-[(1-Carboxyvinyl)oxy]benzoic Acid		1.618	
mws0014	Ferulic acid		1.230	
mws0093	coniferyl alcohol		1.076	
mws0183	3,4-Dihydroxybenzoic Acid (Protocatechuic acid)		4.283	
mws0230	L-Threonine			1.615
mws0254	L-Histidine			-1.003
mws0367	α-Linolenic Acid			-1.054
mws0458	Vanillin		1.355	
mws0639	2,3-Dihydroxybenzoic Acid		4.159	
mws0668	Xanthosine		-2.360	
mws0671	L-Homoserine		2.706	2.457
mws0749	4-Hydroxybenzoic Acid		1.881	
mws0853	Sinapyl alcohol		2.748	
mws0906	coniferyl alcohol-4-O-glucoside (Coniferin)			1.652
mws1024	p-Coumaraldehyde		1.213	
mws1090	Glucose-1-phosphate		-1.032	
mws1337	D-Pantothenic Acid		-1.179	
mws1375	Nicotianamine		-3.369	

mws2212	Caffeic acid		1.147	
mws2523	Trehalose 6-phosphate			1.198
mws4052	1-Aminocyclopropane-1-carboxylic acid		-1.630	
mws4085	Sinapic acid		1.084	
mws4170	D-Glucose		1.172	
MWS5083	Flavin Single Nucleotide(FMN)		-1.351	
MWSCX014	Scopoletin (7-Hydroxy-6-methoxycoumarin)		1.492	
MWSmce160	Demethylsuberosin		-1.336	
MWSmce324	Psoralen		1.292	
MWStz103	S-(5'-Adenosyl)-L-methionine		-2.702	
pma6455	Ribulose-5-phosphate			1.628
pmb0532	Inosine 5'-monophosphate		-2.111	
pmb0751	Trans-5-O-(p-Coumaroyl)shikimate		-1.645	
pmb0981	Adenosine 5'-monophosphate		-1.974	
pmb1096	Indole		1.124	
pmb2791	9-Hydroperoxy-10E,12,15Z-octadecatrienoic acid			-3.049
pmb2922	Uridine 5'-diphospho-D-glucose		-4.189	
pme0014	L-Glutamic acid			1.320
pme0026	L-Lysine		-1.155	
pme0195	L-Cysteine			-1.032
pme1210	L-Methionine		-1.208	
pme1654	Jasmonic acid		-1.343	
pme1841	Cadaverine			-1.674
pme2117	Adenosine 5'-diphosphate		-1.571	
pme2292	Putrescine		1.171	
pme2746	Riboflavin 5'-Adenosine Diphosphate		-1.572	1.076
pme3311	D-Fructose-1,6-biphosphate		11.622	
pme3313	D-Fructose 6-phosphate		-1.146	1.037
pme3443	Sinapinaldehyde		2.112	
pmn001689	9-Hydroxy-12-oxo-15(Z)-octadecenoic acid			-2.539
Zmyn000453	Isocitric Acid		-2.158	
Zmyn004449	9-Hydroxy-12-oxo-10(E),15(Z)-octadecadienoic acid		3.009	
Zmzn000078	Dihydroxyacetone phosphate			1.353

Zmzn000079	D-Erythrose-4-phosphate			1.247
Phenylpropanoid biosynthesis [ko00940; P = 0. 1441 (P3CR vs. P3R) and 0.5504 (P5CR vs. P5R)]				
HJN003	1-O-Sinapoyl-D-glucose		-1.239	1.036
Hmln002806	5-O-Caffeoylshikimic acid		1.260	2.066
mws0014	Ferulic acid		1.230	
mws0093	coniferyl alcohol		1.076	
mws0853	Sinapyl alcohol		2.748	
mws0906	coniferyl alcohol-4-O-glucoside (Coniferin)			1.652
mws1024	p-Coumaraldehyde		1.213	
mws2212	Caffeic acid		1.147	
mws4085	Sinapic acid		1.084	
pmb0751	Trans-5-O-(p-Coumaroyl)shikimate		-1.645	
pme3443	Sinapinaldehyde		2.112	
Flavonoid biosynthesis [ko00941; a P = 0.8551 (P3CR vs. P3R) and 0.7052 (P5CR vs. P5R)]				
Hmln002806	5-O-Caffeoylshikimic acid		1.260	2.066
pmb0751	Trans-5-O-(p-Coumaroyl)shikimate		-1.645	
Isoquinoline alkaloid biosynthesis [ko00950; P = 0.8389 (P3CR vs. P3R)]				
Hmgn001653	Protocatechualdehyde		1.981	
Tropane, piperidine and pyridine alkaloid biosynthesis [ko00960; P = 0.8996 (P3CR vs. P3R) and 0.7476 (P5CR vs. P5R)]				
pme0026	L-Lysine		-1.155	
pme1841	Cadaverine			-1.674
pme2292	Putrescine		1.171	
Indole alkaloid biosynthesis [ko00901; P = 0.3646 (P3CR vs. P3R)]				
mws4170	D-Glucose		1.172	
