# Supplementary material for: Mechanisms by Which Increased pH Ameliorates Copper Excess in Citrus sinensis Roots: Insight from a Combined Analysis of Physiology, Transcriptome, and Metabolome
Source: Plants (Basel). 2024 Oct 31;13(21):3054. doi: 10.3390/plants13213054 (PMC11548300; doi:10.3390/plants13213054)
Supplement: Supplementary file 1 [file plants-13-03054-s001.zip › Table S1SecondRevision.pdf]

Table S1. Summary of the RNA-Seq data collected from control and Cu-toxic roots of *C. sinensis* seedlings. Q20 and Q30 mean sequencing error rates lower than 1% and 1‰, respectively.

Samples	Raw reads	Clean reads (%)	Clean bases (G)	Error rate (%)	Q20 (%)	Q30 (%)	Adapter (%)	N (%)	Low quality (%)	GC content (%)
P3R-1	47204340	44577432(94.44）	6.69	0.03	97.67	93.31	5.35	0	0.22	44.31
P3R-2	46007670	43807874(95.22)	6.57	0.03	97.65	93.24	4.55	0	0.22	44.44
P3R-3	46359420	43969072(94.84)	6.6	0.03	97.63	93.14	4.93	0	0.23	44.28
P5R-1	45675834	43245306(94.68)	6.49	0.03	97.61	93.12	5.07	0	0.24	44.22
P5R-2	43936214	41542740(94.55)	6.23	0.03	97.61	93.15	5.21	0	0.23	44.05
P5R-3	56963646	53364872(93.68)	8.00	0.03	97.64	93.18	6.11	0	0.21	44.04
P3CR-1	45387604	42687060(94.05)	6.40	0.03	97.59	93.02	5.76	0	0.19	44.15
P3CR-2	55126130	52090598(94.49)	7.81	0.03	97.47	92.75	5.28	0	0.23	44.13
P3CR-3	53231050	50505904(94.88)	7.58	0.03	97.66	93.20	4.92	0	0.20	44.23
P5CR-1	44500108	41880318(94.11)	6.28	0.03	97.48	92.75	5.65	0	0.23	44.42
P5CR-2	49814742	46918084(94.19)	7.04	0.03	97.60	93.02	5.57	0	0.24	44.27
P5CR-3	44731800	42092206(94.1)	6.31	0.03	97.60	93.06	5.66	0	0.24	44.00

Q20 and Q30 mean sequencing error rates lower than 1% and 1‰, respectively.
