# Supplementary material for: Mechanisms by Which Increased pH Ameliorates Copper Excess in Citrus sinensis Roots: Insight from a Combined Analysis of Physiology, Transcriptome, and Metabolome
Source: Plants (Basel). 2024 Oct 31;13(21):3054. doi: 10.3390/plants13213054 (PMC11548300; doi:10.3390/plants13213054)
Supplement: Supplementary file 1 [file plants-13-03054-s001.zip › Table S20SeconRevision.pdf]

Table S20. Differentially abundant phenylpropanoids detected in P3CR vs. P3R and/or P5CR vs. P5R

**Index (Formula)**	**Compounds**	**Log_2(FC)**	
		P3CR vs. P3R	P5CR vs. P5R
Chalcones			
pmp000801(C19H20O4)	Isobavachalcone A	3.491	
pmp000802(C19H20O4)	Isobavachalcone B	3.500	
Lmjp003722(C20H22O7)	2'-Hydroxy-3,4,5,4',6'-pentamethoxychalcone	-2.567	
MWSslk181(C21H22O9)	Isoliquiritin		1.846
Flavanones			
MWSHY0138(C21H22O4)	Bavachinin	1.155	
pmp000637(C20H20O5)	Sophoraflavanone B	2.769	
Wmkn003942(C21H22O6)	2'-HydroxyIsoxanthohumol	11.562	
pmb2984(C23H24O12)	Eriodictyol-7-O-(6"-acetyl)glucoside	10.193	
pmb2979(C25H26O14)	Hesperetin-7-O-(6"-malonyl)glucoside	-1.058	
HJAP020(C27H32O16)	Eriodictyol-5,3'-Di-O-glucoside	-2.272	-1.917
Flavones			
Zmjp006969(C17H14O6)	Pectolinarigenin	10.714	
pmb0736(C23H24O12)	tricin-7-O-Glucoside	-2.668	
Zmjp004852(C23H24O13)	5,6,3′,4′-Tetrahydroxy-3,7-dimethoxyflavone-6-O-glucoside	12.104	
pmb0608(C25H24O14)	Chrysoeriol-7-O-(6"-malonyl)glucoside	-2.094	
Flavonols			
mws0038(C17H14O6)	Kumatakenin	2.347	
pmn001551(C21H22O9)	Natsudaidain (3-Hydroxy-3',4',5,6,7,8-hexamethoxyflavone)	4.886	2.991
pmp000976(C27H30O10)	Baohuoside I; Icariside II		-1.112
Flavonoid carbonoside			
pmp000236(C26H28O14)	Isovitexin-8-O-xylcoside	-1.298	
mws1073(C27H30O15)	Apigenin-6,8-di-C-glucoside (Vicenin-2)	1.431	
Flavanols			
Cmsp007228(C16H14O6)	7-O-Methyleriodictyol	-1.105	
mws0355(C22H18O10)	Catechin gallate		-2.527
mws1397(C22H18O10)	Epicatechin gallate	11.304	
pmn001527(C22H32O12)	Di-O-galloyl Methyl gallate	-11.000	
Isoflavones			
Lmgp004829(C23H24O12)	5,7,4'-Trihydroxy-6,8-dimethoxyisoflavone-7-O-galactoside	-7.808	
Lignans			
HX1369(C20H24O6)	(-)-Lariciresinol	-1.860	
HX1371(C20H24O6)	(7S,8R)-dihydrodehydrodiconferyl alcohol	-1.846	
Qmzp101909(C21H22O7)	Magnolone	8.782	7.649
HX1288(C19H30O8)	Citroside A	1.004	-1.148
Hmcn002743(C22H26O8)	Lirioresinol A	2.872	
MWS20152(C22H26O8)	syringaresinol	2.951	
mws2162(C24H32O7)	Schisandrol A	4.483	1.580
Ymjm000135(C25H32O11)	8R-Dihydrodehydrodiconferyl alcohol 4-O-β-D-glucopyranoside	-1.632	-2.100
Lmtn003096(C26H36O11)	Secoisolariciresinol 4-O-glucoside	12.351	11.306
Lmnn102709(C26H34O12)	(-)-olivil 4'-O-bate-D-glucopyranoside	-1.734	
Hmln002355(C27H34O12)	5'-Methoxymatairesinoside	-1.315	

Lmmn002260(C27H36O12)	5'-Methoxyisolariciresinol-9'-O-glucoside	-2.089	
Lmqn001932(C28H36O13)	syringaresinol-4'-O-glucoside	1.348	
Zmhn001446(C28H36O13)	syringaresinol-4'-O-glucopyranosid	2.614	1.231
Rfmb26201(C30H38O14)	syringaresinol-4'-O-(6''-acetyl)glucoside	2.929	
Coumarins			
MWSmce439(C9H8O2)	3,4-Dihydrocoumarin	1.392	
mws0987(C10H8O2)	6-MethylCoumarin	-1.239	
mws0139(C10H8O3)	7-Hydroxy-4-methylcoumarin	2.016	
mws1074(C9H6O4)	Daphnetin	1.040	
Lhqp101815(C11H6O3)	Bakuchicin	-2.314	
MWSmce324(C11H6O3)	Psoralen	1.292	
HX1363(C10H8O4)	isoscopoletin	1.389	
Hmcp002123(C10H8O4)	Isoscopoletin (6-Hydroxy-7-Methoxycoumarin)	1.681	
MWSCX014(C10H8O4)	Scopoletin (7-Hydroxy-6-methoxycoumarin)	1.492	
pmp000284(C11H10O5)	Fraxidin (8-Hydroxy-6,7-dimethoxycoumarin)	3.190	
mws1639(C11H10O5)	Isofraxidin	2.958	
MWSmce160(C14H14O3)	Demethylsuberosin	-1.336	
Lmjp101203(C15H14O4)	phebalosin	-1.524	
HX1386(C15H14O4)	braylin		-1.119
Zmnn010135(C15H16O4)	Sibiricol	1.344	
Cmpp005475(C15H16O4)	Meranzin	4.816	
Cmpp007452(C15H16O4)	Auraptenol	1.239	
Lmwp102713(C14H16O5)	(S)-Peucedanol	2.231	
pmf0526(C16H14O4)	Isoimperatorin	-17.045	
Lmjp101205(C16H18O4)	8-Isopentenyllimettin		-1.055
Lmjp101207(C15H16O5)	(S)-Murpanidin	1.280	
MWSmce224(C19H22O3)	Auraptene	-4.378	-5.790
pmp000022(C16H16O6)	Oxypeucedanin hydrate		-1.622
Qmzp101840(C19H22O4)	Epoxyaurapten	1.572	
mws1015(C15H16O9)	Esculin (6,7-DihydroxyCoumarin-6-glucoside)	-2.613	
Cmyn001328(C15H16O9)	Daphnin	-2.873	
Lmbn001162(C15H16O9)	Esculetin-7-O-glucoside	-1.306	
Lmbp003208(C17H20O10)	Fraxidin-8-O-glucoside	1.622	3.006
Lmjp003090(C17H20O10)	Isofraxidin-7-O-glucoside	2.756	1.612
Cmpp004617(C26H32O8)	Deacetylnomilin		1.920
Cmpp004946(C26H32O8)	Isoobacunoic acid		1.825
Cmpp004143(C26H34O9)	Deacetylnomilinic acid		2.028
Total	Upregulated metabolites	38	11
	Downregulated metabolites	24	9
