# Supplementary material for: Mechanisms by Which Increased pH Ameliorates Copper Excess in Citrus sinensis Roots: Insight from a Combined Analysis of Physiology, Transcriptome, and Metabolome
Source: Plants (Basel). 2024 Oct 31;13(21):3054. doi: 10.3390/plants13213054 (PMC11548300; doi:10.3390/plants13213054)
Supplement: Supplementary file 1 [file plants-13-03054-s001.zip › Table S21SecondRevision.pdf]

Table S21. Differentially abundant phenolic compounds detected in P3CR vs. P3R and/or P5CR vs. P5R

Index (Formula)	Compounds	$Log_2(FC)$	
		P3CR vs. P3R	P5CR vs. P5R
**Phenols**			
Organic acids			
MWS4396(C11H8O3)	1-Hydroxy-2-Naphthoate	4.040	1.494
MWS2073(C8H6Cl2O3)	2,4-Dichlorophenoxyacetic Acid	1.428	
Chalcones			
pmp000801(C19H20O4)	Isobavachalcone A	3.491	
pmp000802(C19H20O4)	Isobavachalcone B	3.500	
Lmjp003722(C20H22O7)	2'-Hydroxy-3,4,5,4',6'-pentamethoxychalcone	-2.567	
MWSslk181(C21H22O9)	Isoliquiritin		1.846
Anthraquinone			
pmp000601(C19H18O7)	Chryso obtusin		
pmn001340(C23H22O11)	Emodin-8-O-(6''-O-acetyl)glucoside	-1.046	
Zmdn006214(C24H24O11)	Physcion-8-0-(6-acetyl)-glucoside		-1.376
Flavanones			
MWSHY0138(C21H22O4)	Bavachinin	1.155	
pmp000637(C20H20O5)	Sophoraflavanone B	2.769	
Wmkn003942(C21H22O6)	2'-HydroxyIsoxanthohumol	11.562	
pmb2984(C23H24O12)	Eriodictyol-7-O-(6''-acetyl)glucoside	10.193	
pmb2979(C25H26O14)	Hesperetin-7-O-(6''-malonyl)glucoside	-1.058	
HJAP020(C27H32O16)	Eriodictyol-5,3'-Di-O-glucoside	-2.272	-1.917
Flavones			
Zmjp006969(C17H14O6)	Pectolinarigenin	10.714	
pmb0736(C23H24O12)	tricin-7-O-Glucoside	-2.668	
Zmjp004852(C23H24O13)	5,6,3′,4′-Tetrahydroxy-3,7-dimethoxyflavone-6-O-glucoside	12.104	
pmb0608(C25H24O14)	Chrysoeriol-7-O-(6''-malonyl)glucoside	-2.094	
Flavonols			
mws0038(C17H14O6)	Kumatakenin	2.347	
pmn001551(C21H22O9)	Natsudaidain (3-Hydroxy-3',4',5,6,7,8-hexamethoxyflavone)	4.886	2.991
pmp000976(C27H30O10)	Baohuoside I; Icariside II		-1.112
Flavonoid carbonoside			
pmp000236(C26H28O14)	Isovitexin-8-O-xylcoside	-1.298	
mws1073(C27H30O15)	Apigenin-6,8-di-C-glucoside (Vicenin-2)	1.431	
Flavanols			
Cmsp007228(C16H14O6)	7-O-Methyleriodictyol	-1.105	
mws0355(C22H18O10)	Catechin gallate		-2.527
mws1397(C22H18O10)	Epicatechin gallate	11.304	
pmn001527(C22H32O12)	Di-O-galloyl Methyl gallate	-11.000	
Isoflavones			
Lmgp004829(C23H24O12)	5,7,4'-Trihydroxy-6,8-dimethoxyisoflavone-7-O-galactoside	-7.808	
Lignans			
HX1369(C20H24O6)	(-)-Lariciresinol	-1.860	

HX1371(C20H24O6)	(7S,8R)-dihydrodehydrodiconferyl alcohol	-1.846	
Qmzp101909(C21H22O7)	Magnolone	8.782	7.649
HX1288(C19H30O8)	Citroside A	1.004	-1.148
Hmcn002743(C22H26O8)	Lirioresinol A	2.872	
MWS20152(C22H26O8)	syringaresinol	2.951	
mws2162(C24H32O7)	Schisandrol A	4.483	1.580
Ymjm000135(C25H32O11)	8R-Dihydrodehydrodiconferyl alcohol 4-O-β-D-glucopyranoside	-1.632	-2.100
Lmtn003096(C26H36O11)	Secoisolariciresinol 4-O-glucoside	12.351	11.306
Lmnn102709(C26H34O12)	(-)-olivil 4'-O-bate-D-glucopyranoside	-1.734	
Hmln002355(C27H34O12)	5'-Methoxymatairesinoside	-1.315	
Lmmn002260(C27H36O12)	5'-Methoxyisolariciresinol-9'-O-glucoside	-2.089	
Lmqn001932(C28H36O13)	syringaresinol-4'-O-glucoside	1.348	
Zmhn001446(C28H36O13)	syringaresinol-4'-O-glucopyranosid	2.614	1.231
Rfmb26201(C30H38O14)	syringaresinol-4'-O-(6''-acetyl)glucoside	2.929	
Coumarins			
MWSmce439(C9H8O2)	3,4-Dihydrocoumarin	1.392	
mws0987(C10H8O2)	6-MethylCoumarin	-1.239	
mws0139(C10H8O3)	7-Hydroxy-4-methylcoumarin	2.016	
mws1074(C9H6O4)	Daphnetin	1.040	
Lhqp101815(C11H6O3)	Bakuchicin	-2.314	
MWSmce324(C11H6O3)	Psoralen	1.292	
HX1363(C10H8O4)	isoscopoletin	1.389	
Hmcp002123(C10H8O4)	Isoscopoletin (6-Hydroxy-7-Methoxycoumarin)	1.681	
MWSCX014(C10H8O4)	Scopoletin (7-Hydroxy-6-methoxycoumarin)	1.492	
MWSmce082(C11H10O4)	Scoparone		
pmp000284(C11H10O5)	Fraxidin (8-Hydroxy-6,7-dimethoxycoumarin)	3.190	
mws1639(C11H10O5)	Isofraxidin	2.958	
MWSmce160(C14H14O3)	Demethylsuberosin	-1.336	
Lmjp101203(C15H14O4)	phebalosin	-1.524	
HX1386(C15H14O4)	braylin		-1.119
Zmnn010135(C15H16O4)	Sibiricol	1.344	
Cmpp005475(C15H16O4)	Meranzin	4.816	
Cmpp007452(C15H16O4)	Auraptenol	1.239	
Lmwp102713(C14H16O5)	(S)-Peucedanol	2.231	
pmf0526(C16H14O4)	Isoimperatorin	-17.045	
Lmjp101205(C16H18O4)	8-Isopentenyllimettin		-1.055
Lmjp101207(C15H16O5)	(S)-Murpanidin	1.280	
MWSmce224(C19H22O3)	Auraptene	-4.378	-5.790
pmp000022(C16H16O6)	Oxypeucedanin hydrate		-1.622
Qmzp101840(C19H22O4)	Epoxyaurapten	1.572	
mws1015(C15H16O9)	Esculin (6,7-DihydroxyCoumarin-6-glucoside)	-2.613	
Cmyn001328(C15H16O9)	Daphnin	-2.873	
Lmbn001162(C15H16O9)	Esculetin-7-O-glucoside	-1.306	
Lmbp003208(C17H20O10)	Fraxidin-8-O-glucoside	1.622	3.006
Lmjp003090(C17H20O10)	Isofraxidin-7-O-glucoside	2.756	1.612
Cmpp004617(C26H32O8)	Deacetylnomilin		1.920
Cmpp004946(C26H32O8)	Isoobacunoic acid		1.825
Cmpp004143(C26H34O9)	Deacetylnomilinic acid		2.028
Others			
Lmjp004702(C9H10O4)	2',4'-Dihydroxy-6'-methoxyacetophenone	6.321	
Zmdn004863(C13H12O4)	Aloesone	5.013	1.807
Zjbn102516(C15H16O3)	3,3'-Dihydroxy-5-methoxybibenzyl	2.076	

pmp000292(C15H16O5)	Hamaudol	2.239	1.186
Lmcp005089(C18H16O5)	6,8-Dihydroxy-2-[2-(4-methoxyphenyl)ethyl]chromone	-2.621	
Zmxn110201(C14H20O9)	2,6-Dimethoxy-4-hydroxyphenol-1-O-ß-D-glucopyranoside	-1.651	
Lhhp102901(C20H24O6)	3-(α,4-Dihydroxy-3-methoxybenzyl)-4-(hydroxy-3-methoxybe nzyl)	-1.649	
pmp000309(C22H28O11)	Cimifugin-7-O-glucoside	-1.562	
Cmsp003083(C26H32O11)	Dehydrodiconiferyl alcohol-4-O-glucoside	-2.629	
**Phenolic acids**			
Phenolic acids			
mws0628(C7H6O2)	4-Hydroxybenzaldehyde	1.266	
NK10264324(C6H6O3)	1,3,5-Benzenetriol	1.064	
MWS1848(C8H8O2)	Phenyl acetate	1.387	
MWSmce466(C8H8O2)	4-Hydroxyacetophenone	1.596	
mws1336(C7H7NO2)	4-Aminobenzoic Acid	-1.452	
Hmgn001653(C7H6O3)	Protocatechualdehyde	1.981	
Lmgn001670(C7H6O3)	Salicylic acid	1.882	
mws0749(C7H6O3)	4-Hydroxybenzoic Acid	1.881	
mws1024(C9H8O2)	p-Coumaraldehyde	1.213	
mws0458(C8H8O3)	Vanillin	1.355	
mws0180(C7H6O4)	2,5-Dihydroxybenzoic Acid; Gentisic Acid	3.594	
mws0183(C7H6O4)	3,4-Dihydroxybenzoic Acid (Protocatechuic acid)	4.283	
mws0639(C7H6O4)	2,3-Dihydroxybenzoic Acid	4.159	
Lmqp008175(C10H10O2)	Methyl cinnamate	-2.139	
Lmmn001643(C9H8O3)	2-Hydroxycinnamic acid	1.044	
mws1686(C9H10O3)	Paeonol	1.063	1.018
mws0467(C9H10O3)	3-(4-Hydroxyphenyl)-propionic acid		-1.050
MWSslk084(C10H14O2)	4-Butylresorcinol	1.530	1.447
NK10253223(C8H9NO3)	2-Amino-3-methoxybenzoic Acid	-2.986	
pme2598(C8H8O4)	3,4-Dihydroxybenzeneacetic acid		1.680
MWSmce501(C8H8O4)	Methyl 3,4-dihydroxybenzoate	10.497	
MWSmce490(C8H8O4)	Phloracetophenone	4.914	
mws1195(C10H10O3)	p-Coumaric acid methyl ester		-1.611
mws1200(C10H10O3)	Trans-4-Hydroxycinnamic acid Methyl Ester		
pmb2795(C10H10O3)	4-Methoxycinnamic acid		-1.018
mws2212(C9H8O4)	Caffeic acid	1.147	
mws0093(C10H12O3)	coniferyl alcohol	1.076	
mws1350(C9H10O4)	Syringaldehyde; 4-Hydroxy-3,5-Dimethoxybenzaldehyde	2.186	
mws0014(C10H10O4)	Ferulic acid	1.230	
pme0422(C10H10O4)	Isoferulic Acid	1.150	
MWSmce632(C10H10O4)	Vanillin acetate		-1.724
MWSmce364(C10H12O4)	Xanthoxylin	1.770	
mws0027(C9H10O5)	syringic acid	1.684	
MA10107783(C10H8O5)	3-[(1-Carboxyvinyl)oxy]benzoic Acid	1.618	
pme3443(C11H12O4)	Sinapinaldehyde	2.112	
Lmbn004790(C11H14O4)	Methyl 3-(3-hydroxy-4-methoxyphenyl)propanoate	3.237	
mws0853(C11H14O4)	Sinapyl alcohol	2.748	
MWSmce177(C12H14O4)	Ethyl ferulate	-1.514	-1.102
mws4085(C11H12O5)	Sinapic acid	1.084	
Cmsp002787(C11H14O5)	3,4'-Dihydroxy-3',5'-dimethoxypropiophenone	-1.383	
Lmlp002765(C15H16O2)	Stilbostemin B		1.157
pme0428(C12H14O5)	3,4,5-Trimethoxycinnamic acid	1.333	
Lmzn001925(C12H16O5)	3,4'-Dihydroxy-3'-methoxybenzenepentanoic acid		1.491
Lmsp102510(C14H14O4)	Torachrysone		1.548
Lmhn003373(C11H10O7)	p-Hydroxybenzoylmalic acid		1.422

mws1297(C13H18O6)	Benzyl glucoside	2.436	1.298
pme3472(C12H16O7)	Arbutin	-1.328	
Ymjm000152(C12H16O7)	p-Hydroxypheny-β-D-allopyranoside	-1.439	
Hmqp002567(C17H17NO3)	P-Hydroxycinnamic acid p-hydroxyphenethylamine	6.729	3.231
Zmhn001926(C13H16O8)	1-O-Salicyl-D-glucose	4.360	
Zmhn001358(C13H16O8)	4-O-Glucosyl-4-hydroxybenzoic Acid	15.476	
pmb3142(C13H16O8)	Salicylic acid-2-O-glucoside	-1.101	
Hmtn001302(C13H16O8)	Glucosyloxybenzoic Acid	15.339	
Zmhn000892(C13H18O8)	4-O-Glucosyl-3,4-dihydroxybenzyl alcohol	-1.332	
pmb2871(C13H16O9)	1-O-Gentisoyl-D-glucoside	2.159	
pmn001367(C13H16O9)	Protocatechuic acid-4-O-glucoside	2.044	
Zmtn001661(C14H20O8)	Vanilloloside	2.391	
Cmqn010367(C21H32O2)	3-(8Z,11E)-8,11-Pentadecadienyl-1,2-benzenediol	6.347	2.817
pmb0751(C16H16O7)	Trans-5-O-(p-Coumaroyl)shikimate	-1.645	
pmb3075(C16H16O7)	3-O-p-Coumaroylshikimic acid	-1.638	1.109
Hmhn003067(C15H18O8)	Phenylpropionic acid-O-β-D-glucopyranoside	-1.248	
Zmzn001549(C15H18O8)	4-O-beta-D-glucosyl-4-coumaric acid	-1.377	
Zmhn002301(C15H18O8)	p-Coumaric acid-4-O-glucoside	-1.029	
pmn001690(C16H24O7)	3-Hydroxy-4-isopropylbenzylalcohol-3-O-glucoside	-1.575	1.443
Lmtn002565(C14H18O9)	1-O-Vanilloyl-D-Glucose	2.950	
Lmmn003663(C14H18O9)	5-Glucosyloxy-2-Hydroxybenzoic Acid methyl ester	2.612	
Hmln002806(C16H16O8)	5-O-Caffeoylshikimic acid	1.260	2.066
Wmqn000813(C15H16O9)	Cichoriin	-2.569	
pmn001420(C15H18O9)	1-O-[(E)-Caffeoyl]-D-glucose		1.750
Zmhn001793(C15H18O9)	6-O-Caffeoyl-D-glucose	-1.018	1.689
mws0906(C16H22O8)	coniferyl alcohol-4-O-glucoside (Coniferin)		1.652
Zmln000899(C15H20O9)	Syringaldehyde-4-O-glucoside	1.033	
Lmmn000774(C15H20O9)	Dihydrocaffeoylglucose	1.227	1.267
MWSmce270(C20H26O5)	Rosmanol	1.673	
Lmsn003318(C16H20O9)	1-O-Feruloyl-β-D-glucose	1.313	1.293
Hmmn002544(C16H20O9)	Ferulic acid-4-O-glucoside	-1.507	
Ymjm000141(C17H20O9)	4-O-Caffeoylquinic acid methyl ester	1.877	
Ymjm000142(C17H20O9)	5-O-Caffeoylquinic acid methyl ester	1.786	
Lmtn003598(C18H24O8)	3-Prenyl-4-O-glucosyloxy-4-hydroxybenzoic acid	1.406	
pmn001695(C16H20O10)	Trihydroxycinnamoylquinic acid	2.022	
pmb2940(C17H22O10)	1-O-Glucosyl sinapate	2.129	1.914
Zmhn002227(C17H22O10)	Sinapoyl-4-O-glucoside	-1.411	1.276
HJN003(C17H22O10)	1-O-Sinapoyl-D-glucose	-1.239	1.036
Hmln003529(C18H26O10)	Benzyl β-primeveroside	11.486	
Cmjn004337(C18H26O10)	Benzyl-(2''-O-xylosyl)glucoside	3.967	
Cmyp005062(C20H22O9)	Salireposide		-1.585
Lmtn002324(C19H28O11)	Benzyl-(2''-O-glucosyl)glucoside	3.834	1.739
Lmyn003028(C19H28O11)	Benzyl-β-gentiobioside	4.411	1.395
Zmhn002508(C21H28O12)	4-p-Cumaroyl-rhamnosyl-(1→6)-D-glucose		1.298
Ymjm000144(C21H30O13)	2,3,4,5-Trihydroxybutyl 6-O-(E)-caffeoyl-β-D-glucopyranoside	15.211	11.850
pmn001458(C22H30O14)	Sibiricose A5	1.373	
Hmbn002692(C22H30O14)	6'-O-Feruloyl-D-sucrose	1.430	
Lmsp003655(C26H32O11)	Dehydrodiconiferyl alcohol-gamma'-O-glucoside	-2.861	
Lmsn003628(C23H32O15)	6′-O-Sinapoylsucrose	1.620	
Zmhn002032(C28H34O15)	4-O-(6'-O-Glucosylcaffeoylglucosyl)-4-hydroxybenzyl alcohol	2.383	
mws1150(C35H46O20)	Echinacoside		1.474
Total	Upregulated metabolites	104	40
	Downregulated metabolites	52	16
