# Supplementary material for: Mechanisms by Which Increased pH Ameliorates Copper Excess in Citrus sinensis Roots: Insight from a Combined Analysis of Physiology, Transcriptome, and Metabolome
Source: Plants (Basel). 2024 Oct 31;13(21):3054. doi: 10.3390/plants13213054 (PMC11548300; doi:10.3390/plants13213054)
Supplement: Supplementary file 1 [file plants-13-03054-s001.zip › Table S22SecondRevision.pdf]

Table S22. DEGs and DAMs related to ROS detoxification and cell redox homeostasis in P3CR vs. P3R and/or P5CR vs. P5R

**Accession No.**	**KEGG**	**Swissprot (KOG)**	**Log_2(FC)**	
			P3CR vs. P3R	P5CR vs. P5R
Glutathione metabolism [ko00480; P = 0.2222 (P3CR vs. P3R) and 0.0286 (P5CR vs. P5R)]				
Cs1g08610	K00799 glutathione S-transferase [EC:2.5.1.18] | (RefSeq) hypothetical protein (A)	Probable glutathione S-transferase parA; EC=2.5.1.18; Auxin-regulated protein parA; STR246C protein (At1g17180)	2.606	
Cs1g25730	K00434 L-ascorbate peroxidase [EC:1.11.1.11] | (RefSeq) hypothetical protein (A)	L-ascorbate peroxidase 3; AtAPx03; EC=1.11.1.11 (At4g35000)		-1.050
Cs3g17910	K00434 L-ascorbate peroxidase [EC:1.11.1.11] | (RefSeq) thylakoid lumenal 29 kDa protein, chloroplastic (A)	Thylakoid lumenal 29 kDa protein, chloroplastic; TL29; EC=1.-.-.-; LeAPx09; P29; Flags: Precursor (At4g09010)		-1.085
Cs4g13090	K18592 gamma-glutamyltranspeptidase / glutathione hydrolase / leukotriene-C4 hydrolase [EC:2.3.2.2 3.4.19.13 3.4.19.14] | (RefSeq) gamma-glutamyltranspeptidase 3-like (A)	Glutathione hydrolase 3; EC=3.4.19.13; Gamma-glutamyltransferase 3; Gamma-glutamyltranspeptidase 3; EC=2.3.2.2; Gamma-glutamyltranspeptidase 4 (At4g29210)		1.227
Cs5g03810	K00799 glutathione S-transferase [EC:2.5.1.18] | (RefSeq) hypothetical protein (A)	Glutathione S-transferase T1; AtGSTT1; EC=2.5.1.18; GST class-theta member 1; Glutathione S-transferase 10 (At5g41210)		1.212
Cs5g03830	K00432 glutathione peroxidase [EC:1.11.1.9] | (RefSeq) CIT-SAP, PHGPx, csa, gpx1; probable phospholipid hydroperoxide glutathione peroxidase (A)	Probable phospholipid hydroperoxide glutathione peroxidase; PHGPx; EC=1.11.1.12; Salt-associated protein (At4g11600)		-1.179
Cs5g03900	K00799 glutathione S-transferase [EC:2.5.1.18] | (RefSeq) glutathione S-transferase U10-like (A)	Glutathione S-transferase U10; AtGSTU10; EC=2.5.1.18; GST class-tau member 10 (At1g74590)	1.140	-1.094
Cs5g14810	K10807 ribonucleoside-diphosphate reductase subunit M1 [EC:1.17.4.1] | (RefSeq) ribonucleoside-diphosphate reductase large subunit (A)		-1.375	
Cs5g25270	K18592 gamma-glutamyltranspeptidase / glutathione hydrolase / leukotriene-C4 hydrolase [EC:2.3.2.2 3.4.19.13 3.4.19.14] | (RefSeq) glutathione hydrolase 3-like (A)	S-norcoclaurine synthase 1 {ECO:0000303|PubMed:15925393}; PsNCS1 {ECO:0000303|PubMed:15925393}; EC=4.2.1.78 {ECO:0000269|PubMed:15925393}; Flags: Precursor	1.771	
Cs5g32780	K00799 glutathione S-transferase [EC:2.5.1.18] | (RefSeq) glutathione S-transferase F13 (A)	Glutathione S-transferase F13; AtGSTF13; EC=2.5.1.18; GST class-phi member 13 (At3g62760)	-1.125	
Cs5g34430	K00799 glutathione S-transferase [EC:2.5.1.18] | (RefSeq) hypothetical protein (A)	Probable glutathione S-transferase; EC=2.5.1.18; Auxin-induced protein PGNT1/PCNT110 (At2g29420)	1.378	1.305
Cs6g03820	K00799 glutathione S-transferase [EC:2.5.1.18] | (RefSeq)	Glutathione S-transferase L3; AtGSTL3; EC=2.5.1.18; GST	1.213	

	glutathione S-transferase L3-like (A)	class-lambda member 3 (At5g02790)		
Cs6g03830	K00799 glutathione S-transferase [EC:2.5.1.18] | (RefSeq) glutathione S-transferase L3-like (A)	Glutathione S-transferase L3; AtGSTL3; EC=2.5.1.18; GST class-lambda member 3 (At5g02790)		1.372
Cs6g03850	K00799 glutathione S-transferase [EC:2.5.1.18] | (RefSeq) glutathione S-transferase L3-like (A)	Glutathione S-transferase L3; AtGSTL3; EC=2.5.1.18; GST class-lambda member 3 (At5g02790)		1.249
Cs6g04140	K00434 L-ascorbate peroxidase [EC:1.11.1.11] | (RefSeq) hypothetical protein (A)	L-ascorbate peroxidase 2, cytosolic; EC=1.11.1.11; L-ascorbate peroxidase 1b; APX1b; AtAPx02 (At3g09640)	1.147	
Cs6g07240	K00799 glutathione S-transferase [EC:2.5.1.18] | (RefSeq) glutathione S-transferase U8-like (A)	Probable glutathione S-transferase; EC=2.5.1.18; G2-4; Heat shock protein 26A (At3g09270)	1.642	1.194
Cs7g04580	K00799 glutathione S-transferase [EC:2.5.1.18] | (RefSeq) glutathione S-transferase U17-like (A)	Glutathione S-transferase U17; AtGSTU17; EC=2.5.1.18; GST class-tau member 17; Glutathione S-transferase 30; Protein EARLY RESPONSIVE TO DEHYDRATION 9 (At1g10360)		-1.751
Cs7g04600	K00799 glutathione S-transferase [EC:2.5.1.18] | (RefSeq) glutathione S-transferase U17-like (A)	Glutathione S-transferase U17; AtGSTU17; EC=2.5.1.18; GST class-tau member 17; Glutathione S-transferase 30; Protein EARLY RESPONSIVE TO DEHYDRATION 9 (At1g10360)		-1.385
Cs7g05430	K00434 L-ascorbate peroxidase [EC:1.11.1.11] | (RefSeq) putative L-ascorbate peroxidase 6 (A)	Putative L-ascorbate peroxidase 6; AtAPx08; EC=1.11.1.11 (At4g32320)		1.117
Cs7g14120	K00799 glutathione S-transferase [EC:2.5.1.18] | (RefSeq) probable glutathione S-transferase (A)	Probable glutathione S-transferase parC; EC=2.5.1.18; Auxin-regulated protein parC (At1g78370)		-4.257
Cs7g15760	K00799 glutathione S-transferase [EC:2.5.1.18] | (RefSeq) probable glutathione S-transferase (A)	Probable glutathione S-transferase; EC=2.5.1.18; Auxin-induced protein PGNT35/PCNT111 (At2g29420)		-3.338
Cs7g18550	K00799 glutathione S-transferase [EC:2.5.1.18] | (RefSeq) glutathione S-transferase F9-like (A)	Glutathione S-transferase F9; AtGSTF9; EC=2.5.1.18; AtGSTF7; GST class-phi member 9 (At2g30860)	1.427	3.342
Cs8g19390	K00799 glutathione S-transferase [EC:2.5.1.18] | (RefSeq) hypothetical protein (A)	Glutathione S-transferase U7; AtGSTU7; EC=2.5.1.18; GST class-tau member 7; Glutathione S-transferase 25 (At2g29420)		1.371
Cs9g10430	K00799 glutathione S-transferase [EC:2.5.1.18] | (RefSeq) glutathione transferase GST 23-like (A)	Probable glutathione S-transferase; EC=2.5.1.18; G2-4; Heat shock protein 26A (At3g09270)	4.021	
orange1.1t03452	K00799 glutathione S-transferase [EC:2.5.1.18] | (RefSeq) glutathione transferase GST 23-like (A)	Glutathione transferase GST 23; EC=2.5.1.18; Glutathione transferase GST 36 (At3g09270)		-1.047
orange1.1t03456	K00799 glutathione S-transferase [EC:2.5.1.18] | (RefSeq) probable glutathione S-transferase (A)	Probable glutathione S-transferase; EC=2.5.1.18; G2-4; Heat shock protein 26A (At2g29420)	3.694	
orange1.1t03535	K01581 ornithine decarboxylase [EC:4.1.1.17] | (RefSeq) hypothetical protein (A)	Ornithine decarboxylase; ODC; EC=4.1.1.17 (YKL184w)		-1.815

orange1.1t03605	K00799 glutathione S-transferase [EC:2.5.1.18] | (RefSeq) probable glutathione S-transferase (A)	Glutathione transferase GST 23; EC=2.5.1.18; Glutathione transferase GST 36 (At2g29420)		2.427
orange1.1t03618	K00799 glutathione S-transferase [EC:2.5.1.18] | (RefSeq) probable glutathione S-transferase (A)	Probable glutathione S-transferase; EC=2.5.1.18; G2-4; Heat shock protein 26A (At2g29420)	1.186	
orange1.1t03624	K00799 glutathione S-transferase [EC:2.5.1.18] | (RefSeq) probable glutathione S-transferase (A)	Probable glutathione S-transferase; EC=2.5.1.18; G2-4; Heat shock protein 26A (At2g29420)		-1.245
orange1.1t03628	K00799 glutathione S-transferase [EC:2.5.1.18] | (RefSeq) probable glutathione S-transferase (A)	Glutathione transferase GST 23; EC=2.5.1.18; Glutathione transferase GST 36 (At3g09270)		-1.183
orange1.1t03632	K00799 glutathione S-transferase [EC:2.5.1.18] | (RefSeq) probable glutathione S-transferase (A)	Glutathione transferase GST 23; EC=2.5.1.18; Glutathione transferase GST 36 (At2g29420)		-1.088
orange1.1t04722	K00799 glutathione S-transferase [EC:2.5.1.18] | (RefSeq) hypothetical protein (A)	Glutathione S-transferase U19; AtGSTU19; EC=2.5.1.18; GST class-tau member 19; Glutathione S-transferase 8 (At1g17180)		-7.884
orange1.1t04916	K00799 glutathione S-transferase [EC:2.5.1.18] | (RefSeq) probable glutathione S-transferase parC (A)	Probable glutathione S-transferase parC; EC=2.5.1.18; Auxin-regulated protein parC (At1g17180)		-6.674
Glutathione transferase activity [GO:0004364; P = 0.0140 (P3CR vs. P3R) and 0.0005 (P5CR vs. P5R)]				
Cs1g08610	K00799 glutathione S-transferase [EC:2.5.1.18] | (RefSeq) hypothetical protein (A)	Probable glutathione S-transferase parA; EC=2.5.1.18; Auxin-regulated protein parA; STR246C protein (At1g17180)	2.606	
Cs2g30630	K00943 dTMP kinase [EC:2.7.4.9] | (RefSeq) glutathione S-transferase T3-like (A)	Glutathione S-transferase T3; AtGSTT3; EC=2.5.1.18; GST class-theta member 3; Glutathione S-transferase 10C (At2g22710)	-1.904	-1.560
Cs3g01240	K01800 maleylacetoacetate isomerase [EC:5.2.1.2] | (RefSeq) glutathione S-transferase zeta class-like (A)	Glutathione S-transferase zeta class; EC=2.5.1.18 (At2g02390)	1.749	
Cs3g20220	K22077 ganglioside-induced differentiation-associated protein 1 | (RefSeq) glutathione S-transferase TCHQD (A)	Glutathione S-transferase TCHQD; EC=2.5.1.18; Protein tetrachlorohydroquinone dehalogenase-homolog (At1g77290)		1.056
Cs5g03810	K00799 glutathione S-transferase [EC:2.5.1.18] | (RefSeq) hypothetical protein (A)	Glutathione S-transferase T1; AtGSTT1; EC=2.5.1.18; GST class-theta member 1; Glutathione S-transferase 10 (At5g41210)		1.212
Cs5g03900	K00799 glutathione S-transferase [EC:2.5.1.18] | (RefSeq) glutathione S-transferase U10-like (A)	Glutathione S-transferase U10; AtGSTU10; EC=2.5.1.18; GST class-tau member 10 (At1g74590)	1.140	-1.094
Cs5g32780	K00799 glutathione S-transferase [EC:2.5.1.18] | (RefSeq) glutathione S-transferase F13 (A)	Glutathione S-transferase F13; AtGSTF13; EC=2.5.1.18; GST class-phi member 13 (At3g62760)	-1.125	
Cs5g34430	K00799 glutathione S-transferase [EC:2.5.1.18] | (RefSeq) hypothetical protein (A)	Probable glutathione S-transferase; EC=2.5.1.18; Auxin-induced protein PGNT1/PCNT110 (At2g29420)	1.378	1.305
Cs6g03820	K00799 glutathione S-transferase [EC:2.5.1.18] | (RefSeq)	Glutathione S-transferase L3; AtGSTL3; EC=2.5.1.18; GST	1.213	

	glutathione S-transferase L3-like (A)	class-lambda member 3 (At5g02790)		
Cs6g03830	K00799 glutathione S-transferase [EC:2.5.1.18] | (RefSeq) glutathione S-transferase L3-like (A)	Glutathione S-transferase L3; AtGSTL3; EC=2.5.1.18; GST class-lambda member 3 (At5g02790)		1.372
Cs6g03850	K00799 glutathione S-transferase [EC:2.5.1.18] | (RefSeq) glutathione S-transferase L3-like (A)	Glutathione S-transferase L3; AtGSTL3; EC=2.5.1.18; GST class-lambda member 3 (At5g02790)		1.249
Cs6g07240	K00799 glutathione S-transferase [EC:2.5.1.18] | (RefSeq) glutathione S-transferase U8-like (A)	Probable glutathione S-transferase; EC=2.5.1.18; G2-4; Heat shock protein 26A (At3g09270)	1.642	1.194
Cs7g04580	K00799 glutathione S-transferase [EC:2.5.1.18] | (RefSeq) glutathione S-transferase U17-like (A)	Glutathione S-transferase U17; AtGSTU17; EC=2.5.1.18; GST class-tau member 17; Glutathione S-transferase 30; Protein EARLY RESPONSIVE TO DEHYDRATION 9 (At1g10360)		-1.751
Cs7g04600	K00799 glutathione S-transferase [EC:2.5.1.18] | (RefSeq) glutathione S-transferase U17-like (A)	Glutathione S-transferase U17; AtGSTU17; EC=2.5.1.18; GST class-tau member 17; Glutathione S-transferase 30; Protein EARLY RESPONSIVE TO DEHYDRATION 9 (At1g10360)		-1.385
Cs7g14120	K00799 glutathione S-transferase [EC:2.5.1.18] | (RefSeq) probable glutathione S-transferase (A)	Probable glutathione S-transferase parC; EC=2.5.1.18; Auxin-regulated protein parC (At1g78370)		-4.257
Cs7g15760	K00799 glutathione S-transferase [EC:2.5.1.18] | (RefSeq) probable glutathione S-transferase (A)	Probable glutathione S-transferase; EC=2.5.1.18; Auxin-induced protein PGNT35/PCNT111 (At2g29420)		-3.338
Cs7g18550	K00799 glutathione S-transferase [EC:2.5.1.18] | (RefSeq) glutathione S-transferase F9-like (A)	Glutathione S-transferase F9; AtGSTF9; EC=2.5.1.18; AtGSTF7; GST class-phi member 9 (At2g30860)	1.427	3.342
Cs8g19390	K00799 glutathione S-transferase [EC:2.5.1.18] | (RefSeq) hypothetical protein (A)	Glutathione S-transferase U7; AtGSTU7; EC=2.5.1.18; GST class-tau member 7; Glutathione S-transferase 25 (At2g29420)		1.371
Cs9g10430	K00799 glutathione S-transferase [EC:2.5.1.18] | (RefSeq) glutathione transferase GST 23-like (A)	Probable glutathione S-transferase; EC=2.5.1.18; G2-4; Heat shock protein 26A (At3g09270)	4.021	
orange1.1t03452	K00799 glutathione S-transferase [EC:2.5.1.18] | (RefSeq) glutathione transferase GST 23-like (A)	Glutathione transferase GST 23; EC=2.5.1.18; Glutathione transferase GST 36 (At3g09270)		-1.047
orange1.1t03456	K00799 glutathione S-transferase [EC:2.5.1.18] | (RefSeq) probable glutathione S-transferase (A)	Probable glutathione S-transferase; EC=2.5.1.18; G2-4; Heat shock protein 26A (At2g29420)	3.694	
orange1.1t03605	K00799 glutathione S-transferase [EC:2.5.1.18] | (RefSeq) probable glutathione S-transferase (A)	Glutathione transferase GST 23; EC=2.5.1.18; Glutathione transferase GST 36 (At2g29420)		2.427
orange1.1t03624	K00799 glutathione S-transferase [EC:2.5.1.18] | (RefSeq) probable glutathione S-transferase (A)	Probable glutathione S-transferase; EC=2.5.1.18; G2-4; Heat shock protein 26A (At2g29420)		-1.245
orange1.1t03628	K00799 glutathione S-transferase [EC:2.5.1.18] | (RefSeq) probable glutathione S-transferase (A)	Glutathione transferase GST 23; EC=2.5.1.18; Glutathione transferase GST 36 (At3g09270)		-1.183

orange1.1t03632	K00799 glutathione S-transferase [EC:2.5.1.18] | (RefSeq) probable glutathione S-transferase (A)	Glutathione transferase GST 23; EC=2.5.1.18; Glutathione transferase GST 36 (At2g29420)		-1.088
orange1.1t04722	K00799 glutathione S-transferase [EC:2.5.1.18] | (RefSeq) hypothetical protein (A)	Glutathione S-transferase U19; AtGSTU19; EC=2.5.1.18; GST class-tau member 19; Glutathione S-transferase 8 (At1g17180)		-7.884
orange1.1t04916	K00799 glutathione S-transferase [EC:2.5.1.18] | (RefSeq) probable glutathione S-transferase parC (A)	Probable glutathione S-transferase parC; EC=2.5.1.18; Auxin-regulated protein parC (At1g17180)		-6.674
Antioxidant activity [GO:0016209; P = 0.0762 (P3CR vs. P3R) and < 0.0001 (P5CR vs. P5R)]				
Cs1g22960	K00430 peroxidase [EC:1.11.1.7] | (RefSeq) peroxidase 10-like (A)	Peroxidase 10; Atperox P10; EC=1.11.1.7; ATP5a; Flags: Precursor (At1g49570)		1.690
Cs1g25730	K00434 L-ascorbate peroxidase [EC:1.11.1.11] | (RefSeq) hypothetical protein (A)	L-ascorbate peroxidase 3; AtAPx03; EC=1.11.1.11 (At4g35000)		-1.050
Cs1g25930	K00430 peroxidase [EC:1.11.1.7] | (RefSeq) peroxidase 64-like (A)	Peroxidase 64; Atperox P64; EC=1.11.1.7; ATP17a; PRXR4; Flags: Precursor (At5g42180)		2.616
Cs2g05220	K00430 peroxidase [EC:1.11.1.7] | (RefSeq) peroxidase 19 (A)	Peroxidase 19; Atperox P19; EC=1.11.1.7; ATP51; Flags: Precursor (At2g34060)		1.187
Cs2g09200	K00430 peroxidase [EC:1.11.1.7] | (RefSeq) cationic peroxidase 1-like (A)	Cationic peroxidase 1; EC=1.11.1.7; PNPC1; Flags: Precursor (At5g05340)	5.690	
Cs2g09210	K00430 peroxidase [EC:1.11.1.7] | (RefSeq) peroxidase P7-like (A)	Peroxidase 52; Atperox P52; EC=1.11.1.7; ATP49; Flags: Precursor (At5g05340)		1.669
Cs2g09310	K00430 peroxidase [EC:1.11.1.7] | (RefSeq) peroxidase 4 (A)	Peroxidase 4 {ECO:0000250|UniProtKB:Q42578}; EC=1.11.1.7; Flags: Precursor (At5g58400)		1.590
Cs2g11200	K12260 sulfiredoxin [EC:1.8.98.2] | (RefSeq) hypothetical protein (A)	Sulfiredoxin, chloroplastic/mitochondrial; AtSRX; EC=1.8.98.2; Flags: Precursor (At1g31170)		1.069
Cs2g12960	K03671 thioredoxin 1 | (RefSeq) hypothetical protein (A)	Thioredoxin-like 3-3; Thioredoxin-like 1 (At3g53220)		-1.334
Cs2g21810	K00430 peroxidase [EC:1.11.1.7] | (RefSeq) hypothetical protein (A)	Peroxidase 27; Atperox P27; EC=1.11.1.7; ATP12a; PRXR7; Flags: Precursor (At3g01190)		1.538
Cs2g25450	K00430 peroxidase [EC:1.11.1.7] | (RefSeq) peroxidase 55-like (A)	Peroxidase 55; Atperox P55; EC=1.11.1.7; ATP20a; Flags: Precursor (At5g14130)	1.229	
Cs2g28670	K10529 alpha-dioxygenase [EC:1.-.-.-] | (RefSeq) alpha-dioxygenase 1-like (A)	Alpha-dioxygenase 1; Alpha DOX1; EC=1.14.99.-; Fatty acid dioxygenase AlphaDOX1; Pathogen-induced oxygenase; Plant alpha dioxygenase 1; Flags: Precursor (At3g01420)	1.027	-2.590
Cs2g28680	K10529 alpha-dioxygenase [EC:1.-.-.-] | (RefSeq)	Alpha-dioxygenase 1; Alpha DOX1; EC=1.14.99.-; Fatty acid		-3.541

	alpha-dioxygenase 1-like (A)	dioxygenase AlphaDOX1; Pathogen-induced oxygenase; Plant alpha dioxygenase 1; Flags: Precursor (At3g01420)		
Cs3g02270	K00430 peroxidase [EC:1.11.1.7] | (RefSeq) lignin-forming anionic peroxidase-like (A)	Lignin-forming anionic peroxidase; EC=1.11.1.7; Flags: Precursor (At1g14550)		1.775
Cs3g02280	K00430 peroxidase [EC:1.11.1.7] | (RefSeq) lignin-forming anionic peroxidase-like (A)	Lignin-forming anionic peroxidase; EC=1.11.1.7; Flags: Precursor (At1g14550)		1.108
Cs3g09460	K08654 proprotein convertase subtilisin/kexin type 5 [EC:3.4.21.-] | (RefSeq) uncharacterized protein LOC104445957 (A)	Probable nucleoredoxin 1-1; OsNrx1-1; EC=1.8.1.8 (At2g42060)		2.064
Cs3g14240	K13447 respiratory burst oxidase [EC:1.6.3.- 1.11.1.-] | (RefSeq) hypothetical protein (A)	Respiratory burst oxidase homolog protein B; EC=1.11.1.-; EC=1.6.3.-; NADPH oxidase RBOHB; StRBOHB (At1g09090)		1.942
Cs3g17910	K00434 L-ascorbate peroxidase [EC:1.11.1.11] | (RefSeq) thylakoid lumenal 29 kDa protein, chloroplastic (A)	Thylakoid lumenal 29 kDa protein, chloroplastic; TL29; EC=1.-.-.-; LeAPx09; P29; Flags: Precursor (At4g09010)		-1.085
Cs3g21730	K00430 peroxidase [EC:1.11.1.7] | (RefSeq) peroxidase 12 (A)	Peroxidase 12; Atperox P12; EC=1.11.1.7; ATP4a; PRXR6; Flags: Precursor (At1g71695)		-1.066
Cs3g26600	K00430 peroxidase [EC:1.11.1.7] | (RefSeq) peroxidase 64 (A)	Peroxidase 64; Atperox P64; EC=1.11.1.7; ATP17a; PRXR4; Flags: Precursor (At5g42180)		1.204
Cs3g26640	K17609 nucleoredoxin [EC:1.8.1.8] | (RefSeq) probable nucleoredoxin 2 (A)	Probable nucleoredoxin 2; OsNrx2; EC=1.8.1.8 (At1g60420)	2.582	
Cs4g03740	K00430 peroxidase [EC:1.11.1.7] | (RefSeq) hypothetical protein (A)	Peroxidase 16; Atperox P16; EC=1.11.1.7; ATP22a; Flags: Precursor (At2g18980)	-1.821	
Cs4g06920	K13447 respiratory burst oxidase [EC:1.6.3.- 1.11.1.-] | (RefSeq) respiratory burst oxidase homolog protein E (A)	Respiratory burst oxidase homolog protein E; EC=1.11.1.-; EC=1.6.3.-; NADPH oxidase RBOHE; AtRBOHE (At1g19230)		2.468
Cs4g12290	K03671 thioredoxin 1 | (RefSeq) ATC; thioredoxin-like protein CXXS1 (A)	Thioredoxin-like protein CXXS1; AtCXXS1; Mono-cysteine thioredoxin 1 (At1g11530)		-1.459
Cs4g17860	K00430 peroxidase [EC:1.11.1.7] | (RefSeq) peroxidase 25 (A)	Peroxidase 25; Atperox P25; EC=1.11.1.7; Flags: Precursor (At2g41480)		-1.000
Cs5g02940	K13447 respiratory burst oxidase [EC:1.6.3.- 1.11.1.-] | (RefSeq) respiratory burst oxidase homolog protein A (A)	Respiratory burst oxidase homolog protein F; EC=1.11.1.-; EC=1.6.3.-; Cytochrome b245 beta chain homolog RbohAp108; NADPH oxidase RBOHF; AtRBOHF (At1g64060)		1.105
Cs5g03810	K00799 glutathione S-transferase [EC:2.5.1.18] | (RefSeq) hypothetical protein (A)	Glutathione S-transferase T1; AtGSTT1; EC=2.5.1.18; GST class-theta member 1; Glutathione S-transferase 10 (At5g41210)		1.212
Cs5g03830	K00432 glutathione peroxidase [EC:1.11.1.9] | (RefSeq) CIT-SAP, PHGPx, csa, gpx1; probable phospholipid hydroperoxide	Probable phospholipid hydroperoxide glutathione peroxidase; PHGPx; EC=1.11.1.12; Salt-associated protein (At4g11600)		-1.179

	glutathione peroxidase (A)			
Cs5g11890	K13447 respiratory burst oxidase [EC:1.6.3.- 1.11.1.-] | (RefSeq) respiratory burst oxidase homolog protein A-like (A)	Respiratory burst oxidase homolog protein A; EC=1.11.1.-; EC=1.6.3.-; NADPH oxidase RBOHA; StRBOHA (At5g47910)	1.226	3.960
Cs5g16520	K08654 proprotein convertase subtilisin/kexin type 5 [EC:3.4.21.-] | (RefSeq) uncharacterized protein LOC104445957 (A)	Probable nucleoredoxin 1; AtNrx1; EC=1.8.1.8 (At2g27660)		1.359
Cs5g23280	K00430 peroxidase [EC:1.11.1.7] | (RefSeq) hypothetical protein (A)	Probable peroxidase 61; Atperox P61; EC=1.11.1.7; Flags: Precursor (At5g24070)	-1.229	
Cs5g32270	K00430 peroxidase [EC:1.11.1.7] | (RefSeq) peroxidase 3 (A)	Peroxidase 3; Atperox P3; EC=1.11.1.7; ATPRC; RCI3A; Rare cold-inducible protein; Flags: Precursor (At1g05260)		-1.201
Cs6g04000	K10878 meiotic recombination protein SPO11 | (RefSeq) DNA topoisomerase 6 subunit A-like (A)	Thioredoxin-like protein AAED1, chloroplastic; AhpC/TSA antioxidant enzyme domain-containing protein 1; Flags: Precursor (At2g37240)	1.571	
Cs6g04140	K00434 L-ascorbate peroxidase [EC:1.11.1.11] | (RefSeq) hypothetical protein (A)	L-ascorbate peroxidase 2, cytosolic; EC=1.11.1.11; L-ascorbate peroxidase 1b; APX1b; AtAPx02 (At3g09640)	1.147	
Cs6g09680	K00430 peroxidase [EC:1.11.1.7] | (RefSeq) cationic peroxidase 1-like (A)	Cationic peroxidase 1; EC=1.11.1.7; PNPC1; Flags: Precursor (At5g05340)		1.111
Cs7g05430	K00434 L-ascorbate peroxidase [EC:1.11.1.11] | (RefSeq) putative L-ascorbate peroxidase 6 (A)	Putative L-ascorbate peroxidase 6; AtAPx08; EC=1.11.1.11 (At4g32320)		1.117
Cs7g07690	K17609 nucleoredoxin [EC:1.8.1.8] | (RefSeq) probable nucleoredoxin 3 (A)	Probable nucleoredoxin 3; AtNrx3; EC=1.8.1.8 (At4g31240)	1.132	
Cs7g12370	K00430 peroxidase [EC:1.11.1.7] | (RefSeq) peroxidase 11 (A)	Peroxidase 11; Atperox P11; EC=1.11.1.7; ATP23a/ATP23b; Flags: Precursor (At1g68850)	1.028	4.241
Cs7g18550	K00799 glutathione S-transferase [EC:2.5.1.18] | (RefSeq) glutathione S-transferase F9-like (A)	Glutathione S-transferase F9; AtGSTF9; EC=2.5.1.18; AtGSTF7; GST class-phi member 9 (At2g30860)	1.427	3.342
Cs7g19250	K04564 superoxide dismutase, Fe-Mn family [EC:1.15.1.1] | (RefSeq) hypothetical protein (A)	Superoxide dismutase [Fe], chloroplastic; EC=1.15.1.1; Flags: Precursor (At5g51100)	-1.176	
Cs7g29170	K03283 heat shock 70kDa protein 1/2/6/8 | (RefSeq) heat shock cognate 70 kDa protein 2-like isoform X1 (A)	Probable nucleoredoxin 1-2; OsNrx1-2; EC=1.8.1.8 (At2g16050)	2.726	2.927
Cs7g29190	K03283 heat shock 70kDa protein 1/2/6/8 | (RefSeq) heat shock cognate 70 kDa protein 2-like isoform X1 (A)	Probable nucleoredoxin 1-2; OsNrx1-2; EC=1.8.1.8 (At2g16050)	4.565	
Cs8g12000	K13447 respiratory burst oxidase [EC:1.6.3.- 1.11.1.-] | (RefSeq) respiratory burst oxidase homolog protein D (A)	Respiratory burst oxidase homolog protein D; EC=1.11.1.-; EC=1.6.3.-; NADPH oxidase RBOHD; AtRBOHD (At5g47910)		1.497
Cs9g05130	K00430 peroxidase [EC:1.11.1.7] | (RefSeq) peroxidase 3-like (A)	Peroxidase 3; Atperox P3; EC=1.11.1.7; ATPRC; RCI3A; Rare		1.261

		cold-inducible protein; Flags: Precursor (At1g05260)		
Cs9g16590	K00430 peroxidase [EC:1.11.1.7] | (RefSeq) peroxidase 66 (A)	Peroxidase 66; Atperox P66; EC=1.11.1.7; ATP27a; Flags: Precursor (At5g51890)	2.440	5.362
novel.431	K10529 alpha-dioxygenase [EC:1.-.-.-] | (RefSeq) alpha-dioxygenase 1-like (A)	DOX1_ARATH Alpha-dioxygenase 1; Alpha DOX1; EC=1.14.99.-; Fatty acid dioxygenase AlphaDOX1; Pathogen-induced oxygenase; Plant alpha dioxygenase 1; Flags: Precursor (At3g01420)		-2.296
novel.477	K00430 peroxidase [EC:1.11.1.7] | (RefSeq) lignin-forming anionic peroxidase-like (A)	PERX_NICSY Lignin-forming anionic peroxidase; EC=1.11.1.7; Flags: Precursor (At1g14550)	-1.786	2.002
orange1.1t02033	K00430 peroxidase [EC:1.11.1.7] | (RefSeq) peroxidase 59-like (A)	Peroxidase N; EC=1.11.1.7; Neutral peroxidase; Flags: Precursor (At5g19890)		2.207
orange1.1t02041	K00430 peroxidase [EC:1.11.1.7] | (RefSeq) peroxidase A2 (A)	Peroxidase C3; EC=1.11.1.7; Flags: Precursor (At5g06720)		-1.203
orange1.1t02045	K00430 peroxidase [EC:1.11.1.7] | (RefSeq) peroxidase 15-like (A)	Peroxidase 15 {ECO:0000303|PubMed:17936696}; Prx15 {ECO:0000303|PubMed:17936696}; EC=1.11.1.7; Anionic peroxidase {ECO:0000303|PubMed:17936696}; Flags: Precursor (At5g06720)	-1.018	
orange1.1t02059	K00430 peroxidase [EC:1.11.1.7] | (RefSeq) peroxidase A2 (A)	Lignin-forming anionic peroxidase; EC=1.11.1.7; TOPA; Flags: Precursor (At5g06720)		1.147
orange1.1t02220	K03671 thioredoxin 1 | (RefSeq) hypothetical protein (A)	Thioredoxin-like 3-1, chloroplastic; Thioredoxin WCRKC-1; Flags: Precursor (At5g06690)	1.286	1.531
orange1.1t02225	K00430 peroxidase [EC:1.11.1.7] | (RefSeq) LOW QUALITY PROTEIN: peroxidase A2-like (A)	Peroxidase 53; Atperox P53; EC=1.11.1.7; ATPA2; Flags: Precursor (At5g06720)		1.571
orange1.1t04722	K00799 glutathione S-transferase [EC:2.5.1.18] | (RefSeq) hypothetical protein (A)	Glutathione S-transferase U19; AtGSTU19; EC=2.5.1.18; GST class-tau member 19; Glutathione S-transferase 8 (At1g17180)		-7.884
orange1.1t05402	K00430 peroxidase [EC:1.11.1.7] | (RefSeq) peroxidase P7-like (A)	Peroxidase 52; Atperox P52; EC=1.11.1.7; ATP49; Flags: Precursor (At5g05340)		1.615
Peroxidase activity [GO:0004601; P = 0.2771 (P3CR vs. P3R) and < 0.0001 (P5CR vs. P5R)]				
Cs1g22960	K00430 peroxidase [EC:1.11.1.7] | (RefSeq) peroxidase 10-like (A)	Peroxidase 10; Atperox P10; EC=1.11.1.7; ATP5a; Flags: Precursor (At1g49570)		1.690
Cs1g25730	K00434 L-ascorbate peroxidase [EC:1.11.1.11] | (RefSeq) hypothetical protein (A)	L-ascorbate peroxidase 3; AtAPx03; EC=1.11.1.11 (At4g35000)		-1.050
Cs1g25930	K00430 peroxidase [EC:1.11.1.7] | (RefSeq) peroxidase 64-like (A)	Peroxidase 64; Atperox P64; EC=1.11.1.7; ATP17a; PRXR4; Flags: Precursor (At5g42180)		2.616

Cs2g05220	K00430 peroxidase [EC:1.11.1.7] | (RefSeq) peroxidase 19 (A)	Peroxidase 19; Atperox P19; EC=1.11.1.7; ATP51; Flags: Precursor (At2g34060)		1.187
Cs2g09200	K00430 peroxidase [EC:1.11.1.7] | (RefSeq) cationic peroxidase 1-like (A)	Cationic peroxidase 1; EC=1.11.1.7; PNPC1; Flags: Precursor (At5g05340)	5.690	
Cs2g09210	K00430 peroxidase [EC:1.11.1.7] | (RefSeq) peroxidase P7-like (A)	Peroxidase 52; Atperox P52; EC=1.11.1.7; ATP49; Flags: Precursor (At5g05340)		1.669
Cs2g09310	K00430 peroxidase [EC:1.11.1.7] | (RefSeq) peroxidase 4 (A)	Peroxidase 4 {ECO:0000250|UniProtKB:Q42578}; EC=1.11.1.7; Flags: Precursor (At5g58400)		1.590
Cs2g21810	K00430 peroxidase [EC:1.11.1.7] | (RefSeq) hypothetical protein (A)	Peroxidase 27; Atperox P27; EC=1.11.1.7; ATP12a; PRXR7; Flags: Precursor (At3g01190)		1.538
Cs2g25450	K00430 peroxidase [EC:1.11.1.7] | (RefSeq) peroxidase 55-like (A)	Peroxidase 55; Atperox P55; EC=1.11.1.7; ATP20a; Flags: Precursor (At5g14130)	1.229	
Cs2g28670	K10529 alpha-dioxygenase [EC:1.-.-.-] | (RefSeq) alpha-dioxygenase 1-like (A)	Alpha-dioxygenase 1; Alpha DOX1; EC=1.14.99.-; Fatty acid dioxygenase AlphaDOX1; Pathogen-induced oxygenase; Plant alpha dioxygenase 1; Flags: Precursor (At3g01420)	1.027	-2.590
Cs2g28680	K10529 alpha-dioxygenase [EC:1.-.-.-] | (RefSeq) alpha-dioxygenase 1-like (A)	Alpha-dioxygenase 1; Alpha DOX1; EC=1.14.99.-; Fatty acid dioxygenase AlphaDOX1; Pathogen-induced oxygenase; Plant alpha dioxygenase 1; Flags: Precursor (At3g01420)		-3.541
Cs3g02270	K00430 peroxidase [EC:1.11.1.7] | (RefSeq) lignin-forming anionic peroxidase-like (A)	Lignin-forming anionic peroxidase; EC=1.11.1.7; Flags: Precursor (At1g14550)		1.775
Cs3g02280	K00430 peroxidase [EC:1.11.1.7] | (RefSeq) lignin-forming anionic peroxidase-like (A)	Lignin-forming anionic peroxidase; EC=1.11.1.7; Flags: Precursor (At1g14550)		1.108
Cs3g14240	K13447 respiratory burst oxidase [EC:1.6.3.- 1.11.1.-] | (RefSeq) hypothetical protein (A)	Respiratory burst oxidase homolog protein B; EC=1.11.1.-; EC=1.6.3.-; NADPH oxidase RBOHB; StRBOHB (At1g09090)		1.942
Cs3g17910	K00434 L-ascorbate peroxidase [EC:1.11.1.11] | (RefSeq) thylakoid lumenal 29 kDa protein, chloroplastic (A)	Thylakoid lumenal 29 kDa protein, chloroplastic; TL29; EC=1.-.-.-; LeAPx09; P29; Flags: Precursor (At4g09010)		-1.085
Cs3g21730	K00430 peroxidase [EC:1.11.1.7] | (RefSeq) peroxidase 12 (A)	Peroxidase 12; Atperox P12; EC=1.11.1.7; ATP4a; PRXR6; Flags: Precursor (At1g71695)		-1.066
Cs3g26600	K00430 peroxidase [EC:1.11.1.7] | (RefSeq) peroxidase 64 (A)	Peroxidase 64; Atperox P64; EC=1.11.1.7; ATP17a; PRXR4; Flags: Precursor (At5g42180)		1.204
Cs4g03740	K00430 peroxidase [EC:1.11.1.7] | (RefSeq) hypothetical protein (A)	Peroxidase 16; Atperox P16; EC=1.11.1.7; ATP22a; Flags: Precursor (At2g18980)	-1.821	
Cs4g06920	K13447 respiratory burst oxidase [EC:1.6.3.- 1.11.1.-] | (RefSeq)	Respiratory burst oxidase homolog protein E; EC=1.11.1.-;		2.468

	respiratory burst oxidase homolog protein E (A)	EC=1.6.3.-; NADPH oxidase RBOHE; AtRBOHE (At1g19230)		
Cs4g17860	K00430 peroxidase [EC:1.11.1.7] | (RefSeq) peroxidase 25 (A)	Peroxidase 25; Atperox P25; EC=1.11.1.7; Flags: Precursor (At2g41480)		-1.000
Cs5g02940	K13447 respiratory burst oxidase [EC:1.6.3.- 1.11.1.-] | (RefSeq) respiratory burst oxidase homolog protein A (A)	Respiratory burst oxidase homolog protein F; EC=1.11.1.-; EC=1.6.3.-; Cytochrome b245 beta chain homolog RbohAp108; NADPH oxidase RBOHF; AtRBOHF (At1g64060)		1.105
Cs5g03810	K00799 glutathione S-transferase [EC:2.5.1.18] | (RefSeq) hypothetical protein (A)	Glutathione S-transferase T1; AtGSTT1; EC=2.5.1.18; GST class-theta member 1; Glutathione S-transferase 10 (At5g41210)		1.212
Cs5g03830	K00432 glutathione peroxidase [EC:1.11.1.9] | (RefSeq) CIT-SAP, PHGPx, csa, gpx1; probable phospholipid hydroperoxide glutathione peroxidase (A)	Probable phospholipid hydroperoxide glutathione peroxidase; PHGPx; EC=1.11.1.12; Salt-associated protein (At4g11600)		-1.179
Cs5g11890	K13447 respiratory burst oxidase [EC:1.6.3.- 1.11.1.-] | (RefSeq) respiratory burst oxidase homolog protein A-like (A)	Respiratory burst oxidase homolog protein A; EC=1.11.1.-; EC=1.6.3.-; NADPH oxidase RBOHA; StRBOHA (At5g47910)	1.226	3.960
Cs5g23280	K00430 peroxidase [EC:1.11.1.7] | (RefSeq) hypothetical protein (A)	Probable peroxidase 61; Atperox P61; EC=1.11.1.7; Flags: Precursor (At5g24070)	-1.229	
Cs5g32270	K00430 peroxidase [EC:1.11.1.7] | (RefSeq) peroxidase 3 (A)	Peroxidase 3; Atperox P3; EC=1.11.1.7; ATPRC; RCI3A; Rare cold-inducible protein; Flags: Precursor (At1g05260)		-1.201
Cs6g04140	K00434 L-ascorbate peroxidase [EC:1.11.1.11] | (RefSeq) hypothetical protein (A)	L-ascorbate peroxidase 2, cytosolic; EC=1.11.1.11; L-ascorbate peroxidase 1b; APX1b; AtAPx02 (At3g09640)	1.147	
Cs6g09680	K00430 peroxidase [EC:1.11.1.7] | (RefSeq) cationic peroxidase 1-like (A)	Cationic peroxidase 1; EC=1.11.1.7; PNPC1; Flags: Precursor (At5g05340)		1.111
Cs7g05430	K00434 L-ascorbate peroxidase [EC:1.11.1.11] | (RefSeq) putative L-ascorbate peroxidase 6 (A)	Putative L-ascorbate peroxidase 6; AtAPx08; EC=1.11.1.11 (At4g32320)		1.117
Cs7g12370	K00430 peroxidase [EC:1.11.1.7] | (RefSeq) peroxidase 11 (A)	Peroxidase 11; Atperox P11; EC=1.11.1.7; ATP23a/ATP23b; Flags: Precursor (At1g68850)	1.028	4.241
Cs7g18550	K00799 glutathione S-transferase [EC:2.5.1.18] | (RefSeq) glutathione S-transferase F9-like (A)	Glutathione S-transferase F9; AtGSTF9; EC=2.5.1.18; AtGSTF7; GST class-phi member 9 (At2g30860)	1.427	3.342
Cs8g12000	K13447 respiratory burst oxidase [EC:1.6.3.- 1.11.1.-] | (RefSeq) respiratory burst oxidase homolog protein D (A)	Respiratory burst oxidase homolog protein D; EC=1.11.1.-; EC=1.6.3.-; NADPH oxidase RBOHD; AtRBOHD (At5g47910)		1.497
Cs9g05130	K00430 peroxidase [EC:1.11.1.7] | (RefSeq) peroxidase 3-like (A)	Peroxidase 3; Atperox P3; EC=1.11.1.7; ATPRC; RCI3A; Rare cold-inducible protein; Flags: Precursor (At1g05260)		1.261
Cs9g16590	K00430 peroxidase [EC:1.11.1.7] | (RefSeq) peroxidase 66 (A)	Peroxidase 66; Atperox P66; EC=1.11.1.7; ATP27a; Flags: Precursor (At5g51890)	2.440	5.362

novel.431	K10529 alpha-dioxygenase [EC:1.-.-.-] | (RefSeq) alpha-dioxygenase 1-like (A)	DOX1_ARATH Alpha-dioxygenase 1; Alpha DOX1; EC=1.14.99.-; Fatty acid dioxygenase AlphaDOX1; Pathogen-induced oxygenase; Plant alpha dioxygenase 1; Flags: Precursor (At3g01420)		-2.296
novel.477	K00430 peroxidase [EC:1.11.1.7] | (RefSeq) lignin-forming anionic peroxidase-like (A)	PERX_NICSY Lignin-forming anionic peroxidase; EC=1.11.1.7; Flags: Precursor (At1g14550)	-1.786	2.002
orange1.1t02033	K00430 peroxidase [EC:1.11.1.7] | (RefSeq) peroxidase 59-like (A)	Peroxidase N; EC=1.11.1.7; Neutral peroxidase; Flags: Precursor (At5g19890)		2.207
orange1.1t02041	K00430 peroxidase [EC:1.11.1.7] | (RefSeq) peroxidase A2 (A)	Peroxidase C3; EC=1.11.1.7; Flags: Precursor (At5g06720)		-1.203
orange1.1t02045	K00430 peroxidase [EC:1.11.1.7] | (RefSeq) peroxidase 15-like (A)	Peroxidase 15 {ECO:0000303|PubMed:17936696}; Prx15 {ECO:0000303|PubMed:17936696}; EC=1.11.1.7; Anionic peroxidase {ECO:0000303|PubMed:17936696}; Flags: Precursor (At5g06720)	-1.018	
orange1.1t02059	K00430 peroxidase [EC:1.11.1.7] | (RefSeq) peroxidase A2 (A)	Lignin-forming anionic peroxidase; EC=1.11.1.7; TOPA; Flags: Precursor (At5g06720)		1.147
orange1.1t02225	K00430 peroxidase [EC:1.11.1.7] | (RefSeq) LOW QUALITY PROTEIN: peroxidase A2-like (A)	Peroxidase 53; Atperox P53; EC=1.11.1.7; ATPA2; Flags: Precursor (At5g06720)		1.571
orange1.1t04722	K00799 glutathione S-transferase [EC:2.5.1.18] | (RefSeq) hypothetical protein (A)	Glutathione S-transferase U19; AtGSTU19; EC=2.5.1.18; GST class-tau member 19; Glutathione S-transferase 8 (At1g17180)		-7.884
orange1.1t05402	K00430 peroxidase [EC:1.11.1.7] | (RefSeq) peroxidase P7-like (A)	Peroxidase 52; Atperox P52; EC=1.11.1.7; ATP49; Flags: Precursor (At5g05340)		1.615
Response to oxidative stress [GO:0006979; P = 0.3087 (P3CR vs. P3R) and 0.0001 (P5CR vs. P5R)]				
Cs1g03720	K22048 mechanosensitive ion channel protein 4/5/6/7/8/9/10 | (RefSeq) mechanosensitive ion channel protein 10-like (A)	Mechanosensitive ion channel protein 10; Mechanosensitive channel of small conductance-like 10; MscS-Like protein 10; AtMSL10 (At5g12080)	1.255	
Cs1g07440		Protein GRIM REAPER {ECO:0000303|PubMed:19279211}; Stigma-specific STIG1-like protein GRI {ECO:0000303|PubMed:19279211}; Contains: GRIp {ECO:0000303|PubMed:25398910}; Flags: Precursor (At1g53130)		1.169
Cs1g09670	K00521 ferric-chelate reductase [EC:1.16.1.7] | (RefSeq) ferric reduction oxidase 7, chloroplastic-like (A)	NAC domain-containing protein 92 {ECO:0000303|PubMed:15029955}; ANAC092 {ECO:0000303|PubMed:15029955}; AtNAC2 {ECO:0000303|PubMed:16359384}; AtNAC6	1.261	1.377

		{ECO:0000303|PubMed:21511905}; Protein ORESARA 1 {ECO:0000303|PubMed:9351240} (At3g18400)		
Cs1g16780	K13993 HSP20 family protein | (RefSeq) 17.4 kDa class III heat shock protein (A)	17.4 kDa class III heat shock protein; 17.4 kDa heat shock protein 2; AtHsp17.4B (At1g54050)	1.083	
Cs1g22960	K00430 peroxidase [EC:1.11.1.7] | (RefSeq) peroxidase 10-like (A)	Peroxidase 10; Atperox P10; EC=1.11.1.7; ATP5a; Flags: Precursor (At1g49570)		1.690
Cs1g25730	K00434 L-ascorbate peroxidase [EC:1.11.1.11] | (RefSeq) hypothetical protein (A)	L-ascorbate peroxidase 3; AtAPx03; EC=1.11.1.11 (At4g35000)		-1.050
Cs1g25930	K00430 peroxidase [EC:1.11.1.7] | (RefSeq) peroxidase 64-like (A)	Peroxidase 64; Atperox P64; EC=1.11.1.7; ATP17a; PRXR4; Flags: Precursor (At5g42180)		2.616
Cs2g04400	K09419 heat shock transcription factor, other eukaryote | (RefSeq) heat stress transcription factor A-4a (A)	Heat stress transcription factor A-4a; AtHsfA4a; AtHsf-15; Heat shock factor protein 21; HSF 21; Heat shock transcription factor 21; HSTF 21 (At4g18880)		1.395
Cs2g05220	K00430 peroxidase [EC:1.11.1.7] | (RefSeq) peroxidase 19 (A)	Peroxidase 19; Atperox P19; EC=1.11.1.7; ATP51; Flags: Precursor (At2g34060)		1.187
Cs2g09200	K00430 peroxidase [EC:1.11.1.7] | (RefSeq) cationic peroxidase 1-like (A)	Cationic peroxidase 1; EC=1.11.1.7; PNPC1; Flags: Precursor (At5g05340)	5.690	
Cs2g09210	K00430 peroxidase [EC:1.11.1.7] | (RefSeq) peroxidase P7-like (A)	Peroxidase 52; Atperox P52; EC=1.11.1.7; ATP49; Flags: Precursor (At5g05340)		1.669
Cs2g09310	K00430 peroxidase [EC:1.11.1.7] | (RefSeq) peroxidase 4 (A)	Peroxidase 4 {ECO:0000250|UniProtKB:Q42578}; EC=1.11.1.7; Flags: Precursor (At5g58400)		1.590
Cs2g11200	K12260 sulfiredoxin [EC:1.8.98.2] | (RefSeq) hypothetical protein (A)	Sulfiredoxin, chloroplastic/mitochondrial; AtSRX; EC=1.8.98.2; Flags: Precursor (At1g31170)		1.069
Cs2g12960	K03671 thioredoxin 1 | (RefSeq) hypothetical protein (A)	Thioredoxin-like 3-3; Thioredoxin-like 1 (At3g53220)		-1.334
Cs2g17100		Protein PLANT CADMIUM RESISTANCE 2; AtPCR2 (At1g14870)	1.426	1.448
Cs2g17170		Protein PLANT CADMIUM RESISTANCE 2; AtPCR2 (At1g14870)	1.529	
Cs2g18250	K13691 pathogen-inducible salicylic acid glucosyltransferase [EC:2.4.1.-] | (RefSeq) UGT3; UDP-glycosyltransferase 74G1 (A)	UDP-glycosyltransferase 74E2; EC=2.4.1.- (At1g05680)		-2.275
Cs2g19230	K22763 deubiquitinase DESI2 [EC:3.4.19.12] | (Kazusa) Lj2g3v1989220.3; - (A)	Nudix hydrolase 2; AtNUDT2; EC=3.6.1.-; ADP-ribose pyrophosphatase; EC=3.6.1.13; NADH pyrophosphatase; EC=3.6.1.22 (At5g47650)		1.015
Cs2g21810	K00430 peroxidase [EC:1.11.1.7] | (RefSeq) hypothetical protein (A)	Peroxidase 27; Atperox P27; EC=1.11.1.7; ATP12a; PRXR7; Flags: Precursor (At3g01190)		1.538

Cs2g23750	K13691 pathogen-inducible salicylic acid glucosyltransferase [EC:2.4.1.-] | (RefSeq) hypothetical protein (A)	UDP-glycosyltransferase 74E2; EC=2.4.1.- (At1g05680)		-2.697
Cs2g25450	K00430 peroxidase [EC:1.11.1.7] | (RefSeq) peroxidase 55-like (A)	Peroxidase 55; Atperox P55; EC=1.11.1.7; ATP20a; Flags: Precursor (At5g14130)	1.229	
Cs2g27610	K06617 raffinose synthase [EC:2.4.1.82] | (RefSeq) probable galactinolsucrose galactosyltransferase 5 (A)	Probable galactinolsucrose galactosyltransferase 5; EC=2.4.1.82; Protein SEED IMBIBITION 1-LIKE; Raffinose synthase 5 (At5g40390)		-1.467
Cs2g28670	K10529 alpha-dioxygenase [EC:1.-.-.-] | (RefSeq) alpha-dioxygenase 1-like (A)	Alpha-dioxygenase 1; Alpha DOX1; EC=1.14.99.-; Fatty acid dioxygenase AlphaDOX1; Pathogen-induced oxygenase; Plant alpha dioxygenase 1; Flags: Precursor (At3g01420)	1.027	-2.590
Cs2g28680	K10529 alpha-dioxygenase [EC:1.-.-.-] | (RefSeq) alpha-dioxygenase 1-like (A)	Alpha-dioxygenase 1; Alpha DOX1; EC=1.14.99.-; Fatty acid dioxygenase AlphaDOX1; Pathogen-induced oxygenase; Plant alpha dioxygenase 1; Flags: Precursor (At3g01420)		-3.541
Cs3g02270	K00430 peroxidase [EC:1.11.1.7] | (RefSeq) lignin-forming anionic peroxidase-like (A)	Lignin-forming anionic peroxidase; EC=1.11.1.7; Flags: Precursor (At1g14550)		1.775
Cs3g02280	K00430 peroxidase [EC:1.11.1.7] | (RefSeq) lignin-forming anionic peroxidase-like (A)	Lignin-forming anionic peroxidase; EC=1.11.1.7; Flags: Precursor (At1g14550)		1.108
Cs3g06080	K10525 allene oxide cyclase [EC:5.3.99.6] | (RefSeq) allene oxide cyclase 4, chloroplastic-like (A)	Allene oxide cyclase, chloroplastic {ECO:0000303|Ref.1, ECO:0000303|Ref.2}; OsAOC {ECO:0000303|Ref.1}; EC=5.3.99.6 {ECO:0000269|PubMed:23347338}; Protein COLEOPTILE PHOTOMORPHOGENESIS 2 {ECO:0000303|PubMed:23347338}; Protein HEBIBA {ECO:0000303|PubMed:14605232}; Flags: Precursor (At1g13280)	-1.814	-1.501
Cs3g17910	K00434 L-ascorbate peroxidase [EC:1.11.1.11] | (RefSeq) thylakoid lumenal 29 kDa protein, chloroplastic (A)	Thylakoid lumenal 29 kDa protein, chloroplastic; TL29; EC=1.-.-.-; LeAPx09; P29; Flags: Precursor (At4g09010)		-1.085
Cs3g18450	K01087 trehalose 6-phosphate phosphatase [EC:3.1.3.12] | (RefSeq) probable trehalose-phosphate phosphatase D (A)	Probable trehalose-phosphate phosphatase D; AtTPPD; EC=3.1.3.12; Trehalose 6-phosphate phosphatase (At1g35910)		1.440
Cs3g21730	K00430 peroxidase [EC:1.11.1.7] | (RefSeq) peroxidase 12 (A)	Peroxidase 12; Atperox P12; EC=1.11.1.7; ATP4a; PRXR6; Flags: Precursor (At1g71695)		-1.066
Cs3g21790	K12124 GIGANTEA | (RefSeq) protein GIGANTEA (A)	Protein GIGANTEA (At1g22770)		2.087
Cs3g26600	K00430 peroxidase [EC:1.11.1.7] | (RefSeq) peroxidase 64 (A)	Peroxidase 64; Atperox P64; EC=1.11.1.7; ATP17a; PRXR4; Flags: Precursor (At5g42180)		1.204
Cs4g01600	K03098 apolipoprotein D and lipocalin family protein | (RefSeq) hypothetical protein (A)	Temperature-induced lipocalin-1 {ECO:0000303|PubMed:18671872}; AtTIL1	1.978	

		{ECO:0000303|PubMed:18671872} (At5g58070)		
Cs4g03740	K00430 peroxidase [EC:1.11.1.7] | (RefSeq) hypothetical protein (A)	Peroxidase 16; Atperox P16; EC=1.11.1.7; ATP22a; Flags: Precursor (At2g18980)	-1.821	
Cs4g05880	K13993 HSP20 family protein | (RefSeq) 17.7 kDa class II heat shock protein (A)	17.7 kDa class II heat shock protein; 17.7 kDa heat shock protein; AtHsp17.7 (At5g12030)		2.404
Cs4g12290	K03671 thioredoxin 1 | (RefSeq) ATC; thioredoxin-like protein CXXS1 (A)	Thioredoxin-like protein CXXS1; AtCXXS1; Mono-cysteine thioredoxin 1 (At1g11530)		-1.459
Cs4g12540	K06892 feruloyl-CoA ortho-hydroxylase [EC:1.14.11.-] | (RefSeq) hypothetical protein (A)	Feruloyl CoA ortho-hydroxylase 1; EC=1.14.11.- (At3g12900)	1.199	2.220
Cs4g13870	K05933 aminocyclopropanecarboxylate oxidase [EC:1.14.17.4] | (RefSeq) hypothetical protein (A)	1-aminocyclopropane-1-carboxylate oxidase 1; ACC oxidase 1; AtACO1; EC=1.14.17.4 (At2g19590)	-1.124	
Cs4g17860	K00430 peroxidase [EC:1.11.1.7] | (RefSeq) peroxidase 25 (A)	Peroxidase 25; Atperox P25; EC=1.11.1.7; Flags: Precursor (At2g41480)		-1.000
Cs4g18140	K03006 DNA-directed RNA polymerase II subunit RPB1 [EC:2.7.7.6] | (RefSeq) early nodulin-like protein 2 (A)	Blue copper protein; Blue copper-binding protein; AtBCB; Phytocyanin 1; Stellacyanin; Flags: Precursor (At5g20230)		-1.225
Cs5g03830	K00432 glutathione peroxidase [EC:1.11.1.9] | (RefSeq) CIT-SAP, PHGPx, csa, gpx1; probable phospholipid hydroperoxide glutathione peroxidase (A)	Probable phospholipid hydroperoxide glutathione peroxidase; PHGPx; EC=1.11.1.12; Salt-associated protein (At4g11600)		-1.179
Cs5g06440	K06617 raffinose synthase [EC:2.4.1.82] | (RefSeq) probable galactinolsucrose galactosyltransferase 2 (A)	Probable galactinolsucrose galactosyltransferase 2; EC=2.4.1.82; Protein SEED IMBIBITION 2; Raffinose synthase 2 (At3g57520)		-2.019
Cs5g22010	K19613 leucine-rich repeat protein SHOC2 | (RefSeq) hypothetical protein (A)	Putative disease resistance protein At4g11170 (At5g17680)		1.491
Cs5g23280	K00430 peroxidase [EC:1.11.1.7] | (RefSeq) hypothetical protein (A)	Probable peroxidase 61; Atperox P61; EC=1.11.1.7; Flags: Precursor (At5g24070)	-1.229	
Cs5g28040	K14963 COMPASS component SWD3 | (RefSeq) transcriptional corepressor LEUNIG_HOMOLOG-like (A)	Transcriptional corepressor LEUNIG_HOMOLOG; Protein MUCILAGE-MODIFIED 1 {ECO:0000303|PubMed:11706181} (At2g32700)		1.712
Cs5g32270	K00430 peroxidase [EC:1.11.1.7] | (RefSeq) peroxidase 3 (A)	Peroxidase 3; Atperox P3; EC=1.11.1.7; ATPRC; RCI3A; Rare cold-inducible protein; Flags: Precursor (At1g05260)		-1.201
Cs6g04140	K00434 L-ascorbate peroxidase [EC:1.11.1.11] | (RefSeq) hypothetical protein (A)	L-ascorbate peroxidase 2, cytosolic; EC=1.11.1.11; L-ascorbate peroxidase 1b; APX1b; AtAPx02 (At3g09640)	1.147	
Cs6g07540	K12349 neutral ceramidase [EC:3.5.1.23] | (RefSeq) neutral ceramidase (A)	Neutral ceramidase 1 {ECO:0000303|PubMed:26150824}; AtNCER1 {ECO:0000303|PubMed:26150824}; N-CDase 1		-2.025

		{ECO:0000303|PubMed:26150824}; NCDase 1 {ECO:0000303|PubMed:26150824}; EC=3.5.1.23 {ECO:0000250|UniProtKB:O06769}; Acylsphingosine deacylase 1; N-acylsphingosine amidohydrolase 1; Flags: Precursor (At1g07380)		
Cs6g09680	K00430 peroxidase [EC:1.11.1.7] | (RefSeq) cationic peroxidase 1-like (A)	Cationic peroxidase 1; EC=1.11.1.7; PNPC1; Flags: Precursor (At5g05340)		1.111
Cs6g15290	K21396 ATP-binding cassette, subfamily G (WHITE), eye pigment precursor transporter | (RefSeq) ABC transporter G family member 7 (A)	ABC transporter G family member 39; ABC transporter ABCG.39; AtABCG39; Pleiotropic drug resistance protein 11 (At2g36380)		1.110
Cs6g16020	K04733 interleukin-1 receptor-associated kinase 4 [EC:2.7.11.1] | (RefSeq) cysteine-rich receptor-like protein kinase 2 (A)	Cysteine-rich receptor-like protein kinase 2; Cysteine-rich RLK2; EC=2.7.11.-; Flags: Precursor (At1g70520)	1.555	
Cs6g18100	K10747 DNA ligase 1 [EC:6.5.1.1 6.5.1.6 6.5.1.7] | (RefSeq) DNA ligase 1 (A)	DNA ligase 6 {ECO:0000303|PubMed:20584150}; AtLIG6 {ECO:0000303|PubMed:20584150}; DNA ligase VI {ECO:0000303|PubMed:25641249}; EC=6.5.1.1 {ECO:0000250|UniProtKB:P56709, ECO:0000255|PROSITE-ProRule:PRU10135}; Ligase 1 {ECO:0000303|PubMed:15155891} (At1g66730_2)		1.031
Cs6g18400	K07304 peptide-methionine (S)-S-oxide reductase [EC:1.8.4.11] | (RefSeq) peptide methionine sulfoxide reductase-like (A)	Peptide methionine sulfoxide reductase A4, chloroplastic; AtMSRA4; EC=1.8.4.11; Peptide-methionine (S)-S-oxide reductase; Peptide Met(O) reductase; Protein-methionine-S-oxide reductase; Flags: Precursor (At4g25130)		-1.259
Cs7g01770	K20456 oxysterol-binding protein 1 | (RefSeq) hypothetical protein (A)	Protein DETOXIFICATION 43 {ECO:0000303|PubMed:11739388}; AtDTX43 {ECO:0000303|PubMed:11739388}; Multidrug and toxic compound extrusion protein 43 {ECO:0000305}; MATE protein 43 {ECO:0000305}; Protein FERRIC REDUCTASE DEFECTIVE 3 {ECO:0000303|PubMed:12172022}; AtFRD3 {ECO:0000303|PubMed:12172022}; Protein MANGANESE ACCUMULATOR 1 {ECO:0000303|PubMed:8754685} (At3g08040)		1.853
Cs7g05430	K00434 L-ascorbate peroxidase [EC:1.11.1.11] | (RefSeq) putative L-ascorbate peroxidase 6 (A)	Putative L-ascorbate peroxidase 6; AtAPx08; EC=1.11.1.11 (At4g32320)		1.117
Cs7g09010	K04733 interleukin-1 receptor-associated kinase 4 [EC:2.7.11.1] | (RefSeq) cysteine-rich receptor-like protein kinase 2 isoform X2 (A)	Cysteine-rich receptor-like protein kinase 2; Cysteine-rich RLK2; EC=2.7.11.-; Flags: Precursor (At1g70520)		1.038

Cs7g12370	K00430 peroxidase [EC:1.11.1.7] | (RefSeq) peroxidase 11 (A)	Peroxidase 11; Atperox P11; EC=1.11.1.7; ATP23a/ATP23b; Flags: Precursor (At1g68850)	1.028	4.241
Cs7g15850	K22048 mechanosensitive ion channel protein 4/5/6/7/8/9/10 | (RefSeq) mechanosensitive ion channel protein 10-like (A)	Mechanosensitive ion channel protein 10; Mechanosensitive channel of small conductance-like 10; MscS-Like protein 10; AtMSL10 (At5g12080)		1.491
Cs7g16460	K09060 plant G-box-binding factor | (RefSeq) bZIP transcription factor 16-like (A)	UV-B-induced protein At3g17800, chloroplastic {ECO:0000305}; Flags: Precursor (At3g07310)		-1.133
Cs7g32260	K13993 HSP20 family protein | (RefSeq) 18.1 kDa class I heat shock protein-like (A)	18.1 kDa class I heat shock protein; 18.1 kDa heat shock protein; AtHsp18.1 (At5g59720)		-4.082
Cs8g03710		Probable zinc metallopeptidase EGY3, chloroplastic; EC=3.4.24.-; Protein ETHYLENE-DEPENDENT GRAVITROPISM-DEFICIENT AND YELLOW-GREEN 3; AtEGY3; Flags: Precursor (At1g17870)	3.514	
Cs8g15600	K18486 heart-and neural crest derivatives-expressed protein 2 | (RefSeq) transcription factor bHLH27-like (A)	Transcription factor FER-LIKE IRON DEFICIENCY-INDUCED TRANSCRIPTION FACTOR; Basic helix-loop-helix protein 29; AtbHLH29; bHLH 29; FER-LIKE REGULATOR OF IRON UPTAKE; Transcription factor EN 43; Transcription factor Fe-DEFICIENCY INDUCED TRANSCRIPTION FACTOR 1; bHLH transcription factor bHLH029 (At2g28160)		1.704
Cs8g17360	K20536 mitogen-activated protein kinase 3 [EC:2.7.11.24] | (RefSeq) MAPK1, MAP; mitogen-activated protein kinase 1 (A)	Mitogen-activated protein kinase 3; AtMPK3; MAP kinase 3; EC=2.7.11.24 (At3g45640)		1.145
Cs8g17960	K23480 zinc finger protein 362/384 | (RefSeq) zinc finger protein ZAT5 (A)	Zinc finger protein ZAT12; Protein RESPONSIVE TO HIGH LIGHT 41 (At2g28710)	1.154	
Cs8g18240	K03283 heat shock 70kDa protein 1/2/6/8 | (RefSeq) probable mediator of RNA polymerase II transcription subunit 37c (A)	Probable mediator of RNA polymerase II transcription subunit 37c; Heat shock 70 kDa protein 4; Heat shock cognate 70 kDa protein 4; Heat shock cognate protein 70-4; AtHsc70-4; Heat shock protein 70-4; AtHsp70-4 (At3g12580)		1.617
Cs9g02590	K17710 pentatricopeptide repeat domain-containing protein 1 | (RefSeq) pentatricopeptide repeat-containing protein At1g63330-like isoform X1 (A)	Putative pentatricopeptide repeat-containing protein At3g16890, mitochondrial; Protein PENTATRICOPEPTIDE REPEAT 40; Flags: Precursor (At3g16890)	1.103	2.696
Cs9g02930	K06892 feruloyl-CoA ortho-hydroxylase [EC:1.14.11.-] | (RefSeq) feruloyl CoA ortho-hydroxylase 1 (A)	Feruloyl CoA ortho-hydroxylase 1; EC=1.14.11.- (At3g13610)	2.493	1.665
Cs9g05130	K00430 peroxidase [EC:1.11.1.7] | (RefSeq) peroxidase 3-like (A)	Peroxidase 3; Atperox P3; EC=1.11.1.7; ATPRC; RCI3A; Rare cold-inducible protein; Flags: Precursor (At1g05260)		1.261
Cs9g09850		Plastid-lipid-associated protein 6, chloroplastic	1.264	

		{ECO:0000303|PubMed:16850419}; Fibrillin-4 {ECO:0000303|PubMed:16461379}; Fibrillin-6 {ECO:0000303|PubMed:18455927}; Harpin-binding protein 1 {ECO:0000303|Ref.1}; HrBP1 {ECO:0000303|Ref.1}; Plastoglobulin 30.4 {ECO:0000303|PubMed:16414959}; AtPGL30.4 {ECO:0000303|PubMed:16414959}; Flags: Precursor (At3g23400)		
Cs9g12460	K06617 raffinose synthase [EC:2.4.1.82] | (RefSeq) probable galactinolsucrose galactosyltransferase 2 (A)	Probable galactinolsucrose galactosyltransferase 2; EC=2.4.1.82; Protein SEED IMBIBITION 2; Raffinose synthase 2 (At3g57520)	1.342	1.225
Cs9g16590	K00430 peroxidase [EC:1.11.1.7] | (RefSeq) peroxidase 66 (A)	Peroxidase 66; Atperox P66; EC=1.11.1.7; ATP27a; Flags: Precursor (At5g51890)	2.440	5.362
novel.1107	K09286 EREBP-like factor | (RefSeq) ethylene-responsive transcription factor CRF2-like (A)	RA211_ARATH Ethylene-responsive transcription factor RAP2-11; Protein RELATED TO APETALA2 11 (At5g19790)		-7.424
novel.431	K10529 alpha-dioxygenase [EC:1.-.-.-] | (RefSeq) alpha-dioxygenase 1-like (A)	DOX1_ARATH Alpha-dioxygenase 1; Alpha DOX1; EC=1.14.99.-; Fatty acid dioxygenase AlphaDOX1; Pathogen-induced oxygenase; Plant alpha dioxygenase 1; Flags: Precursor (At3g01420)		-2.296
novel.477	K00430 peroxidase [EC:1.11.1.7] | (RefSeq) lignin-forming anionic peroxidase-like (A)	PERX_NICSY Lignin-forming anionic peroxidase; EC=1.11.1.7; Flags: Precursor (At1g14550)	-1.786	2.002
orange1.1t00464	K14506 jasmonic acid-amino synthetase | (RefSeq) jasmonic acid-amido synthetase JAR1 (A)	Jasmonic acid-amido synthetase JAR1 {ECO:0000305}; EC=6.3.2.-; Auxin-responsive GH3-like protein 5 {ECO:0000303|PubMed:15856348}; OsGH3-5 {ECO:0000303|PubMed:15856348}; Indole-3-acetic acid-amido synthetase GH3.5 {ECO:0000305}; Jasmonate-amino acid synthetase JAR1 {ECO:0000305}; Protein JASMONATE RESISTANT 1 {ECO:0000303|PubMed:18266905}; OsJAR1 {ECO:0000303|PubMed:18266905} (At2g46370)		-1.108
orange1.1t00550	K13422 transcription factor MYC2 | (RefSeq) transcription factor MYC2-like (A)	Transcription factor MYC2; AtMYC2; Basic helix-loop-helix protein 6; AtbHLH6; bHLH 6; Protein JASMONATE INSENSITIVE 1; R-homologous Arabidopsis protein 1; RAP-1; Transcription factor EN 38; Z-box binding factor 1 protein; bHLH transcription factor bHLH006; rd22BP1 (At1g32640)	-1.178	-1.600
orange1.1t01627	K14724 solute carrier family 9 (sodium/hydrogen exchanger), member 8 | (RefSeq) sodium/hydrogen exchanger 6-like isoform X1 (A)	Sodium/hydrogen exchanger 7; Na(+)/H(+) exchanger 7; NHE-7; Protein SALT OVERLY SENSITIVE 1 (At2g01980)	1.221	1.756
orange1.1t019	K22736 vacuolar iron transporter family protein | (RefSeq)	Vacuolar iron transporter homolog 1; Protein NODULIN-LIKE 1	-1.803	

89	vacuolar iron transporter homolog 2.1 (A)	(At1g76800)		
orange1.1t02033	K00430 peroxidase [EC:1.11.1.7] | (RefSeq) peroxidase 59-like (A)	Peroxidase N; EC=1.11.1.7; Neutral peroxidase; Flags: Precursor (At5g19890)		2.207
orange1.1t02041	K00430 peroxidase [EC:1.11.1.7] | (RefSeq) peroxidase A2 (A)	Peroxidase C3; EC=1.11.1.7; Flags: Precursor (At5g06720)		-1.203
orange1.1t02045	K00430 peroxidase [EC:1.11.1.7] | (RefSeq) peroxidase 15-like (A)	Peroxidase 15 {ECO:0000303|PubMed:17936696}; Prx15 {ECO:0000303|PubMed:17936696}; EC=1.11.1.7; Anionic peroxidase {ECO:0000303|PubMed:17936696}; Flags: Precursor (At5g06720)	-1.018	
orange1.1t02059	K00430 peroxidase [EC:1.11.1.7] | (RefSeq) peroxidase A2 (A)	Lignin-forming anionic peroxidase; EC=1.11.1.7; TOPA; Flags: Precursor (At5g06720)		1.147
orange1.1t02220	K03671 thioredoxin 1 | (RefSeq) hypothetical protein (A)	Thioredoxin-like 3-1, chloroplastic; Thioredoxin WCRKC-1; Flags: Precursor (At5g06690)	1.286	1.531
orange1.1t02225	K00430 peroxidase [EC:1.11.1.7] | (RefSeq) LOW QUALITY PROTEIN: peroxidase A2-like (A)	Peroxidase 53; Atperox P53; EC=1.11.1.7; ATPA2; Flags: Precursor (At5g06720)		1.571
orange1.1t03701	K22038 volume-regulated anion channel | (RefSeq) TMV resistance protein N-like isoform X1 (A)	Putative disease resistance protein At4g11170 (At5g17680)		2.124
orange1.1t03802	K18875 enhanced disease susceptibility 1 protein | (RefSeq) protein EDS1-like (A)	Protein EDS1 {ECO:0000305}; Enhanced disease susceptibility 1 {ECO:0000303|PubMed:10077677} (At3g48090)	1.628	1.415
orange1.1t04722	K00799 glutathione S-transferase [EC:2.5.1.18] | (RefSeq) hypothetical protein (A)	Glutathione S-transferase U19; AtGSTU19; EC=2.5.1.18; GST class-tau member 19; Glutathione S-transferase 8 (At1g17180)		-7.884
orange1.1t05402	K00430 peroxidase [EC:1.11.1.7] | (RefSeq) peroxidase P7-like (A)	Peroxidase 52; Atperox P52; EC=1.11.1.7; ATP49; Flags: Precursor (At5g05340)		1.615
orange1.1t05657	K13691 pathogen-inducible salicylic acid glucosyltransferase [EC:2.4.1.-] | (RefSeq) hypothetical protein (A)	UDP-glycosyltransferase 74E2; EC=2.4.1.- (At1g05680)		-1.370
Cell redox homeostasis [GO:0045454; P = 0.5644 (P3CR vs. P3R) and 0.8700 (P5CR vs. P5R)]				
Cs2g06140	K07390 monothiol glutaredoxin | (RefSeq) uncharacterized protein LOC7458002 isoform X3 (A)	Monothiol glutaredoxin-S17 {ECO:0000303|PubMed:15170506}; AtGrxS17 {ECO:0000303|PubMed:15170506} (At4g04950)		1.024
Cs2g12960	K03671 thioredoxin 1 | (RefSeq) hypothetical protein (A)	Thioredoxin-like 3-3; Thioredoxin-like 1 (At3g53220)		-1.334
Cs2g13810	K03676 glutaredoxin 3 | (RefSeq) hypothetical protein (A)	Glutaredoxin-C1 (At5g14070)	-2.107	-3.511
Cs3g02660		(At3g11920)		1.586
Cs3g09460	K08654 proprotein convertase subtilisin/kexin type 5 [EC:3.4.21.-]	Probable nucleoredoxin 1-1; OsNrx1-1; EC=1.8.1.8 (At2g42060)		2.064

	| (RefSeq) uncharacterized protein LOC104445957 (A)			
Cs3g18420	K09527 DnaJ homolog subfamily C member 7 | (RefSeq) TPR repeat-containing thioredoxin TTL1 (A)	TPR repeat-containing thioredoxin TTL1; Tetratricopeptide repeat thioredoxin-like 1 (At5g65160)	-2.726	
Cs3g26640	K17609 nucleoredoxin [EC:1.8.1.8] | (RefSeq) probable nucleoredoxin 2 (A)	Probable nucleoredoxin 2; OsNrx2; EC=1.8.1.8 (At1g60420)	2.582	
Cs4g12290	K03671 thioredoxin 1 | (RefSeq) ATC; thioredoxin-like protein CXXS1 (A)	Thioredoxin-like protein CXXS1; AtCXXS1; Mono-cysteine thioredoxin 1 (At1g11530)		-1.459
Cs4g18320	K03676 glutaredoxin 3 | (RefSeq) hypothetical protein (A)	Glutaredoxin-C6; AtGrxC6; Protein ROXY 21 (At4g33040)	-1.376	-1.445
Cs5g10830	K03671 thioredoxin 1 | (RefSeq) thioredoxin-like 1-2, chloroplastic isoform X1 (A)	Thioredoxin-like 1-2, chloroplastic; Atypical cysteine/histidine-rich thioredoxin 5; AtACHT5; Lilium-type thioredoxin 1-2; Flags: Precursor (At5g61440)	-1.288	-1.433
Cs5g16520	K08654 proprotein convertase subtilisin/kexin type 5 [EC:3.4.21.-] | (RefSeq) uncharacterized protein LOC104445957 (A)	Probable nucleoredoxin 1; AtNrx1; EC=1.8.1.8 (At2g27660)		1.359
Cs7g07690	K17609 nucleoredoxin [EC:1.8.1.8] | (RefSeq) probable nucleoredoxin 3 (A)	Probable nucleoredoxin 3; AtNrx3; EC=1.8.1.8 (At4g31240)	1.132	
Cs7g29170	K03283 heat shock 70kDa protein 1/2/6/8 | (RefSeq) heat shock cognate 70 kDa protein 2-like isoform X1 (A)	Probable nucleoredoxin 1-2; OsNrx1-2; EC=1.8.1.8 (At2g16050)	2.726	2.927
Cs7g29190	K03283 heat shock 70kDa protein 1/2/6/8 | (RefSeq) heat shock cognate 70 kDa protein 2-like isoform X1 (A)	Probable nucleoredoxin 1-2; OsNrx1-2; EC=1.8.1.8 (At2g16050)	4.565	
Cs9g04770	K03676 glutaredoxin 3 | (RefSeq) hypothetical protein (A)	Monothiol glutaredoxin-S2; AtGrxS2; Protein ROXY 10 (At5g18600)		-1.770
orange1.1t02220	K03671 thioredoxin 1 | (RefSeq) hypothetical protein (A)	Thioredoxin-like 3-1, chloroplastic; Thioredoxin WCRKC-1; Flags: Precursor (At5g06690)	1.286	1.531
orange1.1t03073	K09527 DnaJ homolog subfamily C member 7 | (RefSeq) TPR repeat-containing thioredoxin TTL1-like (A)	TPR repeat-containing thioredoxin TTL1; Tetratricopeptide repeat thioredoxin-like 1 (At1g53300_1)	-1.215	
orange1.1t04754	K20367 endoplasmic reticulum-Golgi intermediate compartment protein 3 | (RefSeq) protein disulfide-isomerase 5-4-like (A)	Protein disulfide-isomerase 5-4; AtPDIL5-4; Protein disulfide-isomerase 7; PDI7; Protein disulfide-isomerase 8-2; AtPDIL8-2; Flags: Precursor (At4g27080)		1.361
Sulfur metabolism [ko00920; P = 0.6583 (P3CR vs. P3R) and 0.4794 (P5CR vs. P5R)]				
Cs1g06660	K01082 3'(2'), 5'-bisphosphate nucleotidase [EC:3.1.3.7] | (RefSeq) PAP-specific phosphatase HAL2-like (A)	PAP-specific phosphatase HAL2-like; 3'(2'),5'-bisphosphate nucleotidase; EC=3.1.3.7; 3'(2'),5-bisphosphonucleoside 3'(2')-phosphohydrolase; DPNPase; Halotolerance protein		-2.002

		(At5g54390)		
Cs1g21470	K00860 adenylylsulfate kinase [EC:2.7.1.25] | (RefSeq) adenylyl-sulfate kinase 3 (A)	Adenylyl-sulfate kinase, chloroplastic; EC=2.7.1.25; ATP adenosine-5'-phosphosulfate 3'-phosphotransferase; Adenosine-5'-phosphosulfate kinase; APS kinase; Flags: Precursor (At4g39940)		-1.407
Cs2g06630	K01082 3'(2'), 5'-bisphosphate nucleotidase [EC:3.1.3.7] | (RefSeq) PAP-specific phosphatase HAL2-like (A)	PAP-specific phosphatase HAL2-like; 3'(2'),5'-bisphosphate nucleotidase; EC=3.1.3.7; 3'(2'),5-bisphosphonucleoside 3'(2')-phosphohydrolase; DPNPase; Halotolerance protein (At5g54390)		1.257
Cs2g11750	K01082 3'(2'), 5'-bisphosphate nucleotidase [EC:3.1.3.7] | (RefSeq) Inositol monophosphatase, conserved site (A)	(At5g44730)	-2.047	
Cs3g11490	K01738 cysteine synthase A [EC:2.5.1.47] | (RefSeq) hypothetical protein (A)	Cysteine synthase, chloroplastic/chromoplastic; EC=2.5.1.47; CSase B; CS-B; O-acetylserine (thiol)-lyase; O-acetylserine sulfhydrylase; OAS-TL B; Flags: Precursor (At2g43750)	1.049	1.627
Cs3g11520	K01738 cysteine synthase A [EC:2.5.1.47] | (RefSeq) cysteine synthase-like (A)	Cysteine synthase {ECO:0000303|PubMed:8280125}; CSase; EC=2.5.1.47 {ECO:0000269|PubMed:8280125, ECO:0000269|PubMed:9013806}; Beta-PA/CSase; EC=2.5.1.51 {ECO:0000269|PubMed:8280125, ECO:0000269|PubMed:9013806}; Beta-pyrazolylalanine synthase {ECO:0000303|PubMed:8280125}; EC=4.2.1.50 {ECO:0000269|PubMed:8280125, ECO:0000269|PubMed:9013806}; L-mimosine synthase; EC=2.5.1.52 {ECO:0000269|PubMed:9013806}; O-acetylserine (thiol)-lyase; OAS-TL; O-acetylserine sulfhydrylase (At5g28020)		1.745
Cs9g07780	K13811 3'-phosphoadenosine 5'-phosphosulfate synthase [EC:2.7.7.4 2.7.1.25] | (RefSeq) hypothetical protein (A)	ATP sulfurylase 1, chloroplastic; AtPS1; EC=2.7.7.4; Flags: Precursor (At3g22890)	-1.997	-1.224
Cysteine and methionine metabolism [ko00270; P = 0.3614 (P3CR vs. P3R) and 0.6384 (P5CR vs. P5R)]				
Cs1g08010	K00815 tyrosine aminotransferase [EC:2.6.1.5] | (RefSeq) hypothetical protein (A)	Probable aminotransferase TAT2; EC=2.6.1.-; Tyrosine aminotransferase 2 (At5g53970)	1.218	
Cs1g25560	K00025 malate dehydrogenase [EC:1.1.1.37] | (RefSeq) malate dehydrogenase, cytoplasmic-like (A)	Oligopeptide transporter 7; AtOPT7 (At4g10770)	-1.049	
Cs2g02500	K05933 aminocyclopropanecarboxylate oxidase [EC:1.14.17.4] | (RefSeq) hypothetical protein (A)	1-aminocyclopropane-1-carboxylate oxidase; ACC oxidase; EC=1.14.17.4; Ethylene-forming enzyme; EFE (At1g05010)		-1.030

Cs2g14460	K00928 aspartate kinase [EC:2.7.2.4] | (RefSeq) aspartokinase 2, chloroplastic-like (A)	Aspartokinase 3, chloroplastic; EC=2.7.2.4; Aspartate kinase 3; Flags: Precursor (At3g02020)	-1.045	
Cs2g23840	K00815 tyrosine aminotransferase [EC:2.6.1.5] | (RefSeq) hypothetical protein (A)	Probable aminotransferase TAT2; EC=2.6.1.-; Tyrosine aminotransferase 2 (At5g53970)		1.425
Cs2g23850	K00815 tyrosine aminotransferase [EC:2.6.1.5] | (RefSeq) hypothetical protein (A)	Probable aminotransferase TAT2; EC=2.6.1.-; Tyrosine aminotransferase 2 (At5g53970)		1.042
Cs2g28930	K00549 5-methyltetrahydropteroyltriglutamate--homocysteine methyltransferase [EC:2.1.1.14] | (RefSeq) hypothetical protein (A)	5-methyltetrahydropteroyltriglutamatehomocysteine methyltransferase; EC=2.1.1.14; Cobalamin-independent methionine synthase isozyme; Vitamin-B12-independent methionine synthase isozyme (At5g17920)		1.292
Cs3g11490	K01738 cysteine synthase A [EC:2.5.1.47] | (RefSeq) hypothetical protein (A)	Cysteine synthase, chloroplastic/chromoplastic; EC=2.5.1.47; CSase B; CS-B; O-acetylserine (thiol)-lyase; O-acetylserine sulfhydrylase; OAS-TL B; Flags: Precursor (At2g43750)	1.049	1.627
Cs3g11520	K01738 cysteine synthase A [EC:2.5.1.47] | (RefSeq) cysteine synthase-like (A)	Cysteine synthase {ECO:0000303|PubMed:8280125}; CSase; EC=2.5.1.47 {ECO:0000269|PubMed:8280125, ECO:0000269|PubMed:9013806}; Beta-PA/CSase; EC=2.5.1.51 {ECO:0000269|PubMed:8280125, ECO:0000269|PubMed:9013806}; Beta-pyrazolylalanine synthase {ECO:0000303|PubMed:8280125}; EC=4.2.1.50 {ECO:0000269|PubMed:8280125, ECO:0000269|PubMed:9013806}; L-mimosine synthase; EC=2.5.1.52 {ECO:0000269|PubMed:9013806}; O-acetylserine (thiol)-lyase; OAS-TL; O-acetylserine sulfhydrylase (At5g28020)		1.745
Cs3g16400	K01762 1-aminocyclopropane-1-carboxylate synthase [EC:4.4.1.14] | (RefSeq) 1-aminocyclopropane-1-carboxylate synthase 8 (A)	1-aminocyclopropane-1-carboxylate synthase 8; ACC synthase 8; EC=4.4.1.14; S-adenosyl-L-methionine methylthioadenosine-lyase 8 (At4g37770)		2.594
Cs3g16440	K00826 branched-chain amino acid aminotransferase [EC:2.6.1.42] | (RefSeq) branched-chain-amino-acid aminotransferase 2, chloroplastic (A)	Branched-chain-amino-acid aminotransferase 2, chloroplastic; Atbcat-2; EC=2.6.1.42; Flags: Precursor (At1g10070)		1.092
Cs3g16870	K00025 malate dehydrogenase [EC:1.1.1.37] | (RefSeq) malate dehydrogenase, cytoplasmic-like (A)	Oligopeptide transporter 4; AtOPT4 (At5g64410)	1.016	
Cs4g02260	K01611 S-adenosylmethionine decarboxylase [EC:4.1.1.50] | (RefSeq) hypothetical protein (A)	S-adenosylmethionine decarboxylase proenzyme; AdoMetDC; SAMDC; EC=4.1.1.50; Contains: S-adenosylmethionine decarboxylase alpha chain; Contains: S-adenosylmethionine		1.316

		decarboxylase beta chain; Flags: Precursor (At3g25570)		
Cs4g08360	K00025 malate dehydrogenase [EC:1.1.1.37] | (RefSeq) malate dehydrogenase, cytoplasmic-like (A)	Oligopeptide transporter 1; AtOPT1 (At5g55930)	1.141	
Cs4g08370	K00025 malate dehydrogenase [EC:1.1.1.37] | (RefSeq) malate dehydrogenase, cytoplasmic-like (A)	Oligopeptide transporter 1; AtOPT1 (At5g55930)		-1.145
Cs4g08380	K00025 malate dehydrogenase [EC:1.1.1.37] | (RefSeq) malate dehydrogenase, cytoplasmic-like (A)	Oligopeptide transporter 1; AtOPT1 (At5g55930)		-1.837
Cs4g13490	K01762 1-aminocyclopropane-1-carboxylate synthase [EC:4.4.1.14] | (RefSeq) 1-aminocyclopropane-1-carboxylate synthase 7 (A)	1-aminocyclopropane-1-carboxylate synthase 7; ACC synthase 7; EC=4.4.1.14; S-adenosyl-L-methionine methylthioadenosine-lyase 7 (At4g26200)		1.634
Cs4g13870	K05933 aminocyclopropanecarboxylate oxidase [EC:1.14.17.4] | (RefSeq) hypothetical protein (A)	1-aminocyclopropane-1-carboxylate oxidase 1; ACC oxidase 1; AtACO1; EC=1.14.17.4 (At2g19590)	-1.124	
Cs5g01510	K01761 methionine-gamma-lyase [EC:4.4.1.11] | (RefSeq) methionine gamma-lyase (A)	Methionine gamma-lyase; AtMGL; EC=4.4.1.11; L-methioninase (At1g64660)		-1.687
Cs5g03060	K20772 1-aminocyclopropane-1-carboxylate synthase 1/2/6 [EC:4.4.1.14] | (RefSeq) hypothetical protein (A)	1-aminocyclopropane-1-carboxylate synthase; ACC synthase; EC=4.4.1.14; S-adenosyl-L-methionine methylthioadenosine-lyase (At4g11280)	1.327	1.903
Cs5g04240	K00549 5-methyltetrahydropteroyltriglutamate--homocysteine methyltransferase [EC:2.1.1.14] | (RefSeq) protein QUIRKY-like (A)	Protein QUIRKY {ECO:0000303|PubMed:19180193} (At1g74720)		1.383
Cs5g27350	K00549 5-methyltetrahydropteroyltriglutamate--homocysteine methyltransferase [EC:2.1.1.14] | (RefSeq) protein QUIRKY-like (A)	Protein QUIRKY {ECO:0000303|PubMed:19180193} (At3g03680)	-1.466	-1.678
Cs6g09640	K00827 alanine-glyoxylate transaminase / (R)-3-amino-2-methylpropionate-pyruvate transaminase [EC:2.6.1.44 2.6.1.40] | (RefSeq) hypothetical protein (A)	Alanineglyoxylate aminotransferase 2 homolog 3, mitochondrial; EC=2.6.1.44; Beta-alanine-pyruvate aminotransferase 3; Flags: Precursor (At3g08860)		-1.565
Cs6g09690	K14455 aspartate aminotransferase, mitochondrial [EC:2.6.1.1] | (RefSeq) hypothetical protein (A)	Aspartate aminotransferase, mitochondrial; EC=2.6.1.1; Transaminase A; Flags: Precursor (At2g30970)	-1.627	
Cs6g19210	K01611 S-adenosylmethionine decarboxylase [EC:4.1.1.50] | (RefSeq) S-adenosylmethionine decarboxylase proenzyme (A)	S-adenosylmethionine decarboxylase proenzyme; AdoMetDC; SAMDC; EC=4.1.1.50; Contains: S-adenosylmethionine decarboxylase alpha chain; Contains: S-adenosylmethionine decarboxylase beta chain; Flags: Precursor (At3g02470)		-1.025
Cs7g13320	K00547 homocysteine S-methyltransferase [EC:2.1.1.10] | (RefSeq) hypothetical protein (A)	Homocysteine S-methyltransferase 1; BoHMT1; EC=2.1.1.10 (At3g25900)		1.047

Cs7g16800	K00558 DNA (cytosine-5)-methyltransferase 1 [EC:2.1.1.37] | (RefSeq) DNA (cytosine-5)-methyltransferase CMT2-like (A)	(At2g14640_1)	1.698	1.533
Cs8g02970	K00025 malate dehydrogenase [EC:1.1.1.37] | (RefSeq) malate dehydrogenase, cytoplasmic-like (A)	Oligopeptide transporter 4; AtOPT4 (At5g64410)	-1.428	
Cs8g03410	K00826 branched-chain amino acid aminotransferase [EC:2.6.1.42] | (RefSeq) hypothetical protein (A)	Branched-chain-amino-acid aminotransferase 2, chloroplastic; Atbcat-2; EC=2.6.1.42; Flags: Precursor (At1g10070)	1.282	
Cs9g05430	K01611 S-adenosylmethionine decarboxylase [EC:4.1.1.50] | (RefSeq) S-adenosylmethionine decarboxylase proenzyme 4 (A)	S-adenosylmethionine decarboxylase proenzyme 4 {ECO:0000305}; AdoMetDC4 {ECO:0000305}; EC=4.1.1.50 {ECO:0000269|PubMed:16699540}; Contains: S-adenosylmethionine decarboxylase 1 alpha chain {ECO:0000250|UniProtKB:P17707}; Contains: S-adenosylmethionine decarboxylase 1 beta chain {ECO:0000250|UniProtKB:P17707}; Protein BUSHY AND DWARF 2 {ECO:0000303|PubMed:16699540}; Flags: Precursor (At5g18930)		-1.207
Cs9g07420	K00547 homocysteine S-methyltransferase [EC:2.1.1.10] | (RefSeq) homocysteine S-methyltransferase 3 (A)	Homocysteine S-methyltransferase 3; EC=2.1.1.10; S-methylmethionine:homocysteine methyltransferase 3; AtHMT-3; SMM:Hcy S-methyltransferase 3 (At3g22740)	1.233	
novel.25	K00815 tyrosine aminotransferase [EC:2.6.1.5] | (RefSeq) LOW QUALITY PROTEIN: tyrosine aminotransferase-like (A)	(At2g06190)	-1.347	
orange1.1t00345	K00549 5-methyltetrahydropteroyltriglutamate--homocysteine methyltransferase [EC:2.1.1.14] | (RefSeq) protein QUIRKY-like (A)	FT-interacting protein 1 {ECO:0000303|PubMed:22529749} (At4g11610)	1.167	1.418
orange1.1t00416	K20772 1-aminocyclopropane-1-carboxylate synthase 1/2/6 [EC:4.4.1.14] | (RefSeq) hypothetical protein (A)	1-aminocyclopropane-1-carboxylate synthase 1; ACC synthase 1; EC=4.4.1.14; S-adenosyl-L-methionine methylthioadenosine-lyase (At3g61510)	2.773	
orange1.1t02074	K00558 DNA (cytosine-5)-methyltransferase 1 [EC:2.1.1.37] | (RefSeq) LOW QUALITY PROTEIN: DNA (cytosine-5)-methyltransferase CMT3-like (A)			-1.148
orange1.1t04496	K00058 D-3-phosphoglycerate dehydrogenase / 2-oxoglutarate reductase [EC:1.1.1.95 1.1.1.399] | (RefSeq) D-3-phosphoglycerate dehydrogenase 2, chloroplastic-like (A)	D-3-phosphoglycerate dehydrogenase 2, chloroplastic; PGDH; EC=1.1.1.95 {ECO:0000269|PubMed:24368794, ECO:0000269|PubMed:9867856}; Flags: Precursor (At1g17740)		1.349
Sulfur compound metabolic process [GO:0006790; P = 0.2442 (P3CR vs. P3R) and 0.0235 (P5CR vs. P5R)]				

Cs1g06660	K01082 3'(2'), 5'-bisphosphate nucleotidase [EC:3.1.3.7] | (RefSeq) PAP-specific phosphatase HAL2-like (A)	PAP-specific phosphatase HAL2-like; 3'(2'),5'-bisphosphate nucleotidase; EC=3.1.3.7; 3'(2'),5-bisphosphonucleoside 3'(2')-phosphohydrolase; DPNPase; Halotolerance protein (At5g54390)		-2.002
Cs1g15710	K05681 ATP-binding cassette, subfamily G (WHITE), member 2 | (RefSeq) hypothetical protein (A)	ABC transporter G family member 36; ABC transporter ABCG.36; AtABCG36; Pleiotropic drug resistance protein 8; Protein PENETRATION 3 (At1g59870)		-2.119
Cs1g21310	K09285 AP2-like factor, ANT lineage | (RefSeq) AP2-like ethylene-responsive transcription factor ANT (A)	AP2-like ethylene-responsive transcription factor ANT; Complementing a protein kinase C mutant protein 1; Protein AINTEGUMENTA; Protein DRAGON; Protein OVULE MUTANT (At4g37750)		1.416
Cs1g21350	K22382 WD repeat-containing protein 26 | (RefSeq) WD repeat-containing protein 26 homolog (A)	Growth-regulating factor 1; AtGRF1; Transcription activator GRF1 (At2g22840)		-1.225
Cs1g21470	K00860 adenylylsulfate kinase [EC:2.7.1.25] | (RefSeq) adenylyl-sulfate kinase 3 (A)	Adenylyl-sulfate kinase, chloroplastic; EC=2.7.1.25; ATP adenosine-5'-phosphosulfate 3'-phosphotransferase; Adenosine-5'-phosphosulfate kinase; APS kinase; Flags: Precursor (At4g39940)		-1.407
Cs2g06630	K01082 3'(2'), 5'-bisphosphate nucleotidase [EC:3.1.3.7] | (RefSeq) PAP-specific phosphatase HAL2-like (A)	PAP-specific phosphatase HAL2-like; 3'(2'),5'-bisphosphate nucleotidase; EC=3.1.3.7; 3'(2'),5-bisphosphonucleoside 3'(2')-phosphohydrolase; DPNPase; Halotolerance protein (At5g54390)		1.257
Cs2g19240	K20285 Rab9 effector protein with kelch motifs | (RefSeq) nitrile-specifier protein 5-like (A)	Nitrile-specifier protein 5; AtNSP5 (At3g07720)		1.201
Cs2g22030	K03521 electron transfer flavoprotein beta subunit | (RefSeq) electron transfer flavoprotein subunit beta, mitochondrial-like (A)	Transcription factor ILR3; Basic helix-loop-helix protein 105; AtbHLH105; bHLH 105; Protein IAA-LEUCINE RESISTANT 3; Transcription factor EN 133; bHLH transcription factor bHLH105 (At5g54680)		1.631
Cs2g28930	K00549 5-methyltetrahydropteroyltriglutamate--homocysteine methyltransferase [EC:2.1.1.14] | (RefSeq) hypothetical protein (A)	5-methyltetrahydropteroyltriglutamatehomocysteine methyltransferase; EC=2.1.1.14; Cobalamin-independent methionine synthase isozyme; Vitamin-B12-independent methionine synthase isozyme (At5g17920)		1.292
Cs2g30630	K00943 dTMP kinase [EC:2.7.4.9] | (RefSeq) glutathione S-transferase T3-like (A)	Glutathione S-transferase T3; AtGSTT3; EC=2.5.1.18; GST class-theta member 3; Glutathione S-transferase 10C (At2g22710)	-1.904	-1.560
Cs3g11490	K01738 cysteine synthase A [EC:2.5.1.47] | (RefSeq) hypothetical	Cysteine synthase, chloroplastic/chromoplastic; EC=2.5.1.47; CSase	1.049	1.627

	protein (A)	B; CS-B; O-acetylserine (thiol)-lyase; O-acetylserine sulfhydrylase; OAS-TL B; Flags: Precursor (At2g43750)		
Cs3g11520	K01738 cysteine synthase A [EC:2.5.1.47] | (RefSeq) cysteine synthase-like (A)	Cysteine synthase {ECO:0000303|PubMed:8280125}; CSase; EC=2.5.1.47 {ECO:0000269|PubMed:8280125, ECO:0000269|PubMed:9013806}; Beta-PA/CSase; EC=2.5.1.51 {ECO:0000269|PubMed:8280125, ECO:0000269|PubMed:9013806}; Beta-pyrazolylalanine synthase {ECO:0000303|PubMed:8280125}; EC=4.2.1.50 {ECO:0000269|PubMed:8280125, ECO:0000269|PubMed:9013806}; L-mimosine synthase; EC=2.5.1.52 {ECO:0000269|PubMed:9013806}; O-acetylserine (thiol)-lyase; OAS-TL; O-acetylserine sulfhydrylase (At5g28020)		1.745
Cs3g18630	K11262 acetyl-CoA carboxylase / biotin carboxylase 1 [EC:6.4.1.2 6.3.4.14 2.1.3.15] | (RefSeq) acetyl-CoA carboxylase 1-like (A)	Acetyl-CoA carboxylase 1; AtACC1; EC=6.4.1.2 {ECO:0000269|PubMed:9008389}; Protein EMBRYO DEFECTIVE 22; Protein GURKE; Protein PASTICCINO 3; Includes: Biotin carboxylase; EC=6.3.4.14 (At1g36160)		1.119
Cs3g20220	K22077 ganglioside-induced differentiation-associated protein 1 | (RefSeq) glutathione S-transferase TCHQD (A)	Glutathione S-transferase TCHQD; EC=2.5.1.18; Protein tetrachlorohydroquinone dehalogenase-homolog (At1g77290)		1.056
Cs3g21550	K20617 cytochrome P450 family 71 subfamily A | (RefSeq) hypothetical protein (A)	Cytochrome P450 83B1; EC=1.14.14.45 {ECO:0000269|PubMed:12970475}; Protein ALTERED TRYPTOPHAN REGULATION 4; Protein RED ELONGATED 1; Protein SUPERROOT 2 (At4g31500)		2.005
Cs3g25780	K20617 cytochrome P450 family 71 subfamily A | (RefSeq) cytochrome P450 71A1-like (A)	Cytochrome P450 83B1; EC=1.14.14.45 {ECO:0000269|PubMed:12970475}; Protein ALTERED TRYPTOPHAN REGULATION 4; Protein RED ELONGATED 1; Protein SUPERROOT 2 (At4g31500)	3.636	
Cs4g02260	K01611 S-adenosylmethionine decarboxylase [EC:4.1.1.50] | (RefSeq) hypothetical protein (A)	S-adenosylmethionine decarboxylase proenzyme; AdoMetDC; SAMDC; EC=4.1.1.50; Contains: S-adenosylmethionine decarboxylase alpha chain; Contains: S-adenosylmethionine decarboxylase beta chain; Flags: Precursor (At3g25570)		1.316
Cs4g03720	K22074 NFU1 iron-sulfur cluster scaffold homolog, mitochondrial | (RefSeq) nifU-like protein 1, chloroplastic (A)	NifU-like protein 3, chloroplastic; AtCNfu3; AtCnfU-IVa; Flags: Precursor (At4g25910)	-1.172	-2.083
Cs4g06620	K14157 alpha-aminoadipic semialdehyde synthase [EC:1.5.1.8 1.5.1.9] | (RefSeq) alpha-aminoadipic semialdehyde synthase (A)	Alpha-aminoadipic semialdehyde synthase; cAt-LKR/SDH; LKR/SDH; Includes: Lysine ketoglutarate reductase; LKR;	1.865	

		EC=1.5.1.8; Includes: Saccharopine dehydrogenase; EC=1.5.1.9; cAt-SDH; SDH (At4g33150)		
Cs4g11090	K03146 thiamine thiazole synthase | (RefSeq) THI1, thi; thiamine thiazole synthase, chloroplastic-like (A)	Thiamine thiazole synthase, chloroplastic {ECO:0000255|HAMAP-Rule:MF_03158}; Thiazole biosynthetic enzyme {ECO:0000255|HAMAP-Rule:MF_03158}; Flags: Precursor (At5g54770)	1.144	1.498
Cs4g13090	K18592 gamma-glutamyltranspeptidase / glutathione hydrolase / leukotriene-C4 hydrolase [EC:2.3.2.2 3.4.19.13 3.4.19.14] | (RefSeq) gamma-glutamyltranspeptidase 3-like (A)	Glutathione hydrolase 3; EC=3.4.19.13; Gamma-glutamyltransferase 3; Gamma-glutamyltranspeptidase 3; EC=2.3.2.2; Gamma-glutamyltranspeptidase 4 (At4g29210)		1.227
Cs5g01510	K01761 methionine-gamma-lyase [EC:4.4.1.11] | (RefSeq) methionine gamma-lyase (A)	Methionine gamma-lyase; AtMGL; EC=4.4.1.11; L-methioninase (At1g64660)		-1.687
Cs5g03440	K15398 fatty acid omega-hydroxylase [EC:1.14.-.-] | (RefSeq) cytochrome P450 86A22 (A)	Cytochrome P450 86A22 {ECO:0000303|PubMed:19940120}; EC=1.14.13.204 {ECO:0000269|PubMed:19940120}; Long-chain acyl-CoA omega-monooxygenase {ECO:0000305} (At2g45970)	1.219	2.145
Cs5g03810	K00799 glutathione S-transferase [EC:2.5.1.18] | (RefSeq) hypothetical protein (A)	Glutathione S-transferase T1; AtGSTT1; EC=2.5.1.18; GST class-theta member 1; Glutathione S-transferase 10 (At5g41210)		1.212
Cs5g03900	K00799 glutathione S-transferase [EC:2.5.1.18] | (RefSeq) glutathione S-transferase U10-like (A)	Glutathione S-transferase U10; AtGSTU10; EC=2.5.1.18; GST class-tau member 10 (At1g74590)	1.140	-1.094
Cs5g12370	K01025 sulfotransferase [EC:2.8.2.-] | (RefSeq) flavonol 4'-sulfotransferase-like (A)	Cytosolic sulfotransferase 16; AtSOT16; EC=2.8.2.24 {ECO:0000269|PubMed:19077143}; Aromatic desulfoglucosinolate sulfotransferase {ECO:0000305}; Desulfoglucosinolate sulfotransferase A; AtST5a; Protein CORONATINE INDUCED 7 (At1g74100)		1.153
Cs5g25060	K20617 cytochrome P450 family 71 subfamily A | (RefSeq) cytochrome P450 71A1-like (A)	Cytochrome P450 83B1; EC=1.14.14.45 {ECO:0000269|PubMed:12970475}; Protein ALTERED TRYPTOPHAN REGULATION 4; Protein RED ELONGATED 1; Protein SUPERROOT 2 (At4g31500)		-1.027
Cs5g25070	K20617 cytochrome P450 family 71 subfamily A | (RefSeq) cytochrome P450 71A1-like (A)	Cytochrome P450 83B1; EC=1.14.14.45 {ECO:0000269|PubMed:12970475}; Protein ALTERED TRYPTOPHAN REGULATION 4; Protein RED ELONGATED 1; Protein SUPERROOT 2 (At4g31500)		2.331
Cs5g26130	K00521 ferric-chelate reductase [EC:1.16.1.7] | (RefSeq) ferric reduction oxidase 7, chloroplastic-like (A)	NAC transcription factor 47 {ECO:0000305}; NAC domain-containing protein 47 {ECO:0000305}; ANAC047 {ECO:0000305}; Protein SPEEDY HYPONASTIC GROWTH		1.004

		{ECO:0000303|PubMed:24363315} (At3g04070)		
Cs5g32780	K00799 glutathione S-transferase [EC:2.5.1.18] | (RefSeq) glutathione S-transferase F13 (A)	Glutathione S-transferase F13; AtGSTF13; EC=2.5.1.18; GST class-phi member 13 (At3g62760)	-1.125	
Cs5g34830		Protein SULFUR DEFICIENCY-INDUCED 1 {ECO:0000303|PubMed:19154231}; AtSDI1 {ECO:0000303|PubMed:19154231} (At5g48850)	2.690	
Cs6g03820	K00799 glutathione S-transferase [EC:2.5.1.18] | (RefSeq) glutathione S-transferase L3-like (A)	Glutathione S-transferase L3; AtGSTL3; EC=2.5.1.18; GST class-lambda member 3 (At5g02790)	1.213	
Cs6g03830	K00799 glutathione S-transferase [EC:2.5.1.18] | (RefSeq) glutathione S-transferase L3-like (A)	Glutathione S-transferase L3; AtGSTL3; EC=2.5.1.18; GST class-lambda member 3 (At5g02790)		1.372
Cs6g03850	K00799 glutathione S-transferase [EC:2.5.1.18] | (RefSeq) glutathione S-transferase L3-like (A)	Glutathione S-transferase L3; AtGSTL3; EC=2.5.1.18; GST class-lambda member 3 (At5g02790)		1.249
Cs6g07240	K00799 glutathione S-transferase [EC:2.5.1.18] | (RefSeq) glutathione S-transferase U8-like (A)	Probable glutathione S-transferase; EC=2.5.1.18; G2-4; Heat shock protein 26A (At3g09270)	1.642	1.194
Cs6g09420	K13424 WRKY transcription factor 33 | (RefSeq) hypothetical protein (A)	Probable WRKY transcription factor 33; WRKY DNA-binding protein 33 (At2g38470)	1.908	1.729
Cs6g15360	K09286 EREBP-like factor | (RefSeq) dehydration-responsive element-binding protein 1F-like (A)	Dehydration-responsive element-binding protein 1D; Protein DREB1D; C-repeat/dehydration-responsive element-binding factor 4; C-repeat-binding factor 4; CRT/DRE-binding factor 4 (At5g51990)	-1.480	-1.520
Cs6g19210	K01611 S-adenosylmethionine decarboxylase [EC:4.1.1.50] | (RefSeq) S-adenosylmethionine decarboxylase proenzyme (A)	S-adenosylmethionine decarboxylase proenzyme; AdoMetDC; SAMDC; EC=4.1.1.50; Contains: S-adenosylmethionine decarboxylase alpha chain; Contains: S-adenosylmethionine decarboxylase beta chain; Flags: Precursor (At3g02470)		-1.025
Cs6g21240	K08026 one cut domain, family member 1, hepatocyte nuclear factor 6 | (RefSeq) zinc-finger homeodomain protein 9-like (A)	Zinc-finger homeodomain protein 9; AtZHD9; Homeobox protein 34; AtHB-34 (At3g28920)	-1.030	
Cs7g04580	K00799 glutathione S-transferase [EC:2.5.1.18] | (RefSeq) glutathione S-transferase U17-like (A)	Glutathione S-transferase U17; AtGSTU17; EC=2.5.1.18; GST class-tau member 17; Glutathione S-transferase 30; Protein EARLY RESPONSIVE TO DEHYDRATION 9 (At1g10360)		-1.751
Cs7g04600	K00799 glutathione S-transferase [EC:2.5.1.18] | (RefSeq) glutathione S-transferase U17-like (A)	Glutathione S-transferase U17; AtGSTU17; EC=2.5.1.18; GST class-tau member 17; Glutathione S-transferase 30; Protein EARLY RESPONSIVE TO DEHYDRATION 9 (At1g10360)		-1.385
Cs7g08110	K11820 N-hydroxythioamide S-beta-glucosyltransferase [EC:2.4.1.195] | (RefSeq) UDP-glycosyltransferase 74B1 (A)	UDP-glycosyltransferase 74B1; EC=2.4.1.-; N-hydroxythioamide S-beta-glucosyltransferase; EC=2.4.1.195; Thiohydroximate	-1.156	-2.258

		S-glucosyltransferase (At1g24100)		
Cs7g13320	K00547 homocysteine S-methyltransferase [EC:2.1.1.10] | (RefSeq) hypothetical protein (A)	Homocysteine S-methyltransferase 1; BoHMT1; EC=2.1.1.10 (At3g25900)		1.047
Cs7g17600	K00521 ferric-chelate reductase [EC:1.16.1.7] | (RefSeq) ferric reduction oxidase 7, chloroplastic-like (A)	Transcription factor JUNGBRUNNEN 1; NAC domain-containing protein 42; ANAC042 (At2g43000)	1.057	1.759
Cs7g18550	K00799 glutathione S-transferase [EC:2.5.1.18] | (RefSeq) glutathione S-transferase F9-like (A)	Glutathione S-transferase F9; AtGSTF9; EC=2.5.1.18; AtGSTF7; GST class-phi member 9 (At2g30860)	1.427	3.342
Cs7g30780	K22920 UTP-glucose-1-phosphate uridylyltransferase [EC:2.7.7.9] | (RefSeq) hypothetical protein (A)	UTPglucose-1-phosphate uridylyltransferase 3, chloroplastic {ECO:0000305}; EC=2.7.7.9 {ECO:0000269|PubMed:19286968}; UDP-glucose pyrophosphorylase 3 {ECO:0000303|PubMed:19286968}; Flags: Precursor (At3g56040)		1.098
Cs8g15220	K13356 alcohol-forming fatty acyl-CoA reductase [EC:1.2.1.84] | (RefSeq) fatty acyl-CoA reductase 3-like (A)	Fatty acyl-CoA reductase 3 {ECO:0000305}; EC=1.2.1.84 {ECO:0000269|PubMed:16980563, ECO:0000269|PubMed:19062129}; Protein ECERIFERUM 4 (At4g33790)	3.039	6.671
Cs8g17360	K20536 mitogen-activated protein kinase 3 [EC:2.7.11.24] | (RefSeq) MAPK1, MAP; mitogen-activated protein kinase 1 (A)	Mitogen-activated protein kinase 3; AtMPK3; MAP kinase 3; EC=2.7.11.24 (At3g45640)		1.145
Cs8g19390	K00799 glutathione S-transferase [EC:2.5.1.18] | (RefSeq) hypothetical protein (A)	Glutathione S-transferase U7; AtGSTU7; EC=2.5.1.18; GST class-tau member 7; Glutathione S-transferase 25 (At2g29420)		1.371
Cs9g05430	K01611 S-adenosylmethionine decarboxylase [EC:4.1.1.50] | (RefSeq) S-adenosylmethionine decarboxylase proenzyme 4 (A)	S-adenosylmethionine decarboxylase proenzyme 4 {ECO:0000305}; AdoMetDC4 {ECO:0000305}; EC=4.1.1.50 {ECO:0000269|PubMed:16699540}; Contains: S-adenosylmethionine decarboxylase 1 alpha chain {ECO:0000250|UniProtKB:P17707}; Contains: S-adenosylmethionine decarboxylase 1 beta chain {ECO:0000250|UniProtKB:P17707}; Protein BUSHY AND DWARF 2 {ECO:0000303|PubMed:16699540}; Flags: Precursor (At5g18930)		-1.207
Cs9g07420	K00547 homocysteine S-methyltransferase [EC:2.1.1.10] | (RefSeq) homocysteine S-methyltransferase 3 (A)	Homocysteine S-methyltransferase 3; EC=2.1.1.10; S-methylmethionine:homocysteine methyltransferase 3; AtHMT-3; SMM:Hcy S-methyltransferase 3 (At3g22740)	1.233	
Cs9g07780	K13811 3'-phosphoadenosine 5'-phosphosulfate synthase [EC:2.7.7.4 2.7.1.25] | (RefSeq) hypothetical protein (A)	ATP sulfurylase 1, chloroplastic; AtPS1; EC=2.7.7.4; Flags: Precursor (At3g22890)	-1.997	-1.224
Cs9g10430	K00799 glutathione S-transferase [EC:2.5.1.18] | (RefSeq)	Probable glutathione S-transferase; EC=2.5.1.18; G2-4; Heat shock	4.021	

	glutathione transferase GST 23-like (A)	protein 26A (At3g09270)		
orange1.1t02087	K20617 cytochrome P450 family 71 subfamily A | (RefSeq) cytochrome P450 71A1-like (A)	Cytochrome P450 83B1; EC=1.14.14.45 {ECO:0000269|PubMed:12970475}; Protein ALTERED TRYPTOPHAN REGULATION 4; Protein RED ELONGATED 1; Protein SUPERROOT 2 (At4g31500)		-2.078
orange1.1t02246	K13356 alcohol-forming fatty acyl-CoA reductase [EC:1.2.1.84] | (RefSeq) fatty acyl-CoA reductase 2-like (A)	Fatty acyl-CoA reductase 2 {ECO:0000305}; EC=1.2.1.84 {ECO:0000269|PubMed:19062129}; Fatty acid reductase 2; Male sterility protein 2 (At3g11980)	-1.567	
orange1.1t02662	K20604 mitogen-activated protein kinase kinase 9 [EC:2.7.12.2] | (RefSeq) mitogen-activated protein kinase kinase 9 (A)	Mitogen-activated protein kinase kinase 9; AtMKK9; MAP kinase kinase 9; EC=2.7.12.2 (At1g73500)	-1.280	-1.159
orange1.1t02796	K20617 cytochrome P450 family 71 subfamily A | (RefSeq) cytochrome P450 71A1-like (A)	Cytochrome P450 83B1; EC=1.14.14.45 {ECO:0000269|PubMed:12970475}; Protein ALTERED TRYPTOPHAN REGULATION 4; Protein RED ELONGATED 1; Protein SUPERROOT 2 (At4g31500)	2.393	1.485
orange1.1t03456	K00799 glutathione S-transferase [EC:2.5.1.18] | (RefSeq) probable glutathione S-transferase (A)	Probable glutathione S-transferase; EC=2.5.1.18; G2-4; Heat shock protein 26A (At2g29420)	3.694	
orange1.1t03618	K00799 glutathione S-transferase [EC:2.5.1.18] | (RefSeq) probable glutathione S-transferase (A)	Probable glutathione S-transferase; EC=2.5.1.18; G2-4; Heat shock protein 26A (At2g29420)	1.186	
orange1.1t03624	K00799 glutathione S-transferase [EC:2.5.1.18] | (RefSeq) probable glutathione S-transferase (A)	Probable glutathione S-transferase; EC=2.5.1.18; G2-4; Heat shock protein 26A (At2g29420)		-1.245
orange1.1t04722	K00799 glutathione S-transferase [EC:2.5.1.18] | (RefSeq) hypothetical protein (A)	Glutathione S-transferase U19; AtGSTU19; EC=2.5.1.18; GST class-tau member 19; Glutathione S-transferase 8 (At1g17180)		-7.884
orange1.1t05897	K01012 biotin synthase [EC:2.8.1.6] | (RefSeq) biotin synthase-like (A)	Biotin synthase, mitochondrial; EC=2.8.1.6; Flags: Precursor (At2g43360)		-2.375
Sulfur compound biosynthetic process [GO:0044272; P = 0.4997 (P3CR vs. P3R) and 0.0442 (P5CR vs. P5R)]				
Cs1g21470	K00860 adenylylsulfate kinase [EC:2.7.1.25] | (RefSeq) adenylyl-sulfate kinase 3 (A)	Adenylyl-sulfate kinase, chloroplastic; EC=2.7.1.25; ATP adenosine-5'-phosphosulfate 3'-phosphotransferase; Adenosine-5'-phosphosulfate kinase; APS kinase; Flags: Precursor (At4g39940)		-1.407
Cs2g28930	K00549 5-methyltetrahydropteroyltriglutamate--homocysteine methyltransferase [EC:2.1.1.14] | (RefSeq) hypothetical protein (A)	5-methyltetrahydropteroyltriglutamatehomocysteine methyltransferase; EC=2.1.1.14; Cobalamin-independent methionine synthase isozyme; Vitamin-B12-independent methionine synthase isozyme (At5g17920)		1.292

Cs3g11490	K01738 cysteine synthase A [EC:2.5.1.47] | (RefSeq) hypothetical protein (A)	Cysteine synthase, chloroplastic/chromoplastic; EC=2.5.1.47; CSase B; CS-B; O-acetylserine (thiol)-lyase; O-acetylserine sulfhydrylase; OAS-TL B; Flags: Precursor (At2g43750)	1.049	1.627
Cs3g11520	K01738 cysteine synthase A [EC:2.5.1.47] | (RefSeq) cysteine synthase-like (A)	Cysteine synthase {ECO:0000303|PubMed:8280125}; CSase; EC=2.5.1.47 {ECO:0000269|PubMed:8280125, ECO:0000269|PubMed:9013806}; Beta-PA/CSase; EC=2.5.1.51 {ECO:0000269|PubMed:8280125, ECO:0000269|PubMed:9013806}; Beta-pyrazolylalanine synthase {ECO:0000303|PubMed:8280125}; EC=4.2.1.50 {ECO:0000269|PubMed:8280125, ECO:0000269|PubMed:9013806}; L-mimosine synthase; EC=2.5.1.52 {ECO:0000269|PubMed:9013806}; O-acetylserine (thiol)-lyase; OAS-TL; O-acetylserine sulfhydrylase (At5g28020)		1.745
Cs3g18630	K11262 acetyl-CoA carboxylase / biotin carboxylase 1 [EC:6.4.1.2 6.3.4.14 2.1.3.15] | (RefSeq) acetyl-CoA carboxylase 1-like (A)	Acetyl-CoA carboxylase 1; AtACC1; EC=6.4.1.2 {ECO:0000269|PubMed:9008389}; Protein EMBRYO DEFECTIVE 22; Protein GURKE; Protein PASTICCINO 3; Includes: Biotin carboxylase; EC=6.3.4.14 (At1g36160)		1.119
Cs3g21550	K20617 cytochrome P450 family 71 subfamily A | (RefSeq) hypothetical protein (A)	Cytochrome P450 83B1; EC=1.14.14.45 {ECO:0000269|PubMed:12970475}; Protein ALTERED TRYPTOPHAN REGULATION 4; Protein RED ELONGATED 1; Protein SUPERROOT 2 (At4g31500)		2.005
Cs3g25780	K20617 cytochrome P450 family 71 subfamily A | (RefSeq) cytochrome P450 71A1-like (A)	Cytochrome P450 83B1; EC=1.14.14.45 {ECO:0000269|PubMed:12970475}; Protein ALTERED TRYPTOPHAN REGULATION 4; Protein RED ELONGATED 1; Protein SUPERROOT 2 (At4g31500)	3.636	
Cs4g02260	K01611 S-adenosylmethionine decarboxylase [EC:4.1.1.50] | (RefSeq) hypothetical protein (A)	S-adenosylmethionine decarboxylase proenzyme; AdoMetDC; SAMDC; EC=4.1.1.50; Contains: S-adenosylmethionine decarboxylase alpha chain; Contains: S-adenosylmethionine decarboxylase beta chain; Flags: Precursor (At3g25570)		1.316
Cs4g11090	K03146 thiamine thiazole synthase | (RefSeq) THI1, thi; thiamine thiazole synthase, chloroplastic-like (A)	Thiamine thiazole synthase, chloroplastic {ECO:0000255|HAMAP-Rule:MF_03158}; Thiazole biosynthetic enzyme {ECO:0000255|HAMAP-Rule:MF_03158}; Flags: Precursor (At5g54770)	1.144	1.498
Cs5g01510	K01761 methionine-gamma-lyase [EC:4.4.1.11] | (RefSeq)	Methionine gamma-lyase; AtMGL; EC=4.4.1.11; L-methioninase		-1.687

	methionine gamma-lyase (A)	(At1g64660)		
Cs5g12370	K01025 sulfotransferase [EC:2.8.2.-] | (RefSeq) flavonol 4'-sulfotransferase-like (A)	Cytosolic sulfotransferase 16; AtSOT16; EC=2.8.2.24 {ECO:0000269|PubMed:19077143}; Aromatic desulfoglucosinolate sulfotransferase {ECO:0000305}; Desulfoglucosinolate sulfotransferase A; AtST5a; Protein CORONATINE INDUCED 7 (At1g74100)		1.153
Cs5g25060	K20617 cytochrome P450 family 71 subfamily A | (RefSeq) cytochrome P450 71A1-like (A)	Cytochrome P450 83B1; EC=1.14.14.45 {ECO:0000269|PubMed:12970475}; Protein ALTERED TRYPTOPHAN REGULATION 4; Protein RED ELONGATED 1; Protein SUPERROOT 2 (At4g31500)		-1.027
Cs5g25070	K20617 cytochrome P450 family 71 subfamily A | (RefSeq) cytochrome P450 71A1-like (A)	Cytochrome P450 83B1; EC=1.14.14.45 {ECO:0000269|PubMed:12970475}; Protein ALTERED TRYPTOPHAN REGULATION 4; Protein RED ELONGATED 1; Protein SUPERROOT 2 (At4g31500)		2.331
Cs5g34830		Protein SULFUR DEFICIENCY-INDUCED 1 {ECO:0000303|PubMed:19154231}; AtSDI1 {ECO:0000303|PubMed:19154231} (At5g48850)	2.690	
Cs6g09420	K13424 WRKY transcription factor 33 | (RefSeq) hypothetical protein (A)	Probable WRKY transcription factor 33; WRKY DNA-binding protein 33 (At2g38470)	1.908	1.729
Cs6g19210	K01611 S-adenosylmethionine decarboxylase [EC:4.1.1.50] | (RefSeq) S-adenosylmethionine decarboxylase proenzyme (A)	S-adenosylmethionine decarboxylase proenzyme; AdoMetDC; SAMDC; EC=4.1.1.50; Contains: S-adenosylmethionine decarboxylase alpha chain; Contains: S-adenosylmethionine decarboxylase beta chain; Flags: Precursor (At3g02470)		-1.025
Cs7g08110	K11820 N-hydroxythioamide S-beta-glucosyltransferase [EC:2.4.1.195] | (RefSeq) UDP-glycosyltransferase 74B1 (A)	UDP-glycosyltransferase 74B1; EC=2.4.1.-; N-hydroxythioamide S-beta-glucosyltransferase; EC=2.4.1.195; Thiohydroximate S-glucosyltransferase (At1g24100)	-1.156	-2.258
Cs7g13320	K00547 homocysteine S-methyltransferase [EC:2.1.1.10] | (RefSeq) hypothetical protein (A)	Homocysteine S-methyltransferase 1; BoHMT1; EC=2.1.1.10 (At3g25900)		1.047
Cs7g17600	K00521 ferric-chelate reductase [EC:1.16.1.7] | (RefSeq) ferric reduction oxidase 7, chloroplastic-like (A)	Transcription factor JUNGBRUNNEN 1; NAC domain-containing protein 42; ANAC042 (At2g43000)	1.057	1.759
Cs7g30780	K22920 UTP-glucose-1-phosphate uridylyltransferase [EC:2.7.7.9] | (RefSeq) hypothetical protein (A)	UTPglucose-1-phosphate uridylyltransferase 3, chloroplastic {ECO:0000305}; EC=2.7.7.9 {ECO:0000269|PubMed:19286968}; UDP-glucose pyrophosphorylase 3 {ECO:0000303|PubMed:19286968}; Flags: Precursor (At3g56040)		1.098

Cs8g17360	K20536 mitogen-activated protein kinase 3 [EC:2.7.11.24] | (RefSeq) MAPK1, MAP; mitogen-activated protein kinase 1 (A)	Mitogen-activated protein kinase 3; AtMPK3; MAP kinase 3; EC=2.7.11.24 (At3g45640)		1.145
Cs9g05430	K01611 S-adenosylmethionine decarboxylase [EC:4.1.1.50] | (RefSeq) S-adenosylmethionine decarboxylase proenzyme 4 (A)	S-adenosylmethionine decarboxylase proenzyme 4 {ECO:0000305}; AdoMetDC4 {ECO:0000305}; EC=4.1.1.50 {ECO:0000269|PubMed:16699540}; Contains: S-adenosylmethionine decarboxylase 1 alpha chain {ECO:0000250|UniProtKB:P17707}; Contains: S-adenosylmethionine decarboxylase 1 beta chain {ECO:0000250|UniProtKB:P17707}; Protein BUSHY AND DWARF 2 {ECO:0000303|PubMed:16699540}; Flags: Precursor (At5g18930)		-1.207
Cs9g07420	K00547 homocysteine S-methyltransferase [EC:2.1.1.10] | (RefSeq) homocysteine S-methyltransferase 3 (A)	Homocysteine S-methyltransferase 3; EC=2.1.1.10; S-methylmethionine:homocysteine methyltransferase 3; AtHMT-3; SMM:Hcy S-methyltransferase 3 (At3g22740)	1.233	
Cs9g07780	K13811 3'-phosphoadenosine 5'-phosphosulfate synthase [EC:2.7.7.4 2.7.1.25] | (RefSeq) hypothetical protein (A)	ATP sulfurylase 1, chloroplastic; AtPS1; EC=2.7.7.4; Flags: Precursor (At3g22890)	-1.997	-1.224
orange1.1t02087	K20617 cytochrome P450 family 71 subfamily A | (RefSeq) cytochrome P450 71A1-like (A)	Cytochrome P450 83B1; EC=1.14.14.45 {ECO:0000269|PubMed:12970475}; Protein ALTERED TRYPTOPHAN REGULATION 4; Protein RED ELONGATED 1; Protein SUPERROOT 2 (At4g31500)		-2.078
orange1.1t02662	K20604 mitogen-activated protein kinase kinase 9 [EC:2.7.12.2] | (RefSeq) mitogen-activated protein kinase kinase 9 (A)	Mitogen-activated protein kinase kinase 9; AtMKK9; MAP kinase kinase 9; EC=2.7.12.2 (At1g73500)	-1.280	-1.159
orange1.1t02796	K20617 cytochrome P450 family 71 subfamily A | (RefSeq) cytochrome P450 71A1-like (A)	Cytochrome P450 83B1; EC=1.14.14.45 {ECO:0000269|PubMed:12970475}; Protein ALTERED TRYPTOPHAN REGULATION 4; Protein RED ELONGATED 1; Protein SUPERROOT 2 (At4g31500)	2.393	1.485
orange1.1t05897	K01012 biotin synthase [EC:2.8.1.6] | (RefSeq) biotin synthase-like (A)	Biotin synthase, mitochondrial; EC=2.8.1.6; Flags: Precursor (At2g43360)		-2.375
Sulfur compound catabolic process [GO:0044273; P = 0.2116 (P5CR vs. P5R)]				
Cs1g15710	K05681 ATP-binding cassette, subfamily G (WHITE), member 2 | (RefSeq) hypothetical protein (A)	ABC transporter G family member 36; ABC transporter ABCG.36; AtABCG36; Pleiotropic drug resistance protein 8; Protein PENETRATION 3 (At1g59870)		-2.119
Cs2g19240	K20285 Rab9 effector protein with kelch motifs | (RefSeq)	Nitrile-specifier protein 5; AtNSP5 (At3g07720)		1.201

	nitrile-specifier protein 5-like (A)			
Cs4g13090	K18592 gamma-glutamyltranspeptidase / glutathione hydrolase / leukotriene-C4 hydrolase [EC:2.3.2.2 3.4.19.13 3.4.19.14] | (RefSeq) gamma-glutamyltranspeptidase 3-like (A)	Glutathione hydrolase 3; EC=3.4.19.13; Gamma-glutamyltransferase 3; Gamma-glutamyltranspeptidase 3; EC=2.3.2.2; Gamma-glutamyltranspeptidase 4 (At4g29210)		1.227
Cs5g01510	K01761 methionine-gamma-lyase [EC:4.4.1.11] | (RefSeq) methionine gamma-lyase (A)	Methionine gamma-lyase; AtMGL; EC=4.4.1.11; L-methioninase (At1g64660)		-1.687
Ascorbate and aldarate metabolism [ko00053; P = 0.3733 (P3CR vs. P3R) and 0.1687 (P5CR vs. P5R)]				
Cs1g01800	K00423 L-ascorbate oxidase [EC:1.10.3.3] | (RefSeq) L-ascorbate oxidase-like (A)	L-ascorbate oxidase; ASO; Ascorbase; EC=1.10.3.3; Flags: Precursor (At4g39830)		1.402
Cs1g24850	K00128 aldehyde dehydrogenase (NAD+) [EC:1.2.1.3] | (RefSeq) aldehyde dehydrogenase family 3 member F1 (A)	Aldehyde dehydrogenase family 3 member F1; EC=1.2.1.3 (At4g36250)	1.544	
Cs1g25730	K00434 L-ascorbate peroxidase [EC:1.11.1.11] | (RefSeq) hypothetical protein (A)	L-ascorbate peroxidase 3; AtAPx03; EC=1.11.1.11 (At4g35000)		-1.050
Cs2g23200	K17744 L-galactose dehydrogenase [EC:1.1.1.316] | (RefSeq) hypothetical protein (A)	Probable voltage-gated potassium channel subunit beta; K(+) channel subunit beta; Potassium voltage beta 1; KV-beta1 (At1g04690)	-1.602	
Cs3g14390	K00128 aldehyde dehydrogenase (NAD+) [EC:1.2.1.3] | (RefSeq) aldehyde dehydrogenase 5, mitochondrial-like (A)	Translationally-controlled tumor protein homolog; TCTP (At3g16640)		-2.145
Cs3g17910	K00434 L-ascorbate peroxidase [EC:1.11.1.11] | (RefSeq) thylakoid lumenal 29 kDa protein, chloroplastic (A)	Thylakoid lumenal 29 kDa protein, chloroplastic; TL29; EC=1.-.-.-; LeAPx09; P29; Flags: Precursor (At4g09010)		-1.085
Cs4g13120	K00469 inositol oxygenase [EC:1.13.99.1] | (RefSeq) hypothetical protein (A)	Inositol oxygenase 2; EC=1.13.99.1; Myo-inositol oxygenase 2; AtMIOX2; MI oxygenase 2 (At4g26260)		-1.533
Cs4g19710	K00103 L-gulonolactone oxidase [EC:1.1.3.8] | (RefSeq) L-gulonolactone oxidase 3 (A)	L-gulonolactone oxidase 3 {ECO:0000303|PubMed:20622436}; AtGulLO3 {ECO:0000303|PubMed:20622436}; EC=1.1.3.8 {ECO:0000269|PubMed:20622436}; Flags: Precursor (At5g11540)		-1.415
Cs5g24640	K00699 glucuronosyltransferase [EC:2.4.1.17] | (RefSeq) UDP-glycosyltransferase 76C4-like (A)	UDP-glycosyltransferase 76F1; EC=2.4.1.- (At3g55700)	2.193	-1.003
Cs6g04140	K00434 L-ascorbate peroxidase [EC:1.11.1.11] | (RefSeq) hypothetical protein (A)	L-ascorbate peroxidase 2, cytosolic; EC=1.11.1.11; L-ascorbate peroxidase 1b; APX1b; AtAPx02 (At3g09640)	1.147	
Cs6g17580	K14190 GDP-L-galactose phosphorylase [EC:2.7.7.69] | (RefSeq) hypothetical protein (A)	GDP-L-galactose phosphorylase 1; EC=2.7.7.69; Protein VITAMIN C DEFECTIVE 2 (At5g55120)	1.157	2.069
Cs7g05430	K00434 L-ascorbate peroxidase [EC:1.11.1.11] | (RefSeq) putative	Putative L-ascorbate peroxidase 6; AtAPx08; EC=1.11.1.11		1.117

	L-ascorbate peroxidase 6 (A)	(At4g32320)		
Cs7g32210	K00699 glucuronosyltransferase [EC:2.4.1.17] | (RefSeq) UDP-glycosyltransferase 76C4-like (A)	UDP-glycosyltransferase 76F1; EC=2.4.1.- (At3g55700)		-2.226
Cs7g32220	K00699 glucuronosyltransferase [EC:2.4.1.17] | (RefSeq) UDP-glycosyltransferase 76G1-like (A)	UDP-glycosyltransferase 76B1 {ECO:0000305}; EC=2.4.1.- {ECO:0000305} (At3g11340)		-2.117
Cs8g18300	K00699 glucuronosyltransferase [EC:2.4.1.17] | (RefSeq) UDP-glycosyltransferase 76G1-like (A)	UDP-glycosyltransferase 76B1 {ECO:0000305}; EC=2.4.1.- {ECO:0000305} (At3g11340)	-1.264	-1.334
Cs8g18310	K00699 glucuronosyltransferase [EC:2.4.1.17] | (RefSeq) UDP-glycosyltransferase 76G1-like (A)	UDP-glycosyltransferase 76B1 {ECO:0000305}; EC=2.4.1.- {ECO:0000305} (At3g11340)	-1.040	-3.150
L-Ascorbic acid biosynthetic process [GO:0019853; P = 0.7603 (P3CR vs. P3R) and 0.4020 (P5CR vs. P5R)]				
Cs4g13120	K00469 inositol oxygenase [EC:1.13.99.1] | (RefSeq) hypothetical protein (A)	Inositol oxygenase 2; EC=1.13.99.1; Myo-inositol oxygenase 2; AtMIOX2; MI oxygenase 2 (At4g26260)		-1.533
Cs4g19710	K00103 L-gulonolactone oxidase [EC:1.1.3.8] | (RefSeq) L-gulonolactone oxidase 3 (A)	L-gulonolactone oxidase 3 {ECO:0000303|PubMed:20622436}; AtGulLO3 {ECO:0000303|PubMed:20622436}; EC=1.1.3.8 {ECO:0000269|PubMed:20622436}; Flags: Precursor (At5g11540)		-1.415
Cs6g17580	K14190 GDP-L-galactose phosphorylase [EC:2.7.7.69] | (RefSeq) hypothetical protein (A)	GDP-L-galactose phosphorylase 1; EC=2.7.7.69; Protein VITAMIN C DEFECTIVE 2 (At5g55120)	1.157	2.069
**Index**	**Compounds**			
			P3CR vs. P3R	P5CR vs. P5R
Glutathione metabolism [ko00480; P = 0.4275 (P3CR vs. P3R) and 0.0247 (P5CR vs. P5R)]				
mws4134	Oxiglutatione		-3.379	-1.013
pme0014	L-Glutamic acid			1.320
pme0195	L-Cysteine			-1.032
pme1086	Glutathione reduced form		-5.975	
pme1841	Cadaverine			-1.674
pme2292	Putrescine		1.171	
pme2651	NADP (Nicotinamide adenine dinucleotide phosphate)		-1.247	
Sulfur metabolism [ko00920; P = 0.8985 (P3CR vs. P3R) and 0.1450 (P5CR vs. P5R)]				
mws0671	L-Homoserine		2.706	2.457

pme0195	L-Cysteine			-1.032
Cysteine and methionine metabolism [ko00270; P = 0.1498 (P3CR vs. P3R) and 0.2677 (P5CR vs. P5R)]				
MWS0631	S-Sulfo-L-cysteine			-2.103
mws0671	L-Homoserine		2.706	2.457
mws1375	Nicotianamine		-3.369	
mws4052	1-Aminocyclopropane-1-carboxylic acid		-1.630	
MWStz103	S-(5'-Adenosyl)-L-methionine		-2.702	
pme0195	L-Cysteine			-1.032
pme1086	Glutathione reduced form		-5.975	
pme1210	L-Methionine		-1.208	
pme1474	5'-Deoxy-5'-(methylthio)adenosine		-1.213	
Ascorbate and aldarate metabolism [ko00053; P = 0.7733 (P3CR vs. P3R)]				
Lmbn000198	3-Dehydro-L-Threonic Acid			
pmb2922	Uridine 5'-diphospho-D-glucose		-4.189	
Zmpn000199	D-Galactaric acid		2.025	
Zmyn000108	D-Saccharic acid		-1.701	
