# Supplementary material for: Mechanisms by Which Increased pH Ameliorates Copper Excess in Citrus sinensis Roots: Insight from a Combined Analysis of Physiology, Transcriptome, and Metabolome
Source: Plants (Basel). 2024 Oct 31;13(21):3054. doi: 10.3390/plants13213054 (PMC11548300; doi:10.3390/plants13213054)
Supplement: Supplementary file 1 [file plants-13-03054-s001.zip › Table S23SecondRevision.pdf]

Table S23. Specific primer pairs used for qRT-PCR expression analysis

ID	Description	Forward primers (5′→3′)
Cs2g27180	*Glucuronokinase 1*	AAACGAGGTTGGGGAAGAAAA
Cs3g18420	*TPR repeat-containing thioredoxin TTL1*	CAGGTTACGCAGGGCAGACT
Cs6g04290	*Probable E3 ubiquitin ligase SUD1*	CTCGCCTCTACGACAGCAACA
Cs9g06160	*Probable aspartic proteinase GIP2*	GGGTTGATTGCGAGAAAGGTT
Cs1g19393	*Defensin-like protein 6*	TTCTTTGGGATTTTCTTGCTGC
Cs2g14920	*Probable xyloglucan endotransglucosylase/hydrolase protein 5*	ATGGTTCCTGGTGATTCTGCC
Cs4g05470	*AAI domain-containing protein*	GTGGGTTGGTTCACATTGGTCT
Cs6g11700	*Aquaporin PIP2-2*	CAAAAGCCAGACGGACGCC
Cs1g21430	*Calcium-transporting ATPase 2*	AGGAGGCTTACGTGCCCATAG
Cs2g10140	*Berberine bridge enzyme-like 18*	TTCAGATTCCGCAACTCCATTC
Cs3g22470	*Leucine-rich repeat receptor-like tyrosine-protein kinase PXC3*	TTCAAACCAAACAAGCACCATG
Cs4g02690	*EG45-like domain containing protein*	CTCCTCCTTATGTCCCCTCTGC
Cs1g19930	*LanC-like protein GCL1*	TAACAACAGCGAACGCCACG
Cs2g24740	*Kelch repeat-containing protein*	TTGTTGGTGGTCAAGAGGGTG
Cs3g20810	*Alcohol dehydrogenase*	CAAAACCTGCAAAAGGCTCAA
Cs4g15770	*Polyol transporter 5*	TATGTGCTGCCGTCTCGTTCT
Cs1g15710	*ABC transporter G family member 36*	TTGAAAGTTGTGGGTTCTGCTG
Cs3g13930	*Probable linoleate 9S-lipoxygenase 5*	TTTACCCCGCGAAAGACTACA
Cs4g09780	*Glucan endo-1,3-beta-glucosidase 13*	GCAGACCCGAAGAACACGAC
Cs5g10550	*Thaumatin-like protein*	GCCGTGCCCCGTGTTCAA
Cs1g13290	--	AACTTGGAAACGAACGACTGGG
Cs2g31160	--	GCGGTTGTGAAGAAGGTTGATG
Cs5g09060	*14 kDa proline-rich protein DC2.15*	AATCCCAAACCCTAACCCAAAT
Cs6g21260	*Gibberellin-regulated protein 4*	GGTCGTGGCAGCAAATGG
Cs1g13730	*Pleiotropic drug resistance protein 1*	TCTGTTCATCGGCGTTCAATAC

Cs3g15140	*Nicotianamine synthase*	TGGTGGGGATGGACAAGGA	CGAACCCGAGAAGATCAGAAGG
Cs4g10860	*Disease resistance protein*	AGGGTGAAGGAATGTGGGAAAT	ATGGGCGGTGGTGGGATA
Cs5g04630	*Germin-like protein subfamily T member 2*	AGAGGAACGGTGCTCGTAGGT	TCATTGGGAACGGGAGGC
Cs1g01540	*Annexin D2*	AGGCACAGATAAATGCGACCC	CCCATTCGTCTGTCCCCAAC
Cs3g18090	*7-deoxyloganetin glucosyltransferase*	ACCCGAGTTTGTGACGGAGAC	CCACCCACTGTGCGTTAAGAAT
Cs5g32930	*Translocator protein homolog*	GGCTTCCACAAGAAACCCACG	CCAAACCAAAGACCCAGACAAA
Cs6g21520	*NAC domain-containing protein 100*	TATTCTGCCTACGACCACCCC	TGAGCCTAATTTCACCATCCCT
Cg1g026080	*Actin*	AGAACTATGAACTGCCTGATGGC	GCTTGGAGCAAGTGCTGTGATT
Cg7g019550	*U4/U6 small muclear ribonucleoprotiein PRP31 (PRPF31)*	ACTCATGGGAACGGCTGGTGGTC	TCGGCAGGCACGCATCCTTAGAG
