# Supplementary material for: Mechanisms by Which Increased pH Ameliorates Copper Excess in Citrus sinensis Roots: Insight from a Combined Analysis of Physiology, Transcriptome, and Metabolome
Source: Plants (Basel). 2024 Oct 31;13(21):3054. doi: 10.3390/plants13213054 (PMC11548300; doi:10.3390/plants13213054)
Supplement: Supplementary file 1 [file plants-13-03054-s001.zip › Table S8SecondRevision.pdf]

Table S8. List of DAMs detected in P3R vs. P5R, P3CR vs. P3R, P5CR vs. P5R, and/or P3CR vs. P5CR

Index (Formula)	Compounds	Log_2(fold change)				Class
		P3R vs. P5R	P3CR vs. P3R	P5CR vs. P5R	P3CR vs P5CR	
**Amino acids and amino acid derivatives (AADs)**						
AADs						
Lmbp000123(C6H13NO2S)	L-Homomethionine		-1.720		-2.210	PM
Lmhp001670(C11H22N2O3)	L-Valyl-L-leucine	-1.740	-1.392	-2.517		PM
Lmhp002001(C14H20N2O3)	L-Valyl-L-phenylalanine	-1.822	-1.306	-2.750		PM
Lmqp000427(C6H11NO3)	N-Methyl-trans-4-hydroxy-L-proline				-1.547	PM
Lmrj001698(C9H18N2O4)	L-Seryl-L-Isoleucine	-1.785	-1.294	-2.704		PM
Lmrj002087(C10H18N2O5)	L-Isoleucyl-L-aspartate		-1.698		-1.853	PM
mws0227(C6H13NO2)	L-Leucine				-1.712	PM
mws0230(C4H9NO3)	L-Threonine			1.615		PM
mws0254(C6H9N3O2)	L-Histidine			-1.003		PM
mws0258(C6H13NO2)	L-Isoleucine				-1.734	PM
mws0520(C11H13NO4)	N-Acetyl-L-tyrosine		2.674		1.481	PM
MWS0631(C3H7NO5S2)	S-Sulfo-L-cysteine	-1.768		-2.103		PM
mws0671(C4H9NO3)	L-Homoserine	1.760	2.706	2.457	2.009	PM
mws0715(C13H16N2O4)	Phenylacetyl-L-glutamine	-1.191				PM
mws0736(C8H16N2O3)	N-Glycyl-L-leucine	-1.395		-1.643		PM
mws1051(C15H23N6O5S)	S-Adenosylmethionine		-11.470		-10.647	PM
mws1550(C6H11NO2S)	S-Allyl-L-cysteine			1.296	-1.237	PM
mws1587(C6H13NO2)	L-Norleucine		-1.105		-1.832	PM
mws4134(C20H32N6O12S2)	Oxiglutatione		-3.379	-1.013	-2.172	PM
mws4176(C12H16N2O3)	L-Alanyl-L-phenylalanine	-1.340		-2.075		PM
MWS4296(C11H14N2O3)	Glycylphenylalanine	-1.554	-2.467	-2.638	-1.383	PM
mws5035(C15H22N2O3)	L-Leucyl-L-phenylalanine	-1.029		-2.116		PM
MWSmce056(C11H12N2O2)	DL-Tryptophan		1.466		1.211	PM
MWSmce251(C16H15NO4)	N-Benzoyl-(2R,3S)-3-phenylisoserine		-1.050			PM
MWStz103(C15H22N6O5S)	S-(5'-Adenosyl)-L-methionine		-2.702		-3.479	PM
pmb2591(C13H14N2O3)	N-Acetyl-L-tryptophan		2.102		1.615	PM
pmb2673(C9H9NO5)	6-Hydroxydopaquinone		-2.093		-2.342	PM
pmb2857(C11H19NO9)	L-Glutamic acid-O-glycoside	-1.304				PM
pme0014(C5H9NO4)	L-Glutamic acid			1.320	-2.315	PM
pme0026(C6H14N2O2)	L-Lysine		-1.155		-1.408	PM
pme0122(C8H16N2O3)	N6-Acetyl-L-lysine		-1.971		-2.360	PM
pme0137(C7H12N2O4)	N-Acetyl-L-glutamine				1.086	PM
pme0193(C5H10N2O3)	L-Glutamine		-1.155		-1.384	PM
pme0195(C3H7NO2S)	L-Cysteine			-1.032	1.093	PM
pme1086(C10H17N3O6S)	Glutathione reduced form	2.502	-5.975		-4.398	PM
pme1210(C5H11NO2S)	L-Methionine		-1.208		-1.649	PM
pme1419(C6H13NO2S)	L-Methionine methyl ester		-11.321		-11.333	PM
pme2527(C5H12N2O2)	L-Ornithine				-1.501	PM
pme2617(C5H11NO3S)	L-Methionine sulfoxide				-1.267	PM
pme2758(C5H9NO5)	4-Hydroxy-L-glutamic acid				-1.052	PM
pme2914(C6H10O5)	3-Hydroxy-3-methylpentane-1,5-dioic acid		-1.397		-2.226	PM
pme3033(C4H9NO2)	N,N-Dimethylglycine			1.166		PM
Zmdp001647(C10H18N2O5)	γ-Glutamyl-L-valine				-1.098	PM
Zmdp001857(C14H18N2O6)	γ-Glutamyltyrosine		-1.699			PM
**Organic acids (OAs)**						

OAs						
Lmbn002072(C7H12O4)	2-Propylsuccinic acid		-1.742		-2.173	PM
Lmmn000806(C5H8O4)	Dimethylmalonic acid				-1.172	PM
Lmmn002164(C5H8O4)	Monomethyl succinate				-1.327	PM
Lmmn003323(C16H32O3)	2-Hydroxyhexadecanoic acid		5.256	1.006	3.897	PM
Lmqp000873(C4H4O3)	Succinic anhydride		-1.174		-1.631	PM
mws0147(C5H10O3)	β-Hydroxyisovaleric acid				-1.332	PM
mws0192(C4H6O4)	Succinic acid				-2.231	PM
mws0208(C6H10O4)	Adipic acid				-1.049	PM
mws0242(C8H14O4)	Suberic acid			1.228		PM
mws0277(C7H12O6)	Quinic acid		-2.326		-3.038	PM
mws0425(C5H6O4)	Citraconic acid				-1.321	PM
mws0470(C4H6O4)	Methylmalonic acid				-1.522	PM
mws0473(C5H8O4)	2-Methylsuccinic acid				-1.369	PM
mws0576(C4H8O3)	3-Hydroxybutyric acid		-2.669		-2.798	PM
mws0601(C5H5NO2)	Pyrrole-2-carboxylic acid		1.251			PM
mws0924(C6H10O4)	2-Methylglutaric acid				-1.183	PM
MWS1912(C13H22O3)	Methyl dihydrojasmonate		1.469		1.258	PM
MWS2073(C8H6Cl2O3)	2,4-Dichlorophenoxyacetic acid		1.428		1.791	PM
mws4052(C4H7NO2)	1-Aminocyclopropane-1-carboxylic acid		-1.630		-2.181	PM
MWS4396(C11H8O3)	1-Hydroxy-2-Naphthoate		4.040	1.494	2.238	PM
pmb2657(C10H18N4O6)	Argininosuccinic acid				-1.162	PM
pmb3101(C7H12O5)	2-Isopropylmalic acid				-2.372	PM
pme0295(C6H11NO3)	4-Acetamidobutyric acid			1.215	-1.325	PM
pme1216(C6H5NO2)	2-Picolinic acid		1.046			PM
pme1654(C12H18O3)	Jasmonic acid		-1.343		-2.157	PM
pme3011(C4H9NO2)	γ-Aminobutyric acid			1.241		PM
pme3096(C3H5NO4)	Aminomalonic acid				-1.520	PM
Wmzn000227(C6H10O4)	2,2-Dimethylsuccinic acid			1.363		PM
Zmgn004894(C8H8O3)	Methyl 4-hydroxybenzoate		1.242			PM
Zmjn001813(C7H12O4)	Pimelic acid		-1.730		-2.150	PM
Zmyn000453(C6H8O7)	Isocitric acid		-2.158		-2.800	PM
Zmyn002323(C8H8O3)	2-Hydroxyphenylacetic acid		-10.564		-10.876	PM
**Lipids**						
Glycerol ester						
Lmhp008744(C33H56O14)	1-α-Linolenoyl-glycerol-2,3-di-O-glucoside			-1.734		PM
Lmhp009773(C27H46O9)	1-α-Linolenoyl-glycerol-3-O-glucoside			-1.037		PM
Lmhp011388(C21H36O4)	2-α-Linolenoyl-glycerol			-1.356		PM
Lmhp011562(C21H36O4)	1-α-Linolenoyl-glycerol			-1.422		PM
Lmhp012042(C21H38O4)	2-Linoleoylglycerol			-1.523		PM
Lmhp112042(C21H38O4)	1-Linoleoylglycerol			-1.455		PM
Lmxp010913(C35H66NO9P)	1-(9Z-Octadecenoyl)-2-(9-oxo-nonanoyl)-sn-glycero-3-phosphocholine		9.169	11.137	-1.968	PM
pmb0296(C21H40O4)	1-Oleoyl-Sn-Glycerol		-1.135	-1.237		PM
Lysophosphatidylcholine (LysoPC)						
Lmhp009890(C28H52NO7P)	LysoPC 20:3	-2.819	-3.151	-5.756		PM
pma1303(C24H46NO7P)	LysoPC 16:2			1.541		PM
pmb0854(C26H48NO7P)	LysoPC 18:3		-1.070		-1.893	PM
pmb0863(C24H46NO7P)	LysoPC 16:2(2n isomer)		1.801	1.943		PM
pmb0865(C26H48NO7P)	LysoPC 18:3(2n isomer)		1.586	1.482		PM
pmb2406(C25H52NO7P)	LysoPC 17:0		-1.278		-1.248	PM
pmc0960(C28H50NO7P)	LysoPC 20:4	-2.415		-4.966	2.011	PM

pmd0130(C22H46NO7P)	LysoPC 14:0		1.488	1.296		PM
pmd0136(C26H54NO7P)	LysoPC 18:0(2n isomer)		1.653			PM
pmd0143(C28H58NO7P)	LysoPC 20:0		-1.296		-1.688	PM
pmd0147(C28H54NO7P)	LysoPC 20:2		-1.441		-1.709	PM
pmp001251(C26H50NO7P)	LysoPC 18:2(2n isomer)		1.975	1.431		PM
pmp001270(C24H48NO7P)	LysoPC 16:1	-2.606	-11.829	-2.756	-11.679	PM
pmp001273(C26H50NO7P)	LysoPC 18:2				-1.238	PM
pmp001281(C26H52NO7P)	LysoPC 18:1		-1.644		-1.828	PM
Lysophosphatidylethanolamine (LysoPE)						
pmb0856(C23H46NO7P)	LysoPE 18:1(2n isomer)		1.089			PM
pmb0864(C19H40NO7P)	LysoPE 14:0				-1.473	PM
pmb0880(C23H48NO7P)	LysoPE 18:0(2n isomer)		-1.392		-1.643	PM
pmb0881(C23H44NO7P)	LysoPE 18:2		-1.235		-1.777	PM
pmd0160(C21H44NO7P)	LysoPE 16:0(2n isomer)				-1.428	PM
Free fatty acids (FFAs)						
Hmqn003054(C18H34O5)	9,10,11-Trihydroxy-12-octadecenoic acid		1.402		1.469	PM
Lmbn003970(C18H34O5)	9,12,13-TriHOME; 9(S),12(S),13(S)-Trihydroxy-10(E)-octadecenoic acid	-1.066		-1.516		PM
Lmbn004240(C18H34O5)	9,10-Dihydroxy-12,13-epoxyoctadecanoic acid		2.513		2.640	PM
Lmbn005487(C18H34O4)	12,13-DHOME; (9Z)-12,13-Dihydroxyoctadec-9-enoic acid		1.087			PM
Lmbn005662(C18H32O3)	9(10)-EpOME;(9R,10S)-(12Z)-9,10-Epoxyoctadecenoic acid		1.516		1.179	PM
Lmbn005822(C18H34O4)	9,10-DHOME; (12Z)-9,10-Dihydroxyoctadec-12-enoic acid		-1.734			PM
Lmbn005923(C18H30O2)	Crepenynic acid		-1.095	-1.237		PM
Lmcn009122(C16H30O2)	(7Z)-Hexadecenoic acid	-1.664	-2.453	-1.808		PM
Lmcn009539(C19H34O2)	E,E,Z-1,3,12-Nonadecatriene-5,14-diol		1.386		1.762	PM
Lmxn015337(C17H34O2)	Methyl palmitate		1.200		1.506	PM
mws0367(C18H30O2)	α-Linolenic acid			-1.054		PM
mws0369(C20H32O2)	Arachidonic acid	-2.770	-1.534	-6.057		PM
MWS2430(C15H30O2)	13-methylmyristic acid		1.930		1.734	PM
mws5045(C12H24O3)	12-Hydroxydodecanoic acid		1.049			PM
MWS80006(C18H32O4)	9-Hydroperoxy-9Z,11E-Octadecadienoic acid		3.315		2.056	PM
MWSmce549(C17H34O4)	1-Monomyristin			1.090		PM
MWSmce689(C19H32O2)	Methyl linolenate		2.403	-1.611	4.084	PM
pmb0889(C18H30O2)	Punicic acid (9Z,11E,13Z-octadecatrienoic acid)				1.041	PM
pmb2787(C18H30O3)	9-Oxo-10E,12Z-octadecadienoic acid			-1.607		PM
pmb2791(C18H30O4)	9-Hydroperoxy-10E,12,15Z-octadecatrienoic acid	-1.449		-3.049		PM
pmb2804(C18H32O4)	13S-Hydroperoxy-9Z,11E-octadecadienoic acid		3.012		2.788	PM
pmn001689(C18H32O4)	9-Hydroxy-12-oxo-15(Z)-octadecenoic acid	-2.255		-2.539		PM
pmn001691(C18H32O5)	9,12,13-Trihydroxy-10,15-octadecadienoic acid	-1.780		-2.604		PM

pmn001694(C18H34O5)	9,10,13-Trihydroxy-11-Octadecenoic acid	-1.122		-1.507		PM
pmp001276(C33H56O14)	1-Linolenoyl-rac-glycerol-diglucoside		-11.491		-11.157	PM
YC512118(C18H35NO)	Oleamide (9-Octadecenamide)			1.046		PM
Zmpn003368(C18H30O3)	13S-Hydroxy-9Z,11E,15Z-octadecatrienoic acid			-1.050		PM
Zmyn004449(C18H30O4)	9-Hydroxy-12-oxo-10(E),15(Z)-octadecadienoic acid		3.009		2.658	PM
Zmyn004676(C18H30O3)	17-Hydroxylinolenic acid		1.895		2.106	PM
Zmyn004732(C18H30O3)	2R-hydroxy-9Z,12Z,15Z-octadecatrienoic acid		3.560		3.448	PM
Zmyn005252(C17H34O3)	3-Hydroxy-palmitic acid methyl ester		5.395	1.233	4.456	PM
Zmyn005384(C18H36O3)	2R-Hydroxyoctadecanoic acid		4.754	1.057	3.537	PM
**Nucleotides and derivatives (NDs)**						
NDs						
Cmdn000784(C5H4N4O3)	Uric acid	-1.122				PM
Lmqn000780(C17H27N3O17P2)	Uridine 5'-diphospho-N-acetylglucosamine		-3.232		-2.469	PM
Lmqp000329(C8H14N3O7P)	5-Aminoimidazole ribonucleotide		1.594		1.190	PM
ML10180524(C9H13N3O5)	Cytarabine	-1.585		-2.478		PM
mws0248(C9H12N2O6)	Uridine	-1.207	-1.506	-3.211		PM
mws0609(C10H12N5O7P)	Guanosine 3',5'-cyclic monophosphate		-1.036		-1.411	PM
mws0668(C10H12N4O6)	Xanthosine		-2.360		-2.404	PM
mws0884(C10H12N5O6P)	Cyclic 3',5'-adenylic acid	-2.031	-4.634	-4.142	-2.523	PM
mws1060(C10H12N4O5)	9-(Arabinosyl)hypoxanthine		-2.277		-2.540	PM
mws1409(C15H22N2O18P2)	Uridine-5′-diphosphoglucuronic acid	1.054	-11.774	1.132	-11.852	PM
mws1715(C10H13N5O3)	Cordycepin (3'-Deoxyadenosine)		-10.297		-10.012	PM
MWS5083(C17H21N4O9P)	Flavin single nucleotide (FMN)		-1.351		-1.840	PM
MWSmce116(C10H13N5O3)	5'-Deoxyadenosine			1.252	-1.282	PM
MWSmce333(C10H13N5O5)	Crotonoside		-2.452	-2.617		PM
pmb0374(C5H5N5)	2-Aminopurine	-1.884	-2.086	-3.296		PM
pmb0530(C21H27N7O14P2)	Nicotinic acid adenine dinucleotide		-4.606		-4.737	PM
pmb0532(C10H13N4O8P)	Inosine 5'-monophosphate		-2.111		-1.745	PM
pmb0981(C10H14N5O7P)	Adenosine 5'-monophosphate		-1.974		-1.300	PM
pmb0998(C10H14N5O8P)	Guanosine 5'-monophosphate		-2.056		-1.946	PM
pmb2507(C5H11O7P)	2-Deoxyribose-1-phosphate		-1.659		-2.314	PM
pmb2922(C15H24N2O17P2)	Uridine 5'-diphospho-D-glucose	1.460	-4.189			PM
pmc0066(C10H13N4O7P)	2'-Deoxyinosine-5'-monophosphate			-1.115	1.354	PM
pmc0281(C15H21N5O8)	Ribosyladenosine				-1.102	PM
pme0183(C5H5N5O)	Isoguanine	-1.211		-1.536		PM
pme0230(C10H13N5O4)	Adenosine	-1.937	-2.316	-3.720		PM
pme0256(C5H4N4O2)	Xanthine	-1.015				PM
pme0257(C4H4N2O2)	Uracil				1.220	PM
pme0264(C10H14N2O5)	Thymidine		-1.715		-2.030	PM
pme1173(C5H4N4O)	Allopurinol			-1.025		PM
pme1178(C10H13N5O5)	Guanosine		-4.025	-2.007	-2.367	PM
pme1184(C10H13N5O4)	2'-Deoxyguanosine		-3.568		-3.320	PM
pme1474(C11H15N5O3S)	5'-Deoxy-5'-(methylthio)adenosine		-1.213		-1.488	PM
pme2117(C10H15N5O10P2)	Adenosine 5'-diphosphate		-1.571		-1.338	PM
pme2651(C21H28N7O17P3)	NADP (Nicotinamide adenine dinucleotide phosphate)		-1.247		-1.570	PM
pme2746(C27H33N9O15P2)	Riboflavin 5'-adenosine		-1.572	1.076	-2.845	PM

	diphosphate					
pme2776(C10H12N4O4)	2'-Deoxyinosine		-1.918		-1.533	PM
pme3007(C9H14N2O12P2)	Uridine 5'-diphosphate			1.761	-2.102	PM
pme3188(C9H13N2O9P)	Uridine 5'-monophosphate		-1.433		-1.480	PM
pme3732(C9H13N3O5)	Cytidine	-1.211		-2.304		PM
pme3961(C10H13N5O3)	2'-Deoxyadenosine		-1.721		-1.519	PM
pme3967(C12H17N5O5)	2-(Dimethylamino)guanosine				-1.046	PM
Zmfn000481(C14H22N2O16P2)	Uridine-5'-diphosphate-D-xylose	1.036	-3.460		-3.067	PM
**Phenolic acids (PAs)**						
PAs						
Cmjn004337(C18H26O10)	Benzyl-(2''-O-xylosyl)glucoside		3.967		2.736	SM
Cmqn010367(C21H32O2)	3-(8Z,11E)-8,11-Pentadecadienyl-1,2-benzenediol		6.347	2.817	3.249	SM
Cmsp002787(C11H14O5)	3,4'-Dihydroxy-3',5'-dimethoxypropiophenone	1.038	-1.383			SM
Cmyp005062(C20H22O9)	Salireposide	-1.149		-1.585		SM
Cmzp002057(C14H18O8)	6-O-Acetylarbutin				-1.373	SM
HJN003(C17H22O10)	1-O-Sinapoyl-D-glucose		-1.239	1.036	-1.913	SM
Hmbn002692(C22H30O14)	6'-O-Feruloyl-D-sucrose		1.430			SM
Hmgn001653(C7H6O3)	Protocatechualdehyde		1.981		1.227	SM
Hmhn003067(C15H18O8)	Phenylpropionic acid-O-β-D-glucopyranoside		-1.248			SM
Hmln000873(C13H16O10)	2-O-Galloyl-D-glucose				-1.173	SM
Hmln002806(C16H16O8)	5-O-Caffeoylshikimic acid		1.260	2.066		SM
Hmln003529(C18H26O10)	Benzyl β-primeveroside		11.486		11.486	SM
Hmmn002544(C16H20O9)	Ferulic acid-4-O-glucoside		-1.507		-2.175	SM
Hmqp002567(C17H17NO3)	P-Hydroxycinnamic acid p-hydroxyphenethylamine		6.729	3.231	2.952	SM
Hmtn001302(C13H16O8)	Glucosyloxybenzoic acid		15.339		15.339	SM
Lmbn004790(C11H14O4)	Methyl 3-(3-hydroxy-4-methoxyphenyl)propanoate		3.237		2.858	SM
Lmgn001670(C7H6O3)	Salicylic acid		1.882		1.252	SM
Lmhn003373(C11H10O7)	p-Hydroxybenzoylmalic acid			1.422		SM
Lmlp002765(C15H16O2)	Stilbostemin B			1.157	-1.211	SM
Lmmn000774(C15H20O9)	Dihydrocaffeoylglucose		1.227	1.267		SM
Lmmn001643(C9H8O3)	2-Hydroxycinnamic acid		1.044			SM
Lmmn002179(C14H18O8)	Methyl salicylate-2-O-glucoside				-1.034	SM
Lmmn003663(C14H18O9)	5-Glucosyloxy-2-hydroxybenzoic acid methyl ester	-2.181	2.612			SM
Lmqp008175(C10H10O2)	Methyl cinnamate		-2.139		-1.055	SM
Lmsn003318(C16H20O9)	1-O-Feruloyl-β-D-glucose		1.313	1.293		SM
Lmsn003628(C23H32O15)	6′-O-Sinapoylsucrose		1.620		1.711	SM
Lmsp003655(C26H32O11)	Dehydrodiconiferyl alcohol-gamma'-O-glucoside		-2.861		-2.438	SM
Lmsp102510(C14H14O4)	Torachrysone			1.548	-1.821	SM
Lmtn002324(C19H28O11)	Benzyl-(2''-O-glucosyl)glucoside		3.834	1.739	2.507	SM
Lmtn002565(C14H18O9)	1-O-vanilloyl-D-glucose	-2.301	2.950			SM
Lmtn003598(C18H24O8)	3-Prenyl-4-O-glucosyloxy-4-hydroxybenzoic acid		1.406			SM
Lmyn003028(C19H28O11)	Benzyl-β-gentiobioside		4.411	1.395	2.989	SM
Lmzn001925(C12H16O5)	3,4'-Dihydroxy-3'-methoxybenzenepentanoic acid			1.491	-1.103	SM
MA10107783(C10H8O5)	3-[(1-Carboxyvinyl)oxy]benzoic acid		1.618		1.201	SM

mad2085(C22H18O11)	Caffeoyl-p-coumaroyltartaric acid				-3.006	SM
ML10179289(C8H10O)	2-Phenylethanol				-1.234	SM
mws0014(C10H10O4)	Ferulic acid		1.230			SM
mws0027(C9H10O5)	Syringic acid		1.684		1.552	SM
mws0093(C10H12O3)	Coniferyl alcohol		1.076		1.198	SM
mws0180(C7H6O4)	2,5-Dihydroxybenzoic acid; gentisic acid		3.594		3.411	SM
mws0183(C7H6O4)	3,4-Dihydroxybenzoic acid (Protocatechuic acid)		4.283		3.953	SM
mws0458(C8H8O3)	Vanillin		1.355			SM
mws0467(C9H10O3)	3-(4-Hydroxyphenyl)-propionic acid	-1.093		-1.050		SM
mws0628(C7H6O2)	4-Hydroxybenzaldehyde		1.266			SM
mws0639(C7H6O4)	2,3-Dihydroxybenzoic acid		4.159		3.826	SM
mws0749(C7H6O3)	4-Hydroxybenzoic acid		1.881		1.160	SM
mws0853(C11H14O4)	Sinapyl alcohol		2.748		2.810	SM
mws0906(C16H22O8)	Coniferyl alcohol-4-O-glucoside (Coniferin)			1.652	-1.410	SM
mws1024(C9H8O2)	p-Coumaraldehyde		1.213			SM
mws1150(C35H46O20)	Echinacoside			1.474	-1.731	SM
mws1195(C10H10O3)	p-Coumaric acid methyl ester	-1.116		-1.611		SM
mws1200(C10H10O3)	Trans-4-Hydroxycinnamic acid methyl ester	-1.106				SM
mws1297(C13H18O6)	Benzyl glucoside		2.436	1.298	1.432	SM
mws1336(C7H7NO2)	4-Aminobenzoic acid		-1.452		-1.173	SM
mws1350(C9H10O4)	Syringaldehyde; 4-hydroxy-3,5-dimethoxybenzaldehyde		2.186		1.354	SM
mws1686(C9H10O3)	Paeonol		1.063	1.018		SM
MWS1848(C8H8O2)	Phenyl acetate		1.387		1.479	SM
mws2212(C9H8O4)	Caffeic acid		1.147			SM
mws2213(C9H8O2)	Cinnamic acid				-1.605	SM
mws4085(C11H12O5)	Sinapic acid		1.084		1.341	SM
MWSmce177(C12H14O4)	Ethyl ferulate		-1.514	-1.102		SM
MWSmce270(C20H26O5)	Rosmanol		1.673		2.284	SM
MWSmce294(C12H16O3)	Elemicin				2.152	SM
MWSmce364(C10H12O4)	Xanthoxylin		1.770		1.663	SM
MWSmce466(C8H8O2)	4-Hydroxyacetophenone		1.596		1.617	SM
MWSmce490(C8H8O4)	Phloracetophenone	-1.733	4.914		4.178	SM
MWSmce501(C8H8O4)	Methyl 3,4-dihydroxybenzoate		10.497		10.497	SM
MWSmce632(C10H10O4)	Vanillin acetate	-2.026		-1.724		SM
MWSslk084(C10H14O2)	4-Butylresorcinol		1.530	1.447		SM
NK10253223(C8H9NO3)	2-Amino-3-methoxybenzoic acid		-2.986		-2.709	SM
NK10264324(C6H6O3)	1,3,5-Benzenetriol		1.064			SM
pmb0751(C16H16O7)	Trans-5-O-(p-coumaroyl)shikimate		-1.645		-2.280	SM
pmb2497(C9H10O5)	4-Hydroxy-3-methoxymandelate				1.104	SM
pmb2654(C19H27NO12)	Anthranilate-1-O-sophoroside				-2.182	SM
pmb2795(C10H10O3)	4-Methoxycinnamic acid	-1.343		-1.018		SM
pmb2871(C13H16O9)	1-O-Gentisoyl-D-glucoside		2.159		1.538	SM
pmb2940(C17H22O10)	1-O-Glucosyl sinapate	1.033	2.129	1.914	1.248	SM
pmb3074(C16H18O8)	5-O-p-Coumaroylquinic acid				-1.877	SM
pmb3075(C16H16O7)	3-O-p-Coumaroylshikimic acid		-1.638	1.109	-2.292	SM
pmb3142(C13H16O8)	Salicylic acid-2-O-glucoside		-1.101		-1.187	SM
pme0422(C10H10O4)	Isoferulic acid		1.150			SM
pme0428(C12H14O5)	3,4,5-Trimethoxycinnamic acid		1.333		1.387	SM
pme2598(C8H8O4)	3,4-Dihydroxybenzeneacetic acid			1.680	-1.225	SM
pme3443(C11H12O4)	Sinapinaldehyde		2.112		1.555	SM

pme3472(C12H16O7)	Arbutin		-1.328		-1.338	SM
pmn001367(C13H16O9)	Protocatechuic acid-4-O-glucoside		2.044		1.432	SM
pmn001420(C15H18O9)	1-O-[(E)-Caffeoyl]-D-glucose			1.750	-1.702	SM
pmn001421(C16H18O8)	3-O-p-Coumaroylquinic acid				-1.510	SM
pmn001458(C22H30O14)	Sibiricose A5		1.373		1.387	SM
pmn001690(C16H24O7)	3-Hydroxy-4-isopropylbenzylalcohol-3-O-glucoside		-1.575	1.443	-2.420	SM
pmn001695(C16H20O10)	Trihydroxycinnamoylquinic acid		2.022		2.057	SM
Smcn000644(C14H20O9)	Leonuriside A		-1.551		-1.473	SM
Wmqn000813(C15H16O9)	Cichoriin		-2.569		-2.263	SM
Wmyn000217(C14H20O9)	2,6-dimethoxy-hydroquinone-4-O-β-D-glucopyranoside				-1.130	SM
Ymjm000141(C17H20O9)	4-O-Caffeoylquinic acid methyl ester		1.877		2.141	SM
Ymjm000142(C17H20O9)	5-O-Caffeoylquinic acid methyl ester		1.786		1.787	SM
Ymjm000144(C21H30O13)	2,3,4,5-Trihydroxybutyl 6-O-(E)-caffeoyl-β-D-glucopyranoside		15.211	11.850	3.361	SM
Ymjm000151(C16H18O9)	5-O-Caffeoylquinic acid	10.278			12.722	SM
Ymjm000152(C12H16O7)	p-Hydroxypheny-β-D-allopyranoside		-1.439		-1.476	SM
Zmhn000892(C13H18O8)	4-O-Glucosyl-3,4-dihydroxybenzyl alcohol		-1.332			SM
Zmhn001358(C13H16O8)	4-O-Glucosyl-4-hydroxybenzoic acid		15.476		15.476	SM
Zmhn001793(C15H18O9)	6-O-Caffeoyl-D-glucose		-1.018	1.689	-2.123	SM
Zmhn001926(C13H16O8)	1-O-Salicyl-D-glucose		4.360		4.163	SM
Zmhn002032(C28H34O15)	4-O-(6'-O-Glucosylcaffeoylglucosyl)-4-hydroxybenzyl alcohol		2.383		2.538	SM
Zmhn002227(C17H22O10)	Sinapoyl-4-O-glucoside		-1.411	1.276	-1.940	SM
Zmhn002301(C15H18O8)	p-Coumaric acid-4-O-glucoside		-1.029			SM
Zmhn002508(C21H28O12)	4-p-Cumaroyl-rhamnosyl-(1→6)-D-glucose			1.298	-1.741	SM
Zmln000899(C15H20O9)	Syringaldehyde-4-O-glucoside		1.033			SM
Zmtn001661(C14H20O8)	Vanilloloside		2.391		1.441	SM
Zmzn001549(C15H18O8)	4-O-beta-D-glucosyl-4-coumaric acid		-1.377			SM
**Flavonoids**						
Chalcones						
Lmjp003722(C20H22O7)	2'-Hydroxy-3,4,5,4',6'-pentamethoxychalcone		-2.567		-2.099	SM
MWSslk181(C21H22O9)	Isoliquiritin			1.846	-1.017	SM
pmp000801(C19H20O4)	Isobavachalcone A		3.491		2.121	SM
pmp000802(C19H20O4)	Isobavachalcone B		3.500		2.127	SM
Flavanones						
HJAP020(C27H32O16)	Eriodictyol-5,3'-Di-O-glucoside		-2.272	-1.917		SM
MWSHY0138(C21H22O4)	Bavachinin		1.155			SM
pmb2979(C25H26O14)	Hesperetin-7-O-(6"-malonyl)glucoside		-1.058		-1.273	SM
pmb2984(C23H24O12)	Eriodictyol-7-O-(6"-acetyl)glucoside		10.193		10.193	SM
pmp000637(C20H20O5)	Sophoraflavanone B		2.769		1.701	SM
Smln011469(C33H26O10)	2,3-Dihydrosciadopitysin				-2.779	SM
Wmkn003942(C21H22O6)	2'-HydroxyIsoxanthohumol		11.562		11.562	SM

Flavanonols					1 vs 0	
mws0914(C15H12O5)	Pinobanksin				1.562	SM
Flavones						
mws0043(C21H22O8)	Nobiletin (5,6,7,8,3',4'-Hexamethoxyflavone)				-1.404	SM
mws0055(C20H20O7)	Tangeretin (4',5,6,7,8-Pentamethoxyflavone)	-1.679				SM
mws1313(C20H20O7)	Sinensetin (5,6,7,3',4'-Pentamethoxyflavone)				-1.545	SM
MWSHY0118(C19H18O6)	Scutellarein tetramethyl ether	-1.011			-1.151	SM
pmb0608(C25H24O14)	Chrysoeriol-7-O-(6''-malonyl)glucoside		-2.094		-1.816	SM
pmb0736(C23H24O12)	Tricin-7-O-glucoside		-2.668		-2.305	SM
pmp001076(C20H20O7)	Isosinensetin				-2.083	SM
Zmjp004852(C23H24O13)	5,6,3′,4′-Tetrahydroxy-3,7-dimethoxyflavone-6-O-glucoside		12.104		12.104	SM
Zmjp006969(C17H14O6)	Pectolinarigenin		10.714		10.714	SM
Zmsp007375(C20H20O7)	3,5,7,3′4′-Pentamethoxyflavone				-1.228	SM
Flavonols						
Li512111(C24H24O13)	Isorhamnetin-3-O-(6''-acetylglucoside)				-2.320	SM
mws0038(C17H14O6)	Kumatakenin		2.347		1.880	SM
pmn001551(C21H22O9)	Natsudaidain (3-Hydroxy-3',4',5,6,7,8-hexamethoxyflavone)		4.886	2.991	1.480	SM
pmp000976(C27H30O10)	Baohuoside I; Icariside II			-1.112		SM
Flavonoid carbonoside			1 vs 1		1 vs 1	
mws1073(C27H30O15)	Apigenin-6,8-di-C-glucoside (Vicenin-2)		1.431		1.817	SM
pmp000236(C26H28O14)	Isovitexin-8-O-xylcoside		-1.298		-1.112	SM
Flavanols						
Cmsp007228(C16H14O6)	7-O-Methyleriodictyol		-1.105			SM
mws0355(C22H18O10)	Catechin gallate	-1.902		-2.527		SM
mws1397(C22H18O10)	Epicatechin gallate	-11.129	11.304		11.304	SM
pmn001527(C22H32O12)	Di-O-galloyl methyl gallate		-11.000		-10.520	SM
Isoflavones						
Lmgp004829(C23H24O12)	5,7,4'-Trihydroxy-6,8-dimethoxyisoflavone-7-O-galactoside	-1.964	-7.808		-10.310	SM
**Quinones**						
Anthraquinone						
pmn001340(C23H22O11)	Emodin-8-O-(6''-O-acetyl)glucoside		-1.046		-1.306	SM
pmp000601(C19H18O7)	Chryso obtusin				-1.025	SM
Zmdn006214(C24H24O11)	Physcion-8-0-(6-acetyl)-glucoside	-1.063		-1.376		SM
**Lignans and coumarins**						
Lignans			9 vs 6	4 vs 2	8 vs 5	
Hmcn002743(C22H26O8)	Lirioresinol A		2.872		2.074	SM
Hmln002355(C27H34O12)	5'-Methoxymatairesinoside		-1.315		-1.031	SM
HX1288(C19H30O8)	Citroside A		1.004	-1.148	1.458	SM
HX1369(C20H24O6)	(-)-Lariciresinol		-1.860		-1.625	SM

HX1371(C20H24O6)	(7S,8R)-dihydrodehydrodiconferyl alcohol		-1.846		-2.005	SM
Lmmn002260(C27H36O12)	5'-Methoxyisolariciresinol-9'-O-glucoside		-2.089		-2.667	SM
Lmnn102709(C26H34O12)	(-)-Olivil 4'-O-bate-D-glucopyranoside		-1.734		-1.328	SM
Lmqn001932(C28H36O13)	Syringaresinol-4'-O-glucoside		1.348		1.142	SM
Lmtn003096(C26H36O11)	Secoisolariciresinol 4-O-glucoside		12.351	11.306		SM
MWS20152(C22H26O8)	Syringaresinol		2.951		2.012	SM
mws2162(C24H32O7)	Schisandrol A		4.483	1.580	2.177	SM
Qmzp101909(C21H22O7)	Magnolone		8.782	7.649	1.133	SM
Rfmb26201(C30H38O14)	Syringaresinol-4'-O-(6"-acetyl)glucoside		2.929		1.780	SM
Ymjm000135(C25H32O11)	8R-Dihydrodehydrodiconferyl Alcohol 4-O-β-D-glucopyranoside		-1.632	-2.100		SM
Zmhn001446(C28H36O13)	Syringaresinol-4'-O-glucopyranosid		2.614	1.231	1.518	SM
Coumarins						
Cmpp004143(C26H34O9)	Deacetylnomilinic acid			2.028	-1.338	SM
Cmpp004617(C26H32O8)	Deacetylnomilin			1.920	-1.352	SM
Cmpp004946(C26H32O8)	Isoobacunoic acid			1.825	-1.380	SM
Cmpp005475(C15H16O4)	Meranzin		4.816		5.156	SM
Cmpp007319(C15H16O4)	7-Methoxy-5-prenyloxycoumarin				1.092	SM
Cmpp007452(C15H16O4)	Auraptenol		1.239			SM
Cmyn001328(C15H16O9)	Daphnin		-2.873		-2.801	SM
Hmcp002123(C10H8O4)	Isoscopoletin (6-Hydroxy-7-methoxycoumarin)		1.681		1.508	SM
HX1363(C10H8O4)	Isoscopoletin		1.389		1.167	SM
HX1386(C15H14O4)	Braylin			-1.119		SM
HX1388(C15H14O4)	Xanthoxyletin				1.257	SM
HX1389(C15H14O4)	5-Methoxyseselin				1.112	SM
Lhqp101815(C11H6O3)	Bakuchicin		-2.314		-1.107	SM
Lmbn001162(C15H16O9)	Esculetin-7-O-glucoside		-1.306		-1.756	SM
Lmbp002255(C16H18O10)	Fraxetin-8-O-glucoside (Fraxin)	-1.192			-1.343	SM
Lmbp003208(C17H20O10)	Fraxidin-8-O-glucoside	1.042	1.622	3.006		SM
Lmjp003090(C17H20O10)	Isofraxidin-7-O-glucoside		2.756	1.612	1.193	SM
Lmjp101203(C15H14O4)	Phebalosin		-1.524			SM
Lmjp101205(C16H18O4)	8-Isopentenyllimettin			-1.055		SM
Lmjp101207(C15H16O5)	(S)-Murpanidin		1.280		1.030	SM
Lmwp102713(C14H16O5)	(S)-Peucedanol		2.231		2.082	SM
mws0139(C10H8O3)	7-Hydroxy-4-methylcoumarin		2.016			SM
mws0987(C10H8O2)	6-methylcoumarin		-1.239			SM
mws1015(C15H16O9)	Esculin (6,7-Dihydroxycoumarin-6-glucoside)		-2.613		-2.540	SM
mws1074(C9H6O4)	Daphnetin		1.040			SM
mws1639(C11H10O5)	Isofraxidin		2.958		1.933	SM
mws1668(C11H6O3)	Angelicin				1.245	SM
MWSCX014(C10H8O4)	Scopoletin (7-Hydroxy-6-methoxycoumarin)		1.492		1.298	SM
MWSmce082(C11H10O4)	Scoparone				1.763	SM
MWSmce160(C14H14O3)	Demethylsuberosin		-1.336		-1.311	SM
MWSmce224(C19H22O3)	Auraptene	-1.366	-4.378	-5.790		SM
MWSmce324(C11H6O3)	Psoralen		1.292		1.413	SM
MWSmce439(C9H8O2)	3,4-Dihydrocoumarin		1.392			SM
pmf0526(C16H14O4)	Isoimperatorin		-17.045			SM
pmp000022(C16H16O6)	Oxypeucedanin hydrate			-1.622	1.734	SM

pmp000284(C11H10O5)	Fraxidin (8-Hydroxy-6,7-dimethoxycoumarin)		3.190		1.974	SM
Qmzp101840(C19H22O4)	Epoxyaurapten		1.572		1.093	SM
Zmnn010135(C15H16O4)	Sibiricol		1.344		1.210	SM
**Alkaloids**						
Pyrrole alkaloids						
MWStz014(C13H11NO)	1-Cinnamoylpyrrole				1.674	SM
Benzylphenylethylamine alkaloids						
MWSslk106(C8H11N)	2-Phenylethylamine		-1.296		-1.593	SM
Phenolamine						
Lmgp000796(C8H7NO2)	4-Hydroxymandelonitrile		-1.177		-1.607	SM
Lmhp005872(C25H30N2O6)	Diferuloylcadaverine			2.212	-1.377	SM
MWSmce089(C17H17NO3)	p-Coumaroyltyramine		7.039	3.450	3.236	SM
pma0702(C29H39N3O8)	N1,N8-Bis(sinapoyl)spermidine		-2.157		-1.879	SM
pmb0492(C34H37N3O6)	N',N'',N'''-p-Coumaroyl-cinnamoyl-caffeoyl spermidine	-1.169	-1.259	-2.164		SM
Quinoline alkaloids						
Lmwp102905(C15H17NO2)	4-methoxy-3-(3-methylbut-2-en-1-yl)quinolin-2-ol	-1.577	4.685		2.938	SM
Lmyp003387(C10H9NO3)	2-Oxo-3,4-dihydro-1H-quinoline-3-carboxylic acid			-1.060		SM
MA10074217(C9H7NO)	4-Hydroxyquinoline		1.524		1.110	SM
pmp001099(C15H17NO3)	Platydesmine	-1.435	6.261		3.775	SM
Zmnn009241(C16H15NO5)	Citpressine I		4.319		2.643	SM
Zmnn011234(C20H21NO5)	Buntanine	-1.057	-2.690			SM
Zmnn011624(C20H19NO5)	Honyumine	-1.555	3.562	-1.364	3.372	SM
Piperidine alkaloids						
MWSmce460(C5H9NO)	2-Piperidone		1.347			SM
pmb0782(C5H11N)	Piperidine				-1.779	SM
pmp001198(C6H13NO2)	6-Deoxyfagomine				-1.780	SM
Alkaloids						
Hmtp000776(C8H9NO3)	4,5,6-Trihydroxy-2-cyclohexen-1-ylideneacetonitrile				-1.033	SM
Lmmp002080(C11H16N2O)	N-(4-Aminobutyl)benzamide		-1.389			SM
Lmtn001569(C20H29NO12)	2-Glucosyl-glucosyloxy-2-phenylacetic acid amide		-1.071		-1.121	SM
mws0704(C2H8NO4P)	O-Phosphorylethanolamine		1.012			SM
mws1375(C12H21N3O6)	Nicotianamine		-3.369		-4.290	SM
MWSmce331(C4H7NO2)	Azetidine-2-carboxylic acid		-1.891		-2.255	SM
MWSmce461(C4H7NO2)	L-Azetidine-2-carboxylic acid		-1.744		-2.105	SM
MWSmce526(C6H12N2O3)	Daminozide			1.403	-1.864	SM
MWSmce548(C5H11NO2)	Betaine		2.707		2.600	SM
pme1841(C5H14N2)	Cadaverine			-1.674		SM
pme2292(C4H12N2)	Putrescine		1.171		2.217	SM
pmp001083(C34H53N7O9)	Citrusin I		-1.184		-1.256	SM
pmp001269(C18H39NO)	Hexadecyl ethanolamine		11.198	9.534	1.664	SM
pmp001287(C8H9N)	N-Benzylmethylene isomethylamine				-1.040	SM
Qmgp082709(C16H15NO5)	Citrusinine I	-3.030	3.318	-2.096	2.384	SM
Qmgp082711(C19H17NO4)	5-Hydroxynoracronycine	-1.871	4.098	-1.701	3.927	SM

Qmgp082713(C19H19NO4)	Junosine	-1.577	3.507	-1.740	3.670	SM
Qmhp090808(C29H28N2O4)	18-Demethylparaensidimerin C		-1.551		-1.154	SM
Zmzp005934(C18H37NO)	Stearamide			1.055		SM
Isoquinoline alkaloids						
MWSmce340(C12H14N2O)	L-Praziquanamine				1.176	SM
pmp001101(C15H15NO2)	N-Methylflindersine		11.742		11.742	SM
Plumerane						
Hmmp001310(C11H9NO2)	3-Indoleacrylic acid		1.460		1.070	SM
Lmyn002540(C16H19NO9)	Dioxindole-3-acetyl-3-O-glucoside			-1.272		SM
ML10198935(C11H14N2O)	3-(2-(Methylamino)ethyl)-1H-indol -5-ol	-1.552	5.361		4.181	SM
mws0102(C9H7NO2)	Indole-5-carboxylic acid		1.100			SM
mws0103(C9H7NO)	Indole-3-carboxaldehyde		1.513		1.014	SM
mws1417(C9H7NO2)	Indole-3-carboxylic acid		1.709			SM
MWStz009(C13H11NO)	Glycozolinine				1.815	SM
MWStz012(C13H11NO)	3-Methyl-9H-carbazol-2-ol				1.053	SM
MWStz323(C14H19NO2)	Paniculidine B		-1.037	-1.169		SM
pmb0818(C11H11NO3)	Methoxyindoleacetic acid		1.693		1.126	SM
pmb1096(C8H7N)	Indole		1.124			SM
Zmbp002538(C11H12N2O2)	1-Methoxy-indole-3-acetamide		1.563		1.089	SM
**Terpenoids**						
Triterpene						
HJN110(C30H48O3)	Betulinic acid		-1.148			SM
Lmmn008971(C30H48O3)	Isomangiferolic acid		-1.081			SM
Lmmn009170(C30H48O3)	Mangiferolic acid		-1.033			SM
Lmsn011015(C30H46O5)	1-Oxo-Siaresinolic acid		-1.050			SM
Lmsn011201(C30H46O5)	Swinhoeic acid		-1.039			SM
Lmsn011830(C30H46O5)	2α,19α-Dihydroxy-3-oxours-12-en-28-oic acid		1.146		1.643	SM
Lmzn006024(C30H44O5)	Zizyberanal acid		1.360		1.775	SM
Lmzn006284(C30H48O4)	2α-Hydroxyursolic acid			-1.260		SM
Lmzn106284(C30H48O4)	Alphitolic acid			-1.320		SM
Lmzp003694(C26H32O8)	Obacunoic acid		-1.430	-1.638		SM
mws1610(C30H48O4)	Maslinic acid			-1.268		SM
MWSmce052(C30H48O3)	3-Epiursolic acid	-1.510				SM
pme3544(C26H30O8)	Limonin		-1.157	-1.736		SM
pmn001591(C30H48O5)	Rutundic acid		1.679		1.969	SM
pmn001700(C30H48O3)	24,30-Dihydroxy-12(13)-enolupinol		1.898		2.003	SM
pmn001706(C30H48O4)	2-Hydroxyoleanolic acid			-1.216		SM
pmp000529(C30H46O4)	Melianone		-1.040	-1.672		SM
Smpn009230(C30H48O5)	2α,3α,23-Trihydroxyolean-12-en-28 -oic acid		1.218		1.229	SM
Zjmp102703(C29H44O5)	Norarjunolic acid		-1.359	-1.498		SM
**Others**						
Others						
Cmsp003083(C26H32O11)	Dehydrodiconiferyl alcohol-4-O-glucoside		-2.629		-2.113	SM
HJAP158(C19H21NO4)	Aromatide		4.188		3.101	SM
Hmcn000773(C21H32O14)	6'-O-Glucosylaucubin				-1.554	SM
Jmbn003691(C15H20O7)	3,4-dihydroxy-allylbenzene-3-O-β-D-glucopyranoside		2.187		1.771	SM
Lhhp102901(C20H24O6)	3-(α,4-Dihydroxy-3-methoxybenzyl)-4-(hydroxy-3-methoxybenzyl)		-1.649		-1.639	SM

Lmcp005089(C18H16O5)	6,8-Dihydroxy-2-[2-(4-methoxyphenyl)ethyl]chromone		-2.621		-1.723	SM
Lmjp004702(C9H10O4)	2',4'-Dihydroxy-6'-methoxyacetophenone		6.321		3.853	SM
Lmzn003953(C28H36O10)	Nomilinic acid			-1.724	1.054	SM
pmp000292(C15H16O5)	Hamaudol		2.239	1.186	1.400	SM
pmp000309(C22H28O11)	Cimifugin-7-O-glucoside		-1.562			SM
pmp001045(C15H22O8)	Bartsioside		-2.414		-1.770	SM
pmp001229(C12H16O3)	Senkyunolide K				1.935	SM
pmp001236(C11H10O4)	Clove chromone	-1.110	2.489			SM
Zjbn102516(C15H16O3)	3,3'-Dihydroxy-5-methoxybibenzyl		2.076		1.587	SM
Zmdn004863(C13H12O4)	Aloesone		5.013	1.807	3.303	SM
Zmxn110201(C14H20O9)	2,6-Dimethoxy-4-hydroxyphenol-1-O-ß-D-glucopyranoside		-1.651		-1.551	SM
Saccharides and alcohols						
Lmbn000198(C4H6O5)	3-Dehydro-L-Threonic Acid				1.071	PM
mws0214(C6H14O6)	D-Sorbitol		1.889		1.971	PM
mws0866(C6H13O9P)	D-Glucose 6-phosphate				-1.701	PM
mws0889(C4H8O5)	D-Threonic acid				-1.563	PM
mws1090(C6H13O9P)	Glucose-1-phosphate		-1.032		-1.791	PM
mws1164(C6H12O6)	D-Fructose		1.207			PM
mws2523(C12H23O14P)	Trehalose 6-phosphate			1.198	-1.533	PM
mws4170(C6H12O6)	D-Glucose		1.172			PM
MWSslk235(C6H14O6)	Allitol		2.241		1.306	PM
pma6455(C5H11O8P)	Ribulose-5-phosphate			1.628	-1.711	PM
pme2237(C6H14O6)	Dulcitol		2.003		2.369	PM
pme3311(C6H14O12P2)	D-Fructose-1,6-biphosphate		11.622		11.622	PM
pme3313(C6H13O9P)	D-Fructose 6-phosphate		-1.146	1.037	-2.085	PM
pmf0138(C6H12O6)	D-Mannose		1.073			PM
Zmpn000095(C6H15O9P)	Sorbitol-6-phosphate				-1.666	PM
Zmpn000199(C6H10O8)	D-Galactaric acid	1.104	2.025		3.449	PM
Zmyn000083(C6H14O12P2)	D-Glucose 1,6-bisphosphate			1.216		PM
Zmyn000108(C6H10O8)	D-Saccharic acid	-1.399	-1.701		-3.170	PM
Zmzn000078(C3H7O6P)	Dihydroxyacetone phosphate			1.353		PM
Zmzn000079(C4H9O7P)	D-Erythrose-4-phosphate			1.247	-1.642	PM
Vitamin						
mws0133(C6H6N2O)	Nicotinamide		-1.277		-1.586	PM
mws1337(C9H17NO5)	D-Pantothenic acid		-1.179		-1.194	PM
MWSmce278(C21H26O3)	Acitretin		3.314		2.154	PM
pma1751(C12H15NO7)	N-(beta-D-Glucosyl)nicotinate		13.311		6.390	PM
pme0490(C6H5NO2)	Nicotinic acid (Vitamin B3)	-1.548				PM
pme1306(C8H12NO6P)	Pyridoxine-5'-phosphate		11.994		11.994	PM
pme2266(C10H16N2O3S)	Biotin		-11.447	-1.182	-10.807	PM
Zmjp000624(C8H9NO3)	Pyridoxal		-2.238		-2.222	PM

PM, primary metabolite; SM, secondary metabolite.
